# The assembled and annotated genome of the masked palm civet (*Paguma larvata*)

**DOI:** 10.1093/gigascience/giac041

**Published:** 2022-05-18

**Authors:** Ping Liu, Hai-Ying Jiang, Lin-Miao Li, Jia-Bin Zhou, Wen-Zhong Huang, Jin-Ping Chen

**Affiliations:** Guangdong Key Laboratory of Animal Conservation and Resource Utilization, Guangdong Public Laboratory of Wild Animal Conservation and Utilization, Institute of Zoology, Guangdong Academy of Sciences, Guangzhou 510260, China; Guangdong Key Laboratory of Animal Conservation and Resource Utilization, Guangdong Public Laboratory of Wild Animal Conservation and Utilization, Institute of Zoology, Guangdong Academy of Sciences, Guangzhou 510260, China; Guangdong Key Laboratory of Animal Conservation and Resource Utilization, Guangdong Public Laboratory of Wild Animal Conservation and Utilization, Institute of Zoology, Guangdong Academy of Sciences, Guangzhou 510260, China; Guangdong Key Laboratory of Animal Conservation and Resource Utilization, Guangdong Public Laboratory of Wild Animal Conservation and Utilization, Institute of Zoology, Guangdong Academy of Sciences, Guangzhou 510260, China; Guangdong Key Laboratory of Animal Conservation and Resource Utilization, Guangdong Public Laboratory of Wild Animal Conservation and Utilization, Institute of Zoology, Guangdong Academy of Sciences, Guangzhou 510260, China; Guangdong Key Laboratory of Animal Conservation and Resource Utilization, Guangdong Public Laboratory of Wild Animal Conservation and Utilization, Institute of Zoology, Guangdong Academy of Sciences, Guangzhou 510260, China

**Keywords:** masked palm civet, genome assembly, Hi-C proximity mapping, gene family evolution, phylogeny, positive selection

## Abstract

**Background:**

The masked palm civet (*Paguma larvata*) acts as an intermediate host of severe acute respiratory syndrome coronavirus (SARS-CoV), which caused SARS, and transfered this virus from bats to humans. Additionally, *P. larvata* has the potential to carry a variety of zoonotic viruses that may threaten human health. However, genome resources for *P. larvata* have not been reported to date.

**Findings:**

A chromosome-level genome assembly of *P. larvata* was generated using PacBio sequencing, Illumina sequencing, and Hi-C technology. The genome assembly was 2.44 Gb in size, of which 95.32% could be grouped into 22 pseudochromosomes, with contig N50 and scaffold N50 values of 12.97 Mb and 111.81 Mb, respectively. A total of 21,582 protein-coding genes were predicted, and 95.20% of the predicted genes were functionally annotated. Phylogenetic analysis of 19 animal species confirmed the close genetic relationship between *P. larvata* and species belonging to the Felidae family. Gene family clustering revealed 119 unique, 243 significantly expanded, and 58 significantly contracted genes in the *P. larvata* genome. We identified 971 positively selected genes in *P. larvata*, and one known human viral receptor gene *PDGFRA* is positively selected in *P. larvata*, which is required for human cytomegalovirus infection.

**Conclusions:**

This high-quality genome assembly provides a valuable genomic resource for exploring virus–host interactions. It will also provide a reliable reference for studying the genetic bases of the morphologic characteristics, adaptive evolution, and evolutionary history of this species.

## Introduction

In human history, the spread of viruses has caused huge losses to the human economy and threatened the safety of human life and property. Diseases affecting animals of economic value, caused by viruses such as avian influenza and African swine fever, have caused serious economic losses [1–3]. Viruses such as severe acute respiratory syndrome coronavirus (SARS-CoV), Middle East respiratory syndrome coronavirus (MERS-CoV), and severe acute respiratory syndrome coronavirus 2 (SARS-CoV-2), pose a great threat to the safety of human life and property [4–6]. Usually, zoonotic viruses can be transmitted to humans via wildlife as intermediate hosts. For example, MERS-CoV is transmitted from camels to humans [7].

Masked palm civets, *Paguma larvata* (NCBI:txid9675) (Mammalia, Carnivora, and Viverridae), are wild mammals widely distributed in Asia [8]. They are omnivores, although they mainly consume fruits. In recent decades, *P. larvata* have been domesticated as meat for human beings, increasing the possibility of transferring pathogenic microorganisms to human beings. In 2003, *P. larvata* gained attention because of its potential involvement in the outbreak of SARS, which originated in southern China and spread to over 30 countries [9]. Further research suggested that *P. larvata* acted as an intermediate host of SARS-CoV, which transmited this virus from bats to humans [10, 11]. The sequence similarity between the SARS-CoV found in *P. larvata* and human SARS-CoV is greater than 99%. However, *P. larvata* can live in harmony with SARS-CoV, whereas humans have severe symptoms following SARS-CoV infection. This shows that the interaction between *P. larvata* and viruses has a certain particularity [12]. Earlier studies on farms raising *P. larvata* also found that these animals rarely become ill. The immune system of *P. larvata* is thus worthy of in-depth study.

Virus receptors, present on the host cell membrane, are important factors in mediating virus invasion and play an extremely important role in the interaction between host cells and viruses. The virus attachment protein can be regarded as the “key” that unlocks the cell by interacting with the “lock” (receptor) on the surface of the host cell, starting a key downstream step in the virus life cycle. In many cases, viruses use multiple receptors to perform different functions during the viral life cycle [13]. Therefore, the virus must first find the lock and then use a specific virus “key” to unlock the cell. The numerous functions of the virus receptor aid the virus in targeting the correct tissue for infection and to cross the cell barrier, which is necessary to deliver the viral genome to the host cell [14]. The interaction with viral receptors is usually mediated by specific proteins on the surface of the virus [13]. Inherent differences in the shape of the virus surface protein (icosahedron or helix) and the composition of the virus coat affect the overall structure of the virus attachment protein. Some viruses infect only certain species; for example, swine fever virus can only infect pigs [3], whereas some viruses can spread between animals and humans, such as SARS-CoV [10, 11] and MERS-CoV [7]; the reason lies in the nature of the interaction between the virus receptor and virus attachment protein. For example, SARS-CoV and SARS-CoV-2 specifically recognize the angiotensin-converting enzyme 2 (*ACE2*) receptor [15–17]. Understanding the nature of virus receptors that cause zoonotic diseases in different species will help in tracing natural hosts during the virus epidemic.

As an important virus host, masked palm civets can be used as a system for reviewing virus and host interactions. However, at the time of writing there is no genome assembly of *P. larvata*, even in Viverridae, which has been reported for studying various virus receptors. In this study, we generated a chromosome-level reference genome of *P. larvata* by integrating PacBio sequencing, Hi-C sequencing, and Illumina sequencing [18–20]. The high-quality genome assembly will provide a valuable genomic resource for exploring virus–host interactions. It will also provide a reliable reference for studying the genetic bases of the morphologic characteristics, adaption evolution, and evolutionary history of the species.

## Data description

### Statement of ethics

The study design was approved by the Ethics Committee for Animal Experiments at the Institute of Zoology, Guangdong Academy of Sciences (reference number: GIZ20190702, 07/01/2019) and followed the basic principles outlined by this committee.

### Isolation of genomic DNA and RNA

A healthy female *P. larvata* individual was collected from a farm in Wengyuan County, Guangdong Province, China. Whole blood was isolated, then immediately frozen in liquid nitrogen and stored at −80°C prior to nucleic acid extraction. Genomic DNA was extracted from whole blood using a magnetic universal genomic DNA kit. Different tissues, including heart, intestine, kidney, liver, lung, muscle, and spleen, were sampled from the same female *P. larvata* individual and flash-frozen in liquid nitrogen for RNA sequencing (RNA-seq). RNA-seq data were used to predict genes in following analysis. Total RNA was extracted using the TRIzol reagent [21].

### Genome sequencing with different technologies

A combined sequencing strategy was used to obtain the masked palm civet genome. Short-insert libraries with a size of 350 bp were sequenced on the Illumina (San Diego, California) HiSeq 2500 platform (RRID:SCR_016383) using the 150-bp paired-end protocol. For PacBio library construction, we sheared the genomic DNA to 20 kb fragments, which we used to prepare a single-molecule real-time library for sequencing with the PacBio Sequel System (RRID:SCR_017989) [22]. Liver tissue was used to construct the Hi-C sequencing library. Briefly, cross-linked chromatin was digested with Dpn II and ligated *in situ* after biotinylation. DNA fragments were enriched via the interaction of biotin and blunt-end ligation, then subjected to Illumina NovaSeq (Illumina NovaSeq 6000 Sequencing System, RRID:SCR_016387) PE 150 sequencing [23].

### Genome assembly and quality assessment

We applied the FALCON (RRID:SCR_018804) v0.3.0 [24] to correct errors in PacBio long reads, according to PacBio short reads (<5 kb), and then generated consensus sequences. The primary scaffolds were assembled with these subreads, and the scaffolds were further corrected in PILON (RRID:SCR_014731) v1.22 [25] using short sequencing reads. To anchor scaffolds onto chromosomes, we aligned the Hi-C sequencing data to the assembly using BWA (RRID:SCR_010910) v0.7.17 [26] and detected valid contacts. Preassembled scaffolds were clustered, ordered, and directed onto pseudochromosomes using the LACHESIS software (RRID:SCR_017644) [27]. To increase the accuracy of the assembled genome, we artificially corrected the LACHESIS-based assembly, filled gaps, and removed duplicate sequences [28].

Genome size was estimated based on k-mer distribution analysis with the program in GCE (RRID:SCR_017332) [29] using Illumina short reads. We used BUSCO (RRID:SCR_015008) v5 [30] and CEGMA (RRID:SCR_015055) [31] to evaluate the completeness and accuracy of the genome assembly and BWA v0.7.17 [26] to align the Illumina short reads to the assembly and evaluate the assembled portion. We assembled expressed sequencing tags (ESTs) using Trinity (RRID:SCR_013048) v2.8.4 [32] with RNA reads from seven different tissues (heart, liver, spleen, lung, kidney, muscle, and intestine). To further examine the quality of the genome assembly, we aligned ESTs to the assembled genome using BLAT (RRID:SCR_011919) [33] with parameters of identity ≥90% and coverage ≥50%. The quality of the genome assembly was evaluated by mapping RNA-Seq reads to the ESTs using TopHat2 (RRID:SCR_013035) [34].

### Repetitive elements annotation

Repetitive sequences were identified at both the DNA and protein levels by integrating homology-based prediction and *de novo* identification. Repeat masking was performed based on repeats in the Repbase TE library from the Repbase server (RRID:SCR_021169) [35]. Using this library, we predicted interspersed repeat elements using RepeatMasker (RRID:SCR_012954) v3.3.0 and ProteinMask and screened tandem repeats using TRF v4.07b [36].

### Gene prediction and functional annotation

We annotated protein-coding genes in the *P. larvata* genome using a combination of *de novo* gene prediction, homology-based prediction, and RNA-seq–based prediction. For *de novo* identification, we predicted the gene models using five *ab initio* gene prediction programs: Augustus (RRID:SCR_008417) [37], GlimmerHMM (RRID:SCR_002654) [38], SNAP (RRID:SCR_002127) [39], GENSCAN (RRID:SCR_013362) [40], and Geneid [41]. We compared proteins from five sequenced animals, namely, *Mus musculus* [42], *Canis lupus familiaris* [43], *Felis catus* [44], *Panthera tigris altaica* [45], and *Acinonyx jubatus* [46], to the *P. larvata* assembly using TBLASTN (RRID:SCR_011822) [47] with an e-value cutoff of 1 × 10^–5^. Gene models were generated using GeneWise (RRID:SCR_015054) v2.2.0 [48]. We applied two methods of RNA-seq–based prediction: (i) we mapped RNA-seq data to the genome using TopHat2 [49] and further generated the gene models in Cufflinks (RRID:SCR_014597) [34] based on exons, and (ii) we aligned ESTs against the assembly to generate the gene models using PASA (RRID:SCR_014656) [50]. We integrated predictions from the three approaches in EVidenceModeler (RRID:SCR_014659) v1.1.1 [51] to generate a nonredundant gene model set. The final gene models were annotated using the non-redundant (NR) protein database of NCBI , Swissprot, KEGG [52], Interprot [53], and Pfam databases v14.1 [54].

Noncoding RNAs include transfer RNA (tRNA), ribsomal RNA (rRNA), microRNA (miRNA), and small nuclear RNA. tRNAscan-SE (RRID:SCR_010835) v1.4 was used to find the tRNA sequence in the genome according to the structural characteristics of tRNA. rRNA is highly conserved, and it is possible to select a closely related species as the reference sequence, and rRNA in the genome is searched by BLAST v2.2.26 [47]. The INFERNAL (RRID:SCR_011809) software that comes with Rfam (RRID:SCR_007891) v14.1 [54] was used to predict the miRNA and small nuclear RNA in the genome using the covariance model of the Rfam family.

### Phylogenetic analysis and estimation of divergence time

We used OrthoFinder (RRID:SCR_017118) v2.5.2 [55] to identify orthologous genes from masked palm civet and 18 other species, including cat (*F. catus*), cattle (*Bos taurus*), dhole (*Cuon alpinus*), dog (*C. lupus familiaris*), human (*Homo sapiens*), leopard (*Panthera pardus*), lion (*Panthera leo*), lynx (*Lynx canadensis*), mouse (*M. musculus*), opossum (*Monodelphis domestica*), panda (*Ailuropoda melanoleuca*), pig (*Sus scrofa*), polar bear (*Ursus maritimus*), rabbit (*Oryctolagus cuniculus*), red fox (*Vulpes vulpes*), tiger (*P. tigris altaica*), wolf (*Canis lupus*), and yak (*Bos mutus*) (Supplementary Table 1). Diamond was used for sequence similarity searches. We aligned the coding region and amino acid sequences of single-copy orthologous genes using MUSCLE (RRID:SCR_011812) v3.7 [56]. All alignments were concatenated to a super-alignment matrix. Based on this matrix, we used IQ-TREE (RRID:SCR_017254) v1.6.10 [57], which automatically detects the best model and then performs a maximum likelihood (ML) phylogenetic tree based on this best model. One thousand bootstrap replicates were performed to obtain confidence values for the branches. Bayesian Evolutionary Analysis Sampling Trees (BEAST, RRID:SCR_010228) v2.6.0 [58] was used to estimate species divergence times based on amino acid sequences of single-copy genes. The tree topology based on IQ-TREE v1.6.10 [56] was used as the starting tree, while splits between tiger and leopard (7.4 Ma), cat and tiger (15.2 Ma), polar bear and giant bear (23.4 Ma), pig and yak (62.0 Ma), mouse and human (90.0 Ma), and human and tiger (96.0 Ma) from TimeTree (RRID:SCR_021162) as calibration. We ran the Markov chain Monte Carlo for 10 million generations with sampling every 1,000 generations, and 25% of the generations were discarded as burn-in. TreeAnnotator in BEAST v2.6.0 [58] was used to summarize the information from trees produced by BEAST v2.6.0 [58] onto a single target tree, while the target tree was visualized and exported as vector diagrams using FigTree (RRID:SCR_008515) v1.4.4.

### Gene family expansion and contraction analysis

To identify gene family evolution as a random birth and death process model, where the gene family either expands or contracts per gene per million years independently along each lineage of the phylogenetic tree, the ML model originally implemented in the software package CAFE (RRID:SCR_005983) v4.2.1 [59] was applied to compare the cluster size differences (gain or loss) between the ancestor and each species. We used species tree with divergence time obtained by BEAST v2.6.0 [58] as an ultrametric tree. We determined gene family significantly expanded or contracted when the *P* value was less than 0.05.

### Positive selection analysis

For positive selection analysis, we first identified single-copy orthologous genes from *P. larvata, C. lupus familiaris*, and the five most closely related species from the Felidae family with assembled genomes: *F. catus, P. pardus, P. leo, L. canadensis*, and *P. tigris altaica*. For these genes, based on phylogenetic topology, we employed the branch-site model incorporated in the PAML package (RRID:SCR_014932) v4.9 [60] to detect positively selected genes (PSGs). *P. larvata* was specified as a foreground branch, and the other species were used as background branches. To determine genes under positive selection, likelihood ratio tests were conducted to determine whether positive selection was operating on the foreground branch. In this study, PSGs were identified only when *P* < 0.01. Receptors were important factors to intermediate virus infection. To study the immune mechanism of *P. larvata*, we looked at 28 known human virus receptors [61] in positive selected genes.

### Sequence analysis of virus receptors under positive selection and *ACE2*

Sequences of virus receptor genes under positive selection and the *ACE2* gene in other animal species of cat (*F. catus*), cattle (*B. taurus*), dhole (*C. alpinus*), dog (*C. lupus familiaris*), human (*H. sapiens*), leopard (*P. pardus*), lion (*P. leo*), lynx (*L. canadensis*), mouse (*M. musculus*), opossum (*M. domestica*), panda (*A. melanoleuca*), pig (*S. scrofa*), polar bear (*U. maritimus*), rabbit (*O. cuniculus*), red fox (*V. vulpes*), tiger (*P. tigris altaica*), and yak (*B. mutus*) were downloaded from NCBI (https://www.ncbi.nlm.nih.gov/). Although *ACE2* may not be positively selected in *P. larvata*, it is a receptor of SARS-CoV and SARS-CoV-2, both resulting in severe pneumonia in human beings. Therefore, we want to know the genetic relationship of virus receptors between *P. larvata* and other animals. Alignment of amino acid sequences of receptors was conducted using MUSCLE v3.7 [56]. ML phylogenetic trees were constructed using IQ-TREE v1.6.10 [57], and 1,000 bootstrap replicates were performed to obtain confidence values for the branches. The sequence identity of amino acid sequences that could demonstrate the genetic distance between receptor genes in different animals was estimated by p-distance in MEGA-X [62].

## Results and discussion

### Genome sequencing and assembly

**Figure 1:**
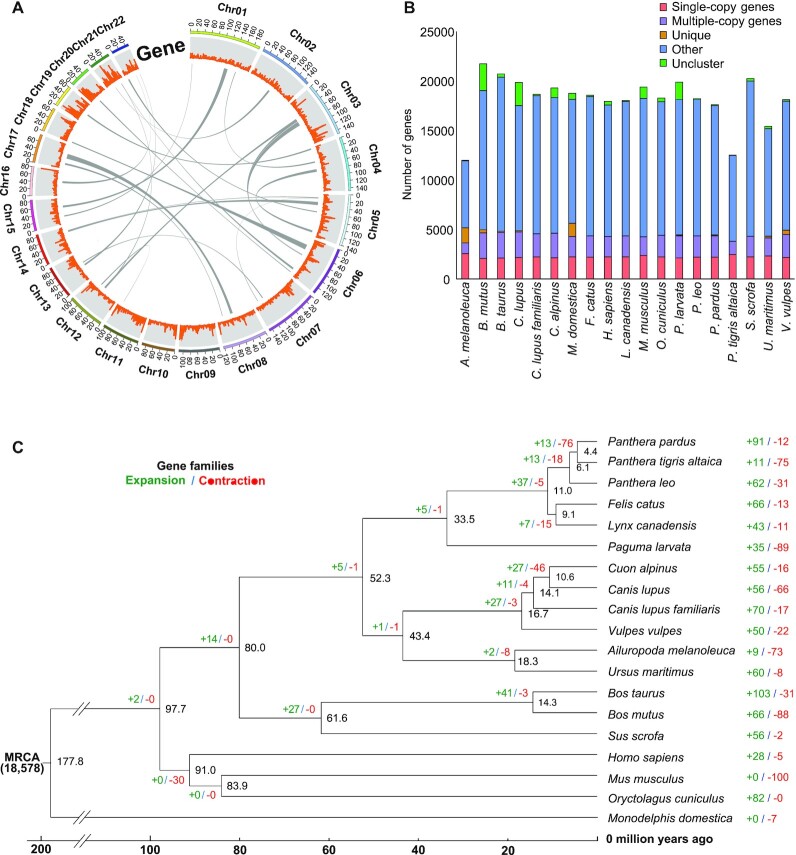
(a) Synteny of corresponding chromosomes and gene density across the *Paguma larvata* genome. (b) Comparison of copy numbers in gene clusters that reside in the genomes of *P. larvata* and 18 other animals. Single-copy orthologs denote that the family can have only one gene for each species, and multicopy orthologs denote that the family clustered more than one gene for each species. Other orthologs denote the family can have any number of genes for each species except the single-copy and multicopy orthologs. Unique paralogs denote species-specific gene families, and unclustered genes denote species-specific genes that cannot cluster with any other genes. (c) Phylogenetic relationship of 19 animal genomes based on the maximum likelihood method. The black numbers indicate estimated divergent times as the number of years (millions) ago, while green and red numbers denote gene families subject to expansion and contraction for each species, respectively.

We sequenced the genome of one female masked palm civet (2n = 44) (Fig. 1a). Here, 557.75 Gb sequences were generated from high-quality short-read sequences (272.68 Gb) and PacBio sequences (285.07 Gb), representing over 216× coverage for the genome. The genome has an estimated size of 2.58 Gb based on the 17-mer depth distribution analysis of the sequenced reads (Supplementary Fig. 1). The final assembled sequence was 2.44 Gb, representing 94.6% of the masked palm civet genome. The assembly consisted of 1,500 scaffolds with N50 of 111.81 Mb (Table 1). We used the Hi-C technology to reorder and anchor 95.32% of the genome onto 22 pseudochromosomes (Fig. 1a, Supplementary Fig. 2). The GC content and heterozygosity rate of the assembled masked palm civet genome were 42.4% and 0.46%, respectively. The completeness of our assembly was assessed by BUSCO and CEGMA. The BUSCO assessment showed that 96.5% of the 9,226 BUSCOs were assembled, with only 1.3% duplication (Supplementary Table 2), while CEGMA assessment showed that 91.94% and 3.2% of 248 core eukaryotic genes were complete and partially assembled, respectively (Supplementary Table 3). We also used BWA v0.7.17 [26] to align Illumina short reads to the assembly and obtained a mapping rate and genome coverage of 99.35% and 99.73%, respectively. Overall, all evidence suggests that the *P. larvata* assembly is a high-quality genome assembly that can be used for downstream analyses.

**Table 1:** *Paguma larvata* genome assembly and annotation summary. CDS indicates coding region sequences; LINE indicates long interspersed nuclear elements; SINE indicates short interspersed nuclear elements; while LTR indicates long terminal repeat.

Genome assembly statistics	
Total length (Gb)	2.44
Scaffold N50 length (Mb)	111.81
Scaffold N90 length (Mb)	58.03
Longest scaffold length (Mb)	195.95
Number of scaffold	1,500
Number of scaffold larger than N50	9
Number of scaffold larger than N90	20
Genome characteristics	
GC content	42.41%
Number of protein-coding genes	21,582
Average transcript length (Kb)	34.57
Average CDS length (Kb)	1.48
Average exon length (bp)	170.53
Average number of exons per gene	8.68
Repetitive sequences (% of genome)	
DNA (Mb)	8.23 (0.34%)
LINE (Mb)	299.13 (12.27%)
SINE (Kb)	613.25 (0.03%)
LTR (Mb)	542.79 (22.27%)
Unknown (Mb)	12.28 (0.5%)
Total (Mb)	775.00 (31.8%)
Gene annotations (% of all genes)	
GO	14,148 (65.6%)
InterProScan	19,770 (91.6%)
KEGG	18,200 (84.3%)
Nr	20,480 (94.9%)
PFAM	17,505 (81.1%)
SwissProt	20,033 (92.8%)
Total	20,546

CDS: coding region sequences; LINE: long interspersed nuclear elements; SINE: short interspersed nuclear elements; LTR: long terminal repeat.

### Repeat annotation and gene prediction

We analyzed repetitive sequences by combining *de novo* prediction and a homology-based search at both the DNA and protein levels and found that repetitive sequences occupied 31.80% (775.0 Mb) of the genome of the masked palm civet (Table 1), which was very similar to the repeat compositions of the cat genome (32.10%) but lower than that of the tiger genome (39.20%) [44, 45]. The most abundant repetitive element type in the masked palm civet genome was long terminal repeat, representing 542.79 Mb (22.27%) of the genome. An annotation pipeline combining *de novo*, homolog-based search, and RNA-seq methods was performed to predict gene models from the repeat-masked masked palm civet genome sequence. These analyses predicted 21,582 protein-coding genes, representing 30.61% of the genome assembly (Supplementary Table 4, Supplementary Fig. 3a). Among the 21,582 genes, 20,546 (95.20%) were successfully annotated for at least one function term by searching against functional databases (Gene Ontology, InterProScan, KEGG, NR, Pfam, and SwissProt) (Table 1, Supplementary Fig. 3b). In addition to the protein-coding genes, we also identified 14,829 miRNA genes, 47,575 tRNA genes, and 544 rRNA genes in the masked palm civet genome (Supplementary Table 5). The number of miRNA and tRNA genes seemed higher than other species. We double checked the methods and conducted noncoding RNAs prediction again and obtained the same results. Maybe the high level of miRNA and tRNA genes is the personality of this species, which need further analysis in the future.

### Gene family comparison

The evolutionary dynamics related to *P. larvata* were deduced by comparison with 10 animal species. We identified 1,438 one-to-one orthologs among these species (Fig. 1b; Supplementary Table 6). Among the 19 species, 119 genes belonging to 44 gene families were unique in *P. larvata* (Supplementary Table 6). A immune-related gene named *VSIG10L*, which is related to familial Barrett neoplasia, was contained in these genes [63]. Based on the *P* value threshold of 0.05, 35 gene families (including 243 genes) were significantly expanded; in contrast, 89 gene families (including 58 genes) were significantly contracted in the *P. larvata* genome (Fig. 1c). There were 10 immune-related genes in significantly expanded genes, which were annotated to 3 genes of *TARM1* (T cell interacting, activating receptor on myeloid cells 1), *IGLL1* (immunoglobulin lambda like polypeptide 1), and *OSCAR* (osteoclast-associated Ig-like receptor) (Supplementary Table 7). *TARM1* is a novel leukocyte receptor complex that can encode the ITAM receptor that costimulates proinflammatory cytokine secretion by macrophages and neutrophils [64]. *TARM1* enables immunoglobulin receptor binding activity. *IGLL1* is a protein-coding gene. Diseases associated with *IGLL1* include agammaglobulinemia 2, autosomal recessive, and lambda 5 deficiency [65]. *OSCAR* is a member of the leukocyte receptor complex protein family that plays critical roles in the regulation of both innate and adaptive immune responses [66]. The encoded protein may play a role in oxidative stress–mediated atherogenesis as well as monocyte adhesion. Recent studies showed that *OSCAR* can promote proliferation and migration of lung adenocarcinoma in women [67]. We did not detect immune-related genes in significantly contracted genes, which was in accordance with the high immune response level of masked palm civets.

### Phylogenetic analysis

To confirm the phylogenetic position of *P. larvata* and estimate divergence time using whole-genome data, we analyzed the orthologous gene relationships between *P. larvata* and 18 other animals (Supplementary Table 1) based on a super alignment matrix of amino acid sequences and coding region sequences of 1,438 single-copy genes with opossum as the outgroup. The best models of coding region sequences and amino acid sequences are GTR+F+R3 and JTT+F+R5. Both trees shared the same topology that the genetic relationship between *P. larvata* and five species belonging to the Felidae family was closely related. The phylogenetic tree based on amino acid sequences was used as a starting tree to calculate the divergence time, which showed that *P. larvata* split with the Felidae family approximately 33.5 million years ago (Fig. 1c). This is the first time the divergence time of masked palm civets has been obtained based on whole-genome sequences.

### Genes under positive selection

**Figure 2:**
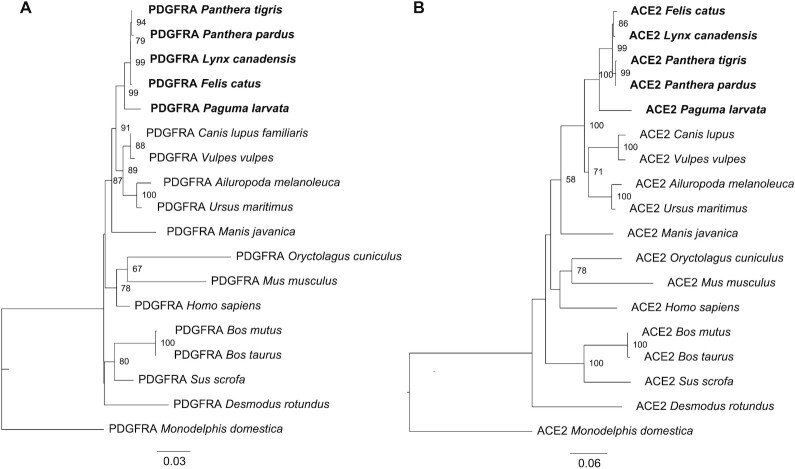
Phylogenetic relationships of known human virus receptor of *PDGFRA* under positive selection and *ACE2* receptor in *P. larvata* and other species.

We used *P. larvata* as the foreground branch and other species of *C. lupus familiaris, F. catus, P. pardus, P. leo, L. canadensis*, and *P. tigris altaica* as background branches to detect genes under selection. Based on 5,696 single-copy genes of these seven species, we identified 971 PSGs (*P* < 0.01; Supplementary Table 9). We did not observe genes under positive selection overlapped with expanded and contracted genes. One of known human viral receptor genes, *PDGFRA*, was included in genes under positive selection in *P. larvata. PDGFRA* can activate mutations in gastrointestinal stromal tumors [68] and is required for human cytomegalovirus infection [69, 70]. There has been no study focus on the *PDGFRA* receptor in masked palm civets, and further study is needed. *ACE2* is an important receptor intermediate the infection of SARS-CoV and SARS-CoV-2. Though it is not under positive selection, *ACE2* receptor was involved in this study. The phylogeny of *PDGFRA* and *ACE2* receptors from different animal species was conducted based on amino acid sequences downloaded from NCBI. The relationship of *PDGFRA* and *ACE2* in *P. larvata* was closest to four of the most closely related species from the Felidae family of *F. catus, P. pardus, L. canadensis*, and *P. tigris altaica* (Fig. 2). The amino acid sequence identities of *PDGFRA* and *ACE2* were also highest among *P. larvata, F. catus, P. pardus, L. canadensis*, and *P. tigris altaica* (Table 2), demonstrating viruses using *PDGFRA* and *ACE2* might easily transfer between these species. However, further research is needed to reveal the differences in the receptor-binding loci of these receptors in *P. larvata* and other species, as well as the immunoreaction of cells after virus infection in consideration of the different severity of symptoms in different species. Besides virus receptors, 10 immune-related genes were also under selection (Supplementary Table 10). While this article was under review, another study was published on the *P. larvata* genome [71]. On top of these efforts, further studies are needed to reveal the roles of these immune-related genes under positive selection in civet immunity.

**Table 2:** Sequence identity of receptor under positive selection and *ACE2* between masked palm civet and other animal species. The bolded values indicate the highest sequence identity.

Species	Sequence identity (%)	
	*PDGFRA*	*ACE2*
*Lynx canadensis*	**97.95**	**93.17**
*Felis catus*	**97.85**	**92.67**
*Panthera tigris altaica*	**97.85**	**92.60**
*Panthera pardus*	**97.67**	**92.80**
*Canis lupus familiaris*	96.55	88.93
*Vulpes vulpes*	96.55	88.81
*Ursus maritimus*	95.99	89.94
*Ailuropoda melanoleuca*	95.24	89.79
*Homo sapiens*	95.05	83.35
*Sus scrofa*	94.86	82.48
*Manis javanica*	93.54	85.47
*Bos taurus*	93.18	81.84
*Bos mutus*	93.09	81.84
*Desmodus rotundus*	92.16	79.35
*Mus musculus*	90.01	81.74
*Oryctolagus cuniculus*	88.14	83.11
*Monodelphis domestica*	83.49	71.14

The bolded values indicate highest sequence identity.

## Conclusion

In this study, we present a high-quality chromosome-level genome assembly of *P. larvata* using PacBio single-molecule long-read sequencing, supplemented with Illumina short-read sequencing and Hi-C sequencing. The final genome assembly was 2.44 Gb, 95.32% of which could be grouped into 22 pseudochromosomes. This assembly is highly contiguous with contig N50 and scaffold N50 values of 12.97 Mb and 111.81 Mb. The repeat sequence comprised 31.80% of the genome. A total of 21,582 protein-coding genes were predicted, 95.20% of which could be functionally annotated. Phylogenetic analysis based on 1,438 single-copy orthogroups across 19 animals resulted in a close genetic relationship between *P. larvata* and five species from the Felidae family. The divergence time of *P. larvata* split from the Felidae family was estimated to be 33.5 Ma. Gene family clustering revealed 119 unique, 243 significantly expanded, and 58 significantly contracted genes in *P. larvata* genome. We identified 971 genes under positive selection in *P. larvata*, among which one known human viral receptor gene was found. The high-quality genome assembly will provide a valuable genomic resource for elucidating the immune mechanisms in this animal. It will also provide a reliable reference for studying the genetic bases of the morphologic characteristics, adaptive evolution, and evolutionary history of this species.

## Data availability

Raw sequencing data and genome assembly were deposited at the NCBI under the BioProject PRJNA756156 with biosample accession number SAMN20856571. The annotations and predicted peptides are available in FigShare [72]. All other supporting data and materials are available in the *GigaScience* GigaDB database [73].

## Additional files

**Supplementary Figure 1**.K-mer frequency distribution curve (k = 17) of Illumina short reads of the masked palm civet genome.

**Supplementary Figure 2**. Hi-C contact data mapped to the genome of masked palm civet. The heatmap represents the normalized contact matrix. The strongest and weakest contacts are shown in red and yellow, respectively.

**Supplementary Figure 3**. Prediction and annotation of genes in masked palm civet genome. (a) Number of genes predicted with *de novo*, homolog, and RNA-seq. All predicted genes were integrated by EVM (EVidenceModeler software). (b) Number of genes annotated with databases of Swissprot, NR, GO, KEGG, Pfam, and InterPro.

**Supplementary Table 1**. List of animal genome sequences used in the comparative genomic analysis except masked palm civet.

**Supplementary Table 2**. Evaluation of the genome assembly of *P. larvata* using BUSCO.

**Supplementary Table 3**. Evaluation of the genome assembly of *P. larvata* using CEGMA.

**Supplementary Table 4**. Summary statistics of predicted protein-coding genes in *P. larvata*.

**Supplementary Table 5**. Noncoding RNAs predicted in the genome of *P. larvata*.

**Supplementary Table 6**. Gene families clustered by OrthoFinder in 19 species. Genes used for OrthoFinder were proteins without splice variants.

**Supplementary Table 7**. Genes in significant expanded gene families in masked palm civet. Yellow background indicates immunoglobulin-related genes.

**Supplementary Table 8**. Genes in significant contracted gene families in masked palm civet.

**Supplementary Table 9**. Positively selected genes in the genome of masked palm civet. Yellow background indicates immunoglobulin-related genes.

**Supplementary Table 10**. Immunoglobulin-related genes positively selected in the genome of the masked palm civet.

giac041_GIGA-D-21-00297_Original_Submission

giac041_GIGA-D-21-00297_Revision_1

giac041_GIGA-D-21-00297_Revision_2

giac041_Response_to_Reviewer_Comments_Original_Submission

giac041_Response_to_Reviewer_Comments_Revision_1

giac041_Reviewer_1_Report_Original_Submission Zhen Liu -- 11/5/2021 Reviewed

giac041_Reviewer_1_Report_Revision_1 Zhen Liu -- 2/17/2022 Reviewed

giac041_Reviewer_2_Report_Original_Submission Graham Hughes -- 11/19/2021 Reviewed

giac041_Reviewer_2_Report_Revision_1 Graham Hughes -- 2/25/2022 Reviewed

giac041_Reviewer_2_Report_Revision_2 Graham Hughes -- 3/14/2022 Reviewed

giac041_Reviewer_3_Report_Original_Submission Vineet K. Sharma -- 11/26/2021 Reviewed

giac041_Reviewer_3_Report_Revision_1 Vineet K. Sharma -- 3/8/2022 Reviewed

giac041_Supplemental_Files

## Abbreviations

BEAST: Bayesian Evolutionary Analysis Sampling Trees; bp: base pair; BUSCO: Benchmarking Universal Single-Copy Orthologs; CEGMA: Core Eukaryote Gene Mapping Approach; EST: expressed sequencing tag; kb: kilobase: KEGG: Kyoto Encyclopedia of Genes and Genomes; MERS-CoV: Middle East respiratory syndrome coronavirus; miRNA: microRNA; ML: maximum likelihood; NCBI: The National Center for Biotechnology Information; PSG: positively selected gene; RNA-seq: RNA sequencing; rRNA: ribosomal RNA; SARS-CoV: severe acute respiratory syndrome coronavirus; SARS-CoV-2: severe acute respiratory syndrome coronavirus 2; tRNA: transfer RNA.

## Conflicts of Interest

The authors declare that the research described herein was conducted in the absence of any commercial or financial relationships that could be construed as a potential conflict of interest.

## Funding

This project was supported by the surveillance of wild animals and epidemic diseases in important ecological areas of the Guangdong Province Project from the Forestry Administration of Guangdong Province, GDAS Special Project of Science and Technology Development (2020GDASYL-20200103090).
