# Supplementary material for: The assembled and annotated genome of the masked palm civet (Paguma larvata)
Source: Gigascience. 2022 May 18;11:giac041. doi: 10.1093/gigascience/giac041 (PMC9116208; doi:10.1093/gigascience/giac041)
Supplement: giac041_Supplemental_Files [file giac041_supplemental_files.zip › Supplementary Figures-20220210.docx]

**
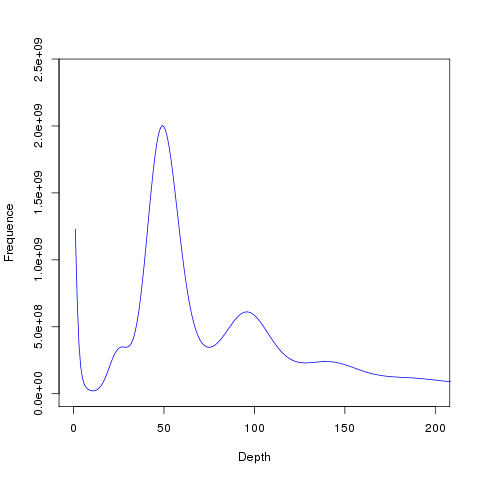
**

**Supplementary Figure S1** K-mer frequency distribution curve (k =17) of Illumina short reads of the masked palm civet genome.

**
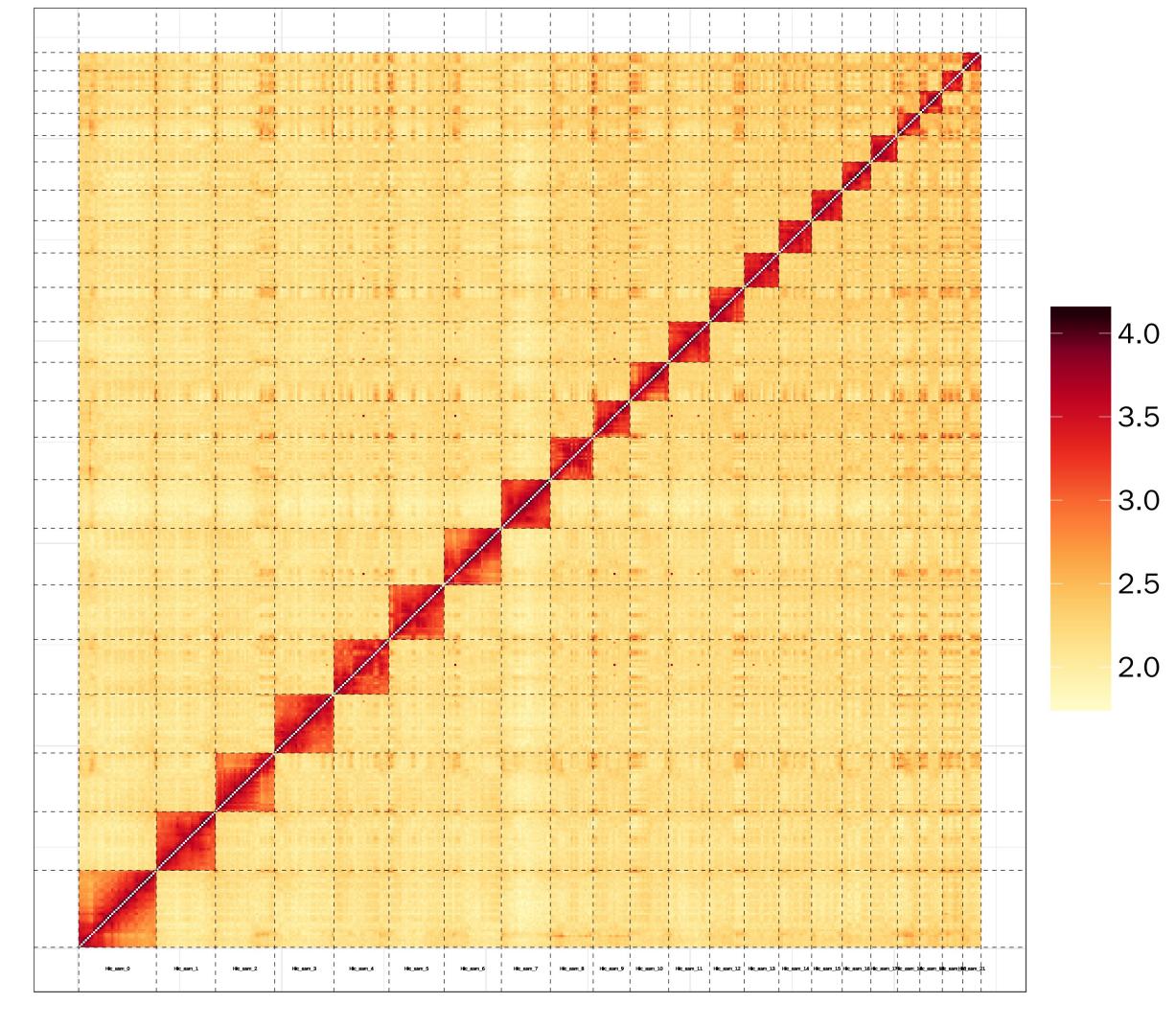
**

**Supplementary Figure S2** Hi-C contact data mapped to the genome of masked palm civet. The heat map represents the normalized contact matrix. The strongest and weakest contact are shown in red and yellow, respectively.

**
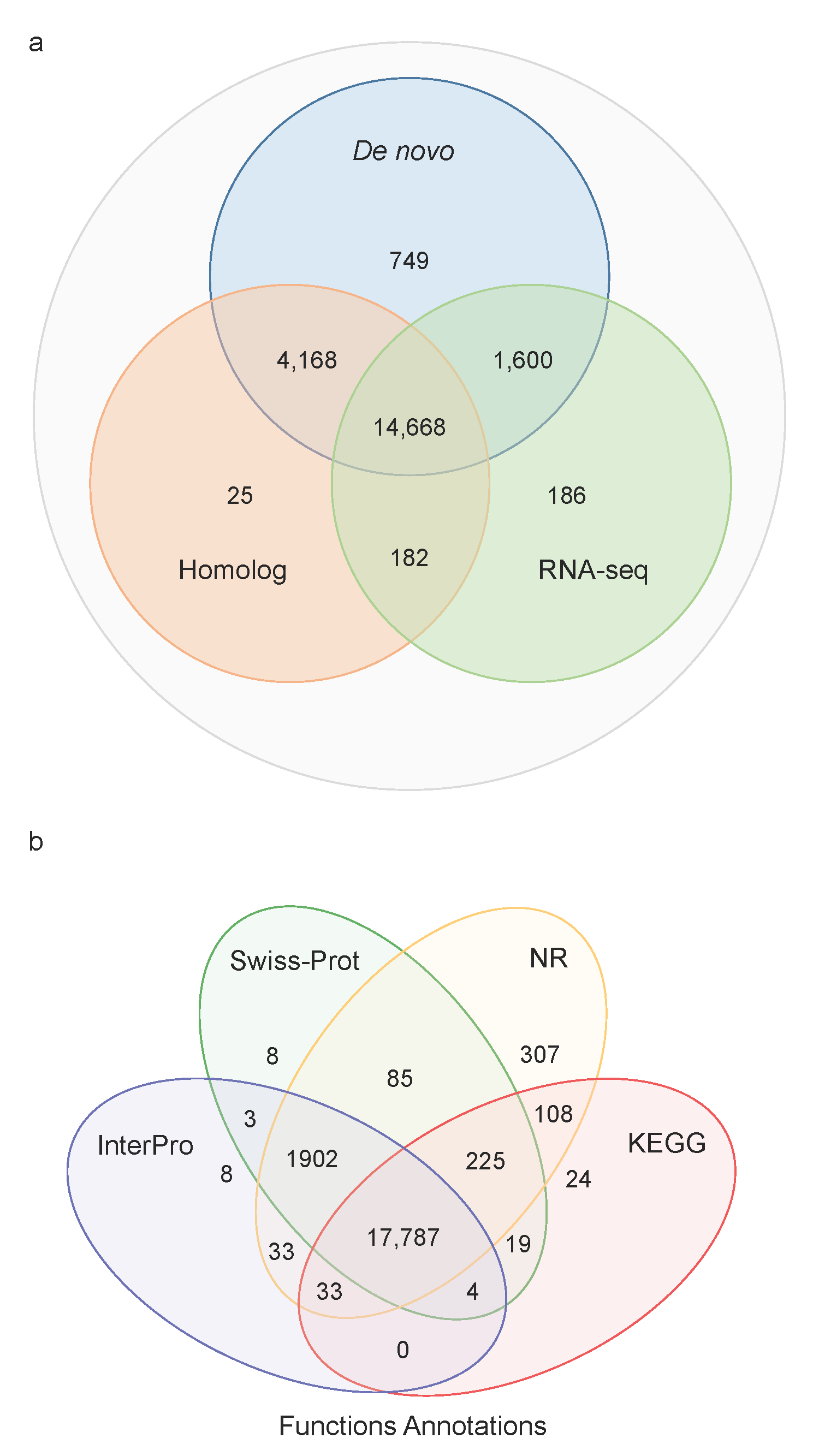
**

**Supplementary Figure S3** Prediction and annotation of genes in masked palm civet genome. (a) Number of genes predicted with *de novo*, homolog, and RNA-seq. All predicted genes were integrated by EVM. (b) Number of genes annotated with databases of Swissprot, NR, GO, KEGG, Pfam and InterPro.
