# Supplementary material for: The assembled and annotated genome of the masked palm civet (Paguma larvata)
Source: Gigascience. 2022 May 18;11:giac041. doi: 10.1093/gigascience/giac041 (PMC9116208; doi:10.1093/gigascience/giac041)
Supplement: giac041_GIGA-D-21-00297_Original_Submission [file giac041_giga-d-21-00297_original_submission.pdf]

GigaScience

The Assembled and Annotated Genome of the Masked Palm Civet (Paguma larvata)

--Manuscript Draft--

**Manuscript Number:**	GIGA-D-21-00297
**Full Title:**	The Assembled and Annotated Genome of the Masked Palm Civet (Paguma larvata)
**Article Type:**	Data Note
**Funding Information:**	Guangdong Province project from the Forestry Administration of Guangdong Province — DR Ping Liu<br>GDAS Special Project of Science and Technology Development (2020GDASYL-20200103090) — DR Ping Liu
**Abstract:**	Background: The masked palm civet (Paguma larvata) acts as an intermediate host of severe acute respiratory syndrome coronavirus (SARS -CoV), which causes SARS, and transfers this virus from bats to humans. Additionally, Paguma larvata has the potential to carry a variety of zoonotic viruses that may threaten human health. However, genome resources for Paguma larvata have not been reported to date.<br>Findings: A chromosome-level genome assembly of Paguma larvata was generated using PacBio sequencing, Illumina sequencing, and Hi-C technology. The genome assembly was 2.44 Gb in size, of which 95.32% could be grouped into 22 pseudochromosomes, with contig N50 and scaffold N50 values of 12.97 Mb, respectively. A total of 21,582 protein-coding genes were predicted, and 95.20% of the predicted genes were functionally annotated. Phylogenetic analysis of 11 animal species confirmed the close genetic relationship between Paguma larvata , Panthera tigris , and Felis Catus . Gene family clustering revealed 87 unique, 312 significantly expanded, and 139 significantly contracted gene families in the Paguma larvata genome. We identified 285 positively selected genes in Paguma larvata , and four known human viral receptor genes were positively selected in Paguma larvata .<br>Conclusions: This high-quality genome assembly provides a valuable genomic resource for elucidating immune mechanisms in this animal. It will also provide a reliable reference for studying the genetic bases of the morphological characteristics, adaptive evolution, and evolutionary history of this species.
**Corresponding Author:**	Jin-Ping Chen<br>Guangdong Academy of Sciences<br>Guangzhou, CHINA
**Corresponding Author Secondary Information:**	
**Corresponding Author's Institution:**	Guangdong Academy of Sciences
**Corresponding Author's Secondary Institution:**	
**First Author:**	Ping Liu
**First Author Secondary Information:**	
**Order of Authors:**	Ping Liu<br>Hai-Ying Jiang<br>Lin-Miao Li<br>Jia-Bin Zhou<br>Wen-Zhong Huang<br>Jin-Ping Chen
**Order of Authors Secondary Information:**	
**Additional Information:**	

Powered by Editorial Manager® and ProduXion Manager® from Aries Systems Corporation

Question	Response
Are you submitting this manuscript to a special series or article collection?	No
**Experimental design and statistics**<br><br>Full details of the experimental design and statistical methods used should be given in the Methods section, as detailed in our Minimum Standards Reporting Checklist. Information essential to interpreting the data presented should be made available in the figure legends.<br><br>Have you included all the information requested in your manuscript?	Yes
**Resources**<br><br>A description of all resources used, including antibodies, cell lines, animals and software tools, with enough information to allow them to be uniquely identified, should be included in the Methods section. Authors are strongly encouraged to cite Research Resource Identifiers (RRIDs) for antibodies, model organisms and tools, where possible.<br><br>Have you included the information requested as detailed in our Minimum Standards Reporting Checklist?	Yes
**Availability of data and materials**<br><br>All datasets and code on which the conclusions of the paper rely must be either included in your submission or deposited in publicly available repositories (where available and ethically appropriate), referencing such data using a unique identifier in the references and in the "Availability of Data and Materials" section of your manuscript.	Yes

Have you have met the above requirement as detailed in our Minimum Standards Reporting Checklist?	

Powered by Editorial Manager® and ProduXion Manager® from Aries Systems Corporation

Manuscript

Click here to access/download;Manuscript;paper-20211007-clear.docx

The Assembled and Annotated Genome of the Masked Palm Civet (*Paguma larvata*)

Ping Liu[1], Hai-Ying Jiang[1], Lin-Miao Li[1], Jia-Bin Zhou[1], Wen-Zhong Huang[1], Jin-Ping Chen[1]

[1]Guangdong Key Laboratory of Animal Conservation and Resource Utilization, Guangdong Public Laboratory of Wild Animal Conservation and Utilization, Institute of Zoology, Guangdong Academy of Sciences, Guangzhou 510260, China; pingliu0330@126.com

Corresponding author: Jin-Ping Chen, Institute of Zoology, Guangdong Academy of Sciences, Guangzhou 510260, China. chenjp@giz.gd.cn; Tel.: +020-8910-0920

Abstract

Background: The masked palm civet (*Paguma larvata*) acts as an intermediate host of severe acute respiratory syndrome coronavirus (SARS-CoV), which causes SARS, and transfers this virus from bats to humans. Additionally, *Paguma larvata* has the potential to carry a variety of zoonotic viruses that may threaten human health. However, genome resources for *Paguma larvata* have not been reported to date. **Findings:** A chromosome-level genome assembly of *Paguma larvata* was generated using PacBio sequencing, Illumina sequencing, and Hi-C technology. The genome assembly was 2.44 Gb in size, of which 95.32% could be grouped into 22 pseudochromosomes, with contig N50 and scaffold N50 values of 12.97 Mb, respectively. A total of 21,582 protein-coding genes were predicted, and 95.20% of the predicted genes were functionally annotated. Phylogenetic analysis of 11 animal species confirmed the close genetic relationship between *Paguma larvata*, *Panthera tigris*, and *Felis Catus*. Gene family clustering revealed 87 unique, 312 significantly expanded, and 139 significantly contracted gene families in the *Paguma larvata* genome. We identified 285 positively selected genes in *Paguma larvata*, and four

known human viral receptor genes were positively selected in *Paguma larvata*. **Conclusions:** This high-quality genome assembly provides a valuable genomic resource for elucidating immune mechanisms in this animal. It will also provide a reliable reference for studying the genetic bases of the morphological characteristics, adaptive evolution, and evolutionary history of this species.

Keywords: Masked palm civet, genome assembly, Hi-C proximity mapping, gene family evolution, phylogeny, positive selection

Introduction

In human history, the spread of viruses has caused huge losses to the human economy and threatened the safety of human life and property. Diseases affecting animals of economic value, caused by viruses such as avian influenza and African swine fever, have caused serious economic losses [1-3]. Viruses such as severe acute respiratory syndrome coronavirus (SARS-CoV), Middle East respiratory syndrome coronavirus (MERS-CoV), and SARS-CoV-2, posed a great threat to the safety of human life and property [4-6]. Usually, zoonotic viruses can be transmitted to humans via wild animals as intermediate hosts. For example, MERS-CoV is transmitted from camels to humans [7].

Masked palm civets, *Paguma larvata* (Mammalia, Carnivora, and Viverridae) are wild mammals that are widely distributed in Asia [8]. They are omnivores, although they mainly consume fruits. In recent decades, *Paguma larvata* used to be domesticated as meat of human beings, increasing the possibility of transferring pathogenic microorganisms to human beings. In 2003, *Paguma larvata* gained attention because of its potential involvement in the outbreak of severe acute respiratory syndrome (SARS), which originated in southern China and spread to over 30 countries [9]. Further research suggests that *Paguma larvata* acts as an intermediate host of SARS-CoV, which transmits this virus from bats to humans [10, 11]. The sequence similarity between the SARS-CoV found in *Paguma larvata* and human SARS-CoV is greater than 99%. However, *Paguma larvata* can live in harmony with SARS-CoV, whereas humans can have severe symptoms following SARS-CoV infection. This shows that

the interaction between *Paguma larvata* and viruses has a certain particularity [12]. Earlier studies on farms raising *Paguma larvata* also found that these animals rarely become ill. The immune system of *Paguma larvata* is thus worthy of in-depth study.

Virus receptors, present on the host cell membrane, are important factors in mediating virus invasion and play an extremely important role in the interaction between host cells and viruses. The virus attachment protein can be regarded as the “key” that unlocks the cell by interacting with the “lock” (receptor) on the surface of the host cell, starting a key downstream step in the virus life cycle. In many cases, viruses use multiple receptors to perform different functions during the viral life cycle [13]. Therefore, the virus must first find the lock and then use a specific virus “key” to unlock the cell. The numerous functions of the virus receptor aid the virus in targeting the correct tissue for infection and to cross the cell barrier, which is necessary to deliver the viral genome to the host cell [14]. The interaction with viral receptors is usually mediated by specific proteins on the surface of the virus [13]. Inherent differences in the shape of the virus surface protein (icosahedron or helix) and the composition of the virus coat affect the overall structure of the virus attachment protein. Some viruses infect only certain species; for example, swine fever virus can only infect pigs [3]. Whereas some viruses can spread between animals and humans, such as SARS-CoV [10, 11] and MERS-CoV [7]; the reason lies in the nature of the interaction between the virus receptor and virus attachment protein. For example, SARS-CoV and SARS-CoV-2 specifically recognize the angiotensin-converting enzyme 2 (ACE2) receptor [15, 16, 17]. Understanding the nature of virus receptors that cause zoonotic diseases in different species will help in tracing natural hosts during the virus epidemic.

As an important virus host, masked palm civets can be used as a system for reviewing virus and host interactions. However, there is currently no genome assembly of *Paguma larvata*, even in Viverridae which has been reported for studying various virus receptors. In this study, we generated a chromosome-level reference genome of *Paguma larvata* by integrating PacBio sequencing, Hi-C sequencing, and Illumina sequencing [18-20]. The high-quality genome assembly will provide a valuable genomic resource for elucidating the immune mechanisms in this animal. It will also provide a reliable reference for studying the genetic bases of the morphological

characteristics, adaption evolution, and evolutionary history of the species.

Data Description

Statement of Ethics

The study design was approved by the Ethics Committee for Animal Experiments at the Institute of Zoology, Guangdong Academy of Sciences (reference number: GIZ20190702, 07/01/2019) and followed the basic principles outlined by this committee.

Isolation of genomic DNA and RNA

A healthy female *Paguma larvata* individual was collected from a farm in Wengyuan County, Guangdong Province, China. Whole blood was isolated from the same masked palm civet and washed with saline nasal spray. These samples were immediately frozen in liquid nitrogen and stored at -80 °C prior to nucleic acid extraction. Genomic DNA was extracted from whole blood using a magnetic universal genomic DNA kit. Different tissues, including heart, intestine, kidney, liver, lung, muscle, and spleen were sampled and flash-frozen in liquid nitrogen for total RNA sequencing. Total RNA was extracted using the TRIzol reagent [21].

Genome sequencing with different technologies

A combined sequencing strategy was used to obtain the masked palm civet genome. Short-insert libraries with a size of 350 bp were sequenced on the Illumina HiSeq 2500 platform using the 150 bp paired-end protocol. For PacBio library construction, we sheared the genomic DNA to 20 kb fragments, which we used to prepare a single-molecule real-time (SMRT) library for sequencing with the PacBio Sequel system [22]. Liver tissue was used to construct the Hi-C sequencing library. Briefly, cross-linked chromatin was digested with Dpn II and ligated in situ after biotinylation. DNA fragments were enriched via the interaction of biotin and blunt-end ligation and then subjected to Illumina NovaSeq PE 150 sequencing [23].

Genome assembly and quality assessment

We applied the FALCON v0.3.0 [24] to correct errors in PacBio long reads, according to PacBio short reads (<5 kb) and then generated consensus sequences. The primary scaffolds were assembled with these subreads, and the scaffolds were further corrected in PILON v1.22 [25] using short sequencing reads. To anchor scaffolds onto chromosomes, we aligned the Hi-C sequencing data to the assembly using BWA v0.7.17 [26] and detected valid contacts. Preassembled scaffolds were clustered, ordered, and directed onto pseudochromosomes using the LACHESIS software [27]. To increase the accuracy of the assembled genome, we artificially corrected the LACHESIS-based assembly, filled gaps, and removed duplicate sequences [28].

Genome size was estimated based on k-mer distribution analysis with the program in GCE [29] using Illumina short reads. We used Benchmarking Universal Single-Copy Orthologs (BUSCO) [30] and Core Eukaryotic Genes Mapping Approach (CEGMA) [31] to evaluate the completeness and accuracy of the genome assembly and BWA v0.7.17 [26] to align the Illumina short reads to the assembly and evaluate the assembled portion. We assembled expressed sequencing tags (ESTs) using Trinity v2.8.4 [32] with RNA reads from seven different tissues (heart, liver, spleen, lung, kidney, muscle, and intestine). To further examine the quality of the genome assembly, we aligned ESTs to the assembled genome using BLAT [33] using parameters of identity $\geq$90% and coverage $\geq$50%. The quality of the genome assembly was evaluated by mapping RNA-Seq reads from the five different tissues to the ESTs using TopHat2 [34].

Repetitive elements annotation

Repetitive sequences were identified at both the DNA and protein levels by integrating homology-based prediction and *de novo* identification. Repeat masking was performed based on repeats in the Repbase TE library from the Repbase server [35]. Using this library, we predicted interspersed repeat elements using RepeatMasker v3.3.0 and ProteinMask (http://www.repeatmasker.org) and screened tandem repeats using TRF v4.07b [36].

Gene prediction and functional annotation

We annotated protein-coding genes in the *Paguma larvata* genome using a combination of *de novo* gene prediction, homology-based prediction, and RNA-seq-based prediction. For *de novo* identification, we predicted the gene models using five *ab initio* gene prediction programs: Augustus [37], GlimmerHMM [38], SNAP [39], Genscan [40], and Geneid [41]. We aligned proteins from five sequenced animals, namely, *Mus musculus* [42], *Canis familiaris* [43], *F. catus* [44], *Panthera tigris* [45], and *Acinonyx jubatus* [46], to the *Paguma larvata* assembly using tBlastN [47] with an e-value cut-off of 1×10^{-5}. Gene models were generated using GeneWise v2.2.0 [48]. We applied two methods of RNA-Seq-based prediction: (i) we mapped RNA-Seq data to the genome using TopHat2 [49] and further generated the gene models in Cufflinks [34] based on exons, and (ii) we aligned ESTs against the assembly to generate the gene models using PASA [50]. We integrated predictions from the three approaches in EVidenceModeler v1.1.1 [51] to generate a non-redundant gene model set. The final gene models were annotated using the non-redundant protein database of NCBI (NR) and Swissprot, Kyoto Encyclopedia of Genes and Genomes (KEGG) [52], Interprot [53], and Pfam databases.

Phylogenetic analysis and estimation of divergence time

We used the Treefam protocol [54] to identify orthologous genes from civet and ten other species, including cat (*F. catus*), chicken (*G. gallus*), dog (*C. familiaris*), giant panda (*Ailuropoda melanoleuca*), human beings (*Homo sapiens*), mouse (*M. musculus*), pig (*Sus scrofa*), polar bear (*Ursus maritimus*), tiger (*Panthera tigris*), and yak (*Bos mutus*) (Supplementary Table S1). We aligned the coding region sequences of single-copy orthologous genes using MUSCLE v3.7 [55]. All alignments were concatenated to a super-alignment matrix. Based on this matrix, we used RAxML v8.0.19 [56] to perform a maximum-likelihood (ML) phylogenetic tree for these species. The MCMCTree program of PAML v4.9 [57] was used to estimate species divergence times using single-copy orthologs based on splits between cat and tiger (12.3–18.1 Ma), polar bear and giant bear (17.8–28.9 Ma), dog and polar bear (42.0–49.0 Ma), dogs and cats (52.0–57.0 Ma), as well as human beings and mice (85.0–94.0 Ma) from TimeTree

(http://www.timetree.org/, last accessed March 17, 2021) as calibration.

Gene family expansion and contraction analysis

To identify gene family evolution as a random birth and death process model, where the gene family either expands or contracts per gene per million years independently along each lineage of the phylogenetic tree, the ML model originally implemented in the software package CAFE v4.2.1 [58] was applied to compare the cluster size differences (gain or loss) between the ancestor and each species. We determined gene family expansions or contractions only when the change in gene copy number was significant ($P < 0.05$).

Positive selection analysis

For positive selection analysis, we first identified single-copy orthologous genes from *Paguma larvata* and four other species: *Panthera tigris, A. melanoleuca, S. scrofa*, and *F. catus*. For these genes, based on phylogenetic topology, we employed the branch-site model incorporated in the PAML package v4.9 [59] to detect positively selected genes (PSGs). *Paguma larvata* was specified as a foreground branch, the other species were used as background branches, and PSGs were identified only when $P < 0.01$.

Sequence analysis of receptors under positive selection and ACE2

Sequences of receptor genes under positive selection and the ACE2 gene in other animal species were downloaded from NCBI. Alignment of coding region sequences and amino acid sequences was conducted using Mafft v7.427 [60]. ML phylogenetic trees were constructed using IQ-TREE v1.6.10 [61]. The sequence identity was estimated using MEGA-X [62].

Results and Discussion

Genome sequencing and assembly

We sequenced the genome of one female masked palm civet (2n = 44) (Figure 1a). Here, 557.75 Gb sequences were generated from high-quality short-read sequences

(272.68 Gb) and PacBio sequences (285.07 Gb), representing over 216× coverage for the genome, which has an estimated size of 2.58 Gb based on the 17-mer depth distribution analysis of the sequenced reads (Supplementary Figure S1) The final assembled sequence was 2.44 Gb, representing 94.6% of the masked palm civet genome. The assembly consisted of 1,785 scaffolds with an N50 of 12.97 Mb (Table 1). We used the Hi-C technology to reorder and anchor 95.32% of the genome onto 22 pseudochromosomes (Figure 1a, Supplementary Figure S2). The GC content and heterozygosity rate of the assembled masked palm civet genome were 42.4% and 0.46%, respectively. The completeness of our assembly was assessed by BUSCO and CEGMA. The BUSCO assessment showed that 95.8% of the 4,104 BUSCOs were assembled, with only 1.3% duplication (Supplementary Table S2), while CEGMA assessment showed that 91.94% and 3.2% of 248 CEGs (Core Eukaryotic Genes) were complete and partially assembled, respectively (Supplementary Table S3). We also used BWA v0.7.17 [26] to align Illumina short reads to the assembly and obtained a mapping rate and genome coverage of 99.35% and 99.73%, respectively. Overall, all evidence suggests that the *Paguma larvata* assembly is a high-quality genome assembly that can be used for further downstream analyses.

Repeat annotation and gene prediction

We analyzed repetitive sequences by combining *de novo* prediction and a homology-based search at both the DNA and protein levels, and found that repetitive sequences occupied 31.80% (775.0 Mb) of the genome of the masked palm civet (Table 1, Figure 1a), which was very similar to the repeat compositions of the cat genome (32.10%), but lower than that of the tiger genome (39.20%) [44, 45]. The most abundant repetitive element type in the masked palm civet genome was long terminal repeat (LTR), representing 542.79 Mb (22.27%) of the genome. We performed an annotation pipeline combining *de novo*, homolog-based search, and RNA-Seq methods to predict gene models from the repeat-masked masked palm civet genome sequence. These analyses predicted 21,582 protein-coding genes, representing 30.61% of the genome assembly (Supplementary Table S4, Supplementary Figure S3a). Among the 21,582 genes, 20,546 (95.20%) were successfully annotated for at least one function term by

searching against functional databases (Gene Ontology (GO), InterProScan, KEGG, NR, Pfam, and SwissProt) (Table 1, Supplementary Figure S3b). The number of protein-coding genes in the *Paguma larvata* genome is slightly higher than that in the cat and tiger genomes (20,285 and 20,226 genes, respectively) [43, 44] (Pontius et al. 2007; Cho et al. 2013). In addition to the protein-coding genes, we also identified 14,829 miRNA genes, 47,575 tRNA genes, and 544 rRNA genes in the masked palm civet genome (Supplementary Table S5).

Gene family comparison

The evolutionary dynamics related to *Paguma larvata* were deduced by comparison with ten animal species. We identified 5,417 one-to-one orthologs among these species (Figure 1b; Supplementary Table S6). Among the 11 species, 87 gene families were unique to *Paguma larvata* (Supplementary Table S6). A total of 312 gene families (including 1,147 genes) were expanded in the masked palm civet. In contrast, 139 gene families (including 276 genes) were significantly contracted in the *Paguma larvata* genome (Figure 1b). The expanded genes were enriched in 51 GO terms, and 43 immunoglobulin-related genes were included (Supplementary Table S7, Supplementary Figure S4). The contracted genes were enriched in 22 GO terms, among which there were 13 immunoglobulin-related genes (Supplementary Table S8, Supplementary Figure S5). There were more immunoglobulin-related genes expanded than contracted, which was in accordance with the high immune response level of masked palm civets.

Phylogenetic Analysis

To confirm the phylogenetic position of *Paguma larvata* and estimate divergence time using whole-genome data, we analyzed the orthologous gene relationships between *Paguma larvata* and ten other animals (see "Phylogenetic analysis and estimation of divergence time" section of "Materials and methods"). Based on a super alignment matrix of coding region sequences of 5,417 single-copy orthologs with *G. gallus* as the outgroup. The phylogenetic analysis supported the genetic relationship between *Paguma larvata*, *Panthera tigris*, and *F. Catus* were closely related, whereas

the time of *Paguma larvata* split with *Panthera tigris* was approximately 37.2 (million years ago (Figure 1c). We obtained the divergence time of masked palm civets for the first time based on whole genome sequences.

Genes under positive selection and ACE2 gene

We used *Paguma larvata* as the foreground branch, and other species of *Panthera tigris*, *A. melanoleuca*, *S. scrofa*, and *F. catus* as background branches to detect genes under selection. Based on 9,414 single-copy genes of these five species, We identified 285 PSGs ($P < 0.01$; Supplementary Table S9). A GO functional classification of significant PSGs in *Paguma larvata* revealed that the majority of genes under positive selection (194 genes) were associated with protein binding (Supplementary Table S10, Supplementary Figure S6). Currently, humans have reported more than 28 types of viral receptors [63]. Four of these known human viral receptor genes, *CD55*, *HSPG2*, *PDGFRA*, and *SCARB2*, were included in genes under positive selection in *Paguma larvata* (Supplementary Table S11), which may be related to the high virus resistance ability of *Paguma larvata.*

The phylogeny of CD55, HSPG2, PDGFRA, SCARB2, and ACE2 receptors from different animal species was conducted based on amino acid sequences downloaded from NCBI. The relationship of these four receptor genes under positive selection in *Paguma larvata* was closest to that of *F. catus* and *Panthera tigris* in the four phylogenetic trees (Figure 2). The amino acid sequence identity of CD55 was the highest between *Paguma larvata* and *Panthera tigris*, whereas HSPG2 and SCARB2 showed the highest identity between *Paguma larvata* and *F. catus*; moreover, amino acid sequence of PDGFRA was the highest among *Paguma larvata*, *F. catus*, and *Panthera tigris* (Table 2). ACE2 acted as a receptor for SARS and SARS-CoV-2, and we conducted phylogenetic analysis and sequence identity based on amino acid sequences of different animal species. ACE2 of *Paguma larvata* was closely related to *F. catus* and *Panthera tigris* (Table 2). Amino acid sequence of CD55, HSPG2, PDGFRA, SCARB2, and ACE2 between *Paguma larvata* and human beings were 35.09%, 89.20%, 94.92%, 83.91%, and 81.79%, respectively. Further research is needed to reveal the differences in the receptor-binding loci of these four receptors in

Paguma larvata and humans.

Conclusion

In this study, we present a high-quality chromosome-level genome assembly of *Paguma larvata* using PacBio single-molecule long-read sequencing, supplemented with Illumina short-read sequencing and Hi-C sequencing. The final genome assembly was 2.44 Gb, 95.32% of which could be grouped into 22 pseudochromosomes. This assembly is highly contiguous with contig N50 and scaffold N50 values of 12.97 Mb. The repeat sequence comprised 31.80% of the genome. A total of 21,582 protein-coding genes were predicted, 95.20% of which could be functionally annotated. Phylogenetic analysis based on 5,417 single-copy orthogroups across 11 animals resulted in a close genetic relationship between *Paguma larvata*, *Panthera tigris,* and *F. Catus*. The divergence time of *Paguma larvata* split from *Panthera tigris* and *F. Catus* was estimated to be 37.2 Ma. Gene family clustering revealed 87 unique, 312 significantly expanded, and 139 significantly contracted gene families in the *Paguma larvata* genome. We identified 285 genes under positive selection in *Paguma larvata*, among which four known human viral receptor genes were found. The high-quality genome assembly will provide a valuable genomic resource for elucidating the immune mechanisms in this animal. It will also provide a reliable reference for studying the genetic bases of the morphological characteristics, adaptive evolution, and evolutionary history of this species.

Data Availability

Raw sequencing data and genome assembly were deposited at the NCBI under the BioProject PRJNA756156 with biosample accession number SAMN20856571. The annotations and predicted peptides are available in FigShare at the link: https://doi.org/10.6084/m9.figshare.15273879.

Competing Interests

The authors declare that they have no competing interests.

Funding

This project was supported by the surveillance of wild animals and epidemic diseases in important ecological areas of Guangdong Province project from the Forestry Administration of Guangdong Province, GDAS Special Project of Science and Technology Development (2020GDASYL-20200103090).

Figure Legends

Figure 1 (a) Synteny of corresponding chromosomes and gene density across the *Paguma larvata* genome; (b) Comparison of copy numbers in gene clusters reside in the genomes of *Paguma larvata* and other ten animals. Single-copy orthologs denote the family can have only one gene for each species, and multicopy orthologs denote the family clustered more than one gene for each species. Other orthologs denote the family can have any number of genes for each species except the single-copy and multi-copy orthologs. Unique paralogs denote species-specific gene families, and unclustered genes denote species-specific genes that cannot cluster with any other genes; (c) Phylogenetic relationships of 11 animal genomes based on the maximum likelihood method. The black numbers indicate estimated divergent times as the number of years (millions) ago, while green and red numbers denote gene families subject to expansion and contraction for each species, respectively.

Figure 2 Phylogenetic relationships of four known human virus receptors under positive selection in *Paguma larvata*.

References

1. Iwami S, Takeuchi Y, Liu X. Avian flu pandemic: Can we prevent it?. J Thero Biol 2009;**257**(1):181-190.
2. Siddiqui AA. The global threat of bird (Avian) flu its treatment methods and public health preventive measures. SOJ Vet Sci 2018; **4**(3):1-4.
3. Dixon L K, Sun H, Roberts H. African swine fever. Antivir Res 2019;**165**:34-41.
4. Rota PA, Oberste MS, Monroe SS, et al. Characterization of a novel coronavirus associated with severe acute respiratory syndrome. Science 2003;**300**(5624):1394-1399.
5. Zumla A, Hui D S, Perlman S. Middle East respiratory syndrome. The Lancet 2015;**386**(9997):995-1007.
6. Wu D, Wu T, Liu Q, et al. The SARS-CoV-2 outbreak: what we know. Int J Infect Dis 2020;**94**:44-48.
7. Sabir JSM, Lam TTY, Ahmed MMM, et al. Co-circulation of three camel coronavirus species and recombination of MERS-CoVs in Saudi Arabia. Science 2016;**351**(6268):81-84.
8. Inoue T, Kaneko Y, Yamazaki K, et al. Genetic population structure of the masked palm civet *Paguma larvata*,(Carnivora: Viverridae) in Japan, revealed from analysis of newly identified compound microsatellites. Conserv Genet 2012;**13**(4):1095-1107.
9. Guan Y, Zheng BJ, He YQ, et al. Isolation and characterization of viruses related to the SARS coronavirus from animals in southern China. Science 2003;**302**(5643):276-278.
10. Li W, Shi Z, Yu M, et al. Bats are natural reservoirs of SARS-like coronaviruses. Science 2005;**310**(5748):676-679.
11. Wang LF, Eaton BT. Bats, civets and the emergence of SARS. Wildlife and emerging zoonotic diseases: the biology, circumstances and consequences of cross-species transmission, 2007:325-344.
12. Wan Y, Shang J, Graham R, et al. Receptor recognition by the novel coronavirus from Wuhan: an analysis based on decade-long structural studies of SARS coronavirus. J Virol 2020;**94**(7):e00127-20.

13. Marsh M, Helenius A. Virus entry: open sesame. Cell 2006;124(4):729-740.

14. Grove J, Marsh M. The cell biology of receptor-mediated virus entry. J Cell Biol 2011;**195**(7):1071-1082.

15. Li W, Moore MJ, Vasilieva N, et al. Angiotensin-converting enzyme 2 is a functional receptor for the SARS coronavirus. Nature 2003;**426**(6965):450-454.

16. Shi R, Shan C, Duan X, et al. A human neutralizing antibody targets the receptor-binding site of SARS-CoV-2. Nature 2020;**584**(7819):120-124.

17. Wu Y, Wang F, Shen C, et al. A noncompeting pair of human neutralizing antibodies block COVID-19 virus binding to its receptor ACE2. Science 2020;**368**(6496):1274-1278.

18. Bickhart DM, Rosen BD, Koren S, et al. Single-molecule sequencing and chromatin conformation capture enable de novo reference assembly of the domestic goat genome. Nat genet, 2017;**49**(4):643-650.

19. Mahajan S, Wei K, Nalley M, et al. De novo assembly of a young Drosophila Y chromosome using Single-Molecule sequencing and Chromatin Conformation capture. PLoS Biol 2018;**16**:1-28.

20. Zhang Z, Chen Y, Zhang J, et al. Improved genome assembly provides new insights into genome evolution in a desert poplar (*Populus euphratica*). Mol Ecol Resour 2020;**20**(3):781-794.

21. Rio DC, Ares M, Hannon GJ, et al. Purification of RNA using TRIzol (TRI reagent). Cold Spring Harbor Protocols, 2010;**2010**(6):pdb. prot5439.

22. Chin CS, Alexander DH, Marks P, et al. Nonhybrid, finished microbial genome assemblies from long-read SMRT sequencing data. Nat methods 2013;**10**(6):563-569.

23. Belton JM, McCord RP, Gibcus JH, et al. Hi‐C: a comprehensive technique to capture the conformation of genomes. Methods 2012;**58**(3):268-276.

24. Chin CS, Peluso P, Sedlazeck FJ, et al. Phased diploid genome assembly with single-molecule real-time sequencing. Nat methods 2016;**13**(12):1050-1054.

25. Walker BJ, Abeel T, Shea T, et al. Pilon: an integrated tool for comprehensive microbial variant detection and genome assembly improvement. PloS one 2014;9(11):e112963.

26. Li H, Durbin R. Fast and accurate short read alignment with Burrows-Wheeler transform. Bioinformatics 2009;**25**:175-1760.
27. Hariharan R, Toyama K. Project Lachesis: parsing and modeling location histories//International Conference on Geographic Information Science. Springer, Berlin, Heidelberg, 2004:106-124.
28. Burton JN, Adey A, Patwardhan RP, et al. Chromosome-scale scaffolding of de novo genome assemblies based on chromatin interactions. Nat Biotechnol 2013;**31**:1119-1125.
29. Liu B, Shi Y, Yuan J, et al. Estimation of genomic characteristics by analyzing k-mer frequency in de novo genome projects. 2013. http://arxiv.org/abs/1308.2012.
30. Manni M, Berkeley MR, Seppey M, et al. BUSCO update: novel and streamlined workflows along with broader and deeper phylogenetic coverage for scoring of eukaryotic, prokaryotic, and viral genomes. Mol Biol Evol 2021;**38**(10): 4647-s4654
31. Parra G, Bradnam K, Korf I. CEGMA: A pipeline to accurately annotate core genes in eukaryotic genomes. Bioinformatics 2007;**23**:1061-1067.
32. Grabherr MG, Haas BJ, Yassour M, et al. Full-length transcriptome assembly from RNA-Seq data without a reference genome. Nat Biotechnol 2011;**29**:644-652.
33. Kent WJ. BLAT—the BLAST-Like Alignment Tool. Genome Res 2002;12:656-664.
34. Trapnell C, Roberts A, Goff L, et al. Differential gene and transcript expression analysis of RNA-seq experiments with TopHat and Cufflinks. Nat Protoc 2012;**7**:562-578.
35. Bao W, Kojima KK, Kohany O. Repbase Update, a database of repetitive elements in eukaryotic genomes. Mob DNA 2015;**6**:4-9.
36. Benson G. Tandem repeats finder: A program to analyze DNA sequences. Nucleic Acids Res 1999;**27**:573-580.
37. Stanke M, Schöffmann O, Morgenstern B, et al. Gene prediction in eukaryotes with a generalized hidden Markov model that uses hints from external sources. BMC Bioinformatics 2006;**7**:1-11.
38. Majoros WH, Pertea M, Salzberg SL. TigrScan and GlimmerHMM: Two open

source ab initio eukaryotic gene-finders. Bioinformatics 2004;**20**:2878-2879.

39. Korf I. Gene finding in novel genomes. BMC Bioinformatics 2004;**5**:1-9.
40. Burge C, Karlin S. Prediction of complete gene structures in human genomic DNA. J Mol Biol 1997;**268**:78-94.
41. Guigó R, Knudsen S, Drake N, et al. Prediction of gene structure. J Mol Biol 1992;**226**:141-157.
42. Gnerre S, MacCallum I, Przybylski D, et al. High-quality draft assemblies of mammalian genomes from massively parallel sequence data. Proc Natl Acad Sci USA 2011;**108**:1513-1518.
43. Kirkness EF, Bafna V, Halpern AL, et al. The dog genome: Survey sequencing and comparative analysis. Science 2003;**301**(5641):1898-1903.
44. Pontius JU, Mullikin JC, Smith DR, et al. Initial sequence and comparative analysis of the cat genome. Genome Res 2007;**17**(11):1675-1689.
45. Cho Y S, Hu L, Hou H, et al. The tiger genome and comparative analysis with lion and snow leopard genomes. Nat Commun 2013;**4**(1):1-7.
46. Dobrynin P, Liu S, Tamazian G, et al. Genomic legacy of the African cheetah, *Acinonyx jubatus*. Genome Biol 2015;**16**(1):1-20.
47. Altschul SF, Gish W, Miller W, et al. Basic local alignment search tool. J Mol Biol 1990;**215**:403-410.
48. Birney E, Clamp M, Durbin R. GeneWise and Genomewise. Genome Res 2004;**14**:988-995.
49. Kim D, Pertea G, Trapnell C, et al. TopHat2: accurate alignment of transcriptomes in the presence of insertions, deletions and gene fusions. Genome biol 2013;**14**(4):1-13.
50. Haas BJ, Delcher AL, Mount SM, et al. Improving the Arabidopsis genome annotation using maximal transcript alignment assemblies. Nucleic Acids Res 2003;**31**:5654-5666.
51. Haas BJ, Salzberg SL, Zhu W, et al. Automated eukaryotic gene structure annotation using EVidenceModeler and the Program to Assemble Spliced Alignments. Genome biol 2008;**9**(1):1-22.
52. Kanehisa M, Sato Y, Kawashima M, et al. KEGG as a reference resource for gene

and protein annotation. Nucleic Acids Res 2016;**44**:457-462.

53. Quevillon E, Silventoinen V, Pillai S, et al. InterProScan: protein domains identifier. Nucleic Acids Res 2005;**33**:116-120.
54. Li H, Coghlan A, Ruan J, et al. TreeFam: a curated database of phylogenetic trees of animal gene families. Nucleic Acids Res 2006;**34**:572-580.
55. Edgar RC. MUSCLE: Multiple sequence alignment with high accuracy and high throughput. Nucleic Acids Res 2004;**32**:1792-1797.
56. Stamatakis A. RAxML version 8: A tool for phylogenetic analysis and post-analysis of large phylogenies. Bioinformatics 2014;**30**:1312-1313.
57. Yang Z. PAML 4: Phylogenetic analysis by maximum likelihood. Mol Biol Evol 2007;**24**:1586-1591.
58. De Bie T, Cristianini N, Demuth JP, et al. CAFE: A computational tool for the study of gene family evolution. Bioinformatics 2006;**22**:1269-1271.
59. Zhang J, Nielsen R, Yang Z. Evaluation of an improved branch-site likelihood method for detecting positive selection at the molecular level. Mol Biol Evol 2005;**22**(12):2472-2479.
60. Katoh K, Standley DM. MAFFT multiple sequence alignment software version 7: improvements in performance and usability. Mol Biol Evol 2013;**30**(4):772-780.
61. Nguyen LT, Schmidt HA, Von Haeseler A, et al. IQ-TREE: a fast and effective stochastic algorithm for estimating maximum-likelihood phylogenies. Mol Biol Evol 2015;**32**(1):268-274.
62. Kumar S, Stecher G, Li M, et al. MEGA X: molecular evolutionary genetics analysis across computing platforms. Mol Biol Evol 2018;**35**(6):1547.
63. Ren L, Wu C, Guo L, et al. Single-cell transcriptional atlas of the Chinese horseshoe bat (*Rhinolophus sinicus*) provides insight into the cellular mechanisms which enable bats to be viral reservoirs. bioRxiv 2020. DOI:10.1101/2020.06.30.175778

Tables

Table 1 *Paguma larvata* Genome Assembly and Annotation Summary.

Genome assembly statistics	
Total length (bp)	2,437,385,791
Contig/Scaffold N50 length (bp)	12,971,789
Contig/Scaffold N90 length (bp)	2,389,527
Longest contig length (bp)	52,858,878
Number of contigs	1,785
Number of contigs larger than N50	56
Number of contigs larger than N90	210
Genome characteristics	
GC content	42.41%
Number of protein-coding genes	21,582
Average transcript length (bp)	34,567.54
Average CDS length (bp)	1,481.00
Average exon length (bp)	170.53
Average exons per gene (bp)	8.68
Repetitive sequences (% of genome)	
DNA (bp)	8,232,288 (0.34%)
LINE (bp)	299,126,376 (12.27%)
SINE (bp)	613,247 (0.03%)
LTR (bp)	542,789,387 (22.27%)
Unknown (bp)	12,280,567 (0.5%)
Total (bp)	775,001,321 (31.8%)
Gene annotations (% of all genes)	
GO	14,148 (65.6%)
InterProScan	19,770 (91.6%)
KEGG	18,200 (84.3%)
Nr	20,480 (94.9%)
PFAM	17,505 (81.1%)
SwissProt	20,033 (92.8%)
Total	21,582

Table 2 Sequence identity of receptors under positive selection between masked palm civet and other animal species.

Species	Sequence identity (%)				
	**CD55**	**HSPG2**	**PDGFRA**	**SCARB2**	**ACE2**
Felis catus	**64.44**	**95.39**	**97.83**	**92.28**	**92.39**
Panthera tigris	**73.00**	**93.84**	**97.83**	90.25	**92.31**
Ursus maritimus	60.04	93.63	95.90	91.27	89.39
Ailuropoda melanoleuca	59.79	93.10	95.12	90.25	89.23
Canis lupus	60.91	93.02	96.48	89.91	88.27
Sus scrofa	50.78	91.65	94.73	85.00	80.74
Manis javanica	53.29	90.27	93.32	81.71	84.29
Desmodus rotundus	45.04	89.48	91.83	80.21	76.87
Homo sapiens	35.09	89.20	94.92	83.91	81.79
Bos mutus	41.39	89.03	92.84	85.36	79.96
Mus musculus	25.59	84.46	89.47	77.92	79.84
Gallus gallus	--	55.03	77.52	47.46	60.88

Click here to access/download;Table;Supplementary Tables-0922.xlsx

Supplementary table S1 List of animal genome sequences used in the comparative ge

Species	Version
Gallus gallus	NCBI.GCF_000002315.5
Mus musculus	NCBI.GCF_000001635.26; ensembl.v86
Homo sapiens	NCBI.GCF_000001405.38
Sus scrofa	ensembl.v86
Canis lupus	ncbi.vertebrate_mammalian.GCF_000002285.3
Ailuropoda melanoleuca	ensembl.v86
Panthera tigris	ncbi.vertebrate_mammalian.GCF_000464555.1
Felis catus	ensembl.v86
Bos mutus	ncbi.vertebrate_mammalian.GCF_000298355.1
Ursus maritimus	ncbi.vertebrate_mammalian.GCF_000687225.1

enomic analysis except masked palm civet.

Supplementary Table S2 Evaluation of the genome assembly of P. larvata using Benchmarking Universa

BUSCO assessment results[a]	Percentage (%)
Complete BUSCOs[b]	95.6%
Complete and single-copy BUSCOs	94.6%
Complete and duplicated BUSCOs[c]	1.0%
Fragmented BUSCOs[d]	2.3%
Missing BUSCOs	2.1%

[a] The number of total BUSCO groups is 4104.

b'Complete BUSCOs': The length of aligned sequences of orthologs from *P. larvata* is over 95% of BUSCO gı

[c]'Complete Duplicated BUSCOs': Multiple copies of complete BUSCOs.

[d]Fragmented BUSCOs: The BUSCOs meet the 'expected-score' cut-off, but not complete.

l Single-Copy Orthologs (BUSCO).

roup's mean length.

Supplementary Table S3 Evaluation of the genome assembly of P. larvata using core Eukaryotic genes

Category	Prot[a]	Completeness (%)[b]
Complete[c]	230	92.7
Complete + Partial[d]	238	96.0

[a]Prots: The number of core genes assembled in *P. larvata* .

[b]Completeness: The number of core genes in *P. larvata* / 248 (the total number of the set of core genes), whil

[c]'Complete' refers to those predicted proteins has an alignment length that is over 70% of the protein length.

[d]'Partial' refers to the proteins that exceeds a pre-computed minimum alignment score, but not complete.

; mapping approach (CEGMA).

le the set of 248 core genes were conserved in 6 model eukaryotes.

Supplementary Table S4 Summary statistics of predicted protein-coding genes in P. larvata.

Approach	Software/species	No.	Average gene length (bp)	Average CDS length per gene(bp)
De novo identification	Augustus	25,408	22,359	1,194
	GlimmerHMM	565,306	3,692	461
	SNAP	173,674	17,764	490
	Geneid	47,478	28,722	965
	Genscan	59,717	29,244	1,275
Homology-based prediction (tBlasntN + Genewise)	*Acinonyx. jubatus*	18,282	25,424	1,342
	Canis. familiaris	19,439	26,629	1,459
	Felis. Catus	19,755	24,602	1,407
	Panthera. Tigris	19,013	26,770	1,429
	Mus. musculus	18,960	27,238	1,489
RNA-Seq-based prediction	PASA	190,071	20,398	1,144
	Transcripts	39,933	45,972	3,865
EVM		32,654	21,360	1,103
**Final set**		**21,582**	**34,568**	**1,481**

Average exons per gene	Average exon length (bp)	Average intron length (bp)
6.4	185.7	3898.1
2.4	190.1	2263.0
3.5	139.7	6881.3
5.3	182.3	6465.7
7.8	163.1	4104.9
7.8	171.2	3519.6
8.3	176.5	3463.1
8.1	173.9	3271.4
8.3	171.9	3467.2
8.3	179.2	3521.7
6.9	166.3	3274.2
10.1	382.6	4626.5
6.2	177.4	3882.8
**8.7**	**170.5**	**4305.6**

Supplementary Table S5 Non-coding RNAs predicted in the genome of P.

Type		Number	Average length (bp)	Total length (bp)	% of genome
miRNA		14829	102.08	1513731	0.062104
tRNA		47575	83.94	3993560	0.16
rRNA	**rRNA**	**544**	**83.45**	**45396**	**0.001862**
	18S	17	188.53	3205	0.000131
	28S	61	128.21	7821	0.000321
	5.8S	1	78	78	0.000003
	5S	465	73.75	34292	0.001407
snRNA	snRNA	2221	119.94	266391	0.010929
	CD-box	324	95.16	30832	0.001265
	HACA-box	326	139.75	45557	0.001869
	splicing	1498	118.44	177426	0.007279
	scaRNA	67	182.13	12203	0.000501
	Unknown	6	62.17	373	0.000015

Supplementary Table S6 Gene families clustered by OrthoFinder in 11 species. Genes used for Orthol

Species	Gene number	Single copy genes	Multiple copy genes	Unique genes
Gallus gallus	17,468	8,202	367	1453
Mus musculus	13,692	7,430	2555	351
Homo sapiens	19,717	6,849	3912	148
Sus scrofa	20,653	6,928	3809	57
Canis lupus	20,949	6,719	4500	61
Ailuropoda melanoleuca	22,440	6,567	5207	75
Paguma larvata	21,582	6,556	4416	196
Panthera tigris	18,496	6,987	3505	5
Felis catus	19,735	6,924	3711	20
Bos mutus	20,506	6,755	4421	29
Ursus maritimus	19,379	6,983	3638	11

Unique genes: Number of species-specific paralogous genes.

Unclustered genes: Number of species-specific genes that are not assigned to any families.

Finder were proteins without splice variants.

Other genes	Unclustered genes
5770	1676
2967	389
8494	314
9182	677
8902	767
9739	852
7441	2973
7635	364
8555	525
8732	569
8187	560

Supplementary Table S7 Genes in significant expanded gene families in masked palm civet.

Gene ID	NR_descripti	Swissprot_description	KEGG_an
evm.model.HIC_ASM_14.534	hypothetical	40S ribosomal protein S11 OS=Canis lupus fan	K02949 sm
evm.model.HIC_ASM_3.354	PREDICTEI	40S ribosomal protein S11 OS=Rattus norvegic	K02949 sm
evm.model.HIC_ASM_18.351	40S ribosom	40S ribosomal protein S11 OS=Rattus norvegic	K02949 sm
evm.model.HIC_ASM_3.1137	60S ribosom	40S ribosomal protein S11 OS=Rattus norvegic	K02949 sm
evm.model.HIC_ASM_6.222	PREDICTEI	40S ribosomal protein S11 OS=Rattus norvegic	K02949 sm
evm.model.HIC_ASM_3.421.2	casein kinase	Casein kinase I isoform alpha OS=Mus muscul	K08957 ca
evm.model.HIC_ASM_8.877	PREDICTEI	Casein kinase I isoform alpha OS=Ovis aries O	K08957 ca
evm.model.CTG_1542.1	wiskott-Aldri	--	--
evm.model.HIC_ASM_8.430	WW domain	--	--
evm.model.HIC_ASM_5.825	DNA primas	Nascent polypeptide-associated complex subun	K15730 cy
evm.model.HIC_ASM_0.936	PREDICTEI	Nascent polypeptide-associated complex subun	K03626 na
evm.model.HIC_ASM_8.359	Opioid-bindi	Opioid-binding protein/cell adhesion molecule	K06773 op
evm.model.HIC_ASM_8.356	PREDICTEI	Neurotrimin OS=Bos taurus OX=9913 GN=NT	K06774 ne
evm.model.HIC_ASM_12.351	PREDICTEI	Zinc finger protein 396 OS=Homo sapiens OX	K09230 SC
evm.model.HIC_ASM_2.1996	zinc finger a	Zinc finger and SCAN domain-containing prote	K09229 KI
evm.model.HIC_ASM_1.4	zinc finger p	Zinc finger protein 197 OS=Homo sapiens OX	K09229 KI
evm.model.HIC_ASM_17.85	--	--	--
evm.model.HIC_ASM_17.80	--	--	--
evm.model.HIC_ASM_6.302	PREDICTEI	Olfactory receptor 2W3 OS=Homo sapiens OX	K04257 olf
evm.model.HIC_ASM_6.269	PREDICTEI	Putative olfactory receptor 2B3 OS=Homo sapi	K04257 olf
evm.model.HIC_ASM_8.190	LOW QUAL	Trehalase OS=Oryctolagus cuniculus OX=998	K01194 alp
evm.model.HIC_ASM_8.192	trehalase isof	Trehalase OS=Homo sapiens OX=9606 GN=T	K01194 alp
evm.model.HIC_ASM_18.43	PREDICTEI	Voltage-dependent calcium channel gamma-8 s	K04873 vo
evm.model.HIC_ASM_20.105	PREDICTEI	Voltage-dependent calcium channel gamma-4 s	K04869 vo
evm.model.HIC_ASM_5.520	PREDICTEI	Synaptonemal complex protein 3 OS=Homo sa	K19528 sy
evm.model.HIC_ASM_9.837	PREDICTEI	Synaptonemal complex protein 3 OS=Bos taur	K19528 sy
evm.model.HIC_ASM_6.1104	60S ribosom	60S ribosomal protein L31 OS=Paralichthys ol	K02910 lar
evm.model.HIC_ASM_3.824	60S ribosom	60S ribosomal protein L31 OS=Paralichthys ol	K02910 lar
evm.model.HIC_ASM_10.144	mCG49764	60S ribosomal protein L31 OS=Rattus norvegic	K02910 lar
evm.model.HIC_ASM_4.397	60S ribosom	60S ribosomal protein L31 OS=Paralichthys ol	K02910 lar
evm.model.HIC_ASM_13.785	60S ribosom	60S ribosomal protein L31 OS=Paralichthys ol	K02910 lar
evm.model.HIC_ASM_15.183	hypothetical	60S ribosomal protein L31 OS=Paralichthys ol	K02910 lar
evm.model.HIC_ASM_12.542	60S ribosom	60S ribosomal protein L31 OS=Paralichthys ol	K02910 lar
evm.model.HIC_ASM_7.1193	hypothetical	60S ribosomal protein L31 OS=Paralichthys ol	K02910 lar
evm.model.HIC_ASM_18.746	60s ribosom	60S ribosomal protein L31 OS=Rattus norvegic	K02910 lar
evm.model.HIC_ASM_7.984	60S ribosom	60S ribosomal protein L31 OS=Rattus norvegic	K02910 lar
evm.model.HIC_ASM_21.125	60S ribosom	60S ribosomal protein L31 OS=Paralichthys ol	K02910 lar
evm.model.HIC_ASM_13.965	60S ribosom	60S ribosomal protein L31 OS=Rattus norvegic	K02910 lar
evm.model.HIC_ASM_2.1780	hypothetical	60S ribosomal protein L31 OS=Paralichthys ol	K02910 lar
evm.model.HIC_ASM_3.1421	60S ribosom	60S ribosomal protein L31 OS=Paralichthys ol	K02910 lar
evm.model.HIC_ASM_15.526	60S ribosom	60S ribosomal protein L31 OS=Paralichthys ol	K02910 lar
evm.model.HIC_ASM_5.1065	60S ribosom	60S ribosomal protein L31 OS=Paralichthys ol	K02910 lar
evm.model.HIC_ASM_1.323	mCG49764	60S ribosomal protein L31 OS=Ictalurus punct	K02910 lar
evm.model.HIC_ASM_14.851	unnamed pro	60S ribosomal protein L31 OS=Paralichthys ol	K02910 lar
evm.model.HIC_ASM_2.714	60S ribosom	60S ribosomal protein L31 OS=Rattus norvegic	K02910 lar
evm.model.HIC_ASM_5.823	60S ribosom	60S ribosomal protein L31 OS=Rattus norvegic	K02910 lar

evm.model.HIC_ASM_7.940	60S ribosoma	60S ribosomal protein L31 OS=Rattus norvegic	K02910 lar
evm.model.CTG_117.2	60S ribosoma	60S ribosomal protein L31 OS=Paralichthys ol	K02910 lar
evm.model.HIC_ASM_4.922	Hypothetical	60S ribosomal protein L31 OS=Paralichthys ol	K02910 lar
evm.model.HIC_ASM_14.107	40S ribosoma	40S ribosomal protein S17 OS=Rattus norvegic	K02962 sm
evm.model.HIC_ASM_4.1912	40S ribosoma	40S ribosomal protein S17 OS=Sus scrofa OX=	K02962 sm
evm.model.HIC_ASM_5.682	PREDICTEI	40S ribosomal protein S17 OS=Sus scrofa OX=	K02962 sm
evm.model.HIC_ASM_12.212	60S ribosoma	60S ribosomal protein L29 OS=Macaca fascicu	K02905 lar
evm.model.HIC_ASM_13.782	hypothetical	60S ribosomal protein L29 OS=Macaca fascicu	K02905 lar
evm.model.HIC_ASM_19.964	60S ribosoma	60S ribosomal protein L29 OS=Macaca fascicu	K02905 lar
evm.model.HIC_ASM_6.59	PREDICTEI	60S ribosomal protein L29 OS=Macaca fascicu	K02905 lar
evm.model.HIC_ASM_7.209	60S ribosoma	60S ribosomal protein L29 OS=Macaca fascicu	K02905 lar
evm.model.HIC_ASM_12.554	60S ribosoma	60S ribosomal protein L29 OS=Macaca fascicu	K02905 lar
evm.model.HIC_ASM_1.424	putative pept	Peptidyl-prolyl cis-trans isomerase A OS=Sus s	K03767 pe
evm.model.HIC_ASM_18.917	peptidyl-prol	Peptidyl-prolyl cis-trans isomerase A OS=Sus s	K03767 pe
evm.model.HIC_ASM_2.551	PREDICTEI	Peptidyl-prolyl cis-trans isomerase A OS=Sus s	K03767 pe
evm.model.HIC_ASM_0.1485	PREDICTEI	Peptidyl-prolyl cis-trans isomerase A OS=Sus s	K03767 pe
evm.model.HIC_ASM_7.737	peptidyl-prol	Peptidyl-prolyl cis-trans isomerase A OS=Sus s	K03767 pe
evm.model.HIC_ASM_16.589	peptidyl-prol	Peptidyl-prolyl cis-trans isomerase A OS=Sus s	K03767 pe
evm.model.HIC_ASM_1.1126	Peptidyl-prol	Peptidyl-prolyl cis-trans isomerase A OS=Sus s	K03767 pe
evm.model.HIC_ASM_20.206	peptidyl-prol	Peptidyl-prolyl cis-trans isomerase A OS=Sus s	K12738 pe
evm.model.HIC_ASM_3.366	peptidyl-prol	Peptidyl-prolyl cis-trans isomerase A OS=Sus s	K03767 pe
evm.model.HIC_ASM_16.285	peptidyl-prol	Peptidyl-prolyl cis-trans isomerase A OS=Sus s	K03767 pe
evm.model.HIC_ASM_9.253	Peptidyl-prol	Peptidyl-prolyl cis-trans isomerase A OS=Oryc	K03767 pe
evm.model.HIC_ASM_1.143	hypothetical	NK-tumor recognition protein OS=Homo sapie	K12740 NI
evm.model.CTG_949.1	protein phosp	Serine/threonine-protein phosphatase PP1-beta	K06269 ser
evm.model.HIC_ASM_13.345	protein phosp	Serine/threonine-protein phosphatase PP1-beta	K06269 ser
evm.model.HIC_ASM_3.125	60S ribosoma	60S ribosomal protein L10a OS=Rattus norveg	K02865 lar
evm.model.HIC_ASM_2.867	PREDICTEI	60S ribosomal protein L10a OS=Mus musculus	K02865 lar
evm.model.HIC_ASM_6.551	60S ribosoma	60S ribosomal protein L10a OS=Rattus norveg	K02865 lar
evm.model.HIC_ASM_18.188	60S ribosoma	60S ribosomal protein L10a OS=Macaca fascic	K02865 lar
evm.model.HIC_ASM_18.967	60S ribosoma	60S ribosomal protein L10a OS=Mus musculus	K02865 lar
evm.model.HIC_ASM_2.870	Prefoldin sub	Prefoldin subunit 5 OS=Mus musculus OX=10(	K04797 pro
evm.model.HIC_ASM_0.1013	PREDICTEI	Prefoldin subunit 5 OS=Homo sapiens OX=96(	K04797 pro
evm.model.HIC_ASM_12.164	PREDICTEI	Prefoldin subunit 5 OS=Homo sapiens OX=96(	K04797 pro
evm.model.HIC_ASM_5.948	PREDICTEI	Prefoldin subunit 5 OS=Homo sapiens OX=96(	K04797 pro
evm.model.HIC_ASM_18.115	zinc finger ho	Zinc finger homeobox protein 3 OS=Homo sap	K09378 AT
evm.model.HIC_ASM_15.202	PREDICTEI	Zinc finger homeobox protein 4 OS=Homo sap	K09380 zin
evm.model.HIC_ASM_14.138	ADP/ATP tra	ADP/ATP translocase 2 OS=Mus musculus OX	K05863 so
evm.model.HIC_ASM_3.13	ADP/ATP tra	ADP/ATP translocase 2 OS=Homo sapiens OX	K05863 so
evm.model.HIC_ASM_7.916	ADP/ATP tra	ADP/ATP translocase 2 OS=Bos taurus OX=9(	K05863 so
evm.model.HIC_ASM_7.530	spindlin-2B i	Spindlin-2 OS=Pongo abelii OX=9601 GN=SP	--
evm.model.HIC_ASM_7.532	spindlin-2B i	Spindlin-2 OS=Pongo abelii OX=9601 GN=SP	--
evm.model.HIC_ASM_7.529	PREDICTEI	Spindlin-2 OS=Bos taurus OX=9913 GN=SPIN	--
evm.model.HIC_ASM_18.49	hypothetical	T-cell-interacting, activating receptor on myelo	K14377 os
evm.model.HIC_ASM_18.48	PREDICTEI	T-cell-interacting, activating receptor on myelo	K14377 os
evm.model.HIC_ASM_18.923	ATP-binding	ATP-binding cassette sub-family C member 11	K05671 AT
evm.model.HIC_ASM_3.739	phosphatidyl	Phosphatidylcholine translocator ABCB4 OS=I	K05659 AT
evm.model.HIC_ASM_5.144	PREDICTEI	Ataxin-10 OS=Bos taurus OX=9913 GN=ATX	K19323 ata

evm.model.HIC_ASM_3.908	ataxin-10 [O	Ataxin-10 OS=Bos taurus OX=9913 GN=ATX	K19323 ata
evm.model.HIC_ASM_19.106	60S ribosoma	60S ribosomal protein L32 OS=Rattus norvegic	K02912 lar
evm.model.HIC_ASM_5.766	hypothetical	60S ribosomal protein L32 OS=Rattus norvegic	K02912 lar
evm.model.HIC_ASM_20.16	60S ribosoma	60S ribosomal protein L32 OS=Rattus norvegic	K02912 lar
evm.model.HIC_ASM_0.637	Chondroitin s	60S ribosomal protein L32 OS=Bos taurus OX	K02912 lar
evm.model.HIC_ASM_5.446	60S ribosoma	60S ribosomal protein L32 OS=Bos taurus OX	K02912 lar
evm.model.HIC_ASM_4.1551	RPL32 [Cric	60S ribosomal protein L32 OS=Ictalurus puncta	K02912 lar
evm.model.HIC_ASM_13.596	hypothetical	60S ribosomal protein L32 OS=Rattus norvegic	K02912 lar
evm.model.HIC_ASM_9.477	PREDICTED	60S ribosomal protein L32 OS=Rattus norvegic	K02912 lar
evm.model.HIC_ASM_5.1851	RPL32 [Cric	60S ribosomal protein L32 OS=Rattus norvegic	K02912 lar
evm.model.HIC_ASM_16.235	PREDICTED	60S ribosomal protein L32 OS=Rattus norvegic	K02912 lar
evm.model.HIC_ASM_7.181	60S ribosoma	60S ribosomal protein L32 OS=Rattus norvegic	K02912 lar
evm.model.CTG_1113.2	60s ribosoma	60S ribosomal protein L32 OS=Ictalurus puncta	K02912 lar
evm.model.HIC_ASM_2.460	elongation fa	Elongation factor 1-beta OS=Homo sapiens OX	K03232 elo
evm.model.HIC_ASM_10.860	elongation fa	Elongation factor 1-beta OS=Xenopus laevis O	K03232 elo
evm.model.HIC_ASM_15.732	elongation fa	Elongation factor 1-delta OS=Macaca fascicula	K15410 elo
evm.model.CTG_1696.2	integrator co	--	K13180 AT
evm.model.CTG_432.1	PREDICTED	--	--
evm.model.CTG_1696.1	PREDICTED	--	--
evm.model.HIC_ASM_16.288	PREDICTED	Protocadherin-17 OS=Homo sapiens OX=9606	K16499 pro
evm.model.HIC_ASM_7.743	hypothetical	Protocadherin-19 OS=Homo sapiens OX=9606	K16499 pro
evm.model.HIC_ASM_3.481	PREDICTED	Heat shock factor-binding protein 1 OS=Homo	K19765 he
evm.model.HIC_ASM_7.379	PREDICTED	Heat shock factor-binding protein 1 OS=Pongo	K19765 he
evm.model.HIC_ASM_2.974	PREDICTED	Heat shock factor-binding protein 1 OS=Pongo	K19765 he
evm.model.HIC_ASM_14.258	PREDICTED	Receptor-type tyrosine-protein phosphatase eps	K18033 rec
evm.model.HIC_ASM_6.1273	PREDICTED	Receptor-type tyrosine-protein phosphatase kap	K06776 rec
evm.model.HIC_ASM_12.494	PREDICTED	Receptor-type tyrosine-protein phosphatase mu	K05693 rec
evm.model.HIC_ASM_21.504	PREDICTED	Receptor-type tyrosine-protein phosphatase T (	K13297 rec
evm.model.CTG_401.2	tensin-3 [Fel	Tensin-2 OS=Homo sapiens OX=9606 GN=TN	K18080 ter
evm.model.CTG_495.1	PREDICTED	--	K18080 ter
evm.model.CTG_96.5	PREDICTED	Tensin-3 OS=Mus musculus OX=10090 GN=T	K18080 ter
evm.model.CTG_1043.4	PREDICTED	Bromodomain-containing protein 9 OS=Homo	K22184 bro
evm.model.HIC_ASM_10.173	PREDICTED	Bromodomain-containing protein 9 OS=Homo	K22184 bro
evm.model.CTG_830.5	bromodomain	Bromodomain-containing protein 9 OS=Homo	K22184 bro
evm.model.CTG_1655.2	PREDICTED	Bromodomain-containing protein 9 OS=Homo	K22184 bro
evm.model.HIC_ASM_11.476	PREDICTED	Heterogeneous nuclear ribonucleoproteins A2/I	K13158 he
evm.model.HIC_ASM_4.1266	PREDICTED	Heterogeneous nuclear ribonucleoprotein A3 ho	K12741 he
evm.model.CTG_96.1	hypothetical	Putative ubiquitin thioesterase L96 OS=Tipula	K19464 plo
evm.model.CTG_605.4	hypothetical	Golgin subfamily A member 6-like protein 1 O	K19464 plo
evm.model.CTG_319.4	hypothetical	Putative ubiquitin thioesterase L96 OS=Tipula	K19464 plo
evm.model.CTG_120.2	hypothetical	Putative ubiquitin thioesterase L96 OS=Tipula	K19464 plo
evm.model.CTG_786.1	hypothetical	--	K19464 plo
evm.model.HIC_ASM_18.143	hypothetical	Probable G-protein coupled receptor 152 OS=M	K23455 co
evm.model.CTG_320.1	hypothetical	Golgin subfamily A member 6-like protein 1 O	K19464 plo
evm.model.HIC_ASM_12.395	PREDICTED	40S ribosomal protein S26 OS=Rattus norvegic	K02976 sm
evm.model.HIC_ASM_5.856.1	PREDICTED	40S ribosomal protein S26 OS=Rattus norvegic	K02976 sm
evm.model.HIC_ASM_8.129	hypothetical	40S ribosomal protein S26 OS=Octopus vulgar	K02976 sm
evm.model.HIC_ASM_1.1153	PREDICTED	40S ribosomal protein S26 OS=Rattus norvegic	K02976 sm

evm.model.HIC_ASM_7.47	PREDICTEI	40S ribosomal protein S26 OS=Rattus norvegic	K02976	sm
evm.model.HIC_ASM_4.1046	PREDICTEI	T cell receptor alpha variable 2 OS=Homo sapi	K10784	T
evm.model.HIC_ASM_4.1028	Hypothetical	T cell receptor alpha variable 26-2 OS=Homo s	K10784	T
evm.model.CTG_1638.1	probable palı	Probable palmitoyltransferase ZDHHC11 OS=l	K20027	pa
evm.model.CTG_789.3	probable palı	Probable palmitoyltransferase ZDHHC11 OS=l	K20027	pa
evm.model.HIC_ASM_18.113	probable palı	Probable palmitoyltransferase ZDHHC1 OS=M	K20027	pa
evm.model.HIC_ASM_10.173	probable palı	Probable palmitoyltransferase ZDHHC11 OS=l	K20027	pa
evm.model.HIC_ASM_4.1822	NT-3 growth	NT-3 growth factor receptor OS=Pan troglodyt	K05101	ne
evm.model.HIC_ASM_9.906	BDNF/NT-3	BDNF/NT-3 growth factors receptor OS=Hom	K04360	ne
evm.model.HIC_ASM_7.537	unnamed pro	Y-box factor homolog OS=Aplysia californica	K06099	co
evm.model.HIC_ASM_7.538	unnamed pro	Y-box factor homolog OS=Aplysia californica	K06099	co
evm.model.HIC_ASM_14.72	zinc finger pı	Zinc finger protein 248 OS=Homo sapiens OX:	K09228	KI
evm.model.HIC_ASM_18.747	zinc finger pı	Zinc finger protein 567 OS=Homo sapiens OX:	K09228	KI
evm.model.HIC_ASM_20.522	chromodoma	Chromodomain-helicase-DNA-binding protein	K11642	ch
evm.model.HIC_ASM_20.523	chromodoma	Chromodomain-helicase-DNA-binding protein	K11642	ch
evm.model.HIC_ASM_14.92	ryanodine re	Ryanodine receptor 2 OS=Homo sapiens OX=9	K04962	rya
evm.model.CTG_251.1	ryanodine re	Ryanodine receptor 2 OS=Homo sapiens OX=9	K04962	rya
evm.model.HIC_ASM_9.756	60S ribosom	60S ribosomal protein L12 OS=Mus musculus	K02870	lar
evm.model.HIC_ASM_0.1329				
evm.model.HIC_ASM_8.1481	neurexin-2-b	Neurexin-2 OS=Rattus norvegicus OX=10116	K07377	ne
evm.model.HIC_ASM_4.374	PREDICTEI	Neurexin-3 OS=Mus musculus OX=10090 GN	K07377	ne
evm.model.HIC_ASM_13.837	neurexin-1-li	Neurexin-1 OS=Homo sapiens OX=9606 GN=	K07377	ne
evm.model.HIC_ASM_4.868	--	--	--	
evm.model.HIC_ASM_4.867	--	--	--	
evm.model.HIC_ASM_0.1175	--	--	--	
evm.model.HIC_ASM_10.285	--	--	--	
evm.model.CTG_228.5	--	--	--	
evm.model.HIC_ASM_6.1158	sodium-depe	Sodium-dependent glucose transporter 1 OS=H	K08175	M
evm.model.HIC_ASM_17.37	PREDICTEI	Major facilitator superfamily domain-containin	K08175	M
evm.model.HIC_ASM_0.1639	60S ribosom	60S ribosomal protein L35 OS=Sus scrofa OX=	K02918	lar
evm.model.HIC_ASM_4.890	PREDICTEI	60S ribosomal protein L35 OS=Bos taurus OX:	K02918	lar
evm.model.HIC_ASM_9.338	60S ribosom	60S ribosomal protein L35 OS=Bos taurus OX:	K02918	lar
evm.model.HIC_ASM_9.1069	Contactin-as	Contactin-associated protein-like 4 OS=Mus m	K07380	co
evm.model.HIC_ASM_2.1012	contactin-ass	Contactin-associated protein-like 5 OS=Canis l	K07380	co
evm.model.HIC_ASM_11.932	contactin-ass	Contactin-associated protein-like 2 OS=Mus m	K07380	co
evm.model.HIC_ASM_3.1396	PREDICTEI	Succinate dehydrogenase [ubiquinone] cytochr	K00237	su
evm.model.HIC_ASM_8.68	PREDICTEI	Succinate dehydrogenase [ubiquinone] cytochr	K00237	su
evm.model.HIC_ASM_1.1046	ephrin type-A	Ephrin type-A receptor 6 OS=Homo sapiens O	K05107	Ep
evm.model.HIC_ASM_1.1047	ephrin type-A	Ephrin type-A receptor 6 OS=Homo sapiens O	K05107	Ep
evm.model.HIC_ASM_2.231	nuclear autoa	Nuclear autoantigen Sp-100 OS=Homo sapiens	K15413	nu
evm.model.HIC_ASM_2.237	sp110 nuclea	Interferon-induced protein 75 OS=Mus caroli (	K15413	nu
evm.model.HIC_ASM_16.256	PREDICTEI	60S ribosomal protein L36a OS=Rattus norveg	K02929	lar
evm.model.HIC_ASM_2.856	60S ribosom	60S ribosomal protein L36a OS=Rattus norveg	K02929	lar
evm.model.HIC_ASM_0.704	60S ribosom	60S ribosomal protein L36a OS=Rattus norveg	K02929	lar
evm.model.HIC_ASM_3.254	60S ribosom	60S ribosomal protein L36a OS=Takifugu rubr	K02929	lar
evm.model.HIC_ASM_4.3	60S ribosom	60S ribosomal protein L36a OS=Rattus norveg	K02929	lar
evm.model.HIC_ASM_1.1075	Hypothetical	60S ribosomal protein L36a-like OS=Pongo ab	K02929	lar
evm.model.HIC_ASM_7.764.1	ribosomal pr	60S ribosomal protein L36a OS=Rattus norveg	K02929	lar

evm.model.HIC_ASM_2.1292	PREDICTEI	60S ribosomal protein L36a OS=Rattus norveg	K02929	la
evm.model.HIC_ASM_3.1400	A disintegrin	A disintegrin and metalloproteinase with throm	K08618	a c
evm.model.HIC_ASM_0.1172	PREDICTEI	A disintegrin and metalloproteinase with throm	K08619	a c
evm.model.CTG_248.1	LOW QUAL	A disintegrin and metalloproteinase with throm	K08622	a c
evm.model.HIC_ASM_8.1783	ribonuclease	Ribonuclease inhibitor OS=Sus scrofa OX=982	K16634	rib
evm.model.HIC_ASM_7.835	NACHT, LR	NACHT, LRR and PYD domains-containing pr	K20865	N/
evm.model.HIC_ASM_18.135	NACHT, LR	NACHT, LRR and PYD domains-containing pr	K22626	N/
evm.model.HIC_ASM_3.1503	NACHT, LR	NACHT, LRR and PYD domains-containing pr	K12800	N/
evm.model.HIC_ASM_19.999	PREDICTEI	Scm-like with four MBT domains protein 1 OS	K10599	pr
evm.model.HIC_ASM_5.1804	PREDICTEI	Scm-like with four MBT domains protein 2 OS	K11465	se
evm.model.HIC_ASM_21.573	PREDICTEI	Ubiquitin-conjugating enzyme E2 C OS=Bos ta	K06688	ub
evm.model.HIC_ASM_12.156	ubiquitin-con	Ubiquitin-conjugating enzyme E2 C OS=Bos ta	K06688	ub
evm.model.HIC_ASM_7.979	ubiquitin-con	Ubiquitin-conjugating enzyme E2 C OS=Maca	K06688	ub
evm.model.CTG_1273.1	PREDICTEI	Methionine-R-sulfoxide reductase B2, mitocho	K07305	pe
evm.model.CTG_1273.3	PREDICTEI	Methionine-R-sulfoxide reductase B2, mitocho	K07305	pe
evm.model.HIC_ASM_2.1813	PREDICTEI	Autism susceptibility gene 2 protein OS=Homo	K23214	au
evm.model.HIC_ASM_2.1809	PREDICTEI	Autism susceptibility gene 2 protein OS=Homo	K23214	au
evm.model.HIC_ASM_13.704	protein FAM	Protein FAM136A OS=Homo sapiens OX=960	--	
evm.model.HIC_ASM_8.1014	hypothetical	Protein FAM136A OS=Bos taurus OX=9913 C	--	
evm.model.HIC_ASM_14.484	protein fam1	Protein FAM136A OS=Bos taurus OX=9913 C	--	
evm.model.HIC_ASM_3.1430	PREDICTEI	T-cell-specific guanine nucleotide triphosphate	K17072	int
evm.model.HIC_ASM_3.1433	T-cell-specifi	T-cell-specific guanine nucleotide triphosphate	K17072	int
evm.model.HIC_ASM_8.707	olfactory rec	Olfactory receptor 51D1 OS=Homo sapiens O	K04257	olf
evm.model.HIC_ASM_8.726	olfactory rec	Olfactory receptor 51L1 OS=Homo sapiens O	K04257	olf
evm.model.HIC_ASM_8.711	olfactory rec	Olfactory receptor 51E2 OS=Rattus norvegicus	K04257	olf
evm.model.HIC_ASM_8.718	olfactory rec	Olfactory receptor 51I2 OS=Homo sapiens OX	K04257	olf
evm.model.HIC_ASM_8.715	PREDICTEI	Olfactory receptor 51F1 OS=Homo sapiens O	K04257	olf
evm.model.HIC_ASM_8.721	olfactory rec	Olfactory receptor 51L1 OS=Homo sapiens O	K04257	olf
evm.model.HIC_ASM_8.708	PREDICTEI	Olfactory receptor 51E1 OS=Homo sapiens O	K04257	olf
evm.model.HIC_ASM_8.755	olfactory rec	Olfactory receptor 51I2 OS=Homo sapiens OX	K04257	olf
evm.model.HIC_ASM_20.665	--	--	K21765	pro
evm.model.CTG_1467.5	--	--	K21765	pro
evm.model.HIC_ASM_19.119	metabotropic	Metabotropic glutamate receptor 7 OS=Mus mu	K04608	me
evm.model.HIC_ASM_6.1389	metabotropic	Metabotropic glutamate receptor 1 OS=Homo s	K04603	me
evm.model.HIC_ASM_11.666	metabotropic	Metabotropic glutamate receptor 8 OS=Homo s	K04608	me
evm.model.HIC_ASM_20.124	40S ribosom	40S ribosomal protein S12 OS=Sus scrofa OX=	K02951	sm
evm.model.HIC_ASM_20.545	PREDICTEI	40S ribosomal protein S12 OS=Sus scrofa OX=	K02951	sm
evm.model.HIC_ASM_6.1303	ribosomal L7	40S ribosomal protein S12 OS=Sus scrofa OX=	K02951	sm
evm.model.HIC_ASM_13.974	LOW QUAL	40S ribosomal protein S12 OS=Gallus gallus O	K02951	sm
evm.model.HIC_ASM_8.434	hypothetical	Rho GTPase-activating protein 42 OS=Homo s	K20651	Rh
evm.model.HIC_ASM_7.565	oligophrenin	Oligophrenin-1 OS=Pongo pygmaeus OX=960	K20650	oli
evm.model.HIC_ASM_3.1074	rho GTPase-	Rho GTPase-activating protein 26 OS=Homo s	K20071	Rh
evm.model.HIC_ASM_1.113	translation fa	Eukaryotic translation initiation factor 1b OS=	K03113	tra
evm.model.CTG_1091.1	Eukaryotic tr	Eukaryotic translation initiation factor 1 (Fragn	K03113	tra
evm.model.HIC_ASM_1.1103	Eukaryotic tr	Eukaryotic translation initiation factor 1 (Fragn	K03113	tra
evm.model.HIC_ASM_13.145	PREDICTEI	Ornithine decarboxylase OS=Homo sapiens O	K01581	or
evm.model.HIC_ASM_10.713	ornithine dec	Antizyme inhibitor 2 OS=Xenopus laevis OX=	K01581	or
evm.model.HIC_ASM_18.297	PREDICTEI	SH3 and multiple ankyrin repeat domains prote	K15009	SH

evm.model.HIC_ASM_8.1722	SH3 and mul	SH3 and multiple ankyrin repeat domains prote	K15009 SH
evm.model.CTG_180.1	LOW QUAL	SH3 and multiple ankyrin repeat domains prote	K15009 SH
evm.model.HIC_ASM_4.791	Hypothetical	Ig mu chain C region membrane-bound form O	K06856 im
evm.model.HIC_ASM_4.785	immunoglobu	Immunoglobulin heavy constant epsilon OS=H	K06856 im
evm.model.HIC_ASM_4.783	IgA constant	Immunoglobulin heavy constant alpha 2 OS=H	K06856 im
evm.model.CTG_355.6	short stature	Short stature homeobox protein OS=Homo sapi	K09331 sh
evm.model.CTG_1310.8	short stature	Short stature homeobox protein OS=Homo sapi	K09331 sh
evm.model.HIC_ASM_2.1932	hypothetical	--	K10667 E3
evm.model.HIC_ASM_2.1931	hypothetical	--	--
evm.model.HIC_ASM_19.140	ankyrin repea	Ankyrin repeat domain-containing protein 26 C	K17299 PC
evm.model.CTG_773.1	coiled-coil do	Ankyrin repeat domain-containing protein 26 C	K17299 PC
evm.model.HIC_ASM_5.965	keratin, type	Keratin, type II cytoskeletal 8 OS=Bos taurus C	K07605 typ
evm.model.HIC_ASM_5.988	keratin, type	Keratin, type II cytoskeletal 7 OS=Pan troglody	K07605 typ
evm.model.CTG_445.1	keratin, type	Keratin, type II cytoskeletal 8 OS=Bos taurus C	K07605 typ
evm.model.HIC_ASM_14.1	keratin, type	Keratin, type II cytoskeletal 8 OS=Bos taurus C	K07605 typ
evm.model.HIC_ASM_13.603	catenin alpha	Catenin alpha-2 OS=Pongo abelii OX=9601 GI	K05691 ca
evm.model.HIC_ASM_14.106	catenin alpha	Catenin alpha-3 OS=Homo sapiens OX=9606 (	K05691 ca
evm.model.HIC_ASM_0.1006	PREDICTED	Glutamate receptor ionotropic, delta-2 OS=Hon	K05207 glu
evm.model.HIC_ASM_14.848	glutamate rec	Glutamate receptor ionotropic, delta-1 OS=Mus	K05206 glu
evm.model.CTG_1403.1	melanoma-as	Melanoma-associated antigen D2 OS=Pongo ab	--
evm.model.HIC_ASM_7.508	melanoma-as	Melanoma-associated antigen D2 OS=Pongo ab	--
evm.model.CTG_901.1	olfactory rec	--	K04257 olf
evm.model.CTG_1750.1	olfactory rec	--	K04257 olf
evm.model.CTG_1333.2	PREDICTED	Protein FAM156A/FAM156B OS=Homo sapie	--
evm.model.HIC_ASM_7.485	PREDICTED	Protein FAM156A/FAM156B OS=Homo sapie	--
evm.model.CTG_1333.3	PREDICTED	Protein FAM156A/FAM156B OS=Homo sapie	--
evm.model.HIC_ASM_5.1141	PREDICTED	DnaJ homolog subfamily B member 6 OS=Bos	K09512 Dn
evm.model.HIC_ASM_7.1351	dnaJ homolog	DnaJ homolog subfamily B member 6 OS=Bos	K09512 Dn
evm.model.HIC_ASM_7.273	dystrophin is	Dystrophin OS=Canis lupus familiaris OX=961	K10366 dy
evm.model.HIC_ASM_6.1377	PREDICTED	Utrophin OS=Homo sapiens OX=9606 GN=UT	K10366 dy
evm.model.CTG_509.3	--	--	--
evm.model.CTG_945.2	--	--	--
evm.model.HIC_ASM_0.1625	PREDICTED	40S ribosomal protein S16 OS=Rattus norvegic	K02960 sm
evm.model.HIC_ASM_8.130	40S ribosoma	40S ribosomal protein S16 OS=Ictalurus puncta	K02960 sm
evm.model.HIC_ASM_18.664	PREDICTED	40S ribosomal protein S16 OS=Rattus norvegic	K02960 sm
evm.model.HIC_ASM_1.327	40S ribosoma	40S ribosomal protein S16 OS=Heteropneustes	K02960 sm
evm.model.HIC_ASM_8.816	PREDICTED	Interferon-induced very large GTPase 1 OS=H	K12797 ca
evm.model.HIC_ASM_8.821	PREDICTED	Interferon-induced very large GTPase 1 OS=H	K12797 ca
evm.model.CTG_807.2	PREDICTED	Interferon-induced very large GTPase 1 OS=H	--
evm.model.CTG_807.4	PREDICTED	Interferon-induced very large GTPase 1 OS=H	K12797 ca
evm.model.HIC_ASM_8.820	interferon-ind	Interferon-induced very large GTPase 1 OS=H	--
evm.model.HIC_ASM_8.817	PREDICTED	Interferon-induced very large GTPase 1 OS=H	K12797 ca
evm.model.CTG_807.1	interferon-ind	Interferon-induced very large GTPase 1 OS=H	--
evm.model.HIC_ASM_18.113	leucine-rich r	Leucine-rich repeat-containing protein 36 OS=I	K16594 leu
evm.model.CTG_494.5	centrosomal p	Centrosomal protein of 72 kDa OS=Mus muscu	K16532 ce
evm.model.HIC_ASM_8.879	PREDICTED	Polypeptide N-acetylgalactosaminyltransferase	K00710 po
evm.model.HIC_ASM_13.363	polypeptide N	Polypeptide N-acetylgalactosaminyltransferase	K00710 po
evm.model.HIC_ASM_7.819	PREDICTED	Small ubiquitin-related modifier 2 OS=Rattus n	K12160 sm

evm.model.HIC_ASM_1.1382	PREDICTEI	Small ubiquitin-related modifier 3 OS=Bos tau	K12160	sm
evm.model.HIC_ASM_4.283	small ubiquit	Small ubiquitin-related modifier 2 OS=Rattus n	K12160	sm
evm.model.HIC_ASM_1.954	small ubiquit	Small ubiquitin-related modifier 2-A OS=Xeno	K12160	sm
evm.model.HIC_ASM_0.1510	PREDICTEI	Elongation factor 1-alpha 2 OS=Oryctolagus cu	K03231	elo
evm.model.HIC_ASM_2.423	PREDICTEI	Elongation factor 1-alpha 1 OS=Equus caballus	K03231	elo
evm.model.HIC_ASM_6.1225	elongation fa	Elongation factor 1-alpha 1 OS=Equus caballus	K03231	elo
evm.model.HIC_ASM_7.975	elongation fa	Elongation factor 1-alpha OS=Danio rerio OX=	K03231	elo
evm.model.CTG_94.2	PREDICTEI	--	K04603	me
evm.model.HIC_ASM_6.1388	PREDICTEI	--	K04603	me
evm.model.HIC_ASM_11.878	Hypothetical	T cell receptor beta variable 28 OS=Homo sapi	K10785	T-
evm.model.HIC_ASM_11.871	t-cell recepto	T cell receptor beta variable 3-1 OS=Homo sap	K10785	T-
evm.model.HIC_ASM_11.875	T-cell recept	T cell receptor beta variable 19 OS=Homo sapi	K10785	T-
evm.model.HIC_ASM_11.874	T-cell recept	T-cell receptor beta chain V region 3H.25 OS=	K10785	T-
evm.model.HIC_ASM_11.876	Hypothetical	T cell receptor beta variable 27 OS=Homo sapi	K10785	T-
evm.model.HIC_ASM_11.867	Hypothetical	T cell receptor beta variable 9 OS=Homo sapie	K10785	T-
evm.model.HIC_ASM_11.868	PREDICTEI	T cell receptor beta variable 7-2 OS=Homo sap	K10785	T-
evm.model.HIC_ASM_6.51	unnamed pro	Serine/threonine-protein kinase MARK2 OS=R	K08798	M
evm.model.HIC_ASM_19.758	MAP/microtu	MAP/microtubule affinity-regulating kinase 3 (	K08798	M
evm.model.HIC_ASM_3.1136	prefoldin sub	Prefoldin subunit 1 OS=Pongo abelii OX=9601	K09548	pr
evm.model.HIC_ASM_18.920	prefoldin sub	Prefoldin subunit 1 OS=Pongo abelii OX=9601	K09548	pr
evm.model.CTG_138.1	PREDICTEI	ATP-dependent zinc metalloprotease YME1L1	K08955	A
evm.model.CTG_510.1	ATP-depende	ATP-dependent zinc metalloprotease YME1L1	K08955	A
evm.model.HIC_ASM_8.553	PREDICTEI	Teneurin-4 OS=Homo sapiens OX=9606 GN=	K06252	ter
evm.model.HIC_ASM_3.567	teneurin-2 iso	Teneurin-2 OS=Homo sapiens OX=9606 GN=	K06252	ter
evm.model.HIC_ASM_14.348	deleted in ma	Deleted in malignant brain tumors 1 protein OS	K13912	de
evm.model.HIC_ASM_14.352	PREDICTEI	Deleted in malignant brain tumors 1 protein OS	K13912	de
evm.model.HIC_ASM_14.347	cub and zona	CUB and zona pellucida-like domain-containin	K13912	de
evm.model.CTG_1402.2	LOW QUAL	Uncharacterized protein CXorf49 homolog OS	--	
evm.model.CTG_233.1	PREDICTEI	Uncharacterized protein CXorf49 homolog OS	--	
evm.model.HIC_ASM_7.617	PREDICTEI	Uncharacterized protein CXorf49 homolog OS	--	
evm.model.HIC_ASM_7.616	PREDICTEI	Uncharacterized protein CXorf49 homolog OS	K04188	C-
evm.model.HIC_ASM_7.618	PREDICTEI	Uncharacterized protein CXorf49 homolog OS	--	
evm.model.CTG_1402.1	LOW QUAL	Uncharacterized protein CXorf49 homolog OS	--	
evm.model.CTG_1447.3	lymphocyte a	Secreted Ly-6/uPAR domain-containing protei	K06846	lyı
evm.model.HIC_ASM_15.701	PREDICTEI	Lymphocyte antigen 6E OS=Gallus gallus OX=	K06846	lyı
evm.model.HIC_ASM_19.575	GUK1 [Cerv	Guanylate kinase OS=Bos taurus OX=9913 GN	K00942	gu
evm.model.HIC_ASM_3.1471	guanylate kir	Guanylate kinase OS=Mus musculus OX=1009	K00942	gu
evm.model.HIC_ASM_11.215	lipoma HMG	LHFPL tetraspan subfamily member 3 protein (	K18631	tel
evm.model.CTG_1533.1	lipoma HMG	LHFPL tetraspan subfamily member 3 protein (	K18631	tel
evm.model.HIC_ASM_11.218	hypothetical	LHFPL tetraspan subfamily member 3 protein (	K18631	tel
evm.model.HIC_ASM_3.597	nucleophosm	Nucleophosmin OS=Bos taurus OX=9913 GN=	K11276	nu
evm.model.HIC_ASM_19.117	PREDICTEI	Nucleophosmin OS=Homo sapiens OX=9606 (	K11276	nu
evm.model.HIC_ASM_6.1082	Nucleophosn	Nucleophosmin OS=Bos taurus OX=9913 GN=	K11276	nu
evm.model.HIC_ASM_18.180	PREDICTEI	Zinc finger protein 134 OS=Homo sapiens OX	K09228	KI
evm.model.HIC_ASM_18.169	zinc finger pı	Zinc finger protein 772 OS=Homo sapiens OX	K09228	KI
evm.model.HIC_ASM_7.235	40S ribosom	40S ribosomal protein S2 (Fragment) OS=Cric	K02981	sm
evm.model.HIC_ASM_0.1509	40S ribosom	40S ribosomal protein S2 OS=Urechis caupo O	K02981	sm
evm.model.HIC_ASM_11.104	mCG7334 [I	40S ribosomal protein S2 (Fragment) OS=Cric	K02981	sm

evm.model.HIC_ASM_16.210	PREDICTEI	Nuclear transport factor 2 OS=Rattus norvegicu	K14285 NT
evm.model.HIC_ASM_21.37	Chain A, Stru	Nuclear transport factor 2 OS=Rattus norvegicu	--
evm.model.HIC_ASM_18.120	nuclear trans	Nuclear transport factor 2 OS=Rattus norvegicu	K14285 NT
evm.model.HIC_ASM_8.482	PREDICTEI	Elongation factor 1-alpha, oocyte form OS=Xe	K03231 elo
evm.model.HIC_ASM_16.212	unnamed pro	Elongation factor 1-alpha 1 OS=Oryctolagus cu	K03231 elo
evm.model.HIC_ASM_10.506	PREDICTEI	Elongation factor 1-alpha, somatic form OS=X	K03231 elo
evm.model.HIC_ASM_6.1224	eukaryotic tra	Elongation factor 1-alpha 1 OS=Oryctolagus cu	K03231 elo
evm.model.HIC_ASM_7.150	elongation fa	Elongation factor 1-alpha 1 OS=Oryctolagus cu	K03231 elo
evm.model.CTG_812.1	unnamed pro	Elongation factor 1-alpha 1 OS=Oryctolagus cu	K03231 elo
evm.model.HIC_ASM_6.927	unnamed pro	Elongation factor 1-alpha 1 OS=Oryctolagus cu	K03231 elo
evm.model.HIC_ASM_2.1645	elongation fa	Elongation factor Tu, mitochondrial OS=Bos ta	K02358 elo
evm.model.HIC_ASM_4.656	elongation fa	Elongation factor 1-alpha 1 OS=Equus caballus	K03231 elo
evm.model.HIC_ASM_21.636	--	--	--
evm.model.HIC_ASM_21.629	PREDICTEI	--	--
evm.model.HIC_ASM_2.881	PREDICTEI	60S ribosomal protein L18a OS=Homo sapiens	K02882 lar
evm.model.HIC_ASM_15.356	60S ribosoma	60S ribosomal protein L18a OS=Homo sapiens	K02882 lar
evm.model.HIC_ASM_1.542	PREDICTEI	60S ribosomal protein L18a OS=Homo sapiens	K02882 lar
evm.model.HIC_ASM_19.665	60S ribosoma	60S ribosomal protein L18a OS=Homo sapiens	K02882 lar
evm.model.HIC_ASM_10.130	60S ribosoma	60S ribosomal protein L18a OS=Rattus norveg	K02882 lar
evm.model.HIC_ASM_0.433	PREDICTEI	60S ribosomal protein L18a OS=Homo sapiens	K02882 lar
evm.model.HIC_ASM_5.501	60S ribosoma	60S ribosomal protein L27a OS=Mus musculus	K02900 lar
evm.model.HIC_ASM_4.1736	PREDICTEI	60S ribosomal protein L27a OS=Mus musculus	K02900 lar
evm.model.HIC_ASM_4.1256	60S ribosoma	60S ribosomal protein L27a OS=Bos taurus OX	K02900 lar
evm.model.HIC_ASM_17.698	60S ribosoma	60S ribosomal protein L27a OS=Xenopus laevi	K02900 lar
evm.model.HIC_ASM_12.386	60S ribosoma	60S ribosomal protein L27a OS=Bos taurus OX	K02900 lar
evm.model.HIC_ASM_6.995	PREDICTEI	60S ribosomal protein L27a OS=Bos taurus OX	K02900 lar
evm.model.HIC_ASM_8.23	unnamed pro	60S ribosomal protein L27a OS=Xenopus laevi	K02900 lar
evm.model.HIC_ASM_3.309	60S ribosoma	60S ribosomal protein L27a OS=Rattus norveg	K02900 lar
evm.model.HIC_ASM_8.843	60S ribosoma	60S ribosomal protein L27a OS=Bos taurus OX	K02900 lar
evm.model.HIC_ASM_13.101	PREDICTEI	60S ribosomal protein L27a OS=Xenopus laevi	K02900 lar
evm.model.HIC_ASM_0.1454	PREDICTEI	60S ribosomal protein L27a OS=Bos taurus OX	K02900 lar
evm.model.HIC_ASM_4.723	PREDICTEI	Kinesin-like protein KIF26A OS=Homo sapien	K10404 kin
evm.model.HIC_ASM_17.105	LOW QUAL	Kinesin-like protein KIF26B OS=Homo sapien	K10404 kin
evm.model.HIC_ASM_18.301	synaptotagmi	Synaptotagmin-3 OS=Homo sapiens OX=9606	K19903 sy
evm.model.HIC_ASM_5.656	hypothetical	Synaptotagmin-1 OS=Pongo abelii OX=9601 (	K15290 sy
evm.model.CTG_945.8	PREDICTEI	P2Y purinoceptor 8 OS=Homo sapiens OX=96	K08386 P2
evm.model.CTG_509.10	PREDICTEI	P2Y purinoceptor 8 OS=Homo sapiens OX=96	K08386 P2
evm.model.HIC_ASM_20.144	Serine/threor	Serine/threonine-protein kinase Nek8 OS=Hom	K20877 NI
evm.model.HIC_ASM_17.172	serine/threon	Serine/threonine-protein kinase Nek7 OS=Hom	K20876 NI
evm.model.HIC_ASM_7.595.1	disks large ho	Disks large homolog 3 OS=Rattus norvegicus (	K21098 dis
evm.model.HIC_ASM_7.594	disks large ho	Disks large homolog 3 OS=Rattus norvegicus (	K21098 dis
evm.model.HIC_ASM_8.532	PREDICTEI	Disks large homolog 2 OS=Rattus norvegicus (	K12075 dis
evm.model.HIC_ASM_14.20	protocadheri	Protocadherin-15 OS=Homo sapiens OX=9606	K16500 pro
evm.model.HIC_ASM_0.203	protocadheri	Protocadherin Fat 1 OS=Homo sapiens OX=96	K16506 pro
evm.model.HIC_ASM_18.261	sialic acid-bi	Sialic acid-binding Ig-like lectin 10 OS=Homo	K06749 sia
evm.model.HIC_ASM_18.251	sialic acid-bi	Myeloid cell surface antigen CD33 OS=Homo	K06549 sia
evm.model.HIC_ASM_18.245	sialic acid-bi	Sialic acid-binding Ig-like lectin 8 OS=Homo s	K06748 sia
evm.model.HIC_ASM_18.276	PREDICTEI	Myeloid cell surface antigen CD33 OS=Homo	K06473 CI

evm.model.CTG_474.2_evm.n	sialic acid-bi	Sialic acid-binding Ig-like lectin 10 OS=Homo	K06749 sia
evm.model.HIC_ASM_18.248	sialic acid-bi	Myeloid cell surface antigen CD33 OS=Homo	K06738 sia
evm.model.HIC_ASM_18.241	PREDICTEI	Sialic acid-binding Ig-like lectin 14 OS=Homo	K06549 sia
evm.model.HIC_ASM_18.275	PREDICTEI	Myeloid cell surface antigen CD33 OS=Homo	K06473 CI
evm.model.HIC_ASM_18.280	PREDICTEI	Sialic acid-binding Ig-like lectin 8 OS=Homo s	K06748 sia
evm.model.CTG_474.1	PREDICTEI	Sialic acid-binding Ig-like lectin 14 OS=Homo	K06549 sia
evm.model.HIC_ASM_18.242	PREDICTEI	Sialic acid-binding Ig-like lectin 14 OS=Homo	K06549 sia
evm.model.HIC_ASM_18.272	sialic acid-bi	Sialic acid-binding Ig-like lectin 5 OS=Homo s	K06549 sia
evm.model.HIC_ASM_18.252	sialic acid-bi	Sialic acid-binding Ig-like lectin 10 OS=Homo	K06749 sia
evm.model.HIC_ASM_20.130	keratin-assoc	Keratin-associated protein 3-2 OS=Ovis aries C	--
evm.model.HIC_ASM_20.130	keratin-assoc	Keratin-associated protein 3-1 OS=Ovis aries C	--
evm.model.CTG_1512.2	keratin-assoc	Keratin-associated protein 3-1 OS=Ovis aries C	--
evm.model.HIC_ASM_20.131	keratin-assoc	Keratin-associated protein 3-3 OS=Bos taurus (	--
evm.model.CTG_1512.1	keratin-assoc	Keratin-associated protein 3-2 OS=Ovis aries C	--
evm.model.HIC_ASM_9.1043	PREDICTEI	GTP:AMP phosphotransferase AK3, mitochon	K00944 nu
evm.model.HIC_ASM_3.1370	PREDICTEI	GTP:AMP phosphotransferase AK3, mitochon	K00944 nu
evm.model.HIC_ASM_9.1049	PREDICTEI	GTP:AMP phosphotransferase AK3, mitochon	K00944 nu
evm.model.HIC_ASM_20.137	hypothetical	60S ribosomal protein L19 OS=Rattus norvegic	K02885 lar
evm.model.HIC_ASM_15.275	60S ribosoma	60S ribosomal protein L19 OS=Rattus norvegic	K02885 lar
evm.model.HIC_ASM_6.908	PREDICTEI	60S ribosomal protein L19 OS=Rattus norvegic	K02885 lar
evm.model.HIC_ASM_11.855	LOW QUAL	60S ribosomal protein L19 OS=Rattus norvegic	K02885 lar
evm.model.HIC_ASM_13.595	PREDICTEI	60S ribosomal protein L19 OS=Rattus norvegic	K02885 lar
evm.model.HIC_ASM_1.1085	development	Developmental pluripotency-associated protein	--
evm.model.HIC_ASM_17.547	development	--	--
evm.model.HIC_ASM_1.740	Hypothetical	--	--
evm.model.HIC_ASM_13.306	Hypothetical	--	--
evm.model.HIC_ASM_3.1195	Hypothetical	--	K11426 SE
evm.model.HIC_ASM_7.874	Intraflagellar	--	K11426 SE
evm.model.HIC_ASM_10.125	Hypothetical	--	K11426 SE
evm.model.HIC_ASM_4.246	Intraflagellar	--	K11426 SE
evm.model.CTG_161.2	40S ribosoma	40S ribosomal protein S3a (Fragment) OS=Feli	K02984 sm
evm.model.HIC_ASM_0.497	40S ribosoma	40S ribosomal protein S3a (Fragment) OS=Feli	K02984 sm
evm.model.HIC_ASM_0.496	40S ribosoma	40S ribosomal protein S3a OS=Homo sapiens (	K02984 sm
evm.model.HIC_ASM_0.688	40S ribosoma	40S ribosomal protein S3a OS=Homo sapiens (	K02984 sm
evm.model.HIC_ASM_7.350	ribosmal prot	40S ribosomal protein S3a OS=Homo sapiens (	K02984 sm
evm.model.HIC_ASM_4.228	PREDICTEI	Sodium/calcium exchanger 3 OS=Mus musculu	K05849 so
evm.model.HIC_ASM_13.438	PREDICTEI	Sodium/calcium exchanger 1 OS=Felis catus O	K05849 so
evm.model.HIC_ASM_2.1596	PREDICTEI	Leucine carboxyl methyltransferase 1 OS=Bos	K18203 [pl
evm.model.CTG_1138.2	PREDICTEI	Leucine carboxyl methyltransferase 1 OS=Bos	K18203 [pl
evm.model.HIC_ASM_12.965	histone H3.3	Histone H3.2 OS=Cricetulus longicaudatus OX	K11253 his
evm.model.HIC_ASM_7.917	PREDICTEI	Histone H3.3 OS=Trichinella pseudospiralis O	K11253 his
evm.model.HIC_ASM_4.1413	histone H3.3	Histone H3.Y OS=Homo sapiens OX=9606 PE	K11253 his
evm.model.HIC_ASM_20.912	Chain E, Cry	Histone H3.3 OS=Xenopus tropicalis OX=836	K11253 his
evm.model.HIC_ASM_17.102	Chain E, Cry	Histone H3.3 OS=Xenopus tropicalis OX=836	K11253 his
evm.model.HIC_ASM_2.555	PREDICTEI	Histone H3.3 OS=Pongo abelii OX=9601 PE=	K11253 his
evm.model.HIC_ASM_20.138	60S ribosoma	60S ribosomal protein L23 OS=Rattus norvegic	K02894 lar
evm.model.HIC_ASM_18.899	60S ribosoma	60S ribosomal protein L23 OS=Danio rerio OX	K02894 lar
evm.model.HIC_ASM_8.75	PREDICTEI	60S ribosomal protein L23 OS=Pongo abelii O	K02894 lar

evm.model.HIC_ASM_4.73	PREDICTEI	60S ribosomal protein L23 OS=Danio rerio OX	K02894	lar
evm.model.HIC_ASM_15.694	PREDICTEI	Ly6/PLAUR domain-containing protein 2 OS=	K06846	lyı
evm.model.HIC_ASM_15.693	Hypothetical	Secreted Ly-6/uPAR-related protein 1 OS=Mus	K06846	lyı
evm.model.CTG_1447.10	Hypothetical	Secreted Ly-6/uPAR-related protein 1 OS=Mus	K06846	lyı
evm.model.CTG_1447.9	PREDICTEI	Ly6/PLAUR domain-containing protein 2 OS=	K06846	lyı
evm.model.HIC_ASM_13.618	PREDICTEI	60S ribosomal protein L12 OS=Homo sapiens (	K02870	lar
evm.model.HIC_ASM_15.373	rCG64424 [	60S ribosomal protein L12 OS=Mus musculus	K02870	lar
evm.model.HIC_ASM_9.627	hypothetical	60S ribosomal protein L12 OS=Mus musculus	K02870	lar
evm.model.HIC_ASM_7.112	PREDICTEI	60S ribosomal protein L12 OS=Homo sapiens (	K02870	lar
evm.model.HIC_ASM_19.111	60S ribosoma	60S ribosomal protein L12 OS=Rattus norvegic	K02870	lar
evm.model.HIC_ASM_5.671	PREDICTEI	60S ribosomal protein L12 OS=Mus musculus	K02870	lar
evm.model.HIC_ASM_21.89	60S ribosoma	60S ribosomal protein L12 OS=Mus musculus	K02870	lar
evm.model.HIC_ASM_9.299	PREDICTEI	60S ribosomal protein L12 OS=Homo sapiens (	K02870	lar
evm.model.HIC_ASM_4.1199	hypothetical	60S ribosomal protein L12 OS=Mus musculus	K02870	lar
evm.model.HIC_ASM_10.611	60s ribosoma	60S ribosomal protein L12 OS=Mus musculus	K02870	lar
evm.model.HIC_ASM_0.819	60S ribosoma	60S ribosomal protein L12 OS=Mus musculus	K02870	lar
evm.model.HIC_ASM_20.729	PREDICTEI	60S ribosomal protein L12 OS=Mus musculus	K02870	lar
evm.model.HIC_ASM_16.598	hypothetical	60S ribosomal protein L12 OS=Chinchilla lani	K02870	lar
evm.model.HIC_ASM_11.933	60S ribosoma	60S ribosomal protein L12 OS=Homo sapiens (	K02870	lar
evm.model.HIC_ASM_14.771	60S ribosoma	60S ribosomal protein L12 OS=Mus musculus	K02870	lar
evm.model.HIC_ASM_20.851	PREDICTEI	60S ribosomal protein L12 OS=Homo sapiens (	K02870	lar
evm.model.HIC_ASM_17.108	PREDICTEI	60S ribosomal protein L12 OS=Mus musculus	K02870	lar
evm.model.HIC_ASM_14.395	60S ribosoma	60S ribosomal protein L12 OS=Rattus norvegic	K02870	lar
evm.model.HIC_ASM_0.67	60S ribosoma	60S ribosomal protein L12 OS=Rattus norvegic	K02870	lar
evm.model.HIC_ASM_7.898	60S ribosoma	60S ribosomal protein L12 OS=Rattus norvegic	K02870	lar
evm.model.CTG_490.1	rCG64424 [	60S ribosomal protein L12 OS=Mus musculus	K02870	lar
evm.model.HIC_ASM_18.5	PREDICTEI	60S ribosomal protein L12 OS=Homo sapiens (	K02870	lar
evm.model.HIC_ASM_15.305	60S ribosoma	60S ribosomal protein L12 OS=Homo sapiens (	K02870	lar
evm.model.HIC_ASM_17.762	PREDICTEI	--	K06448	T-
evm.model.HIC_ASM_17.769	T-cell surfac	T-cell surface glycoprotein CD1b OS=Aotus na	K06448	T-
evm.model.HIC_ASM_10.150	PREDICTEI	Calcium-binding mitochondrial carrier protein	K14684	so
evm.model.HIC_ASM_10.150	calcium-bind	Calcium-binding mitochondrial carrier protein	K14684	so
evm.model.HIC_ASM_10.150	calcium-bind	Calcium-binding mitochondrial carrier protein	K14684	so
evm.model.HIC_ASM_4.1767	hypothetical	60S ribosomal protein L23a OS=Rattus norveg	K02893	lar
evm.model.HIC_ASM_20.363	PREDICTEI	60S ribosomal protein L23a OS=Rattus norveg	K02893	lar
evm.model.HIC_ASM_5.1178	ribosomal pr	60S ribosomal protein L23a OS=Rattus norveg	K02893	lar
evm.model.HIC_ASM_17.210	plexin-A2 isc	Plexin-A2 OS=Homo sapiens OX=9606 GN=P	K06820	plc
evm.model.HIC_ASM_11.746	plexin-A4 isc	Plexin-A4 OS=Mus musculus OX=10090 GN=	K06820	plc
evm.model.HIC_ASM_2.2006	ATP synthase	ATP synthase subunit f, mitochondrial OS=Sus	K02130	F-
evm.model.HIC_ASM_2.858	ATP synthase	ATP synthase subunit f, mitochondrial OS=Bos	K02130	F-
evm.model.HIC_ASM_4.1363	ATP synthase	ATP synthase subunit f, mitochondrial OS=Sus	K02130	F-
evm.model.HIC_ASM_0.635	ATP synthase	ATP synthase subunit f, mitochondrial OS=Sus	K02130	F-
evm.model.HIC_ASM_1.550	ATP synthase	ATP synthase subunit f, mitochondrial OS=Bos	K02130	F-
evm.model.HIC_ASM_6.885	Hypothetical	ATP synthase subunit f, mitochondrial OS=Bos	K02130	F-
evm.model.HIC_ASM_4.1393	ATP synthase	ATP synthase subunit f, mitochondrial OS=Bos	K02130	F-
evm.model.HIC_ASM_0.1008	ATP synthase	ATP synthase subunit f, mitochondrial OS=Bos	K02130	F-
evm.model.HIC_ASM_17.91	PREDICTEI	60S ribosomal protein L9 OS=Macaca fascicul	K02940	lar
evm.model.HIC_ASM_13.608	LOW QUAL	60S ribosomal protein L9 OS=Rattus norvegicu	K02940	lar

evm.model.HIC_ASM_3.1308	LOW QUAL	60S ribosomal protein L9 OS=Bos taurus OX=	K02940	la
evm.model.HIC_ASM_2.806	PREDICTEI	Complement component 1 Q subcomponent-bi	K15414	co
evm.model.HIC_ASM_20.637	complement	Complement component 1 Q subcomponent-bi	K15414	co
evm.model.HIC_ASM_4.1376	complement	Complement component 1 Q subcomponent-bi	K15414	co
evm.model.HIC_ASM_2.769	complement	Complement component 1 Q subcomponent-bi	K15414	co
evm.model.HIC_ASM_0.1239	glyceraldehy	Glyceraldehyde-3-phosphate dehydrogenase O	K00134	gl
evm.model.HIC_ASM_5.1182	glyceraldehy	Glyceraldehyde-3-phosphate dehydrogenase O	K00134	gl
evm.model.HIC_ASM_18.898	PREDICTEI	Glyceraldehyde-3-phosphate dehydrogenase (F	K00134	gl
evm.model.HIC_ASM_0.780	glyceraldehy	Glyceraldehyde-3-phosphate dehydrogenase O	K00134	gl
evm.model.HIC_ASM_6.1043	glyceraldehy	Glyceraldehyde-3-phosphate dehydrogenase O	K00134	gl
evm.model.HIC_ASM_13.531	Ig kappa chai	Immunoglobulin kappa light chain OS=Homo s	K06554	im
evm.model.HIC_ASM_12.827	hypothetical	Immunoglobulin lambda-like polypeptide 1 OS	K06554	im
evm.model.HIC_ASM_12.826	Hypothetical	Immunoglobulin lambda constant 1 OS=Homo	K06554	im
evm.model.HIC_ASM_12.831	PREDICTEI	Ig lambda chain C region OS=Sus scrofa OX=	K06554	im
evm.model.HIC_ASM_12.823	PREDICTEI	Immunoglobulin lambda constant 1 OS=Homo	K06554	im
evm.model.HIC_ASM_18.598	carcinoembry	Carcinoembryonic antigen-related cell adhesio	K06499	ca
evm.model.HIC_ASM_18.572	PREDICTEI	Carcinoembryonic antigen-related cell adhesio	K06499	ca
evm.model.HIC_ASM_19.118	PREDICTEI	Metabotropic glutamate receptor 7 OS=Rattus	K04608	m
evm.model.HIC_ASM_6.518	PREDICTEI	Metabotropic glutamate receptor 4 OS=Macac	K04607	m
evm.model.HIC_ASM_11.667	PREDICTEI	Metabotropic glutamate receptor 8 OS=Homo	K04608	m
evm.model.HIC_ASM_8.500	glutamate rec	Metabotropic glutamate receptor 5 OS=Homo	K04604	m
evm.model.HIC_ASM_6.1387	PREDICTEI	Metabotropic glutamate receptor 1 OS=Homo	K04603	m
evm.model.CTG_927.1	PREDICTEI	Pachytene checkpoint protein 2 homolog OS=S	K22399	pa
evm.model.CTG_830.1	pachytene ch	Pachytene checkpoint protein 2 homolog OS=F	K22399	pa
evm.model.CTG_1043.3	PREDICTEI	Pachytene checkpoint protein 2 homolog OS=S	K22399	pa
evm.model.CTG_1612.1	pachytene ch	Pachytene checkpoint protein 2 homolog OS=C	K22399	pa
evm.model.HIC_ASM_3.1	PREDICTEI	Pachytene checkpoint protein 2 homolog OS=C	K22399	pa
evm.model.HIC_ASM_5.1350	PREDICTEI	40S ribosomal protein S20 OS=Rattus norvegi	K02969	sn
evm.model.HIC_ASM_20.320	PREDICTEI	40S ribosomal protein S20 OS=Xenopus laevis	K02969	sn
evm.model.HIC_ASM_2.611	hypothetical	40S ribosomal protein S20 OS=Sus scrofa OX=	K02969	sn
evm.model.HIC_ASM_8.257	TPA: ribosor	40S ribosomal protein S20 OS=Sus scrofa OX=	K02969	sn
evm.model.HIC_ASM_15.64	30S ribosom	40S ribosomal protein S20 OS=Rattus norvegi	K02969	sn
evm.model.HIC_ASM_14.933	PREDICTEI	40S ribosomal protein S20 OS=Rattus norvegi	K02969	sn
evm.model.HIC_ASM_10.623	PREDICTEI	40S ribosomal protein S20 OS=Pongo abelii O	K02969	sn
evm.model.HIC_ASM_18.19	LOW QUAL	Zinc finger protein 677 OS=Homo sapiens OX	K09228	KI
evm.model.HIC_ASM_18.20	LOW QUAL	Zinc finger protein 677 OS=Homo sapiens OX	K09228	KI
evm.model.HIC_ASM_1.482	translocon-as	Translocon-associated protein subunit gamma (	K13251	tra
evm.model.HIC_ASM_21.9	PREDICTEI	Translocon-associated protein subunit gamma (	K13251	tra
evm.model.HIC_ASM_21.10	translocon-as	Translocon-associated protein subunit gamma (	K13251	tra
evm.model.HIC_ASM_2.1957	PREDICTEI	Insulin receptor substrate 1 OS=Xenopus tropi	K17445	ins
evm.model.HIC_ASM_2.269	insulin recep	Insulin receptor substrate 1 OS=Homo sapiens	K16172	ins
evm.model.HIC_ASM_7.35	neuroligin-4,	Neuroligin-4, X-linked OS=Homo sapiens OX=	K07378	ne
evm.model.HIC_ASM_1.585	PREDICTEI	Neuroligin-1 OS=Rattus norvegicus OX=1011	K07378	ne
evm.model.HIC_ASM_2.622	stathmin-like	Stathmin OS=Sus scrofa OX=9823 GN=STMN	K04381	sta
evm.model.HIC_ASM_10.567	stathmin isof	Stathmin OS=Sus scrofa OX=9823 GN=STMN	K04381	sta
evm.model.HIC_ASM_0.595	tripartite mot	Tripartite motif-containing protein 60 OS=Hon	K12029	tri
evm.model.HIC_ASM_3.1484	PREDICTEI	E3 ubiquitin-protein ligase TRIM17 OS=Bos ta	K12007	tri
evm.model.HIC_ASM_5.486	PREDICTEI	Mitochondrial 10-formyltetrahydrofolate dehyd	K00289	fo

evm.model.HIC_ASM_9.972	PREDICTEI	Retinal dehydrogenase 1 OS=Ovis aries OX=9	K07249 ret
evm.model.HIC_ASM_1.1086	development	Developmental pluripotency-associated protein	--
evm.model.HIC_ASM_4.803	PREDICTEI	Developmental pluripotency-associated protein	--
evm.model.HIC_ASM_13.586	LOW QUAL	Dynein heavy chain 6, axonemal OS=Homo saj	K10408 dy
evm.model.HIC_ASM_2.547.1	PREDICTEI	Dynein heavy chain 7, axonemal OS=Homo saj	K10408 dy
evm.model.HIC_ASM_11.437	dynein heavy	Dynein heavy chain 11, axonemal OS=Homo sa	K10408 dy
evm.model.HIC_ASM_20.437	PREDICTEI	Dynein heavy chain 9, axonemal OS=Homo saj	K10408 dy
evm.model.CTG_774.13	PREDICTEI	Msx2-interacting protein OS=Homo sapiens O	--
evm.model.CTG_774.9	PREDICTEI	Msx2-interacting protein OS=Homo sapiens O	--
evm.model.HIC_ASM_2.533	10 kDa heat	10 kDa heat shock protein, mitochondrial OS=I	K04078 ch
evm.model.HIC_ASM_11.147	10 kDa heat	10 kDa heat shock protein, mitochondrial OS=(	K04078 ch
evm.model.HIC_ASM_3.964	10 kDa heat	10 kDa heat shock protein, mitochondrial OS=(	K04078 ch
evm.model.HIC_ASM_4.820	PREDICTEI	10 kDa heat shock protein, mitochondrial OS=I	K04078 ch
evm.model.HIC_ASM_7.688	10 kDa heat	10 kDa heat shock protein, mitochondrial OS=I	K04078 ch
evm.model.HIC_ASM_11.651	PREDICTEI	NADH dehydrogenase [ubiquinone] 1 alpha sul	K03949 N/
evm.model.HIC_ASM_18.115	PREDICTEI	NADH dehydrogenase [ubiquinone] 1 alpha sul	K03949 N/
evm.model.HIC_ASM_1.1420	vomeronasal	Olfactory receptor class A-like protein 1 OS=D	K04614 vo
evm.model.HIC_ASM_18.422	PREDICTEI	Vomeronasal type-1 receptor 2 OS=Homo sapi	K04614 vo
evm.model.HIC_ASM_0.1086	heterogeneou	Heterogeneous nuclear ribonucleoprotein D-lik	K13044 he
evm.model.HIC_ASM_7.1141	heterogeneou	Heterogeneous nuclear ribonucleoprotein D-lik	K13044 he
evm.model.HIC_ASM_17.525	heterogeneou	Heterogeneous nuclear ribonucleoprotein D-lik	K13044 he
evm.model.HIC_ASM_5.589	39S ribosom	39S ribosomal protein L42, mitochondrial OS=	K17423 lar
evm.model.HIC_ASM_6.56	PREDICTEI	39S ribosomal protein L42, mitochondrial OS=	K17423 lar
evm.model.HIC_ASM_0.1158	PREDICTEI	39S ribosomal protein L42, mitochondrial OS=	K17423 lar
evm.model.HIC_ASM_0.1338	PREDICTEI	39S ribosomal protein L42, mitochondrial OS=	K17423 lar
evm.model.HIC_ASM_18.237	39S ribosom	39S ribosomal protein L42, mitochondrial OS=	K17423 lar
evm.model.HIC_ASM_0.662	39S ribosom	39S ribosomal protein L42, mitochondrial OS=	K17423 lar
evm.model.CTG_667.6	cohesin subu	Cohesin subunit SA-1 OS=Xenopus laevis OX=	K06671 co
evm.model.CTG_735.13	PREDICTEI	Cohesin subunit SA-1 OS=Homo sapiens OX=	K06671 co
evm.model.CTG_1208.4	--	--	--
evm.model.CTG_907.1	--	--	--
evm.model.HIC_ASM_13.732	transmembra	Transmembrane protein 59 OS=Sus scrofa OX=	--
evm.model.HIC_ASM_10.112	PREDICTEI	Transmembrane protein 59 OS=Bos taurus OX	--
evm.model.HIC_ASM_6.526	PREDICTEI	40S ribosomal protein S10 OS=Homo sapiens (	K02947 sm
evm.model.HIC_ASM_17.252	hypothetical	40S ribosomal protein S10 OS=Rattus norvegic	K02947 sm
evm.model.HIC_ASM_18.145	PREDICTEI	Zinc finger protein 28 homolog OS=Homo sapi	K09228 KI
evm.model.HIC_ASM_18.743	PREDICTEI	Zinc finger protein 82 homolog OS=Homo sapi	K09228 KI
evm.model.HIC_ASM_18.733	zinc finger pı	Zinc finger protein 570 OS=Homo sapiens OX=	K09228 KI
evm.model.HIC_ASM_18.731	hypothetical	Zinc finger protein 527 OS=Homo sapiens OX=	K09228 KI
evm.model.HIC_ASM_20.828	PREDICTEI	Dynein heavy chain 17, axonemal OS=Homo sa	K10408 dy
evm.model.HIC_ASM_11.434	PREDICTEI	Dynein heavy chain 11, axonemal OS=Homo sa	K10408 dy
evm.model.HIC_ASM_18.18	--	--	--
evm.model.HIC_ASM_18.17	--	--	--
evm.model.HIC_ASM_10.150	PREDICTEI	Guanine nucleotide exchange factor VAV3 OS	K05730 gu
evm.model.HIC_ASM_10.150	PREDICTEI	Guanine nucleotide exchange factor VAV3 OS	K05730 gu
evm.model.HIC_ASM_9.97	guanine nucl	Guanine nucleotide exchange factor VAV2 OS	K05730 gu
evm.model.HIC_ASM_21.950	elongation fa	Elongation factor 1-alpha 2 OS=Oryctolagus cu	K03231 elc
evm.model.HIC_ASM_2.874	PREDICTEI	Elongation factor 1-alpha 1 OS=Rattus norvegi	K03231 elc

evm.model.HIC_ASM_8.1323	unnamed pro	Elongation factor 1-alpha 1 OS=Rattus norvegi	K03231	elo
evm.model.CTG_513.2	PREDICTEI	40S ribosomal protein S21 OS=Xenopus tropic	K02971	sm
evm.model.HIC_ASM_21.905	40S ribosom	40S ribosomal protein S21 OS=Sus scrofa OX=	K02971	sm
evm.model.HIC_ASM_4.1169	PREDICTEI	40S ribosomal protein S21 OS=Xenopus tropic	K02971	sm
evm.model.HIC_ASM_16.207	PREDICTEI	40S ribosomal protein S21 OS=Sus scrofa OX=	K02971	sm
evm.model.HIC_ASM_0.1308	PREDICTEI	40S ribosomal protein S21 OS=Sus scrofa OX=	K02971	sm
evm.model.HIC_ASM_16.268	polyubiquitin	Ubiquitin (Fragment) OS=Lumbricus terrestris	K08770	ub
evm.model.HIC_ASM_9.622	polyubiquitin	Ubiquitin OS=Lupinus polyphyllus OX=3874 I	K02927	lar
evm.model.HIC_ASM_4.1025	T-cell recept	T cell receptor alpha variable 38-1 OS=Homo s	K10784	T
evm.model.HIC_ASM_4.1042	Hypothetical	T cell receptor alpha variable 14/delta variable	K10784	T
evm.model.HIC_ASM_5.1066	PREDICTEI	Tubulin alpha-1B chain OS=Rattus norvegicus	K07374	tul
evm.model.HIC_ASM_6.1240	tubulin alpha	Tubulin alpha-1C chain OS=Mus musculus OX	K07374	tul
evm.model.HIC_ASM_4.1024	hCG181212	T cell receptor alpha variable 40 OS=Homo sap	K10784	T
evm.model.HIC_ASM_4.1045	uncharacteriz	T cell receptor alpha variable 8-6 OS=Homo sa	K10784	T
evm.model.HIC_ASM_4.1037	PREDICTEI	T cell receptor alpha variable 16 OS=Homo sap	K10784	T
evm.model.HIC_ASM_4.1034	T-cell recept	T cell receptor alpha variable 8-3 OS=Homo sa	K10784	T
evm.model.HIC_ASM_4.1032	Hypothetical	T-cell receptor alpha chain V region PHDS58 (	K10784	T
evm.model.HIC_ASM_15.201	60S ribosom	60S ribosomal protein L23a OS=Rattus norveg	K02893	lar
evm.model.HIC_ASM_0.1090	hypothetical	60S ribosomal protein L23a OS=Rattus norveg	K02893	lar
evm.model.HIC_ASM_19.381	60S ribosom	60S ribosomal protein L23a OS=Rattus norveg	K02893	lar
evm.model.HIC_ASM_20.142	60S ribosom	60S ribosomal protein L23a OS=Rattus norveg	K02893	lar
evm.model.HIC_ASM_1.1233	60S ribosom	60S ribosomal protein L23a OS=Rattus norveg	K02893	lar
evm.model.HIC_ASM_19.379	60S ribosom	60S ribosomal protein L23a OS=Rattus norveg	K02893	lar
evm.model.HIC_ASM_4.828	mCG104725	60S ribosomal protein L23a OS=Rattus norveg	K02893	lar
evm.model.HIC_ASM_7.405	RPL23A [C	60S ribosomal protein L23a OS=Rattus norveg	K02893	lar
evm.model.HIC_ASM_21.182	PREDICTEI	60S ribosomal protein L23a OS=Rattus norveg	K02893	lar
evm.model.HIC_ASM_19.378	60S ribosom	60S ribosomal protein L23a OS=Rattus norveg	K02893	lar
evm.model.HIC_ASM_5.1524	PREDICTEI	60S ribosomal protein L23a OS=Rattus norveg	K02893	lar
evm.model.HIC_ASM_2.889	60S ribosom	60S ribosomal protein L23a OS=Rattus norveg	K02893	lar
evm.model.HIC_ASM_18.246	cytoplasmic t	Cytoplasmic tRNA 2-thiolation protein 1 OS=E	K14168	cy
evm.model.HIC_ASM_18.244	cytoplasmic t	Cytoplasmic tRNA 2-thiolation protein 1 OS=E	K14168	cy
evm.model.HIC_ASM_5.1469	hypothetical	60S ribosomal protein L29 OS=Macaca fascicu	K02905	lar
evm.model.HIC_ASM_3.1253	60S ribosom	60S ribosomal protein L29 OS=Macaca fascicu	K02905	lar
evm.model.HIC_ASM_15.728	gasdermin-D	Gasdermin-D OS=Homo sapiens OX=9606 GN	K20917	ga
evm.model.CTG_1021.1	gasdermin-C	Gasdermin-C OS=Homo sapiens OX=9606 GN	K22143	ga
evm.model.CTG_1021.3	gasdermin-C	Gasdermin-C OS=Homo sapiens OX=9606 GN	K22143	ga
evm.model.HIC_ASM_20.134	gasdermin-A	Gasdermin-A OS=Mus musculus OX=10090 G	K22141	ga
evm.model.CTG_1021.2	gasdermin-C	Gasdermin-C OS=Homo sapiens OX=9606 GN	K22143	ga
evm.model.HIC_ASM_15.538	gasdermin-C	Gasdermin-C OS=Homo sapiens OX=9606 GN	K22143	ga
evm.model.HIC_ASM_10.120	dedicator of	Dedicator of cytokinesis protein 7 OS=Homo s	K21852	de
evm.model.HIC_ASM_7.902	dedicator of	Dedicator of cytokinesis protein 11 OS=Homo	K21853	de
evm.model.HIC_ASM_9.1012	PREDICTEI	Dedicator of cytokinesis protein 8 OS=Homo s	K21852	de
evm.model.HIC_ASM_13.840	neurexin-1-b	Neurexin-1-beta OS=Mus musculus OX=1009	K07377	ne
evm.model.HIC_ASM_4.381	neurexin 3 is	Neurexin-3-beta OS=Homo sapiens OX=9606	K07377	ne
evm.model.HIC_ASM_4.380	Neurexin-3-b	Neurexin-3-beta OS=Bos taurus OX=9913 GN	K07377	ne
evm.model.HIC_ASM_15.190	L-lactate deh	L-lactate dehydrogenase B chain OS=Macaca f	K00016	L-
evm.model.HIC_ASM_8.1130	L-lactate deh	L-lactate dehydrogenase B chain OS=Pan trogl	K00016	L-
evm.model.HIC_ASM_4.1106	formin-1 isof	Formin-1 OS=Homo sapiens OX=9606 GN=FI	K10367	for

evm.model.HIC_ASM_17.109	PREDICTEI	Formin-2 OS=Homo sapiens OX=9606 GN=FI	K02184 fo
evm.model.HIC_ASM_0.937	translationall	Translationally-controlled tumor protein OS=O	--
evm.model.HIC_ASM_3.1075	translationall	Translationally-controlled tumor protein OS=O	K01115 ph
evm.model.HIC_ASM_16.342	PREDICTEI	Translationally-controlled tumor protein OS=S	K01115 ph
evm.model.HIC_ASM_7.970	testis-expres	Putative testis-expressed protein 13C OS=Hom	--
evm.model.HIC_ASM_7.964	PREDICTEI	Testis-expressed protein 13D OS=Homo sapier	--
evm.model.HIC_ASM_7.938	testis-expres	Putative testis-expressed protein 13C OS=Hom	--
evm.model.HIC_ASM_3.645	ADP-ribosyl	ADP-ribosylation factor-like protein 2-binding	K16742 AI
evm.model.HIC_ASM_5.969	PREDICTEI	ADP-ribosylation factor-like protein 2-binding	K16742 AI
evm.model.HIC_ASM_18.102	PREDICTEI	ADP-ribosylation factor-like protein 2-binding	K16742 AI
evm.model.CTG_1135.2	LOW QUAL	Oligosaccharyltransferase complex subunit OS	--
evm.model.HIC_ASM_0.911	PREDICTEI	Oligosaccharyltransferase complex subunit OS	--
evm.model.HIC_ASM_0.1083	LOW QUAL	Oligosaccharyltransferase complex subunit OS	--
evm.model.HIC_ASM_6.287	oligosacchar	Oligosaccharyltransferase complex subunit OS	--
evm.model.CTG_1630.2	--	--	--
evm.model.HIC_ASM_3.339	--	--	--
evm.model.CTG_945.6	ADP/ATP tr	ADP/ATP translocase 3 OS=Homo sapiens OX	K05863 so
evm.model.HIC_ASM_3.317	hypothetical	ADP/ATP translocase 3 OS=Sus scrofa OX=9	K05863 so
evm.model.CTG_509.7	ADP/ATP tr	ADP/ATP translocase 3 OS=Homo sapiens OX	K05863 so
evm.model.HIC_ASM_14.109	LOW QUAL	Glyceraldehyde-3-phosphate dehydrogenase O	K00134 gl
evm.model.HIC_ASM_5.1183	glyceraldehy	Glyceraldehyde-3-phosphate dehydrogenase O	K00134 gl
evm.model.HIC_ASM_14.294	glyceraldehy	Glyceraldehyde-3-phosphate dehydrogenase O	K00134 gl
evm.model.HIC_ASM_5.1453	glyceraldehy	Glyceraldehyde-3-phosphate dehydrogenase O	K00134 gl
evm.model.HIC_ASM_1.624	LOW QUAL	Glyceraldehyde-3-phosphate dehydrogenase O	K00134 gl
evm.model.HIC_ASM_9.561	glyceraldehy	Glyceraldehyde-3-phosphate dehydrogenase (F	K00134 gl
evm.model.HIC_ASM_9.562	PREDICTEI	Glyceraldehyde-3-phosphate dehydrogenase O	K00134 gl
evm.model.HIC_ASM_18.788	Glyceraldehy	Glyceraldehyde-3-phosphate dehydrogenase, te	K10705 gl
evm.model.HIC_ASM_0.781	hypothetical	Glyceraldehyde-3-phosphate dehydrogenase O	K00134 gl
evm.model.HIC_ASM_15.118	glyceraldehy	Glyceraldehyde-3-phosphate dehydrogenase O	K00134 gl
evm.model.HIC_ASM_7.842	glyceraldehy	Glyceraldehyde-3-phosphate dehydrogenase O	K00134 gl
evm.model.HIC_ASM_7.843	PREDICTEI	Glyceraldehyde-3-phosphate dehydrogenase (F	K00134 gl
evm.model.HIC_ASM_20.76	PREDICTEI	Glyceraldehyde-3-phosphate dehydrogenase O	K00134 gl
evm.model.HIC_ASM_3.1319	glyceraldehy	Glyceraldehyde-3-phosphate dehydrogenase O	K00134 gl
evm.model.HIC_ASM_17.109	glyceraldehy	Glyceraldehyde-3-phosphate dehydrogenase O	K00134 gl
evm.model.HIC_ASM_4.886	PREDICTEI	Glyceraldehyde-3-phosphate dehydrogenase (F	K00134 gl
evm.model.HIC_ASM_6.648	CMRF35-lik	Trem-like transcript 4 protein OS=Mus muscul	K14364 tre
evm.model.HIC_ASM_6.653	natural cytot	Trem-like transcript 4 protein OS=Mus muscul	K06742 na
evm.model.HIC_ASM_6.654	Hypothetical	Natural cytotoxicity triggering receptor 2 OS=I	K06742 na
evm.model.HIC_ASM_11.625	ankyrin repe	Ankyrin repeat domain-containing protein 7 OS	K17299 PC
evm.model.CTG_908.1	ankyrin repe	Ankyrin repeat domain-containing protein 26 C	K17299 PC
evm.model.CTG_598.1	ankyrin repe	Ankyrin repeat domain-containing protein 26 C	K17299 PC
evm.model.HIC_ASM_5.1634	ankyrin repe	Ankyrin repeat domain-containing protein 26 C	K17299 PC
evm.model.HIC_ASM_5.1636	ankyrin repe	Ankyrin repeat domain-containing protein 26 C	K17299 PC
evm.model.HIC_ASM_7.1145	PREDICTEI	ATP synthase F(0) complex subunit C2, mitocl	K02128 F-
evm.model.HIC_ASM_9.971	PREDICTEI	ATP synthase F(0) complex subunit C1, mitocl	K02128 F-
evm.model.HIC_ASM_1.1101	PREDICTEI	ATP synthase F(0) complex subunit C2, mitocl	K02128 F-
evm.model.HIC_ASM_20.145	ATP synthas	ATP synthase F(0) complex subunit C1, mitocl	K02128 F-
evm.model.HIC_ASM_10.161	PREDICTEI	Membrane-associated guanylate kinase, WW a	K06112 m

evm.model.HIC_ASM_19.110	PREDICTEI	Membrane-associated guanylate kinase, WW aı	K05631	atı
evm.model.HIC_ASM_11.248	membrane-as	Membrane-associated guanylate kinase, WW aı	K05629	atı
evm.model.HIC_ASM_11.133	unnamed pro	40S ribosomal protein S18 OS=Rattus norvegic	K02964	sm
evm.model.HIC_ASM_13.818	PREDICTEI	40S ribosomal protein S18 OS=Ictalurus puncta	K02964	sm
evm.model.HIC_ASM_6.486.1	40S riboson	40S ribosomal protein S18 OS=Rattus norvegic	K02964	sm
evm.model.HIC_ASM_8.1507	PREDICTEI	Ubiquitin-like protein FUBI OS=Sus scrofa OX	K02983	sm
evm.model.HIC_ASM_8.1523	ubiquitin-like	Putative ubiquitin-like protein FUBI-like protei	K02983	sm
evm.model.HIC_ASM_19.622	FAU [Cricet	Ubiquitin-like protein FUBI OS=Rattus norveg	K02983	sm
evm.model.CTG_122.1	--	UBX domain-containing protein 6 OS=Homo s	K14011	UI
evm.model.HIC_ASM_1.191	--	UBX domain-containing protein 6 OS=Homo s	K14011	UI
evm.model.HIC_ASM_16.267	PREDICTEI	Protocadherin-9 OS=Homo sapiens OX=9606 (	K16498	pr
evm.model.HIC_ASM_0.1417	LOW QUAL	Protocadherin-9 OS=Homo sapiens OX=9606 (	K16498	pr
evm.model.HIC_ASM_18.163	PREDICTEI	Zinc finger protein 543 OS=Homo sapiens OX:	K09228	KI
evm.model.HIC_ASM_18.164	PREDICTEI	Zinc finger protein 543 OS=Homo sapiens OX:	K09228	KI
evm.model.HIC_ASM_9.848	PREDICTEI	Mitochondrial import receptor subunit TOM20	K17770	mi
evm.model.CTG_738.2	mitochondria	Mitochondrial import receptor subunit TOM20	K17770	mi
evm.model.HIC_ASM_14.124	mitochondria	Mitochondrial import receptor subunit TOM20	K17770	mi
evm.model.HIC_ASM_12.438	proteolipid p	Proteolipid protein 2 OS=Homo sapiens OX=9	--	
evm.model.HIC_ASM_3.1015	PREDICTEI	Proteolipid protein 2 OS=Homo sapiens OX=9	--	
evm.model.HIC_ASM_7.454	proteolipid p	Proteolipid protein 2 OS=Homo sapiens OX=9	--	
evm.model.CTG_830.6	PREDICTEI	Bromodomain-containing protein 9 OS=Homo	K22184	br
evm.model.CTG_1655.1	bromodomai	Bromodomain-containing protein 9 OS=Homo	K22184	br
evm.model.CTG_816.1	bromodomai	Bromodomain-containing protein 9 OS=Homo	K22184	br
evm.model.CTG_168.1	PREDICTEI	Bromodomain-containing protein 9 OS=Homo	K22184	br
evm.model.CTG_1025.1	bromodomai	Bromodomain-containing protein 9 OS=Homo	K22184	br
evm.model.CTG_1043.6	PREDICTEI	Bromodomain-containing protein 9 OS=Homo	K22184	br
evm.model.HIC_ASM_21.319	LOW QUAL	Tyrosine-protein kinase HCK OS=Mus muscul	K08893	he
evm.model.HIC_ASM_3.729	tyrosine-prot	Tyrosine-protein kinase Fer OS=Canis lupus fa	K08889	tyı
evm.model.HIC_ASM_18.266	V-set and im	V-set and immunoglobulin domain-containing	--	
evm.model.HIC_ASM_18.259	V-set and im	V-set and immunoglobulin domain-containing	--	
evm.model.HIC_ASM_12.325	40s ribosoma	40S ribosomal protein S23 OS=Rattus norvegic	K02973	sm
evm.model.HIC_ASM_10.647	PREDICTEI	40S ribosomal protein S23 OS=Rattus norvegic	K02973	sm
evm.model.HIC_ASM_2.801	hypothetical	40S ribosomal protein S23 OS=Rattus norvegic	K02973	sm
evm.model.HIC_ASM_18.150	embryonic pc	Embryonic polyadenylate-binding protein 2 OS	K14396	po
evm.model.HIC_ASM_18.150	embryonic pc	Embryonic polyadenylate-binding protein 2 OS	K14396	po
evm.model.HIC_ASM_18.4	polyadenylat	Polyadenylate-binding protein 2 OS=Bos tauru	K14396	po
evm.model.HIC_ASM_4.984	polyadenylat	Polyadenylate-binding protein 2 OS=Homo sap	K14396	po
evm.model.CTG_1743.1	polyadenylat	Polyadenylate-binding protein 2 OS=Bos tauru	K14396	po
evm.model.HIC_ASM_7.421	zinc finger pı	Zinc finger protein 630 OS=Homo sapiens OX:	K09228	KI
evm.model.HIC_ASM_3.457_	zinc finger pı	Zinc finger protein 300 OS=Homo sapiens OX:	K09228	KI
evm.model.HIC_ASM_0.683	calmodulin, p	Calmodulin OS=Pyuridae sp. OX=7734 PE=1 S	K02183	ca
evm.model.HIC_ASM_7.213	PREDICTEI	Calmodulin OS=Electrophorus electricus OX=8	K02183	ca
evm.model.HIC_ASM_7.684	calmodulin-li	Calmodulin OS=Electrophorus electricus OX=8	K02183	ca
evm.model.HIC_ASM_4.432.2	PREDICTEI	Calmodulin OS=Stichopus japonicus OX=3079	K02183	ca
evm.model.HIC_ASM_0.515	Calmodulin	Calmodulin OS=Pythium splendens OX=82926	K02183	ca
evm.model.HIC_ASM_10.58	unnamed pro	Calmodulin-like protein 6 OS=Homo sapiens C	K02183	ca
evm.model.HIC_ASM_5.1830	calmodulin-li	Calmodulin-like protein 3 OS=Homo sapiens C	K02183	ca
evm.model.HIC_ASM_18.468	calmodulin-li	Calmodulin-2 B OS=Xenopus laevis OX=8355	K02183	ca

evm.model.HIC_ASM_12.466	Hypothetical	Calmodulin OS=Neurospora crassa (strain ATC	K02183 ca
evm.model.HIC_ASM_15.157	hypothetical	Ribosome biogenesis protein NSA2 homolog C	K14842 rib
evm.model.HIC_ASM_1.803	hypothetical	Ribosome biogenesis protein NSA2 homolog C	K14842 rib
evm.model.HIC_ASM_3.911	ribosome bio	Ribosome biogenesis protein NSA2 homolog C	K14842 rib
evm.model.HIC_ASM_15.156	ribosome bio	Ribosome biogenesis protein NSA2 homolog C	K14842 rib
evm.model.HIC_ASM_4.132	PREDICTEI	Rho-related GTP-binding protein RhoJ OS=Ho	K07864 Ra
evm.model.HIC_ASM_13.868	hypothetical	Rho-related GTP-binding protein RhoQ OS=Ra	K07194 Ra
evm.model.CTG_1310.1	cohesin subu	Cohesin subunit SA-2 OS=Mus musculus OX=	K06671 co
evm.model.CTG_667.7	cohesin subu	Cohesin subunit SA-1 OS=Homo sapiens OX=!	K06671 co
evm.model.CTG_735.12	cohesin subu	Cohesin subunit SA-2 OS=Mus musculus OX=	K06671 co
evm.model.CTG_1184.1	cohesin subu	Cohesin subunit SA-2 OS=Homo sapiens OX=!	K06671 co
evm.model.CTG_1490.5	cohesin subu	Cohesin subunit SA-1 OS=Xenopus laevis OX=	K06671 co
evm.model.HIC_ASM_7.1062	PREDICTEI	Retrotransposon Gag-like protein 8 OS=Bos ta	--
evm.model.HIC_ASM_7.1122	protein LDO	Protein LDOC1 OS=Gorilla gorilla gorilla OX=	--
evm.model.HIC_ASM_5.169	PREDICTEI	Retrotransposon Gag-like protein 6 OS=Homo	K20780 me
evm.model.HIC_ASM_7.1054	PREDICTEI	Retrotransposon Gag-like protein 8 OS=Bos ta	--
evm.model.HIC_ASM_7.1064	uncharacteriz	Retrotransposon Gag-like protein 8 OS=Bos ta	--
evm.model.HIC_ASM_7.1055	PREDICTEI	Retrotransposon Gag-like protein 8 OS=Bos ta	--
evm.model.HIC_ASM_4.822	PREDICTEI	Tubulin alpha-1 chain (Fragment) OS=Gallus g	K07374 tul
evm.model.HIC_ASM_7.413	PREDICTEI	Protein UXT OS=Homo sapiens OX=9606 GN	K07374 tul
evm.model.HIC_ASM_0.1318	tubulin alpha	Tubulin alpha-1 chain OS=Homarus americanu	K07374 tul
evm.model.HIC_ASM_3.1420	PREDICTEI	Tubulin alpha-1 chain OS=Oryza sativa subsp.	K07374 tul
evm.model.HIC_ASM_12.118	PREDICTEI	Transmembrane protein 132B OS=Homo sapie	K17599 tra
evm.model.HIC_ASM_12.121	PREDICTEI	Transmembrane protein 132C OS=Homo sapie	K17599 tra
evm.model.HIC_ASM_12.123	transmembra	Transmembrane protein 132D OS=Homo sapie	K17599 tra
evm.model.HIC_ASM_3.577	PREDICTEI	Dedicator of cytokinesis protein 2 OS=Homo s	K12367 de
evm.model.HIC_ASM_3.576	dedicator of	Dedicator of cytokinesis protein 2 OS=Homo s	K12367 de
evm.model.HIC_ASM_14.268	PREDICTEI	Dedicator of cytokinesis protein 1 OS=Mus mu	K13708 de
evm.model.HIC_ASM_3.713	band 4.1-like	Band 4.1-like protein 4A OS=Mus musculus O	K18037 tyi
evm.model.HIC_ASM_19.112	PREDICTEI	FERM domain-containing protein 4B OS=Hom	K18025 tyi
evm.model.CTG_62.1	solute carrier	Solute carrier family 41 member 3 OS=Rattus r	K15122 so
evm.model.CTG_978.1	solute carrier	Solute carrier family 41 member 3 OS=Rattus r	K15122 so
evm.model.CTG_412.1	PREDICTEI	(E2-independent) E3 ubiquitin-conjugating enz	K16773 fra
evm.model.HIC_ASM_13.663	alstrom synd	Alstrom syndrome protein 1 OS=Homo sapiens	K16741 als
evm.model.HIC_ASM_9.171	PREDICTEI	Protein PRRC2B OS=Homo sapiens OX=9606	--
evm.model.HIC_ASM_17.456	protein PRR	Protein PRRC2C OS=Homo sapiens OX=9606	--
evm.model.HIC_ASM_1.1089	roundabout h	Roundabout homolog 1 OS=Mus musculus OX	K06753 ro
evm.model.HIC_ASM_8.431	contactin-5 [	Contactin-5 OS=Homo sapiens OX=9606 GN=	K06763 co
evm.model.HIC_ASM_12.239	netrin recept	Netrin receptor DCC OS=Homo sapiens OX=9	K06765 de
evm.model.HIC_ASM_1.1093	roundabout h	Roundabout homolog 2 OS=Homo sapiens OX	K06754 ro
evm.model.HIC_ASM_2.1244	zymogen gra	Zymogen granule protein 16 homolog B OS=H	--
evm.model.HIC_ASM_2.1689	unnamed pro	Zymogen granule membrane protein 16 OS=Mu	--
evm.model.HIC_ASM_2.1692	PREDICTEI	Zymogen granule membrane protein 16 OS=Ra	--
evm.model.HIC_ASM_2.1691	PREDICTEI	Zymogen granule membrane protein 16 OS=Ra	--
evm.model.HIC_ASM_2.1693	zymogen gra	Zymogen granule membrane protein 16 OS=Hc	--
evm.model.HIC_ASM_2.1694	unnamed pro	Zymogen granule membrane protein 16 OS=Ra	--
evm.model.HIC_ASM_20.122	--	--	--
evm.model.CTG_643.1_evm.n	mediator of I	Coiled-coil domain-containing protein 200 OS=	--

evm.model.HIC_ASM_18.25	zinc finger pr	Zinc finger protein 836 OS=Homo sapiens OX=	K09228	KI
evm.model.HIC_ASM_18.26	PREDICTED	Zinc finger protein 677 OS=Homo sapiens OX=	K09228	KI
evm.model.HIC_ASM_2.649.4	PREDICTED	Heterogeneous nuclear ribonucleoprotein A3 O	K12741	he
evm.model.HIC_ASM_5.1197	Heterogeneou	Heterogeneous nuclear ribonucleoprotein A1 O	K12741	he
evm.model.HIC_ASM_11.615	heterogeneou	Heterogeneous nuclear ribonucleoprotein A1 O	K12741	he
evm.model.HIC_ASM_4.208	PREDICTED	Heterogeneous nuclear ribonucleoprotein A1 O	K12741	he
evm.model.HIC_ASM_11.443	heterogeneou	Heterogeneous nuclear ribonucleoprotein A1 O	K12741	he
evm.model.HIC_ASM_7.577	PREDICTED	Heterogeneous nuclear ribonucleoprotein A1 O	K12741	he
evm.model.HIC_ASM_4.838	heterogeneou	Heterogeneous nuclear ribonucleoprotein A1 O	K12741	he
evm.model.HIC_ASM_11.661	PREDICTED	Heterogeneous nuclear ribonucleoprotein A1 O	K12741	he
evm.model.HIC_ASM_5.759	LOW QUAL	Heterogeneous nuclear ribonucleoprotein A1 O	K12741	he
evm.model.HIC_ASM_5.1485	PREDICTED	Heterogeneous nuclear ribonucleoprotein A1 O	K12741	he
evm.model.HIC_ASM_6.1412	Heterogeneou	Heterogeneous nuclear ribonucleoprotein A3 O	K12741	he
evm.model.HIC_ASM_19.113	LOW QUAL	Heterogeneous nuclear ribonucleoprotein A1 O	K12741	he
evm.model.HIC_ASM_16.147	unnamed pro	Heterogeneous nuclear ribonucleoprotein A1-li	K12741	he
evm.model.HIC_ASM_8.915	PREDICTED	Heterogeneous nuclear ribonucleoprotein A1 O	K12741	he
evm.model.HIC_ASM_1.1087	protein TFG	Heterogeneous nuclear ribonucleoprotein A1 O	K12741	he
evm.model.HIC_ASM_3.909	Putative hete	Heterogeneous nuclear ribonucleoprotein A1 O	K12741	he
evm.model.HIC_ASM_7.982	heterogeneou	Heterogeneous nuclear ribonucleoprotein A1 O	K12741	he
evm.model.HIC_ASM_19.134	PREDICTED	Platelet glycoprotein IX OS=Homo sapiens OX	K06263	pla
evm.model.HIC_ASM_12.582	platelet glyco	Platelet glycoprotein Ib beta chain OS=Papio c	K06262	pla
evm.model.HIC_ASM_6.1106	signal transdu	Signal transducer CD24 OS=Homo sapiens OX	K06469	CI
evm.model.HIC_ASM_8.1089	signal transdu	Signal transducer CD24 OS=Mus musculus OX	K06469	CI
evm.model.HIC_ASM_19.119	glutamate rec	Metabotropic glutamate receptor 7 OS=Mus mu	K04608	me
evm.model.CTG_94.1	metabotropic	Metabotropic glutamate receptor 1 OS=Homo s	K04603	me
evm.model.HIC_ASM_5.755	40S ribosoma	40S ribosomal protein S15a OS=Pongo abelii (	K02957	sm
evm.model.HIC_ASM_4.1336	PREDICTED	40S ribosomal protein S15a OS=Rattus norveg	K02957	sm
evm.model.HIC_ASM_8.1264	PREDICTED	40S ribosomal protein S15a OS=Rattus norveg	K02957	sm
evm.model.HIC_ASM_14.622	PREDICTED	40S ribosomal protein S15a OS=Rattus norveg	K02957	sm
evm.model.HIC_ASM_0.25	PREDICTED	40S ribosomal protein S15a OS=Pongo abelii (	K02957	sm
evm.model.HIC_ASM_10.111	PREDICTED	40S ribosomal protein S15a OS=Rattus norveg	K02957	sm
evm.model.HIC_ASM_15.73	PREDICTED	40S ribosomal protein S15a OS=Pongo abelii (	K02957	sm
evm.model.HIC_ASM_2.1504	40S ribosoma	40S ribosomal protein S15a OS=Rattus norveg	K02957	sm
evm.model.HIC_ASM_3.489	ribosomal pro	40S ribosomal protein S15a OS=Rattus norveg	K02957	sm
evm.model.HIC_ASM_7.525	40S ribosoma	40S ribosomal protein S15a OS=Rattus norveg	K02957	sm
evm.model.HIC_ASM_16.265	40S ribosoma	40S ribosomal protein S15a OS=Rattus norveg	K02957	sm
evm.model.HIC_ASM_15.332	PREDICTED	60S ribosomal protein L30 OS=Ophiophagus h	K02908	lar
evm.model.HIC_ASM_18.907	PREDICTED	60S ribosomal protein L30 OS=Rattus norvegic	K02908	lar
evm.model.HIC_ASM_1.1061	60S ribosoma	60S ribosomal protein L30 OS=Ictalurus puncta	K02908	lar
evm.model.HIC_ASM_9.813	60S ribosoma	60S ribosomal protein L30 OS=Ophiophagus h	K02908	lar
evm.model.HIC_ASM_14.111	60s ribosoma	60S ribosomal protein L30 OS=Rattus norvegic	K02908	lar
evm.model.HIC_ASM_11.291	unnamed pro	60S ribosomal protein L30 OS=Ophiophagus h	K02908	lar
evm.model.HIC_ASM_12.244	60S ribosoma	60S ribosomal protein L30 OS=Ophiophagus h	K02908	lar
evm.model.HIC_ASM_6.555	60S ribosoma	60S ribosomal protein L30 OS=Ophiophagus h	K02908	lar
evm.model.HIC_ASM_1.785.5	nuclear cap-b	Nuclear cap-binding protein subunit 2 OS=Hom	K12883	nu
evm.model.HIC_ASM_0.967	nuclear cap b	Nuclear cap-binding protein subunit 2 OS=Hom	K12883	nu
evm.model.HIC_ASM_7.832	nuclear cap-b	Nuclear cap-binding protein subunit 2 OS=Ratt	K12883	nu
evm.model.HIC_ASM_11.137	LOW QUAL	40S ribosomal protein S6 OS=Bos taurus OX=9	K02991	sm

evm.model.HIC_ASM_5.1184	hypothetical	40S ribosomal protein S6 OS=Rattus norvegicu	K02991	sm
evm.model.HIC_ASM_11.930	PREDICTED	40S ribosomal protein S6 OS=Rattus norvegicu	K02991	sm
evm.model.HIC_ASM_1.1055	40S ribosoma	40S ribosomal protein S6 OS=Rattus norvegicu	K02991	sm
evm.model.HIC_ASM_4.129	40S ribosoma	40S ribosomal protein S6 OS=Rattus norvegicu	K02991	sm
evm.model.HIC_ASM_16.277	hypothetical	40S ribosomal protein S6 OS=Rattus norvegicu	K02991	sm
evm.model.HIC_ASM_9.790	PREDICTED	40S ribosomal protein S6 OS=Rattus norvegicu	K02991	sm
evm.model.HIC_ASM_7.382	PREDICTED	40S ribosomal protein S6 OS=Rattus norvegicu	K02991	sm
evm.model.HIC_ASM_3.1298	fibrillin-2 [F	Fibrillin-2 OS=Homo sapiens OX=9606 GN=F	K23342	fib
evm.model.HIC_ASM_4.1289	fibrillin-1 iso	Fibrillin-1 OS=Sus scrofa OX=9823 GN=FBN	K06825	fib
evm.model.HIC_ASM_0.1234	PREDICTED	Ephrin type-A receptor 5 OS=Homo sapiens O	K05106	Ep
evm.model.HIC_ASM_1.1049	PREDICTED	Ephrin type-A receptor 6 OS=Homo sapiens O	K05107	Ep
evm.model.HIC_ASM_10.485	ephrin type-E	Ephrin type-B receptor 2 OS=Homo sapiens O	K05111	Ep
evm.model.HIC_ASM_1.316	hypothetical	Ephrin type-B receptor 1 OS=Homo sapiens O	K05110	Ep
evm.model.HIC_ASM_6.1036	ephrin type-A	Ephrin type-A receptor 7 OS=Homo sapiens O	K05108	Ep
evm.model.HIC_ASM_0.1235	ephrin type-A	Ephrin type-A receptor 5 OS=Homo sapiens O	K05106	Ep
evm.model.HIC_ASM_7.724	achain crysta	Profilin-1 OS=Bos taurus OX=9913 GN=PFN1	K05759	pro
evm.model.HIC_ASM_6.1069	profilin-1 [M	Profilin-1 OS=Bos taurus OX=9913 GN=PFN1	K05759	pro
evm.model.HIC_ASM_20.621	profilin-1 [S	Profilin-1 OS=Bos taurus OX=9913 GN=PFN1	K05759	pro
evm.model.HIC_ASM_0.1330	Chain A, Stru	Profilin-1 OS=Rattus norvegicus OX=10116 G	K05759	pro
evm.model.HIC_ASM_1.432	profilin 2, isc	Profilin-2 OS=Mus musculus OX=10090 GN=I	K05759	pro
evm.model.HIC_ASM_8.1121	profilin-1 [D	Profilin-1 OS=Rattus norvegicus OX=10116 G	K05759	pro
evm.model.CTG_786.3	PREDICTED	Ral guanine nucleotide dissociation stimulator	K08732	ral
evm.model.CTG_608.6	ral guanine n	Ral guanine nucleotide dissociation stimulator	K08732	ral
evm.model.CTG_225.7	PREDICTED	Ral guanine nucleotide dissociation stimulator	K08732	ral
evm.model.CTG_1110.1	PREDICTED	Ral guanine nucleotide dissociation stimulator	K08732	ral
evm.model.HIC_ASM_4.1030	T-cell recepto	T cell receptor alpha variable 22 OS=Homo sa	K10784	T
evm.model.HIC_ASM_4.1029	hCG1812138	T cell receptor alpha variable 29/delta variable	K10784	T
evm.model.HIC_ASM_4.1044	T-cell recepto	T cell receptor alpha variable 6 OS=Homo sapi	K10784	T
evm.model.HIC_ASM_4.1036	hCG2014123	T cell receptor alpha variable 17 OS=Homo sa	K10784	T
evm.model.HIC_ASM_4.1033	hypothetical	T cell receptor alpha variable 25 OS=Homo sa	K10784	T
evm.model.HIC_ASM_4.1041	Hypothetical	T cell receptor alpha variable 22 OS=Homo sa	K10784	T
evm.model.HIC_ASM_4.1039	Hypothetical	T cell receptor alpha variable 10 OS=Homo sa	K10784	T
evm.model.HIC_ASM_4.1031	Hypothetical	T cell receptor alpha variable 27 OS=Homo sa	K10784	T
evm.model.HIC_ASM_4.1027	T cell recepto	T cell receptor alpha variable 34 OS=Homo sa	K10784	T
evm.model.HIC_ASM_4.1026	hypothetical	T cell receptor alpha variable 35 OS=Homo sa	K10784	T
evm.model.HIC_ASM_4.1043	Hypothetical	T cell receptor alpha variable 22 OS=Homo sa	K10784	T
evm.model.HIC_ASM_4.1038	Hypothetical	T cell receptor alpha variable 12-3 OS=Homo s	K10784	T
evm.model.HIC_ASM_20.131	SWI/SNF-rel	SWI/SNF-related matrix-associated actin-deper	K11651	SV
evm.model.HIC_ASM_7.553	SWI/SNF-rel	SWI/SNF-related matrix-associated actin-deper	K11651	SV
evm.model.HIC_ASM_7.872	PREDICTED	SWI/SNF-related matrix-associated actin-deper	K11651	SV
evm.model.CTG_1362.2	PREDICTED	Polycystic kidney disease protein 1-like 1 OS=I	K04987	po
evm.model.HIC_ASM_11.129	PREDICTED	Polycystic kidney disease protein 1-like 1 OS=I	K04987	po
evm.model.CTG_225.4	LOW QUAL	Polycystic kidney disease protein 1-like 1 OS=I	K04987	po
evm.model.HIC_ASM_3.1480	obscurin isof	Obscurin OS=Mus musculus OX=10090 GN=O	K17531	ob
evm.model.HIC_ASM_3.1481	LOW QUAL	Obscurin OS=Homo sapiens OX=9606 GN=OI	K17531	ob
evm.model.HIC_ASM_2.878	mCG120681	60S ribosomal protein L28 OS=Mus musculus	K02903	lar
evm.model.HIC_ASM_7.364	60S ribosoma	60S ribosomal protein L28 OS=Bos taurus OX	K02903	lar
evm.model.HIC_ASM_18.102	60S ribosoma	60S ribosomal protein L28 OS=Homo sapiens	K02903	lar

evm.model.HIC_ASM_0.633	PREDICTEI	60S ribosomal protein L28 OS=Bos taurus OX	K02903	lar
evm.model.HIC_ASM_9.762	60s ribosoma	60S ribosomal protein L28 OS=Mus musculus	K02903	lar
evm.model.HIC_ASM_4.913	hypothetical	60S ribosomal protein L28 OS=Homo sapiens	K02903	lar
evm.model.HIC_ASM_4.1137	60S ribosoma	60S ribosomal protein L28 OS=Mus musculus	K02903	lar
evm.model.HIC_ASM_2.1876	huntingtin-in	Huntingtin-interacting protein 1 OS=Homo sap	K04559	hu
evm.model.HIC_ASM_9.655	unnamed pro	Talin-1 OS=Mus musculus OX=10090 GN=Tl	K06271	tal
evm.model.HIC_ASM_1.1106	hypothetical	60S ribosomal protein L9 OS=Macaca fascicul	K02940	lar
evm.model.HIC_ASM_0.1046	ribosomal pr	60S ribosomal protein L9 OS=Mus musculus C	K02940	lar
evm.model.HIC_ASM_12.303	LOW QUAL	60S ribosomal protein L9 OS=Macaca fascicul	K02940	lar
evm.model.HIC_ASM_10.121	TPA: ribosor	60S ribosomal protein L9 OS=Macaca fascicul	K02940	lar
evm.model.HIC_ASM_1.1105	60S ribosoma	60S ribosomal protein L9 OS=Mus musculus C	K02940	lar
evm.model.HIC_ASM_8.507	PREDICTEI	60S ribosomal protein L9 OS=Homo sapiens O	K02940	lar
evm.model.HIC_ASM_0.1378	PREDICTEI	60S ribosomal protein L9 OS=Homo sapiens O	K02940	lar
evm.model.HIC_ASM_6.1222	60S ribosoma	60S ribosomal protein L9 OS=Macaca fascicul	K02940	lar
evm.model.HIC_ASM_12.119	60S ribosoma	60S ribosomal protein L9 OS=Macaca fascicul	K02940	lar
evm.model.HIC_ASM_5.1208	hypothetical	60S ribosomal protein L9 OS=Macaca fascicul	K02940	lar
evm.model.HIC_ASM_5.730	PREDICTEI	60S ribosomal protein L9 OS=Pongo abelii OX	K02940	lar
evm.model.HIC_ASM_8.1390	PREDICTEI	Secretoglobin family 1D member 1 OS=Homo	--	
evm.model.HIC_ASM_8.1388	secretoglobir	Secretoglobin family 1D member 2 OS=Homo	--	
evm.model.HIC_ASM_8.1387	secretoglobir	Secretoglobin family 1D member OS=Bos taur	--	
evm.model.HIC_ASM_3.1513	PREDICTEI	Butyrophilin subfamily 1 member A1 OS=Hon	K06712	bu
evm.model.HIC_ASM_3.1510	PREDICTEI	Butyrophilin subfamily 1 member A1 OS=Hon	K06712	bu
evm.model.HIC_ASM_3.1507	PREDICTEI	Butyrophilin subfamily 1 member A1 OS=Bos	K06712	bu
evm.model.HIC_ASM_20.143	cytochrome b	Cytochrome b5 OS=Homo sapiens OX=9606 C	K17418	lar
evm.model.HIC_ASM_12.96.1	cytochrome b	Cytochrome b5 OS=Sus scrofa OX=9823 GN=	K17418	lar
evm.model.HIC_ASM_0.940	PREDICTEI	Fatty acid desaturase 2-like protein FADS2P1 (	K10226	ac
evm.model.CTG_207.1	solute carrier	Solute carrier organic anion transporter family	K14353	so
evm.model.HIC_ASM_1.303	PREDICTEI	Solute carrier organic anion transporter family	K14345	so
evm.model.HIC_ASM_20.563	PREDICTEI	Protein phosphatase inhibitor 2 OS=Rattus nor	K16833	pr
evm.model.HIC_ASM_7.359	putative type	Protein phosphatase inhibitor 2 OS=Oryctolagu	K16833	pr
evm.model.HIC_ASM_1.776	protein phosp	Protein phosphatase inhibitor 2 OS=Oryctolagu	K16833	pr
evm.model.CTG_335.31	cathepsin D	Cathepsin D OS=Canis lupus familiaris OX=96	K01379	ca
evm.model.HIC_ASM_8.1725	Cathepsin D	Cathepsin D OS=Canis lupus familiaris OX=96	K01379	ca
evm.model.HIC_ASM_18.315	PREDICTEI	Napsin-A OS=Homo sapiens OX=9606 GN=N	K08565	na
evm.model.HIC_ASM_3.257	PREDICTEI	A disintegrin and metalloproteinase with throm	K08626	a c
evm.model.HIC_ASM_3.1398	PREDICTEI	A disintegrin and metalloproteinase with throm	K08618	a c
evm.model.HIC_ASM_19.312	PREDICTEI	A disintegrin and metalloproteinase with throm	K08625	a c
evm.model.HIC_ASM_4.852	PREDICTEI	60S ribosomal protein L13 OS=Mus musculus	K02873	lar
evm.model.HIC_ASM_18.153	PREDICTEI	60S ribosomal protein L13 OS=Homo sapiens	K02873	lar
evm.model.HIC_ASM_7.1016	LOW QUAL	60S ribosomal protein L13 OS=Mus musculus	K02873	lar
evm.model.HIC_ASM_6.887	ribosomal pr	60S ribosomal protein L13 OS=Homo sapiens	K02873	lar
evm.model.HIC_ASM_3.1512	erythroid me	Erythroid membrane-associated protein OS=M	K06712	bu
evm.model.HIC_ASM_3.1511	PREDICTEI	Erythroid membrane-associated protein OS=M	K06712	bu
evm.model.CTG_764.1	PREDICTEI	Calcineurin B homologous protein 3 OS=Hom	K17612	ca
evm.model.HIC_ASM_15.206	PREDICTEI	Calcineurin B homologous protein 3 OS=Hom	K17612	ca
evm.model.HIC_ASM_14.158	homeobox pr	Homeobox protein MSX-3 OS=Mus musculus	K09341	ho
evm.model.HIC_ASM_3.640	PREDICTEI	Homeobox protein MSX-2 OS=Bos taurus OX	K09341	ho
evm.model.HIC_ASM_0.259	teneurin-3 [I	Teneurin-3 OS=Homo sapiens OX=9606 GN=	--	

evm.model.HIC_ASM_3.564	teneurin-2 isc	Teneurin-2 OS=Homo sapiens OX=9606 GN='	--
evm.model.HIC_ASM_8.552	teneurin-4 isc	Teneurin-4 OS=Mus musculus OX=10090 GN:	--
evm.model.HIC_ASM_3.563	PREDICTEI	Teneurin-2 OS=Homo sapiens OX=9606 GN='	--
evm.model.HIC_ASM_18.21	protein FRG2	Protein FRG2 OS=Homo sapiens OX=9606 GI	K09228 KI
evm.model.HIC_ASM_18.14	protein FRG2	Protein FRG2 OS=Homo sapiens OX=9606 GI	--
evm.model.HIC_ASM_20.88	LOW QUAL	60S ribosomal protein L13a OS=Pongo abelii (	K02872 lar
evm.model.HIC_ASM_0.813	ribosomal pr	60S ribosomal protein L13a OS=Canis lupus fa	K02872 lar
evm.model.HIC_ASM_19.417	non-receptor	Non-receptor tyrosine-protein kinase TYK2 OS	K11219 no
evm.model.HIC_ASM_10.121	tyrosine-prot	Tyrosine-protein kinase JAK1 OS=Homo sapie	K11217 Ja
evm.model.HIC_ASM_7.896	Hypothetical	Testis-expressed protein 13B OS=Homo sapien	--
evm.model.HIC_ASM_7.812	PREDICTEI	Testis-expressed protein 13A OS=Homo sapien	--
evm.model.HIC_ASM_7.813	hypothetical	Testis-expressed protein 13B OS=Homo sapien	--
evm.model.CTG_786.4	--	--	--
evm.model.CTG_1634.2	--	--	--
evm.model.HIC_ASM_6.251	LOW QUAL	Putative vomeronasal receptor-like protein 4 O	K04614 vo
evm.model.HIC_ASM_6.252	LOW QUAL	Vomeronasal type-1 receptor 3 OS=Homo sapi	K04614 vo
evm.model.HIC_ASM_0.287	putative prot	Putative protein FAM90A23P OS=Homo sapie	K09228 KI
evm.model.CTG_457.1	putative prot	Putative protein FAM90A23P OS=Homo sapie	K09228 KI
evm.model.CTG_271.1	putative prot	Putative protein FAM90A12P OS=Homo sapie	K09228 KI
evm.model.CTG_457.3	putative prot	Putative protein FAM90A23P OS=Homo sapie	K09228 KI
evm.model.CTG_457.4	putative prot	Putative protein FAM90A23P OS=Homo sapie	K09228 KI
evm.model.CTG_457.7	putative prot	Putative protein FAM90A23P OS=Homo sapie	K09228 KI
evm.model.CTG_457.2	putative prot	Putative protein FAM90A23P OS=Homo sapie	K09228 KI
evm.model.CTG_457.5	putative prot	Putative protein FAM90A23P OS=Homo sapie	K09228 KI
evm.model.CTG_271.4	putative prot	Putative protein FAM90A12P OS=Homo sapie	K09228 KI
evm.model.CTG_457.6	putative prot	Protein FAM90A1 OS=Homo sapiens OX=96C	K09228 KI
evm.model.HIC_ASM_4.1035	PREDICTEI	T cell receptor alpha variable 19 OS=Homo saj	K10784 T
evm.model.HIC_ASM_4.1040	hypothetical	T cell receptor alpha variable 14/delta variable	K10784 T
evm.model.HIC_ASM_12.970	phosphatidyl	Phosphatidylethanolamine-binding protein 1 O	K17439 lar
evm.model.HIC_ASM_3.234	phosphatidyl	Phosphatidylethanolamine-binding protein 1 O	K17439 lar
evm.model.HIC_ASM_0.1191	PREDICTEI	Glycine cleavage system H protein, mitochondr	K02437 gly
evm.model.HIC_ASM_2.727	hypothetical	Glycine cleavage system H protein, mitochondr	K02437 gly
evm.model.HIC_ASM_18.132	PREDICTEI	Glycine cleavage system H protein, mitochondr	K02437 gly
evm.model.HIC_ASM_10.263	PREDICTEI	Serine/threonine-protein kinase mTOR OS=Ho	K07203 ser
evm.model.HIC_ASM_8.20	serine-protei	Serine-protein kinase ATM OS=Homo sapiens	K04728 ser
evm.model.HIC_ASM_7.281	protein FAM	Protein FAM209A OS=Homo sapiens OX=96C	--
evm.model.HIC_ASM_21.751	PREDICTEI	Protein FAM209A OS=Homo sapiens OX=96C	--
evm.model.HIC_ASM_7.287	protein FAM	Protein FAM209A OS=Homo sapiens OX=96C	--
evm.model.HIC_ASM_7.289	protein FAM	Protein FAM209A OS=Homo sapiens OX=96C	--
evm.model.HIC_ASM_11.331	PREDICTEI	Collagen alpha-2(I) chain OS=Canis lupus fami	K06236 co
evm.model.HIC_ASM_6.478	collagen alph	Collagen alpha-2(XI) chain OS=Homo sapiens	K19721 co
evm.model.HIC_ASM_10.133	collagen alph	Collagen alpha-1(XXIV) chain OS=Homo sapi	K19721 co
evm.model.HIC_ASM_2.591	collagen alph	Collagen alpha-1(III) chain OS=Homo sapiens	K19720 co
evm.model.HIC_ASM_1.468	protein tyrosi	Protein tyrosine phosphatase type IVA 1 OS=R	K18041 pro
evm.model.HIC_ASM_6.868	Chain B, Cry	Protein tyrosine phosphatase type IVA 1 OS=R	K18041 pro
evm.model.HIC_ASM_8.518	protein tyrosi	Protein tyrosine phosphatase type IVA 1 OS=P	K18041 pro
evm.model.HIC_ASM_3.740	60S ribosoma	60S ribosomal protein L27 OS=Rattus norvegic	K02901 lar
evm.model.HIC_ASM_20.122	60S ribosoma	60S ribosomal protein L27 OS=Rattus norvegic	K02901 lar

evm.model.HIC_ASM_10.121	unnamed pro	60S ribosomal protein L27 OS=Rattus norvegic	K02901 lar
evm.model.HIC_ASM_14.11	PREDICTEI	60S ribosomal protein L27 OS=Canis lupus fan	K02901 lar
evm.model.HIC_ASM_5.1377	hCG2040271	60S ribosomal protein L27 OS=Canis lupus fan	K02901 lar
evm.model.HIC_ASM_13.433	60S ribosoma	60S ribosomal protein L27 OS=Rattus norvegic	K02901 lar
evm.model.CTG_1115.4	--	--	K13073 po
evm.model.HIC_ASM_0.1193	--	--	K13073 po
evm.model.HIC_ASM_17.517	complement	Complement factor H OS=Homo sapiens OX=9	K04004 co
evm.model.HIC_ASM_17.519	complement	Complement factor H OS=Bos taurus OX=991	K04004 co
evm.model.CTG_1199.2	ankyrin repea	Ankyrin repeat domain-containing protein 26 C	K17299 PC
evm.model.CTG_1199.5	ankyrin repea	Ankyrin repeat domain-containing protein 26 C	K17299 PC
evm.model.HIC_ASM_5.1633	PREDICTEI	Ankyrin repeat domain-containing protein 26 C	K17299 PC
evm.model.HIC_ASM_5.1635	PREDICTEI	Ankyrin repeat domain-containing protein 26 C	K17299 PC
evm.model.CTG_1319.1	ankyrin repea	Ankyrin repeat domain-containing protein 26 C	K17299 PC
evm.model.HIC_ASM_20.389	centromere p	Centromere protein V OS=Homo sapiens OX=9	--
evm.model.HIC_ASM_7.484	centromere p	Centromere protein V-like protein 3 OS=Homo	--
evm.model.HIC_ASM_7.483	centromere p	Centromere protein V-like protein 3 OS=Homo	--
evm.model.HIC_ASM_15.108	60S acidic ril	60S acidic ribosomal protein P1 OS=Sus scrofa	K02942 lar
evm.model.HIC_ASM_6.1254	PREDICTEI	60S acidic ribosomal protein P1 OS=Homo sap	K02942 lar
evm.model.HIC_ASM_4.1519	60S acidic ril	60S acidic ribosomal protein P1 OS=Homo sap	K02942 lar
evm.model.HIC_ASM_4.917	60s acidic rit	60S acidic ribosomal protein P1 OS=Sus scrofa	K02942 lar
evm.model.HIC_ASM_8.523	60S acidic ril	60S acidic ribosomal protein P1 OS=Homo sap	K02942 lar
evm.model.HIC_ASM_9.840	60S acidic ril	60S acidic ribosomal protein P1 OS=Rattus noi	K02942 lar
evm.model.HIC_ASM_9.819	hypothetical	60S acidic ribosomal protein P1 OS=Rattus noi	K02942 lar
evm.model.HIC_ASM_10.186	60s acidic rit	60S acidic ribosomal protein P1 OS=Homo sap	K02942 lar
evm.model.CTG_1723.1	Hypothetical	Protein FAM71C OS=Homo sapiens OX=9606	--
evm.model.CTG_1541.1	protein FAM	Protein FAM71A OS=Macaca fascicularis OX=	--
evm.model.HIC_ASM_5.538	PREDICTEI	Protein FAM71C OS=Macaca fascicularis OX=	K10772 AI
evm.model.HIC_ASM_3.514	protein FAM	Protein FAM71B OS=Homo sapiens OX=9606	K10772 AI
evm.model.HIC_ASM_17.259	protein FAM	Protein FAM71A OS=Macaca fascicularis OX=	K10772 AI
evm.model.CTG_477.1	PREDICTEI	Protein FAM71A OS=Homo sapiens OX=9606	--
evm.model.CTG_901.2	protein FAM	Protein FAM71A OS=Macaca fascicularis OX=	--
evm.model.CTG_225.8	PREDICTEI	Ral guanine nucleotide dissociation stimulator (	K08732 ral
evm.model.CTG_786.2	ral guanine n	--	K08732 ral
evm.model.CTG_297.2	ral guanine n	Ral guanine nucleotide dissociation stimulator (	K08732 ral
evm.model.CTG_230.2	ral guanine n	Ral guanine nucleotide dissociation stimulator (	K08732 ral
evm.model.CTG_377.2	PREDICTEI	Ral guanine nucleotide dissociation stimulator (	K08732 ral
evm.model.CTG_1651.1	ral guanine n	Ral guanine nucleotide dissociation stimulator (	K08732 ral
evm.model.CTG_608.7	PREDICTEI	--	K08732 ral
evm.model.HIC_ASM_11.127	ral guanine n	Ral guanine nucleotide dissociation stimulator (	K08732 ral
evm.model.CTG_1634.1	ral guanine n	Ral guanine nucleotide dissociation stimulator (	K08732 ral
evm.model.CTG_495.2	ral guanine n	Ral guanine nucleotide dissociation stimulator (	K08732 ral
evm.model.CTG_1720.2	ral guanine n	Ral guanine nucleotide dissociation stimulator (	K08732 ral
evm.model.CTG_1110.2	PREDICTEI	--	K08732 ral
evm.model.HIC_ASM_16.295	Protocadheri	Protocadherin-8 OS=Homo sapiens OX=9606 (	K16499 pr
evm.model.HIC_ASM_0.787	PREDICTEI	Protocadherin-18 OS=Bos taurus OX=9913 GI	K16499 pr
evm.model.HIC_ASM_20.159	PREDICTEI	Dynein light chain 2, cytoplasmic OS=Rattus n	K10418 dy
evm.model.HIC_ASM_1.594	PREDICTEI	Dynein light chain 1, cytoplasmic OS=Rattus n	K10418 dy
evm.model.HIC_ASM_1.526	Hypothetical	--	K11426 SI

evm.model.HIC_ASM_5.757	Hypothetical	--	--
evm.model.HIC_ASM_18.107	hypothetical	--	--
evm.model.HIC_ASM_0.92	Hypothetical	--	--
evm.model.HIC_ASM_20.448	putative trans	Transmembrane protein 238 OS=Mus musculus	K15216 RN
evm.model.HIC_ASM_18.101	PREDICTED	Transmembrane protein 238 OS=Mus musculus	K15216 RN
evm.model.HIC_ASM_11.360	PREDICTED	S-phase kinase-associated protein 1 OS=Rattus	K03094 S-
evm.model.HIC_ASM_3.1240	S-phase kinas	S-phase kinase-associated protein 1 OS=Xenop	K03094 S-
evm.model.HIC_ASM_0.1051	SKP1 [Cricet	S-phase kinase-associated protein 1 OS=Xenop	K03094 S-
evm.model.HIC_ASM_11.642	hypothetical	S-phase kinase-associated protein 1 OS=Xenop	K03094 S-
evm.model.HIC_ASM_9.768	cyclin-depend	Cyclin-dependent kinase inhibitor 2A OS=Hom	K06621 cy
evm.model.HIC_ASM_9.767	cyclin-depend	Cyclin-dependent kinase 4 inhibitor B OS=Hom	K04685 cy
evm.model.HIC_ASM_9.1045	PREDICTED	Excitatory amino acid transporter 3 OS=Homo	K05612 so
evm.model.HIC_ASM_9.1038	PREDICTED	Excitatory amino acid transporter 3 OS=Homo	K05612 so
evm.model.HIC_ASM_3.1506	butyrophilin-	Erythroid membrane-associated protein OS=Ho	K06712 bu
evm.model.HIC_ASM_6.449	PREDICTED	Butyrophilin subfamily 1 member A1 OS=Bos	K06712 bu
evm.model.HIC_ASM_3.1515	butyrophilin-	Erythroid membrane-associated protein OS=Ho	K06712 bu
evm.model.HIC_ASM_6.448	butyrophilin	Butyrophilin subfamily 1 member A1 OS=Bos	K06712 bu
evm.model.HIC_ASM_18.128	ret finger 4a-	Ret finger protein-like 4A OS=Homo sapiens C	K12015 tri
evm.model.HIC_ASM_6.1170	PREDICTED	Ret finger protein-like 4B OS=Homo sapiens C	K12009 tri
evm.model.CTG_739.2	collagen alph	Collagen alpha-1(XXIII) chain OS=Homo sapi	--
evm.model.CTG_1155.9	collagen alph	Collagen alpha-1(XXIII) chain OS=Homo sapi	--
evm.model.CTG_24.2	ankyrin repea	Ankyrin repeat domain-containing protein 26 C	K17299 PC
evm.model.CTG_1227.1	PREDICTED	Ankyrin repeat domain-containing protein 26 C	K17299 PC
evm.model.CTG_138.3	ankyrin repea	Ankyrin repeat domain-containing protein 26 C	K17299 PC
evm.model.HIC_ASM_20.161	acyl carrier p	Acyl carrier protein, mitochondrial OS=Pan tro	K03955 NA
evm.model.HIC_ASM_2.1574	acyl carrier p	Acyl carrier protein, mitochondrial OS=Bos tau	K03955 NA
evm.model.HIC_ASM_7.929	acyl carrier p	Acyl carrier protein, mitochondrial OS=Bos tau	K03955 NA
evm.model.HIC_ASM_10.119	acyl carrier p	Acyl carrier protein, mitochondrial OS=Pongo	K03955 NA
evm.model.CTG_1562.4	PREDICTED	Long-chain-fatty-acid--CoA ligase 6 OS=Homo	K01897 lon
evm.model.CTG_1562.1	PREDICTED	Long-chain-fatty-acid--CoA ligase 6 OS=Homo	K01897 lon
evm.model.HIC_ASM_5.217	cytochrome p	Cytochrome P450 2D14 OS=Bos taurus OX=9	K07414 cy
evm.model.HIC_ASM_10.118	cytochrome P	Cytochrome P450 2J2 OS=Homo sapiens OX=	K07418 cy
evm.model.CTG_626.1	PREDICTED	Sushi, nidogen and EGF-like domain-containin	K16669 pr
evm.model.CTG_1610.1	PREDICTED	Sushi, nidogen and EGF-like domain-containin	K02599 No
evm.model.HIC_ASM_1.973	Non-histone	Non-histone chromosomal protein HMG-14 OS	K11299 hig
evm.model.HIC_ASM_1.1274	non-histone c	Non-histone chromosomal protein HMG-14 OS	K11299 hig
evm.model.HIC_ASM_16.127	PREDICTED	Non-histone chromosomal protein HMG-14 OS	K11299 hig
evm.model.HIC_ASM_16.438	PREDICTED	Transcription factor SPT20 homolog OS=Hom	K21245 tra
evm.model.HIC_ASM_7.250	PREDICTED	Transcription factor SPT20 homolog OS=Mus	K21245 tra
evm.model.HIC_ASM_7.224	PREDICTED	Transcription factor SPT20 homolog OS=Rattu	K21245 tra
evm.model.HIC_ASM_7.225	Hypothetical	Transcription factor SPT20 homolog OS=Mus	K21245 tra
evm.model.HIC_ASM_4.1089	PREDICTED	Olfactory receptor 4F3/4F16/4F29 OS=Homo s	K04257 olf
evm.model.HIC_ASM_4.1087	olfactory rec	Olfactory receptor 4K1 OS=Homo sapiens OX	K04257 olf
evm.model.HIC_ASM_17.81	etoposide-ind	Etoposide-induced protein 2.4 homolog OS=Bo	K10134 eto
evm.model.HIC_ASM_17.86	etoposide-ind	Etoposide-induced protein 2.4 homolog OS=Ho	K10134 eto
evm.model.HIC_ASM_8.419	hypothetical	Glutamate receptor 4 OS=Rattus norvegicus OX	K05200 glu
evm.model.HIC_ASM_7.954	glutamate rec	Glutamate receptor 3 OS=Macaca fascicularis (	K05199 glu
evm.model.HIC_ASM_12.483	PREDICTED	Lethal(3)malignant brain tumor-like protein 4 (	K11457 po

evm.model.HIC_ASM_21.512	PREDICTEI	Lethal(3)malignant brain tumor-like protein 1 (	K23192 my
evm.model.HIC_ASM_7.252	60S ribosoma	60S ribosomal protein L32 OS=Rattus norvegic	K02912 lar
evm.model.HIC_ASM_4.406	hypothetical	Putative 60S ribosomal protein L32' OS=Mus r	K02912 lar
evm.model.HIC_ASM_11.24	--	--	--
evm.model.CTG_283.1	--	--	--
evm.model.HIC_ASM_12.329	CUGBP Elav	CUGBP Elav-like family member 4 OS=Macac	K13207 CU
evm.model.HIC_ASM_5.1786	CUGBP Elav	CUGBP Elav-like family member 2 OS=Rattus	K13207 CU
evm.model.HIC_ASM_20.964	PREDICTEI	--	K20395 CC
evm.model.HIC_ASM_20.965	CMRF35-lik	--	K20395 CC
evm.model.HIC_ASM_17.760	T-cell surface	T-cell surface glycoprotein CD1c OS=Homo sa	K06448 T-
evm.model.HIC_ASM_17.770	T-cell surface	T-cell surface glycoprotein CD1b-1 OS=Ovis a	K06448 T-
evm.model.HIC_ASM_17.771	PREDICTEI	T-cell surface glycoprotein CD1b-1 OS=Ovis a	K06448 T-
evm.model.HIC_ASM_17.768	PREDICTEI	T-cell surface glycoprotein CD1a OS=Sus scro	K06448 T-
evm.model.HIC_ASM_17.763	T-cell surface	T-cell surface glycoprotein CD1b-1 OS=Ovis a	K06448 T-
evm.model.HIC_ASM_17.764	PREDICTEI	T-cell surface glycoprotein CD1b OS=Aotus na	K06448 T-
evm.model.HIC_ASM_17.758	PREDICTEI	T-cell surface glycoprotein CD1e, membrane-a	K06448 T-
evm.model.HIC_ASM_17.759	PREDICTEI	T-cell surface glycoprotein CD1b-1 OS=Ovis a	K06448 T-
evm.model.HIC_ASM_17.766	PREDICTEI	T-cell surface glycoprotein CD1b-1 OS=Ovis a	K06448 T-
evm.model.HIC_ASM_21.219	60S ribosoma	60S ribosomal protein L35a OS=Bos taurus OX	K02917 lar
evm.model.HIC_ASM_19.108	60S ribosoma	60S ribosomal protein L35a OS=Pongo abelii (	K02917 lar
evm.model.HIC_ASM_5.274	ribosomal pro	60S ribosomal protein L35a OS=Pongo abelii (	K02917 lar
evm.model.HIC_ASM_1.816	60s ribosoma	60S ribosomal protein L35a OS=Pongo abelii (	K02917 lar
evm.model.HIC_ASM_14.105	60S ribosoma	60S ribosomal protein L35a OS=Pongo abelii (	K02917 lar
evm.model.HIC_ASM_19.104	60s ribosoma	60S ribosomal protein L35a OS=Pongo abelii (	K02917 lar
evm.model.HIC_ASM_3.552	ribosomal pro	60S ribosomal protein L35a OS=Bos taurus OX	K02917 lar
evm.model.HIC_ASM_2.1612	proliferating	Proliferating cell nuclear antigen OS=Macaca f	K04802 pro
evm.model.HIC_ASM_1.435	Chain A, Pro	Proliferating cell nuclear antigen OS=Macaca f	K04802 pro
evm.model.HIC_ASM_13.592	PREDICTEI	Proliferating cell nuclear antigen OS=Rattus no	K04802 pro
evm.model.HIC_ASM_21.167	PCNA [Hon	Proliferating cell nuclear antigen OS=Bos tauru	K04802 pro
evm.model.HIC_ASM_21.168	proliferating	Proliferating cell nuclear antigen OS=Rattus no	K04802 pro
evm.model.HIC_ASM_6.535.1	PREDICTEI	U1 small nuclear ribonucleoprotein C OS=Dan	K11095 U1
evm.model.HIC_ASM_4.988	U1 small nuc	U1 small nuclear ribonucleoprotein C OS=Dan	K11095 U1
evm.model.HIC_ASM_12.455	PREDICTEI	U1 small nuclear ribonucleoprotein C OS=Mor	K11095 U1
evm.model.HIC_ASM_9.1155	NUT family	NUT family member 2D OS=Homo sapiens OX	--
evm.model.HIC_ASM_4.1094	NUT family	NUT family member 1 OS=Homo sapiens OX=	--
evm.model.HIC_ASM_9.1157	NUT family	NUT family member 2D OS=Homo sapiens OX	--
evm.model.HIC_ASM_12.425	Glyceraldehy	--	K00134 gly
evm.model.HIC_ASM_0.1240	glyceraldehy	--	--
evm.model.HIC_ASM_16.338	putative 60S	Putative 60S ribosomal protein L37a OS=Rattu	K02921 lar
evm.model.HIC_ASM_8.861	putative 60S	Putative 60S ribosomal protein L37a OS=Rattu	K02921 lar
evm.model.HIC_ASM_2.2001	60S ribosoma	60S ribosomal protein L37a OS=Gallus gallus (	K02921 lar
evm.model.HIC_ASM_0.1434	putative 60S	Putative 60S ribosomal protein L37a OS=Rattu	K02921 lar
evm.model.HIC_ASM_2.392	putative 60S	Putative 60S ribosomal protein L37a OS=Rattu	K02921 lar
evm.model.HIC_ASM_21.615	60S ribosoma	60S ribosomal protein L37a OS=Gallus gallus (	K02921 lar
evm.model.HIC_ASM_18.832	putative 60S	Putative 60S ribosomal protein L37a OS=Rattu	K02921 lar
evm.model.HIC_ASM_8.548	putative 60S	60S ribosomal protein L37a OS=Gallus gallus (	K02921 lar
evm.model.HIC_ASM_7.1047	PREDICTEI	60S ribosomal protein L37a OS=Rattus norveg	K02921 lar
evm.model.HIC_ASM_5.262	putative 60S	Putative 60S ribosomal protein L37a OS=Rattu	K02921 lar

evm.model.HIC_ASM_5.1703	MAM and LI	MAM and LDL-receptor class A domain-conta	K18762 zy
evm.model.HIC_ASM_5.1702	MAM and LI	MAM and LDL-receptor class A domain-conta	K18762 zy
evm.model.HIC_ASM_3.1110	hypothetical	Protocadherin gamma-A2 OS=Pan troglodytes	K16495 pr
evm.model.HIC_ASM_0.202	protocadheri	Protocadherin Fat 1 OS=Homo sapiens OX=96	K16506 pr
evm.model.HIC_ASM_18.630	u1 small nucl	U1 small nuclear ribonucleoprotein A OS=Sus	K11091 U1
evm.model.HIC_ASM_21.108	U2 small nuc	U2 small nuclear ribonucleoprotein B" OS=Ho	K11094 U2
evm.model.HIC_ASM_9.422	U2 small nuc	U2 small nuclear ribonucleoprotein B" OS=Ho	K11094 U2
evm.model.HIC_ASM_17.3	complement	Complement factor H OS=Homo sapiens OX=9	K04004 co
evm.model.HIC_ASM_17.181	PREDICTED	Complement factor H-related protein 5 OS=Ho	K04004 co
evm.model.HIC_ASM_17.516	complement	Complement factor H OS=Bos taurus OX=991	K04004 co
evm.model.HIC_ASM_17.180	PREDICTED	Coagulation factor XIII B chain OS=Homo sap	K03906 co
evm.model.HIC_ASM_17.370	complement	Complement factor H-related protein 2 OS=Ho	K04004 co
evm.model.HIC_ASM_17.518	complement	Complement factor H OS=Bos taurus OX=991	K04004 co
evm.model.HIC_ASM_20.133	--	--	--
evm.model.HIC_ASM_20.132	--	--	--
evm.model.HIC_ASM_9.549	glutamate rec	Glutamate receptor ionotropic, NMDA 3A OS=	K05213 glu
evm.model.HIC_ASM_7.957	PREDICTED	Glutamate receptor 3 OS=Mus musculus OX=1	K05199 glu
evm.model.HIC_ASM_2.562	unconvention	Unconventional myosin-Ib OS=Mus musculus (	K10356 my
evm.model.HIC_ASM_21.382	myosin-7B [	Myosin-7B OS=Homo sapiens OX=9606 GN=	K10352 my
evm.model.HIC_ASM_2.1778	basic salivary	--	--
evm.model.HIC_ASM_2.1466	transport and	--	--
evm.model.HIC_ASM_2.1529	basic salivary	--	--
evm.model.CTG_203.5	transport and	--	--
evm.model.CTG_1294.2	transport and	--	--
evm.model.CTG_1678.2	transport and	Transport and Golgi organization protein 1 hon	K05692 ac
evm.model.CTG_1422.2	transport and	Transport and Golgi organization protein 1 hon	K05692 ac
evm.model.HIC_ASM_2.1425	transport and	Transport and Golgi organization protein 1 hon	K05692 ac
evm.model.CTG_203.4	transport and	Transport and Golgi organization protein 1 hon	K05692 ac
evm.model.HIC_ASM_2.1426	transport and	--	--
evm.model.CTG_1678.1	transport and	Transport and Golgi organization protein 1 hon	K05692 ac
evm.model.HIC_ASM_18.51	nadh dehydro	NADH dehydrogenase [ubiquinone] 1 alpha sul	K03947 NA
evm.model.HIC_ASM_20.150	PREDICTED	NADH dehydrogenase [ubiquinone] 1 alpha sul	K03947 NA
evm.model.HIC_ASM_6.259	POM121-like	POM121-like protein 2 OS=Homo sapiens OX	K14316 nu
evm.model.HIC_ASM_11.83	PREDICTED	POM121-like protein 12 OS=Homo sapiens OX	K14316 nu
evm.model.HIC_ASM_0.303	non-histone c	Non-histone chromosomal protein HMG-17 OS	K11300 hig
evm.model.HIC_ASM_15.241	Hypothetical	High mobility group nucleosome-binding doma	K11300 hig
evm.model.HIC_ASM_7.949	non-histone c	High mobility group nucleosome-binding doma	K11300 hig
evm.model.HIC_ASM_0.689	high mobility	High mobility group nucleosome-binding doma	K11302 hig
evm.model.HIC_ASM_6.246	PREDICTED	High mobility group nucleosome-binding doma	K11302 hig
evm.model.HIC_ASM_1.547	non-histone c	High mobility group nucleosome-binding doma	K11300 hig
evm.model.HIC_ASM_1.1031	High mobility	Non-histone chromosomal protein HMG-17 OS	K11300 hig
evm.model.HIC_ASM_10.594	non-histone c	Non-histone chromosomal protein HMG-17 OS	K11300 hig
evm.model.HIC_ASM_8.1124	brain acid so	Non-histone chromosomal protein HMG-17 OS	K11300 hig
evm.model.HIC_ASM_15.249	carbonic anh	Carbonic anhydrase 13 OS=Mus musculus OX=	K01672 ca
evm.model.HIC_ASM_18.109	carbonic anh	Carbonic anhydrase 7 OS=Homo sapiens OX=9	K01672 ca

GO_anno
GO:0003735|GO:0005840|GO:0006412
6/2/2020
6/2/2020
6/2/2020
40S ribosomal protein S11, N-terminal
6/2/2020
6/2/2020
--
--
CS domain
Nascent polypeptide-associated complex NAC domain
6/2/2020
6/2/2020
GO:0003700|GO:0006355
GO:0003700|GO:0006355
GO:0003700|GO:0006355
--
--
GO:0004984|GO:0007186|GO:0016021
6/2/2020
GO:0004555|GO:0005991
GO:0004555|GO:0005991
6/2/2020
GO:0006816|GO:0016020
6/2/2020
6/2/2020
GO:0003735|GO:0005840|GO:0006412
GO:0003735|GO:0005840|GO:0006412
6/2/2020
GO:0003735|GO:0005840|GO:0006412
6/2/2020
6/2/2020
6/2/2020
6/2/2020
6/2/2020
GO:0003735|GO:0005840|GO:0006412
GO:0003735|GO:0005840|GO:0006412
6/2/2020
6/2/2020
6/2/2020
6/2/2020
6/2/2020
6/2/2020
GO:0003735|GO:0005840|GO:0006412
6/2/2020
GO:0003735|GO:0005840|GO:0006412

6/2/2020
GO:0003735|GO:0005840|GO:0006412
6/2/2020
GO:0003735|GO:0005840|GO:0006412
6/2/2020
6/2/2020
GO:0003735|GO:0005840|GO:0006412
GO:0003735|GO:0005840|GO:0006412
GO:0003735|GO:0005840|GO:0006412
GO:0003735|GO:0005840|GO:0006412
6/2/2020
GO:0003735|GO:0005840|GO:0006412
6/2/2020
GO:0000413|GO:0003755
GO:0000413|GO:0003755
GO:0000413|GO:0003755
GO:0000413|GO:0003755
6/2/2020
GO:0000413|GO:0003755
6/2/2020
6/2/2020
6/2/2020
6/2/2020
GO:0000413|GO:0003755
GO:0016787
GO:0016787
6/2/2020
Ribosomal protein L1/ribosomal biogenesis protein
Ribosomal protein L1/ribosomal biogenesis protein
6/2/2020
6/2/2020
6/2/2020
Prefoldin alpha-like
6/2/2020
6/2/2020
GO:0003676|GO:0008270
6/2/2020
GO:0005743|GO:0022857|GO:0055085
6/2/2020
6/2/2020
GO:0005634|GO:0051726
GO:0005634|GO:0051726
GO:0007276
6/2/2020
6/2/2020
GO:0005524|GO:0016887
Type I protein exporter
6/2/2020

Ataxin-10 domain
GO:0003735|GO:0005840|GO:0006412
GO:0003735|GO:0005840|GO:0006412
6/2/2020
GO:0003735|GO:0005840|GO:0006412
GO:0003735|GO:0005840|GO:0006412
6/2/2020
6/2/2020
6/2/2020
6/2/2020
GO:0003735|GO:0005840|GO:0006412
GO:0003735|GO:0005840|GO:0006412
GO:0003735|GO:0005840|GO:0006412
6/2/2020
Elongation factor 1 beta central acidic region, eukaryote
GO:0003746|GO:0006414
--
--
--
GO:0005886|GO:0007155
GO:0007155|GO:0007420
6/2/2020
6/2/2020
6/2/2020
6/2/2020
Protein-tyrosine phosphatase, catalytic
GO:0004725|GO:0006470
6/2/2020
GO:0035556
--
GO:0035556
GO:0005515
GO:0005515
GO:0005515
6/2/2020
6/2/2020
6/2/2020
6/2/2020
6/2/2020
6/2/2020
6/2/2020
6/2/2020
6/2/2020
6/2/2020
GO:0003735|GO:0005840|GO:0006412
6/2/2020
GO:0003735|GO:0005840|GO:0006412
GO:0003735|GO:0005840|GO:0006412

6/2/2020
Immunoglobulin-like domain
6/2/2020
6/2/2020
6/2/2020
6/2/2020
6/2/2020
6/2/2020
6/2/2020
6/2/2020
6/2/2020
6/2/2020
6/2/2020
Zinc finger, PHD-type
CHD, N-terminal
GO:0005219|GO:0006874|GO:0016021
6/2/2020
6/2/2020

Laminin G domain
6/2/2020
Laminin G domain
--
--
--
--
--
6/2/2020
6/2/2020
GO:0003735|GO:0005840|GO:0006412
6/2/2020
6/2/2020
Coagulation factor 5/8 C-terminal domain
Coagulation factor 5/8 C-terminal domain
6/2/2020
6/2/2020
GO:0005740|GO:0016021
6/2/2020
GO:0005515
GO:0003677
GO:0003677
GO:0003735|GO:0005840|GO:0006412
6/2/2020
6/2/2020
6/2/2020
GO:0003735|GO:0005840|GO:0006412
6/2/2020
GO:0003735|GO:0005840|GO:0006412

6/2/2020
6/2/2020
Peptidase M12B, propeptide
6/2/2020
6/2/2020
GO:0005515
6/2/2020
6/2/2020
GO:0005634|GO:0006355
GO:0005634|GO:0006355
6/2/2020
6/2/2020
6/2/2020
GO:0006979|GO:0016671|GO:0030091
GO:0033743|GO:0055114
Autism susceptibility gene 2 protein
6/2/2020
6/2/2020
Protein of unknown function DUF842, eukaryotic
Protein of unknown function DUF842, eukaryotic
GO:0005525
6/2/2020
GO:0016021
GO:0016021
GO:0004984|GO:0007186|GO:0016021
6/2/2020
6/2/2020
6/2/2020
GO:0016021
GO:0016021
--
--
6/2/2020
6/2/2020
6/2/2020
6/2/2020
GO:0003735|GO:0005840|GO:0006412
GO:0003735|GO:0005840|GO:0006412
6/2/2020
6/2/2020
6/2/2020
6/2/2020
GO:0003743|GO:0006413
GO:0003743|GO:0006413
GO:0003743|GO:0006413
6/2/2020
GO:0006596
GO:0005515

Ankyrin repeat-containing domain
GO:0005515
Immunoglobulin-like domain
6/2/2020
Immunoglobulin C1-set
GO:0003677
GO:0003677
--
--
--
--
Intermediate filament, rod domain
GO:0045095
GO:0045095
6/2/2020
6/2/2020
GO:0007155|GO:0051015
6/2/2020
6/2/2020
Melanoma-associated antigen
Melanoma-associated antigen
--
--
GO:0035064
GO:0035064
GO:0035064
DnaJ domain
6/2/2020
6/2/2020
GO:0005515
--
--
GO:0003735|GO:0005840|GO:0006412
GO:0003735|GO:0005840|GO:0006412
GO:0003735|GO:0005840|GO:0006412
GO:0003735|GO:0005840|GO:0006412
GO:0005525
6/2/2020
6/2/2020
6/2/2020
6/2/2020
GO:0005525
GO:0005525
GO:0005515
GO:0005515
6/2/2020
6/2/2020
GO:0005515

Rad60/SUMO-like domain
6/2/2020
6/2/2020
6/2/2020
6/2/2020
GO:0003924|GO:0005525
6/2/2020
--
--
6/2/2020
6/2/2020
Immunoglobulin V-set domain
Immunoglobulin V-set domain
6/2/2020
6/2/2020
6/2/2020
GO:0004674
6/2/2020
6/2/2020
6/2/2020
6/2/2020
6/2/2020
6/2/2020
EGF-like domain
6/2/2020
6/2/2020
Zona pellucida domain
Protein of unknown function DUF4641
Protein of unknown function DUF4641
Protein of unknown function DUF4641
6/2/2020
6/2/2020
Protein of unknown function DUF4641
Ly-6 antigen/uPA receptor-like
6/2/2020
--
Guanylate kinase-like domain
6/2/2020
Lipoma HMGIC fusion partner-like protein
Lipoma HMGIC fusion partner-like protein
6/2/2020
6/2/2020
Nucleoplasmin family
GO:0003676
6/2/2020
GO:0003735|GO:0005840|GO:0006412
GO:0003735|GO:0005840|GO:0006412
6/2/2020

Nuclear transport factor 2
Nuclear transport factor 2, eukaryote
6/2/2020
Translation elongation factor EFTu/EF1A, C-terminal
GO:0003924|GO:0005525
GO:0003924|GO:0005525
6/2/2020
6/2/2020
GO:0005525
GO:0005525
GO:0003924|GO:0005525
6/2/2020
--
--
6/2/2020
GO:0003735|GO:0005840|GO:0006412
6/2/2020
GO:0003735|GO:0005840|GO:0006412
6/2/2020
6/2/2020
Ribosomal protein L18e/L15P
6/2/2020
6/2/2020
Ribosomal protein L18e/L15P
Ribosomal protein L18e/L15P
6/2/2020
6/2/2020
6/2/2020
6/2/2020
6/2/2020
Ribosomal protein L18e/L15P
6/2/2020
6/2/2020
C2 domain
C2 domain
GO:0004930|GO:0007186|GO:0016021
GO:0016021
GO:0004672|GO:0005524|GO:0006468
GO:0004672|GO:0005524|GO:0006468
Guanylate kinase/L-type calcium channel beta subunit
6/2/2020
PDZ-associated domain of NMDA receptors
GO:0005509|GO:0007156|GO:0016020
6/2/2020
6/2/2020
Immunoglobulin V-set domain
6/2/2020
Immunoglobulin-like domain

6/2/2020
6/2/2020
Immunoglobulin-like domain
6/2/2020
6/2/2020
Immunoglobulin V-set domain
6/2/2020
Immunoglobulin-like domain
6/2/2020
GO:0005198|GO:0045095
6/2/2020
6/2/2020
6/2/2020
GO:0005198|GO:0045095
GO:0005524|GO:0006139|GO:0019205
6/2/2020
6/2/2020
GO:0003735|GO:0005840|GO:0006412
GO:0003735|GO:0005840|GO:0006412
GO:0003735|GO:0005840|GO:0006412
6/2/2020
6/2/2020
GO:0003682
--
--
--
--
--
--
--
6/2/2020
6/2/2020
GO:0003735|GO:0005840|GO:0006412
6/2/2020
6/2/2020
6/2/2020
GO:0016021|GO:0055085
6/2/2020
GO:0008168
GO:0000786|GO:0003677
GO:0000786|GO:0003677
GO:0000786|GO:0003677
6/2/2020
6/2/2020
6/2/2020
6/2/2020
6/2/2020
GO:0003735|GO:0005840|GO:0006412

GO:0003735|GO:0005840|GO:0006412
Snake toxin/toxin-like
Ly-6 antigen/uPA receptor-like
Ly-6 antigen/uPA receptor-like
Snake toxin/toxin-like
6/2/2020
Ribosomal protein L11, N-terminal
6/2/2020
6/2/2020
GO:0003735|GO:0005840|GO:0006412
GO:0003735|GO:0005840|GO:0006412
6/2/2020
GO:0003735|GO:0005840|GO:0006412
GO:0003735|GO:0005840|GO:0006412
Ribosomal protein L11, N-terminal
GO:0003735|GO:0005840|GO:0006412
GO:0003735|GO:0005840|GO:0006412
GO:0003735|GO:0005840|GO:0006412
GO:0003735|GO:0005840|GO:0006412
GO:0003735|GO:0005840|GO:0006412
Ribosomal protein L11, N-terminal
GO:0003735|GO:0005840|GO:0006412
6/2/2020
6/2/2020
GO:0003735|GO:0005840|GO:0006412
Ribosomal protein L11, N-terminal
GO:0003735|GO:0005840|GO:0006412
GO:0003735|GO:0005840|GO:0006412
--
6/2/2020
Mitochondrial substrate/solute carrier
GO:0005509
Mitochondrial substrate/solute carrier
GO:0003735|GO:0005840|GO:0006412
6/2/2020
GO:0003735|GO:0005840|GO:0006412
Plexin, TIG domain 1
GO:0005515
Mitochondrial F1-F0 ATP synthase subunit F, predicted
Mitochondrial F1-F0 ATP synthase subunit F, predicted
Mitochondrial F1-F0 ATP synthase subunit F, predicted
6/2/2020
6/2/2020
6/2/2020
6/2/2020
Mitochondrial F1-F0 ATP synthase subunit F, predicted
GO:0003735|GO:0005840|GO:0006412|GO:0019843
6/2/2020

6/2/2020
6/2/2020
GO:0005759
GO:0005759
--
GO:0016620|GO:0055114
GO:0016620|GO:0055114
GO:0016620|GO:0055114
GO:0016620|GO:0055114
GO:0016620|GO:0055114
Immunoglobulin C1-set
6/2/2020
6/2/2020
Immunoglobulin C1-set
Immunoglobulin C1-set
6/2/2020
Immunoglobulin subtype
GO:0004930|GO:0007186|GO:0016021
GO:0007186|GO:0016021
6/2/2020
6/2/2020
GO:0004930|GO:0007186|GO:0016021
6/2/2020
GO:0005524
AAA+ ATPase domain
6/2/2020
6/2/2020
Ribosomal protein S10 domain
GO:0003735|GO:0005840|GO:0006412
Ribosomal protein S10 domain
6/2/2020
GO:0003735|GO:0005840|GO:0006412
Ribosomal protein S10 domain
6/2/2020
6/2/2020
6/2/2020
GO:0006614|GO:0016021
GO:0006614|GO:0016021
GO:0006614|GO:0016021
6/2/2020
GO:0005158|GO:0008286
GO:0005887|GO:0007155|GO:0042043|GO:0045202|GO:0050808
6/2/2020
GO:0031110
GO:0031110
GO:0008270
6/2/2020
6/2/2020

6/2/2020
Developmental pluripotency-associated protein 4
GO:0003682
6/2/2020
GO:0003777|GO:0007018
Dynein heavy chain AAA lid domain
GO:0003777|GO:0007018
6/2/2020
6/2/2020
GO:0006457
GO:0006457
6/2/2020
GO:0006457
6/2/2020
GO:0005747|GO:0022904
GO:0005747|GO:0022904
GO:0007186|GO:0016021|GO:0016503
GO:0007186|GO:0016021|GO:0016503
GO:0003676
GO:0003676
6/2/2020
39S ribosomal protein L42, mitochondrial
39S ribosomal protein L42, mitochondrial
39S ribosomal protein L42, mitochondrial
39S ribosomal protein L42, mitochondrial
39S ribosomal protein L42, mitochondrial
39S ribosomal protein L42, mitochondrial
STAG
Cohesin subunit Scc3/SA
--
--
6/2/2020
Uncharacterised protein family, TMEM59
40S Ribosomal protein S10
Plectin/S10, N-terminal
GO:0003676|GO:0006355
GO:0003676|GO:0006355
6/2/2020
6/2/2020
Dynein heavy chain, domain-1
6/2/2020
--
--
Calmodulin-regulated spectrin-associated protein, CH domain
6/2/2020
Calmodulin-regulated spectrin-associated protein, CH domain
6/2/2020
GO:0003924|GO:0005525

GO:0003924|GO:0005525
6/2/2020
GO:0003735|GO:0005840|GO:0006412
6/2/2020
6/2/2020
6/2/2020
GO:0005515
6/2/2020
6/2/2020
Immunoglobulin V-set domain
GO:0003924
6/2/2020
Immunoglobulin V-set domain
6/2/2020
Immunoglobulin subtype
6/2/2020
Immunoglobulin-like domain
6/2/2020
6/2/2020
GO:0003735|GO:0005840|GO:0006412
GO:0003735|GO:0005840|GO:0006412
6/2/2020
6/2/2020
6/2/2020
6/2/2020
6/2/2020
GO:0003735|GO:0005840|GO:0006412
Ribosomal protein L23/L25, N-terminal
6/2/2020
tRNA(Ile)-lysidine/2-thiocytidine synthase, N-terminal
Cytoplasmic tRNA 2-thiolation protein 1, C-terminal
GO:0003735|GO:0005840|GO:0006412
GO:0003735|GO:0005840|GO:0006412
Gasdermin, PUB domain
Gasdermin, pore forming domain
Gasdermin, pore forming domain
Gasdermin, pore forming domain
Gasdermin
Gasdermin, pore forming domain
GO:0005085|GO:0007264
GO:0005085|GO:0007264
DHR-1 domain
Neurexin/syndecan/glycophorin C
6/2/2020
6/2/2020
6/2/2020
GO:0016491|GO:0055114
Formin, FH2 domain

Formin, FH2 domain
Translationally controlled tumour protein
Translationally controlled tumour protein
6/2/2020
6/2/2020
6/2/2020
6/2/2020
6/2/2020
6/2/2020
6/2/2020
6/2/2020
6/2/2020
6/2/2020
6/2/2020
--
--
GO:0022857|GO:0055085
6/2/2020
GO:0022857|GO:0055085
GO:0016620|GO:0055114
GO:0016620|GO:0055114
GO:0016620|GO:0055114
6/2/2020
6/2/2020
GO:0016620|GO:0055114
GO:0016620|GO:0055114
GO:0016620|GO:0055114
GO:0016620|GO:0055114
GO:0016620|GO:0055114
GO:0016620|GO:0055114
GO:0016620|GO:0055114
GO:0016620|GO:0055114
GO:0016620|GO:0055114
GO:0016620|GO:0055114
GO:0016620|GO:0055114
Immunoglobulin-like domain
6/2/2020
Immunoglobulin subtype
GO:0005515
Ankyrin repeat-containing domain
GO:0005515
Domain of unknown function DUF3496
Ankyrin repeat-containing domain
6/2/2020
6/2/2020
GO:0015078|GO:0015991|GO:0033177
GO:0015078|GO:0015986|GO:0045263
6/2/2020

6/2/2020
6/2/2020
6/2/2020
6/2/2020
6/2/2020
6/2/2020
6/2/2020
6/2/2020
--
--
--
6/2/2020
6/2/2020
6/2/2020
GO:0005742|GO:0006605|GO:0006886
GO:0005742|GO:0006605|GO:0006886
GO:0005742|GO:0006605|GO:0006886
GO:0016020
6/2/2020
GO:0016020
6/2/2020
Protein of unknown function DUF3512
Protein of unknown function DUF3512
Protein of unknown function DUF3512
6/2/2020
6/2/2020
GO:0004672|GO:0005524|GO:0006468
GO:0004672|GO:0006468
6/2/2020
6/2/2020
GO:0003735|GO:0005840|GO:0006412
6/2/2020
6/2/2020
6/2/2020
6/2/2020
6/2/2020
6/2/2020
6/2/2020
6/2/2020
6/2/2020
GO:0005509
GO:0005509
GO:0005509
GO:0005509
6/2/2020
6/2/2020
GO:0005509
GO:0005509|GO:0019722

GO:0005509|GO:0019722
Ribosomal protein S8e/ribosomal biogenesis NSA2
Ribosomal protein S8e/ribosomal biogenesis NSA2
Ribosomal biogenesis NSA2 family
6/2/2020
6/2/2020
6/2/2020
Stromalin conservative domain
6/2/2020
Cohesin subunit Scc3/SA
Cohesin subunit Scc3/SA
STAG
Domain of unknown function DUF4939
LDOC1-related
6/2/2020
Domain of unknown function DUF4939
Domain of unknown function DUF4939
Domain of unknown function DUF4939
GO:0005200|GO:0005525|GO:0005874|GO:0007017
GO:0005200|GO:0005525|GO:0005874|GO:0007017
6/2/2020
GO:0005874|GO:0007017
6/2/2020
6/2/2020
Transmembrane protein TMEM132, N-terminal
GO:0005085|GO:0007264|GO:0030036
Dedicator of cytokinesis, N-terminal domain
Dedicator of cytokinesis, C-terminal
GO:0005856
FERM, N-terminal
6/2/2020
6/2/2020
Centrosomal protein C10orf90, N-terminal
6/2/2020
BAT2, N-terminal
6/2/2020
6/2/2020
Immunoglobulin-like domain
Immunoglobulin subtype 2
6/2/2020
6/2/2020
Jacalin-like lectin domain
Jacalin-like lectin domain
Jacalin-like lectin domain
Jacalin-like lectin domain
6/2/2020
--
--

6/2/2020
GO:0003676|GO:0006355
6/2/2020
6/2/2020
6/2/2020
6/2/2020
GO:0003676
6/2/2020
GO:0003676
GO:0003676
GO:0003676
6/2/2020
GO:0003676
6/2/2020
Nuclear factor hnRNPA1
GO:0003676
GO:0003676
GO:0003676
GO:0003676
Cysteine-rich flanking region, C-terminal
6/2/2020
GO:0007155
6/2/2020
6/2/2020
Receptor, ligand binding region
GO:0003735|GO:0005840|GO:0006412
6/2/2020
6/2/2020
GO:0003735|GO:0005840|GO:0006412
GO:0003735|GO:0005840|GO:0006412
6/2/2020
GO:0003735|GO:0005840|GO:0006412
6/2/2020
GO:0003735|GO:0005840|GO:0006412
GO:0003735|GO:0005840|GO:0006412
GO:0003735|GO:0005840|GO:0006412
GO:0003723
GO:0003723
GO:0003723
Ribosomal protein L7Ae/L30e/S12e/Gadd45
GO:0003723
GO:0003723
GO:0003723
GO:0003723|GO:0003735|GO:0022625
GO:0000339|GO:0005846|GO:0045292
6/2/2020
GO:0000339|GO:0005846|GO:0045292
GO:0003735|GO:0005840|GO:0006412

GO:0003735|GO:0005840|GO:0006412
GO:0003735|GO:0005840|GO:0006412
GO:0003735|GO:0005840|GO:0006412
GO:0003735|GO:0005840|GO:0006412
GO:0003735|GO:0005840|GO:0006412
6/2/2020
GO:0003735|GO:0005840|GO:0006412
TB domain
GO:0005509
GO:0005515
GO:0005515
Tyrosine-protein kinase ephrin type A/B receptor-like
GO:0005515
GO:0005515
6/2/2020
6/2/2020
6/2/2020
6/2/2020
6/2/2020
GO:0003779|GO:0030833
6/2/2020
Ras-like guanine nucleotide exchange factor, N-terminal
Ras-like guanine nucleotide exchange factor, N-terminal
6/2/2020
Ras-like guanine nucleotide exchange factor, N-terminal
Immunoglobulin V-set domain
Immunoglobulin-like domain
Immunoglobulin V-set domain
6/2/2020
Immunoglobulin V-set domain
Immunoglobulin V-set domain
6/2/2020
Immunoglobulin V-set domain
6/2/2020
Immunoglobulin subtype
6/2/2020
Immunoglobulin V-set domain
High mobility group box domain
GO:0016514|GO:0043044
GO:0016514|GO:0043044
GO:0005515
6/2/2020
6/2/2020
6/2/2020
Immunoglobulin subtype 2
GO:0003735|GO:0005840|GO:0006412
6/2/2020
6/2/2020

GO:0003735|GO:0005840|GO:0006412
GO:0003735|GO:0005840|GO:0006412
6/2/2020
6/2/2020
GO:0006897|GO:0030276
GO:0006897|GO:0030276
GO:0003735|GO:0005840|GO:0006412|GO:0019843
GO:0003735|GO:0005840|GO:0006412|GO:0019843
GO:0003735|GO:0005840|GO:0006412|GO:0019843
GO:0003735|GO:0005840|GO:0006412|GO:0019843
GO:0003735|GO:0005840|GO:0006412|GO:0019843
GO:0003735|GO:0005840|GO:0006412|GO:0019843
6/2/2020
GO:0003735|GO:0005840|GO:0006412|GO:0019843
GO:0003735|GO:0005840|GO:0006412|GO:0019843
6/2/2020
GO:0003735|GO:0005840|GO:0006412|GO:0019843
6/2/2020
Secretoglobin
Secretoglobin
Immunoglobulin-like domain
CD80-like, immunoglobulin C2-set
6/2/2020
6/2/2020
Cytochrome b5-like heme/steroid binding domain
Cytochrome b5-like heme/steroid binding domain
GO:0005215|GO:0016020|GO:0055085
6/2/2020
GO:0004864|GO:0009966|GO:0043666
GO:0004864|GO:0009966|GO:0043666
GO:0004864|GO:0009966|GO:0043666
Peptidase family A1 domain
6/2/2020
6/2/2020
Peptidase M12B, propeptide
GO:0004222|GO:0006508
Peptidase M12B, propeptide
GO:0003735|GO:0005840|GO:0006412
6/2/2020
GO:0003735|GO:0005840|GO:0006412
GO:0003735|GO:0005840|GO:0006412
6/2/2020
--
6/2/2020
6/2/2020
6/2/2020
GO:0003677
GO:0007165|GO:0016021

6/2/2020
GO:0007165|GO:0016021
GO:0007165|GO:0016021
Protein FRG2
Protein FRG2
GO:0003735|GO:0005840|GO:0006412
6/2/2020
GO:0004672|GO:0006468
6/2/2020
Testis-expressed sequence 13 protein family
Zinc finger, RanBP2-type
6/2/2020
--
--
GO:0007186|GO:0016021|GO:0016503
GO:0007186|GO:0016021|GO:0016503
FAM90
6/2/2020
FAM90
6/2/2020
6/2/2020
6/2/2020
6/2/2020
FAM90
FAM90
FAM90
Immunoglobulin subtype
6/2/2020
Phosphatidylethanolamine-binding protein
6/2/2020
6/2/2020
GO:0005960|GO:0019464
Biotin/lipoyl attachment
Phosphatidylinositol 3-/4-kinase, catalytic domain
GO:0005515
FAM209 family
6/2/2020
6/2/2020
6/2/2020
GO:0005201
6/2/2020
GO:0005201
6/2/2020
6/2/2020
Protein-tyrosine phosphatase, catalytic
GO:0008138|GO:0016311
6/2/2020
6/2/2020

GO:0003735|GO:0005840|GO:0006412
GO:0003735|GO:0005840|GO:0006412
6/2/2020
6/2/2020
--
--
Sushi/SCR/CCP domain
Sushi/SCR/CCP domain
6/2/2020
CCDC144C-like, coiled-coil domain
6/2/2020
6/2/2020
6/2/2020
GO:0016846
6/2/2020
6/2/2020
6/2/2020
6/2/2020
GO:0003735|GO:0005840|GO:0006412
6/2/2020
6/2/2020
6/2/2020
6/2/2020
6/2/2020
6/2/2020
6/2/2020
6/2/2020
6/2/2020
6/2/2020
6/2/2020
Domain of unknown function DUF3699
6/2/2020
6/2/2020
6/2/2020
Ras-like guanine nucleotide exchange factor, N-terminal
6/2/2020
Ras-like guanine nucleotide exchange factor, N-terminal
6/2/2020
6/2/2020
6/2/2020
6/2/2020
6/2/2020
6/2/2020
GO:0005509|GO:0007156|GO:0016020
6/2/2020
GO:0007017|GO:0030286
GO:0007017|GO:0030286
--

--
--
--
TMEM238 protein
TMEM238 protein
GO:0006511
GO:0006511
6/2/2020
6/2/2020
6/2/2020
GO:0005515
6/2/2020
6/2/2020
6/2/2020
Butyrophylin-like, SPRY domain
Butyrophylin-like, SPRY domain
SPRY-associated
Zinc finger, RING-type
Butyrophylin-like, SPRY domain
6/2/2020
6/2/2020
--
6/2/2020
Coiled-coil domain-containing protein 144C
Phosphopantetheine binding ACP domain
6/2/2020
6/2/2020
6/2/2020
AMP-binding enzyme, C-terminal domain
6/2/2020
6/2/2020
GO:0005506|GO:0016705|GO:0020037|GO:0055114
EGF-like, conserved site
EGF-like domain
6/2/2020
6/2/2020
6/2/2020
GO:0000124|GO:0003712
GO:0000124|GO:0003712
6/2/2020
6/2/2020
6/2/2020
6/2/2020
--
--
--
6/2/2020
6/2/2020

GO:0005634|GO:0006355
GO:0003735|GO:0005840|GO:0006412
GO:0003735|GO:0005840|GO:0006412
--
--
GO:0003676
6/2/2020
--
--
6/2/2020
MHC class I-like antigen recognition-like
6/2/2020
6/2/2020
MHC class I-like antigen recognition-like
6/2/2020
6/2/2020
6/2/2020
6/2/2020
6/2/2020
6/2/2020
6/2/2020
6/2/2020
GO:0003735|GO:0005840|GO:0006412
6/2/2020
6/2/2020
6/2/2020
6/2/2020
6/2/2020
6/2/2020
GO:0003677|GO:0006275|GO:0030337
6/2/2020
6/2/2020
GO:0000387|GO:0000398|GO:0005685
Nuclear Testis protein, N-terminal
6/2/2020
Nuclear Testis protein, N-terminal
6/2/2020
--
GO:0003735|GO:0005840|GO:0006412
GO:0003735|GO:0005840|GO:0006412
GO:0003735|GO:0005840|GO:0006412
GO:0003735|GO:0005840|GO:0006412
GO:0003735|GO:0005840|GO:0006412
GO:0003735|GO:0005840|GO:0006412
GO:0003735|GO:0005840|GO:0006412
GO:0003735|GO:0005840|GO:0006412
GO:0003735|GO:0005840|GO:0006412
GO:0003735|GO:0005840|GO:0006412

GO:0005515
6/2/2020
6/2/2020
6/2/2020
GO:0003676
6/2/2020
6/2/2020
6/2/2020
6/2/2020
6/2/2020
Sushi/SCR/CCP domain
Sushi/SCR/CCP domain
Sushi/SCR/CCP domain
--
--
6/2/2020
6/2/2020
GO:0003774|GO:0005524|GO:0016459
6/2/2020
--
--
--
--
--
--
--
6/2/2020
--
--
--
GO:0005743|GO:0005747
GO:0005743|GO:0005747
6/2/2020
Nuclear pore complex protein
6/2/2020
GO:0000785|GO:0005634|GO:0031492
GO:0000785|GO:0005634|GO:0031492
6/2/2020
6/2/2020
GO:0000785|GO:0005634|GO:0031492
6/2/2020
GO:0000785|GO:0005634|GO:0031492
GO:0000785|GO:0005634|GO:0031492
6/2/2020
6/2/2020

Supplementary Table S8 Genes in significant contracted gene families in masked palm civet.

Gene ID	NR_descripti	Swissprot_description	KEGG_an	GO_anno
evm.model.HIC_ASM	PREDICTED	Olfactory receptor 10 OS=Mus musc	K04257 ol	6/2/2020
evm.model.HIC_ASM	olfactory rec	Olfactory receptor 2B2 OS=Homo sa	K04257 ol	GO:0004984|GO:0007
evm.model.HIC_ASM	c-type lectin	C-type lectin domain family 12 meml	K17516 C-	C-type lectin-like
evm.model.HIC_ASM	LOW QUAL	C-type lectin domain family 9 membe	K17515 C-	C-type lectin-like
evm.model.HIC_ASM	PREDICTED	C-type lectin domain family 1 membe	K10069 C-	C-type lectin-like
evm.model.HIC_ASM	PREDICTED	C-type lectin domain family 1 membe	K10070 C-	C-type lectin-like
evm.model.HIC_ASM	PREDICTED	Oxidized low-density lipoprotein rece	K08763 ox	C-type lectin-like
evm.model.HIC_ASM	protocadheri	Protocadherin beta-1 OS=Pan trogloc	K16494 pr	6/2/2020
evm.model.HIC_ASM	IgG receptor	IgG receptor FcRn large subunit p51	K06751 M	MHC class I alpha cha
evm.model.HIC_ASM	class i histoc	HLA class I histocompatibility antige	K06751 M	6/2/2020
evm.model.HIC_ASM	hereditary he	Hereditary hemochromatosis protein	K06751 M	MHC class I-like antig
evm.model.HIC_ASM	putative prot	Zinc finger protein 736 OS=Homo sa	K09228 Kl	6/2/2020
evm.model.HIC_ASM	PREDICTED	Putative protein ZNF720 OS=Homo s	K09228 Kl	GO:0003676|GO:0006
evm.model.HIC_ASM	PREDICTED	Olfactory receptor 6T1 OS=Homo sa	K04257 ol	GO:0016021
evm.model.HIC_ASM	transmembra	Transmembrane emp24 domain-conta	K20346 p2	GOLD domain
evm.model.HIC_ASM	PREDICTED	Transmembrane emp24 domain-conta	K20346 p2	6/2/2020
evm.model.HIC_ASM	E3 ubiquitin-	E3 ubiquitin-protein ligase TRIM38 (	K12014 tri	6/2/2020
evm.model.HIC_ASM	LOW QUAL	E3 ubiquitin-protein ligase TRIM21 (	K10651 tri	GO:0005515
evm.model.HIC_ASM	E3 ubiquitin-	E3 ubiquitin-protein ligase TRIM4 O	K11998 tri	Butyrophylin-like, SPI
evm.model.CTG_133	E3 ubiquitin-	E3 ubiquitin-protein ligase TRIM58 (	K12027 tri	SPRY-associated
evm.model.HIC_ASM	TRIM39-RPl	E3 ubiquitin-protein ligase TRIM39 (	K12015 tri	RNAse P, Rpr2/Rpp21
evm.model.HIC_ASM	tripartite mot	Tripartite motif-containing protein 5	K10648 tri	GO:0008270
evm.model.HIC_ASM	PREDICTED	E3 ubiquitin-protein ligase TRIM68 (	K12033 tri	Butyrophylin-like, SPI
evm.model.HIC_ASM	PREDICTED	Putative tripartite motif-containing pı	K12037 tri	SPRY-associated
evm.model.HIC_ASM	PREDICTED	Tripartite motif-containing protein 6	K11999 tri	Butyrophylin-like, SPI
evm.model.HIC_ASM	PREDICTED	Tripartite motif-containing protein 64	K12019 tri	6/2/2020
evm.model.HIC_ASM	tripartite mot	Tripartite motif-containing protein 34	K11999 tri	Zinc finger, RING-typ
evm.model.HIC_ASM	probable E3	Probable E3 ubiquitin-protein ligase	K12038 tri	6/2/2020
evm.model.HIC_ASM	LOW QUAL	E3 ubiquitin-protein ligase TRIM22 (	K11999 tri	6/2/2020
evm.model.HIC_ASM	PREDICTED	Putative tripartite motif-containing pı	K12037 tri	GO:0008270
evm.model.HIC_ASM	E3 ubiquitin-	E3 ubiquitin-protein ligase TRIM11 (	K10650 tri	6/2/2020
evm.model.HIC_ASM	PREDICTED	Probable E3 ubiquitin-protein ligase	K12039 tri	GO:0005515
evm.model.HIC_ASM	putative tripa	Putative tripartite motif-containing pı	K12037 tri	GO:0005515
evm.model.HIC_ASM	PREDICTED	Myosin-8 OS=Canis lupus familiaris	K10352 my	GO:0003774|GO:0005
evm.model.HIC_ASM	PREDICTED	Myosin-2 OS=Canis lupus familiaris	K10352 my	6/2/2020
evm.model.HIC_ASM	myosin-16 [	Myosin-16 OS=Canis lupus familiari	K17751 my	GO:0003774|GO:0005
evm.model.HIC_ASM	Keratin, type	Keratin, type I cytoskeletal 39 OS=H	K07604 ty	6/2/2020
evm.model.HIC_ASM	CMRF35-lik	CMRF35-like molecule 6 OS=Homo	K06719 CI	Immunoglobulin subty
evm.model.HIC_ASM	CMRF35-lik	CMRF35-like molecule 8 OS=Homo	K06719 CI	Immunoglobulin V-set
evm.model.HIC_ASM	CMRF35-lik	CMRF35-like molecule 1 OS=Homo	K20395 C(	6/2/2020
evm.model.HIC_ASM	PREDICTED	CMRF35-like molecule 7 OS=Mus m	K20395 C(	6/2/2020
evm.model.HIC_ASM	Hypothetical	CMRF35-like molecule 7 OS=Homo	K20395 C(	6/2/2020

evm.model.HIC_ASM	CMRF35-lik	Protein CD300H OS=Homo sapiens (	K06719	CI	6/2/2020
evm.model.HIC_ASM	PREDICTED	CMRF35-like molecule 1 OS=Homo	K20395	C(	6/2/2020
evm.model.HIC_ASM	olfactory rec	Olfactory receptor 4C15 OS=Homo s	K04257	ol	6/2/2020
evm.model.CTG_174	olfactory rec	Olfactory receptor 4C15 OS=Homo s	K04257	ol	GO:0004930|GO:0007
evm.model.HIC_ASM	olfactory rec	Olfactory receptor 4C6 OS=Homo sa	K04257	ol	6/2/2020
evm.model.HIC_ASM	olfactory rec	Olfactory receptor 4C6 OS=Homo sa	K04257	ol	6/2/2020
evm.model.HIC_ASM	olfactory rec	Olfactory receptor 4C6 OS=Homo sa	K04257	ol	GO:0004984|GO:0007
evm.model.HIC_ASM	olfactory rec	Olfactory receptor 4C6 OS=Homo sa	K04257	ol	6/2/2020
evm.model.HIC_ASM	olfactory rec	Olfactory receptor 4C6 OS=Homo sa	K04257	ol	6/2/2020
evm.model.HIC_ASM	olfactory rec	Olfactory receptor 51H1 OS=Homo s	K04257	ol	6/2/2020
evm.model.HIC_ASM	olfactory rec	Olfactory receptor 51I2 OS=Homo sa	K04257	ol	GO:0016021
evm.model.HIC_ASM	PREDICTED	Olfactory receptor 51A7 OS=Homo s	K04257	ol	GO:0004930|GO:0007
evm.model.HIC_ASM	olfactory rec	Olfactory receptor 51I2 OS=Homo sa	K04257	ol	6/2/2020
evm.model.HIC_ASM	PREDICTED	Olfactory receptor 51A7 OS=Homo s	K04257	ol	GO:0004984|GO:0007
evm.model.HIC_ASM	uncharacteriz	Histone H4 OS=Xenopus tropicalis C	K11254	his	6/2/2020
evm.model.HIC_ASM	uncharacteriz	Histone H4 OS=Xenopus tropicalis C	K11254	his	6/2/2020
evm.model.CTG_111	zinc finger pı	Zinc finger protein 484 OS=Homo sa	K09228	KI	GO:0003676
evm.model.HIC_ASM	PREDICTED	DnaJ homolog subfamily B member (	K09512	Dı	DnaJ domain
evm.model.HIC_ASM	PREDICTED	Gamma-crystallin F OS=Bos taurus (	K16810	TI	Uncharacterized protei
evm.model.HIC_ASM	PREDICTED	Gamma-crystallin S OS=Canis lupus	K16810	TI	6/2/2020
evm.model.HIC_ASM	PREDICTED	Zinc finger protein 213 OS=Homo sa	K09229	KI	GO:0003676
evm.model.HIC_ASM	zinc finger pı	Zinc finger protein 174 OS=Homo sa	K09230	S(	GO:0003700|GO:0006
evm.model.HIC_ASM	zinc finger aı	Zinc finger protein with KRAB and S	K09229	KI	6/2/2020
evm.model.HIC_ASM	zinc finger pı	Zinc finger protein 444 OS=Homo sa	K09230	S(	GO:0003676
evm.model.HIC_ASM	zinc finger pı	Zinc finger and SCAN domain-contai	K09230	S(	GO:0003676
evm.model.HIC_ASM	udp-glucuror	UDP-glucuronosyltransferase 3A1 O	K00699	glı	GO:0016758
evm.model.HIC_ASM	PREDICTED	2-hydroxyacylsphingosine 1-beta-gal	K04628	ce	6/2/2020
evm.model.HIC_ASM	PREDICTED	Immunoglobulin lambda variable 4-3	K06553	pr	6/2/2020
evm.model.HIC_ASM	PREDICTED	Immunoglobulin kappa variable 2-30	K06553	pr	Immunoglobulin V-set
evm.model.HIC_ASM	immunoglobı	Immunoglobulin kappa variable 2D-2	K06553	pr	6/2/2020
evm.model.HIC_ASM	zinc finger M	Zinc finger MYM-type protein 4 OS=	--		6/2/2020
evm.model.HIC_ASM	antigen-prese	Antigen-presenting glycoprotein CD1	K06448	T-	6/2/2020
evm.model.HIC_ASM	PREDICTED	Interferon kappa OS=Homo sapiens (	K05441	int	6/2/2020
evm.model.HIC_ASM	PREDICTED	Interferon beta OS=Felis catus OX=9	K05415	int	GO:0005126|GO:0005
evm.model.HIC_ASM	histone H2B	Histone H2B type 1-A OS=Rattus no	K11252	his	GO:0000786|GO:0003
evm.model.HIC_ASM	hypothetical	Histone H2B type 3-B OS=Mus musc	K11252	his	GO:0000786|GO:0003
evm.model.HIC_ASM	histone H2B	Histone H2B type 2-F OS=Homo sap	K11252	his	GO:0000786|GO:0003
evm.model.HIC_ASM	PREDICTED	Histone H2B type 1-A OS=Rattus no	K11252	his	GO:0000786|GO:0003
evm.model.HIC_ASM	PREDICTED	Histone H2B type 1-A OS=Rattus no	K11252	his	GO:0000786|GO:0003
evm.model.HIC_ASM	PREDICTED	Histone H2B subacrosomal variant O	K11252	his	GO:0000786|GO:0003
evm.model.HIC_ASM	PREDICTED	Histone H2B type 1-A OS=Mus musc	K11252	his	GO:0000786|GO:0003
evm.model.HIC_ASM	PREDICTED	Zinc finger protein 333 OS=Homo sa	K09228	KI	GO:0003676
evm.model.HIC_ASM	PREDICTED	Zinc finger protein 585A OS=Homo :	K09228	KI	GO:0003676
evm.model.HIC_ASM	histone-like,	Histone H3.3 OS=Xenopus tropicalis	K11253	his	GO:0000786|GO:0003

evm.model.HIC_ASM	uncharacteriz	Histone H3 OS=Urechis caupo OX=(	K11253 his	GO:0000786|GO:0003
evm.model.HIC_ASM	histone H3.3-	Histone H3.3-like type 2 OS=Caenor	K11253 his	6/2/2020
evm.model.HIC_ASM	PREDICTED	Zinc finger protein 304 OS=Homo sa	K09228 KI	GO:0003676
evm.model.HIC_ASM	LOW QUAL	Zinc finger protein 772 OS=Homo sa	K09228 KI	GO:0003676
evm.model.HIC_ASM	uncharacteriz	Zinc finger protein 17 OS=Homo sap	K09228 KI	GO:0003676
evm.model.HIC_ASM	PREDICTED	Zinc finger protein 140 OS=Homo sa	K09228 KI	6/2/2020
evm.model.HIC_ASM	PREDICTED	Zinc finger protein 181 OS=Bos taur	K09228 KI	GO:0003676
evm.model.HIC_ASM	olfactory rec	Olfactory receptor 51B2 OS=Homo s	K04257 ol	GO:0004930|GO:0007
evm.model.HIC_ASM	olfactory rec	Olfactory receptor 51B6 OS=Homo s	K04257 ol	6/2/2020
evm.model.HIC_ASM	histone H2A-	Histone H2A type 1-A OS=Homo sap	K11251 his	6/2/2020
evm.model.HIC_ASM	histone H2A	Histone H2A type 1-A OS=Homo sap	K11251 his	GO:0000786|GO:0003
evm.model.HIC_ASM	Histone H2A	Histone H2A type 3 OS=Rattus norve	K11251 his	GO:0000786|GO:0003
evm.model.HIC_ASM	calcium-bind	Calcium-binding protein 2 OS=Mus r	K02183 ca	GO:0005509
evm.model.HIC_ASM	PREDICTED	Zinc finger protein 215 OS=Homo sa	K09229 KI	GO:0003700|GO:0006
evm.model.HIC_ASM	zinc finger in	Zinc finger protein 215 OS=Pongo al	K09229 KI	6/2/2020
evm.model.HIC_ASM	PREDICTED	Keratin, type II cytoskeletal 71 OS=E	K07605 ty	6/2/2020
evm.model.HIC_ASM	type ii cytosk	Keratin, type II cytoskeletal 78 OS=E	K07605 ty	Intermediate filament,
evm.model.HIC_ASM	PREDICTED	Keratin, type II cytoskeletal 6C OS=I	K07605 ty	Intermediate filament,
evm.model.HIC_ASM	hypothetical	Zinc finger protein 710 OS=Homo sa	K09228 KI	6/2/2020
evm.model.HIC_ASM	PREDICTED	Zinc finger protein 550 OS=Homo sa	K09228 KI	GO:0003676
evm.model.HIC_ASM	serine protea	Serine protease hepsin OS=Homo sap	K08665 he	GO:0004252|GO:0006
evm.model.HIC_ASM	PREDICTED	Serine protease 27 OS=Homo sapien	K09628 se	GO:0004252|GO:0006
evm.model.HIC_ASM	serine protea	Serine protease 27 OS=Homo sapien	K09625 tes	GO:0004252|GO:0006
evm.model.HIC_ASM	PREDICTED	Prostasin OS=Rattus norvegicus OX=	K08664 pr	GO:0004252|GO:0006
evm.model.HIC_ASM	transmembra	Transmembrane protease serine 13 O	K09643 tra	GO:0004252|GO:0006
evm.model.HIC_ASM	Hypothetical	Putative serine protease 47 OS=Hom	K09628 se	GO:0004252|GO:0006
evm.model.HIC_ASM	serine protea	Serine protease 40 OS=Rattus norveg	K02725 20	GO:0004252|GO:0006
evm.model.HIC_ASM	PREDICTED	Brain-specific serine protease 4 OS=l	K09626 br	GO:0004252|GO:0006
evm.model.HIC_ASM	transmembra	Serine protease 33 OS=Homo sapien	K09629 se	GO:0004252|GO:0006
evm.model.HIC_ASM	PREDICTED	Probable threonine protease PRSS50	K09628 se	GO:0004252|GO:0006
evm.model.HIC_ASM	testisin isofo	Testisin OS=Mus musculus OX=100	K09625 tes	6/2/2020
evm.model.HIC_ASM	serine protea	Serine protease 38 OS=Homo sapien	K09628 se	GO:0004252|GO:0006
evm.model.HIC_ASM	tryptase-like	Tryptase OS=Canis lupus familiaris (	K01340 try	GO:0004252|GO:0006
evm.model.HIC_ASM	serine protea	Serine protease 48 OS=Homo sapien	K09625 tes	GO:0004252|GO:0006
evm.model.HIC_ASM	serine protea	Serine protease 29 OS=Mus musculu	K01340 try	GO:0004252|GO:0006
evm.model.HIC_ASM	PREDICTED	Zinc finger protein 133 OS=Homo sa	K09228 KI	GO:0003676|GO:0006
evm.model.HIC_ASM	PREDICTED	C-type lectin domain family 4 membe	K10057 C-	C-type lectin-like
evm.model.HIC_ASM	PREDICTED	C-type lectin domain family 4 membe	K10061 C-	C-type lectin-like
evm.model.HIC_ASM	PREDICTED	CD209 antigen-like protein 2 OS=Ma	K06563 C-	6/2/2020
evm.model.HIC_ASM	PREDICTED	C-type lectin domain family 6 membe	K17514 C-	C-type lectin-like
evm.model.HIC_ASM	C-type lectin	C-type lectin domain family 4 membe	K10059 C-	C-type lectin-like
evm.model.HIC_ASM	transcription	Transcription elongation factor A prc	--	6/2/2020
evm.model.HIC_ASM	transcription	Transcription elongation factor A prc	--	Transcription elongatio
evm.model.HIC_ASM	Keratin, type	Keratin, type I cytoskeletal 25 OS=B	K07604 ty	Intermediate filament,

evm.model.HIC_ASM	PREDICTED	Keratin, type I cytoskeletal 14 OS=H	K07604 ty	Intermediate filament,
evm.model.HIC_ASM	PREDICTED	Keratin, type I cytoskeletal 12 OS=H	K07604 ty	Intermediate filament,
evm.model.HIC_ASM	PREDICTED	Putative olfactory receptor 56B2 OS=	K04257 ol	GO:0004984|GO:0007
evm.model.HIC_ASM	olfactory rec	Olfactory receptor 688 OS=Mus mus	K04257 ol	GO:0004984|GO:0007
evm.model.HIC_ASM	PREDICTED	Olfactory receptor 688 OS=Mus mus	K04257 ol	6/2/2020
evm.model.HIC_ASM	putative olfa	Putative olfactory receptor 56B2 OS=	K04257 ol	GO:0004984|GO:0007
evm.model.HIC_ASM	olfactory rec	Olfactory receptor 688 OS=Mus mus	K04257 ol	6/2/2020
evm.model.HIC_ASM	PREDICTED	Putative olfactory receptor 56B2 OS=	K04257 ol	GO:0004984|GO:0007
evm.model.HIC_ASM	zinc finger p	Zinc finger protein 10 OS=Homo sap	K09228 K	GO:0003676
evm.model.HIC_ASM	zinc finger p	Zinc finger protein 572 OS=Bos taur	K09228 K	GO:0003676
evm.model.HIC_ASM	PREDICTED	Zinc finger protein with KRAB and S	K09229 K	6/2/2020
evm.model.HIC_ASM	zinc finger X	Zinc finger X-chromosomal protein (	K09228 K	GO:0003676
evm.model.HIC_ASM	PREDICTED	Zinc finger protein 214 OS=Homo sa	K09228 K	6/2/2020
evm.model.HIC_ASM	zinc finger p	Zinc finger protein 229 OS=Homo sa	K09228 K	GO:0003676
evm.model.HIC_ASM	prostaglandi	Prostaglandin-H2 D-isomerase OS=F	K01830 pr	6/2/2020
evm.model.HIC_ASM	PREDICTED	Taste receptor type 2 member 38 OS=	K08474 ta	GO:0004930|GO:0007
evm.model.HIC_ASM	PREDICTED	Taste receptor type 2 member 3 OS=	K08474 ta	GO:0016021
evm.model.HIC_ASM	LOW QUAL	Taste receptor type 2 member 5 OS=	K08474 ta	6/2/2020
evm.model.HIC_ASM	taste recepto	Taste receptor type 2 member 7 OS=	K08474 ta	GO:0004930|GO:0007
evm.model.HIC_ASM	taste recepto	Taste receptor type 2 member 42 OS=	K08474 ta	6/2/2020
evm.model.HIC_ASM	PREDICTED	Taste receptor type 2 member 41 OS=	K08474 ta	GO:0004930|GO:0007
evm.model.HIC_ASM	zinc finger p	Zinc finger protein 850 OS=Homo sa	K09228 K	GO:0003676
evm.model.HIC_ASM	zinc finger p	Zinc finger protein 668 OS=Bos taur	K09228 K	GO:0003676
evm.model.HIC_ASM	LOW QUAL	Zinc finger protein 850 OS=Homo sa	K09228 K	6/2/2020
evm.model.HIC_ASM	PREDICTED	60S ribosomal protein L17 OS=Hom	K02880 la	6/2/2020
evm.model.HIC_ASM	olfactory rec	Olfactory receptor 52L1 OS=Homo s	K04257 ol	GO:0004984|GO:0007
evm.model.HIC_ASM	olfactory rec	Olfactory receptor 52E5 OS=Homo s	K04257 ol	GO:0004984|GO:0007
evm.model.HIC_ASM	olfactory rec	Olfactory receptor 52A5 OS=Homo s	K04257 ol	6/2/2020
evm.model.HIC_ASM	olfactory rec	Olfactory receptor 52M1 OS=Homo	K04257 ol	6/2/2020
evm.model.HIC_ASM	olfactory rec	Olfactory receptor 52A5 OS=Homo s	K04257 ol	6/2/2020
evm.model.HIC_ASM	PREDICTED	Olfactory receptor 52A1 OS=Homo s	K04257 ol	6/2/2020
evm.model.HIC_ASM	olfactory rec	Olfactory receptor 52R1 OS=Homo s	K04257 ol	6/2/2020
evm.model.HIC_ASM	olfactory rec	Olfactory receptor 52W1 OS=Homo	K04257 ol	GO:0004984|GO:0007
evm.model.HIC_ASM	putative olfa	Putative olfactory receptor 52P1 OS=	K04257 ol	6/2/2020
evm.model.HIC_ASM	olfactory rec	Olfactory receptor 52L1 OS=Homo s	K04257 ol	GO:0004930|GO:0007
evm.model.HIC_ASM	PREDICTED	Olfactory receptor 52A1 OS=Homo s	K04257 ol	6/2/2020
evm.model.HIC_ASM	PREDICTED	Histone H1x OS=Homo sapiens OX=	K11275 his	6/2/2020
evm.model.HIC_ASM	PREDICTED	Olfactory receptor 476 OS=Mus mus	K04257 ol	GO:0004984|GO:0007
evm.model.HIC_ASM	GTPase IMA	GTPase IMAP family member 1 OS=	K15261 po	6/2/2020
evm.model.HIC_ASM	GTPase IMA	GTPase IMAP family member 6 OS=	K09228 K	GO:0005525
evm.model.HIC_ASM	GTPase IMA	GTPase IMAP family member 4 OS=	K18626 tri	GO:0005525
evm.model.HIC_ASM	GTPase IMA	GTPase IMAP family member 2 OS=	K09228 K	6/2/2020
evm.model.HIC_ASM	GTPase IMA	GTPase IMAP family member 7 OS=	K00573 pr	6/2/2020
evm.model.HIC_ASM	carcinoembry	Carcinoembryonic antigen-related ce	K06499 ca	Immunoglobulin subty

evm.model.HIC_ASM	PREDICTED	Carcinoembryonic antigen-related ce	K06499	ca	6/2/2020
evm.model.HIC_ASM	PREDICTED	Tigger transposable element-derived	K09228	KI	HTH CenpB-type DNA
evm.model.HIC_ASM	hCG204353	60S ribosomal protein L39 OS=Rattu	K02924	la	GO:0003735|GO:0005
evm.model.HIC_ASM	putative olfa	Putative olfactory receptor 52P1 OS=	K04257	ol	GO:0016021
evm.model.HIC_ASM	olfactory rec	Olfactory receptor 52E2 OS=Homo s	K04257	ol	6/2/2020
evm.model.HIC_ASM	PREDICTED	Olfactory receptor 52E2 OS=Homo s	K04257	ol	GO:0004984|GO:0007
evm.model.HIC_ASM	Olfactory rec	Olfactory receptor 52D1 OS=Homo s	K04257	ol	GO:0016021
evm.model.HIC_ASM	olfactory rec	Olfactory receptor 52E5 OS=Homo s	K04257	ol	6/2/2020
evm.model.HIC_ASM	PREDICTED	Cytochrome c oxidase subunit 6B2 O	K02267	cy	6/2/2020
evm.model.HIC_ASM	PREDICTED	Olfactory receptor 10R2 OS=Homo s	K04257	ol	GO:0004930|GO:0007
evm.model.HIC_ASM	PREDICTED	Olfactory receptor 10T2 OS=Homo s	K04257	ol	GO:0016021
evm.model.HIC_ASM	olfactory rec	Olfactory receptor 10V1 OS=Homo s	K04257	ol	6/2/2020
evm.model.HIC_ASM	PREDICTED	Olfactory receptor 10Q1 OS=Homo s	K04257	ol	6/2/2020
evm.model.HIC_ASM	PREDICTED	Folate receptor alpha OS=Mus muscu	K13649	fo	Folate receptor-like
evm.model.HIC_ASM	sperm-egg fu	Sperm-egg fusion protein Juno OS=H	K13649	fo	Folate receptor-like
evm.model.HIC_ASM	olfactory rec	Olfactory receptor-like protein OLF3	K04257	ol	GO:0016021
evm.model.HIC_ASM	PREDICTED	Olfactory receptor-like protein OLF3	K04257	ol	6/2/2020
evm.model.HIC_ASM	PREDICTED	Olfactory receptor-like protein OLF3	K04257	ol	GO:0004984|GO:0007
evm.model.HIC_ASM	PREDICTED	Olfactory receptor-like protein OLF3	K04257	ol	6/2/2020
evm.model.HIC_ASM	PREDICTED	Olfactory receptor-like protein OLF3	K04257	ol	GO:0004984|GO:0007
evm.model.HIC_ASM	PREDICTED	Olfactory receptor-like protein OLF3	K04257	ol	GO:0004984|GO:0007
evm.model.HIC_ASM	olfactory rec	Olfactory receptor-like protein OLF3	K04257	ol	GO:0004930|GO:0007
evm.model.HIC_ASM	PREDICTED	Olfactory receptor-like protein OLF3	K04257	ol	GO:0004984|GO:0007
evm.model.HIC_ASM	PREDICTED	Olfactory receptor-like protein OLF3	K04257	ol	GO:0004984|GO:0007
evm.model.HIC_ASM	olfactory rec	Olfactory receptor-like protein OLF3	K04257	ol	GO:0016021
evm.model.CTG_160	transmembra	Transmembrane protease serine 11B-	K09751	tra	6/2/2020
evm.model.HIC_ASM	transmembra	Transmembrane protease serine 11A	K09750	tra	SEA domain
evm.model.HIC_ASM	Transmembra	Transmembrane protease serine 11F (	K09752	tra	SEA domain
evm.model.HIC_ASM	hypothetical	Transmembrane protease serine 11E (	K09642	tra	SEA domain
evm.model.HIC_ASM	transmembra	Transmembrane protease serine 11D	K09641	tra	GO:0004252|GO:0006
evm.model.HIC_ASM	keratin-assoc	Keratin-associated protein 13-1 OS=I	--		Keratin-associated pro
evm.model.HIC_ASM	PREDICTED	Anionic trypsin OS=Canis lupus fam	K01312	try	GO:0004252|GO:0006
evm.model.HIC_ASM	PREDICTED	Kallikrein-1 OS=Papio hamadryas O	K01325	tis	GO:0004252|GO:0006
evm.model.HIC_ASM	cationic tryp	Cationic trypsin OS=Canis lupus fam	K01312	try	GO:0004252|GO:0006
evm.model.HIC_ASM	PREDICTED	Kallikrein-15 OS=Homo sapiens OX	K09623	ka	GO:0004252|GO:0006
evm.model.HIC_ASM	kallikrein 10	Kallikrein-5 OS=Homo sapiens OX=	K09617	ka	GO:0004252|GO:0006
evm.model.HIC_ASM	PREDICTED	Kallikrein-4 OS=Homo sapiens OX=	K08666	ka	6/2/2020
evm.model.HIC_ASM	kallikrein-10	Kallikrein-10 OS=Homo sapiens OX	K09619	ka	GO:0004252|GO:0006
evm.model.HIC_ASM	hypothetical	Hemoglobin subunit epsilon OS=Dau	K13825	he	GO:0020037
evm.model.HIC_ASM	PREDICTED	--	--		6/2/2020
evm.model.HIC_ASM	PREDICTED	--	--		6/2/2020
evm.model.HIC_ASM	NKG2-D typ	NKG2-D type II integral membrane p	K06728	kil	C-type lectin-like
evm.model.HIC_ASM	NKG2-A/NK	NKG2-A/NKG2-B type II integral m	K06541	kil	6/2/2020
evm.model.HIC_ASM	NKG2-A/NK	NKG2-A/NKG2-B type II integral m	K06541	kil	C-type lectin-like

evm.model.CTG_710	olfactory rec	Olfactory receptor 2T33 OS=Homo s	K04257 ol	6/2/2020
evm.model.HIC_ASM	PREDICTEI	Olfactory receptor 2Z1 OS=Homo sa	K04257 ol	6/2/2020
evm.model.CTG_299	olfactory rec	Olfactory receptor 2AJ1 OS=Homo s	K04257 ol	Olfactory receptor 2AJ
evm.model.CTG_710	PREDICTEI	Olfactory receptor 2M3 OS=Homo sa	K04257 ol	GO:0004984|GO:0007
evm.model.CTG_381	olfactory rec	Olfactory receptor 2T7 OS=Homo sa	K04257 ol	6/2/2020
evm.model.CTG_120	olfactory rec	Olfactory receptor 2AJ1 OS=Homo s	K04257 ol	Olfactory receptor 2AJ
evm.model.HIC_ASM	cytochrome I	Cytochrome P450 3A12 OS=Canis lu	K07424 cy	6/2/2020
evm.model.HIC_ASM	PREDICTEI	Keratin, type II cytoskeletal 7 OS=Bo	K07605 ty	6/2/2020
evm.model.HIC_ASM	hypothetical	Keratin, type II cytoskeletal 6A OS=I	K07605 ty	Intermediate filament,
evm.model.HIC_ASM	PREDICTEI	Keratin, type II microfibrillar, compo	K07605 ty	Intermediate filament,
evm.model.HIC_ASM	Keratin, type	Keratin, type II cuticular Hb6 OS=M	K07605 ty	Intermediate filament,
evm.model.HIC_ASM	Keratin, type	Keratin, type II cuticular Hb5 OS=M	K07605 ty	6/2/2020
evm.model.HIC_ASM	PREDICTEI	Keratin, type II cuticular Hb1 OS=Ho	K07605 ty	Keratin type II head
evm.model.HIC_ASM	PREDICTEI	Placenta-specific gene 8 protein OS=	--	PLAC8 motif-containi
evm.model.HIC_ASM	PREDICTEI	Cornifelin OS=Mus musculus OX=1(	--	6/2/2020
evm.model.HIC_ASM	Olfactory rec	Olfactory receptor 6B3 OS=Homo sa	K04257 ol	6/2/2020
evm.model.HIC_ASM	PREDICTEI	Leukocyte immunoglobulin-like rece	K06512 leu	6/2/2020
evm.model.HIC_ASM	leukocyte im	Leukocyte immunoglobulin-like rece	K06512 leu	GO:0019843
evm.model.HIC_ASM	PREDICTEI	Platelet glycoprotein VI OS=Homo sa	K06264 pla	6/2/2020
evm.model.HIC_ASM	immunoglobu	Immunoglobulin alpha Fc receptor O	K06513 CI	6/2/2020
evm.model.HIC_ASM	leukocyte im	Leukocyte immunoglobulin-like rece	K06512 leu	Immunoglobulin-like d
evm.model.HIC_ASM	PREDICTEI	Leukocyte immunoglobulin-like rece	K06512 leu	Immunoglobulin subty
evm.model.HIC_ASM	leukocyte im	Leukocyte immunoglobulin-like rece	K06512 leu	6/2/2020
evm.model.HIC_ASM	PREDICTEI	Leukocyte immunoglobulin-like rece	K07980 kil	Immunoglobulin subty
evm.model.CTG_149	olfactory rec	Olfactory receptor 4P4 OS=Homo sa	K04257 ol	6/2/2020
evm.model.CTG_184	LOW QUAL	Olfactory receptor 4P4 OS=Homo sa	K04257 ol	GO:0004984|GO:0007
evm.model.HIC_ASM	olfactory rec	Olfactory receptor 4P4 OS=Homo sa	K04257 ol	GO:0004984|GO:0007
evm.model.HIC_ASM	PREDICTEI	Olfactory receptor 4S2 OS=Homo sa	K04257 ol	6/2/2020
evm.model.CTG_158	PREDICTEI	Olfactory receptor 4S2 OS=Homo sa	K04257 ol	GO:0004984|GO:0007
evm.model.CTG_138	PREDICTEI	Olfactory receptor 4S2 OS=Homo sa	K04257 ol	GO:0016021
evm.model.CTG_382	PREDICTEI	Olfactory receptor 4P4 OS=Homo sa	K04257 ol	GO:0004984|GO:0007
evm.model.CTG_138	olfactory rec	Olfactory receptor 4P4 OS=Homo sa	K04257 ol	6/2/2020
evm.model.CTG_184	PREDICTEI	Olfactory receptor 4P4 OS=Homo sa	K04257 ol	GO:0016021
evm.model.CTG_149	PREDICTEI	Olfactory receptor 4S2 OS=Homo sa	K04257 ol	6/2/2020
evm.model.CTG_649	PREDICTEI	Olfactory receptor 4P4 OS=Homo sa	K04257 ol	GO:0004984|GO:0007
evm.model.CTG_158	olfactory rec	Olfactory receptor 4P4 OS=Homo sa	K04257 ol	6/2/2020
evm.model.HIC_ASM	PREDICTEI	Olfactory receptor 4S2 OS=Homo sa	K04257 ol	GO:0004984|GO:0007
evm.model.CTG_111	PREDICTEI	Olfactory receptor 4P4 OS=Homo sa	K04257 ol	GO:0004984|GO:0007
evm.model.CTG_158	olfactory rec	Olfactory receptor 4P4 OS=Homo sa	K04257 ol	6/2/2020
evm.model.CTG_254	PREDICTEI	Olfactory receptor 4P4 OS=Homo sa	K04257 ol	GO:0004984|GO:0007
evm.model.CTG_724	olfactory rec	Olfactory receptor 4P4 OS=Homo sa	K04257 ol	GO:0004984|GO:0007
evm.model.CTG_104	olfactory rec	Olfactory receptor 1D2 OS=Pongo py	K04257 ol	GO:0004984|GO:0007
evm.model.CTG_160	PREDICTEI	Olfactory receptor 1D2 OS=Homo sa	K04257 ol	GO:0004930|GO:0007
evm.model.CTG_175	olfactory rec	Olfactory receptor 1D2 OS=Pongo py	K04257 ol	GO:0004984|GO:0007

evm.model.HIC_ASM	PREDICTEI	Olfactory receptor 1D2 OS=Pongo py	K04257 ol	GO:0016021
evm.model.CTG_140	PREDICTEI	Olfactory receptor 1D2 OS=Pongo py	K04257 ol	6/2/2020
evm.model.CTG_104	PREDICTEI	Olfactory receptor 1D2 OS=Pongo py	K04257 ol	GO:0004984|GO:0007
evm.model.CTG_408	PREDICTEI	Olfactory receptor 1D2 OS=Homo sa	K04257 ol	GO:0004984|GO:0007
evm.model.CTG_645	PREDICTEI	Olfactory receptor 1D2 OS=Homo sa	K04257 ol	GO:0004930|GO:0007
evm.model.HIC_ASM	unnamed pro	Zinc finger protein 358 OS=Homo sa	K09228 Kl	GO:0003676
evm.model.HIC_ASM	olfactory rec	Olfactory receptor 52B4 OS=Homo s	K04257 ol	6/2/2020
evm.model.HIC_ASM	PREDICTEI	Olfactory receptor 52D1 OS=Homo s	K04257 ol	GO:0016021
evm.model.HIC_ASM	olfactory rec	Olfactory receptor 52B4 OS=Homo s	K04257 ol	GO:0004930|GO:0007
evm.model.HIC_ASM	PREDICTEI	Olfactory receptor 52B2 OS=Homo s	K04257 ol	GO:0004984|GO:0007
evm.model.HIC_ASM	PREDICTEI	Olfactory receptor 6C6 OS=Homo sa	K04257 ol	GO:0004984|GO:0007
evm.model.HIC_ASM	PREDICTEI	Olfactory receptor 6C6 OS=Homo sa	K04257 ol	GO:0004984|GO:0007
evm.model.HIC_ASM	olfactory rec	Olfactory receptor 6C6 OS=Homo sa	K04257 ol	6/2/2020
evm.model.HIC_ASM	PREDICTEI	Olfactory receptor 6C6 OS=Homo sa	K04257 ol	GO:0004984|GO:0007

186|GO:0016021

in, alpha1 alpha2 domains

en recognition-like

355

RY domain

subunit

RY domain

RY domain

e

524|GO:0016459

524|GO:0016459

pe
domain

186|GO:0016021

186|GO:0016021

186|GO:0016021

186|GO:0016021

n C2orf80

355

domain

576|GO:0006952
677
677
677
677
677
677
677

677

677

186|GO:0016021

677
677|GO:0005634

355

rod domain
rod domain

508
508
508
508
508
508
508
508
508
508

508
508
508
508
355

on factor A-like/Brain expressed X-linked
rod domain

rod domain
rod domain
186|GO:0016021
186|GO:0016021

186|GO:0016021

186|GO:0016021

186|GO:0016021|GO:0050909

186|GO:0016021|GO:0050909

186|GO:0016021|GO:0050909

186|GO:0016021
186|GO:0016021

186|GO:0016021

186|GO:0016021

186|GO:0016021

pe

A-binding domain
840|GO:0006412

186|GO:0016021

186|GO:0016021

186|GO:0016021

186|GO:0016021
186|GO:0016021
186|GO:0016021
186|GO:0016021
186|GO:0016021

508
tein, PMG type
508
508
508
508
508

508

1
186|GO:0016021

1

rod domain
rod domain
rod domain

ng protein

lomain
pe

pe

186|GO:0016021
186|GO:0016021

186|GO:0016021

186|GO:0016021

186|GO:0016021

186|GO:0016021
186|GO:0016021

186|GO:0016021
186|GO:0016021
186|GO:0016021
186|GO:0016021
186|GO:0016021

186|GO:0016021
186|GO:0016021
186|GO:0016021

186|GO:0016021
186|GO:0016021
186|GO:0016021
186|GO:0016021

186|GO:0016021

Supplementary Table S9 Positively selected genes in the genome of masked palm civet.

Gene ID	NR_description	Swissprot_description	KEGG_anno
evm.model.HIC_ASM_14.1140	polypeptide N-acetylga	Polypeptide N-acetylga	K00710 polypeptide N
evm.model.HIC_ASM_1.1212	small integral membrai	Small integral membra	--
evm.model.HIC_ASM_18.1205.1	PREDICTED: enhance	Enhancer of mRNA-de	K12616 enhancer of m
evm.model.HIC_ASM_8.348	A disintegrin and meta	A disintegrin and meta	K08623 a disintegrin a
evm.model.HIC_ASM_20.1190	ankyrin repeat and SO(	Ankyrin repeat and SO	K10338 ankyrin repeat
evm.model.HIC_ASM_4.1216	calpain-3 isoform X1 |	Calpain-3 OS=Mus mu	K08573 calpain-3 [EC:
evm.model.HIC_ASM_11.1007	cyclin-dependent kinas	Cyclin-dependent-like	K02090 cyclin-depend
evm.model.HIC_ASM_6.974	acyl-protein thioesteras	Acyl-protein thioestera	K06128 lysophospholi
evm.model.HIC_ASM_19.1251	transcription cofactor v	Transcription cofactor	--
evm.model.HIC_ASM_2.1222.1	enoyl-CoA delta isome	Enoyl-CoA delta isome	K13238 Delta3-Delta2-
evm.model.HIC_ASM_4.358.3	maleylacetoacetate isoı	Maleylacetoacetate iso	K01800 maleylacetoac
evm.model.HIC_ASM_5.1068	LOW QUALITY PRO'	Desert hedgehog protei	K11990 desert hedgeho
evm.model.HIC_ASM_9.1162	PREDICTED: protein 1	Protein tyrosine phospl	K18078 protein tyrosin
evm.model.HIC_ASM_13.165.1(	phosphatidate phospha	Phosphatidate phospha	K15728 phosphatidate
evm.model.HIC_ASM_12.297	E3 ubiquitin-protein lig	E3 ubiquitin-protein lig	K17821 RING finger p
evm.model.HIC_ASM_17.1054.1	LOW QUALITY PRO'	Kinesin-like protein KI	K10404 kinesin family
evm.model.HIC_ASM_3.1333	SNX24 isoform 7 [Par	Sorting nexin-24 OS=F	K17941 sorting nexin-2
evm.model.HIC_ASM_20.135	PREDICTED: protein 1	Protein KIAA0100 OS	K17529 uncharacterize
evm.model.HIC_ASM_6.998	uncharacterized protein	Uncharacterized protei	--
evm.model.HIC_ASM_0.1011_ev	PREDICTED: serine-ri	Serine-rich coiled-coil	--
evm.model.HIC_ASM_16.315	diacylglycerol kinase e	Diacylglycerol kinase ε	K00901 diacylglycerol
evm.model.HIC_ASM_0.1684	alpha-L-iduronidase pr	Alpha-L-iduronidase O	K01217 L-iduronidase
evm.model.HIC_ASM_10.1388	bromodomain testis-sp	Bromodomain testis-sp	K11724 bromodomain
evm.model.HIC_ASM_4.1846	PREDICTED: treslin [	Treslin OS=Homo sapi	--
evm.model.HIC_ASM_18.1558	PREDICTED: LOW Q	Dysbindin domain-con	--
evm.model.HIC_ASM_21.340	PREDICTED: BPI fold	BPI fold-containing far	K05399 lipopolysaccha
evm.model.HIC_ASM_12.124	PREDICTED: cadherin	Cadherin-7 OS=Homo	K06799 cadherin 7, typ
evm.model.HIC_ASM_2.1483	lysine-rich nucleolar pı	Lysine-rich nucleolar p	K04619 G protein-coup
evm.model.HIC_ASM_21.311	interferon regulatory fa	Interferon regulatory fa	K09445 interferon regu
evm.model.HIC_ASM_10.126.3	PREDICTED: DNA fra	DNA fragmentation fac	K02311 DNA fragmen
evm.model.HIC_ASM_8.618.1	PREDICTED: X-ray ra	X-ray radiation resistar	K18808 toll-like recept
evm.model.HIC_ASM_9.985	transient receptor poter	Transient receptor pote	K04978 transient recep
evm.model.HIC_ASM_10.812	collagen alpha-2(VIII)	Collagen alpha-2(VIII)	K23455 collagen, type
evm.model.HIC_ASM_19.158	CUGBP Elav-like fami	CUGBP Elav-like fami	K13207 CUG-BP- and
evm.model.HIC_ASM_11.578.3	acyloxyacyl hydrolase	Acyloxyacyl hydrolase	K01065 acyloxyacyl hy
evm.model.HIC_ASM_19.392	PREDICTED: LOW Q	Retinol dehydrogenase	K11150 retinol dehydro
evm.model.HIC_ASM_1.110	rab effector MyRIP iso	Rab effector MyRIP O	K22237 Rab effector M
evm.model.HIC_ASM_2.1162	coiled-coil domain-con	Coiled-coil domain-coı	--
evm.model.HIC_ASM_17.646	PREDICTED: unchara	Carboxyl-terminal PDZ	K16513 carboxyl-termi
evm.model.HIC_ASM_14.444	PREDICTED: tudor dc	Tudor domain-containi	K18405 tudor domain-
evm.model.HIC_ASM_11.200	cadherin-related family	Cadherin-related family	K16503 cadherin-relate
evm.model.HIC_ASM_1.1063_ev	ephrin type-A receptor	Ephrin type-A receptor	K05104 Eph receptor A

evm.model.HIC_ASM_11.295	PREDICTED: GTP-bi	GTP-binding protein 1	K03979	GTPase [EC:3
evm.model.HIC_ASM_11.640	PREDICTED: receptor	Receptor-type tyrosine	K08114	receptor-type t
evm.model.HIC_ASM_11.624	LSM8 homolog, U6 sn	U6 snRNA-associated	K12627	U6 snRNA-as
evm.model.HIC_ASM_19.406	39S ribosomal protein	39S ribosomal protein	K02926	large subunit r
evm.model.HIC_ASM_3.199	F-box/LRR-repeat prot	F-box/LRR-repeat prot	K10273	F-box and leu
evm.model.HIC_ASM_10.1243	retinoid isomerohydrol	Retinoid isomerohydro	K11158	retinoid isome
evm.model.HIC_ASM_0.1539	PREDICTED: limbin i	Limbin OS=Bos taurus	K19608	ellis van creve
evm.model.HIC_ASM_2.2065	equilibrative nucleosid	Equilibrative nucleosid	K03323	solute carrier f
evm.model.HIC_ASM_4.99	coiled-coil domain-con	Coiled-coil domain-co	--	
evm.model.HIC_ASM_3.517	LOW QUALITY PRO	Magnesium transporter	K22733	magnesium tra
evm.model.HIC_ASM_3.1008	PREDICTED: ras-relat	Ras-related protein Ral	K07883	Ras-related pr
evm.model.HIC_ASM_20.877.1	PREDICTED: alpha-N	Alpha-N-acetylgalacto	K06616	alpha-N-acety
evm.model.HIC_ASM_13.537	threonine synthase-like	Threonine synthase-lik	K06037	O-phospho-L-
evm.model.HIC_ASM_5.604	PREDICTED: plasma	Plasma membrane calc	K05850	P-type Ca2+ t
evm.model.HIC_ASM_10.502	PREDICTED: arf-GAI	Arf-GAP with SH3 do	K12488	Arf-GAP with
evm.model.HIC_ASM_16.158	dedicator of cytokinesi	Dedicator of cytokines	K21853	dedicator of c
evm.model.HIC_ASM_3.270	sperm flagellar protein	Sperm flagellar protein	K00939	adenylate kina
evm.model.HIC_ASM_11.135	UPF0415 protein C7or	UPF0415 protein C7or	K02726	20S proteason
evm.model.HIC_ASM_5.1666	OTU domain-containin	OTU domain-containin	K13716	OTU domain-
evm.model.HIC_ASM_6.1363	PREDICTED: androge	Androgen-induced gen	--	
evm.model.HIC_ASM_7.956	PREDICTED: glutama	Glutamate receptor 3 C	K05199	glutamate rece
evm.model.HIC_ASM_7.1070	PREDICTED: probabl	Adhesion G-protein co	K08455	adhesion G-pr
evm.model.HIC_ASM_19.358.2	mucolipin-1 isoform X	Mucolipin-1 OS=Hom	K04992	mucolipin 1 | (
evm.model.HIC_ASM_20.356_e	PREDICTED: B9 dom	B9 domain-containing	K16744	B9 domain-co
evm.model.HIC_ASM_10.284	anf_felca ame: full=na	Natriuretic peptides B	K12335	natriuretic pep
evm.model.HIC_ASM_20.1076	PREDICTED: testis-ex	Testis-expressed protei	--	
evm.model.HIC_ASM_9.244.1	TBC1 domain family n	TBC1 domain family n	K03357	anaphase-pron
evm.model.HIC_ASM_13.919.3	VIP36-like protein isof	VIP36-like protein OS	K10083	lectin, mannos
evm.model.HIC_ASM_12.654	uncharacterized protei	Uncharacterized protei	--	
evm.model.HIC_ASM_5.196	malonyl-CoA-acyl carr	Malonyl-CoA-acyl car	K00645	[acyl-carrier-p
evm.model.HIC_ASM_0.390.3	PREDICTED: dematin	Dematin OS=Pongo ab	K07520	actin-binding
evm.model.HIC_ASM_21.587	solute carrier family 12	Solute carrier family 1	K14427	solute carrier f
evm.model.HIC_ASM_6.1095	autophagy protein 5 is	Autophagy protein 5 O	K08339	autophagy-rela
evm.model.HIC_ASM_19.1066	uncharacterized protei	Uncharacterized protei	--	
evm.model.HIC_ASM_9.512	LOW QUALITY PRO	DOMON domain-conta	K03843	alpha-1,3/alph
evm.model.HIC_ASM_0.1631.5	SH3 domain-binding p	SH3 domain-binding p	K07984	SH3-domain b
evm.model.HIC_ASM_2.173.2	diacylglycerol kinase d	Diacylglycerol kinase	K00901	diacylglycerol
evm.model.HIC_ASM_2.1525.1	protein N-terminal asp	Protein N-terminal asp	K14662	protein N-term
evm.model.HIC_ASM_9.48.3	protein PAXX isoform	Protein PAXX OS=Ho	--	
evm.model.HIC_ASM_10.531	calcipressin-3 isoform	Calcipressin-3 OS=Ho	K17905	calcipression-
evm.model.HIC_ASM_9.891	PREDICTED: death-as	Death-associated prote	K08803	death-associat
evm.model.HIC_ASM_5.1467	PREDICTED: von Wil	von Willebrand factor	K03900	von Willebran
evm.model.HIC_ASM_0.730.2	PREDICTED: ATP-bi	ATP-binding cassette s	K06174	ATP-binding c
evm.model.HIC_ASM_19.262	cation channel sperm-a	Cation channel sperm-	K16895	cation channel

evm.model.HIC_ASM_20.715.2	ethanolamine-phosphat	Ethanolamine-phospha	K00967	ethanolamine-
evm.model.HIC_ASM_18.915.4	vacuolar protein sortin	Vacuolar protein sortin	K18468	vacuolar prote
evm.model.HIC_ASM_19.693	LOW QUALITY PRO'	IQ domain-containing]	--	
evm.model.HIC_ASM_20.1482.2	PREDICTED: protein]	Protein FAM117A OS=	K20407	WD repeat-co
evm.model.HIC_ASM_0.217	coiled-coil domain-con	Coiled-coil domain-co	--	
evm.model.HIC_ASM_17.809.2	platelet endothelial agg	Platelet endothelial agg	K02258	cytochrome c
evm.model.HIC_ASM_10.1805	circadian-associated tra	Circadian-associated tr	K21599	circadian-asso
evm.model.HIC_ASM_4.1812	PREDICTED: kelch-lil	Kelch-like protein 25 C	K10462	kelch-like prot
evm.model.HIC_ASM_14.938	DUSP13 [Cervus elap	Dual specificity protein	K14165	atypical dual s
evm.model.HIC_ASM_2.1519	meiosis regulator and r	Meiosis regulator and r	K17573	meiosis arrest
evm.model.HIC_ASM_10.1217	VWFA and cache dom	VWFA and cache dom	K10798	poly [ADP-rib
evm.model.HIC_ASM_13.20	thyroid peroxidase [Fe	Thyroid peroxidase OS	K00431	thyroid peroxi
evm.model.HIC_ASM_15.561	potassium voltage-gate	Potassium voltage-gate	K04928	potassium volt
evm.model.HIC_ASM_3.1425.1	vascular endothelial gr	Vascular endothelial gr	K05097	FMS-like tyro
evm.model.HIC_ASM_2.892.1	kynureninase isoform X	Kynureninase OS=Hor	K01556	kynureninase |
evm.model.HIC_ASM_13.435.2	THUMP domain-conta	THUMP domain-conta	K11251	histone H2A |
evm.model.HIC_ASM_14.587	PDZ domain-containin	PDZ domain-containin	K21882	PDZ domain-c
evm.model.HIC_ASM_13.285	otof protein [Lynx par	Otoferlin OS=Rattus n	K19949	otoferlin | (Re
evm.model.HIC_ASM_7.895.1	kita-kyushu lung cance	Kita-kyushu lung canc	--	
evm.model.HIC_ASM_19.808	PREDICTED: ALS2 C	ALS2 C-terminal-like	K04575	amyotrophic la
evm.model.HIC_ASM_12.418.2	transmembrane protein	Transmembrane protein	K15281	solute carrier f
evm.model.HIC_ASM_0.225	PREDICTED: hairy an	Hairy and enhancer of	K09091	hairy and enha
evm.model.HIC_ASM_20.168	protein phosphatase Sl	Protein phosphatase Sl	K05766	protein phosph
evm.model.HIC_ASM_2.869	PREDICTED: methyl-(	Methyl-CpG-binding d	K23219	methyl-CpG-b
evm.model.HIC_ASM_19.204	ankyrin repeat domain-	Ankyrin repeat domain	--	
evm.model.HIC_ASM_11.274	PREDICTED: peroxiso	Peroxisomal carnitine (	K05940	carnitine O-oc
evm.model.HIC_ASM_5.1188	plakophilin-2 [Canis lu	Plakophilin-2 OS=Hon	K12642	plakophilin 2 |
evm.model.HIC_ASM_10.216	carbonic anhydrase 6 [	Carbonic anhydrase 6 (	K01672	carbonic anhy
evm.model.HIC_ASM_9.469	PREDICTED: FK506-l	FK506-binding protein	K17478	FK506-bindin
evm.model.HIC_ASM_12.782	oxysterol-binding prote	Oxysterol-binding prot	K20462	oxysterol-bind
evm.model.HIC_ASM_17.700	PREDICTED: CD48 a	CD48 antigen OS=Hon	K06479	CD48 antigen
evm.model.HIC_ASM_11.811.1	intraflagellar transport	Intraflagellar transport	K19685	intraflagellar t
evm.model.HIC_ASM_6.832	serine/threonine-protei	Serine/threonine-protei	K08828	intestinal cell
evm.model.HIC_ASM_14.787.5	WASH complex subun	WASH complex subun	K18462	WASH compl
evm.model.HIC_ASM_4.897.3	PREDICTED: AP-4 co	AP-4 complex subunit	K12403	AP-4 complex
evm.model.HIC_ASM_8.1287	PREDICTED: E3 ubiq	E3 ubiquitin-protein lig	K09228	KRAB domain
evm.model.HIC_ASM_14.161	PREDICTED: disinteg	Disintegrin and metallo	K06540	disintegrin and
evm.model.HIC_ASM_4.68	PREDICTED: beclin 1	Beclin 1-associated aut	K17889	beclin 1-assoc
evm.model.HIC_ASM_14.575	PREDICTED: F-box/V	F-box/WD repeat-conta	K10262	F-box and WD
evm.model.HIC_ASM_6.621	potassium channel sub	Potassium channel sub	K04924	potassium cha
evm.model.HIC_ASM_1.840	myosin light chain smo	Myosin light chain kina	K00907	myosin-light-c
evm.model.HIC_ASM_18.1500	DNA replication factor	DNA replication factor	K10727	chromatin lice
evm.model.HIC_ASM_18.849	coiled-coil domain-con	Centrosomal protein of	K16543	centrosomal p
evm.model.HIC_ASM_19.1033.2	PREDICTED: rho guan	Rho guanine nucleotide	K20683	Rho guanine n

evm.model.HIC_ASM_19.745.2	SURP and G-patch dor	SURP and G-patch dor	K13096	splicing factor
evm.model.HIC_ASM_4.278.2	ubiquinone biosynthesi	Ubiquinone biosynthes	K06126	ubiquinone bio
evm.model.HIC_ASM_11.176	PREDICTED: neurona	Neuronal cell adhesion	K06756	neuronal cell a
evm.model.HIC_ASM_5.1077	PREDICTED: coiled-c	Dynein regulatory com	--	
evm.model.HIC_ASM_4.1815	cytosolic carboxypepti	Cytosolic carboxypepti	K23436	cytosolic carbo
evm.model.HIC_ASM_13.24	myelin transcription fa	Myelin transcription fa	K23193	myelin transcr
evm.model.HIC_ASM_17.48.7	retinoblastoma-binding	Retinoblastoma-bindin	K14961	COMPASS co
evm.model.HIC_ASM_21.204	uncharacterized protein	Uncharacterized protei	K01855	tRNA pseudou
evm.model.HIC_ASM_0.1285	platelet-derived growth	Platelet-derived growth	K04363	platelet-derive
evm.model.HIC_ASM_0.1037	PREDICTED: dentin n	Dentin matrix acidic pl	K23328	dentin matrix a
evm.model.HIC_ASM_2.1586	PREDICTED: voltage-	Voltage-dependent calc	K04868	voltage-depen
evm.model.HIC_ASM_13.311	dnaJ homolog subfamil	DnaJ homolog subfami	K09525	DnaJ homolog
evm.model.HIC_ASM_15.618	PREDICTED: collager	Collagen alpha-1(XXII	K16630	collagen, type
evm.model.HIC_ASM_2.1269.2	THO complex subunit	THO complex subunit	K13175	THO complex
evm.model.HIC_ASM_4.351	calcium permeable stre	Calcium permeable stre	K21989	calcium perme
evm.model.HIC_ASM_1.1310	uromodulin-like 1 [Ac	Uromodulin-like 1 OS=	K18274	uromodulin | (
evm.model.HIC_ASM_5.44.1	cysteine-rich with EGF	Cysteine-rich with EGI	K17675	ATP-dependen
evm.model.HIC_ASM_10.460	basement membrane-sp	Basement membrane-sp	K06255	basement mem
evm.model.HIC_ASM_8.69	interleukin-18 isoform	Interleukin-18 OS=Feli	K05482	interleukin 18
evm.model.HIC_ASM_21.663	sodium/hydrogen excha	Sodium/hydrogen exch	K14724	solute carrier f
evm.model.HIC_ASM_18.1135.1	PREDICTED: leucine-	Leucine-rich repeat-con	K16594	leucine-rich re
evm.model.HIC_ASM_10.733	coiled-coil domain-con	Coiled-coil domain-con	K04129	muscarinic ace
evm.model.HIC_ASM_11.836	DENN domain-contain	DENN domain-contain	K20161	DENN domain
evm.model.HIC_ASM_9.490	PREDICTED: sushi do	Sushi domain-containi	K17495	CUB and sush
evm.model.HIC_ASM_6.1498	PREDICTED: fibronec	Fibronectin type III dor	K01313	coagulation fa
evm.model.HIC_ASM_17.943	PREDICTED: long-cha	Solute carrier family 2	K08772	solute carrier f
evm.model.HIC_ASM_7.134.3	PREDICTED: ankyrin	Ankyrin repeat and SO	K10333	ankyrin repeat
evm.model.HIC_ASM_17.341	PREDICTED: procolla	Procollagen galactosyl	K11703	collagen beta-
evm.model.HIC_ASM_6.27.3	valacyclovir hydrolase	Valacyclovir hydrolase	K18399	valacyclovir h
evm.model.HIC_ASM_16.402	PREDICTED: WD rep	WD repeat and FYVE	K23299	WD repeat and
evm.model.HIC_ASM_17.4	eukaryotic translation i	Eukaryotic translation	K15027	translation init
evm.model.HIC_ASM_14.570	Kv channel-interacting	Kv channel-interacting	K16575	centractin | (R
evm.model.HIC_ASM_5.515.3	WASH complex subun	WASH complex subun	K18463	WASH comple
evm.model.HIC_ASM_3.204	zinc finger protein 622	Zinc finger protein 622	K14816	pre-60S factor
evm.model.HIC_ASM_9.831.2	PREDICTED: receptor	Receptor-type tyrosine	K06777	receptor-type t
evm.model.HIC_ASM_14.287.1	Hypothetical predicted	BRCA2 and CDKN1A	K15262	protein BCP1
evm.model.HIC_ASM_8.314	cell adhesion [Lynx pa	Cell adhesion molecule	K20033	cell adhesion r
evm.model.HIC_ASM_21.386	matrix metalloproteina	Matrix metalloproteina	K08002	matrix metallo
evm.model.HIC_ASM_12.590.2	protein HIRA isoform	Protein HIRA OS=Hon	K11293	protein HIRA/
evm.model.HIC_ASM_8.1190_ev	diacylglycerol kinase z	Diacylglycerol kinase z	K00901	diacylglycerol
evm.model.HIC_ASM_21.514.2	PREDICTED: intraflag	Intraflagellar transport	K19681	intraflagellar t
evm.model.HIC_ASM_2.1675	PREDICTED: insulin-l	Insulin-like growth fac	--	
evm.model.HIC_ASM_7.86	LOW QUALITY PRO	Rho GTPase-activating	K20631	Rho GTPase-a
evm.model.HIC_ASM_17.1078	centrosomal protein of	Centrosomal protein of	K16463	centrosomal pr

evm.model.HIC_ASM_8.1789.1	N-acetyl-beta-glucosan	N-acetyl-beta-glucosan	K09657	beta-1,4-N-ace
evm.model.HIC_ASM_21.341	BPI fold-containing far	BPI fold-containing far	K08761	phospholipid t
evm.model.HIC_ASM_14.678	opalin-like [Lynx pard	Opalin OS=Homo sapi	--	
evm.model.HIC_ASM_14.432	pancreatic lipase-relate	Pancreatic lipase-relate	K14076	pancreatic lipa
evm.model.HIC_ASM_18.1543	Fanconi anemia group	Fanconi anemia group	K10888	fanconi anemi
evm.model.HIC_ASM_2.1715	proline-rich protein 14	Proline-rich protein 14	--	
evm.model.HIC_ASM_3.1344	PREDICTED: proline-	Protein Largen OS=Ho	--	
evm.model.HIC_ASM_19.782.1	c-c chemokine receptoı	C-C chemokine recepto	K04184	C-C chemokin
evm.model.HIC_ASM_1.32.1	PREDICTED: upstrear	Upstream-binding prot	K09275	transcription f
evm.model.HIC_ASM_9.368	prostaglandin G/H synt	Prostaglandin G/H syn	K00509	prostaglandin-
evm.model.HIC_ASM_19.1296	Hypothetical predicted	Coiled-coil domain-coı	K05724	FYVE, RhoGI
evm.model.HIC_ASM_2.546	dynein heavy chain 7, a	Dynein heavy chain 7,	K10408	dynein heavy
evm.model.HIC_ASM_10.600.1	GPN-loop GTPase 2 is	GPN-loop GTPase 2 O	K06883	uncharacterize
evm.model.HIC_ASM_4.282	protein lin-52 homolog	Protein lin-52 homolog	K21775	protein lin-52
evm.model.HIC_ASM_9.286.1	PREDICTED: cyclin-d	Cyclin-dependent kina	K02211	cyclin-depend
evm.model.HIC_ASM_19.851	Collagen alpha-1(VII)	Collagen alpha-1(VII)	K16628	collagen, type
evm.model.HIC_ASM_21.415	kinetochore-associated	Kinetochore-associatec	K11544	kinetochore-as
evm.model.HIC_ASM_19.1229	PREDICTED: FANCD	FANCD2 opposite stra	--	
evm.model.HIC_ASM_10.363	PREDICTED: SUZ do	SUZ domain-containin	K20069	adaptin ear-bir
evm.model.HIC_ASM_14.948.4	calcium/calmodulin-de	Calcium/calmodulin-de	K04515	calcium/calmo
evm.model.HIC_ASM_5.1115.4	sodium-coupled neutra	Sodium-coupled neutra	K14207	solute carrier f
evm.model.HIC_ASM_17.131	low quality protein: ne	Neuron navigator 1 OS	K16776	neuron naviga
evm.model.HIC_ASM_20.1566	PREDICTED: A-kinas	A-kinase anchor protei	K16518	A-kinase anch
evm.model.HIC_ASM_20.110.2	PREDICTED: nitric ox	Nitric oxide synthase, i	K13241	nitric-oxide sy
evm.model.HIC_ASM_9.140	PREDICTED: general	General transcription f	K11310	general transc
evm.model.HIC_ASM_18.1068.1	putative sodium-couple	Putative sodium-couple	K14994	solute carrier f
evm.model.HIC_ASM_4.259	ppn_human ame: full=	Papilin OS=Homo sapi	K08621	a disintegrin a
evm.model.HIC_ASM_6.129_evı	dysbindin [Puma conc	Dysbindin OS=Homo s	K20189	dysbindin | (R
evm.model.HIC_ASM_12.450	pituitary adenylate cyc	Pituitary adenylate cyc	K05262	pituitary aden
evm.model.HIC_ASM_12.698	beta-crystallin B1 [Vu	Beta-crystallin B1 OS=	K00910	beta-adrenergi
evm.model.HIC_ASM_0.1129	lysosome membrane pı	Lysosome membrane p	K12384	lysosome mem
evm.model.HIC_ASM_5.1204	PREDICTED: caprin-2	Caprin-2 OS=Homo sa	K18744	caprin-2 | (Ref
evm.model.HIC_ASM_20.1391.1	protein AF-17 isoform	Protein AF-17 OS=Hoı	K05175	gamma-amino
evm.model.HIC_ASM_2.1080	axin-1 isoform 2 [Lyn	Axin-1 OS=Mus muscı	K02157	axin 1 | (RefSe
evm.model.HIC_ASM_2.1452	PREDICTED: zinc fin	Zinc finger CCCH dom	K21991	protein unc-45
evm.model.HIC_ASM_7.446.2	grip1-associated protei	GRIP1-associated prot	--	
evm.model.HIC_ASM_20.1647.1	PREDICTED: breast c	Breast carcinoma-ampl	K22991	WD repeat-coı
evm.model.HIC_ASM_1.884	probable g-protein cou	Probable G-protein cou	K04617	gamma-amino
evm.model.HIC_ASM_10.6.2	PREDICTED: PGC-1 a	PGC-1 and ERR-induc	--	
evm.model.HIC_ASM_5.888	nck-associated protein	Nck-associated protein	K05750	NCK-associat
evm.model.HIC_ASM_2.1392	glutamate receptor ion	Glutamate receptor ion	K05209	glutamate rece
evm.model.HIC_ASM_10.1140	PREDICTED: acyl-coe	Acyl-coenzyme A thio	K12417	acyl-CoA thio
evm.model.HIC_ASM_15.799	PREDICTED: forkhea	Forkhead box protein F	K09400	forkhead box p
evm.model.HIC_ASM_5.794.6	rho guanine nucleotide	Rho guanine nucleotid	K20690	Rho guanine n

evm.model.HIC_ASM_6.1143	adenylate kinase 9 [Ac	Adenylate kinase 9 OS	K18533	adenylate/nucl
evm.model.HIC_ASM_1.379	PREDICTED: G protei	Rhodopsin kinase OS=	K00909	rhodopsin kina
evm.model.HIC_ASM_20.145	serine/threonine-protei	Serine/threonine-protei	K20877	NIMA (never
evm.model.HIC_ASM_1.900	T-lymphocyte activatic	T-lymphocyte activatic	K05412	CD80 antigen
evm.model.HIC_ASM_13.647	electrogenic sodium bi	Electrogenic sodium bi	K13857	solute carrier f
evm.model.HIC_ASM_5.473	transcription factor RF	Transcription factor RI	K09174	regulatory fact
evm.model.HIC_ASM_9.875	PREDICTED: fructose	Fructose-1,6-bisphospł	K03841	fructose-1,6-bi
evm.model.HIC_ASM_10.1637	AMP deaminase 1 [Fu	AMP deaminase 1 OS=	K01490	AMP deamina
evm.model.HIC_ASM_2.850.1	telomere-associated pro	Telomere-associated pı	K11138	telomere-assoc
evm.model.HIC_ASM_18.338	putative adrenomedulli	ADM5 OS=Sus scrofa	--	
evm.model.HIC_ASM_7.560	PREDICTED: hephaes	Hephaestin OS=Homo	K14735	hephaestin [EC
evm.model.HIC_ASM_19.821.6	kinesin-like protein KI	Kinesin-like protein KI	K10397	kinesin family
evm.model.HIC_ASM_5.868	SAP domain-containin	SAP domain-containin	K18732	SAP domain-c
evm.model.HIC_ASM_4.554	PREDICTED: B2 brad	B2 bradykinin receptor	K03916	bradykinin rec
evm.model.HIC_ASM_18.1032.2	PREDICTED: ubiquin	Ubiquinone biosynthes	K18587	ubiquinone bio
evm.model.HIC_ASM_5.1392	alpha-2-macroglobulin	Alpha-2-macroglobulir	K03910	alpha-2-macro
evm.model.HIC_ASM_6.572	PREDICTED: transcrip	Transcription factor ET	K03211	ETS transloca
evm.model.HIC_ASM_11.451.1	PREDICTED: insulin-l	Insulin-like growth fac	K13197	insulin-like gro
evm.model.HIC_ASM_10.640	eyes absent homolog 3	Eyes absent homolog 3	K17621	eyes absent ho
evm.model.HIC_ASM_10.1494	netrin-G1 isoform X1	Netrin-G1 OS=Homo s	K07522	netrin-G1 | (Re
evm.model.HIC_ASM_18.55.12	ccr4-not transcription c	CCR4-NOT transcripti	K12580	CCR4-NOT tr
evm.model.HIC_ASM_0.1300	PREDICTED: mitocho	Mitochondria-eating pı	K22257	mitochondria-
evm.model.HIC_ASM_15.395	regulating synaptic me	Regulating synaptic me	K15297	regulating syn
evm.model.HIC_ASM_11.656	hyaluronidase ph-20-lil	Hyaluronidase PH-20 (	K01197	hyaluronogluc
evm.model.HIC_ASM_0.903	Visual pigment-like rec	Leucine-rich repeat, in	K04253	visual pigment
evm.model.HIC_ASM_0.1474	dihydropteridine reduc	Dihydropteridine reduc	K00357	dihydropteridi
evm.model.HIC_ASM_6.619.1	potassium channel sub	Potassium channel sub	K04916	potassium cha
evm.model.HIC_ASM_18.420	zinc finger protein 114	Zinc finger protein 114	K09228	KRAB domain
evm.model.HIC_ASM_21.383	PREDICTED: myosin-	Myosin-7B OS=Homo	K10352	myosin heavy
evm.model.HIC_ASM_1.788	PREDICTED: serine/tl	Serine/threonine-protei	K04410	p21-activated
evm.model.HIC_ASM_20.1238	vacuolar protein-sortin	Vacuolar protein-sortir	K12189	ESCRT-II con
evm.model.HIC_ASM_6.156	RecName: Full=Thiopı	Thiopurine S-methyltra	K00569	thiopurine S-m
evm.model.HIC_ASM_13.842_ev	follicle-stimulating hor	Follicle-stimulating ho	K04247	follicle stimula
evm.model.HIC_ASM_7.599	sorting nexin-12 [Neo	Sorting nexin-12 OS=H	K17918	sorting nexin-3
evm.model.HIC_ASM_20.898	PREDICTED: signal re	Signal recognition part	K03107	signal recogni
evm.model.HIC_ASM_2.2031	hypothetical protein DI	Aminoacyl tRNA synth	K15438	aminoacyl tRN
evm.model.HIC_ASM_20.1045	low quality protein: ax	Axin-2 OS=Homo sapi	K04385	axin 2 | (RefSe
evm.model.HIC_ASM_6.358.9	unnamed protein produ	Flotillin-1 OS=Bos tau	K07192	flotillin | (RefS
evm.model.HIC_ASM_4.1462	PREDICTED: von Wil	Integrator complex sub	K23424	protein O-man
evm.model.HIC_ASM_1.1414_ev	PREDICTED: pericent	Pericentrin OS=Homo	K16481	pericentrin | (F
evm.model.HIC_ASM_14.681.6	PREDICTED: B-cell li	B-cell linker protein O	K07371	B-cell linker p
evm.model.HIC_ASM_5.1721	tRNA (cytosine(38)-C(	tRNA (cytosine(38)-C(	K15336	tRNA (cytosin
evm.model.HIC_ASM_21.127	BTB/POZ domain-con	BTB/POZ domain-con	K10478	BTB/POZ don
evm.model.HIC_ASM_0.467	PREDICTED: bifuncti	Bifunctional epoxide h	K08726	soluble epoxid

evm.model.HIC_ASM_13.335.2	ribokinase isoform X2	Ribokinase OS=Homo	K00852	ribokinase [EC
evm.model.HIC_ASM_17.129	importin 9, isoform CR	Importin-9 OS=Mus m	K20224	importin-9 | (R
evm.model.HIC_ASM_15.187.1	lymphocyte antigen 96	Lymphocyte antigen 96	K05400	lymphocyte an
evm.model.HIC_ASM_19.96.3	PREDICTED: MAP ki	MAP kinase-interacting	K04372	MAP kinase in
evm.model.HIC_ASM_19.1339	PREDICTED: EF-hand	EF-hand and coiled-coi	--	
evm.model.HIC_ASM_7.299	cytochrome b-245 heav	Cytochrome b-245 hea	K21421	NADPH oxida
evm.model.HIC_ASM_18.948	PREDICTED: sorting n	Sorting nexin-20 OS=H	K17931	sorting nexin-2
evm.model.HIC_ASM_17.199.13	complement decay-acc	Complement decay-acc	K04006	decay accelera
evm.model.HIC_ASM_0.619	folliculin-interacting pr	Folliculin-interacting p	K20401	folliculin-inter
evm.model.HIC_ASM_19.521.1	voltage-dependent P/Q	Voltage-dependent P/Q	K04344	voltage-depend
evm.model.HIC_ASM_13.498	tyrosine-protein kinase	Tyrosine-protein kinase	K05117	c-mer proto-on
evm.model.HIC_ASM_21.77	PREDICTED: crooked	Crooked neck-like prot	K12869	crooked neck |
evm.model.HIC_ASM_10.349	msx2-interacting protei	Msx2-interacting prote	K13190	RNA-binding
evm.model.HIC_ASM_6.409	von Willebrand factor	von Willebrand factor	K17341	hemicentin | (R
evm.model.HIC_ASM_21.380	acetyl-coenzyme a [Ly	Acetyl-coenzyme A syn	K01895	acetyl-CoA sy
evm.model.HIC_ASM_1.188	PREDICTED: 5-azacyt	5-azacytidine-induced p	K12651	5-azacytidine-
evm.model.HIC_ASM_2.1685	myc-associated zinc fin	Myc-associated zinc fin	K03252	translation init
evm.model.HIC_ASM_17.992	protein cornichon hom	Protein cornichon hom	K20368	protein cornich
evm.model.HIC_ASM_11.227	PREDICTED: [Lynx p	N-acyl-phosphatidyleth	K13985	N-acyl-phosph
evm.model.HIC_ASM_4.1391.6	BCL2/adenovirus E1B	BCL2/adenovirus E1B	K18448	BCL2/adenovi
evm.model.HIC_ASM_0.421	rho-related BTB domai	Rho-related BTB doma	K07868	Rho-related BT
evm.model.HIC_ASM_18.1097	Coiled-coil domain-con	Telomere repeats-bindi	K10416	dynein light in
evm.model.HIC_ASM_6.728	exportin-5 isoform 3 [	Exportin-5 OS=Homo s	K14289	exportin-5 | (R
evm.model.HIC_ASM_0.1117	CCR4-NOT transcripti	CCR4-NOT transcripti	K12603	CCR4-NOT tr
evm.model.HIC_ASM_16.128.1	PREDICTED: testis-ex	Testis-expressed protei	--	
evm.model.HIC_ASM_12.787	selenoprotein M isofor	Selenoprotein M OS=H	--	
evm.model.HIC_ASM_8.1108	PREDICTED: COMM	COMM domain-contai	K22565	COMM domai
evm.model.HIC_ASM_2.1950	PREDICTED: actin-lik	Actin-like protein 6B C	K11652	actin-like prot
evm.model.HIC_ASM_19.1045	PREDICTED: protein	Protein DENND6A OS	K07939	ADP-ribosylat
evm.model.HIC_ASM_19.1403.3	PREDICTED: Kruepp	Krueppel-like factor 15	K09210	krueppel-like f
evm.model.HIC_ASM_15.138	PREDICTED: MCM d	Minichromosome main	--	
evm.model.HIC_ASM_4.966	capping protein, Arp2/3	Capping protein, Arp2/	K20493	leucine-rich re
evm.model.HIC_ASM_1.1406.1	formimidoyltransferase	Formimidoyltransferas	K13990	glutamate form
evm.model.HIC_ASM_12.950.1	PREDICTED: ras-relat	Ras-related protein Ral	K07876	Ras-related pro
evm.model.HIC_ASM_12.1093.1	Anaphase-promoting co	GPN-loop GTPase 3 O	K06883	uncharacterize
evm.model.HIC_ASM_14.856	hypothetical protein EI	Leucine-rich repeat, im	K16354	leucine-rich re
evm.model.HIC_ASM_12.1149.3	PREDICTED: RILP-lik	RILP-like protein 2 OS	K20173	RILP-like prot
evm.model.HIC_ASM_12.875	transient receptor poten	Transient receptor pote	K04973	transient recep
evm.model.HIC_ASM_17.15	ras-related protein Rab	Ras-related protein Ral	K07898	Ras-related pro
evm.model.HIC_ASM_7.820_evm	RPA-related protein RA	RPA-related protein RA	K10629	E3 ubiquitin-p
evm.model.HIC_ASM_4.811	PREDICTED: Fanconi	Fanconi anemia group	K10896	fanconi anemi
evm.model.HIC_ASM_16.415	phosphatidylinositol 3,	Phosphatidylinositol 3,	K18079	PTEN homolo
evm.model.HIC_ASM_0.1673	E3 ubiquitin-protein tra	E3 ubiquitin-protein tra	K18624	macrophage er
evm.model.HIC_ASM_14.719.1	PREDICTED: exocyst	Exocyst complex comp	K19985	exocyst compl

evm.model.HIC_ASM_15.195	PREDICTED: ganglios	Ganglioside-induced di	K22077	ganglioside-in
evm.model.HIC_ASM_4.97	PREDICTED: trans-3-l	Trans-3-hydroxy-L-pro	K18384	trans-L-3-hydr
evm.model.HIC_ASM_21.346	BPI fold-containing far	BPI fold-containing far	--	
evm.model.HIC_ASM_0.254	PREDICTED: deoxycy	Deoxycytidylate deami	K01493	dCMP deamin
evm.model.HIC_ASM_13.231	apolipoprotein B-100 |	Apolipoprotein B-100 (	K14462	apolipoprotein
evm.model.HIC_ASM_9.600	Tropomodulin-1 [Tupa	Tropomodulin-1 OS=B	K10370	tropomodulin
evm.model.HIC_ASM_1.439	eukaryotic translation i	Eukaryotic translation	K15026	translation init
evm.model.HIC_ASM_2.918	mitogen-activated prot	Mitogen-activated prot	K17533	mitogen-activa
evm.model.HIC_ASM_17.520	PREDICTED: potassiu	Potassium channel sub	K04947	potassium cha
evm.model.HIC_ASM_20.603.1	PREDICTED: C-X-C r	C-X-C motif chemokin	K10035	C-X-C motif c
evm.model.HIC_ASM_0.140	PREDICTED: tankyras	Poly [ADP-ribose] poly	K10799	tankyrase [EC
evm.model.HIC_ASM_5.315.2	chromobox protein hor	Chromobox protein hor	K11454	chromobox pro
evm.model.HIC_ASM_13.827	PREDICTED: unchara	Uncharacterized protei	--	
evm.model.HIC_ASM_2.1637	linker for activation of	Linker for activation of	K07362	linker for activ
evm.model.HIC_ASM_8.220.3	PREDICTED: LOW Q	Cell surface glycoprote	K06534	melanoma cell
evm.model.HIC_ASM_10.993	PREDICTED: dual spe	Dual specificity testis-s	K08842	testis-specific
evm.model.HIC_ASM_4.1200	MAX gene-associated	MAX gene-associated	K23399	MAX gene-ass
evm.model.HIC_ASM_0.1569	transcriptional adapter	Transcriptional adapter	K15127	transcriptional
evm.model.HIC_ASM_4.1261	spatacsin isoform X2 [	Spatacsin OS=Homo sa	K19026	spatacsin | (Re
evm.model.HIC_ASM_9.185.1	tyrosine-protein kinase	Tyrosine-protein kinase	K06619	abelson tyrosin
evm.model.HIC_ASM_8.1702.2	PREDICTED: liprin-al	Liprin-alpha-1 OS=Hor	K07762	pappalysin-1 [
evm.model.HIC_ASM_18.789.3	PREDICTED: supraba	Suprabasin OS=Bos tau	K18626	trichohyalin | (
evm.model.HIC_ASM_2.228	unnamed protein produ	Calcium-binding protei	K08272	calcium bindin
evm.model.HIC_ASM_3.352	PREDICTED: NAD(P)	NAD(P) transhydrogen	K00323	H+-translocati
evm.model.HIC_ASM_21.992	PREDICTED: myelin t	Myelin transcription fa	K23192	myelin transcr
evm.model.HIC_ASM_0.460	stathmin-4 isoform X1	Stathmin-4 OS=Pongo	K12012	tripartite motif
evm.model.HIC_ASM_19.1076	PREDICTED: receptor	Receptor-type tyrosine-	K16667	receptor-type t
evm.model.HIC_ASM_5.146	ataxin-10 isoform X2 |	Ataxin-10 OS=Bos tau	K19323	ataxin-10 | (Re
evm.model.HIC_ASM_17.868.4	protein misato homolog	Protein misato homolog	K17408	small subunit r
evm.model.HIC_ASM_6.661	LOW QUALITY PRO	Transcription factor EI	K15590	transcription f
evm.model.HIC_ASM_17.292	sulfhydryl oxidase 1 [I	Sulfhydryl oxidase 1 O	K10758	thiol oxidase [
evm.model.HIC_ASM_5.240.1	PREDICTED: phospho	Phosphomannomutase	K17497	phosphomanno
evm.model.HIC_ASM_19.412	gata-type zinc finger pr	GATA-type zinc finger	K21630	GATA-type zi
evm.model.HIC_ASM_15.200	hepatocyte nuclear fact	Hepatocyte nuclear fac	K08037	hepatocyte nuc
evm.model.HIC_ASM_10.913	polycomb protein SCM	Polycomb protein SCM	K11461	polycomb prot
evm.model.HIC_ASM_6.146	PREDICTED: adenyly	Adenylyl cyclase-assoc	K17261	adenylyl cycla
evm.model.HIC_ASM_17.731	SLAM family member	SLAM family member	K16853	SLAM family
evm.model.HIC_ASM_3.1217	solute carrier family 25	Solute carrier family 25	K15124	solute carrier f
evm.model.HIC_ASM_7.643.1	cysteine-rich hydropho	Cysteine-rich hydropho	--	
evm.model.HIC_ASM_12.742.3	PREDICTED: AP-1 co	AP-1 complex subunit	K12392	AP-1 complex
evm.model.HIC_ASM_4.204.1	alpha-actinin-1 isoform	Alpha-actinin-1 OS=Bo	K05699	actinin alpha 1
evm.model.HIC_ASM_5.693	leucine-rich repeat-con	Leucine-rich repeat-cor	K04308	leucine-rich re
evm.model.HIC_ASM_17.72	opticin [Puma concolo	Opticin OS=Canis lupu	K08128	opticin | (RefS
evm.model.HIC_ASM_2.599	PREDICTED: E3 ubiq	E3 ubiquitin-protein lig	K15716	E3 ubiquitin-p

evm.model.HIC_ASM_2.1353	PREDICTED: UPF076	UPF0764 protein C16c	--	
evm.model.HIC_ASM_20.863.2	sec14-like protein 1 isc	SEC14-like protein 1 C	K17981	mitochondrial
evm.model.HIC_ASM_17.1088	WD repeat-containing	WD repeat-containing	K02988	small subunit
evm.model.HIC_ASM_18.951	ubiquitin carboxyl-tern	Ubiquitin carboxyl-terr	K08601	ubiquitin carb
evm.model.HIC_ASM_4.154	spectrin beta chain, ery	Spectrin beta chain, ery	K06115	spectrin beta |
evm.model.HIC_ASM_2.1541.1	PREDICTED: ketimine	Ketimine reductase mu	K18258	thiomorpholin
evm.model.HIC_ASM_7.33	neuroligin-4, X-linked	Neuroligin-4, X-linked	K07378	neuroligin | (R
evm.model.HIC_ASM_3.149	methionine synthase re	Methionine synthase re	K00597	methionine sy
evm.model.HIC_ASM_5.1202	DENN domain-contain	DENN domain-contain	K20164	DENN domain
evm.model.HIC_ASM_21.753	PREDICTED: transcri	Transcription factor AI	K09177	transcription f
evm.model.HIC_ASM_13.937	cytochrome c oxidase a	Cytochrome c oxidase	K18178	cytochrome c
evm.model.HIC_ASM_11.211	histone-lysine N-methy	Inactive histone-lysine	K09189	histone-lysine
evm.model.HIC_ASM_2.1963	TSC22 domain family	TSC22 domain family	--	
evm.model.HIC_ASM_16.63.2	PREDICTED: rho guar	Rho guanine nucleotide	K13710	Rho guanine n
evm.model.HIC_ASM_17.784	CD5 antigen-like [Pun	CD5 antigen-like OS=I	K13912	deleted in mal
evm.model.HIC_ASM_2.1883	serine/threonine/tyrosi	Serine/threonine/tyrosi	K18047	serine/threonin
evm.model.HIC_ASM_6.1466	PREDICTED: NADPH	NADPH oxidase 3 OS=	K21422	NADPH oxida
evm.model.HIC_ASM_2.330	PREDICTED: autopha	Autophagy-related prot	K17907	autophagy-rela
evm.model.HIC_ASM_18.1485	zinc finger protein 469	Zinc finger protein 469	--	
evm.model.HIC_ASM_10.893	PREDICTED: transme	Transmembrane and cc	--	
evm.model.CTG_466.12	PREDICTED: ADNP h	Activity-dependent neu	K22591	activity-depen
evm.model.HIC_ASM_18.60	tRNA-splicing endonu	tRNA-splicing endonu	K15323	tRNA-splicing
evm.model.HIC_ASM_6.1125	PREDICTED: lactation	AFG1-like ATPase OS	K18798	peroxisome-as
evm.model.HIC_ASM_4.257	presenilin-1 isoform X	Presenilin-1 OS=Canis	K04505	presenilin 1 [E
evm.model.HIC_ASM_1.966	PREDICTED: PI-PLC	PI-PLC X domain-cont	K01027	3-oxoacid CoA
evm.model.HIC_ASM_14.669	PREDICTED: slit hom	Slit homolog 1 protein	K06838	slit 1 | (RefSec
evm.model.HIC_ASM_5.409	thioredoxin, mitochond	Thioredoxin, mitochon	K03671	thioredoxin 1 |
evm.model.HIC_ASM_0.1174	PREDICTED: neurope	Neuropeptide FF recep	K08375	neuropeptide F
evm.model.HIC_ASM_10.888.5	sodium-dependent lyso	Sodium-dependent lyso	K08824	cyclin-depend
evm.model.HIC_ASM_17.294	PREDICTED: LIM/hor	LIM/homeobox protein	K09374	LIM homeobo
evm.model.HIC_ASM_2.1179	PREDICTED: cytosoli	Cytosolic Fe-S cluster	K03593	ATP-binding p
evm.model.HIC_ASM_0.615	PREDICTED: rap guar	Rap guanine nucleotide	K08018	Rap guanine n
evm.model.CTG_805.3	PREDICTED: calmodu	Calmodulin-regulated s	K17493	calmodulin-re
evm.model.HIC_ASM_9.1016	KN motif and ankyrin	KN motif and ankyrin	K22808	KN motif and
evm.model.HIC_ASM_12.1156	general transcription fa	General transcription fa	K03143	transcription i
evm.model.HIC_ASM_5.544	inhibitor of nuclear fac	Inhibitor of nuclear fac	K12493	ADP-ribosylat
evm.model.HIC_ASM_12.665	small G protein signali	Small G protein signali	K21847	small G protei
evm.model.HIC_ASM_8.3	guanylate cyclase solul	Guanylate cyclase solu	K12318	guanylate cycl
evm.model.HIC_ASM_3.1131	ankyrin repeat and kh c	Ankyrin repeat and KH	K16726	ankyrin repeat
evm.model.HIC_ASM_12.525	metallophosphoesteras	Metallophosphoesteras	K23362	ethanolamine
evm.model.HIC_ASM_3.1361	aminopeptidase Q [Ne	Aminopeptidase Q OS=	K13724	aminopeptidas
evm.model.HIC_ASM_8.801	arfaptin-2 isoform X3	Arfaptin-2 OS=Bos tau	K20314	arfaptin | (Ref
evm.model.HIC_ASM_1.894	PREDICTED: protein	Cilia- and flagella-asso	K08745	solute carrier f
evm.model.HIC_ASM_6.674	PREDICTED: ubiquiti	Ubiquitin carboxyl-terr	K11834	ubiquitin carb

evm.model.HIC_ASM_5.127	polycystic kidney disea	Polycystic kidney disea	K19921	polycystic kid
evm.model.HIC_ASM_19.961.1	PREDICTED: alpha/be	Protein ABHD14B OS	K13706	abhydrolase do
evm.model.CTG_1244.6	hydroxylysine kinase [	Hydroxylysine kinase (	K18201	hydroxylysine
evm.model.HIC_ASM_9.524.4	PREDICTED: Krueppe	Krueppel-like factor 4	K17846	krueppel-like f
evm.model.HIC_ASM_21.392	PREDICTED: unchara	Uncharacterized protei	K03368	beta-galactosic
evm.model.HIC_ASM_3.1034	cell division cycle prot	Cell division cycle prot	K03363	cell division cy
evm.model.HIC_ASM_14.154.1	proline-rich acidic prot	Proline-rich acidic prot	K21250	proline-rich ac
evm.model.HIC_ASM_6.672	mitochondrial import r	Mitochondrial import r	K17772	mitochondrial
evm.model.HIC_ASM_4.759	PREDICTED: unchara	Uncharacterized protei	--	
evm.model.HIC_ASM_2.1843.1	PREDICTED: replicati	Replication factor C su	K10755	replication fac
evm.model.HIC_ASM_17.330	PREDICTED: nicotina	Nicotinamide/nicotinic	K06210	nicotinamide r
evm.model.HIC_ASM_4.559.2	PREDICTED: adenylat	Adenylate kinase 7 OS	K00939	adenylate kina
evm.model.HIC_ASM_0.1437	PREDICTED: phospha	Phosphatidylinositol 4-	K13711	phosphatidylin
evm.model.HIC_ASM_19.470	PREDICTED: calponin	Calponin-1 OS=Bos tau	K20526	transgelin | (R
evm.model.HIC_ASM_13.534	Hypothetical predicted	Testis-expressed seque	--	
evm.model.HIC_ASM_5.17	inositol oxygenase [Fe	Inositol oxygenase OS=	K00469	inositol oxyge
evm.model.HIC_ASM_10.1543	macrophage colony-sti	Macrophage colony-sti	K05453	macrophage co
evm.model.HIC_ASM_5.522	PREDICTED: myosin-	Myosin-binding protein	K12557	myosin-bindin
evm.model.HIC_ASM_7.498	PREDICTED: 3-hydro	3-hydroxyacyl-CoA de	K08683	3-hydroxyacyl
evm.model.HIC_ASM_6.552	transcriptional enhance	Transcriptional enhanc	K09448	transcriptional
evm.model.HIC_ASM_8.301	Dolichyl-diphosphoolig	Dolichyl-diphosphoolig	K07151	dolichyl-dipho
evm.model.HIC_ASM_9.714_evm	PREDICTED: charged	Charged multivesicular	K12198	charged multiv
evm.model.HIC_ASM_5.117	tbc1 domain family me	TBC1 domain family m	K20360	TBC1 domain
evm.model.HIC_ASM_5.1138	a disintegrin and metal	A disintegrin and meta	K09609	a disintegrin a
evm.model.HIC_ASM_10.1677	V-set domain-containin	V-set domain-containin	K06747	immune costim
evm.model.HIC_ASM_6.352	PREDICTED: surfacta	Surfactant-associated p	--	
evm.model.HIC_ASM_1.906	rho GTPase-activating	Rho GTPase-activating	K20646	Rho GTPase-a
evm.model.HIC_ASM_3.1314	PREDICTED: alpha-am	Alpha-aminoadipic sem	K14085	aldehyde dehy
evm.model.HIC_ASM_10.1430	PREDICTED: UDP-N-	UDP-N-acetylglucosam	K07441	beta-1,4-N-ace
evm.model.HIC_ASM_10.1149	lymphocyte expansion	Lymphocyte expansion	--	
evm.model.HIC_ASM_18.1045.4	PREDICTED: katanin	Katanin p80 WD40 rep	K18643	katanin p80 W
evm.model.HIC_ASM_10.665	PREDICTED: transme	Transmembrane protein	K18027	tyrosine-protei
evm.model.HIC_ASM_15.572	thyroglobulin [Felis ca	Thyroglobulin OS=Bos	K10809	thyroglobulin
evm.model.HIC_ASM_9.409	CDK5 regulatory subun	CDK5 regulatory subun	K16542	CDK5 regulato
evm.model.HIC_ASM_11.362	protachykinin-1 isoform	Protachykinin-1 OS=H	K05239	tachykinin 1 | (
evm.model.HIC_ASM_12.623	phosphatidylinositol 4-	Phosphatidylinositol 4-	K00888	phosphatidylin
evm.model.HIC_ASM_19.1083	PREDICTED: sentan is	Sentan OS=Bos taurus	--	
evm.model.HIC_ASM_13.275.1	kinesin family member	Kinesin-like protein KI	K20197	kinesin family
evm.model.HIC_ASM_14.734	PREDICTED: probable	Probable E3 ubiquitin-p	K12232	E3 ubiquitin-p
evm.model.HIC_ASM_20.752.1	putative sodium-couple	Putative sodium-couple	K14996	solute carrier f
evm.model.HIC_ASM_4.1875	PREDICTED: RCC1 d	RCC1 domain-containi	K21991	protein unc-45
evm.model.HIC_ASM_3.1341	PREDICTED: serum re	Serum response factor-	--	
evm.model.HIC_ASM_4.1464	LOW QUALITY PRO	Sodium/potassium/calc	K13749	solute carrier f
evm.model.HIC_ASM_6.1436	PREDICTED: LOW Q	Required for meiotic nu	K21456	glutathione sy

evm.model.HIC_ASM_6.867	lengsin [Puma concolo	Lengsin OS=Canis lup	K01915	glutamine synt
evm.model.HIC_ASM_7.151	PREDICTED: gastrin-r	Gastrin-releasing pepti	K04169	gastrin-releasi
evm.model.HIC_ASM_20.1364	PREDICTED: cyclin-d	Cyclin-dependent kinas	K08819	cyclin-depend
evm.model.HIC_ASM_4.907	forkhead box protein G	Forkhead box protein G	K09385	forkhead box p
evm.model.HIC_ASM_11.717_ev	PREDICTED: transme	Transmembrane protei	--	
evm.model.HIC_ASM_11.18.2	PREDICTED: nudC dc	NudC domain-containi	K00164	2-oxoglutarate
evm.model.HIC_ASM_8.874	PREDICTED: RNA pc	RNA polymerase-assoc	K15176	RNA polymera
evm.model.HIC_ASM_3.1121.1	LRRG00133 [Rattus n	Protein Red OS=Pongo	K13109	IK cytokine | (
evm.model.HIC_ASM_0.1473.1	Hypothetical predicted	Clarin-2 OS=Homo sap	--	
evm.model.HIC_ASM_19.726.3	PREDICTED: probable	Probable ATP-depende	K14778	ATP-depender
evm.model.HIC_ASM_7.703	PREDICTED: cylicin-1	Cylicin-1 OS=Homo sa	--	
evm.model.HIC_ASM_6.1390	metabotropic glutamate	Metabotropic glutamat	K04603	metabotropic g
evm.model.HIC_ASM_3.604.4	PREDICTED: F-box/V	F-box/WD repeat-conta	K03362	F-box and WD
evm.model.HIC_ASM_5.1022	solute carrier family 11	Natural resistance-asso	K21398	natural resista
evm.model.HIC_ASM_4.264	PREDICTED: HEAT r	HEAT repeat-containir	--	
evm.model.HIC_ASM_2.275	PREDICTED: dedicato	Dedicator of cytokines	K21853	dedicator of cy
evm.model.HIC_ASM_3.1033	PREDICTED: probable	Probable glutathione pe	K00432	glutathione pe
evm.model.HIC_ASM_2.1301	PREDICTED: clusterin	Clusterin-associated pr	K19684	clusterin-assoc
evm.model.HIC_ASM_19.974.1	hypothetical protein EI	WD repeat-containing	K14962	COMPASS co
evm.model.HIC_ASM_5.620	centrosomal protein of	Centrosomal protein of	K16533	centrosomal pr
evm.model.HIC_ASM_10.1680	PREDICTED: mannosy	Mannosyl-oligosacchar	K01230	mannosyl-olig
evm.model.HIC_ASM_18.1518	acyl-CoA synthetase fa	Acyl-CoA synthetase fa	K18660	malonyl-CoA/
evm.model.HIC_ASM_3.1219	C-X-C motif chemokin	C-X-C motif chemokin	K10033	C-X-C motif c
evm.model.HIC_ASM_19.577.2	acetolactate synthase-li	Acetolactate synthase-l	K11259	acetolactate sy
evm.model.HIC_ASM_16.449	spermatogenesis- and c	Spermatogenesis- and c	K22495	spermatogenes
evm.model.HIC_ASM_6.447	uncharacterized protein	Testis-expressed basic	--	
evm.model.HIC_ASM_5.242	DNA-directed RNA po	DNA-directed RNA po	K03022	DNA-directed
evm.model.HIC_ASM_0.457	PREDICTED: alpha-1A	Alpha-1A adrenergic re	K04135	adrenergic rec
evm.model.HIC_ASM_6.1079	PREDICTED: glutama	Glutamate receptor ion	K05202	glutamate rece
evm.model.HIC_ASM_7.767	uncharacterized protein	Armadillo repeat-conta	--	
evm.model.HIC_ASM_8.476.4	low quality protein: pro	Centrosomal protein of	K16714	anastral spindl
evm.model.HIC_ASM_0.1381	WD repeat-containing	WD repeat-containing	K19671	WD repeat-co
evm.model.HIC_ASM_19.1199	PREDICTED: protein S	Protein SSUH2 homolo	K06840	semaphorin 3
evm.model.HIC_ASM_5.1096	PREDICTED: collagen	Collagen alpha-1(II) ch	K19719	collagen, type
evm.model.HIC_ASM_17.621	PREDICTED: LIM ho	LIM homeobox transcr	K09371	LIM homeobo
evm.model.HIC_ASM_6.1592	PREDICTED: protein u	Protein unc-93 homolo	K16600	tubulin polygl
evm.model.HIC_ASM_4.1141	G-protein coupled rece	G-protein coupled rece	K08442	G protein-coup
evm.model.HIC_ASM_9.329	PREDICTED: GTPase	GTPase-activating prot	K05767	Ras GTPase-a
evm.model.HIC_ASM_19.533	PREDICTED: unchara	Uncharacterized protei	--	
evm.model.HIC_ASM_1.746	probable cation-transpo	Probable cation-transpo	K14951	cation-transpo
evm.model.HIC_ASM_7.744	protocadherin-19 isofo	Protocadherin-19 OS=I	K16499	protocadherin
evm.model.HIC_ASM_3.620.2	unnamed protein produ	Vesicle transport prote	K08497	protein transpo
evm.model.HIC_ASM_2.1299	hypothetical protein PA	N-alpha-acetyltransfera	K21121	N-alpha-acety
evm.model.HIC_ASM_19.494	microtubule-associated	Microtubule-associated	K08789	microtubule-as

evm.model.HIC_ASM_20.816	galectin-3-binding prot	Galectin-3-binding pro	K17300	galectin-3-bind
evm.model.HIC_ASM_2.189	hypothetical protein Ca	Acetylcholine receptor	K04816	nicotinic acety
evm.model.HIC_ASM_6.680	uncharacterized protein	Uncharacterized protei	--	
evm.model.HIC_ASM_5.497.2	glycosyltransferase 8 d	Glycosyltransferase 8 d	K13648	alpha-1,4-gala
evm.model.HIC_ASM_16.133	LOW QUALITY PRO	Protein-lysine methyltr	K21805	protein N-lysin
evm.model.HIC_ASM_5.1261	ATP-sensitive inward r	ATP-sensitive inward r	K05001	potassium inw
evm.model.HIC_ASM_16.264	protocadherin-9 isoforn	Protocadherin-9 OS=H	K16498	protocadherin
evm.model.HIC_ASM_1.342	PREDICTED: fas apop	Fas apoptotic inhibitor	--	
evm.model.HIC_ASM_14.967	PREDICTED: prolyl 4	Prolyl 4-hydroxylase su	K00472	prolyl 4-hydro
evm.model.HIC_ASM_20.842	synaptogyrin-2 isoform	Putative synaptogyrin-2	K21988	transmembran
evm.model.HIC_ASM_18.1265.1	PREDICTED: fatty aci	Fatty acid 2-hydroxylas	K19703	4-hydroxysphi
evm.model.HIC_ASM_12.531	AFG3-like protein 2 iso	AFG3-like protein 2 O	K08956	AFG3 family p
evm.model.HIC_ASM_2.700	LOW QUALITY PRO	Integrin alpha-6 OS=H	K06485	integrin alpha
evm.model.HIC_ASM_4.1737.1	PREDICTED: U2 smal	U2 small nuclear ribon	K11092	U2 small nucle
evm.model.HIC_ASM_20.1074.6	platelet endothelial cel	Platelet endothelial cel	K06471	platelet/endoth
evm.model.HIC_ASM_19.593	tropomyosin alpha-4 ch	Tropomyosin alpha-1 c	K10375	tropomyosin 4
evm.model.HIC_ASM_1.41	E3 ubiquitin-protein lig	E3 ubiquitin-protein lig	K12035	tripartite motif
evm.model.HIC_ASM_10.40	von Willebrand factor	von Willebrand factor	K08132	collagen, type
evm.model.HIC_ASM_4.1224	stAR-related lipid trans	StAR-related lipid tran	K16491	StAR-related l
evm.model.HIC_ASM_4.1950	transmembrane channe	Transmembrane chann	K21988	transmembran
evm.model.HIC_ASM_3.483	glutamate receptor 1 pr	Glutamate receptor 1 C	K05197	glutamate rece
evm.model.HIC_ASM_7.137	pirin isoform X1 [Feli	Pirin OS=Homo sapien	K06911	uncharacterize
evm.model.HIC_ASM_9.585	Gamma-aminobutyric a	Gamma-aminobutyric a	K04615	gamma-amino
evm.model.HIC_ASM_12.155	GPI ethanolamine phos	GPI ethanolamine phos	K05285	phosphatidylin
evm.model.HIC_ASM_1.130	PREDICTED: trafficki	Trafficking kinesin-bin	K15369	trafficking kin
evm.model.HIC_ASM_10.1571.2	choline/ethanolaminep	Choline/ethanolaminep	K13644	choline/ethano
evm.model.HIC_ASM_8.175.2	T-cell surface glycopro	T-cell surface glycopro	K06451	T-cell surface
evm.model.HIC_ASM_0.227	PREDICTED: long-cha	Long-chain-fatty-acid--	K01897	long-chain acy
evm.model.HIC_ASM_4.407.1	spermatogenesis-associ	Spermatogenesis-assoc	K19655	spermatogenes
evm.model.HIC_ASM_8.1582.2	PREDICTED: 39S ribo	39S ribosomal protein	K02867	large subunit r
evm.model.HIC_ASM_2.1792	transmembrane protein	Transmembrane protei	K20131	Rab5 GDP/GT
evm.model.HIC_ASM_10.408	E3 ubiquitin-protein lig	E3 ubiquitin-protein lig	K10691	E3 ubiquitin-p
evm.model.HIC_ASM_9.535	PREDICTED: choline	Choline transporter-lik	K06515	solute carrier f
evm.model.HIC_ASM_10.377	protein RCC2 [Zaloph	Protein RCC2 OS=Hor	K10614	E3 ubiquitin-p
evm.model.HIC_ASM_2.1048.1	PREDICTED: vitamin	Vitamin K-dependent p	K01344	protein C (acti
evm.model.HIC_ASM_17.855.1	ras-related protein Rab	Ras-related protein Ral	K07906	Ras-related pro
evm.model.HIC_ASM_10.1286	PREDICTED: alpha-N	Alpha-N-acetylgalactos	K03375	N-acetylgalact
evm.model.HIC_ASM_6.1086	E3 ubiquitin-protein lig	E3 ubiquitin-protein lig	K12166	E3 ubiquitin-p
evm.model.HIC_ASM_5.1331	probable G-protein cou	Probable G-protein cou	K04316	G protein-coup
evm.model.HIC_ASM_17.230	hypothetical protein Ca	Digestive organ expans	K14774	U3 small nucle
evm.model.HIC_ASM_19.1265	cullin-associated NEDD	Cullin-associated NED	K17263	cullin-associat
evm.model.HIC_ASM_10.392	immunoglobulin superf	Immunoglobulin super	K17341	hemicentin | (I
evm.model.HIC_ASM_3.1021	Hypothetical predicted	Ankyrin repeat domain	K19626	inversin | (Ref
evm.model.HIC_ASM_3.516	cytoplasmic FMR1-inte	Cytoplasmic FMR1-int	K05749	cytoplasmic F

evm.model.HIC_ASM_18.590	LOW QUALITY PRO'	Protein capicua homolo	K20225	capicua transc
evm.model.HIC_ASM_0.563_evi	H(+)/Cl(-) exchange tra	H(+)/Cl(-) exchange tra	K05012	chloride chann
evm.model.HIC_ASM_3.546	PREDICTED: probable	Probable phospholipid-	K01530	phospholipid-t
evm.model.HIC_ASM_4.1452	ubiquitin-associated pr	Ubiquitin-associated pr	K11292	transcription e
evm.model.HIC_ASM_17.653	PREDICTED: olfacton	Olfactomedin-like prot	--	
evm.model.HIC_ASM_2.431	Parathyroid hormone 2	Parathyroid hormone 2	K04586	parathyroid ho
evm.model.HIC_ASM_1.1394.4	GDP-fucose protein O-	GDP-fucose protein O-	K03691	peptide-O-fuco
evm.model.HIC_ASM_21.228	PREDICTED: transcrip	Transcription factor CO	K09103	early B-cell fa
evm.model.HIC_ASM_5.602	PREDICTED: coiled-c	Coiled-coil domain-con	--	
evm.model.HIC_ASM_10.28.1	integrator complex sub	Integrator complex sub	K13148	integrator com
evm.model.HIC_ASM_4.1570.11	PREDICTED: poly [A]	Protein mono-ADP-ribo	K15258	poly [ADP-rib
evm.model.HIC_ASM_10.331	forkhead-associated do	Forkhead-associated do	--	
evm.model.HIC_ASM_14.973.4	dnaJ homolog subfami	DnaJ homolog subfami	K09518	DnaJ homolog
evm.model.HIC_ASM_20.262.1	tax1-binding protein 3	Tax1-binding protein 3	K08876	SCY1-like pro
evm.model.HIC_ASM_7.1100	PREDICTED: fibrobla	Fibroblast growth facto	K22413	fibroblast grov
evm.model.HIC_ASM_0.972	PREDICTED: unchara	Uncharacterized protei	--	
evm.model.HIC_ASM_8.215.7	NLR family member X	NLR family member X	K12653	NLR family m
evm.model.HIC_ASM_8.1068	uncharacterized protein	Uncharacterized protei	--	
evm.model.HIC_ASM_12.1024	probable RNA-binding	Probable RNA-binding	K14787	multiple RNA-
evm.model.HIC_ASM_6.1492.6	ezrin isoform X1 [Feli	Ezrin OS=Homo sapien	K08007	villin 2 (ezrin)
evm.model.HIC_ASM_2.1852	transmembrane protein	Transmembrane protein	--	
evm.model.HIC_ASM_9.397	PREDICTED: TNF rec	TNF receptor-associate	K03172	TNF receptor-
evm.model.HIC_ASM_15.785	transmembrane protein	Transmembrane protein	K10272	F-box and leuc
evm.model.HIC_ASM_8.959	ubiquitin-conjugating e	Ubiquitin-conjugating	K00016	L-lactate dehy
evm.model.HIC_ASM_10.1147	PREDICTED: tetratric	Tetratricopeptide repea	K01881	prolyl-tRNA s
evm.model.HIC_ASM_3.719	thymic stromal lympho	Thymic stromal lympho	K05436	thymic stroma
evm.model.HIC_ASM_16.237	LIM domain only prote	LIM domain only prote	K06084	F-box protein
evm.model.HIC_ASM_19.327.1	PREDICTED: NADH	NADH dehydrogenase	K03951	NADH dehydr
evm.model.HIC_ASM_10.1846	E3 SUMO-protein liga	E3 SUMO-protein liga	K16064	E3 SUMO-pro
evm.model.HIC_ASM_0.480.7	gtpase slip-gc-like [Ly	Nuclear GTPase SLIP-	--	
evm.model.HIC_ASM_0.715	uncharacterized protein	Reelin domain-contain	K07756	inositol-hexak
evm.model.HIC_ASM_17.597	hhip-like 2 [Lynx pard	HHIP-like protein 2 OS	K15212	TATA box-bin
evm.model.HIC_ASM_10.450	endothelin-converting	Endothelin-converting	K01415	endothelin-con
evm.model.HIC_ASM_0.1052	mitogen-activated prot	Mitogen-activated prot	K04440	mitogen-activa
evm.model.HIC_ASM_5.376	caspase recruitment do	Caspase recruitment do	K20912	caspase recrui
evm.model.HIC_ASM_12.1056	PREDICTED: tyrosine	Tyrosine-protein phosp	K07293	tyrosine-protei
evm.model.HIC_ASM_6.902	galactosylgalactosylxyl	Galactosylgalactosylxy	K10157	galactosylgala
evm.model.HIC_ASM_18.853	putative ATP-depender	Putative ATP-depender	K18409	ATP-depender
evm.model.HIC_ASM_12.892.1	PREDICTED: ubiquiti	Ubiquitin carboxyl-term	K11851	ubiquitin carbo
evm.model.HIC_ASM_11.892	kell blood group glycop	Kell blood group glyco	K06577	Kell blood gro
evm.model.HIC_ASM_4.400.2	galactocerebrosidase is	Galactocerebrosidase O	K01202	galactosylcera
evm.model.HIC_ASM_9.688	interleukin-11 receptor	Interleukin-11 receptor	K05056	interleukin 11
evm.model.HIC_ASM_21.929.3	transcription factor-like	Transcription factor-lik	K09092	transcription f
evm.model.HIC_ASM_15.318	cytochrome b-c1 comp	Cytochrome b-c1 comp	K00417	ubiquinol-cyto

evm.model.HIC_ASM_8.1565	cation channel sperm-a	Cation channel sperm-a	K16889	cation channel
evm.model.HIC_ASM_18.1479	protein BANP isoform	Protein BANP OS=Bos	--	
evm.model.HIC_ASM_3.1175	PREDICTED: eukaryo	Eukaryotic peptide cha	K03265	peptide chain r
evm.model.HIC_ASM_7.472	PREDICTED: diacylgl	Diacylglycerol kinase k	K00901	diacylglycerol
evm.model.HIC_ASM_19.1395	urocanate hydratase-lik	Urocanate hydratase O	K01712	urocanate hydr
evm.model.HIC_ASM_4.1756.1	PREDICTED: synemin	Synemin OS=Homo sa	K10376	desmuslin | (R
evm.model.HIC_ASM_18.1363	PREDICTED: TATA b	TATA box-binding pro	K15214	TATA box-bin
evm.model.HIC_ASM_6.201	PREDICTED: phospha	Phosphatidylinositol-gl	K01127	glycosylphosp
evm.model.HIC_ASM_5.644	PREDICTED: myogen	Myogenic factor 5 OS=	K18484	myogenic fact
evm.model.HIC_ASM_20.1123	cell division cycle prot	Cell division cycle prot	K03350	anaphase-prom
evm.model.HIC_ASM_21.656	LOW QUALITY PRO	Potassium voltage-gate	K04885	potassium volt
evm.model.HIC_ASM_10.1510	fibronectin type III dom	Fibronectin type III dom	K17493	calmodulin-reg
evm.model.HIC_ASM_4.1719	PREDICTED: transien	Transient receptor pote	K04976	transient recep
evm.model.HIC_ASM_5.210	PREDICTED: serine h	Serine hydrolase-like p	K13703	abhydrolase do
evm.model.HIC_ASM_1.862.9	T-lymphocyte activatic	T-lymphocyte activatic	K05413	CD86 antigen
evm.model.HIC_ASM_7.180	adhesion G-protein cou	Adhesion G-protein co	K08451	adhesion G-pr
evm.model.HIC_ASM_19.815.3	parathyroid hormone/p	Parathyroid hormone/p	K04585	parathyroid ho
evm.model.HIC_ASM_3.762_evi	PREDICTED: CMP-N	CMP-N-acetylneurami	K06614	alpha-N-acetyl
evm.model.HIC_ASM_4.1222.2	unnamed protein produ	Leucine-rich repeat-con	K19613	leucine-rich re
evm.model.HIC_ASM_16.28	PREDICTED: guanine	Guanine nucleotide exc	K20685	guanine nucleo
evm.model.HIC_ASM_14.605	PREDICTED: protein	Protein Wnt-8b OS=Ho	K00714	wingless-type
evm.model.HIC_ASM_6.1141.4	F-actin-monooxygenas	[F-actin]-monooxygena	K19947	F-actin monoo
evm.model.HIC_ASM_7.748	sushi repeat-containing	Sushi repeat-containing	K19408	sushi repeat-co
evm.model.HIC_ASM_5.270.1	small G protein signali	Small G protein signali	K20176	small G protei
evm.model.HIC_ASM_11.379	Neurexophilin-1 [Cric	Neurexophilin-1 OS=M	K16656	neurexophilin-
evm.model.HIC_ASM_8.1581	neuronal PAS domain-	Neuronal PAS domain-	K14795	ribosomal RN
evm.model.HIC_ASM_3.938	cocaine- and amphetan	Cocaine- and amphetar	--	
evm.model.HIC_ASM_9.234.1	PREDICTED: nucleop	Nucleoporin NUP188 h	K14311	nuclear pore c
evm.model.HIC_ASM_4.856.2	PREDICTED: NF-kapp	NF-kappa-B inhibitor a	K04734	NF-kappa-B in
evm.model.HIC_ASM_2.592	PREDICTED: collager	Collagen alpha-1(III) c	K19720	collagen, type
evm.model.HIC_ASM_20.1417	pyridoxine-5'-phosphat	Pyridoxine-5'-phosphat	K00275	pyridoxamine
evm.model.HIC_ASM_12.567	tRNA (uracil-5-)-methy	tRNA (uracil-5-)-methy	K15332	tRNA (uracil-5
evm.model.HIC_ASM_19.1137.2	eukaryotic translation i	Eukaryotic translation i	K03259	translation init
evm.model.HIC_ASM_0.1124	PREDICTED: protein S	Protein Shroom3 OS=H	K18625	protein Shroom
evm.model.HIC_ASM_8.345	suppressor of tumorige	Suppressor of tumorige	K08670	suppressor of t
evm.model.HIC_ASM_15.501	metastasis suppressor p	Protein MTSS 1 OS=H	K20128	metastasis sup
evm.model.HIC_ASM_3.538	PWWP domain-contain	PWWP domain-contain	--	
evm.model.HIC_ASM_13.899	pleckstrin homology do	Pleckstrin homology do	--	
evm.model.HIC_ASM_11.439.5	cell division cycle-asso	Cell division cycle-asso	K23408	cell division cy
evm.model.HIC_ASM_11.261	protein piccolo [Felis c	Protein piccolo OS=Ho	K16882	protein piccolo
evm.model.HIC_ASM_9.435	tumor necrosis factor li	Tumor necrosis factor l	K05471	tumor necrosis
evm.model.HIC_ASM_1.38	beta-galactosidase isofo	Beta-galactosidase OS=	K12309	beta-galactosid
evm.model.HIC_ASM_2.24.1	D-2-hydroxyglutarate c	D-2-hydroxyglutarate c	K18204	D-2-hydroxyg
evm.model.HIC_ASM_4.100	PREDICTED: reticulon	Reticulon-1 OS=Homo	K20721	reticulon-1 | (R

evm.model.HIC_ASM_8.503	metabotropic glutamate	Metabotropic glutamat	K04604	metabotropic g
evm.model.HIC_ASM_1.256.1	PREDICTED: ELL-ass	ELL-associated factor	K15186	ELL-associate
evm.model.HIC_ASM_10.789	gap junction alpha-4 pr	Gap junction alpha-4 p	K07613	gap junction a
evm.model.HIC_ASM_6.443.2	G-protein-signaling mo	G-protein-signaling mo	K15838	G-protein sign
evm.model.HIC_ASM_2.158	PREDICTED: secreted	Secreted phosphoprote	--	
evm.model.HIC_ASM_0.885	PREDICTED: protein	Protein ZGRF1 OS=Ho	K10706	senataxin [EC
evm.model.HIC_ASM_12.986	PREDICTED: F-box/W	F-box/WD repeat-conta	K10264	F-box and WD
evm.model.HIC_ASM_10.738	PREDICTED: transme	Transmembrane protein	K15683	NF-X1-type zi
evm.model.HIC_ASM_15.129	PREDICTED: dnaJ hom	DnaJ homolog subfami	K09525	DnaJ homolog
evm.model.HIC_ASM_8.1458.2	tRNA 2'-phosphotransf	tRNA 2'-phosphotransf	K10669	2'-phosphotran
evm.model.HIC_ASM_7.39	PREDICTED: pseudou	Pseudouridine-5'-phosp	K17623	pseudouridine
evm.model.HIC_ASM_3.1024	probable ATP-depende	Probable ATP-depende	K13982	probable ATP-
evm.model.HIC_ASM_18.1535.1	PREDICTED: charged	Charged multivesicular	K12197	charged multiv
evm.model.HIC_ASM_13.150	PREDICTED: V-type p	V-type proton ATPase	K02148	V-type H+-tra
evm.model.HIC_ASM_11.323	PREDICTED: calciton	Calcitonin receptor OS	K04576	calcitonin rece
evm.model.HIC_ASM_2.719	PREDICTED: glutama	Glutamate-rich protein	--	
evm.model.HIC_ASM_5.885	PREDICTED: LOW Q	Protein TESPA1 OS=H	K09079	neurogenic dif
evm.model.HIC_ASM_11.987	GTPase IMAP family m	GTPase IMAP family m	K18626	trichohyalin | (
evm.model.HIC_ASM_17.841	PREDICTED: TSSK6-	TSSK6-activating co-c	--	
evm.model.HIC_ASM_9.10	NMDA receptor synap	NMDA receptor synap	K07359	calcium/calmo
evm.model.HIC_ASM_2.513	origin recognition com	Origin recognition com	K02604	origin recogni
evm.model.HIC_ASM_10.1855	gap junction alpha-8 pr	Gap junction alpha-8 p	K07617	gap junction a
evm.model.HIC_ASM_8.1	PREDICTED: heteroge	Heterogeneous nuclear	K12741	heterogeneous
evm.model.HIC_ASM_3.927	PREDICTED: LOW Q	F-BAR domain only pr	K20042	F-BAR domai
evm.model.HIC_ASM_18.1163	cytochrome b5 type B	Cytochrome b5 type B	K17418	large subunit r
evm.model.HIC_ASM_7.561	PREDICTED: androge	Androgen receptor OS=	K08557	androgen rece
evm.model.HIC_ASM_8.1769	PREDICTED: transme	Transmembrane protein	K19385	transmembran
evm.model.HIC_ASM_15.60	trimethylguanosine syn	Trimethylguanosine sy	K14292	trimethylguan
evm.model.HIC_ASM_6.1378	LOW QUALITY PRO	Utrophin OS=Homo sa	K10366	dystrophin | (F
evm.model.HIC_ASM_2.1891	probable E3 ubiquitin-p	Probable E3 ubiquitin-p	K06058	deltex [EC:2.3
evm.model.HIC_ASM_2.731	kelch-like protein 23 is	Kelch-like protein 23 O	K10460	kelch-like prot
evm.model.HIC_ASM_1.960	PREDICTED: transme	Transmembrane protea	K09638	transmembran
evm.model.HIC_ASM_0.863	neurotrypsin [Acinony	Neurotrypsin OS=Hom	K09624	neurotrypsin [
evm.model.HIC_ASM_20.1449	calcium-binding and co	Calcium-binding and co	K21348	calcium-bindi
evm.model.HIC_ASM_3.1228	PREDICTED: UPF046	UPF0461 protein C5or	--	
evm.model.HIC_ASM_14.1093	PREDICTED: AT-rich	AT-rich interactive dor	K06676	condensin com
evm.model.HIC_ASM_3.1184	LOW QUALITY PRO	Oocyte-specific histone	K18710	histone RNA h
evm.model.HIC_ASM_0.953_evi	B-cell scaffold protein	B-cell scaffold protein	K12230	phosphoinosit
evm.model.HIC_ASM_6.1150	PREDICTED: D-aspar	D-aspartate oxidase OS	K00272	D-aspartate ox
evm.model.HIC_ASM_17.32	PREDICTED: solute ca	Solute carrier family 45	K15379	solute carrier f
evm.model.HIC_ASM_20.287.1	dual specificity mitoge	Dual specificity mitoge	K04432	mitogen-activa
evm.model.HIC_ASM_20.9	protein AATF isoform	Protein AATF OS=Hom	K14782	protein AATF
evm.model.HIC_ASM_7.862	LOW QUALITY PRO	Putative bifunctional U	K07432	beta-1,4-N-ace
evm.model.HIC_ASM_15.485	atpase family aaa doma	ATPase family AAA d	K22531	ATPase family

evm.model.HIC_ASM_2.259	transmembrane 4 L6 fa	Transmembrane 4 L6 f	--	
evm.model.HIC_ASM_6.761	PREDICTED: spermat	Spermatogenesis-assoc	--	
evm.model.HIC_ASM_9.204.4	uncharacterized protein	Telomere length and si	K11703	collagen beta-
evm.model.HIC_ASM_8.828	PREDICTED: synaptot	Synaptotagmin-9 OS=I	K19909	synaptotagmin
evm.model.HIC_ASM_8.296.6	glucose-6-phosphate ex	Glucose-6-phosphate e	K13783	MFS transport
evm.model.HIC_ASM_10.952	KICSTOR complex pro	KICSTOR complex pro	K23298	KICSTOR com
evm.model.HIC_ASM_4.1285	sodium/potassium/calc	Sodium/potassium/calc	K13753	solute carrier f
evm.model.HIC_ASM_21.309	PREDICTED: myosin	Myosin light chain kina	K00907	myosin-light-c
evm.model.HIC_ASM_2.1635.4	exportin-6 isoform X6	Exportin-6 OS=Homo	K04613	vomeronasal 2
evm.model.HIC_ASM_6.1393	LOW QUALITY PRO	Androglobin OS=Hom	K08518	syntaxin-bindi
evm.model.HIC_ASM_19.650.1	PREDICTED: long-cha	Long-chain fatty acid t	K08745	solute carrier f
evm.model.HIC_ASM_8.1103	PREDICTED: tripartite	Tripartite motif-contair	K12020	tripartite motif
evm.model.HIC_ASM_10.1457	PREDICTED: spindle	Spindle assembly abno	K16487	spindle assemb
evm.model.HIC_ASM_0.1039	SPARC-like protein 1 i	SPARC-like protein 1 (	K12819	pre-mRNA-pro
evm.model.HIC_ASM_8.1498.8	sorting nexin-15 [Felis	Sorting nexin-15 OS=F	K17927	sorting nexin-1
evm.model.HIC_ASM_20.1148	spermatogenesis-associ	Spermatogenesis-assoc	K18626	trichohyalin | (
evm.model.HIC_ASM_1.1014	interphotoreceptor mat	Interphotoreceptor mat	K19017	interphotorece
evm.model.HIC_ASM_11.719	serine-rich single-pass	Serine-rich single-pass	--	
evm.model.HIC_ASM_5.209.1	ribosomal rna-processi	Ribosomal RNA-proce	K14545	ribosomal RN.
evm.model.HIC_ASM_17.190	high affinity immunogl	High affinity immunog	K16851	high affinity in
evm.model.HIC_ASM_4.1632.5	PREDICTED: phospho	Phosphopantothenoylcy	K01598	phosphopantot
evm.model.HIC_ASM_12.289	SKI family transcriptio	SKI family transcriptio	K22939	immediate ear
evm.model.HIC_ASM_18.82.4	PREDICTED: protein	Protein phosphatase 1 r	K17457	protein phosph
evm.model.HIC_ASM_21.536.2	adenosine deaminase is	Adenosine deaminase (	K01488	adenosine dea
evm.model.HIC_ASM_11.565	neuropeptide S recepto	Neuropeptide S recepto	K08376	neuropeptide S
evm.model.HIC_ASM_18.1124	LOW QUALITY PRO	Transcription factor E2	K04682	transcription fa
evm.model.HIC_ASM_5.802.4	PREDICTED: methion	Methionine--tRNA liga	K01874	methionyl-tRN
evm.model.HIC_ASM_8.1362	PREDICTED: protein	Protein phosphatase 1 r	K17571	protein phosph
evm.model.HIC_ASM_18.1400	genetic suppressor elen	Genetic suppressor ele	K20045	intersectin | (R
evm.model.HIC_ASM_17.379.1	tyrosine-protein kinase	Tyrosine-protein kinase	K08887	abelson tyrosin
evm.model.HIC_ASM_4.1937	elongation factor-like (	Elongation factor-like (	K14536	ribosome asser
evm.model.HIC_ASM_0.1647	DNA polymerase nu [	DNA polymerase nu O	K16618	DNA polymer
evm.model.HIC_ASM_21.518_ev	TOX high mobility gro	TOX high mobility gro	K18808	toll-like recept
evm.model.HIC_ASM_11.1075	RNA-binding protein 3	RNA-binding protein 3	K12567	titin [EC:2.7.1
evm.model.HIC_ASM_12.996	PREDICTED: mediato	Mediator of RNA poly	K15164	mediator of RI
evm.model.HIC_ASM_11.1004.3	ATP-binding cassette s	ATP-binding cassette s	K05655	ATP-binding c
evm.model.HIC_ASM_17.41	PREDICTED: LEM do	LEM domain-containin	--	
evm.model.HIC_ASM_3.1225	PREDICTED: cation c	Cation channel sperm-a	K16891	cation channel
evm.model.HIC_ASM_2.1352.8	PREDICTED: N-acety	N-acetylglucosamine-1	K01125	N-acetylglucos
evm.model.HIC_ASM_9.1178.1	PREDICTED: ninjurin	Ninjurin-1 OS=Mus mu	K14939	cytochrome P4
evm.model.HIC_ASM_10.1329.1	UPF0690 protein C1or	UPF0690 protein C1or	--	
evm.model.HIC_ASM_19.704.1	single-stranded DNA-b	Single-stranded DNA-b	K08462	adhesion G pro
evm.model.HIC_ASM_11.286	metalloreductase STEA	Metalloreductase STEA	K19876	metalloreducta
evm.model.HIC_ASM_16.617.1	PREDICTED: coiled-c	Coiled-coil domain-con	K18772	C->U-editing (

evm.model.HIC_ASM_18.1497.2	PREDICTED: RING fi	RING finger protein 16	K17044	RING finger p
evm.model.HIC_ASM_21.422	retinoblastoma-like pro	Retinoblastoma-like pr	K04681	retinoblastoma
evm.model.HIC_ASM_13.882	PREDICTED: homeob	Homeobox protein SIX	K19472	homeobox pro
evm.model.HIC_ASM_10.1146	probable proline--tRNA	Probable proline--tRNA	K01881	prolyl-tRNA s
evm.model.HIC_ASM_0.1543	dihydropyrimidinase-re	Dihydropyrimidinase-r	K07528	dihydropyrimi
evm.model.HIC_ASM_2.2144	PREDICTED: cytochro	Cytochrome c oxidase	K18183	cytochrome c
evm.model.HIC_ASM_4.1712	OTU domain-containin	OTU domain-containin	K11860	OTU domain-
evm.model.HIC_ASM_14.989	cadherin-23 isoform X	Cadherin-23 OS=Homo	K06813	cadherin 23 | (
evm.model.HIC_ASM_2.1349	periplakin isoform X1	Periplakin OS=Homo s	K10386	periplakin | (R
evm.model.HIC_ASM_20.609	vitelline membrane out	Vitelline membrane ou	K03131	transcription i
evm.model.HIC_ASM_3.207.1	PREDICTED: unconve	Unconventional myosi	K12559	myosin X | (R
evm.model.HIC_ASM_17.831	tetratricopeptide repeat	Tetratricopeptide repea	K17759	NAD(P)H-hyd
evm.model.HIC_ASM_10.1786	PREDICTED: cathepsi	Cathepsin K OS=Canis	K01371	cathepsin K [E
evm.model.HIC_ASM_19.1.3	PREDICTED: phospho	Phospholipid phosphat	K01080	phosphatidate
evm.model.HIC_ASM_0.23	PREDICTED: neurona	Neuronal acetylcholine	K04808	nicotinic acety
evm.model.HIC_ASM_13.53	PREDICTED: trafficki	Trafficking protein par	K20309	trafficking pro
evm.model.HIC_ASM_9.565	endoplasmic reticulum	Endoplasmic reticulum	K17264	endoplasmic r
evm.model.HIC_ASM_2.742	bile salt export pump is	Bile salt export pump (	K05664	ATP-binding c
evm.model.HIC_ASM_5.630	PREDICTED: leucine-	Leucine-rich repeat an	K17550	protein phosph
evm.model.HIC_ASM_6.689	E3 ubiquitin-protein lig	E3 ubiquitin-protein lig	K10626	E3 ubiquitin-p
evm.model.HIC_ASM_10.1766.1	phosphatidylinositol 4-	Phosphatidylinositol 4-	K00889	1-phosphatidy
evm.model.HIC_ASM_14.340.1	short/branched chain sp	Short/branched chain s	K09478	short/branched
evm.model.HIC_ASM_18.1561	PREDICTED: probable	Histone-lysine N-methy	K20796	histone-lysine
evm.model.CTG_180.14	tumor necrosis factor re	Tumor necrosis factor r	K04722	tumor necrosis
evm.model.HIC_ASM_10.846	ephrin type-A receptor	Ephrin type-A receptor	K08897	Eph receptor A
evm.model.HIC_ASM_8.24	PREDICTED: probable	Probable ATP-depende	K14776	ATP-depender
evm.model.HIC_ASM_21.967.6	ADP-ribosylation facto	ADP-ribosylation facto	K07952	ADP-ribosylat
evm.model.HIC_ASM_11.188	PREDICTED: tRNA-d	tRNA-dihydrouridine(2	K05545	tRNA-dihydro
evm.model.HIC_ASM_3.886	PREDICTED: high aff	High affinity cAMP-sp	K18437	high affinity c
evm.model.HIC_ASM_17.869	PREDICTED: 28S ribo	28S ribosomal protein	K17408	small subunit r
evm.model.HIC_ASM_11.507.1	cyclic AMP-responsive	Cyclic AMP-responsiv	K09047	cyclic AMP-re
evm.model.HIC_ASM_2.1803_ev	PREDICTED: putative	Polypeptide N-acetylga	K00710	polypeptide N
evm.model.HIC_ASM_10.711	serine incorporator 2 is	Serine incorporator 2 (	K16316	serine/threoni
evm.model.HIC_ASM_18.435	ribosome biogenesis pr	Ribosome biogenesis p	K14840	nucleolar prot
evm.model.HIC_ASM_10.1043.1	UMP-CMP kinase isof	UMP-CMP kinase OS=	K13800	UMP-CMP ki
evm.model.HIC_ASM_5.221	PREDICTED: sesquipe	Sesquipedalian-2 OS=I	K22830	essential MCU
evm.model.HIC_ASM_8.1629.1	PREDICTED: AH rece	AH receptor-interacting	K17767	AH receptor-i
evm.model.HIC_ASM_14.152.1	peroxisomal N(1)-acety	Peroxisomal N(1)-acety	K00308	N1-acetylpoly
evm.model.HIC_ASM_20.1242	contactin-associated pr	Contactin-associated p	K07379	contactin asso
evm.model.HIC_ASM_2.612	dnaJ homolog subfamil	DnaJ homolog subfami	K09530	DnaJ homolog
evm.model.HIC_ASM_3.1312	PREDICTED: testis-ex	Testis-expressed protei	--	
evm.model.HIC_ASM_18.1167.2	60S ribosome subunit b	60S ribosome subunit b	K07565	60S ribosome
evm.model.HIC_ASM_16.398	protein FAM124A isof	Protein FAM124A OS=	K09491	stress 70 prote
evm.model.HIC_ASM_12.65	zinc finger protein 516	Zinc finger protein 516	K22411	zinc finger pro

evm.model.HIC_ASM_8.1494	hypothetical protein EI	Membrane-anchored ju	--	
evm.model.HIC_ASM_6.359	mediator of DNA dama	Mediator of DNA dam	K20780	mediator of D
evm.model.HIC_ASM_9.59.3	rab-like protein 6 isofo	Rab-like protein 6 OS=	K04967	transient recep
evm.model.HIC_ASM_9.332	PREDICTED: rab9 eff	Rab9 effector protein v	K20285	Rab9 effector
evm.model.HIC_ASM_3.862	zinc finger FYVE dom	Zinc finger FYVE dom	K04679	MAD, mother
evm.model.HIC_ASM_10.108	PREDICTED: PR dom	Histone-lysine N-meth	K22410	PR domain zir
evm.model.HIC_ASM_12.493.3	receptor-type tyrosine-	Receptor-type tyrosine	K05693	receptor-type t
evm.model.HIC_ASM_3.201_evi	PREDICTED: E3 ubiq	E3 ubiquitin-protein lig	K10659	E3 ubiquitin-p
evm.model.HIC_ASM_4.1576.3	PREDICTED: Bardet-I	Bardet-Biedl syndrome	K16531	Bardet-Biedl s
evm.model.HIC_ASM_4.1491	IQ domain-containing	IQ domain-containing	--	
evm.model.HIC_ASM_13.632	transcription factor LB	Transcription factor LI	K09353	homeobox pro
evm.model.HIC_ASM_20.1435	homeobox protein Hox	Homeobox protein Ho	K09301	homeobox pro
evm.model.HIC_ASM_15.560	PREDICTED: HERV-I	HERV-H LTR-associat	--	
evm.model.HIC_ASM_15.596	zinc finger protein ZF	Zinc finger protein ZF	K09228	KRAB domain
evm.model.HIC_ASM_21.402	cyclic nucleotide-bindi	Cyclic nucleotide-bind	K17532	STE20-related
evm.model.HIC_ASM_19.38.1	PREDICTED: calponir	Calponin-2 OS=Pongo	K16733	Rac GTPase-a
evm.model.HIC_ASM_21.288	beta-defensin 129-like	Beta-defensin 129 OS=	--	
evm.model.HIC_ASM_7.1069.1	MAP7 domain-contain	MAP7 domain-contain	K16807	MAP7 domain
evm.model.HIC_ASM_11.450.11	PREDICTED: mitocho	Mitochondrial assembl	K12734	peptidyl-proly
evm.model.HIC_ASM_15.763	5-oxoprolinase isoform	5-oxoprolinase OS=Ho	K01469	5-oxoprolinase
evm.model.HIC_ASM_21.104	PREDICTED: filensin	Filensin OS=Homo sap	K10378	filensin | (Ref
evm.model.HIC_ASM_2.753	xin actin-binding repea	Xin actin-binding repe	K23026	LIM domain a
evm.model.HIC_ASM_6.1386.5	e3 ubiquitin-protein lig	E3 ubiquitin-protein lig	K15710	E3 ubiquitin-p
evm.model.HIC_ASM_19.1298	glutamate receptor-inte	Glutamate receptor-int	K20251	glutamate rece
evm.model.HIC_ASM_12.850	PREDICTED: leucine-	Leucine-rich repeat-co	K03352	anaphase-pron
evm.model.HIC_ASM_0.1365	PREDICTED: neurona	Neuronal acetylcholine	K04810	nicotinic acety
evm.model.HIC_ASM_14.792.2	2-oxoglutarate dehydro	2-oxoglutarate dehydro	K00164	2-oxoglutarate
evm.model.HIC_ASM_12.890	PREDICTED: uracil-D	Uracil-DNA glycosyla	K03648	uracil-DNA gl
evm.model.HIC_ASM_6.912	regulating synaptic me	Regulating synaptic me	K15291	regulating syn
evm.model.HIC_ASM_6.1133	PREDICTED: armadill	Armadillo repeat-conta	--	
evm.model.HIC_ASM_7.992.1	xaa-Pro aminopeptidas	Xaa-Pro aminopeptidas	K14208	Xaa-Pro amino
evm.model.HIC_ASM_2.1188	PREDICTED: methion	Methionine-R-sulfoxid	K07305	peptide-methio
evm.model.HIC_ASM_13.836	PREDICTED: ankyrin	Ankyrin repeat and SO	K10325	ankyrin repeat
evm.model.HIC_ASM_0.854	Hypothetical predicted	Uncharacterized protei	--	
evm.model.HIC_ASM_19.31.2	PREDICTED: R3H do	R3H domain-containin	K05641	ATP-binding c
evm.model.HIC_ASM_1.750	dynamin-like 120 kDa	Dynamin-like 120 kDa	K17079	optic atrophy
evm.model.HIC_ASM_21.348	Long palate, lung and i	BPI fold-containing fa	--	
evm.model.HIC_ASM_20.673.3	PREDICTED: fructosa	Fructosamine-3-kinase	K15522	protein-fructos
evm.model.HIC_ASM_1.800	PREDICTED: LOW Q	Choline-phosphate cyti	K00968	choline-phosp
evm.model.HIC_ASM_5.1588	coiled-coil domain-con	Coiled-coil domain-co	K20478	golgin subfam
evm.model.HIC_ASM_6.544	signal peptide, CUB an	Signal peptide, CUB a	K06825	fibrillin 1 | (Re
evm.model.HIC_ASM_20.1636	integrator complex sub	Integrator complex sub	K13139	integrator com
evm.model.HIC_ASM_1.100	C-C chemokine recept	C-C chemokine recept	K04183	C-C chemokin
evm.model.HIC_ASM_18.331.1	mediator of RNA poly	Mediator of RNA poly	K15168	mediator of R

evm.model.HIC_ASM_6.90	PREDICTED: synapto	Synaptonemal complex	K19529	synaptonemal
evm.model.HIC_ASM_20.1637	LOW QUALITY PRO	Fanconi anemia group	K15362	fanconi anemi
evm.model.HIC_ASM_5.233	LOW QUALITY PRO	Sterol regulatory eleme	K09107	sterol regulato
evm.model.HIC_ASM_3.685	vesicular integral-mem	Vesicular integral-men	K10082	lectin, mannos
evm.model.HIC_ASM_12.727.2	PREDICTED: serine/tl	Serine/threonine-protei	K06641	serine/threonii
evm.model.HIC_ASM_10.643	PREDICTED: sestrin-2	Sestrin-2 OS=Mus mus	K20394	sestrin 2 | (Ref
evm.model.HIC_ASM_10.1227	leptin receptor-like [L	Leptin receptor OS=Su	K05062	leptin receptor
evm.model.HIC_ASM_11.780	solute carrier family 13	Solute carrier family 13	K14444	solute carrier f
evm.model.HIC_ASM_13.674	rab11 family-interactin	Rab11 family-interacti	K12484	Rab11 family-
evm.model.HIC_ASM_11.237	tyrosine-protein phospl	Tyrosine-protein phosp	K18024	tyrosine-protei
evm.model.HIC_ASM_6.897	collagen alpha-1(IX) cl	Collagen alpha-1(IX) c	K08131	collagen, type
evm.model.HIC_ASM_3.1241.4	transcription factor 7 is	Transcription factor 7 (	K02620	transcription f
evm.model.HIC_ASM_4.324.1	probable ergosterol bio	Probable ergosterol bic	--	
evm.model.HIC_ASM_5.1604	supervillin isoform X8	Supervillin OS=Homo	K10369	supervillin | (F
evm.model.HIC_ASM_10.631.1	syntaxin-12 [Felis catu	Syntaxin-12 OS=Homo	K13813	syntaxin 12/13
evm.model.HIC_ASM_13.543	T-cell surface glycopro	T-cell surface glycopro	K06458	T-cell surface
evm.model.HIC_ASM_10.1382.1	probable ATP-depende	Probable ATP-depende	K15271	ATP-depender
evm.model.HIC_ASM_5.1394	microfibrillar-associate	Microfibrillar-associate	--	
evm.model.HIC_ASM_21.547	STK4 protein, partial |	Serine/threonine-protei	K04411	serine/threonii
evm.model.HIC_ASM_4.1174	abscission/NoCut chec	Abscission/NoCut chec	K12182	hepatocyte grc
evm.model.HIC_ASM_0.64	pleckstrin homology do	Pleckstrin homology do	K12229	dual adapter fo
evm.model.HIC_ASM_0.443	progonadoliberin-1 [E	Progonadoliberin-1 OS	K05252	gonadotropin-
evm.model.HIC_ASM_10.980	kinesin-like protein KI	Kinesin-like protein KI	K10393	kinesin family
evm.model.HIC_ASM_6.778	PREDICTED: calcipre	Calcipressin-2 OS=Por	K17903	calcipressin-2
evm.model.HIC_ASM_5.192_evi	signal peptide, CUB an	Signal peptide, CUB ai	K06825	fibrillin 1 | (Re
evm.model.HIC_ASM_17.969	PREDICTED: protein (	Protein dispatched hon	K10649	tripartite motif
evm.model.HIC_ASM_17.845	protein SMG5 isoform	Protein SMG5 OS=Ho	K11125	protein SMG5
evm.model.HIC_ASM_11.384	von Willebrand factor	von Willebrand factor	K20994	Notch 2 | (Ref
evm.model.HIC_ASM_9.301_evi	solute carrier family 2,	Solute carrier family 2,	K08145	MFS transport
evm.model.HIC_ASM_20.377	Hypothetical predicted	Cytospin-B OS=Homo	K23028	cytospin | (Ref
evm.model.HIC_ASM_20.1068	E3 ubiquitin-protein lig	E3 ubiquitin-protein lig	K04678	E3 ubiquitin li
evm.model.HIC_ASM_16.227.1	LOW QUALITY PRO	Endothelin receptor typ	K04198	endothelin rec
evm.model.HIC_ASM_1.509	intraflagellar transport	Intraflagellar transport	K19678	intraflagellar t
evm.model.HIC_ASM_4.1236	tRNA wybutosine-synt	tRNA wybutosine-synt	K15451	tRNA wybutos
evm.model.HIC_ASM_2.916.3	PREDICTED: DNA an	DNA annealing helicas	K14440	SWI/SNF-rela
evm.model.HIC_ASM_14.1024	apoptosis-inducing fact	Apoptosis-inducing fac	K22745	apoptosis-indu
evm.model.HIC_ASM_13.467	glycerol-3-phosphate a	Glycerol-3-phosphate a	K00629	glycerol-3-pho
evm.model.HIC_ASM_20.180	tumor suppressor candi	Trafficking regulator o	K06566	interferon indu
evm.model.HIC_ASM_21.343	PREDICTED: BPI folc	BPI fold-containing fan	K05399	lipopolysaccha
evm.model.HIC_ASM_2.903_evi	thrombospondin type-1	Thrombospondin type-	K19720	collagen, type
evm.model.HIC_ASM_9.1086	PREDICTED: tyrosine	Tyrosine-protein kinase	K05123	receptor tyrosi
evm.model.HIC_ASM_6.1024	PREDICTED: midasin	Midasin OS=Homo sap	K14572	midasin | (Ref
evm.model.HIC_ASM_18.1170.2	beta-2-syntrophin [Fel	Beta-2-syntrophin OS=	K12196	vacuolar prote
evm.model.HIC_ASM_12.294	PREDICTED: alpha-2,	Alpha-2,8-sialyltransfe	K03369	alpha-N-acety

evm.model.HIC_ASM_2.1652	PREDICTED: apolipo|	Apolipoprotein B rece|	K14572 midasin | (Ref
evm.model.HIC_ASM_3.129.2	mediator of rna polyme	Mediator of RNA poly	K15151 mediator of R
evm.model.HIC_ASM_2.226	g-protein coupled rece|	G-protein coupled rece	K08413 G protein-cou
evm.model.HIC_ASM_18.1532	copine-7 isoform X4 [	Copine-7 OS=Rattus n	K20493 leucine-rich re
evm.model.HIC_ASM_13.252	nuclear receptor coacti	Nuclear receptor coacti	K09101 nuclear recept
evm.model.HIC_ASM_5.1743.1	PREDICTED: heat sho	Heat shock 70 kDa pro	K03283 heat shock 70
evm.model.HIC_ASM_10.83	membrane metallo-end	Membrane metallo-end	K08635 neprilysin [EC
evm.model.HIC_ASM_5.1673	armadillo repeat-contai	Armadillo repeat-conta	K10056 junction plako
evm.model.HIC_ASM_18.691	PREDICTED: coiled-c	Coiled-coil domain-co	K19990 chromogranin-
evm.model.HIC_ASM_6.1574.5	PREDICTED: ribosom	Ribosomal protein S6	K04373 ribosomal prot
evm.model.HIC_ASM_0.999	hematopoietic prostagl	Hematopoietic prostag	K04097 prostaglandin-
evm.model.HIC_ASM_14.1026	collagen alpha-1(XIII)	Collagen alpha-1(XIII)	K16617 collagen, type
evm.model.HIC_ASM_9.13.5	NADPH oxidase activa	NADPH oxidase activa	K21432 NADPH oxida
evm.model.HIC_ASM_0.97	PREDICTED: potassiu	Potassium channel sub	K05274 potassium larg
evm.model.HIC_ASM_6.158	PREDICTED: lysine-s	Lysine-specific histone	K19413 lysine-specific
evm.model.HIC_ASM_9.449	PREDICTED: collage	Collagen alpha-1(XXV	K19721 collagen, type
evm.model.HIC_ASM_18.695.2	heterogeneous nuclear	Heterogeneous nuclear	K13159 heterogeneous
evm.model.HIC_ASM_8.1354.6	triokinase/FMN cyclas	Triokinase/FMN cycla	K00863 triose/dihydro
evm.model.HIC_ASM_7.513.2	PREDICTED: DNA-(a	DNA-(apurinic or apyr	K10772 AP endonucle
evm.model.HIC_ASM_21.189	PREDICTED: AP-5 co	AP-5 complex subunit	K19024 AP-5 complex
evm.model.HIC_ASM_6.365	hypothetical protein D	Uncharacterized protei	--
evm.model.HIC_ASM_7.474	protein Shroom4 [Feli	Protein Shroom4 OS=I	K18625 protein Shroo
evm.model.HIC_ASM_10.1230.1	PREDICTED: SH3-co	SH3-containing GRB2	K20065 SH3-containin
evm.model.HIC_ASM_10.1153	barttin [Acinonyx juba	Barttin OS=Homo sapi	K19331 barttin | (RefS
evm.model.HIC_ASM_9.548	glutamate receptor ion	Glutamate receptor ion	K05213 glutamate rece
evm.model.HIC_ASM_12.469	PREDICTED: disks la	Disks large-associated	K15008 discs, large-as
evm.model.HIC_ASM_13.634	coiled-coil domain-con	Coiled-coil domain-co	K16865 programmed c
evm.model.HIC_ASM_1.176	acidic amino acid deca	Acidic amino acid deca	K18966 sulfinoalanine
evm.model.HIC_ASM_0.841	PREDICTED: LOW Q	Pyroglutamylated RF-a	K08378 pyroglutamyla
evm.model.HIC_ASM_10.1242.2	protein wntless homolo	Protein wntless homolo	--
evm.model.HIC_ASM_10.204.2	ERBB receptor feedba	ERBB receptor feedba	K08886 activated CDC
evm.model.HIC_ASM_11.981	replication initiator 1 is	Replication initiator 1	K09228 KRAB domain
evm.model.HIC_ASM_18.710	cation channel sperm-a	Cation channel sperm-	K16894 cation channel
evm.model.HIC_ASM_10.1570	DNA damage-regulate	DNA damage-regulate	K21956 DNA damage-
evm.model.HIC_ASM_4.1731	probable threonine--tR	Threonine--tRNA ligas	K01868 threonyl-tRNA
evm.model.HIC_ASM_14.1038	putative hexokinase HI	Putative hexokinase H	K00844 hexokinase [E
evm.model.HIC_ASM_12.1071	PREDICTED: acyl-Co	Acyl-CoA dehydrogen	K11729 acyl-CoA deh
evm.model.HIC_ASM_1.769	PREDICTED: transme	Transmembrane protei	K05543 tRNA-dihydro
evm.model.HIC_ASM_19.802	leucine-rich repeat-con	Leucine-rich repeat-co	K16175 protein scribb
evm.model.HIC_ASM_6.1494	radial spoke head prote	Radial spoke head prot	--
evm.model.HIC_ASM_19.1267	EF-hand calcium-bindi	EF-hand calcium-bindi	--
evm.model.HIC_ASM_15.100	chromodomain-helicas	Chromodomain-helicas	K14437 chromodomai
evm.model.HIC_ASM_9.324	PREDICTED: target o	Target of rapamycin co	K20410 target of rapa
evm.model.CTG_509.18	acetylserotonin O-metl	Acetylserotonin O-met	K00543 acetylserotoni

evm.model.HIC_ASM_0.276	ankyrin repeat and socs	Ankyrin repeat and SO	K10327	ankyrin repeat
evm.model.HIC_ASM_20.915.1	PREDICTED: SAP30-	SAP30-binding protein	K12878	THO complex
evm.model.HIC_ASM_14.1010	perforin-1 isoform X1	Perforin-1 OS=Homo s	K07818	perforin 1 | (R
evm.model.HIC_ASM_5.1838	-anhydro-d-fructose rec	1,5-anhydro-D-fructose	K13981	1,5-anhydro-D
evm.model.HIC_ASM_6.394	g patch domain and anl	G patch domain and an	K10324	ankyrin repeat
evm.model.HIC_ASM_5.362	PREDICTED: 2-amino	2-amino-3-ketobutyrate	K00639	glycine C-acet
evm.model.HIC_ASM_11.192	cAMP-dependent prote	cAMP-dependent prote	K04739	cAMP-depend
evm.model.HIC_ASM_8.1148	tetraspanin-18 isoform	Tetraspanin-18 OS=Hc	K17353	tetraspanin-18
evm.model.HIC_ASM_19.543.1	paralemmin-3 [Acinor	Paralemmin-3 OS=Mu	K16519	A-kinase anch
evm.model.HIC_ASM_19.325.2	PREDICTED: KN mot	KN motif and ankyrin	K22808	KN motif and
evm.model.HIC_ASM_5.795.5	PREDICTED: probabl	Probable E3 ubiquitin-	K06058	deltex [EC:2.3
evm.model.HIC_ASM_18.1151	zinc finger homeobox	Zinc finger homeobox	K09378	AT-binding tr
evm.model.HIC_ASM_2.1921.2	zinc finger HIT domair	Zinc finger HIT domai	K11663	zinc finger HI
evm.model.HIC_ASM_10.519	myomesin-3 isoform X	Myomesin-3 OS=Hom	K12567	titin [EC:2.7.1
evm.model.HIC_ASM_1.572.1	solute carrier family 2,	Solute carrier family 2,	K07593	MFS transport
evm.model.HIC_ASM_6.788	PREDICTED: meprin	Meprin A subunit alph	K01395	meprin A [EC
evm.model.HIC_ASM_19.972	protein phosphatase 1M	Protein phosphatase 1M	K17507	protein phosph
evm.model.HIC_ASM_10.10.3	PREDICTED: tyrosine	Tyrosine-protein phosp	K07293	tyrosine-protei
evm.model.HIC_ASM_17.447	phosphatidylinositol N	Phosphatidylinositol N	K03859	phosphatidylin
evm.model.HIC_ASM_4.1700	gamma-aminobutyric a	Gamma-aminobutyric a	K05175	gamma-amino
evm.model.HIC_ASM_5.1132	protein kinase C-bindir	Protein kinase C-bindi	K22017	mucin-4 | (Ref
evm.model.HIC_ASM_16.36	probable phospholipid-	Probable phospholipid-	K01530	phospholipid-t
evm.model.HIC_ASM_9.460.1	DNA polymerase epsil	DNA polymerase epsil	K02326	DNA polymer
evm.model.HIC_ASM_8.1084.1	catalase [Peromyscus	Catalase OS=Canis lup	K03781	catalase [EC:1
evm.model.HIC_ASM_2.314	PREDICTED: acid-sen	Acid-sensing ion chanr	K04831	acid-sensing io
evm.model.HIC_ASM_8.157	sodium/potassium-tran	Sodium/potassium-tran	K01538	sodium/potass
evm.model.HIC_ASM_1.1203	protein downstream ne	Quinone oxidoreductas	K10133	tumor protein
evm.model.HIC_ASM_21.807.7	PREDICTED: syntaxir	Syntaxin-16 OS=Mus r	K08489	syntaxin 16 | (
evm.model.HIC_ASM_14.440.1	probable trna pseudour	Probable tRNA pseudo	K03177	tRNA pseudou
evm.model.HIC_ASM_5.1608	poly(A) RNA polymer	Poly(A) RNA polymer	K18060	poly(A) RNA
evm.model.HIC_ASM_13.744.2	PREDICTED: nuclear	Nuclear nucleic acid-bi	K12592	exosome comp
evm.model.HIC_ASM_0.1131.4	ecto-adp-ribosyltransfe	Ecto-ADP-ribosyltrans	K00775	ADP-ribosyltr
evm.model.HIC_ASM_3.738	unnamed protein produ	Ephrin-A5 OS=Mus m	K05462	ephrin-A | (Re
evm.model.HIC_ASM_9.242.1	putative methyltransfer	Putative methyltransfer	K09142	uncharacterize
evm.model.HIC_ASM_0.220	ankyrin repeat domain-	Ankyrin repeat domain	K04984	transient recep
evm.model.HIC_ASM_20.226.1	PREDICTED: SET and	SET and MYND doma	K11426	SET and MYN
evm.model.HIC_ASM_10.1676.3	tripartite motif-contain	Tripartite motif-contai	K12021	tripartite moti
evm.model.HIC_ASM_13.507	PREDICTED: DNA-di	DNA-directed RNA po	K03002	DNA-directed
evm.model.HIC_ASM_15.805.4	PREDICTED: LOW Q	ATP-dependent DNA I	K10730	ATP-depende
evm.model.HIC_ASM_14.877	pulmonary surfactant-a	Pulmonary surfactant-a	K10067	pulmonary sur
evm.model.HIC_ASM_18.494	basic helix-loop-helix a	Basic helix-loop-helix	--	
evm.model.HIC_ASM_0.1334	protein YIPF7 [Felis c	Protein YIPF7 OS=Bo	K20363	protein YIPF5
evm.model.HIC_ASM_2.160	PREDICTED: LOW Q	Transient receptor pote	K04983	transient recep
evm.model.HIC_ASM_2.789	T-box brain protein 1 |	T-box brain protein 1 (	K10174	T-box brain p

evm.model.HIC_ASM_1.1409	lanosterol synthase iso	Lanosterol synthase OS	K01852	lanosterol synt
evm.model.HIC_ASM_2.616	ceramide kinase-like pr	Ceramide kinase-like p	K19602	ceramide kinas
evm.model.HIC_ASM_9.459	PREDICTED: unchara	Uncharacterized protei	K07524	regulator of G
evm.model.HIC_ASM_10.278	uncharacterized protein	Uncharacterized protei	K16489	protein SFI1 |
evm.model.HIC_ASM_2.1782	beta-glucuronidase isof	Beta-glucuronidase OS	K01195	beta-glucuroni
evm.model.HIC_ASM_6.859.1	PREDICTED: BAG fa	BAG family molecular	K09556	BCL2-associat
evm.model.HIC_ASM_18.510	DNA-directed RNA po	DNA-directed RNA po	--	
evm.model.HIC_ASM_17.636	PREDICTED: UDP-N-	UDP-N-acetylhexosam	K00972	UDP-N-acetyl
evm.model.HIC_ASM_2.428	protein unc-80 homolo	Protein unc-80 homolo	--	
evm.model.HIC_ASM_8.1253	PREDICTED: apelin re	Apelin receptor OS=M	K04174	apelin recepto
evm.model.HIC_ASM_20.1338.5	unnamed protein produ	Thyroid hormone recep	K05547	thyroid hormo
evm.model.HIC_ASM_21.320.1	transmembrane 9 super	Transmembrane 9 supe	K17086	transmembran
evm.model.HIC_ASM_8.286.1	sialate O-acetylesterase	Sialate O-acetylesteras	K05970	sialate O-acety
evm.model.HIC_ASM_5.631	PREDICTED: putative	Putative tetraspanin-19	K17358	tetraspanin-19
evm.model.HIC_ASM_4.495	serine/threonine-protei	Serine/threonine-protei	K15426	serine/threonin
evm.model.HIC_ASM_18.299	putative uncharacterize	Putative uncharacterize	--	
evm.model.HIC_ASM_3.1091	death ligand signal enh	DAP3-binding cell dea	K14026	SEL1 protein |
evm.model.HIC_ASM_11.758	PREDICTED: UDP-xy	UDP-xylose and UDP-	K15278	solute carrier f
evm.model.HIC_ASM_9.1028	PREDICTED: pumilio	Pumilio homolog 3 OS	K14844	pumilio homol
evm.model.HIC_ASM_18.764	CAP-Gly domain-conta	NF-kappa-B inhibitor c	K14214	NF-kappa-B in
evm.model.HIC_ASM_19.1207	LHFPL tetraspan subfa	LHFPL tetraspan subfa	K18631	tektin-4 | (Gen
evm.model.HIC_ASM_12.891	PREDICTED: DNA ox	DNA oxidative demeth	K10859	DNA oxidativ
evm.model.HIC_ASM_0.355.5	glutamate-rich protein	Glutamate-rich protein	K06824	nephronectin |
evm.model.HIC_ASM_20.316	dexamethasone-induce	Dexamethasone-induce	K07843	RAS, dexamet
evm.model.HIC_ASM_4.1495	SKI family transcriptio	SKI family transcriptio	K22939	immediate ear
evm.model.HIC_ASM_0.746.3	type II inositol 3,4-bisp	Type II inositol 3,4-bis	K01109	inositol polypl
evm.model.HIC_ASM_0.1163	PREDICTED: ras asso	Ras association domain	K09854	Ras associatio
evm.model.HIC_ASM_6.547.4	differentially expressed	Differentially expresse	K23213	paired amphip
evm.model.HIC_ASM_3.605	serine/threonine-protei	Serine/threonine-protei	K08837	serine/threonin
evm.model.HIC_ASM_5.934	cyclic AMP-dependent	Cyclic AMP-dependen	K09045	activating tran
evm.model.HIC_ASM_19.136.1	PREDICTED: thimet o	Thimet oligopeptidase	K01392	thimet oligope
evm.model.HIC_ASM_12.555	protein DGCR6L isofo	Protein DGCR6 OS=H	--	
evm.model.HIC_ASM_4.413	PREDICTED: echinod	Echinoderm microtubu	K18597	echinoderm m
evm.model.HIC_ASM_4.1588	PREDICTED: TBC1 d	TBC1 domain family n	K19945	TBC1 domain
evm.model.HIC_ASM_10.1559	prokineticin-1 [Zaloph	Prokineticin-1 OS=Hor	--	
evm.model.HIC_ASM_12.682	unconventional myosin	Unconventional myosi	K10362	myosin XVIII
evm.model.HIC_ASM_10.1081.1	tetratricopeptide repeat	Tetratricopeptide repea	K11155	diacylglycerol
evm.model.HIC_ASM_13.689	PREDICTED: dysferlin	Dysferlin OS=Bos taur	K18261	dysferlin | (Re
evm.model.HIC_ASM_2.2073	PREDICTED: AP-5 co	AP-5 complex subunit	K19025	AP-5 complex
evm.model.HIC_ASM_10.1274	LIM/homeobox protein	LIM/homeobox protein	K09375	LIM homeobo
evm.model.HIC_ASM_2.866.1	enhancer of polycomb	Enhancer of polycomb	K11322	enhancer of po
evm.model.HIC_ASM_12.403	histamine H4 receptor	Histamine H4 receptor	K04152	histamine rece
evm.model.HIC_ASM_12.1126	zinc finger cchc domai	Zinc finger CCHC dom	K13128	zinc finger CC
evm.model.HIC_ASM_12.221	PREDICTED: coiled-c	Coiled-coil domain-con	K22031	myocardial zo

evm.model.HIC_ASM_10.1561	PREDICTED: potassiu	Potassium voltage-gate	K04881	potassium volt
evm.model.HIC_ASM_9.241.4	kynurenine--oxoglutara	Kynurenine--oxoglutar	K00816	kynurenine---c
evm.model.HIC_ASM_4.387	thyrotropin receptor-lik	Thyrotropin receptor C	K04249	thyrotropin rec
evm.model.HIC_ASM_7.1334.1	H/ACA ribonucleoprot	H/ACA ribonucleoprot	K11131	H/ACA ribonu
evm.model.HIC_ASM_21.832	LOW QUALITY PRO'	Phosphatase and actin r	K17594	phosphatase ar
evm.model.HIC_ASM_17.174	LIM/homeobox protein	LIM/homeobox protein	K09373	LIM homeobo
evm.model.HIC_ASM_14.1131.4	TAF5-like RNA polym	TAF5-like RNA polym	K03130	transcription in
evm.model.HIC_ASM_0.420_evi	phosphatidylethanolam	Phosphatidylethanolam	K12367	dedicator of cy
evm.model.HIC_ASM_2.788	sodium-driven chloride	Sodium-driven chloride	K13861	solute carrier f
evm.model.HIC_ASM_7.230_evi	dna polymerase alpha c	DNA polymerase alpha	K02320	DNA polymer
evm.model.HIC_ASM_13.746.3	ewing's tumor-associate	Ewing's tumor-associat	--	
evm.model.HIC_ASM_20.231	PREDICTED: diphthar	2-(3-amino-3-carboxyp	K07561	2-(3-amino-3-c
evm.model.HIC_ASM_5.1448	acrosin-binding protein	Acrosin-binding protei	K11346	inhibitor of gr
evm.model.HIC_ASM_0.1034	matrix extracellular ph	Matrix extracellular ph	--	
evm.model.HIC_ASM_20.1412	leucine-rich repeat-con	Leucine-rich repeat-co	K19753	protein TilB | (
evm.model.HIC_ASM_5.697	receptor-type tyrosine-j	Receptor-type tyrosine-	K05694	receptor-type t
evm.model.HIC_ASM_19.331	LOW QUALITY PRO'	Fibrillin-3 OS=Homo s	K23342	fibrillin 2/3 | (
evm.model.HIC_ASM_4.1510	PREDICTED: NADPH	NADPH oxidase 5 OS=	K21424	NADPH oxida
evm.model.HIC_ASM_4.1245	PREDICTED: LOW Q	Cation channel sperm-a	K16890	cation channel
evm.model.HIC_ASM_9.500_evi	PREDICTED: muscle,	Muscle, skeletal recept	K05129	muscle, skelet
evm.model.HIC_ASM_12.1037	PREDICTED: sodium/	Mitochondrial sodium/	K13754	solute carrier f
evm.model.HIC_ASM_9.844	interleukin-33 [Neomo	Interleukin-33 OS=Car	K12967	interleukin 33
evm.model.HIC_ASM_15.558	otoconin-90 isoform X	Otoconin-90 OS=Mus	K01047	secretory phos
evm.model.HIC_ASM_8.367.4	PREDICTED: vacuolar	Vacuolar protein sortin	K18466	vacuolar prote
evm.model.HIC_ASM_11.525	maturin isoform X4 [C	Maturin OS=Homo sap	K08051	pleckstrin hom
evm.model.HIC_ASM_8.892.3	PREDICTED: alpha-pa	Alpha-parvin OS=Hom	K06275	parvin | (RefSe
evm.model.HIC_ASM_2.544	E3 ubiquitin-protein lig	E3 ubiquitin-protein lig	K12168	E3 ubiquitin-p
evm.model.HIC_ASM_2.2012.1	PREDICTED: actin-rel	Actin-related protein 2/	K05757	actin related p
evm.model.HIC_ASM_18.1118	tumor necrosis factor re	Tumor necrosis factor r	K03171	tumor necrosis
evm.model.HIC_ASM_6.677.3	PREDICTED: G1/S-sp	G1/S-specific cyclin-D	K10152	G1/S-specific
evm.model.HIC_ASM_9.292	PREDICTED: probable	Probable peptidyl-tRN/	K01056	peptidyl-tRNA
evm.model.HIC_ASM_11.342	PREDICTED: cytoplas	Cytoplasmic dynein 1 i	K10415	dynein interme
evm.model.HIC_ASM_21.338	EF-hand calcium-bindi	WD repeat-containing	K02988	small subunit r
evm.model.HIC_ASM_4.1298_ev	PREDICTED: N-acety	N-acetylgalactosamine	K18674	N-acetylgalact
evm.model.HIC_ASM_7.1018	FERM domain-contain	FERM domain-contain	K17477	FERM, RhoGI
evm.model.HIC_ASM_7.828	protein PIH1D3 [Felis	Protein PIH1D3 OS=H	--	
evm.model.HIC_ASM_17.673.1	PREDICTED: NADH	NADH dehydrogenase	K03935	NADH dehydr
evm.model.HIC_ASM_20.340.5	mitochondrial dynamic	Mitochondrial dynamic	K22830	essential MCU
evm.model.HIC_ASM_9.1059.3	plasminogen receptor (	Plasminogen receptor (	--	
evm.model.HIC_ASM_5.1014	PREDICTED: bridging	Bridging integrator 2 C	K20119	bridging integr
evm.model.HIC_ASM_20.1082	Sodium channel protein	Sodium channel protein	K04836	voltage-gated s
evm.model.HIC_ASM_19.633.1	Usher syndrome type-1	Usher syndrome type-1	K21881	Usher syndrom
evm.model.HIC_ASM_8.970	unnamed protein produ	Palmitoyltransferase ZI	K20032	palmitoyltrans
evm.model.HIC_ASM_4.1628.2	unnamed protein produ	Protein FAM219B OS=	--	

evm.model.HIC_ASM_8.1777.5	ras association domain	Ras association domair	K09855	Ras associatio
evm.model.HIC_ASM_5.1827.2	PREDICTED: ankyrin	Ankyrin repeat and SO	K10335	ankyrin repeat
evm.model.HIC_ASM_6.1443	PREDICTED: nesprin-	Nesprin-1 OS=Homo s	K19326	nesprin-1 | (Re
evm.model.HIC_ASM_14.994	PREDICTED: netrin re	Netrin receptor UNC5I	K07521	netrin receptor
evm.model.HIC_ASM_17.422	PREDICTED: probable	G-protein coupled rece	K08407	G protein-coup
evm.model.HIC_ASM_0.1627	nucleolar protein 14 iso	Nucleolar protein 14 O	K14766	nucleolar prot
evm.model.HIC_ASM_8.1095	CD44 antigen isoform	CD44 antigen OS=Hor	K06256	CD44 antigen
evm.model.HIC_ASM_6.1670.3	constitutive coactivator	Constitutive coactivato	K08867	WNK lysine d
evm.model.HIC_ASM_20.1065	nucleosome-remodelin	Nucleosome-remodelir	K11728	nucleosome-re
evm.model.HIC_ASM_3.303.2	LOW QUALITY PRO'	FYN-binding protein 1	K17698	FYN binding p
evm.model.HIC_ASM_2.882	zinc finger E-box-bindi	Zinc finger E-box-bind	K09299	zinc finger ho
evm.model.HIC_ASM_8.687.5	PREDICTED: post-GP	Post-GPI attachment to	K15263	cell growth-re
evm.model.HIC_ASM_2.496	cyclin-dependent kinas	Cyclin-dependent kinas	K15594	cyclin-depend
evm.model.HIC_ASM_9.759	uncharacterized proteir	Izumo sperm-egg fusio	--	
evm.model.HIC_ASM_20.1481.1	unnamed protein produ	Solute carrier family 3:	K15275	solute carrier f
evm.model.HIC_ASM_2.368	probable hydrolase PN	Probable hydrolase PN	K01069	hydroxyacylgl
evm.model.HIC_ASM_16.350	seven in absentia homc	Seven in absentia hom	K22256	seven in absen
evm.model.HIC_ASM_8.263	PREDICTED: unchara	Jhy protein homolog O	K22909	zinc finger CC
evm.model.HIC_ASM_18.527	PREDICTED: apolipo	Apolipoprotein C-II OS	K22287	apolipoprotein
evm.model.HIC_ASM_19.740.1	PREDICTED: DNA-bi	DNA-binding protein F	K08062	regulatory fact
evm.model.HIC_ASM_19.1203	SLIT-ROBO Rho GTP	SLIT-ROBO Rho GTP	K07526	SLIT-ROBO F
evm.model.HIC_ASM_20.304	mitochondrial cardiolip	Mitochondrial cardioli	K16862	mitochondrial
evm.model.HIC_ASM_14.185.2	Hypothetical predicted	Leucine-rich repeat-co	K14837	nucleolar prot
evm.model.HIC_ASM_5.458	tRNA-splicing ligase R	tRNA-splicing ligase R	K14415	tRNA-splicing
evm.model.HIC_ASM_13.548	receptor expression-enl	Receptor expression-er	K17338	receptor expre
evm.model.HIC_ASM_0.1431	protein sel-1 homolog 1	Protein sel-1 homolog 1	K14026	SEL1 protein |
evm.model.HIC_ASM_8.1539.4	gtpase-activating prote	Signal-induced prolifer	K08013	signal-induced
evm.model.HIC_ASM_3.1381	PREDICTED: probable	3'-5' RNA helicase YT	K20099	ATP-depender
evm.model.HIC_ASM_8.676	PREDICTED: nuclear	Nuclear mitotic appara	K16808	nuclear mitoti
evm.model.HIC_ASM_10.1269	PREDICTED: glutama	Glutamate-rich protein	K19721	collagen, type
evm.model.HIC_ASM_0.1527.1	syntaxin-18 isoform X	Syntaxin-18 OS=Homo	K08492	syntaxin 18 | (
evm.model.HIC_ASM_18.1254.4	C-type lectin domain fa	C-type lectin domain fa	K22682	c-type lectin d
evm.model.HIC_ASM_2.970	PREDICTED: tyrosine	Tyrosine-protein phosp	K18037	tyrosine-protei
evm.model.HIC_ASM_8.947	protein saal1-like [Lyr	Protein SAAL1 OS=Ho	K00502	tryptophan 5-n
evm.model.CTG_1511.9	PREDICTED: aryl hyd	Aryl hydrocarbon recep	K09094	aryl hydrocarb
evm.model.HIC_ASM_13.871	Hypothetical predicted	Transmembrane protei	--	
evm.model.HIC_ASM_7.511	LOW QUALITY PRO'	Trophinin OS=Homo s	K12464	melanoma-ass
evm.model.HIC_ASM_20.1610	Hypothetical predicted	Uncharacterized protei	K16943	septin 4 | (Ref
evm.model.HIC_ASM_8.1786.1	anoctamin-9 isoform X	Anoctamin-9 OS=Hom	K19503	anoctamin-9 |
evm.model.HIC_ASM_5.222.1	PREDICTED: alpha-N	Alpha-N-acetylgalacto	K01204	alpha-N-acety
evm.model.HIC_ASM_17.929	uncharacterized proteir	Uncharacterized protei	--	
evm.model.HIC_ASM_9.696	PREDICTED: dynein i	Dynein intermediate ch	K10409	dynein interm
evm.model.HIC_ASM_14.503	PREDICTED: VPS10	VPS10 domain-contain	K12388	sortilin | (RefS
evm.model.HIC_ASM_10.431	PREDICTED: UBX dc	UBX domain-containir	--	

evm.model.HIC_ASM_8.1266	smoothelin-like protein	Smoothelin-like protein	K23028 cytospin | (Ref
evm.model.HIC_ASM_2.170	ubiquitin carboxyl-term	Ubiquitin carboxyl-term	K11869 ubiquitin carbo
evm.model.HIC_ASM_2.1855	methyltransferase-like	Methyltransferase-like	--
evm.model.HIC_ASM_21.379	gamma-glutamyltransfe	Glutathione hydrolase	K00681 gamma-glutam
evm.model.HIC_ASM_12.436	low quality protein: gre	GREB1-like protein OS	--
evm.model.HIC_ASM_2.1198	E3 ubiquitin-protein lig	E3 ubiquitin-protein lig	K22381 E3 ubiquitin-p
evm.model.HIC_ASM_14.808	LOW QUALITY PRO	WD repeat- and FYVE	K22262 WD repeat and
evm.model.HIC_ASM_1.817	leishmanolysin-like pep	Leishmanolysin-like pe	K13539 leishmanolysin
evm.model.HIC_ASM_10.420.2	PREDICTED: neurobla	Neuroblastoma suppres	K19558 neuroblastoma
evm.model.HIC_ASM_17.415	tenascin-N [Felis catus	Tenascin-N OS=Homo	K06252 tenascin | (Ref
evm.model.HIC_ASM_13.293	hypothetical protein PA	Dihydropyrimidinase-r	K07529 dihydropyrimi
evm.model.HIC_ASM_9.471	PREDICTED: probable	Probable low affinity c	K14687 solute carrier f
evm.model.HIC_ASM_0.640	PREDICTED: tryptoph	Tryptophan 2,3-dioxyg	K00453 tryptophan 2,3
evm.model.HIC_ASM_13.365	PREDICTED: calpain-	Calpain-14 OS=Homo	K13442 calpain-14 [EC
evm.model.HIC_ASM_1.133.3	vasoactive intestinal po	Vasoactive intestinal p	K04589 vasoactive inte
evm.model.HIC_ASM_0.858	PREDICTED: myozen	Myozenin-2 OS=Pongo	--
evm.model.HIC_ASM_9.586	tbc1 domain family me	TBC1 domain family n	K20165 TBC1 domain
evm.model.HIC_ASM_6.1260.2	PREDICTED: histidine	Histidine triad nucleoti	K15430 tRNA (guanin
evm.model.HIC_ASM_11.698	kielin/chordin-like prot	Kielin/chordin-like pro	K19398 Bardet-Biedl s
evm.model.HIC_ASM_21.945	Hypothetical predicted	Collagen alpha-1(XX)	K08133 collagen, type
evm.model.HIC_ASM_2.318	aspartyl aminopeptidas	Aspartyl aminopeptida	K01267 aspartyl amino
evm.model.HIC_ASM_6.1338	PREDICTED: brefeldi	Brefeldin A-inhibited g	K17572 brefeldin A-in
evm.model.HIC_ASM_7.444	OTU domain-containin	OTU domain-containin	K12655 OTU domain-c
evm.model.HIC_ASM_5.366	TRIO and F-actin-bind	TRIO and F-actin-bind	K20478 golgin subfam
evm.model.HIC_ASM_5.1337.1	PREDICTED: low-den	Low-density lipoprotei	K03068 low density lip
evm.model.HIC_ASM_10.262.5	exosome component 10	Exosome component 1	K12591 exosome comp
evm.model.HIC_ASM_16.345.2	PREDICTED: conserve	Conserved oligomeric	K20290 conserved olig
evm.model.HIC_ASM_5.857	zinc finger protein Eos	Zinc finger protein Eos	K09220 IKAROS fami
evm.model.HIC_ASM_3.874	dimethylglycine isoforn	Dimethylglycine dehyd	K00315 dimethylglycin
evm.model.HIC_ASM_19.979	PREDICTED: PHD fir	PHD finger protein 7 C	K22371 G2/M phase-sp
evm.model.HIC_ASM_11.928.1	PREDICTED: thiamin	Thiamin pyrophosphok	K00949 thiamine pyrop
evm.model.HIC_ASM_2.343	cilia- and flagella-asso	Cilia- and flagella-asso	K17570 hydrocephalus
evm.model.HIC_ASM_2.2019	PREDICTED: transme	Transmembrane protei	K20732 transmembran
evm.model.HIC_ASM_17.363	protein odr-4 homolog	Protein odr-4 homolog	--
evm.model.HIC_ASM_14.457	PREDICTED: transcrip	Transcription factor 7-	K04491 transcription f
evm.model.HIC_ASM_3.284.2	LOW QUALITY PRO	Ciliogenesis and plana	K22859 protein JBTS1
evm.model.HIC_ASM_14.468	beta-tectorin isoform X	Beta-tectorin OS=Mus	K18274 uromodulin | (
evm.model.HIC_ASM_15.283_ev	N-terminal EF-hand ca	N-terminal EF-hand ca	--
evm.model.HIC_ASM_12.207	LOW QUALITY PRO	Sia-alpha-2,3-Gal-beta-	K06613 alpha-N-acetyl
evm.model.HIC_ASM_3.825.3	PREDICTED: cyclin-H	Cyclin-H OS=Bos taur	K06634 cyclin H | (Ref
evm.model.HIC_ASM_14.60	AMSH-like protease [	AMSH-like protease O	K11867 AMSH-like pr
evm.model.HIC_ASM_9.71	collagen alpha-1(V) ch	Collagen alpha-1(V) ch	K19721 collagen, type
evm.model.HIC_ASM_8.983	neuron navigator 2 isof	Neuron navigator 2 OS	K19483 neuron naviga
evm.model.HIC_ASM_6.1374.1	syntaxin-11-like [Lynx	Syntaxin-11 OS=Homo	K08487 syntaxin 11 | (

evm.model.HIC_ASM_15.704	glycosyl-phosphatidyli	Glycosyl-phosphatidyli	--	
evm.model.HIC_ASM_1.128	PREDICTED: LOW Q	Serine/threonine-protei	K17545	serine/threonin
evm.model.HIC_ASM_5.1300	endoplasmic reticulum	Endoplasmic reticulum	K20355	endoplasmic re
evm.model.HIC_ASM_6.341	PREDICTED: corneod	Corneodesmosin OS=N	K23457	corneodesmos
evm.model.HIC_ASM_8.1348	vacuolar protein sortin	Vacuolar protein sortin	K12185	ESCRT-I com
evm.model.HIC_ASM_19.65.2	guanidinoacetate n-met	Guanidinoacetate N-me	K00542	guanidinoaceta
evm.model.HIC_ASM_4.1348	PREDICTED: GPI man	GPI mannosyltransfera	K05286	phosphatidylin
evm.model.HIC_ASM_1.579	PREDICTED: growth l	Growth hormone secre	K04284	growth hormo
evm.model.HIC_ASM_10.489	putative beta-lactamase	Putative beta-lactamase	K17382	serine beta-lac
evm.model.HIC_ASM_4.27	e3 ubiquitin-protein lig	E3 ubiquitin-protein lig	K10649	tripartite motif
evm.model.HIC_ASM_17.510	PREDICTED: cell surf	Cell surface A33 antige	K06790	cell surface A3
evm.model.HIC_ASM_4.1725	protein FAM189A1 [F	Protein FAM189A1 OS	K01201	glucosylceram
evm.model.HIC_ASM_13.1008.3	four and a half LIM do	Four and a half LIM do	K14380	four and a half
evm.model.HIC_ASM_11.1122	PREDICTED: WD rep	WD repeat-containing	K22869	WD repeat-con
evm.model.HIC_ASM_14.962	tetratricopeptide repeat	DnaJ homolog subfami	K09529	DnaJ homolog
evm.model.HIC_ASM_18.563	immunity-related GTPa	Immunity-related GTPa	--	
evm.model.HIC_ASM_21.585.1	PREDICTED: zinc fing	Zinc finger protein 335	K09228	KRAB domain
evm.model.HIC_ASM_19.669.2	PREDICTED: arrestin	Arrestin domain-contai	K20910	thioredoxin-in
evm.model.HIC_ASM_11.441	PREDICTED: rap guan	Rap guanine nucleotide	K08019	Rap guanine n
evm.model.HIC_ASM_4.305	PREDICTED: translati	Translation initiation fa	K03754	translation init
evm.model.HIC_ASM_20.1172.1	protein DBF4 homolog	Protein DBF4 homolog	K06629	activator of S p
evm.model.HIC_ASM_6.1093	PR domain zinc finger	PR domain zinc finger	K09228	KRAB domain
evm.model.HIC_ASM_2.1893.2	uroplakin-3b isoform X	Uroplakin-3b OS=Bos	K06058	deltex [EC:2.3
evm.model.HIC_ASM_8.1557	testis-specific protein 1	Testis-specific protein	K16772	protein FAM1
evm.model.HIC_ASM_5.256.1	lethal(3)malignant brai	Lethal(3)malignant bra	K10599	pre-mRNA-pro
evm.model.HIC_ASM_14.842	PREDICTED: LIM dom	LIM domain-binding p	K19867	LIM domain-b
evm.model.HIC_ASM_21.198	disintegrin and metallo	Disintegrin and metallo	K08616	disintegrin and
evm.model.HIC_ASM_4.123	PREDICTED: snRNA-	snRNA-activating prote	K15208	snRNA-activat
evm.model.HIC_ASM_13.134	grainyhead-like protein	Grainyhead-like protein	K09275	transcription fa
evm.model.HIC_ASM_17.136.2	PREDICTED: ladinin-	Ladinin-1 OS=Homo s	--	
evm.model.HIC_ASM_12.290	haloacid dehalogenase-	Immediate early respon	K22939	immediate ear
evm.model.HIC_ASM_18.608.1	2-oxoisovalerate dehyd	2-oxoisovalerate dehyd	K00166	2-oxoisovalera
evm.model.HIC_ASM_18.1189	epithelial splicing regu	Epithelial splicing regu	K14947	epithelial splic
evm.model.HIC_ASM_7.162	PREDICTED: ralBP1-a	RalBP1-associated Eps	K20068	RalBP1-associ
evm.model.HIC_ASM_0.99	PREDICTED: netrin re	Netrin receptor UNC5I	K07521	netrin receptor
evm.model.HIC_ASM_20.50	DNA ligase 3 isoform 1	DNA ligase 3 OS=Hon	K10776	DNA ligase 3
evm.model.HIC_ASM_8.1418	PREDICTED: TAF6-li	TAF6-like RNA polym	K03131	transcription in
evm.model.HIC_ASM_2.1672	PREDICTED: HIRA-in	HIRA-interacting prote	--	
evm.model.HIC_ASM_0.1265	acyl-synthetase family	Beta-alanine-activating	K00142	acyl-CoA synt
evm.model.HIC_ASM_19.538.6	PREDICTED: DDB1-	DDB1- and CUL4-asso	K11791	DDB1- and CU
evm.model.HIC_ASM_10.1573	DENN domain-contain	DENN domain-contain	K20161	DENN domain
evm.model.HIC_ASM_14.730	Tankyrase-2 [Tupaia c	Poly [ADP-ribose] poly	K10799	tankyrase [EC
evm.model.HIC_ASM_6.141	hypothetical protein DU	Stathmin domain-conta	--	
evm.model.HIC_ASM_1.726	PREDICTED: tumor p	Tumor protein 63 OS=1	K10149	tumor protein

evm.model.HIC_ASM_1.228	pp2c-like domain-conta	Protein phosphatase 2C	K17506	protein phosph
evm.model.HIC_ASM_1.1135	LOW QUALITY PRO	Cysteine and tyrosine-r	--	
evm.model.HIC_ASM_17.501	Myelin protein zero-lik	Myelin protein zero-lik	K18615	CapZ-interacti
evm.model.HIC_ASM_13.156	rho-associated protein	Rho-associated protein	K17388	Rho-associated
evm.model.HIC_ASM_10.1687	PREDICTED: sperm-a	Sperm-associated antig	--	
evm.model.HIC_ASM_10.5	PREDICTED: pleckstr	Pleckstrin homology do	K13710	Rho guanine n
evm.model.HIC_ASM_10.1341	calcium-activated chlo	Calcium-activated chlo	K05028	calcium-activa
evm.model.HIC_ASM_17.889.3	sugar transporter SWE	Sugar transporter SWE	K15382	solute carrier f
evm.model.HIC_ASM_16.289	protocadherin-17 isofo	Protocadherin-17 OS=H	K16499	protocadherin
evm.model.HIC_ASM_2.522.1	spats2-like protein isof	SPATS2-like protein C	K03211	ETS transloca
evm.model.HIC_ASM_10.525.2	interferon lambda recep	Interferon lambda recep	K05140	interferon lam
evm.model.HIC_ASM_11.737	podocalyxin [Acinony	Podocalyxin OS=Canis	K06817	podocalyxin-li
evm.model.HIC_ASM_6.860.1	ras-related protein Rab	Ras-related protein Rab	K06234	Ras-related pro
evm.model.HIC_ASM_10.1348	PREDICTED: 15 kDa	Selenoprotein F OS=H	K12662	U4/U6 small n
evm.model.HIC_ASM_12.971	V-set and immunoglob	V-set and immunoglob	K06081	poliovirus rece
evm.model.HIC_ASM_10.1610	hypothetical protein EI	Rho-related GTP-bindi	K07857	Ras homolog g
evm.model.HIC_ASM_18.692.2	NF-kappa-B inhibitor b	NF-kappa-B inhibitor b	K02581	NF-kappa-B in
evm.model.HIC_ASM_21.375	tumor protein p53-indu	Tumor protein p53-ind	K21247	tumor protein
evm.model.HIC_ASM_0.1211	odontogenic ameloblas	Odontogenic ameloblas	--	
evm.model.HIC_ASM_2.1090	peroxisomal 2,4-dienoy	Peroxisomal 2,4-dienoy	K13237	peroxisomal 2
evm.model.HIC_ASM_6.799	patched domain-contai	Patched domain-contai	K12385	Niemann-Pick
evm.model.HIC_ASM_4.1209	cytosolic phospholipas	Cytosolic phospholipas	K16342	cytosolic phos
evm.model.HIC_ASM_11.898	PREDICTED: chloride	Chloride channel prote	K05010	chloride chann
evm.model.HIC_ASM_6.709.3	serum response factor	Serum response factor	K04378	serum respons
evm.model.HIC_ASM_4.1097.1	unnamed protein produ	ER membrane protein c	K18203	[phosphatase 2
evm.model.HIC_ASM_8.209.2	porphobilinogen deami	Porphobilinogen deami	K01749	hydroxymethy
evm.model.HIC_ASM_4.1626.2	PREDICTED: secretor	Secretory carrier-assoc	K19995	secretory carri
evm.model.HIC_ASM_5.1264	spexin [Felis catus]	Spexin OS=Bos taurus	--	
evm.model.HIC_ASM_14.121.4	geranylgeranyl pyropho	Geranylgeranyl pyroph	K00804	geranylgerany
evm.model.HIC_ASM_4.369	PREDICTED: DNA ox	Nucleic acid dioxygena	K10765	alkylated DNA
evm.model.HIC_ASM_10.84	tetratricopeptide repeat	Tetratricopeptide repea	K19870	sperm-associat
evm.model.HIC_ASM_1.525	PREDICTED: otolin-1	Otolin-1 OS=Homo sap	K16629	collagen, type
evm.model.HIC_ASM_4.337	tubulinyl-Tyr carboxyp	Tubulinyl-Tyr carboxy	K23355	tubulinyl-Tyr c
evm.model.HIC_ASM_17.910.18	double-stranded RNA-s	Double-stranded RNA-	K12968	double-strande
evm.model.HIC_ASM_19.1294	PREDICTED: unchara	Uncharacterized protei	--	
evm.model.HIC_ASM_14.754	PREDICTED: LOW Q	Monocarboxylate trans	K11810	MFS transport
evm.model.HIC_ASM_4.1919	low quality protein: wa	WASP homolog-associ	K20479	WASP homolo
evm.model.HIC_ASM_2.944	coiled-coil domain-con	Coiled-coil domain-con	--	
evm.model.HIC_ASM_5.724	interferon-gamma [Ail	Interferon gamma OS=	K04687	interferon gam
evm.model.HIC_ASM_1.63	PREDICTED: DNA m	DNA mismatch repair p	K08734	DNA mismatc
evm.model.HIC_ASM_11.98	unnamed protein produ	Growth factor receptor	K20064	growth factor r
evm.model.HIC_ASM_2.394	SWI/SNF-related matri	SWI/SNF-related matri	K14440	SWI/SNF-rela
evm.model.HIC_ASM_12.1271.4	PREDICTED: tRNA ps	tRNA pseudouridine sy	K06173	tRNA pseudou
evm.model.HIC_ASM_4.836.2	protein transport protei	Protein transport protei	K14006	protein transpo

evm.model.HIC_ASM_4.1265	Hypothetical predicted	Telomere repeats-bindi	K12034	tripartite motif
evm.model.HIC_ASM_19.30.11	mediator of RNA polyı	Mediator of RNA poly	K15159	mediator of RI
evm.model.HIC_ASM_3.589	Kv channel-interacting	Kv channel-interacting	K16575	centractin | (R
evm.model.HIC_ASM_3.519	transcription factor SO	Transcription factor SC	K09271	transcription fa
evm.model.HIC_ASM_5.284	PREDICTED: voltage-	Voltage-dependent T-t	K04856	voltage-depen
evm.model.HIC_ASM_20.152	flotillin-2 isoform X1	Flotillin-2 OS=Rattus ı	K07192	flotillin | (RefS
evm.model.HIC_ASM_5.1456	condensin complex sub	Condensin complex sul	K06677	condensin com
evm.model.HIC_ASM_2.697	PREDICTED: rap guar	Rap guanine nucleotide	K04351	Rap guanine n
evm.model.HIC_ASM_8.1212.9	MAP kinase-activating	MAP kinase-activating	K18061	myotubularin-
evm.model.HIC_ASM_18.1372	PREDICTED: TLD do	MTOR-associated prot	--	
evm.model.HIC_ASM_8.269	low quality protein: cx	CXADR-like membran	K06789	adipocyte-spec
evm.model.HIC_ASM_5.664.2	PREDICTED: cysteine	Cysteine and glycine-ri	K09377	cysteine and g
evm.model.HIC_ASM_3.932	pentatricopeptide repea	Pentatricopeptide repea	K17711	pentatricopept
evm.model.HIC_ASM_1.989	HMG box transcriptior	HMG box transcriptior	K21643	HMG box tran
evm.model.HIC_ASM_4.226	SPARC-related modula	SPARC-related modula	K10809	thyroglobulin
evm.model.HIC_ASM_12.690	seizure 6-like protein [	Seizure 6-like protein (	K10362	myosin XVIII
evm.model.HIC_ASM_10.513.1	hydroxymethylglutaryl	Hydroxymethylglutary	K01640	hydroxymethy
evm.model.HIC_ASM_7.505	pre-rRNA-processing p	Pre-rRNA-processing p	K14800	pre-rRNA-pro
evm.model.HIC_ASM_13.845.1	protein phosphatase 1 ı	Protein phosphatase 1 ı	K17562	protein phosph
evm.model.HIC_ASM_3.1290	PREDICTED: UPF025	Major intrinsically disc	K08631	a disintegrin a
evm.model.HIC_ASM_4.1083	telomerase protein com	Telomerase protein cor	K11127	telomerase pro
evm.model.HIC_ASM_19.891_ev	PREDICTED: E3 ubiq	E3 ubiquitin-protein li	K12169	Kip1 ubiquitin
evm.model.HIC_ASM_14.701	PREDICTED: nucleola	Nucleolar complex pro	K14834	nucleolar com
evm.model.HIC_ASM_18.648.1	RAC-beta serine/threoı	RAC-beta serine/threoı	K04456	RAC serine/th
evm.model.HIC_ASM_11.675.1	EBNA-2 co-activator v	Staphylococcal nucleas	K15979	staphylococca
evm.model.HIC_ASM_19.946.2	DDB1- and CUL4-asso	DDB1- and CUL4-asso	K11789	HIV-1 Vpr-bir
evm.model.HIC_ASM_17.631	coiled-coil domain-con	Coiled-coil domain-cor	--	
evm.model.HIC_ASM_10.874	PREDICTED: microtul	Microtubule-actin cros	K19827	microtuble-act
evm.model.HIC_ASM_13.502	zinc finger ccch domai	Zinc finger CCCH dom	--	
evm.model.HIC_ASM_3.650.2	sideroflexin-1 isoform	Sideroflexin-1 OS=Hoı	K03351	anaphase-prom
evm.model.HIC_ASM_10.1525	PREDICTED: myosin-	Myosin-binding protei	K21443	proline/serine-
evm.model.HIC_ASM_17.932	PREDICTED: nuclear	Nuclear pore membran	K14314	nuclear pore c
evm.model.HIC_ASM_19.608	Hypothetical predicted	Uncharacterized protei	--	
evm.model.HIC_ASM_12.886	hypothetical protein EI	Unconventional myosi	K10356	myosin I | (Re
evm.model.HIC_ASM_13.575.2	ELMO domain-contain	ELMO domain-contain	K04139	adrenergic rec
evm.model.HIC_ASM_20.1499	alpha-sarcoglycan [Ac	Alpha-sarcoglycan OS	K12565	alpha-sarcogly
evm.model.HIC_ASM_18.1300	synaptic vesicle memb	Synaptic vesicle memb	K23167	synaptic vesic
evm.model.HIC_ASM_12.259_ev	hypothetical protein Ca	Methyl-CpG-binding d	K11589	methyl-CpG-b
evm.model.HIC_ASM_3.541	cyclin-J-like protein iso	Cyclin-J-like protein O	K06627	cyclin-A | (Re
evm.model.HIC_ASM_11.30	unconventional myosin	Unconventional myosi	K10356	myosin I | (Re
evm.model.HIC_ASM_9.1037	excitatory amino acid t	Excitatory amino acid	K05612	solute carrier f
evm.model.HIC_ASM_21.901.2	PREDICTED: proteaso	Proteasomal ubiquitin ı	K06691	26S proteasom
evm.model.HIC_ASM_13.551	PREDICTED: DNA-di	DNA-directed RNA po	K02999	DNA-directed
evm.model.HIC_ASM_20.112	LYR motif-containing	LYR motif-containing	--	

evm.model.HIC_ASM_5.434.2	unnamed protein produ	Target of Myb protein	K16677 dachs | (RefSe
evm.model.HIC_ASM_9.999	PREDICTED: phospha	Phosphatidylinositol 4-	K00889 1-phosphatidy
evm.model.HIC_ASM_3.708	hypothetical protein PA	Signal recognition part	K03105 signal recogni
evm.model.HIC_ASM_0.1260	PREDICTED: testicula	Testicular haploid expr	K07957 ADP-ribosylat
evm.model.HIC_ASM_3.536.1	tetratricopeptide repeat	Tetratricopeptide repea	K09228 KRAB domain
evm.model.HIC_ASM_18.689.3	serine--tRNA ligase, m	Serine--tRNA ligase, m	K01875 seryl-tRNA sy
evm.model.HIC_ASM_3.238	UPF0489 protein C5or	UPF0489 protein C5or	--
evm.model.HIC_ASM_2.367	ciliogenesis-associated	Ciliogenesis-associated	K12347 natural resistar
evm.model.HIC_ASM_3.185	PREDICTED: dynein h	Dynein heavy chain 5,	K10408 dynein heavy c
evm.model.HIC_ASM_10.1113	doublesex- and mab-3-	Doublesex- and mab-3-	K19492 doublesex- and
evm.model.HIC_ASM_1.559	PREDICTED: sterile a	Sterile alpha motif dom	K11456 polyhomeotic-
evm.model.HIC_ASM_4.476	PREDICTED: protein u	Protein unc-79 homolo	--
evm.model.HIC_ASM_14.652.7	MORN repeat-containi	MORN repeat-containi	K19755 radial spoke h
evm.model.HIC_ASM_3.682.4	PREDICTED: ras-relat	Ras-related protein Ral	K07912 Ras-related pr
evm.model.HIC_ASM_2.565.4	signal transducer and a	Signal transducer and a	K11220 signal transduc
evm.model.HIC_ASM_13.720.1	NFU1 iron-sulfur clust	NFU1 iron-sulfur clust	K22074 NFU1 iron-sul
evm.model.HIC_ASM_18.846.5	rhophilin-2 [Puma con	Rhophilin-2 OS=Bos ta	K18040 tyrosine-protei
evm.model.HIC_ASM_18.875	PREDICTED: zinc fing	Zinc finger protein 536	K22411 zinc finger pro
evm.model.HIC_ASM_0.1545	hypothetical protein H0	Janus kinase and micro	K20478 golgin subfam
evm.model.HIC_ASM_20.1432	src kinase-associated p	Src kinase-associated p	K17699 src kinase asso
evm.model.HIC_ASM_20.234.1	telomerase-binding pro	Telomerase-binding pro	K11124 protein SMG6
evm.model.HIC_ASM_5.36	PREDICTED: RNA he	RNA helicase Mov10l1	K13983 putative helica
evm.model.HIC_ASM_6.1088	PREDICTED: protein l	Protein lin-28 homolog	K18754 protein lin-28
evm.model.HIC_ASM_18.1333	PREDICTED: 1-phosp	1-phosphatidylinositol	K05859 phosphatidylin
evm.model.HIC_ASM_10.527	PREDICTED: grainyhe	Grainyhead-like protein	K09275 transcription f
evm.model.HIC_ASM_12.1112	cilia- and flagella-asso	Cilia- and flagella-asso	K10409 dynein interme
evm.model.HIC_ASM_18.904	neuropilin and tolloid-l	Neuropilin and tolloid-	K14616 cubilin | (RefS
evm.model.HIC_ASM_9.908	PREDICTED: BDNF/N	BDNF/NT-3 growth fa	K04360 neurotrophic t
evm.model.HIC_ASM_10.1753	PREDICTED: sorting n	Sorting nexin-27 OS=M	K17936 sorting nexin-2
evm.model.HIC_ASM_18.1283	PREDICTED: tRNA-sp	tRNA-specific adenosi	K15440 tRNA-specific
evm.model.HIC_ASM_4.1647	chondroitin sulfate pro	Chondroitin sulfate pro	K08115 chondroitin su
evm.model.HIC_ASM_10.12	uncharacterized protein	Uncharacterized protei	--
evm.model.HIC_ASM_4.1764	PREDICTED: protein I	Protein FAM169B OS=	K12869 crooked neck |
evm.model.HIC_ASM_9.446	AT-hook-containing tra	Microtubule organizati	K21404 AT-hook-cont
evm.model.HIC_ASM_20.829	PREDICTED: dynein h	Dynein heavy chain 17	K10408 dynein heavy c
evm.model.HIC_ASM_17.498	PREDICTED: mitocho	Mitochondrial pyruvate	K22139 mitochondrial
evm.model.HIC_ASM_20.382	PREDICTED: zinc fing	Zinc finger SWIM dom	--
evm.model.HIC_ASM_20.1394	PREDICTED: SRC kin	SRC kinase signaling i	K19930 SRC kinase sig
evm.model.HIC_ASM_0.1401	NACHT and WD repea	NACHT and WD repea	K23213 paired amphip
evm.model.HIC_ASM_18.1239.1	hydrocephalus-inducin	Hydrocephalus-inducin	K17570 hydrocephalus
evm.model.HIC_ASM_2.962.1	secretin receptor isofor	Secretin receptor OS=H	K04588 secretin recept
evm.model.HIC_ASM_14.182	PWWP domain-contain	PWWP domain-contain	K17398 DNA (cytosine
evm.model.HIC_ASM_0.796	PREDICTED: UPF046	UPF0462 protein C4or	--
evm.model.HIC_ASM_11.947.2	PREDICTED: cullin-1	Cullin-1 OS=Mus muscu	K03347 cullin 1 | (RefS

evm.model.HIC_ASM_12.1134	huntingtin-interacting p	Huntingtin-interacting	K20040 huntingtin-inte
evm.model.HIC_ASM_5.1653	enkurin [Ursus arctos	Enkurin OS=Homo sap	--
evm.model.HIC_ASM_1.1265.1	PREDICTED: protein	Protein C-ets-2 OS=Ho	K21932 C-ets-2 | (RefS
evm.model.HIC_ASM_10.718	peflin isoform X2 [Su	Peflin OS=Homo sapie	K08573 calpain-3 [EC:
evm.model.HIC_ASM_9.479	thymic stromal cotrans	Thymic stromal cotrans	K14614 MFS transport
evm.model.HIC_ASM_12.1019	PREDICTED: T-box tr	T-box transcription fac	K10179 T-box protein
evm.model.CTG_1467.28.1	PREDICTED: rabanky	Rabankyrin-5 OS=Mus	K20129 rabankyrin-5 |
evm.model.HIC_ASM_1.150	PREDICTED: coiled-c	Coiled-coil domain-cor	--
evm.model.HIC_ASM_2.453	low quality protein: [L	Dystrotelin OS=Homo	K10366 dystrophin | (R
evm.model.HIC_ASM_0.653	PREDICTED: fibrinog	Fibrinogen gamma cha	K03903 fibrinogen alp
evm.model.HIC_ASM_3.479	PREDICTED: glycine	Glycine receptor subun	K05193 glycine recept
evm.model.HIC_ASM_7.215.2	PREDICTED: peroxire	Peroxiredoxin-4 OS=M	K03386 peroxiredoxin
evm.model.HIC_ASM_3.815	metallo-beta-lactamase	Metallo-beta-lactamase	K16466 centrin-3 | (Re
evm.model.HIC_ASM_20.947	cerebellar degeneration	Cerebellar degeneratio	--
evm.model.HIC_ASM_20.1237	serine/threonine-protei	Serine/threonine-protei	K08867 WNK lysine d
evm.model.HIC_ASM_11.1059	dipeptidyl aminopeptid	Dipeptidyl aminopeptic	K23013 dipeptidyl ami
evm.model.HIC_ASM_13.781	PREDICTED: protein	Protein FAM161A OS	K16772 protein FAM1
evm.model.HIC_ASM_9.698	spermatid-specific man	Spermatid-specific mai	K18634 spermatid-spe
evm.model.HIC_ASM_13.576	all-trans-retinol 13,14-	All-trans-retinol 13,14-	K09516 all-trans-retinc
evm.model.HIC_ASM_18.445	PREDICTED: probabl	Probable ATP-depende	K20101 ATP-depender
evm.model.HIC_ASM_9.481	PREDICTED: sorting	Sorting nexin-30 OS=H	K17921 sorting nexin-
evm.model.HIC_ASM_1.322	PREDICTED: cohesin	Cohesin subunit SA-1	K06671 cohesin compl
evm.model.HIC_ASM_12.95	uncharacterized proteir	Uncharacterized protei	--
evm.model.HIC_ASM_0.620	peptidyl-prolyl cis-tran	Peptidyl-prolyl cis-tran	K05864 peptidyl-proly
evm.model.HIC_ASM_1.1307	PREDICTED: C2 dom	C2 domain-containing	--
evm.model.HIC_ASM_8.146	PREDICTED: RING fi	RING finger protein 21	K19475 WAS/WASL-
evm.model.HIC_ASM_14.118.1	UDP-GalNAc:beta-1,3	UDP-GalNAc:beta-1,3	K09654 beta-1,3-N-ace
evm.model.HIC_ASM_11.643	fez family zinc finger p	Fez family zinc finger	K09223 growth factor
evm.model.HIC_ASM_5.752	uncharacterized proteir	Uncharacterized protei	--
evm.model.HIC_ASM_6.813	transcription factor AP	Transcription factor AI	K09178 transcription f
evm.model.HIC_ASM_8.152	Down syndrome cell ad	Down syndrome cell ad	K06768 Down syndron
evm.model.HIC_ASM_1.1404	PREDICTED: collager	Collagen alpha-2(VI) c	K06238 collagen, type
evm.model.HIC_ASM_6.692	PREDICTED: GLTSC	BRD4-interacting chro	K14971 nuclear recept
evm.model.HIC_ASM_0.792	PREDICTED: protocad	Protocadherin-10 OS=	K16499 protocadherin
evm.model.HIC_ASM_4.615	tryptophan--tRNA ligas	Tryptophan--tRNA liga	K01867 tryptophanyl-t
evm.model.HIC_ASM_4.1356	E3 ubiquitin-protein lig	E3 ubiquitin-protein lig	K10591 E3 ubiquitin-p
evm.model.HIC_ASM_11.294	cilia- and flagella-asso	Cilia- and flagella-asso	--
evm.model.HIC_ASM_10.60	cilia- and flagella-asso	Cilia- and flagella-asso	--
evm.model.HIC_ASM_6.1027	PREDICTED: LOW Q	Gap junction alpha-10	K07631 gap junction a
evm.model.HIC_ASM_6.1415	katanin p60 atpase-con	Katanin p60 ATPase-c	K07767 katanin p60 A
evm.model.HIC_ASM_3.710	adenomatous polyposis	Adenomatous polyposi	K02085 adenomatosis
evm.model.HIC_ASM_10.722	PREDICTED: SPOC d	SPOC domain-containi	K17596 SPOC domain
evm.model.HIC_ASM_12.697	PREDICTED: protein-	Protein-tyrosine sulfotr	K01021 protein-tyrosir
evm.model.HIC_ASM_14.1084	Hypothetical predicted	Probable JmjC domain	K11449 jumonji domai

evm.model.HIC_ASM_7.459.1	coiled-coil domain-con	Coiled-coil domain-cor	K23343	coiled-coil dor
evm.model.HIC_ASM_2.1698	leukosialin [Felis catus	Leukosialin OS=Homo	K06477	sialophorin | (l
evm.model.HIC_ASM_11.402	PREDICTED: leucine-	Leucine-rich repeat-co	K11092	U2 small nucle
evm.model.HIC_ASM_19.907	macrophage-stimulatin	Macrophage-stimulatin	K05100	macrophage-st
evm.model.HIC_ASM_21.525	PREDICTED: peptidas	Peptidase inhibitor R3I	K19919	cysteine-rich s
evm.model.HIC_ASM_6.1435	zinc finger and BTB dc	Zinc finger and BTB d	K10489	zinc finger and
evm.model.HIC_ASM_12.292.4	PREDICTED: E3 SUM	E3 SUMO-protein liga	K16063	E3 SUMO-pro
evm.model.HIC_ASM_14.951	zinc finger SWIM dom	Zinc finger SWIM dom	K01231	alpha-mannosi
evm.model.CTG_335.10	PREDICTED: tetraspa	Tetraspanin-32 OS=Ho	K17359	tetraspanin-32
evm.model.HIC_ASM_6.854	collagen alpha-1(XXI)	Collagen alpha-1(XXI)	K16629	collagen, type
evm.model.HIC_ASM_10.1551	sodium-dependent neu	Sodium-dependent neu	K05048	solute carrier f
evm.model.HIC_ASM_13.539	LOW QUALITY PRO	Histone-lysine N-meth	K11426	SET and MYN
evm.model.HIC_ASM_4.1255	FERM domain-contain	FERM domain-contain	K18037	tyrosine-protei
evm.model.HIC_ASM_18.1235	transducin-like enhanc	Transducin-like enhanc	K04497	groucho | (Ref
evm.model.HIC_ASM_4.1870	PREDICTED: GDP-D-	GDP-D-glucose phospl	K15630	GDP-D-glucos
evm.model.HIC_ASM_17.1086	exonuclease 1 [Lynx p	Exonuclease 1 OS=Ho	K10746	exonuclease 1
evm.model.HIC_ASM_21.772	PREDICTED: meiotic	Meiotic recombination	K10878	meiotic recom
evm.model.HIC_ASM_12.1088	PREDICTED: tectonic	Tectonic-1 OS=Homo	K19382	tectonic-1/3 | (
evm.model.HIC_ASM_5.125.2	G2 and S phase-expres	G2 and S phase-expres	K10129	G-2 and S-pha
evm.model.HIC_ASM_5.646	phosphatidylinositol pl	Phosphatidylinositol pl	K16910	receptor-type t
evm.model.HIC_ASM_15.623	potassium channel sub	Potassium channel sub	K04919	potassium cha
evm.model.HIC_ASM_9.350	serine/threonine-protei	Serine/threonine-protei	K20875	NIMA (never
evm.model.HIC_ASM_6.392.1	apolipoprotein M isofo	Apolipoprotein M OS=	--	
evm.model.HIC_ASM_18.1245	L-fucose kinase isoforn	L-fucose kinase OS=H	K05305	fucokinase [EC
evm.model.HIC_ASM_11.577	anillin isoform X3 [Fe	Anillin OS=Homo sapi	K18621	actin-binding p
evm.model.HIC_ASM_10.1239.2	PREDICTED: growth a	Growth arrest and DNA	K04402	growth arrest a
evm.model.HIC_ASM_17.509.1	inactive dual specificit	Inactive dual specificit	K14165	atypical dual s
evm.model.HIC_ASM_5.280	PREDICTED: ENTH c	ENTH domain-contain	--	
evm.model.HIC_ASM_4.1765	PREDICTED: arrestin	Arrestin domain-contai	K20910	thioredoxin-in
evm.model.HIC_ASM_18.1120	heat shock factor prote	Heat shock factor prote	K09417	heat shock tran
evm.model.HIC_ASM_3.1020	PREDICTED: mitogen	Mitogen-activated prot	K04416	mitogen-activa
evm.model.HIC_ASM_8.1226	PREDICTED: protein	NADH dehydrogenase	K03936	NADH dehydr
evm.model.HIC_ASM_10.1829.2	PREDICTED: hemoju	Hemojuvelin OS=Mus	K23100	hemojuvelin |
evm.model.HIC_ASM_8.1666	two pore calcium chan	Two pore calcium char	K14077	two pore calci
evm.model.HIC_ASM_1.257	PREDICTED: methylt	Methyltransferase-like	K00599	methyltransfer
evm.model.HIC_ASM_19.888	protein bassoon [Lynx	Protein bassoon OS=H	K01303	acylaminoacyl
evm.model.HIC_ASM_4.64	disks large-associated	Disks large-associated	K16804	disks large-ass
evm.model.HIC_ASM_8.18.1	protein NPAT isoform	Protein NPAT OS=Ho	--	
evm.model.HIC_ASM_9.202.6	ubiquitin carboxyl-tern	Ubiquitin carboxyl-terr	K11848	ubiquitin carbo
evm.model.HIC_ASM_12.871.1	ARF GTPase-activatin	ARF GTPase-activatin	K12487	G protein-coup
evm.model.HIC_ASM_0.1183	PREDICTED: G-rich s	G-rich sequence factor	K12898	heterogeneous
evm.model.HIC_ASM_1.448	mediator of RNA poly	Mediator of RNA poly	K15162	mediator of RI
evm.model.HIC_ASM_19.663.4	tyrosine-protein kinase	Tyrosine-protein kinas	K11218	Janus kinase 3
evm.model.HIC_ASM_15.31	suppression of tumorig	Suppression of tumorig	K23192	myelin transcr

evm.model.HIC_ASM_19.856	cadherin EGF LAG sev	Cadherin EGF LAG se	K14704	solute carrier f
evm.model.HIC_ASM_18.1236	calretinin-like, partial	Calretinin OS=Mus mu	K14455	aspartate amin
evm.model.HIC_ASM_11.140	inhibin beta A chain pr	Inhibin beta A chain O	K04667	inhibin beta A
evm.model.HIC_ASM_2.376	RUN and FYVE doma	RUN and FYVE domai	K21954	FYVE and coi
evm.model.HIC_ASM_6.660	myoD family inhibitor	MyoD family inhibitor	--	
evm.model.HIC_ASM_1.1128	39S ribosomal protein	39S ribosomal protein	K17420	large subunit r
evm.model.HIC_ASM_21.510	lethal(3)malignant brai	Lethal(3)malignant bra	K11461	polycomb prot
evm.model.HIC_ASM_10.1234	PREDICTED: unchara	Uncharacterized protei	--	
evm.model.HIC_ASM_5.1903	PREDICTED: disco-in	Disco-interacting prote	K05637	laminin, alpha
evm.model.HIC_ASM_10.25	amiloride-sensitive sod	Amiloride-sensitive so	K04826	amiloride-sens
evm.model.HIC_ASM_3.771	ubiquitin-like protein 5	Ubiquitin-like protein 5	K13113	ubiquitin-like
evm.model.HIC_ASM_1.1383.2	PREDICTED: pituitary	Pituitary tumor-transfo	K12160	small ubiquitin
evm.model.HIC_ASM_4.307	PREDICTED: DNA m	DNA mismatch repair	K08739	DNA mismatc
evm.model.HIC_ASM_4.1206	spectrin beta chain, no	Spectrin beta chain, no	K06115	spectrin beta |
evm.model.HIC_ASM_8.46	colorectal cancer-assoc	Colorectal cancer-asso	--	
evm.model.HIC_ASM_2.1794	PREDICTED: S-adeno	S-adenosyl-L-methioni	K15449	tRNA wybutos
evm.model.HIC_ASM_12.730	X-box-binding protein	X-box-binding protein	K09027	X box-binding
evm.model.HIC_ASM_3.1032	PREDICTED: multicil	Multicilin OS=Homo s	K10749	geminin | (Ref
evm.model.HIC_ASM_1.1281	PREDICTED: immuno	Immunoglobulin super	K06786	junctional adh
evm.model.HIC_ASM_14.762.1	ras-GEF domain-contai	Ras-GEF domain-conta	K03781	catalase [EC:1
evm.model.HIC_ASM_17.108	lysine-specific demeth	Lysine-specific demeth	K11446	histone demet
evm.model.HIC_ASM_2.1045	LIM and senescent cell	LIM and senescent cell	K23354	LIM and senes
evm.model.HIC_ASM_10.1778	PREDICTED: protein	Protein MENT OS=Ho	--	
evm.model.HIC_ASM_1.1144	PREDICTED: hemK m	Methyltransferase N6A	K19589	release factor
evm.model.HIC_ASM_2.261	PREDICTED: collagen	Collagen alpha-3(IV) c	K06237	collagen, type
evm.model.HIC_ASM_20.1290	PREDICTED: synapto	Endoplasmic reticulum	K22461	endoplasmic re
evm.model.HIC_ASM_13.565.3	PREDICTED: E3 ubiq	E3 ubiquitin-protein lig	K22378	E3 ubiquitin-p
evm.model.HIC_ASM_5.1687	LOW QUALITY PRO	SKI/DACH domain-co	--	
evm.model.HIC_ASM_4.1917	fibronectin type III and	Fibronectin type III and	K10647	midline 2 [EC
evm.model.HIC_ASM_8.292	roundabout homolog 3	Roundabout homolog 3	K06755	roundabout, ax
evm.model.HIC_ASM_12.321	unnamed protein produ	Synaptotagmin-4 OS=H	K19904	synaptotagmin
evm.model.HIC_ASM_4.1634.1	PREDICTED: COMM	COMM domain-contai	K22560	COMM domai
evm.model.HIC_ASM_0.739	GRB2-associated-bindi	GRB2-associated-bindi	K09593	GRB2-associa
evm.model.HIC_ASM_1.205	PREDICTED: thyroid	Thyroid hormone recep	K08362	thyroid hormo
evm.model.HIC_ASM_1.454	succinate receptor 1 [F	Succinate receptor 1 O	K10042	succinate rece
evm.model.HIC_ASM_6.835	PREDICTED: elongati	Elongation of very long	K10244	elongation of v
evm.model.HIC_ASM_9.192.1	far upstream element-b	Far upstream element-b	K13210	far upstream e
evm.model.HIC_ASM_7.1059	PREDICTED: cancer/t	RNA helicase Mov10l1	K13983	putative helica
evm.model.HIC_ASM_12.883	PREDICTED: ubiquiti	Ubiquitin-protein ligas	K10588	ubiquitin-prote
evm.model.HIC_ASM_10.1220	PREDICTED: tyrosine	Tyrosine-protein kinas	K11217	Janus kinase 1
evm.model.HIC_ASM_10.946	tyrosine-protein kinase	Tyrosine-protein kinas	K05120	tyrosine kinas
evm.model.HIC_ASM_20.268	PREDICTED: transien	Transient receptor pote	K05222	transient recep
evm.model.HIC_ASM_9.934.1	transducin-like enhanc	Transducin-like enhanc	K04497	groucho | (Ref
evm.model.HIC_ASM_9.579	polypeptide N-acetylga	Polypeptide N-acetylga	K00710	polypeptide N

evm.model.HIC_ASM_4.1833.2	hyaluronan and proteo	Hyaluronan and proteo	K06852	hyaluronan an
evm.model.HIC_ASM_3.1251	follistatin-related prote	Follistatin-related prot	K06766	neogenin | (Re
evm.model.HIC_ASM_4.1891.2	ras GTPase-activating-	Ras GTPase-activating	K16848	Ras GTPase-a
evm.model.HIC_ASM_13.625.1	PREDICTED: serine p	Serine protease HTRA	K08669	HtrA serine pe
evm.model.HIC_ASM_9.130	general transcription fa	General transcription f	K15202	general transc
evm.model.HIC_ASM_2.1496	5-demethoxyubiquinon	5-demethoxyubiquinon	K06134	3-demethoxyu
evm.model.HIC_ASM_5.1647	myosin-IIIa isoform X	Myosin-IIIa OS=Homo	K08834	myosin III [EC
evm.model.HIC_ASM_6.1413.1	hypothetical protein C	Peptidyl-prolyl cis-tran	K12735	peptidyl-proly
evm.model.HIC_ASM_20.85	PREDICTED: rhombo	Rhomboid-related prot	K02857	rhomboid-rela
evm.model.HIC_ASM_21.83	PREDICTED: sodium/	Sodium/potassium/calc	K13751	solute carrier f
evm.model.HIC_ASM_6.217	F-actin-uncapping prot	F-actin-uncapping prot	K20493	leucine-rich re
evm.model.HIC_ASM_6.105.2	phosphatase and actin	Phosphatase and actin	K17594	phosphatase an
evm.model.HIC_ASM_19.1128	PREDICTED: forkhea	Forkhead box protein	K09409	forkhead box p
evm.model.HIC_ASM_14.940.1	histone acetyltransfera	Histone acetyltransfera	K11306	histone acetylt
evm.model.HIC_ASM_16.6.1	PREDICTED: ras GTP	Ras GTPase-activating	K12380	Ras GTPase-a
evm.model.HIC_ASM_6.1506	poly(A)-specific ribon	Poly(A)-specific ribon	K01148	poly(A)-specif
evm.model.HIC_ASM_8.966	tyrosine-protein phosp	Tyrosine-protein phosp	K18018	tyrosine-protei
evm.model.HIC_ASM_1.1133	amyloid beta A4 protei	Amyloid-beta A4 prote	K04520	amyloid beta A
evm.model.HIC_ASM_18.617.1	heterogeneous nuclear	Heterogeneous nuclear	K15047	heterogeneous
evm.model.HIC_ASM_12.7.1	rna polymerase ii subu	RNA polymerase II sul	K15732	RNA polymer
evm.model.HIC_ASM_5.235	meiosis inhibitor protei	Meiosis inhibitor prote	--	
evm.model.HIC_ASM_2.1076	PREDICTED: regulato	Regulator of G-protein	K16449	regulator of G
evm.model.HIC_ASM_20.1284	ATP-citrate synthase is	ATP-citrate synthase C	K01648	ATP citrate (p
evm.model.HIC_ASM_16.250	hypothetical protein C	Mitotic-spindle organiz	K18633	mitotic-spindl
evm.model.HIC_ASM_12.794	POZ-, AT hook-, and z	POZ-, AT hook-, and z	K10518	zinc finger and
evm.model.HIC_ASM_12.504	PREDICTED: ankyrin	Ankyrin repeat domain	K21436	ankyrin repeat
evm.model.HIC_ASM_12.989	UPF0454 protein C12c	UPF0454 protein C12c	--	
evm.model.HIC_ASM_10.967	hypothetical protein C	Beta-1,4-galactosyltran	K07967	beta-1,4-galac
evm.model.HIC_ASM_1.1017	PREDICTED: adhesio	Adhesion G-protein co	K08464	adhesion G-pr
evm.model.HIC_ASM_15.133	hydroxyacid-oxoacid t	Hydroxyacid-oxoacid t	K11173	hydroxyacid-o
evm.model.HIC_ASM_2.1540	ankyrin repeat and sam	Ankyrin repeat and SA	K21414	ankyrin repeat
evm.model.HIC_ASM_17.390	c-type lectin [Lynx pa	Putative C-type lectin	K06560	mannose recep
evm.model.HIC_ASM_17.561	PREDICTED: RRP15-	RRP15-like protein OS	--	
evm.model.HIC_ASM_14.399	PREDICTED: protein	Protein FAM204A OS	K10583	ubiquitin-conj
evm.model.HIC_ASM_12.535	proteasome assembly c	Proteasome assembly c	K11876	proteasome as
evm.model.HIC_ASM_5.350	brain-specific angioger	Brain-specific angioge	K20127	BAI1-associat
evm.model.HIC_ASM_10.1425.1	tissue factor [Acinony	Tissue factor OS=Bos	K03901	coagulation fa
evm.model.HIC_ASM_12.453	protein LLP homolog	Uncharacterized protei	K02910	large subunit r
evm.model.HIC_ASM_1.382	PREDICTED: transcri	Transcription factor D	K09392	transcription f
evm.model.HIC_ASM_6.1123	Nuclear receptor subfa	Nuclear receptor subfa	K08545	nuclear recept
evm.model.HIC_ASM_20.1366.3	PREDICTED: F-box/L	F-box/LRR-repeat prot	K10268	F-box and leuc
evm.model.HIC_ASM_7.915	unnamed protein produ	Solute carrier family 2	K15120	solute carrier f
evm.model.HIC_ASM_5.1764	CAMK1D isoform 2, p	Calcium/calmodulin-de	K08794	calcium/calmo
evm.model.HIC_ASM_12.748	PREDICTED: calcium	Calcium-binding protei	K02183	calmodulin | (I

evm.model.HIC_ASM_10.1629	homeodomain-interacti	Homeodomain-interact	K08826	homeodomain
evm.model.HIC_ASM_9.105.1	ADAMTS-like protein	ADAMTS-like protein	K23377	thrombospond
evm.model.HIC_ASM_18.1263	Golgi apparatus protein	Golgi apparatus protein	K06816	golgi apparatu
evm.model.HIC_ASM_9.815	PREDICTED: FRAS1-	FRAS1-related extrace	K23380	FRAS1-related
evm.model.HIC_ASM_12.113	docking protein 6 [Ho	Docking protein 6 OS=	K14752	docking protei
evm.model.HIC_ASM_16.357	leucine-rich repeat-con	Leucine-rich repeat-co	K10130	leucine-rich re
evm.model.HIC_ASM_7.1307	methyl--binding protein	Methyl-CpG-binding p	K11588	methyl CpG bi
evm.model.HIC_ASM_0.407	zinc transporter ZIP14	Zinc transporter ZIP14	K14720	solute carrier f
evm.model.HIC_ASM_5.698.2	PREDICTED: calcium	Calcium-activated pota	K04941	potassium larg
evm.model.HIC_ASM_5.186	EF-hand calcium-bindi	EF-hand calcium-bindi	K02183	calmodulin | (I
evm.model.HIC_ASM_10.577.3	E3 ubiquitin-protein lig	E3 ubiquitin-protein lig	K10655	tripartite motif
evm.model.HIC_ASM_19.353	protein PET100 homol	Protein PET100 homol	K18186	protein PET10
evm.model.HIC_ASM_7.139	PREDICTED: cytoplas	Cytoplasmic tyrosine-p	K08896	cytoplasmic ty
evm.model.HIC_ASM_18.99	Hypothetical predicted	Protein FAM71E2 OS=	K04160	5-hydroxytryp
evm.model.HIC_ASM_4.1361	meiosis-specific nucle	Meiosis-specific nucle	--	
evm.model.HIC_ASM_2.800	secretory phospholipas	Secretory phospholipas	K06560	mannose recep
evm.model.HIC_ASM_8.938.1	harmonin isoform X1	Harmonin OS=Bos tau	K21877	harmonin | (Re
evm.model.HIC_ASM_11.523	pleckstrin homology do	Pleckstrin homology do	K08051	pleckstrin hom
evm.model.HIC_ASM_15.44	regulator of G-protein s	Regulator of G-protein	K16449	regulator of G
evm.model.HIC_ASM_19.1271	PREDICTED: intraflag	Intraflagellar transport	K19656	intraflagellar t
evm.model.HIC_ASM_16.533	PREDICTED: wiskott-	Wiskott-Aldrich syndro	K06083	WAS protein f
evm.model.HIC_ASM_12.753	myotubularin-related p	Myotubularin-related p	K18082	myotubularin-
evm.model.HIC_ASM_5.703	bestrophin-3-like [Lyn	Bestrophin-3 OS=Hom	K13880	bestrophin-3 |
evm.model.HIC_ASM_8.377	Hypothetical predicted	Centrosomal protein of	--	
evm.model.HIC_ASM_13.301	protein ABHD1 [Puma	Protein ABHD1 OS=B	K13696	abhydrolase do
evm.model.HIC_ASM_4.318	jun dimerization protei	Jun dimerization protei	K09033	jun dimerizatio
evm.model.HIC_ASM_13.876	PREDICTED: protein k	Protein kinase C epsilo	K18050	novel protein k
evm.model.HIC_ASM_2.112	RNA-binding protein 4	RNA-binding protein 4	--	
evm.model.HIC_ASM_4.470	PREDICTED: putative	Putative E3 ubiquitin-p	K11979	E3 ubiquitin-p
evm.model.HIC_ASM_19.1377.3	ankyrin repeat and BTI	Ankyrin repeat and BT	K10520	ankyrin repeat
evm.model.HIC_ASM_1.865	ELL-associated factor 2	ELL-associated factor 2	K15186	ELL-associate
evm.model.HIC_ASM_20.91	ATPase family AAA d	ATPase family AAA d	K10754	replication fac
evm.model.HIC_ASM_21.949	potassium voltage-gate	Potassium voltage-gate	K04927	potassium volt
evm.model.HIC_ASM_10.591	LOW QUALITY PRO	Tissue-resident T-cell t	K09214	glass | (RefSec
evm.model.HIC_ASM_0.724	PREDICTED: mothers	Mothers against decape	K04676	mothers agains
evm.model.HIC_ASM_10.77	PREDICTED: 1-phosp	1-phosphatidylinositol	K19006	phosphatidylin
evm.model.HIC_ASM_1.311	PREDICTED: centroso	Centrosomal protein of	K16763	centrosomal pr
evm.model.HIC_ASM_6.712	PREDICTED: 2'-deoxy	2'-deoxynucleoside 5'-p	K22928	2'-deoxynucleo
evm.model.HIC_ASM_18.612	PREDICTED: exosome	Exosome complex com	K12590	exosome comp
evm.model.HIC_ASM_21.285	uncharacterized protein	Uncharacterized protei	--	
evm.model.HIC_ASM_7.1108	proto-oncogene DBL is	Proto-oncogene DBL O	K20685	guanine nucleo
evm.model.HIC_ASM_5.1773	regulator of nonsense t	Regulator of nonsense	K14327	regulator of no
evm.model.HIC_ASM_12.1224	PREDICTED: transme	Transmembrane protein	K17599	transmembran
evm.model.HIC_ASM_21.417	protein SOGA1 isoform	Protein SOGA1 OS=H	--	

evm.model.HIC_ASM_6.1213	nephrocan-like [Acino	Nephrocan OS=Mus m	K13023	carboxypeptid
evm.model.HIC_ASM_17.184	PREDICTED: MAP ki	MAP kinase-activated	K04443	mitogen-activa
evm.model.HIC_ASM_13.22.2	PREDICTED: peroxid	Peroxidasin homolog C	K19511	peroxidase [E
evm.model.HIC_ASM_7.432.7	WD repeat-containing	WD repeat-containing	K14963	COMPASS co
evm.model.HIC_ASM_19.231	dipeptidyl peptidase 9	Dipeptidyl peptidase 9	K08656	dipeptidyl-pep
evm.model.HIC_ASM_10.721	adhesion G protein-co	Adhesion G protein-co	K04597	adhesion G pr
evm.model.HIC_ASM_0.401	pulmonary surfactant-a	Pulmonary surfactant-a	--	
evm.model.HIC_ASM_20.861.4	septin-9 isoform X5 [	Septin-9 OS=Homo sa	K16938	septin 3/9/12 |
evm.model.HIC_ASM_13.694	ankyrin repeat domain-	Ankyrin repeat domain	K21441	ankyrin repeat
evm.model.HIC_ASM_21.40	napb protein [Lynx pa	Beta-soluble NSF attac	K15296	alpha-soluble
evm.model.HIC_ASM_1.1364.2	PREDICTED: periodic	Periodic tryptophan pr	K14558	periodic trypto
evm.model.HIC_ASM_14.641	cartilage acidic protein	Cartilage acidic protei	K17307	fibulin 1/2 | (R
evm.model.HIC_ASM_19.596.1	RAB8A isoform 2 [Pa	Ras-related protein Ra	K07901	Ras-related pr
evm.model.HIC_ASM_19.827	chondroitin sulfate pro	Chondroitin sulfate pr	K08116	chondroitin su
evm.model.HIC_ASM_18.1123	exocyst complex comp	Exocyst complex comp	K19987	exocyst compl
evm.model.HIC_ASM_2.729.1	methyltransferase-like	Methyltransferase-like	K07579	putative methy
evm.model.HIC_ASM_19.43.1	protein strawberry not	Protein strawberry not	K04425	mitogen-activa
evm.model.HIC_ASM_11.269	metabotropic glutamat	Metabotropic glutamat	K04605	metabotropic g
evm.model.HIC_ASM_8.423	PREDICTED: glutama	Glutamate receptor 4 C	K05200	glutamate rece
evm.model.HIC_ASM_19.775	lim domain-containing	LIM domain-containin	K16682	LIM domain-c
evm.model.HIC_ASM_0.89.1	PREDICTED: proline	Pyridoxal phosphate h	K06997	PLP dependen
evm.model.HIC_ASM_2.1527	PREDICTED: RNA po	RNA polymerase I-spe	K15216	RNA polymer
evm.model.HIC_ASM_10.1100	PREDICTED: enoyl-C	Enoyl-CoA hydratase	K05607	methylglutaco
evm.model.HIC_ASM_4.101	leucine-rich repeat-con	Leucine-rich repeat-co	K16606	leucine-rich re
evm.model.HIC_ASM_5.179	PREDICTED: beta-par	Beta-parvin OS=Homo	K06275	parvin | (RefS
evm.model.HIC_ASM_14.173	PREDICTED: cilia- an	Cilia- and flagella-asso	K03910	alpha-2-macro
evm.model.HIC_ASM_3.901	ankyrin repeat and dea	Ankyrin repeat and de	K03511	DNA polymer
evm.model.HIC_ASM_10.1728	cornulin isoform X2 [	Cornulin OS=Homo sa	K18626	trichohyalin | (
evm.model.HIC_ASM_0.838.7	exosome complex com	Exosome complex com	K03678	exosome comp
evm.model.HIC_ASM_18.1020	protein NLRC5 isofor	Protein NLRC5 OS=H	K22615	NLR family C
evm.model.HIC_ASM_17.392	ras GTPase-activating	Ras GTPase-activating	K17633	RAS protein a
evm.model.HIC_ASM_12.370	PREDICTED: GRB2-a	GRB2-associated and	--	
evm.model.HIC_ASM_2.2084	protein sidekick-1 [Fe	Protein sidekick-1 OS=	K16353	protein sideki
evm.model.HIC_ASM_10.256	TAR DNA-binding pro	TAR DNA-binding pro	K13044	heterogeneous
evm.model.HIC_ASM_21.183	PREDICTED: RING fi	RING finger protein 2	K15699	RING finger p
evm.model.HIC_ASM_20.1264_	PREDICTED: GH3 do	GH3 domain-containin	K11224	signal transdu
evm.model.HIC_ASM_20.223	PREDICTED: alpha-2-	Alpha-2-antiplasmin O	K03983	alpha-2-antipl
evm.model.HIC_ASM_10.264	serine/threonine-protei	Serine/threonine-protei	K07203	serine/threoni
evm.model.HIC_ASM_11.461	MAGUK p55 subfamil	MAGUK p55 subfamil	K06103	calcium/calmo
evm.model.HIC_ASM_8.151	centrosomal protein of	Centrosomal protein of	K16462	centrosomal p
evm.model.HIC_ASM_3.695	drebrin isoform X2 [A	Drebrin OS=Homo sap	K20520	drebrin-like p
evm.model.HIC_ASM_18.1088	protein bean-like [Lyn	Protein BEAN1 OS=H	K19324	protein BEAN
evm.model.HIC_ASM_13.817	clathrin heavy chain li	Clathrin heavy chain li	K04646	clathrin heavy
evm.model.HIC_ASM_8.1232.2	PREDICTED: nuclear	Nuclear pore complex	K14303	nuclear pore c

evm.model.HIC_ASM_2.2097	PREDICTED: BRCA1	BRCA1-associated AT	K23112	BRCA1-assoc
evm.model.HIC_ASM_0.129	RNA-binding protein v	RNA-binding protein v	K02728	20S proteasom
evm.model.HIC_ASM_9.30.3	taperin-partial [Lynx p	Taperin OS=Mus musc	K17559	phostensin | (F
evm.model.HIC_ASM_2.2039	delphilin [Acinonyx ju	Delphilin OS=Homo sa	K13806	sn1-specific di
evm.model.HIC_ASM_6.1432	monofunctional C1-tet	Monofunctional C1-tet	K13402	monofunctiona
evm.model.HIC_ASM_12.681	beta-adrenergic recepto	Beta-adrenergic recepto	K00910	beta-adrenergi
evm.model.HIC_ASM_10.1803.3	u4 u6 small nuclear [L	U4/U6 small nuclear ri	K12843	U4/U6 small n
evm.model.HIC_ASM_12.648	protein phosphatase 1F	Protein phosphatase 1F	K17502	protein phosph
evm.model.HIC_ASM_20.1509.1	acyl-CoA synthetase fa	Acyl-CoA synthetase fa	K00666	fatty-acyl-CoA
evm.model.HIC_ASM_0.642	acid-sensing ion chann	Acid-sensing ion chann	K04832	acid-sensing io
evm.model.HIC_ASM_4.1176	protein phosphatase 1 r	Protein phosphatase 1 r	K17557	protein phosph
evm.model.HIC_ASM_2.357	PREDICTED: zinc fing	Zinc finger protein 142	K09228	KRAB domain
evm.model.HIC_ASM_19.788	FYVE and coiled-coil (	FYVE and coiled-coil (	K21954	FYVE and coi
evm.model.HIC_ASM_17.1045	protein elys [Lynx par	Protein ELYS OS=Hor	--	
evm.model.HIC_ASM_5.815.1	PREDICTED: NGFI-A	NGFI-A-binding protei	K22388	NGFI-A-bindi
evm.model.HIC_ASM_5.762_evi	PREDICTED: protein p	Protein phosphatase 1F	K17503	protein phosph
evm.model.HIC_ASM_10.1024	MAP kinase-interacting	MAP kinase-interacting	K04372	MAP kinase in
evm.model.HIC_ASM_6.541	ankyrin repeat and SAM	Ankyrin repeat and SA	K21413	ankyrin repeat
evm.model.HIC_ASM_12.537	nucleoporin seh1 isofo	Nucleoporin SEH1 OS	K14299	nucleoporin SE
evm.model.HIC_ASM_1.1227	PREDICTED: SET dor	SET domain-containing	K19199	histone-lysine
evm.model.HIC_ASM_6.135	ataxin-1 [Ursus arctos	Ataxin-1 OS=Rattus no	K14718	solute carrier f
evm.model.HIC_ASM_12.1217	transmembrane protein	Transmembrane protei	K17599	transmembran
evm.model.HIC_ASM_6.1358	transcription factor HIV	Transcription factor HI	K09239	human immun
evm.model.HIC_ASM_18.1043.1	adhesion G protein-cou	Adhesion G protein-co	K08452	adhesion G-pr
evm.model.HIC_ASM_14.558	PSD [Cervus elaphus h	PH and SEC7 domain-	K12494	PH and SEC7
evm.model.HIC_ASM_8.464	PREDICTED: probable	Probable G-protein cou	K04210	G protein-coup
evm.model.HIC_ASM_19.1337	uncharacterized protein	Uncharacterized protei	--	
evm.model.HIC_ASM_12.575	PREDICTED: guanine	Guanine nucleotide-bin	K16482	centriolar prot
evm.model.HIC_ASM_10.1211	PREDICTED: phospho	Phosphoglucomutase-1	K01835	phosphogluco
evm.model.HIC_ASM_12.215.1	Thioredoxin-like protei	Thioredoxin-like protei	K03671	thioredoxin 1 |
evm.model.HIC_ASM_13.180	neuroblastoma-amplifi	Neuroblastoma-amplifi	K20473	neuroblastoma
evm.model.HIC_ASM_2.1841	CAP-Gly domain-conta	CAP-Gly domain-conta	K10422	CAP-Gly dom
evm.model.HIC_ASM_11.1061.4	pax-interacting protein	PAX-interacting protei	K14972	PAX-interacti
evm.model.HIC_ASM_11.714.1	nuclear-interacting part	Nuclear-interacting par	--	
evm.model.HIC_ASM_0.992	netrin receptor UNC5C	Netrin receptor UNC5C	K07521	netrin receptor
evm.model.HIC_ASM_16.22.2	growth hormone-regula	Growth hormone-regul	K19953	TBC1 domain
evm.model.HIC_ASM_13.346	speedy protein A isofo	Speedy protein A OS=I	K08694	speedy | (RefS
evm.model.HIC_ASM_18.594.1	multiple epidermal gro	Multiple epidermal gro	K23330	leucine-zipper
evm.model.HIC_ASM_17.532	ubiquitin carboxyl-term	Ubiquitin carboxyl-term	K05610	ubiquitin carbo
evm.model.HIC_ASM_10.1594	probable ATP-depende	Probable ATP-depende	K13131	ATP-dependen
evm.model.HIC_ASM_5.665	PREDICTED: palmitoy	Palmitoyltransferase Z	K20032	palmitoyltrans
evm.model.HIC_ASM_20.1188	PREDICTED: ataxin-7	Ataxin-7-like protein 3	K11363	SAGA-associa
evm.model.HIC_ASM_9.163	hypothetical protein PA	Protein O-mannosyl-tra	K00728	dolichyl-phosp
evm.model.HIC_ASM_1.1196	PREDICTED: interfero	Interferon alpha/beta re	K05130	interferon rece

evm.model.HIC_ASM_1.772	protein FAM43A [Cal	Protein FAM43A OS=1	K12474	low density lip
evm.model.HIC_ASM_20.1278.8	PREDICTED: dnaJ hor	DnaJ homolog subfami	K09527	DnaJ homolog
evm.model.HIC_ASM_18.1229	PH domain leucine-ricl	PH domain leucine-ricl	K16340	PH domain an
evm.model.HIC_ASM_1.26.3	SNF-related serine/thre	SNF-related serine/thre	K08802	SNF related ki
evm.model.HIC_ASM_18.393_ev	PREDICTED: MEF2-a	MEF2-activating motif	K06240	laminin, alpha
evm.model.HIC_ASM_17.151	innate immunity activa	Innate immunity activa	K08405	G protein-coup
evm.model.HIC_ASM_13.873.1	endothelial PAS domai	Endothelial PAS doma	K09095	hypoxia-induc
evm.model.HIC_ASM_12.723	tetratricopeptide repeat	Tetratricopeptide repea	K15837	G-protein sign
evm.model.HIC_ASM_2.1858.1	protein ABHD11 isofo	Protein ABHD11 OS=I	K13703	abhydrolase do
evm.model.HIC_ASM_11.619	cystic fibrosis transmen	Cystic fibrosis transme	K05031	cystic fibrosis
evm.model.HIC_ASM_2.335.3	AN1-type zinc finger p	AN1-type zinc finger p	K08582	calpain-15 [EC
evm.model.HIC_ASM_4.10.1	PREDICTED: nuclear	Nuclear export mediato	--	
evm.model.HIC_ASM_20.692.2	PREDICTED: T-cell an	T-cell antigen CD7 OS	K06457	T-cell surface
evm.model.HIC_ASM_9.648	prostate-associated mic	Prostate-associated mic	--	
evm.model.HIC_ASM_13.356	PREDICTED: protein y	Protein yippee-like 5 O	K23339	protein yippee
evm.model.HIC_ASM_5.1170_ev	PREDICTED: proton n	Proton myo-inositol co	K08150	MFS transport
evm.model.HIC_ASM_4.1906	alpha-protein kinase 3	Alpha-protein kinase 3	K08868	alpha-kinase |
evm.model.HIC_ASM_5.554.4	ETS domain-containing	ETS domain-containing	K09430	ETS domain-c
evm.model.HIC_ASM_0.1500	biorientation of chromo	Biorientation of chrom	--	
evm.model.HIC_ASM_15.181_ev	PREDICTED: double-s	Double-stranded RNA-	K17597	double-strande
evm.model.HIC_ASM_7.433.2	PREDICTED: wiskott-	Wiskott-Aldrich syndro	K05747	Wiskott-Aldric
evm.model.HIC_ASM_11.801.1	putative transporter SV	Putative transporter SV	K06258	MFS transport
evm.model.HIC_ASM_19.73.3	ADAMTS-like protein	ADAMTS-like protein	K08630	a disintegrin a
evm.model.HIC_ASM_7.160	histone-binding protein	Histone-binding protein	K11659	histone-bindin
evm.model.HIC_ASM_9.230	PREDICTED: mitogua	Mitoguardin 2 OS=Bos	--	
evm.model.HIC_ASM_10.604	unnamed protein produ	Keratinocyte differenti	K23346	keratinocyte d
evm.model.HIC_ASM_16.554	tumor necrosis factor re	Tumor necrosis factor r	K05155	tumor necrosis
evm.model.HIC_ASM_8.1085	PREDICTED: ETS-rel	ETS-related transcripti	K17101	E74-like factor
evm.model.HIC_ASM_18.419	coiled-coil domain-con	Coiled-coil domain-con	--	
evm.model.HIC_ASM_2.1168	intraflagellar transport	Intraflagellar transport	K19672	intraflagellar t
evm.model.HIC_ASM_16.379	PREDICTED: histone-	Histone-lysine N-methy	K18494	histone-lysine
evm.model.HIC_ASM_20.996	PREDICTED: unchara	Uncharacterized protei	K02130	F-type H+-tran
evm.model.HIC_ASM_0.1624	PREDICTED: G protei	G protein-coupled rece	K08291	G protein-coup
evm.model.HIC_ASM_11.1095	nucleolar MIF4G doma	Nucleolar MIF4G dom	K17583	nucleolar MIF
evm.model.HIC_ASM_20.394	leucine-rich repeat-con	Leucine-rich repeat-con	K03352	anaphase-prom
evm.model.HIC_ASM_12.1084.2	PPP1CC isoform 6 [Pa	Serine/threonine-protei	K06269	serine/threonin
evm.model.CTG_1467.19	myb-binding protein 1A	Myb-binding protein 1.	K02331	DNA polymer
evm.model.HIC_ASM_10.735	PREDICTED: LOW Q	IQ domain-containing p	--	
evm.model.HIC_ASM_13.297	PREDICTED: cytosoli	Cytosolic carboxypepti	K23438	cytosolic carbo
evm.model.HIC_ASM_18.912	myosin light chain kina	Myosin light chain kina	K00907	myosin-light-c
evm.model.HIC_ASM_1.929	PREDICTED: coiled-c	Coiled-coil domain-con	--	
evm.model.HIC_ASM_20.450	manganese-dependent	Manganese-dependent	K01517	manganese-de
evm.model.HIC_ASM_4.1902	PREDICTED: neurome	Neuromedin-B OS=Ho	K05223	neuromedin B
evm.model.HIC_ASM_2.1479	PREDICTED: G-protei	G-protein coupled rece	K04619	G protein-coup

evm.model.HIC_ASM_11.123.1	PREDICTED: LOW Q	Uridine phosphorylase	K00757	uridine phospl
evm.model.HIC_ASM_14.104.2	ectodysplasin-A recept	Ectodysplasin-A recept	K23324	ectodysplasin-
evm.model.HIC_ASM_18.566.1	DNA repair protein XF	DNA repair protein XF	K10803	DNA-repair pr
evm.model.HIC_ASM_5.991	PREDICTED: keratin,	Keratin, type II cytoske	K07605	type II keratin,
evm.model.HIC_ASM_3.987	hypothetical protein PA	Importin-11 OS=Homo	K20224	importin-9 | (R
evm.model.HIC_ASM_0.1038	PREDICTED: dentin s	Dentin sialophosphopro	K08899	lemur tyrosine
evm.model.HIC_ASM_13.699	factor in the germline a	Factor in the germline a	K09066	factor in the ge
evm.model.HIC_ASM_8.1764.2	PREDICTED: Parkinso	Glutamine amidotransf	K06537	CD151 antigen
evm.model.HIC_ASM_4.1154	PREDICTED: 1-phosp	1-phosphatidylinositol	K05858	phosphatidylin
evm.model.HIC_ASM_19.889	hepatocyte growth fact	Hepatocyte growth fact	K23441	hepatocyte gro
evm.model.HIC_ASM_1.37	PREDICTED: cartilage	Cartilage-associated pr	K19606	cartilage-asso
evm.model.HIC_ASM_8.173	myelin protein zero-lik	Myelin protein zero-lik	K06770	myelin protein
evm.model.HIC_ASM_3.893	proteinase-activated re	Proteinase-activated re	K03914	coagulation fa
evm.model.HIC_ASM_6.1148_ev	methyltransferase-like	Methyltransferase-like	K12816	pre-mRNA-pro
evm.model.HIC_ASM_5.568.1	and ph [Lynx pardinus	FYVE, RhoGEF and P	K05724	FYVE, RhoGE
evm.model.HIC_ASM_2.264	PREDICTED: collagen	Collagen alpha-4(IV) c	K06237	collagen, type
evm.model.HIC_ASM_11.796	cyclic AMP-responsive	Cyclic AMP-responsiv	K09048	cyclic AMP-re
evm.model.HIC_ASM_20.1402.1	39S ribosomal protein	39S ribosomal protein	K17426	large subunit r
evm.model.HIC_ASM_0.639	cathepsin O [Puma con	Cathepsin O OS=Homo	K01374	cathepsin O [E
evm.model.HIC_ASM_12.389	aquaporin-4 isoform X	Aquaporin-4 OS=Bos t	K09866	aquaporin-4 | (
evm.model.HIC_ASM_10.327	kazrin isoform X2 [Za	Kazrin OS=Homo sapi	K07762	pappalysin-1 [
evm.model.HIC_ASM_11.219	origin recognition com	Origin recognition com	K02607	origin recogni
evm.model.HIC_ASM_14.1034	tetraspanin-15 isoform	Tetraspanin-15 OS=Bo	K17297	tetraspanin-15
evm.model.HIC_ASM_10.37	39S ribosomal protein	39S ribosomal protein	K02887	large subunit r
evm.model.HIC_ASM_16.157	ubiquitin-associated do	Ubiquitin-associated d	K14589	cap1 methyltra
evm.model.HIC_ASM_13.568.1	vitamin K-dependent g	Vitamin K-dependent g	K10106	vitamin K-dep
evm.model.HIC_ASM_8.608	PREDICTED: kelch-lik	Kelch-like protein 35 C	K10461	kelch-like prot
evm.model.HIC_ASM_8.1200	zinc finger protein 408	Zinc finger protein 408	K06792	aggrecan 1 | (C
evm.model.HIC_ASM_17.980	LOW QUALITY PRO	Calpain-8 OS=Homo s	K08577	calpain-8 [EC:
evm.model.HIC_ASM_13.489.4	PREDICTED: bcl-2-lik	Bcl-2-like protein 11 O	K16341	Bcl-2-like prot
evm.model.HIC_ASM_17.994.1	WD repeat-containing	WD repeat-containing	K22382	WD repeat-con
evm.model.CTG_1467.25	ubiquitin-conjugating e	Ubiquitin-conjugating	K10575	ubiquitin-conj
evm.model.HIC_ASM_10.1764.1	PREDICTED: phospha	Phosphatidylinositol 4-	K19801	phosphatidylin
evm.model.HIC_ASM_16.447.1	spartin [Puma concolo	Spartin OS=Homo sapi	K19366	spartin | (RefS
evm.model.HIC_ASM_17.52	neurofascin isoform X5	Neurofascin OS=Homo	K06757	neurofascin | (
evm.model.HIC_ASM_2.2026	hypothetical protein PA	Vacuolar fusion protein	K08269	serine/threonin
evm.model.HIC_ASM_3.531	hypothetical protein Ca	Interleukin-12 subunit	K05425	interleukin 12I
evm.model.HIC_ASM_19.418	PREDICTED: non-rece	Non-receptor tyrosine-	K11219	non-receptor t
evm.model.HIC_ASM_0.111_evi	pro-neuregulin-1, mem	Pro-neuregulin-1, mem	K05455	neuregulin 1 |
evm.model.HIC_ASM_10.563	membrane progestin re	Membrane progestin re	K17387	kinesin family
evm.model.HIC_ASM_2.1939	PREDICTED: acetylch	Acetylcholinesterase O	K01049	acetylcholines
evm.model.HIC_ASM_2.860	methylmalonic aciduria	Methylmalonic aciduri	K22538	mitochondrial
evm.model.HIC_ASM_8.208	PREDICTED: vacuola	Vacuolar protein sortin	K20179	vacuolar prote
evm.model.HIC_ASM_1.842	PREDICTED: adenyla	Adenylate cyclase type	K08045	adenylate cycl

evm.model.HIC_ASM_17.821.4	PREDICTED: protein	Protein RRNAD1 OS=	--	
evm.model.HIC_ASM_17.455	PREDICTED: protein	Protein PRRC2C OS=H	K13219	splicing factor
evm.model.HIC_ASM_8.333	Friend leukemia integr	Friend leukemia integr	K09436	friend leukemi
evm.model.HIC_ASM_6.713	tau tubulin kinase 1, is	Tau-tubulin kinase 1 O	K08815	tau tubulin kin
evm.model.HIC_ASM_10.950.2	Mediator of RNA poly	Mediator of RNA poly	K15129	mediator of RI
evm.model.HIC_ASM_6.1023	PREDICTED: LYR mo	LYR motif-containing	K12824	transcription e
evm.model.HIC_ASM_4.107	PREDICTED: protein	Protein SIX6OS1 OS=	--	
evm.model.HIC_ASM_6.1673	TATA-box-binding pro	TATA-box-binding pro	K03120	transcription i
evm.model.HIC_ASM_15.751.5	poly(U)-binding-splici	Poly(U)-binding-splici	K12838	poly(U)-bindi
evm.model.HIC_ASM_19.1032	protein TASOR isofor	Protein TASOR OS=H	K21873	protein TASO
evm.model.HIC_ASM_6.431	tenascin-X isoform X1	Tenascin-X OS=Homo	K06252	tenascin | (Ref
evm.model.HIC_ASM_18.976	PREDICTED: retinobl	Retinoblastoma-like pr	K16332	retinoblastoma
evm.model.HIC_ASM_21.225	transmembrane protein	Transmembrane protei	--	
evm.model.HIC_ASM_5.1541	voltage-dependent calc	Voltage-dependent calc	K04861	voltage-depen
evm.model.HIC_ASM_18.1109	hypothetical protein D	Cytosolic iron-sulfur a	K07374	tubulin alpha |
evm.model.HIC_ASM_8.854	DENN domain-contain	DENN domain-contain	K20164	DENN domain
evm.model.HIC_ASM_18.385	protein phosphatase 1	Protein phosphatase 1	K14019	protein phosph
evm.model.HIC_ASM_13.157.1	transcription factor E2	Transcription factor E2	K09390	transcription f
evm.model.HIC_ASM_5.1177	PREDICTED: kinesin-	Kinesin-like protein K	K10395	kinesin family
evm.model.HIC_ASM_6.598	PREDICTED: transme	Transmembrane protei	--	
evm.model.HIC_ASM_12.1090	cell cycle checkpoint c	Cell cycle checkpoint c	K10995	cell cycle chec
evm.model.HIC_ASM_8.935	ATP-sensitive inward r	ATP-sensitive inward r	K05004	potassium inw
evm.model.HIC_ASM_6.833	PREDICTED: F-box o	F-box only protein 9 O	K10295	F-box protein
evm.model.HIC_ASM_12.1133	PREDICTED: coiled-c	Coiled-coil domain-cor	K20040	huntingtin-inte
evm.model.HIC_ASM_12.604	PREDICTED: kelch-lil	Kelch-like protein 22 C	K10459	kelch-like prot
evm.model.HIC_ASM_14.488	gamma-adducin isofor	Gamma-adducin OS=H	K18622	adducin | (Ref
evm.model.HIC_ASM_19.752	YjeF N-terminal doma	YjeF N-terminal doma	K11353	NADH dehydr
evm.model.HIC_ASM_16.122	DNA repair protein co	DNA repair protein co	K10846	DNA excision
evm.model.HIC_ASM_19.35	WD repeat-containing	WD repeat-containing	K14829	pre-rRNA-pro
evm.model.HIC_ASM_2.990	zinc finger protein GL	Zinc finger protein GL	K16798	zinc finger pro
evm.model.HIC_ASM_19.1224	PREDICTED: cysteine	Cysteine-rich with EG	K17675	ATP-depende
evm.model.HIC_ASM_9.1035	zinc finger protein GL	Zinc finger protein GL	K09232	zinc finger pro
evm.model.HIC_ASM_20.933	adp-ribosylation factor	ADP-ribosylation facto	K12404	ADP-ribosylat
evm.model.HIC_ASM_6.68.2	translocon-associated p	Translocon-associated	K13249	translocon-ass
evm.model.HIC_ASM_20.1089.2	SWI/SNF-related matri	SWI/SNF-related matri	K11650	SWI/SNF-rela
evm.model.HIC_ASM_5.943	anti-Muellerian hormo	Anti-Muellerian hormo	K04672	anti-mullerian
evm.model.HIC_ASM_10.501	transcription elongatio	Transcription elongatic	K03145	transcription e
evm.model.HIC_ASM_1.73	deleted in lung and eso	Deleted in lung and esc	K17570	hydrocephalus
evm.model.HIC_ASM_9.616	PREDICTED: FERM a	FERM and PDZ domai	K05725	focal adhesion
evm.model.HIC_ASM_19.473	tartrate-resistant acid p	Tartrate-resistant acid	K14379	tartrate-resista
evm.model.CTG_180.12.2	low quality protein: ox	Oxysterol-binding prot	K20464	oxysterol-bind
evm.model.HIC_ASM_20.1225	PREDICTED: rho-rela	Rho-related GTP-bindi	K07858	Rho family G
evm.model.HIC_ASM_10.1689	PREDICTED: T-box tr	T-box transcription fac	K10182	T-box protein
evm.model.HIC_ASM_9.454.1	zinc finger protein 618	Zinc finger protein 618	K09228	KRAB domain

evm.model.HIC_ASM_9.1123	PREDICTED: SHC-tra	SHC-transforming prot	K17448	SHC-transforn
evm.model.HIC_ASM_20.1513	Hypothetical predicted	MYCBP-associated pro	--	
evm.model.HIC_ASM_2.929	Hypothetical predicted	Nck-associated protein	--	
evm.model.HIC_ASM_12.358.1	dystrobrevin alpha isof	Dystrobrevin alpha OS	K10366	dystrophin | (R
evm.model.HIC_ASM_7.136	PREDICTED: vasculai	Vascular endothelial gı	K05449	vascular endot
evm.model.HIC_ASM_10.672	receptor-type tyrosine-]	Receptor-type tyrosine-	K16662	receptor-type t
evm.model.HIC_ASM_6.1018	PREDICTED: ubiquiti	Ubiquitin-conjugating	K10578	ubiquitin-conj
evm.model.HIC_ASM_21.956_ev	PREDICTED: helicase	Helicase with zinc fing	K18422	helicase MOV
evm.model.HIC_ASM_1.467	probable G-protein cou	Probable G-protein cou	K08433	G protein-coup
evm.model.HIC_ASM_14.477	RNA-binding protein 2	RNA-binding protein 2	K13213	matrin 3 | (Ref
evm.model.HIC_ASM_9.916.3	PREDICTED: G kinase	G kinase-anchoring pro	K04523	ubiquilin | (Re
evm.model.HIC_ASM_12.316	urea transporter 2 [Pur	Urea transporter 2 OS=	K08716	solute carrier f
evm.model.HIC_ASM_9.251	outer dense fiber protei	Outer dense fiber prote	K16479	outer dense fib
evm.model.HIC_ASM_7.53	PREDICTED: anosmin	Anosmin-1 OS=Homo	K23413	| (RefSeq) AN
evm.model.HIC_ASM_10.1326	PREDICTED: WD rep	WD repeat-containing	K17770	mitochondrial
evm.model.HIC_ASM_2.1545	modulator of smoothen	Modulator of smoothen	K18328	lariat debranch
evm.model.HIC_ASM_3.590	PREDICTED: gamma-	Gamma-aminobutyric a	K05189	gamma-amino
evm.model.HIC_ASM_8.837	PREDICTED: tubby pı	Tubby protein homolog	K19600	tubby-related p
evm.model.HIC_ASM_13.579	PREDICTED: transcrip	Transcription factor 7-l	K04490	transcription fa
evm.model.HIC_ASM_8.462	PREDICTED: ankyrin	Ankyrin repeat domain	K21439	ankyrin repeat
evm.model.HIC_ASM_14.91	zona pellucida sperm-b	Zona pellucida sperm-b	K19926	zona pellucida
evm.model.HIC_ASM_13.443.1	protein kinase domain-	Extracellular tyrosine-p	K17548	extracellular ty
evm.model.HIC_ASM_8.1590.1	copper chaperone for s	Copper chaperone for s	K04569	copper chapero
evm.model.HIC_ASM_11.686	HILPDA [Cervus elap	Hypoxia-inducible lipi	--	
evm.model.HIC_ASM_6.748	calpain-11 isoform X1	Calpain-11 OS=Homo	K08580	calpain-11 [EC
evm.model.HIC_ASM_0.1315	PREDICTED: tyrosine	Tyrosine-protein kinase	K08016	tyrosine-protei
evm.model.HIC_ASM_21.102	ribosome-binding prote	Ribosome-binding prot	K14000	ribosome-bind
evm.model.HIC_ASM_10.927	unnamed protein produ	Zinc finger MYND dor	--	
evm.model.HIC_ASM_11.1124	vasoactive intestinal pc	Vasoactive intestinal p	K04590	vasoactive inte
evm.model.HIC_ASM_10.1818.2	PREDICTED: myotubı	Myotubularin-related p	K18085	myotubularin-
evm.model.HIC_ASM_21.729	zinc finger protein 217	Zinc finger protein 217	K22411	zinc finger pro
evm.model.HIC_ASM_0.1189	PREDICTED: enameli	Enamelin OS=Sus scro	K23444	enamelin | (Re
evm.model.HIC_ASM_18.502_ev	PREDICTED: optic atı	Optic atrophy 3 proteir	K23166	optic atrophy 3
evm.model.HIC_ASM_11.765	non-muscle caldesmon	Non-muscle caldesmor	K12327	caldesmon | (R
evm.model.HIC_ASM_1.1139	A disintegrin and meta	A disintegrin and meta	K08620	a disintegrin a
evm.model.HIC_ASM_3.863	spermatogenic leucine	Spermatogenic leucine	K17482	spermatogenic
evm.model.HIC_ASM_3.1132	PREDICTED: anion ex	Anion exchange protei	K13860	solute carrier f
evm.model.HIC_ASM_1.248	PREDICTED: polypep	Polypeptide N-acetylga	K00710	polypeptide N
evm.model.HIC_ASM_2.452	putative malate dehydr	Putative malate dehydr	K00025	malate dehydr
evm.model.HIC_ASM_0.444.1	BTB/POZ domain-cont	BTB/POZ domain-cont	K21919	BTB/POZ dom
evm.model.HIC_ASM_1.679	PREDICTED: mitogen	Mitogen-activated prot	K04422	mitogen-activa
evm.model.HIC_ASM_18.645	PREDICTED: unchara	Uncharacterized protei	--	
evm.model.HIC_ASM_21.208.3	DDRGK domain-conta	DDRGK domain-conta	K23344	DDRGK doma
evm.model.HIC_ASM_0.302	PREDICTED: microce	Microcephalin OS=Pai	K19403	microcephalin

evm.model.HIC_ASM_10.503	PREDICTED: transcri[	Transcription factor E2	K09389	transcription f
evm.model.HIC_ASM_20.625	LOW QUALITY PRO	Protein INCA1 OS=Hc	--	
evm.model.HIC_ASM_8.1568.2	PREDICTED: splicing	Splicing factor 3B subu	K12829	splicing factor
evm.model.HIC_ASM_6.382	hypothetical protein Ca	NF-kappa-B inhibitor-l	K09256	NF-kappa-B in
evm.model.HIC_ASM_19.768.1	tetranectin [Felis catus	Tetranectin OS=Homo	K17520	C-type lectin d
evm.model.HIC_ASM_17.728	PREDICTED: coiled-c	Cilia- and flagella-asso	--	
evm.model.HIC_ASM_10.986	protein patched homolo	Protein patched homolo	K11101	patched 2 | (R
evm.model.HIC_ASM_6.828	unnamed protein produ	Transmembrane protei	--	
evm.model.HIC_ASM_5.1018	PREDICTED: POU do	POU domain, class 6, t	K09368	POU domain t
evm.model.HIC_ASM_16.409	serine/threonine-protei	Serine/threonine-protei	K08857	NIMA (never
evm.model.HIC_ASM_18.631	PREDICTED: UPF069	UPF0692 protein C19o	--	
evm.model.HIC_ASM_10.1168	FYN-binding protein 2	FYN-binding protein 2	--	
evm.model.HIC_ASM_12.1160	LOW QUALITY PRO	Dynein heavy chain 10	K10408	dynein heavy c
evm.model.HIC_ASM_4.220	PREDICTED: sushi do	Sushi domain-containi	--	
evm.model.HIC_ASM_1.1152	MAP3K7 C-terminal-li	MAP3K7 C-terminal-li	K04427	mitogen-activa
evm.model.HIC_ASM_1.1015	PREDICTED: target of	Target of Nesh-SH3 O	K08592	sentrin-specifi
evm.model.HIC_ASM_20.832.3	PREDICTED: CDP-dia	CDP-diacylglycerol--gl	K00995	CDP-diacylgly
evm.model.HIC_ASM_10.741	PREDICTED: protein	Protein FAM167B OS=	--	
evm.model.HIC_ASM_21.925.3	MRG/MORF4L-bindir	MRG/MORF4L-bindir	K11343	MRG-binding
evm.model.HIC_ASM_10.742	tyrosine-protein kinase	Tyrosine-protein kinas	K05856	lymphocyte ce
evm.model.HIC_ASM_10.663	protein 4.1 isoform X1	Protein 4.1 OS=Homo	K06107	erythrocyte me
evm.model.HIC_ASM_1.376	PREDICTED: ribonucl	--	--	
evm.model.HIC_ASM_7.863	PREDICTED: short tra	Short transient recepto	K04968	transient recep
evm.model.HIC_ASM_14.438	attractin-like protein 1	Attractin-like protein 1	K23330	leucine-zipper
evm.model.HIC_ASM_14.577.1	protein DPCD isoform	Protein DPCD OS=Ho	K20800	protein DPCD
evm.model.HIC_ASM_0.761	unnamed protein produ	Calmegin OS=Canis lu	K09551	calmegin | (Re
evm.model.HIC_ASM_2.221	DUF3443 domain-cont	Spermatogenesis-assoc	K13912	deleted in mal
evm.model.HIC_ASM_2.485	PREDICTED: ribosom	Ribosome biogenesis p	K14863	ribosome biog
evm.model.HIC_ASM_1.576	PREDICTED: transme	Transmembrane protei	K22196	transmembran
evm.model.HIC_ASM_0.941	centromere-associated	Centromere-associated	K11498	centromeric pr
evm.model.HIC_ASM_5.1882	WD repeat-containing	WD repeat-containing	K21358	serine/threonin
evm.model.HIC_ASM_20.1022.1	inward rectifier potassi	Inward rectifier potassi	K05009	potassium inw
evm.model.HIC_ASM_5.1486	PREDICTED: fibrobla	Fibroblast growth facto	K04358	fibroblast grov
evm.model.HIC_ASM_6.1159.4	DNA polymerase zeta	DNA polymerase zeta	K02350	DNA polymer
evm.model.HIC_ASM_18.1060	PREDICTED: coiled-c	Coiled-coil domain-co	K14837	nucleolar prot
evm.model.HIC_ASM_18.1007	PREDICTED: LOW Q	Guanine nucleotide-bi	K04534	guanine nucle
evm.model.HIC_ASM_17.393	quinone oxidoreductas	Quinone oxidoreductas	K03650	tRNA modific
evm.model.HIC_ASM_14.111	nidogen-1 [Canis lupu	Nidogen-1 OS=Homo	K06826	nidogen (entac
evm.model.HIC_ASM_4.1372	PREDICTED: myocar	Myocardial zonula adh	K22031	myocardial zo
evm.model.HIC_ASM_20.899	envoplakin isoform X1	Envoplakin OS=Homo	K10383	envoplakin | (
evm.model.HIC_ASM_18.1144	Dihydroorotate dehydr	Dihydroorotate dehydr	K00254	dihydroorotate
evm.model.HIC_ASM_11.802	PREDICTED: V-type	V-type proton ATPase	K02154	V-type H+-tra
evm.model.HIC_ASM_3.681	histone-lysine n- [Lyn	Histone-lysine N-meth	K15588	histone-lysine
evm.model.HIC_ASM_2.605	zinc finger protein 804	Zinc finger protein 804	K01514	exopolyphospl

evm.model.HIC_ASM_20.1521.2	canalicular multispecif	Canalicular multispecif	K05667	ATP-binding c
evm.model.HIC_ASM_3.1023.1	interleukin-31 receptor	Interleukin-31 receptor	K22630	interleukin 31
evm.model.HIC_ASM_10.1635	pre-mrna-splicing facto	Pre-mRNA-splicing fac	K12861	pre-mRNA-sp
evm.model.HIC_ASM_5.1545	voltage-dependent L-ty	Voltage-dependent L-ty	K04850	voltage-depend
evm.model.HIC_ASM_19.767.2	exosome complex com	Exosome complex com	K12589	exosome comp
evm.model.HIC_ASM_20.1231	hypothetical protein DI	Alanyl-tRNA editing p	K07050	misacylated tR
evm.model.HIC_ASM_7.881	interleukin-13 receptor	Interleukin-13 receptor	K05077	interleukin 13
evm.model.HIC_ASM_13.400	serine threonine-protei	Serine/threonine-protei	K06070	protein kinase
evm.model.HIC_ASM_10.838.3	chromatin modification	Chromatin modificatio	K11344	chromatin mod
evm.model.HIC_ASM_8.1184	PREDICTED: PHD fin	PHD finger protein 21	K11643	chromodomain
evm.model.HIC_ASM_10.1055	metalloprotease TIKI2	Metalloprotease TIKI2	--	
evm.model.HIC_ASM_6.1109.3	uncharacterized protein	Uncharacterized protei	K12662	U4/U6 small r
evm.model.HIC_ASM_21.260	syntaphilin isoform X2	Syntaphilin OS=Homo	--	
evm.model.HIC_ASM_10.1012	PREDICTED: DNA re	DNA repair and recom	K10875	DNA repair an
evm.model.HIC_ASM_4.296	protein kiaa0317-like |	Apoptosis-resistant E3	K22370	apoptosis-resis
evm.model.HIC_ASM_14.507	PREDICTED: cilia- an	Cilia- and flagella-asso	K10381	desmoplakin |
evm.model.HIC_ASM_6.1589	c-c chemokine recepto	C-C chemokine recepto	K04181	C-C chemokin
evm.model.HIC_ASM_2.1838.2	general transcription fa	General transcription f	K03121	transcription i
evm.model.HIC_ASM_8.479	PREDICTED: deuteros	Deuterosome assembly	K16763	centrosomal p
evm.model.HIC_ASM_18.798	myelin-associated glyc	Myelin-associated glyc	K06771	myelin associa
evm.model.HIC_ASM_9.335	PREDICTED: protein	Protein SCAI OS=Hom	--	
evm.model.HIC_ASM_5.216	PREDICTED: cytochro	Cytochrome P450 2D1	K07414	cytochrome P4
evm.model.HIC_ASM_2.300	Ephrin type-A receptor	Ephrin type-A receptor	K05105	Eph receptor A
evm.model.HIC_ASM_4.819	PREDICTED: fibrous	Fibrous sheath CABYI	--	
evm.model.HIC_ASM_5.1101	histone deacetylase 7 is	Histone deacetylase 7 (	K11408	histone deacet
evm.model.HIC_ASM_10.851	type II inositol 1,4,5-tri	Type II inositol 1,4,5-t	K01099	inositol polyp
evm.model.HIC_ASM_6.564	PREDICTED: testis an	Testis anion transporte	K14705	solute carrier f
evm.model.HIC_ASM_11.948.4	PREDICTED: histone-	Histone-lysine N-meth	K11430	histone-lysine
evm.model.HIC_ASM_0.1213	PREDICTED: beta-cas	Beta-casein OS=Sus sc	K17107	beta-casein | (I
evm.model.HIC_ASM_14.635	pyridine nucleotide-dis	Pyridine nucleotide-dis	K00280	lysyl oxidase-l
evm.model.HIC_ASM_0.1369	rho-related GTP-bindir	Rho-related GTP-bindi	K07873	Ras homolog g
evm.model.HIC_ASM_21.385	ER degradation-enhanc	ER degradation-enhanc	K10085	ER degradatio
evm.model.HIC_ASM_21.463	protein FAM83D [Fel	Protein FAM83D OS=	K16805	protein FAM8
evm.model.HIC_ASM_4.410	PREDICTED: tyrosine	Tyrosine-protein phosp	K18025	tyrosine-protei
evm.model.HIC_ASM_10.999	PREDICTED: coiled-c	Coiled-coil domain-co	--	
evm.model.HIC_ASM_20.329	PREDICTED: TOM1-l	TOM1-like protein 2 C	K16677	dachs | (RefSe
evm.model.HIC_ASM_5.378.2	beta-1,3-N-acetylgluco	Beta-1,3-N-acetylgluco	K05948	fringe [EC:2.4
evm.model.HIC_ASM_6.1017	PREDICTED: gamma-	Gamma-aminobutyric a	K05190	gamma-amino
evm.model.HIC_ASM_2.1268.2	PREDICTED: host cell	Host cell factor C1 reg	--	
evm.model.HIC_ASM_8.6	CWF19-like protein 2 i	CWF19-like protein 2 (	K12318	guanylate cycl
evm.model.HIC_ASM_12.1080.2	LOW QUALITY PRO	Homeobox protein cut-	K09313	homeobox pro
evm.model.HIC_ASM_20.1153.7	PREDICTED: acyl-Co	Acyl-CoA-binding dom	K15189	protein HEXIN
evm.model.HIC_ASM_0.902	pro-epidermal growth f	Pro-epidermal growth f	K04357	epidermal grov
evm.model.HIC_ASM_5.1316	glutamate receptor iono	Glutamate receptor ion	K05210	glutamate rece

evm.model.HIC_ASM_1.548	MDS1 and EVI1 comp	Histone-lysine N-meth	K04462	ecotropic virus
evm.model.HIC_ASM_4.1250	serine incorporator 4 is	Serine incorporator 4 C	K00791	tRNA dimethy
evm.model.HIC_ASM_0.760	alpha-1,3-mannosyl-gl	Alpha-1,3-mannosyl-gl	K00738	alpha-1,3-man
evm.model.HIC_ASM_6.1434	A-kinase anchor protei	A-kinase anchor protei	K16528	A-kinase anch
evm.model.HIC_ASM_2.1328	PREDICTED: cell dea	Cell death-inducing p5	K19363	lipopolysaccha
evm.model.HIC_ASM_9.666	phospholipid-transport	Phospholipid-transport	K01530	phospholipid-t
evm.model.HIC_ASM_19.874	Hypothetical predicted	Coiled-coil domain-co	K06243	laminin, beta 2
evm.model.HIC_ASM_6.556	PREDICTED: armadill	Armadillo repeat-conta	--	
evm.model.HIC_ASM_0.1436	zinc finger CCHC dom	rRNA N6-adenosine-m	K02367	glucuronyl/N-
evm.model.HIC_ASM_17.126	leiomodin-1 [Acinony	Leiomodin-1 OS=Hom	K22030	leiomodin | (R
evm.model.HIC_ASM_8.643	pleckstrin homology do	Pleckstrin homology do	K06569	melanoma-ass
evm.model.HIC_ASM_6.1457	PREDICTED: LOW Q	Mu-type opioid recepto	K04215	mu-type opioi
evm.model.HIC_ASM_18.1231	tyrosine aminotransfera	Tyrosine aminotransfe	K00815	tyrosine amino
evm.model.HIC_ASM_9.32	anaphase-promoting co	Anaphase-promoting c	K03349	anaphase-pron
evm.model.HIC_ASM_12.308	proline-serine-threonin	Proline-serine-threonin	K20124	proline-serine-
evm.model.HIC_ASM_17.641	PREDICTED: serine/th	Serine/threonine-protei	K08877	serine/threonin
evm.model.HIC_ASM_8.341	PREDICTED: PR dom	PR domain zinc finger	K20796	histone-lysine
evm.model.HIC_ASM_1.317	ephrin type-B receptor	Ephrin type-B receptor	K05110	Eph receptor B
evm.model.HIC_ASM_13.690.6	zinc finger protein 638	Zinc finger protein 638	K13213	matrin 3 | (Ref
evm.model.HIC_ASM_1.1041	gamma-aminobutyric a	Gamma-aminobutyric a	K05190	gamma-amino
evm.model.HIC_ASM_14.533	LOW QUALITY PRO	Alpha-internexin OS=H	K07608	internexin neu
evm.model.HIC_ASM_0.250	protein WWC2 isoform	Protein WWC2 OS=Ho	K16685	protein KIBRA
evm.model.HIC_ASM_9.279.6	alpha-N-acetyl-neuram	Alpha-N-acetyl-neuran	K03374	N-acetylgalact
evm.model.HIC_ASM_11.816	killer cell lectin-like re	Killer cell lectin-like re	K22681	c-type lectin d
evm.model.HIC_ASM_14.1050	gamma-1-syntrophin [	DNA replication ATP-	K10742	DNA replicati
evm.model.HIC_ASM_0.1546	wolframin [Felis catus	Wolframin OS=Mus m	K14020	wolfamin | (Re
evm.model.HIC_ASM_18.1116	UPF0183 protein C16o	UPF0183 protein C16o	--	
evm.model.HIC_ASM_0.24	neuronal acetylcholine	Neuronal acetylcholine	K04814	nicotinic acety
evm.model.HIC_ASM_7.1190.15	PREDICTED: synaptic	Synaptic functional reg	K15516	fragile X ment
evm.model.HIC_ASM_5.677	PREDICTED: calcyph	Calcyphosin-2 OS=Ho	--	
evm.model.HIC_ASM_19.180	tight junction protein Z	Tight junction protein Z	K06097	tight junction p
evm.model.HIC_ASM_15.648	PREDICTED: DENN d	DENN domain-contain	K20162	DENN domain
evm.model.HIC_ASM_20.922.3	PREDICTED: tRNA-sp	tRNA-splicing endonu	K15326	tRNA-splicing
evm.model.HIC_ASM_16.223	unnamed protein produ	RING finger protein 21	K10667	E3 ubiquitin-p
evm.model.HIC_ASM_20.324	PREDICTED: retinoic	Retinoic acid-induced p	K19749	retinoic acid-in
evm.model.HIC_ASM_18.1119	PREDICTED: F-box/L	F-box/LRR-repeat prot	K10274	F-box and leuc
evm.model.HIC_ASM_13.1031	Hypothetical predicted	Coiled-coil domain-co	K11167	dehydrogenase
evm.model.HIC_ASM_14.828.1	synaptotagmin-15-like	Synaptotagmin-15 OS=	K19914	synaptotagmin
evm.model.HIC_ASM_13.122	hypothetical protein D	Cleavage and polyaden	K14403	cleavage and p
evm.model.HIC_ASM_13.948.1	39s ribosomal protein	39S ribosomal protein	K02907	large subunit r
evm.model.HIC_ASM_16.482	mesenteric estrogen-de	Mesenteric estrogen-de	--	
evm.model.HIC_ASM_18.901_ev	phosphorylase b kinase	Phosphorylase b kinase	K07190	phosphorylase
evm.model.HIC_ASM_15.284	PREDICTED: unchara	Uncharacterized protei	--	
evm.model.HIC_ASM_8.334.1	PREDICTED: ATP-sen	ATP-sensitive inward r	K04995	potassium inw

evm.model.HIC_ASM_11.803	PREDICTED: transme	Transmembrane protei	--	
evm.model.HIC_ASM_2.2127	MICAL-like protein 2	MICAL-like protein 2	K21068	MICAL-like p
evm.model.HIC_ASM_2.44	calpain-10 isoform 2 [	Calpain-10 OS=Homo	K08579	calpain-10 [EC
evm.model.HIC_ASM_0.802_evı	PREDICTED: protein	Protein ABHD18 OS=]	--	
evm.model.HIC_ASM_5.1200.1	protein AMN1 homolo	Protein AMN1 homolo	K10268	F-box and leu
evm.model.HIC_ASM_1.734	interleukin-1 receptor a	Interleukin-1 receptor a	K04723	interleukin 1 r
evm.model.HIC_ASM_6.159.1	protein DEK isoform X	Protein DEK OS=Hom	K17046	protein DEK |
evm.model.HIC_ASM_10.1280	PREDICTED: mutS pr	MutS protein homolog	K08740	DNA mismatc
evm.model.HIC_ASM_12.1039	two pore calcium chan	Two pore calcium char	K16896	two pore calci
evm.model.HIC_ASM_0.1152	epigen [Acinonyx juba	Epigen OS=Homo sapi	K13403	methylenetetra
evm.model.HIC_ASM_10.358.1	PREDICTED: ephrin t	Ephrin type-A receptor	K05103	Eph receptor A
evm.model.HIC_ASM_4.433_evı	PREDICTED: tetratric	Tetratricopeptide repea	K21843	tetratricopepti
evm.model.HIC_ASM_20.691	secreted and transmem	Secreted and transmem	K06457	T-cell surface
evm.model.HIC_ASM_10.809	Tektin-2 [Pteropus ale	Tektin-2 OS=Bos tauru	K18629	tektin-2 | (Ref
evm.model.HIC_ASM_21.991	PREDICTED: neurope	Neuropeptides B/W rec	K08377	neuropeptides
evm.model.HIC_ASM_11.610.1	caveolin-1 [Felis catus	Caveolin-1 OS=Neofel	K06278	caveolin 1 | (R
evm.model.HIC_ASM_2.477	LOW QUALITY PRO	T-cell-specific surface	K06470	CD28 antigen
evm.model.HIC_ASM_15.416.3	ER membrane protein	ER membrane protein	--	
evm.model.HIC_ASM_5.645	PREDICTED: myogen	Myogenic factor 6 OS=	K18485	myogenic fact
evm.model.HIC_ASM_19.1086	PREDICTED: ataxin-7	Ataxin-7 OS=Homo sa	K11318	ataxin-7 | (Ref
evm.model.HIC_ASM_9.517	PREDICTED: elongat	Elongator complex pro	K11373	elongator com
evm.model.HIC_ASM_7.448	PREDICTED: coiled-c	Coiled-coil domain-coı	--	
evm.model.HIC_ASM_4.300	PREDICTED: prosper	Prospero homeobox pr	K20212	prospero home
evm.model.HIC_ASM_0.748.3	interleukin-15 [Acinor	Interleukin-15 OS=Feli	K05433	interleukin 15
evm.model.HIC_ASM_5.372.1	PREDICTED: ADP-rit	ADP-ribosylation facto	K12404	ADP-ribosylat
evm.model.HIC_ASM_11.35	NAC-alpha domain-cor	NAC-alpha domain-cor	K03626	nascent polype
evm.model.HIC_ASM_5.1124_ev	AT-rich interactive dor	AT-rich interactive dor	K11765	AT-rich intera
evm.model.HIC_ASM_1.845	hypothetical protein H	Protein disulfide-isome	K09583	protein disulfi
evm.model.HIC_ASM_10.807	PREDICTED: protein	Protein argonaute-3 OS	K11593	eukaryotic trar
evm.model.HIC_ASM_17.427.7	antithrombin-III [Eum	Antithrombin-III OS=F	K03911	antithrombin I
evm.model.HIC_ASM_6.1517	PREDICTED: mitogen	Mitogen-activated prot	K04428	mitogen-activa
evm.model.HIC_ASM_0.1000.5	SWI/SNF-related matri	SWI/SNF-related matri	K14439	SWI/SNF-rela
evm.model.HIC_ASM_16.400	PREDICTED: serpin E	Serpin E3 OS=Homo s	K23429	| (RefSeq) SEI
evm.model.HIC_ASM_6.440	advanced glycosylation	Advanced glycosylatio	K19722	receptor for ad
evm.model.HIC_ASM_11.174.1	PREDICTED: calcium	Calcium-independent p	K16815	calcium-indep
evm.model.HIC_ASM_9.492	PREDICTED: unchara	Protein shortage in chi	--	
evm.model.HIC_ASM_14.604	protein transport protei	Protein transport protei	K14005	protein transp
evm.model.HIC_ASM_16.238	PREDICTED: ubiquiti	Ubiquitin carboxyl-terr	K05609	ubiquitin carb
evm.model.HIC_ASM_1.354.1	coatomer subunit beta'	Coatomer subunit beta'	K17302	coatomer subu
evm.model.HIC_ASM_2.317	striated muscle prefere	Striated muscle prefere	K08809	striated muscl
evm.model.HIC_ASM_1.778	D-beta-hydroxybutyrat	D-beta-hydroxybutyrat	K00019	3-hydroxybuty
evm.model.HIC_ASM_20.706	leucine-rich repeat-con	Leucine-rich repeat-coı	K12175	COP9 signalos
evm.model.HIC_ASM_2.1156	ribosome biogenesis pr	Ribosome biogenesis p	K09140	pre-rRNA-pro
evm.model.HIC_ASM_9.678	PREDICTED: dnaJ ho	DnaJ homolog subfami	K09511	DnaJ homolog

evm.model.HIC_ASM_5.369	PREDICTED: pyridox	Pyridoxal phosphate pl	K07758	pyridoxal pho
evm.model.HIC_ASM_2.2072	ras-associating and dil	Ras-associating and dil	K14376	poly(A) polym
evm.model.HIC_ASM_19.321.5	E3 ubiquitin-protein lig	E3 ubiquitin-protein lig	K10657	E3 ubiquitin-p
evm.model.HIC_ASM_6.1251	PREDICTED: tumor p	Tumor protein D53 OS	--	
evm.model.HIC_ASM_5.457	PREDICTED: BPI fold	BPI fold-containing fa	K08761	phospholipid t
evm.model.HIC_ASM_2.933	G-protein coupled rece	G-protein coupled rece	K08412	G protein-cou
evm.model.HIC_ASM_6.898	protein FAM135A isof	Protein FAM135A OS	--	
evm.model.HIC_ASM_1.661	protein FAM131A isof	Protein FAM131A OS	K05010	chloride chann
evm.model.HIC_ASM_2.307	PREDICTED: anion ex	Anion exchange protei	K13856	solute carrier f
evm.model.HIC_ASM_5.1196_e	protein bicaudal D hon	Protein bicaudal D hon	K18739	protein bicaud
evm.model.HIC_ASM_5.177	gamma-parvin isoform	Gamma-parvin OS=Ho	K06275	parvin | (RefS
evm.model.HIC_ASM_2.1723	PREDICTED: coiled-c	Coiled-coil domain-co	--	
evm.model.HIC_ASM_10.1126	Hypothetical predicted	Leucine-rich repeat-co	--	
evm.model.HIC_ASM_16.526.3	ubiquitin carboxyl-tern	Ubiquitin carboxyl-ter	K11842	ubiquitin carb
evm.model.HIC_ASM_10.510	PITH domain-containi	PITH domain-containi	--	
evm.model.HIC_ASM_18.1194	PREDICTED: probabl	Probable ATP-depende	K20096	ATP-depende
evm.model.HIC_ASM_18.717	PREDICTED: zinc fin	Zinc finger protein neu	K22198	BRG1-associa
evm.model.HIC_ASM_15.176	putative uncharacterize	--	--	
evm.model.HIC_ASM_1.366	tripartite motif-contain	Tripartite motif-contai	K12018	tripartite moti
evm.model.HIC_ASM_17.829.2	g patch domain-contair	G patch domain-contai	K04849	voltage-depen
evm.model.HIC_ASM_20.270	PREDICTED: transien	Transient receptor pote	K04972	transient recep
evm.model.HIC_ASM_2.282	hypothetical protein P	WD repeat and FYVE	K23299	WD repeat an
evm.model.HIC_ASM_2.2023.1	tectonin beta-propeller	Tectonin beta-propelle	K17988	tectonin beta-
evm.model.HIC_ASM_2.1721	Phosphorylase b kinase	Phosphorylase b kinase	K00871	phosphorylase
evm.model.HIC_ASM_2.1414	PREDICTED: protein	Protein CLEC16A OS	K19513	protein CLEC
evm.model.HIC_ASM_12.1042.4	ATP-dependent RNA h	ATP-dependent RNA h	K14808	ATP-depende

GO_anno
Glycosyltransferase 2-like
Small integral membrane protein 11A
--
GO:0008237|GO:0008270|GO:0062023
GO:0005515
GO:0004198|GO:0006508
GO:0004672|GO:0005524|GO:0006468
--
GO:0006355
GO:0003824
--
--
Protein-tyrosine phosphatase, catalytic
LIPIN family
--
GO:0003777|GO:0005524|GO:0007018|GO:0008017
GO:0035091
--
UPF0708 protein C6orf162
Serine-rich coiled-coil domain-containing protein 1
GO:0016301
GO:0004553|GO:0005975
Bromodomain protein 4, C-terminal
Treslin, N-terminal
GO:0005737
--
--
--
GO:0003700|GO:0006355
GO:0005634|GO:0005737|GO:0006309|GO:0016787
GO:0005515
TRPM, SLOG domain
C1q domain
GO:0003676
GO:0016788
--
--
Coiled-coil domain-containing protein 154
GO:0005515
EGF-like domain
--
GO:0005515

GO:0005525
GO:0004725|GO:0006470
--
50S ribosomal protein uL4
GO:0005515
GO:0016702|GO:0055114
--
GO:0005337|GO:0016021|GO:1901642
Coiled-coil domain-containing protein 175
GO:0015095|GO:0015693|GO:0016021
--
GO:0006486|GO:0008373
--
--
Ankyrin repeat-containing domain
GO:0005085|GO:0007264
--
GO:0004175|GO:0006511|GO:0019773
--
GO:0016021
--
--
Polycystin cation channel, PKD1/PKD2
B9 domain
--
--
Rab-GTPase-TBC domain
GO:0016020
Uncharacterized protein CXorf65-like
--
Putative adherens-junction anchoring domain
--
GO:0005737|GO:0006914
Uncharacterised protein C3orf67
DOMON domain
Pleckstrin homology domain
GO:0035556
GO:0008418
GO:0006303
GO:0019722
GO:0004672|GO:0005524|GO:0006468
von Willebrand factor, type D domain
4Fe-4S ferredoxin-type, iron-sulphur binding domain
--

--
--
--
--
--
EGF-like domain
GO:0000976|GO:0032922
GO:0005515
GO:0016311|GO:0016791
OST-HTH/LOTUS domain
--
GO:0005509
GO:0005216|GO:0006811|GO:0016020|GO:0055085
VEGFR-2, Transmembrane domain
GO:0005737|GO:0006569|GO:0009435|GO:0030170|GO:0030429
Putative RNA methylase domain
GO:0005515
GO:0016021
Kita-kyushu lung cancer antigen 1
--
--
GO:0003677|GO:0006355
DEK, C-terminal
Methyl-CpG-binding domain protein 5/6
--
Choline/carnitine acyltransferase domain
--
Alpha carbonic anhydrase domain
GO:0003755
Oxysterol-binding protein
--
--
GO:0004672|GO:0005524|GO:0006468
--
GO:0015031
GO:0003676
ADAM, cysteine-rich domain
--
--
--
Immunoglobulin subtype 2
--
GO:0005814|GO:0007005|GO:0007268|GO:0060271
Pleckstrin homology domain

SURP and G-patch domain-containing protein 1/2
--
Immunoglobulin-like domain
--
--
GO:0003700|GO:0005634|GO:0006355|GO:0008270
GO:0005515
Uncharacterized protein C20orf194-like
--
GO:0001503|GO:0030198
GO:0016021
--
--
GO:0005515
GO:0016020
GO:0005509
GO:0005509
Laminin G domain
Interleukin-18
GO:0006814|GO:0006885|GO:0015385|GO:0016021|GO:0055085
--
--
uDENN domain
GO:0005509
--
GO:0004467|GO:0016021|GO:0031957
GO:0005515
--
--
Zinc finger, FYVE-related
Pre-PUA domain
GO:0005509
GO:0071203
ZN622/Rei1/Reh1, zinc finger C2H2-type
GO:0005515
--
Immunoglobulin subtype 2
MHC class I-like antigen recognition-like
GO:0005515
GO:0004143|GO:0007165
Intraflagellar transport protein 52 homolog
TMEM248/TMEM219
GO:0007165
CEP170, C-terminal

PA14/GLEYA domain
GO:0008289
Opalin
GO:0052689
Fanconi anaemia group A protein, N-terminal domain
--
--
GO:0016021
CP2 transcription factor
Haem peroxidase, animal-type
Protein of unknown function DUF4078
Dynein heavy chain 3, AAA+ lid domain
GPN-loop GTPase 2
GO:0006351|GO:0070176
GO:0004672|GO:0005524|GO:0006468
GO:0005515
GO:0000444|GO:0007059|GO:0051301
FANCD2 opposite strand protein
SUZ-C domain
GO:0004683|GO:0005516|GO:0006468
--
GO:0022008
Tudor domain
GO:0016491|GO:0055114
--
--
GO:0004867
GO:0005737
--
GO:0005212|GO:0007601
--
C1q domain
Extended PHD (ePHD) domain
DIX domain
GO:0046872
GRIP1-associated protein 1
--
GO:0004930|GO:0007186|GO:0016021
--
GO:0004114|GO:0007165
Glutamate [NMDA] receptor, epsilon subunit, C-terminal
--
GO:0003700|GO:0006355|GO:0043565
GO:0005089|GO:0035023

--
GO:0004672|GO:0005524|GO:0006468
Regulator of chromosome condensation, RCC1
--
GO:0006820|GO:0016021
--
GO:0042132
--
Telomere-associated protein Rif1, N-terminal
--
GO:0005886|GO:0006826|GO:0006879
GO:0003777|GO:0007018
--
GO:0016021
GO:0005743|GO:0006744|GO:0008289
GO:0004866
GO:0003700|GO:0006355|GO:0043565
GO:0003676
GO:0004725
Laminin, N-terminal
GO:0030015
--
GO:0005515
--
Leucine-rich repeat, typical subtype
--
GO:0005267|GO:0016020|GO:0071805
--
GO:0005515
GO:0004674|GO:0042981
GO:0000814|GO:0071985
GO:0008757
BspA type Leucine rich repeat region
GO:0035091
GO:0005047|GO:0005786|GO:0006614|GO:0008312|GO:0030942
AIMP2, lysyl-tRNA synthetase binding domain
Axin-1/2, tankyrase-binding domain
Flotillin family
von Willebrand factor, type A
--
SH2 domain
GO:0008168
BTB/Kelch-associated
Alpha/beta hydrolase fold-1

Carbohydrate kinase PfkB
--
MD-2-related lipid-recognition domain
GO:0004672|GO:0005524|GO:0006468
Coiled-coil domain-containing protein 48
--
GO:0035091
Sushi/SCR/CCP domain
Folliculin-interacting protein, middle domain
Voltage-dependent L-type calcium channel, IQ-associated domain
GO:0005515
--
GO:0003676
--
Acetyl-coenzyme A synthetase, N-terminal domain
--
GO:0003676
GO:0016192
Metallo-beta-lactamase
Bcl2-/adenovirus E1B 19kDa-interacting protein 2
--
--
GO:0051168
Leucine-rich repeat, typical subtype
--
Selenoprotein F/M
COMM domain-containing protein 9
Actin family
GO:0017112
--
--
von Willebrand factor, type A
GO:0005542|GO:0016740
--
GPN-loop GTPase 3
--
RH1 domain
GO:0005216|GO:0006811|GO:0016021
GO:0003924|GO:0005525
Zinc finger, RING-type
Helicase superfamily 1/2, ATP-binding domain
--
GO:0045721
--

GO:0005515
Proline racemase family
GO:0008289
Cytidine and deoxycytidylate deaminase domain
GO:0005319|GO:0006869
GO:0005523|GO:0051694
GO:0003743|GO:0006413
GO:0005515
--
GO:0005041|GO:0005044|GO:0006898|GO:0006935|GO:0008009|GO:0030307|GO:0030335|GO:0034
GO:0005515
Chromo/chromo shadow domain
Protein of unknown function with motif GDWWSH
GO:0007165|GO:0016020
Immunoglobulin-like domain
GO:0004672|GO:0006468
GO:0003700|GO:0071339
SANT domain
mRNA decay factor PAT1 domain
SH2 domain
LAR-interacting protein, Liprin
--
Mo25-like
Alanine dehydrogenase/pyridine nucleotide transhydrogenase, NAD(H)-binding domain
GO:0003700|GO:0005634|GO:0006355|GO:0008270
GO:0031110
Protein-tyrosine phosphatase, catalytic
--
--
--
GO:0045454
--
--
--
SLED domain
Adenylyl cyclase-associated protein CAP2
--
Mitochondrial substrate/solute carrier
Cysteine-rich hydrophobic domain-containing protein 1/2
GO:0006886|GO:0016192|GO:0030117
GO:0005515
GO:0005515
--
GO:0008270

Protein of unknown function DUF4735
CRAL-TRIO lipid binding domain
--
Ubiquitin specific protease domain
--
Ornithine cyclodeaminase/mu-crystallin
--
GO:0010181|GO:0055114
RUN domain
GO:0003700|GO:0005634|GO:0006355
Cytochrome c oxidase assembly protein PET191
GO:0005515
Protein of unknown function DUF4703
--
--
GO:0008138|GO:0016311
--
GO:0006914
Zinc finger protein 469
Transmembrane and coiled-coil domain-containing protein 2
--
--
--
GO:0016020|GO:0035556|GO:0042500|GO:0042987
--
Leucine-rich repeat, typical subtype
--
GO:0007186|GO:0008188|GO:0016021
--
--
--
Ras-like guanine nucleotide exchange factor, N-terminal
GO:0008017
--
GO:0000439|GO:0006289|GO:0006355
Inhibitor of nuclear factor kappa-B kinase-interacting protein
RUN domain
--
--
GO:0006506
GO:0008237|GO:0008270
GO:0019904
Cilia- and flagella-associated protein 91
--

Polycystin cation channel, PKD1/PKD2
Alpha/beta hydrolase fold-1
--
--
--
GO:0005515
--
GO:0005742
--
AAA+ ATPase domain
--
Dpy-30 motif
Phosphatidylinositol 3-/4-kinase, catalytic domain
GO:0005515
Testis-expressed sequence 37 protein
GO:0005506|GO:0005737|GO:0019310|GO:0050113|GO:0055114
GO:0005125|GO:0008083|GO:0016021
MyBP-C, tri-helix bundle domain
Short-chain dehydrogenase/reductase SDR
Tubby, C-terminal
--
GO:0007034
Rab-GTPase-TBC domain
--
--
Surfactant-associated protein 2
--
GO:0016491|GO:0055114
GO:0006488
Lymphocyte expansion molecule
GO:0005515
--
Thyroglobulin type-1
--
GO:0007217
Phosphoinositide 3-kinase, accessory (PIK) domain
--
GO:0003777|GO:0005524|GO:0007018|GO:0008017
GO:0004842
--
Regulator of chromosome condensation, RCC1
Bud22/Serum response factor-binding protein 1
GO:0016021|GO:0055085
--

--
GO:0004930|GO:0007186|GO:0016021
--
--
Cytochrome B561-related
CS domain
--
GO:0005515
--
RNA helicase, DEAD-box type, Q motif
--
GO:0004930|GO:0007186
GO:0005515
GO:0016020|GO:0030001|GO:0046873
--
DHR-2 domain
GO:0004602|GO:0006979|GO:0055114
Clusterin-associated protein-1
GO:0005515
GO:0005634|GO:0005813|GO:0060271
GO:0004571|GO:0005509|GO:0016020
--
GO:0005576|GO:0006955|GO:0008009
GO:0030976
GO:0046983
Testis-expressed basic protein 1
GO:0003899|GO:0006351
--
GO:0005216|GO:0006811|GO:0016020|GO:0038023
--
--
GO:0005515
Protein SSUH2 homologue
--
GO:0003677
--
--
GO:0043087
Protein of unknown function DUF4671
--
--
--
--
GO:0005515

GO:0005044|GO:0016020
GO:0004888|GO:0005216|GO:0016021|GO:0034220
--
--
--
Potassium channel, inwardly rectifying, transmembrane domain
GO:0005509|GO:0007156|GO:0016020
GO:0043066
GO:0004656|GO:0005783|GO:0016702|GO:0055114
GO:0016020
Cytochrome b5-like heme/steroid binding domain
GO:0005524
Integrin alpha beta-propellor
U2A'/phosphoprotein 32 family A, C-terminal
Immunoglobulin-like domain
Tropomyosin
Filamin/ABP280 repeat-like
--
GO:0005515
GO:0016021
GO:0004970|GO:0016020
--
GO:0004965|GO:0007186|GO:0016021
GO:0005789|GO:0006506|GO:0016740
HAP1, N-terminal
Choline/ethanolamine phosphotransferase
Immunoglobulin-like domain
GO:0003824
Spermatogenesis-associated protein 7
--
TMEM248/TMEM219 domain
GO:0008270
Choline transporter-like
Regulator of chromosome condensation, RCC1
GO:0005509|GO:0005576
--
--
Ankyrin repeat-containing domain
--
GO:0005634
--
Immunoglobulin-like domain
GO:0005515
--

High mobility group box domain
CBS domain
--
UMA domain
--
GO:0004930|GO:0007186|GO:0016021
--
IPT domain
--
--
GO:0003950
--
DnaJ domain
--
GO:0008083
--
--
Uncharacterized protein C11orf91-like
GO:0003676
GO:0005856
Williams-Beuren syndrome chromosomal region 28 protein homologue
--
Protein of unknown function DUF4579
GO:0006464|GO:0015031
GO:0005515
GO:0005125
Domain of unknown function DUF4757
GO:0005743|GO:0008137|GO:0042773
--
--
Reeler domain
--
GO:0004222|GO:0006508
GO:0004672|GO:0005524|GO:0006468
GO:0042981
SH2 domain
GO:0015018|GO:0016020
GO:0003676|GO:0005524
GO:0016579|GO:0036459
GO:0004222|GO:0006508
GO:0004336|GO:0006683
--
GO:0046983
GO:0005750|GO:0006122

GO:0005216|GO:0006811|GO:0016020|GO:0055085
BEN domain
eRF1 domain 3
GO:0035556
GO:0016153
GO:0005200|GO:0005882|GO:0008307|GO:0045104
GO:0006360
GO:0004621|GO:0005576
GO:0003677|GO:0005634|GO:0006355|GO:0007517
GO:0005515
GO:0005249|GO:0006813|GO:0008076
GO:0005515
TRPM, SLOG domain
--
--
GO:0004930|GO:0007186|GO:0016021
GO:0004930|GO:0007186|GO:0016021
--
--
GO:0005089|GO:0035023
GO:0005102|GO:0005576|GO:0007275|GO:0016055
GO:0003779|GO:0005737|GO:0016709|GO:0019417|GO:0030042
Domain of unknown function DUF4174
Rab-GTPase-TBC domain
--
PAS domain
--
GO:0017056
GO:0005515
GO:0005515
--
GO:0006396|GO:0008173
GO:0003723|GO:0003743|GO:0006413
GO:0000902|GO:0001843|GO:0002064|GO:0003779
SEA domain
GO:0003779|GO:0030036
--
--
GO:0006355|GO:0008284
GO:0007416|GO:0048786
--
Glycoside hydrolase 35, catalytic domain
--
Reticulon

GO:0007186|GO:0016021
--
GO:0005922|GO:0007154
GO:0030695
GO:0005576|GO:0046849
GO:0008270
--
Putative transmembrane family 234
DnaJ domain
GO:0006388|GO:0016772
Haloacid dehalogenase-like hydrolase
Helicase superfamily 1/2, ATP-binding domain
GO:0007034
--
GO:0004948|GO:0016020
Glutamate-rich protein 2
--
--
GO:0051087
GO:0048168|GO:2001222
GO:0000808|GO:0005634|GO:0006260
GO:0005922|GO:0007154
GO:0003676
FCH domain
Cytochrome b5-like heme/steroid binding domain
GO:0003700|GO:0005634|GO:0006355|GO:0008270|GO:0043565
Uncharacterised protein family, transmembrane-17
GO:0001510|GO:0008168|GO:0009452
--
WWE domain
GO:0005515
GO:0004252|GO:0006508
GO:0004252|GO:0006508
--
DUF5568 family
GO:0003677
GO:0003729
--
GO:0016491|GO:0055114
--
GO:0004672|GO:0005524|GO:0006468
Protein AATF/Bfr2
--
GO:0005515

GO:0016021
Spermatogenesis-associated serine-rich protein 1
Telomere length and silencing protein 1
C2 domain
GO:0016021|GO:0055085
GO:0005777
GO:0005802|GO:0006874|GO:0008273|GO:0070588
--
GO:0006886|GO:0008536
--
--
GO:0008270
--
--
GO:0035091
GO:0007283
EGF-like domain
Protein of unknown function DUF4636
--
--
--
GO:0046332
GO:0005515
--
GO:0004930|GO:0007186|GO:0016021
GO:0000278|GO:0003700|GO:0006355
--
Protein phosphatase 1 regulatory subunit 32
--
GO:0005515
--
--
--
--
MID domain of medPIWI
GO:0005524|GO:0016887
--
GO:0005216|GO:0006811|GO:0016020|GO:0055085
EGF-like domain
Ninjurin1
Protein of unknown function DUF4660
GO:0003697
--
GO:0070072

--
Retinoblastoma-associated protein, N-terminal
GO:0003677
--
GO:0000226|GO:0007399|GO:0007411
--
GO:0003677|GO:0008270
GO:0005509|GO:0007156|GO:0016020
Desmoplakin, SH3 domain
--
--
GO:0005515
GO:0006508|GO:0008234
Phosphatidic acid phosphatase type 2/haloperoxidase
GO:0004888|GO:0005216|GO:0016021|GO:0034220
GO:0005515
--
Type I protein exporter
GO:0005515
GO:0008270
GO:0016307|GO:0046488
GO:0016627|GO:0055114
GO:0006355
GO:0005515
GO:0005515
Domain of unknown function DUF4217
GO:0005525
--
PAS domain
GO:0005761|GO:0006915|GO:0015935
GO:0003700|GO:0006355
Ricin B, lectin domain
GO:0016020
--
--
Pleckstrin homology domain
AH receptor-interacting protein
--
EGF-like domain
GO:0045454
Protein of unknown function DUF4513
--
FAM124
GO:0003676

GO:0003677
GO:0005515
--
GO:0005515
GO:0046872
GO:0003676
GO:0005515
--
GO:0005515
GO:0005515
--
GO:0003677
HERV-H LTR-associating protein 1
GO:0003676
Cyclic nucleotide-binding domain-containing protein 2
Calponin repeat
--
GO:0000226|GO:0015630
--
--
--
Zinc finger, LIM-type
Zinc finger, RING-type
PDZ domain 6
--
GO:0004888|GO:0005216|GO:0016021|GO:0034220
--
--
C2 domain
GO:0005515
--
--
GO:0005515
Uncharacterized protein C4orf3
R3H-associated N-terminal domain
Dynamin superfamily
--
Fructosamine/Ketosamine-3-kinase
--
--
Tyrosine-protein kinase ephrin type A/B receptor-like
GO:0016180|GO:0032039
GO:0004930|GO:0007186|GO:0016021
--

Synaptonemal complex protein 2, armadillo-repeat-like domain
GO:0003676|GO:0005524|GO:0006139|GO:0008026|GO:0016818
--
--
--
GO:0005634|GO:1901031
Leptin receptor, immunoglobulin-like domain
GO:0005215|GO:0016020|GO:0055085
C2 domain
GO:0004725|GO:0006470
Collagen triple helix repeat
GO:0016055
--
--
--
CD8 alpha subunit
Sec63 domain
GO:0001527
GO:0004672|GO:0005524|GO:0006468
Zinc finger, FYVE-related
Pleckstrin homology domain
GO:0005179|GO:0005576|GO:0007275
GO:0003777|GO:0005524|GO:0007018|GO:0008017
Calcipressin-2
GO:0005509
Sterol-sensing domain
--
EGF-like domain
GO:0016021|GO:0022857|GO:0055085
--
C2 domain
--
GO:0005515
GO:0008168|GO:0032259
Zinc finger, RanBP2-type
--
GO:0016746
--
GO:0008289
Thrombospondin type-1 (TSP1) repeat
GO:0005515
KRBA1 family repeat
Pleckstrin homology domain
GO:0006486|GO:0008373

GO:0006869|GO:0030228
--
--
C2 domain
GO:0005634
Heat shock protein 70 family
GO:0004222|GO:0006508
GO:0005515
--
--
--
--
GO:0005515
--
GO:0016491|GO:0055114
--
GO:0003676
--
GO:0004518|GO:0006281
GO:0000724|GO:0016197|GO:0030119
--
Apx/Shrm Domain 2
Dynein light chain Tctex-1 like
GO:0006821
--
GO:0007268|GO:0045202
Coiled-coil domain-containing protein 142
GO:0016831|GO:0019752|GO:0030170
GO:0016021
--
Mig-6 domain
GO:0003676
GO:0036128|GO:0097228
--
--
GO:0001678|GO:0004396|GO:0005524|GO:0005536|GO:0005975
--
--
Leucine-rich repeat, typical subtype
Radial spoke 3
--
Helicase, C-terminal
--
GO:0008168

SOCS box domain
GO:0006355
Membrane attack complex component/perforin (MACPF) domain
GO:0016491|GO:0055114
GO:0003676
--
Cyclic nucleotide-binding domain
GO:0016021
Paralemmin-3
--
--
GO:0003676
Zinc finger, HIT-type
--
--
--
GO:0003824
GO:0016311|GO:0016791
GO:0006506|GO:0016021|GO:0017176
GO:0004888|GO:0005216|GO:0016021|GO:0034220
EGF-like domain
P-type ATPase, C-terminal
Transcription factor CBF/NF-Y/archaeal histone domain
GO:0004096|GO:0020037|GO:0055114
--
--
GO:0016491
--
GO:0001522|GO:0003723|GO:0009451|GO:0009982
--
Sas10/Utp3/C1D
--
Ephrin
Putative RNA methyltransferase
GO:0005515
Zinc finger, MYND-type
GO:0008270
GO:0003677|GO:0003899|GO:0006351
Helicase, C-terminal
--
GO:0046983
--
GO:0006812|GO:0016021|GO:0016048|GO:0050951
GO:0003700|GO:0005634|GO:0006355

Squalene cyclase, N-terminal
--
--
Protein of unknown function DUF4684
--
GO:0000774|GO:0051087
GO:0006360
UDP-sugar pyrophosphorylase
--
--
GO:0003700|GO:0005634|GO:0006355|GO:0008270|GO:0043565
--
Sialate O-acetylesterase domain
--
Serine/threonine-protein phosphatase 4 regulatory subunit 4
Protein of unknown function DUF4732
Sel1-like repeat
--
GO:0003723
Ankyrin repeat-containing domain
Lipoma HMGIC fusion partner-like protein
--
Glutamate-rich protein 1
--
GO:0046332
--
C-terminal RASSF family
--
GO:0004674
--
GO:0004222|GO:0006508
Gonadal family
GO:0005515
Rab-GTPase-TBC domain
Prokineticin domain
GO:0003774|GO:0005524|GO:0016459
Inclusion body clearance protein Iml2/Tetratricopeptide repeat protein 39
GO:0016021
GO:0044599
Zinc finger, LIM-type
Enhancer of polycomb-like, N-terminal
GO:0004969|GO:0006954|GO:0007186|GO:0007204|GO:0016021|GO:0043408
GO:0003676|GO:0008270
Coiled-coil domain-containing protein 68

--
GO:0006575|GO:0016212|GO:0047804|GO:0070189|GO:0070548
GO:0004930|GO:0007186|GO:0016021
GO:0006396
RPEL repeat
Zinc finger, LIM-type
GO:0005515
--
GO:0006820|GO:0008509|GO:0016021
GO:0000166|GO:0003677
Ewing's tumour-associated antigen 1
Diphthamide synthesis DPH1/DPH2
Proacrosin binding sp32
Osteoregulin
GO:0005515
GO:0005515
EGF-like domain
Ferric reductase transmembrane component-like domain
GO:0005227|GO:0030317|GO:0036128
Immunoglobulin I-set
GO:0016021|GO:0055085
GO:0005125
GO:0004623|GO:0005509|GO:0016042
GO:0006886
Maturin
GO:0005515
C2 domain
GO:0005515
GO:0005515|GO:0007165
Cyclin, N-terminal
GO:0004045
GO:0005515
GO:0005515
GO:0005524
FERM, C-terminal PH-like domain
PIH1 domain-containing protein 3
GO:0016651|GO:0048038|GO:0051287|GO:0055114
Mab-21 domain
--
Amphiphysin
GO:0001518|GO:0005248|GO:0006814
Usher syndrome type-1C protein-binding protein 1-like
GO:0015095|GO:0016409
Protein FAM219

GO:0070507
GO:0005515
--
Immunoglobulin I-set
GO:0004930|GO:0007186|GO:0016021
GO:0032040
GO:0005540|GO:0007155
--
Zinc finger, PHD-finger
GO:0005515
Drought induced 19 protein type, zinc-binding domain
--
GO:0004672|GO:0005524|GO:0006468
Izumo sperm-egg fusion protein
--
Metallo-beta-lactamase
--
--
GO:0006629|GO:0006869|GO:0008047|GO:0042627
GO:0005515
F-BAR domain
--
--
GO:0006396|GO:0008452
TB2/DP1/HVA22-related protein
Sel1-like repeat
GO:0005096|GO:0051056
Domain of unknown function DUF1605
GO:0000139
Protein of unknown function DUF4590
SNARE-complex protein Syntaxin-18, N-terminal
EGF-like domain
GO:0004725|GO:0006470
--
GO:0046983
Transmembrane protein 247
--
Protein of unknown function DUF4655
GO:0061588
GO:0004553|GO:0005975
Protein of unknown function DUF4558
GO:0005515
Sortilin, C-terminal
--

--
--
--
--
GREB1
GO:0003676
--
--
DAN
GO:0005515
GO:0016787
GO:0005375|GO:0016021|GO:0035434
--
--
GO:0004999|GO:0007186|GO:0016020
Myozenin
Rab-GTPase-TBC domain
--
GO:0005515
GO:0005515
GO:0004177|GO:0006508|GO:0008270
GO:0005086|GO:0032012
OTU domain
--
LDLR class B repeat
--
GO:0005801|GO:0006886|GO:0016020
--
GO:0016491|GO:0055114
Extended PHD (ePHD) domain
--
--
--
ODR-4-like
--
Ciliogenesis and planar polarity effector 1
Zona pellucida domain
Antibiotic biosynthesis monooxygenase domain
--
Cyclin, C-terminal domain 2
USP8 dimerisation domain
--
--
--

--
--
--
GO:0043589
GO:0000813|GO:0032509
--
GO:0016757
GO:0004930|GO:0007186|GO:0016021
--
--
Immunoglobulin V-set domain
--
Zinc finger, LIM-type
--
DnaJ domain
GO:0005525
GO:0003676
--
GO:0035556
GO:0044237
--
--
Uroplakin-3b
--
GO:0005634|GO:0006355
Zinc finger, LIM-type
--
Small nuclear RNA activating complex (SNAPc), subunit SNAP43
GO:0003700|GO:0006357
GO:0005198
Yos1-like
--
--
GO:0005515
ZU5 domain
DNA ligase 3, BRCT domain
GO:0003899|GO:0006351
Histone chaperone domain CHZ
--
DDB1- and CUL4-associated factor 15, WD40 repeat-containing domain
--
GO:0005515
GO:0031110
GO:0044212

GO:0003824
Cysteine and tyrosine-rich protein 1
Immunoglobulin V-set domain
GO:0004674|GO:0005524|GO:0006468
--
Pleckstrin homology domain
von Willebrand factor, type A
--
GO:0005509|GO:0007156|GO:0016020
--
GO:0005515
CD34/Podocalyxin
GO:0005525|GO:0007165|GO:0016020
--
--
GO:0003924|GO:0005525
--
Tumour protein p53-inducible nuclear protein
Odontogenic ameloblast-associated protein
Short-chain dehydrogenase/reductase SDR
Sterol-sensing domain
C2 domain
GO:0005247|GO:0006821|GO:0016020|GO:0055085
--
TMEM85/ER membrane protein complex subunit 4
GO:0004418|GO:0033014
GO:0015031|GO:0016021
GO:0005184
--
--
GO:0005515
Collagen triple helix repeat
GO:0005737|GO:0045765
GO:0003723|GO:0003726
FAM194, C-terminal
--
JMY/WHAMM, middle domain
CCDC93, coiled-coil domain
GO:0005133|GO:0005576|GO:0006955
DNA mismatch repair protein Mlh1, C-terminal
SH2 domain
HARP domain
--
GO:0006886|GO:0006888|GO:0030127

Telomere repeats-binding bouquet formation protein 2
GO:0005515
GO:0005509
--
GO:0005245|GO:0005891|GO:0070588
Band 7 domain
Condensin complex subunit 1, N-terminal
GO:0005085|GO:0007264
cDENN domain
--
Immunoglobulin V-set domain
Zinc finger, LIM-type
Pentatricopeptide repeat
High mobility group box domain
GO:0005515
Sushi/SCR/CCP domain
GO:0003824
GO:0000413|GO:0003755
Uncharacterised domain KLRAQ/TTKRSYEDQ, C-terminal
Major intrinsically disordered Notch2-binding receptor 1-like, C-terminal
TEP1, N-terminal
GO:0005515
Nucleolar complex-associated protein 3
--
Staphylococcal nuclease (SNase-like), OB-fold
GO:0005515
Coiled-coil domain-containing protein 190
GO:0005856
--
--
GO:0005515
--
Domain of unknown function DUF4614
--
ELMO domain
GO:0016020
GO:0055114
GO:0003677|GO:0005634
--
GO:0003774|GO:0016459
--
GO:0005634|GO:0005737
GO:0003677|GO:0003899|GO:0006351
--

--
GO:0016307|GO:0046488
GO:0006614|GO:0008312|GO:0048500
--
GO:0005515
GO:0000166|GO:0004812|GO:0005524|GO:0006418
Uncharacterised protein family UPF0489
--
--
GO:0006355|GO:0043565
--
GO:0005515
MORN motif
--
GO:0006355|GO:0007165
--
--
GO:0003676
Janus kinase and microtubule-interacting protein, C-terminal domain
Pleckstrin homology domain
PIN domain
--
GO:0003676
GO:0035556
CP2 transcription factor
--
CUB domain
Leucine-rich repeat N-terminal domain
GO:0007165
--
Laminin G domain
Protein of unknown function DUF4501
Protein FAM169
GO:0000978|GO:0001228|GO:0001837
Dynein heavy chain, domain-2
--
GO:0008270
--
NACHT nucleoside triphosphatase
Hydrocephalus-inducing-like
GO:0004930|GO:0016020
PWWP domain
UPF0462 protein C4orf33
GO:0006511|GO:0031625

ENTH domain
--
--
--
Lipocalin/cytosolic fatty-acid binding domain
--
GO:0005515
Coiled-coil domain-containing protein 13
EF-hand domain, type 2
GO:0005102|GO:0005577|GO:0030168|GO:0051258
GO:0005230|GO:0006811|GO:0016021
--
Metallo-beta-lactamase
--
GO:0004672|GO:0005524|GO:0006468
--
Uncharacterised protein family UPF0564
Spermatid-specific manchette-related protein 1
--
GO:0004386
GO:0035091
--
Domain of unknown function DUF4708
--
C2 domain-containing protein 2-like
--
GO:0006486|GO:0008378|GO:0016020
GO:0003676
Protein of unknown function DUF4551
Transcription factor AP-2, C-terminal
GO:0007155|GO:0007417
von Willebrand factor, type A
--
--
GO:0004812|GO:0005524|GO:0006418
C2 domain
--
--
Connexin, N-terminal
--
GO:0000281|GO:0005634|GO:0005737|GO:0006974|GO:0007026|GO:0007050|GO:0008013|GO:0008
--
--
JmjC domain

Coiled-coil domain-containing protein 22
GO:0004888|GO:0032609|GO:0050863|GO:2000404
GO:0005515
GO:0005515
--
--
SAP domain
--
--
--
GO:0016021
--
--
--
--
GO:0004518|GO:0006281
Meiotic recombination, Spo11
--
G2 and S phase-expressed protein 1, N-terminal
GO:0005515
--
GO:0004672|GO:0005524|GO:0006468
Apolipoprotein M
GO:0016772
--
Ribosomal protein L7Ae/L30e/S12e/Gadd45
GO:0006470|GO:0008138
--
--
GO:0042981
Zinc finger, RING-type
--
GO:0015026|GO:0030509|GO:0036122|GO:0055072|GO:1990712
GO:0005216|GO:0006811|GO:0016020|GO:0055085
Methyltransferase-like
GO:0006508|GO:0008236
GO:0023052
--
GO:0008270
GO:0005096
GO:0003676
--
GO:0005856
--

EGF-like domain
GO:0005509
Transforming growth factor-beta-related
--
MyoD family inhibitor/MyoD family inhibitor domain-containing protein
--
GO:0005634|GO:0006355
Protein of unknown function DUF4545
GO:0003824
--
--
PSI domain
GO:0006298|GO:0016887|GO:0032300
GO:0005515
--
--
GO:0003700|GO:0006355
--
Immunoglobulin I-set
GO:0005085|GO:0007264
JmjC domain
Zinc finger, LIM-type
Methylated in normal thymocytes protein
--
GO:0005201|GO:0005581
--
--
SKI/SNO/DAC domain
GO:0005515
Immunoglobulin-like domain
C2 domain
--
--
Nuclear hormone receptor, ligand-binding domain
GO:0004930|GO:0007186|GO:0016021
GO:0016021
--
S1-like RNA binding domain
GO:0005515
GO:0004672|GO:0005524|GO:0006468
GO:0005515
GO:0005515
GO:0005634|GO:0006355
Ricin B, lectin domain

Immunoglobulin subtype
Immunoglobulin-like domain
GO:0005515
--
--
GO:0006744|GO:0055114
GO:0004672|GO:0005524|GO:0006468
GO:0000413|GO:0003755
--
Sodium/potassium/calcium exchanger
CARMIL, C-terminal domain
RPEL repeat
GO:0003700|GO:0006355|GO:0043565
GO:0004402|GO:0006355|GO:0016573
Ras GTPase-activating protein
--
GO:0004725|GO:0006470
GO:0004867
--
BRCT domain
--
--
GO:0046912
--
GO:0003676
GO:0005515
Uncharacterised protein family UPF0454
Galactosyltransferase, N-terminal
GO:0004930|GO:0007186|GO:0016021
--
GO:0005515
--
GO:0006364
--
Proteasome assembly chaperone 2, eukaryotic
GO:0005515
Interferon/interleukin receptor domain
Learning associated protein 18-like
GO:0003700|GO:0005667|GO:0051726
GO:0003700|GO:0005634|GO:0006355|GO:0008270|GO:0043565
GO:0005515
GO:0022857|GO:0055085
--
--

--
Thrombospondin type-1 (TSP1) repeat
GO:0000139
CSPG repeat
IRS-type PTB domain
GO:0005515
GO:0003677|GO:0005634
--
GO:0006813|GO:0015269|GO:0016020
GO:0005509
COS domain
GO:0005739|GO:0033617
SH2 domain
--
--
C-type lectin-like
GO:0005515
Pleckstrin homology domain
RGS domain
GO:0005515
GO:0003779
Protein-tyrosine phosphatase, catalytic
Bestrophin
GO:0005813|GO:0007052|GO:0031122|GO:0097546|GO:1905515
Alpha/beta hydrolase fold-1
GO:0003700|GO:0006355
--
--
GO:0008270|GO:0061630
Ankyrin repeat-containing domain
--
--
GO:0005249|GO:0006813|GO:0008076
GO:0003676
GO:0006355
Pleckstrin homology domain
Centrosomal protein Cep63/Deup1, N-terminal
Nucleoside 2-deoxyribosyltransferase
Exoribonuclease, phosphorolytic domain 1
Protein of unknown function DUF4618
GO:0005089|GO:0035023
GO:0000184
Transmembrane protein family 132, middle domain
--

GO:0005515
GO:0004672|GO:0005524|GO:0006468
Immunoglobulin I-set
GO:0005515
--
--
GO:0005576|GO:0007585
GO:0005525
GO:0005515
--
GO:0005515
GO:0005509
--
--
GO:0000145|GO:0006887
--
Strawberry notch, AAA domain
GO:0004930|GO:0007186|GO:0016021
GO:0004970|GO:0016020
--
Alanine racemase, N-terminal
RNA polymerase I specific transcription initiation factor RRN3
--
--
--
GO:0035082|GO:0060294
GO:0005515
GO:0005509
--
GO:0005515
GO:0043087
CABIT domain
Immunoglobulin subtype
--
--
--
GO:0005615
GO:0044877
L27 domain, C-terminal
GO:0005515
GO:0003779
Protein BEAN1
Translin-associated factor X-interacting protein 1, N-terminal
Nucleoporin Nup120/160

GO:0006974|GO:0008283
GO:0003676
GO:0007605
--
--
RGS domain
GO:0006397
GO:0003824
GO:0003824
--
GO:0005737|GO:0042325
GO:0003676
GO:0046872
--
GO:0005634|GO:0045892
GO:0003824
GO:0004672|GO:0005524|GO:0006468
--
GO:0005515
GO:0005515
GO:0003723|GO:0005515
Transmembrane protein family 132, middle domain
--
GO:0004888|GO:0007166|GO:0016021
GO:0005086|GO:0032012
--
Domain of unknown function DUF4550
--
GO:0016868|GO:0071704
--
GO:0006890
--
--
--
Immunoglobulin subtype
Rab-GTPase-TBC domain
--
--
GO:0004843|GO:0006511
Helicase, C-terminal
GO:0015095|GO:0016409
SCA7 domain
GO:0016020
GO:0005515

GO:0005515
GO:0005515
--
GO:0004672|GO:0005524|GO:0006468
--
--
GO:0046983
CHAT domain
--
GO:0005524|GO:0016021|GO:0042626|GO:0055085
Ubiquitin interacting motif
--
GO:0002250|GO:0016021|GO:0038023
--
Yippee family
GO:0016020|GO:0022857|GO:0055085
GO:0004674|GO:0005524|GO:0006468
--
--
Double-stranded RNA-binding domain
CRIB domain
GO:0016021|GO:0055085
Netrin module, non-TIMP type
G-protein beta WD-40 repeat
GO:0008053
GO:0003334
Tumour necrosis factor receptor 19
GO:0005634|GO:0043565
--
--
--
Uncharacterized protein C17orf80
GO:0004672|GO:0005524|GO:0006468
--
--
GO:0000164|GO:0004722
GO:0003677|GO:0005730|GO:0006355|GO:0008134
--
--
--
Coiled-coil domain-containing protein 191
--
GO:0007218
GO:0004930|GO:0007186|GO:0016021

--
GO:0005515|GO:0007165
GO:0000012|GO:0003684|GO:0005634
Intermediate filament, rod domain
GO:0006886|GO:0008536
--
GO:0046983
--
--
GO:0004252|GO:0006508
GO:0005783|GO:0018400
GO:0016020
GO:0004930|GO:0007186|GO:0007596|GO:0016021
Methyltransferase domain
Pleckstrin homology domain
--
GO:0003700|GO:0006355
Tim44-like domain
--
GO:0015267|GO:0016020|GO:0055085
GO:0005515
GO:0000808|GO:0005634|GO:0006260
GO:0016021
--
--
GO:0008488|GO:0017187
GO:0005515
GO:0003676
GO:0004198|GO:0006508
--
GO:0005515
--
Phosphoinositide 3-kinase, accessory (PIK) domain
MIT
--
--
Interleukin-12 beta
SH2 domain
Immunoglobulin subtype 2
GO:0016021
--
GO:0009235
Zinc finger, RING-type
GO:0009190|GO:0016849|GO:0035556

--
Protein PRRC2
GO:0005634|GO:0043565
GO:0004672|GO:0005524|GO:0006468
GO:0003712|GO:0006357|GO:0016592
Complex 1 LYR protein
Six6 opposite strand transcript 1
GO:0003677|GO:0006352
--
--
GO:0005515
GO:0005634|GO:0051726
Transmembrane protein 239
von Willebrand factor, type A
--
GO:0005515
--
GO:0003700|GO:0005667|GO:0006355
GO:0003777|GO:0005524|GO:0007018|GO:0008017
Protein of unknown function DUF4534
GO:0000077|GO:0030896
GO:0005242|GO:0006813|GO:0016021
--
--
GO:0005515
--
GRIM-19
GO:0004518
GO:0005515
--
--
--
GO:0006886
--
GO:0005515
GO:0004672|GO:0005524|GO:0006468
GO:0005634
GO:0008285
GO:0005515
GO:0016787
Oxysterol-binding protein
--
GO:0003700|GO:0005634|GO:0006355
GO:0003676

--
MYCBP-associated protein
--
EF-hand domain, type 2
--
GO:0016311|GO:0016791
--
GO:0003723|GO:0004540
--
GO:0003676|GO:0008270
GO:0005794|GO:0007165
GO:0015204|GO:0016021|GO:0071918
Outer dense fibre protein 2-related
GO:0005576|GO:0030414
GO:0005515
Modulator of smoothened protein
GO:0004890|GO:0006811|GO:0016020
Tubby, N-terminal
GO:0016055
GO:0005515
P-type trefoil domain
FAM69, protein-kinase domain
Superoxide dismutase (Cu/Zn) / superoxide dismutase copper chaperone
GO:0001819|GO:0008284|GO:0010884
GO:0004198|GO:0006508
SH2 domain
GO:0015031|GO:0030176
--
GO:0004930|GO:0016020
Myotubularin-related 12-like C-terminal domain
--
Enamelin
Optic atrophy 3-like
Caldesmon/lymphocyte specific protein
ADAMTS/ADAMTS-like
--
GO:0005452|GO:0006820|GO:0016020
--
--
GO:0051260
--
--
DDRGK domain containing protein
GO:0021987

GO:0003700|GO:0005667|GO:0006355
Protein INCA1
PSP, proline-rich
GO:0007249
C-type lectin-like
Cilia- and flagella-associated protein 45
GO:0016021
GO:0016020
GO:0003700|GO:0006355
GO:0004672|GO:0005524|GO:0006468
UPF0692 protein
--
Dynein heavy chain 3, AAA+ lid domain
--
--
--
GO:0008444|GO:0032049
--
GO:0005634|GO:0006355|GO:0043189
--
--
--
GO:0005262|GO:0016021|GO:0070588
--
Protein DPCD
GO:0005509|GO:0005783|GO:0006457|GO:0051082
Spermatogenesis-associated protein 3
GO:0005515
Membrane-spanning 4-domains subfamily A
GO:0003777|GO:0005524|GO:0007018|GO:0008017
GO:0005515
GO:0005242|GO:0006813|GO:0016020
GO:0008083
GO:0006281|GO:0016035|GO:0019985
--
--
GO:0055114
Thyroglobulin type-1
--
Desmoplakin, SH3 domain
GO:0004252|GO:0006508
GO:0015078|GO:0015991|GO:0033179
GO:0005634|GO:0018024
--

GO:0005524|GO:0016021|GO:0042626|GO:0055085
--
GO:0006397
GO:0005245|GO:0005891|GO:0070588
--
GO:0003676|GO:0004813|GO:0005524|GO:0006419
Type I cytokine receptor, cytokine-binding domain
GO:0004672|GO:0005524|GO:0006468
GO:0000123|GO:0016573
Zinc finger, PHD-finger
Metalloprotease TIKI1/2-like
--
--
Helicase superfamily 1/2, ATP-binding domain
--
--
GO:0006935|GO:0006955|GO:0007186|GO:0016021|GO:0016493
GTF2I-like repeat
Centrosomal protein Cep63/Deup1, N-terminal
--
GO:0003714|GO:0006351
GO:0005506|GO:0016705|GO:0020037|GO:0055114
--
--
--
GO:0046856
GO:0008272|GO:0015116|GO:0016021
GO:0018024
Casein, beta
--
--
--
--
FERM, C-terminal PH-like domain
Coiled-coil domain-containing protein 17
Target of Myb1-like 2
--
--
Host cell factor C1 regulator 1
Cwf19-like protein
--
--
LDLR class B repeat
Glutamate [NMDA] receptor, epsilon subunit, C-terminal

GO:0003676
GO:0016020
--
GO:0006605|GO:0007165
LPS-induced tumour necrosis factor alpha factor
GO:0016021
--
--
GO:0008168
GO:0005523|GO:0006936|GO:0051694
Pleckstrin homology domain
GO:0004985|GO:0007186|GO:0016021
GO:0004838|GO:0009074|GO:0030170
Cullin homology domain
--
GO:0004674
GO:0003676
GO:0005515
GO:0003676
--
GO:0005883|GO:0007399
GO:0005515
--
--
--
Wolframin family
UPF0183/Protein broad-minded
GO:0004888|GO:0005216|GO:0016021|GO:0034220
--
GO:0005509
GO:0005515
--
tRNA-splicing endonuclease, subunit Sen54
--
--
--
Coiled-coil domain-containing protein 138
C2 domain
Zn-dependent metallo-hydrolase, RNA specificity domain
GO:0003735|GO:0006412|GO:0015934
GO:0045600
GH15-like domain
--
Inward rectifier potassium channel, C-terminal

TMEM213 family
--
Peptidase C2, calpain, large subunit, domain III
Abhydrolase domain containing 18
--
--
--
GO:0005524|GO:0006298|GO:0030983
GO:0005245
--
--
--
GO:0005125|GO:0006955
Tektin
GO:0004930|GO:0007186|GO:0016021
GO:0070836
GO:0006955|GO:0016020
GO:0005515
GO:0046983
SCA7 domain
GO:0002098|GO:0033588
GO:0120103
--
GO:0005126|GO:0005576|GO:0006955
GO:0006886
Nascent polypeptide-associated complex NAC domain
GO:0003677
Longin domain
GO:0005515
GO:0004867|GO:0008201|GO:2000266
--
Helicase, C-terminal
GO:0005615
Immunoglobulin-like domain
--
GO:0000712|GO:0000794|GO:0016887
--
GO:0004843|GO:0006511
GO:0005515
--
Short-chain dehydrogenase/reductase SDR
--
RNase L inhibitor RLI, possible metal-binding domain
DnaJ domain

--
Dilute domain
GO:0008270
--
GO:0008289
--
--
Protein FAM131
GO:0005452|GO:0006820|GO:0016020
GO:0008093|GO:0070840
GO:0003779|GO:0007155|GO:0031532
Flagellar C1a complex subunit C1a-32
GO:0005515
--
PITH domain
Helicase superfamily 1/2, ATP-binding domain
GO:0003676
--
GO:0008270
GO:0003676
GO:0005216|GO:0006811|GO:0016020|GO:0055085
Zinc finger, FYVE-related
Beta-propeller repeat TECPR
GO:0004672|GO:0005524|GO:0006468
CLEC16A/TT9
GO:0003676|GO:0005524

097|GO:0034341|GO:0034612|GO:0048247

285|GO:0016477|GO:0030178|GO:0043065

Supplementary Table S10 Gene Ontology enrichment analysis of genes under positive selection in maske

Category	GO ID	GO term description	Annotated	Significant	P-value
Cellular component	GO:0043228	non-membrane-bounded organelle	433:19	2.1:1.0	0.001
	GO:1990904	ribonucleoprotein complex	233:5	1.1:0.3	<0.001
Molecular Function	GO:0005515	protein binding	1721:194	8.4:9.9	0.021
	GO:0022857	transmembrane transporter activity	361:48	1.8:2.4	0.028
	GO:0005198	structural molecule activity	255:6	1.2:0.3	<0.001
Biological Process	GO:0051179	localization	715:86	3.5:4.4	0.038
	GO:0032501	multicellular organismal process	182:29	0.9:1.5	0.009

ed palm civet.

Supplementary Table S11 Known human viral receptors genes under positive selection [illegible]			
Gene ID	Receptor	Viruses	GO description
evm.model.HIC_ASM_17.199.13	CD55	Cardiovirulent coxsackieviruses	Sushi/SCR/CCP
evm.model.HIC_ASM_10.460	HSPG2		Laminin G doma
evm.model.HIC_ASM_0.1285	PDGFRA	human cytomegalovirus HCMV	6/2/2020
evm.model.HIC_ASM_0.1129	SCARB2		6/2/2020

in P. larvata.

KEGG description	Swisspront description
K04006 decay accelerating factor | (RefSeq	Complement decay-accelerating factor OS=
K06255 basement membrane-specific hepa	Basement membrane-specific heparan sulfa
K04363 platelet-derived growth factor rece	Platelet-derived growth factor receptor alph
K12384 lysosome membrane protein 2 | (R	Lysosome membrane protein 2 OS=Homo s

NR description
complement decay-accelerating factor isoform X2 [Acinonyx jubatus]
basement membrane-specific heparan sulfate proteoglycan core protein isoform X13 [Acinonyx jubatus]
platelet-derived growth factor receptor alpha [Acinonyx jubatus]
lysosome membrane protein 2 [Acinonyx jubatus]

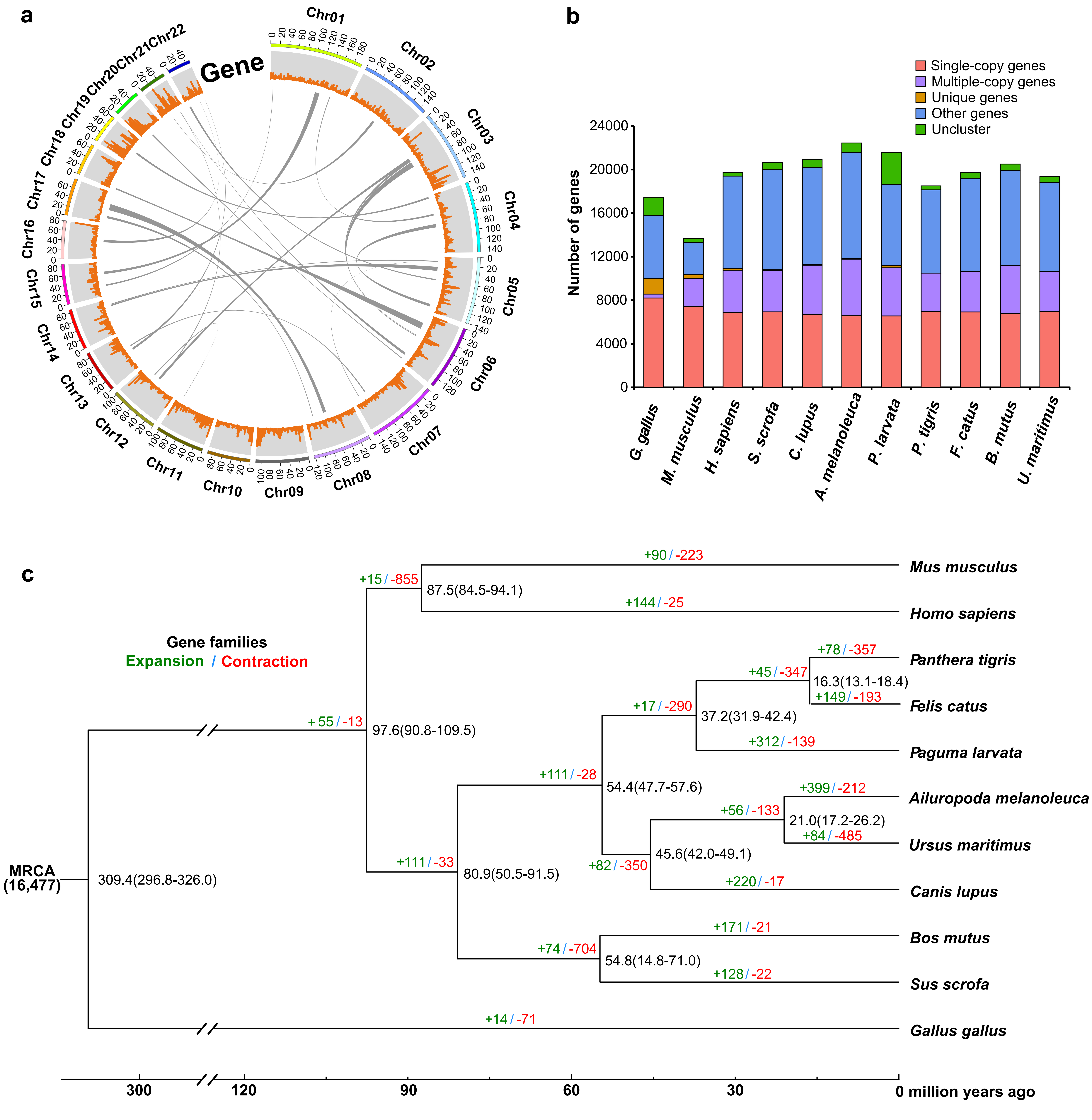
a
Gene
Chr01
Chr02
Chr03
Chr04
Chr05
Chr06
Chr07
Chr08
Chr09
Chr10
Chr11
Chr12
Chr13
Chr14
Chr15
Chr16
Chr17
Chr18
Chr19
Chr20
Chr21
Chr22
b
Single-copy genes
Multiple-copy genes
Unique genes
Other genes
Uncluster
Number of genes
0
4000
8000
12000
16000
20000
24000
G. gallus
M. musculus
H. sapiens
S. scrofa
C. lupus
A. melanoleuca
P. larvata
P. tigris
F. catus
B. mutus
U. maritimus
c
Gene families
Expansion / Contraction
MRCA (16,477)
309.4(296.8-326.0)
+55 / -13
97.6(90.8-109.5)
+15 / -855
87.5(84.5-94.1)
+90 / -223
Mus musculus
+144 / -25
Homo sapiens
+78 / -357
Panthera tigris
+45 / -347
16.3(13.1-18.4)
+149 / -193
Felis catus
+17 / -290
37.2(31.9-42.4)
+312 / -139
Paguma larvata
+111 / -28
54.4(47.7-57.6)
+399 / -212
Ailuropoda melanoleuca
+56 / -133
21.0(17.2-26.2)
+84 / -485
Ursus maritimus
+82 / -350
45.6(42.0-49.1)
+220 / -17
Canis lupus
+111 / -33
80.9(50.5-91.5)
+171 / -21
Bos mutus
+74 / -704
54.8(14.8-71.0)
+128 / -22
Sus scrofa
+14 / -71
Gallus gallus
300
120
90
60
30
0 million years ago

Figure 2
Click here to access/download;Figure;Figure 2-aa_trees.pdf

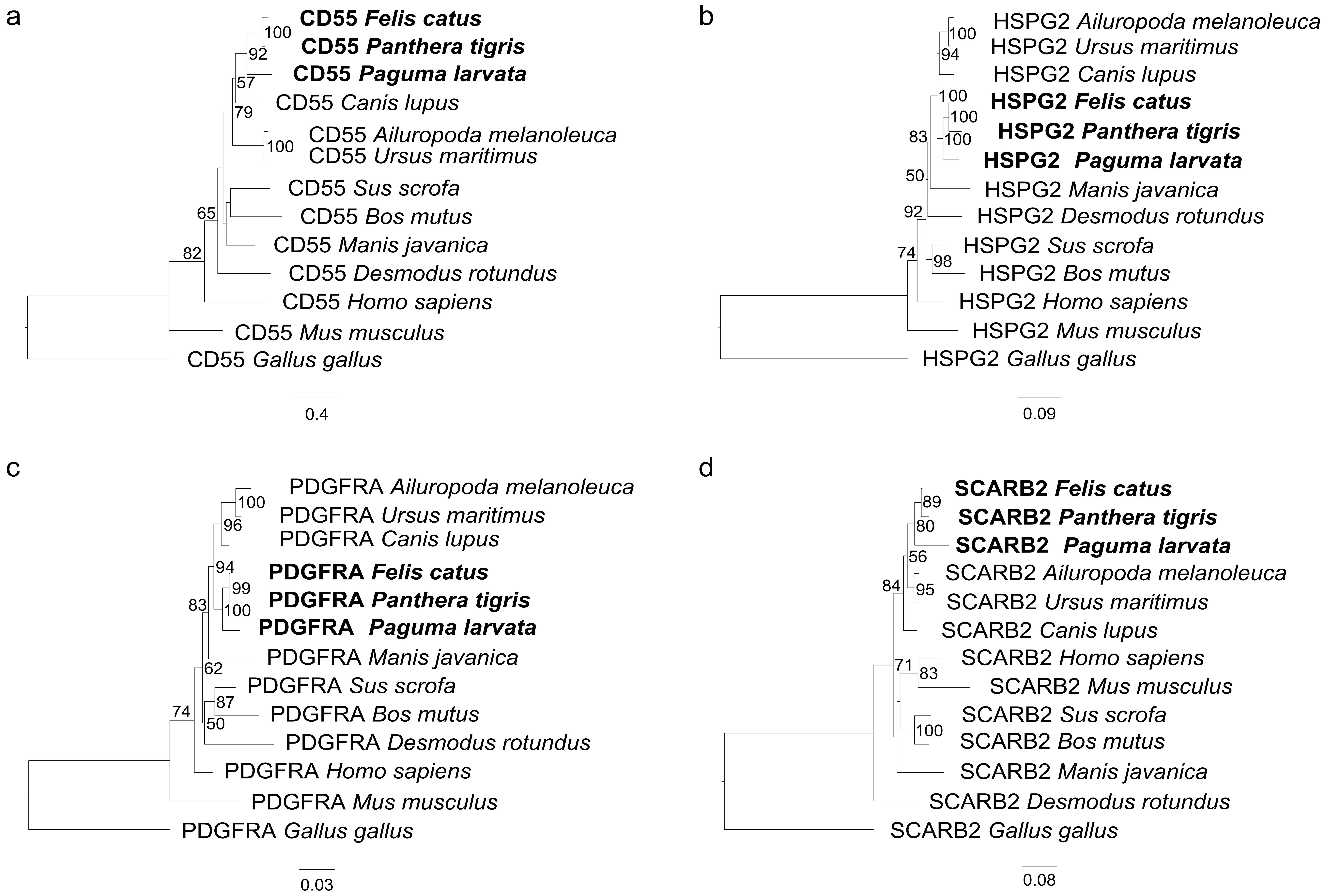

Click here to access/download;Figure;Supplementary Figures-0922.docx

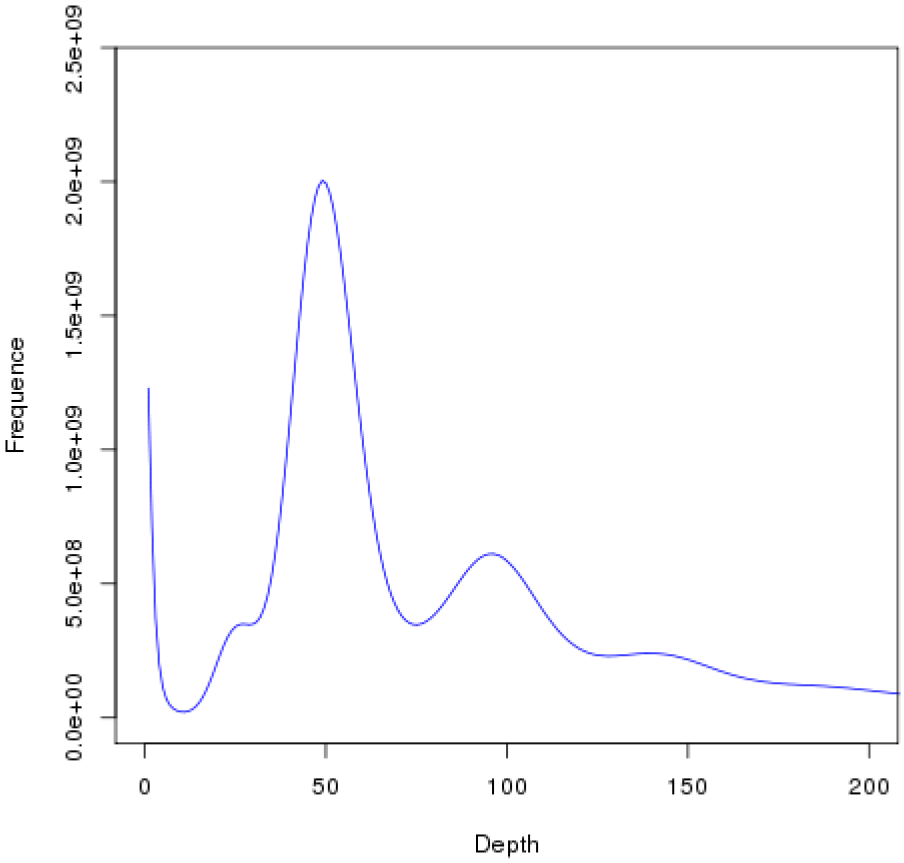

Supplementary Figure S1 K-mer frequency distribution curve (k =17) of Illumina short reads of the masked palm civet genome.

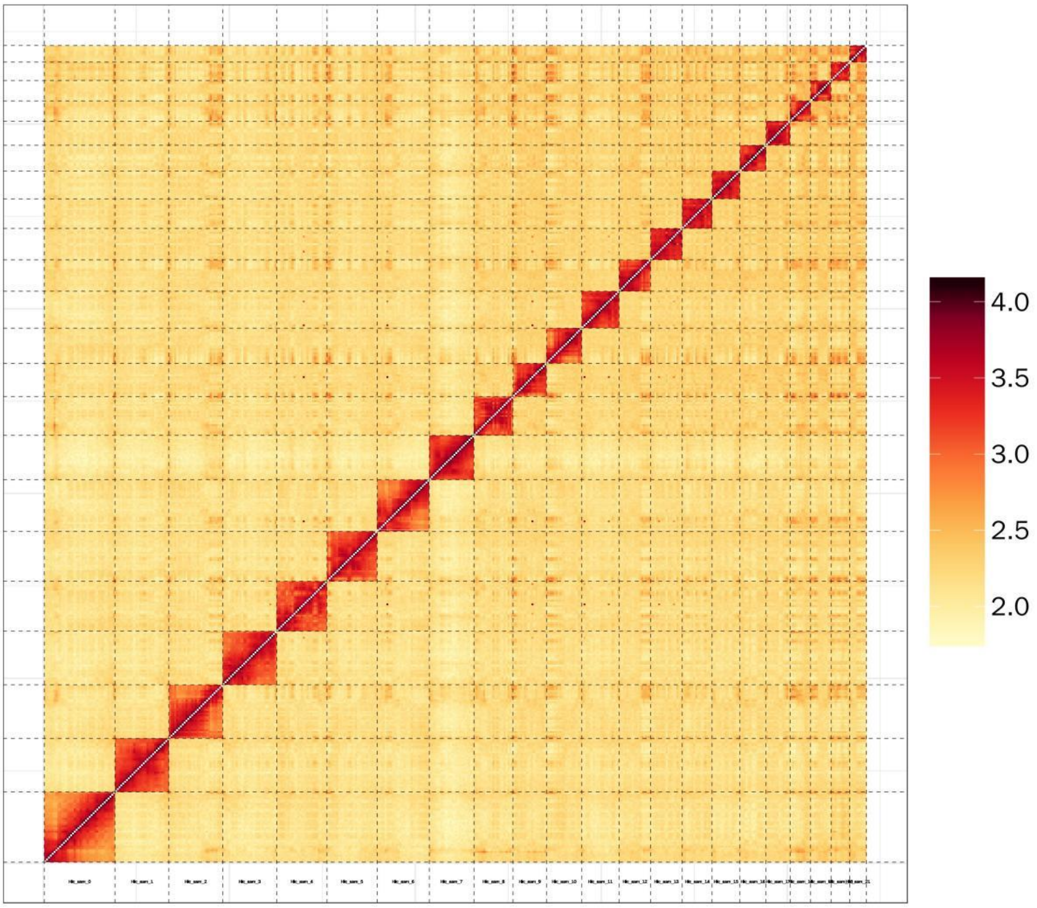

Supplementary Figure S2 Hi-C contact data mapped to the genome of masked palm civet. The heat map represents the normalized contact matrix. The strongest and weakest contact are shown in red and yellow, respectively.

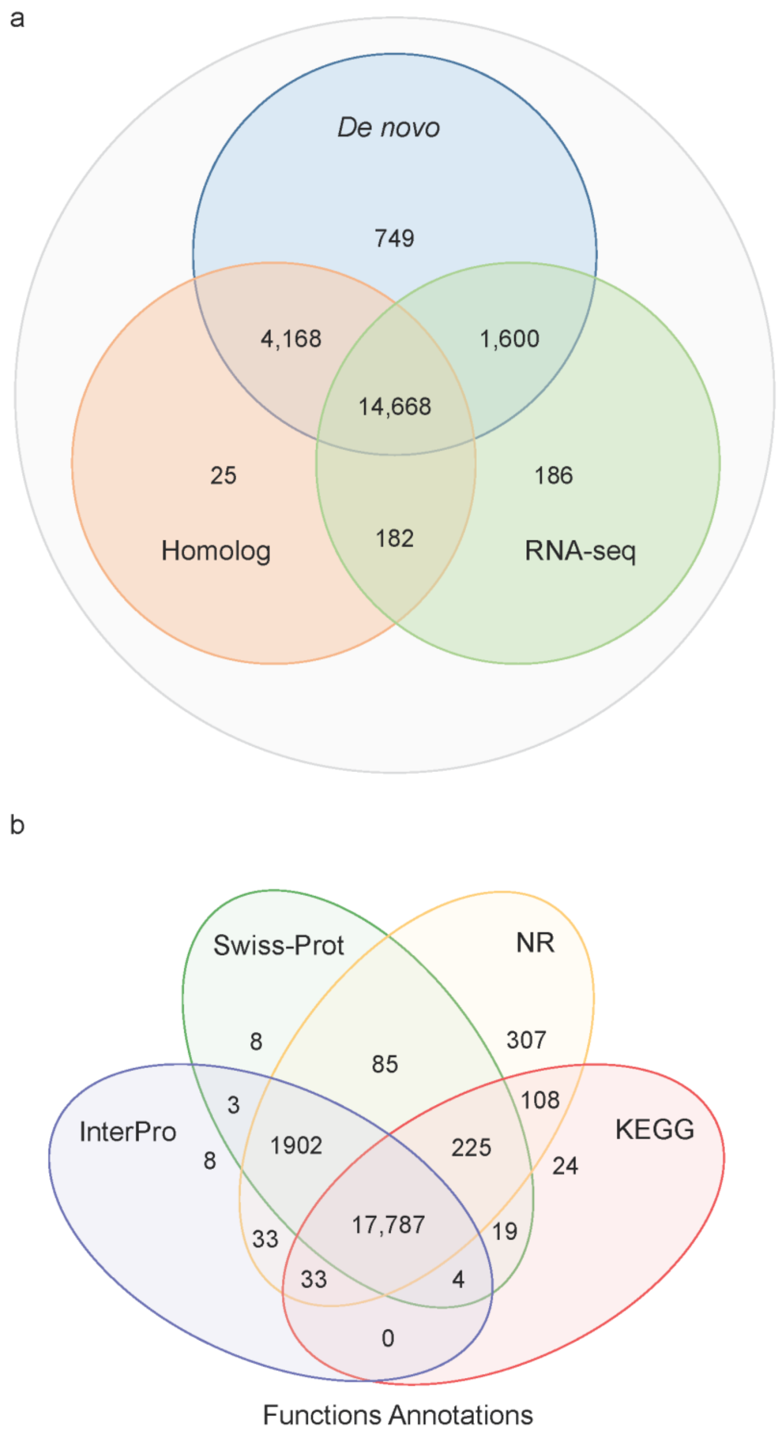

Supplementary Figure S3 Prediction and annotation of genes in masked palm civet genome. (a) Number of genes predicted with *de novo*, homolog, and RNA-seq. All predicted genes were integrated by EVM. (b) Number of genes annotated with databases of Swissprot, NR, GO, KEGG, Pfam and InterPro.

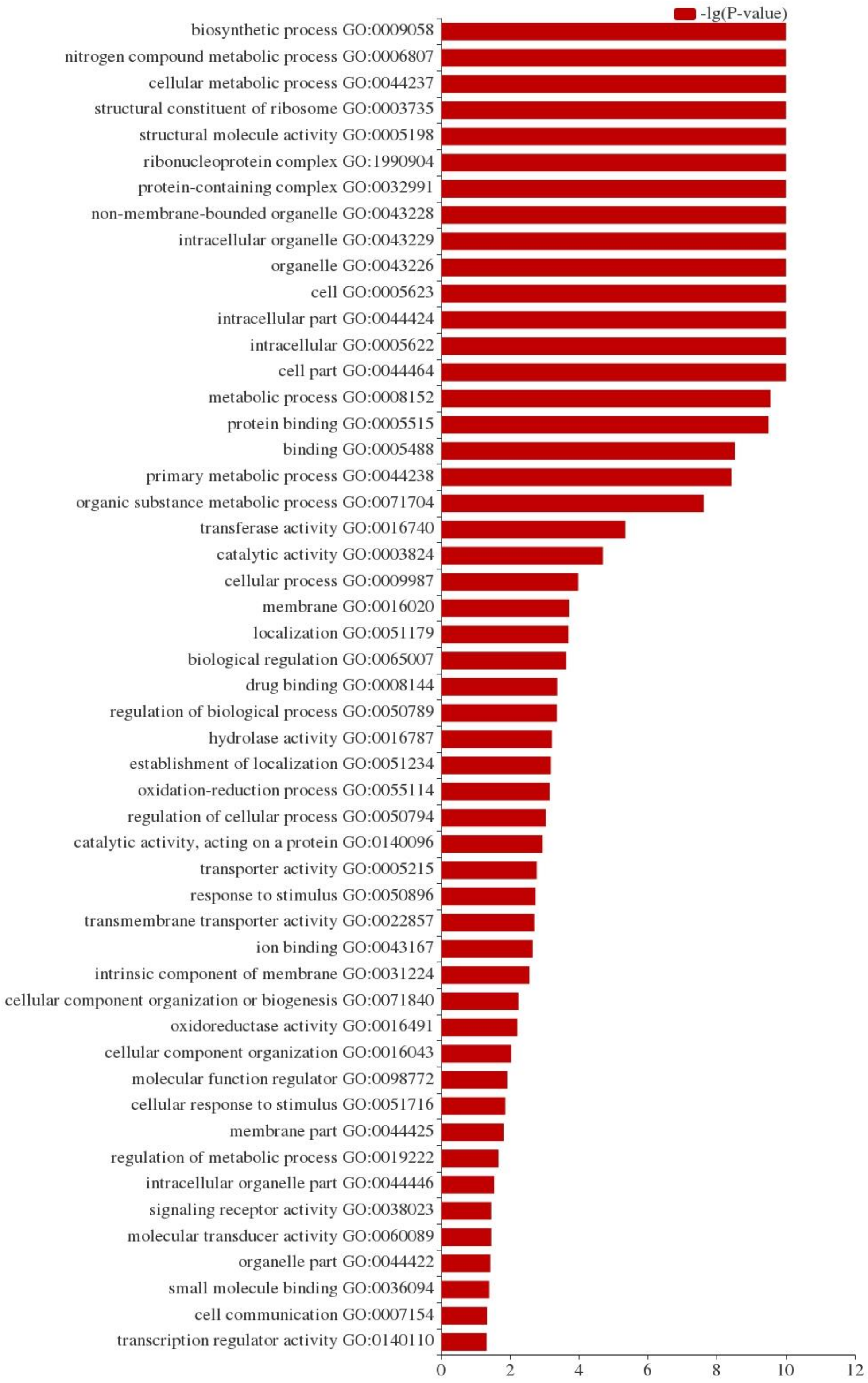

Supplementary Figure S4 Gene ontology enrichment of genes from expanded genes in masked palm civet. Red bars showed top enriched GO terms with the significant of *p*-value < 0.05.

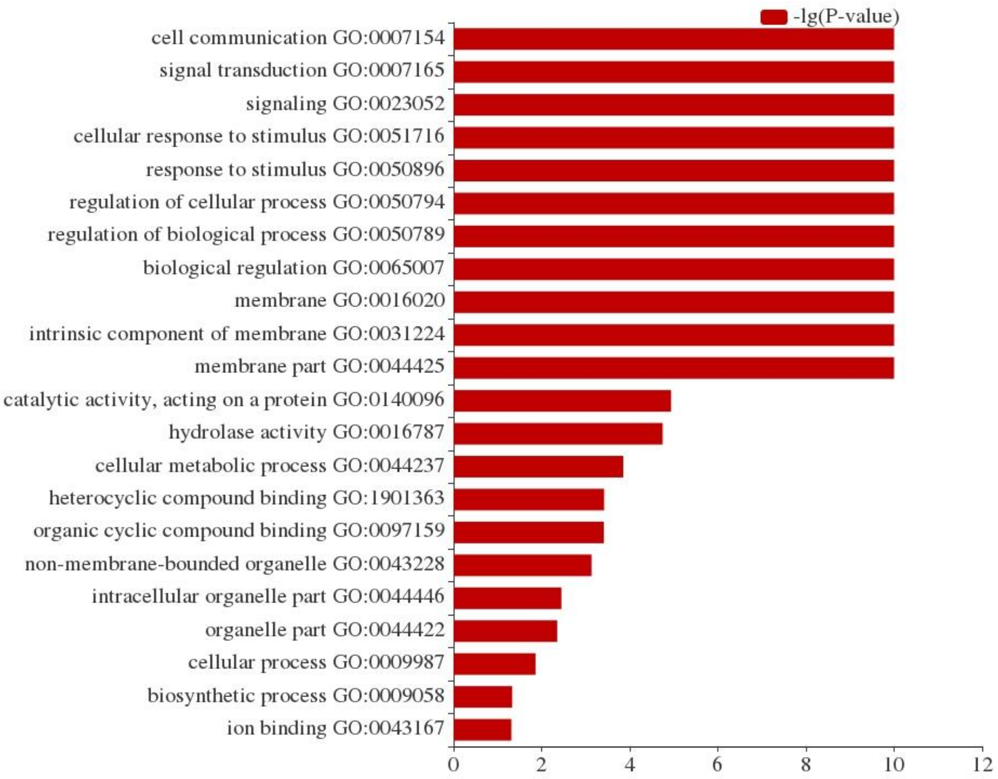

Supplementary Figure S5 Gene ontology enrichment of genes from contracted genes in masked palm civet. Red bars showed top enriched GO terms with the significant of p-value < 0.05.

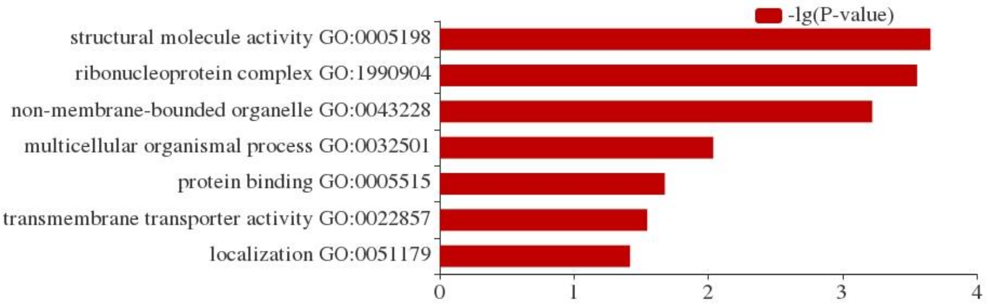

Supplementary Figure S6 Gene ontology enrichment of genes under positive selection in masked palm civet. Red bars showed top enriched GO terms with the significant of p-value < 0.05.

reply letter

Click here to access/download
Supplementary Material
reply.docx

Cover letter

Click here to access/download
Supplementary Material
cover letter.docx
