# Supplementary material for: The assembled and annotated genome of the masked palm civet (Paguma larvata)
Source: Gigascience. 2022 May 18;11:giac041. doi: 10.1093/gigascience/giac041 (PMC9116208; doi:10.1093/gigascience/giac041)
Supplement: giac041_GIGA-D-21-00297_Revision_1 [file giac041_giga-d-21-00297_revision_1.pdf]

GigaScience

The Assembled and Annotated Genome of the Masked Palm Civet (Paguma larvata)

--Manuscript Draft--

**Manuscript Number:**	GIGA-D-21-00297R1
**Full Title:**	The Assembled and Annotated Genome of the Masked Palm Civet (Paguma larvata)
**Article Type:**	Data Note
**Funding Information:**	GDAS Special Project of Science and Technology Development (2020GDASYL-20200103090) — DR Ping Liu<br>Guangdong Province project from the Forestry Administration of Guangdong Province — DR Ping Liu
**Abstract:**	Background: The masked palm civet (Paguma larvata) acts as an intermediate host of severe acute respiratory syndrome coronavirus (SARS-CoV), which caused SARS, and transfers this virus from bats to humans. Additionally, P. larvata has the potential to carry a variety of zoonotic viruses that may threaten human health. However, genome resources for P. larvata have not been reported to date. Findings: A chromosome-level genome assembly of P. larvata was generated using PacBio sequencing, Illumina sequencing, and Hi-C technology. The genome assembly was 2.44 Gb in size, of which 95.32% could be grouped into 22 pseudochromosomes, with contig N50 and scaffold N50 values of 12.97 Mb and 111.87 Mb, respectively. A total of 21,582 protein-coding genes were predicted, and 95.20% of the predicted genes were functionally annotated. Phylogenetic analysis of 19 animal species confirmed the close genetic relationship between P. larvata and species belong to the Felidae family. Gene family clustering revealed 119 unique, 362 significantly expanded, and 58 significantly contracted genes in the P. larvata genome. We identified 971 positively selected genes in P. larvata, and one known human viral receptor gene PDGFRA is positively selected in P. larvata, which is required for human cytomegalovirus (HCMV) infection, Conclusions: This high-quality genome assembly provides a valuable genomic resource for exploring virus-host interactions . It will also provide a reliable reference for studying the genetic bases of the morphological characteristics, adaptive evolution, and evolutionary history of this species.
**Corresponding Author:**	Jin-Ping Chen<br>Guangdong Academy of Sciences<br>Guangzhou, CHINA
**Corresponding Author Secondary Information:**	
**Corresponding Author's Institution:**	Guangdong Academy of Sciences
**Corresponding Author's Secondary Institution:**	
**First Author:**	Ping Liu
**First Author Secondary Information:**	
**Order of Authors:**	Ping Liu<br>Hai-Ying Jiang<br>Lin-Miao Li<br>Jia-Bin Zhou<br>Wen-Zhong Huang<br>Jin-Ping Chen
**Order of Authors Secondary Information:**	
**Response to Reviewers:**	Reviewer reports:

Powered by Editorial Manager® and ProduXion Manager® from Aries Systems Corporation

Reviewer #1: Liu et al. reported a chromosome-level genome assembly of Paguma larvata, which will provide a valuable genomic resource in investigating virus and host interactions because P.larvata was found to be an important host of SARS-Cov. The methods of genome assembly and gene prediction are appropriate; the continuity, completeness, gene number from the annotation of the P. larvata genome are also up to standard. Some suggestions are given for improving the quality of the manuscript.

1. To obtain reliable and correct results, the closely-related relatives of the masked palm civet, rather than Gallus, should be used as the outgroup in evolutionary analyses, especially the comparison of the evolutionary dynamics of gene families.
Reply: Thanks for your precious advice. We understand your opinion that the genetic relationship between gallus and ingroups is too distant. Therefore, in the new version of manuscript, we used opossum as outgroup, which is more closely related to ingroups than Gallus.

2. Because the authors simultaneously examined the positive selection of several genes, the results of statistical tests should be corrected by multiple testing.
Reply: Your advice is perfectly right. Actually, we used branch-site model to determine genes under positive selection, and likelihood ratio tests were conducted to determine whether positive selection was operating on the foreground branch. In this study, PSGs were identified only when $P < 0.01$. I am sorry that we didn't mention this in our manuscript before. We had added these details in "Positive selection analysis" section.

3. The gene IDs used in Supplementary Table S8 are not the gene symbol name, and it is better to annotate the gene symbol names to facilitate data utilization.
Reply: We are sorry for the mistake, we had added the gene symbol name in Supplementary Tables.

4. Line 157-159: Given that only five species were used for the homology-based prediction, I suggested adding a couple of mammalian species with high genome annotation quality, such as Homo sapiens, in this analysis, which will be helpful to further improve the annotation quality.
Reply: Thanks for your advice. The improvement of annotation quality is better to be annotated by adding more mammalian species. However, We annotated protein-coding genes in the Paguma larvata genome using a combination of de novo gene prediction, homology-based prediction, and RNA-seq-based prediction (Supplementary Table S4),which can help to get more annotated genes, and the results of downstream analyses based on these genes are all fine.

5. Please provide the versions of the softwares and the parameters used in the Methods part.
Reply: Thanks for your advice. The versions of softwares and the parameters are added in the Methods part of our manuscript.

6. line22，"with contig N50 and scaffold N50 values of 12.97 Mb, respectively" the authors seem to miss some words or values.
Reply: I am sorry that we did miss the scaffold N50 of 111.87 Mb, which has been added in the manuscript.

Reviewer #2: Reviewer's Comments
In this study, the authors provide a high-quality assembly of the Masked Palm Civet. This genome will be a valuable resource for comparative genomics. While the overall study is good, I felt that it lacked a cohesive narrative, with certain results either not making sense as written, or results being mentioned in passing but not explored further. I also found that certain paragraphs suffered from issues with grammar, such that the true meaning may be lost. Certain analyses e.g. RNA seq are mentioned, but never used to validate results. I believe there are major revisions needed for this study. Please see a list of major and minor comments below:

Minor comments
Line 44: The authors say posed as if it longer poses a threat, however I think this should be 'pose'.
Reply: Yes, we have corrected this word.

Line 50: Saying in recent decades but that something used to be domesticated almost seemed contradictory to me, but that might just be me.
Reply: Actually, the period of masked palm civet domesticated was short, just a few decades, so we said recent decades.

Line 90: I'm being a bit nit-picky here, but I feel like a final sentence or two explicitly stating why the genome was sequenced (e.g. to explore virus-host interactions) would be useful.
Reply: This is an excellent idea!

Line 122: I'm not sure if 'the FALCON' is the correct term to use here?
Reply: Yes, it is the correct term.

Line 139: you use the word 'using' twice in quick succession, perhaps consider something else?
Reply: Yes, your advice is precious. We have changed this sentence.

Line 215: a "." Is missing on this line.
Reply: Yes, it is, we have added a ".".

Line 300: How did the authors 'conduct' sequence identity? What is the importance of sequence identity compared to evidence of positive selection?
Reply: Sequence identity was calculated by p-distance in MEGA-X. The results may tell us the genetic relationship among different hosts. We focus on virus receptor genes under positive selection, which may be useful for study of tracing the origin and transmission of virus in the future.

Major comments

1. You mention that you aligned protein sequences using blast, however blast is not an alignment software. Am I missing something?
Reply: Thanks for your advice, we have changed the word of align to compare ?

2. What were the best-fit models of sequence evolution used with RAxML? Were they inferred? There are no mention of models used.
Reply: I am sorry that we did not mention these information in our manuscript. For cds sequences, we constructed phylogenetic tree based on GTRGAMMA model, while for protein sequences, we constructed phylogenetic tree based on PROTGAMMAAUTO model. Both trees shared the same topology. However, this information is about the old results. In our new results based on 19 species, we used IQ-TREE v1.6.10 which automatically detect the best model and then perform a maximum-likelihood (ML) phylogenetic tree for these species based on this best model.The best model of cds sequences calculated by IQ-TREE v1.6.10 was GTR+F+R3, while the best model of amino acid sequences was JTT+F+R5.

3. Were p-values from PAML corrected for multiple testing?
Reply: Branch-site model was used to determine genes under positive selection. Likelihood ratio tests were conducted todetermine whether positive selection was operating on theforeground branch. In this study, PSGs were identified only when$P < 0.01$.

4. The authors mention downloading sequences of receptors under selection and ACE2 from other animal species. It would be helpful if they listed them here. Is there a reason why the authors use mafft here but used muscle previously?
Reply: We are sorry about this mistake. We have listed other species that we get sequences of receptors under selection and ACE2. Actually, we used mafft and musclerandomly in different analysis, because we do not think there are differences between alignment results of them. In the new results, alignment of sequences was all conducted using muscle.

5. I am a little confused about the statement on line 255. The authors say that 87 gene families are unique to P. larvata. I find this number very difficult to believe. This would suggest that 87 different families of genes have subsequently emerged since sharing a last common ancestor with the cat and tiger. I think there could be a wording issue

Powered by Editorial Manager® and ProduXion Manager® from Aries Systems Corporation

here. But based on these gene numbers, and the divergence time quoted later (37.2 MY), that's one gene family per 427,586 with 'gene family' implying more than one gene. Can the authors clarify this?
Reply: I am sorry that we did not describe this question very clear. Actually, we means there are 196 unique genes in P. larvata, which belong to 87 gene families. These 87 gene family were not unique in P. larvata. It is worth noting that, in our new results, 119 genes were unique in P. larvata, and these genes are from 44 gene families.

6. Was selection detected in ACE2? If not, what does this mean for SARS-CoV in civets? I feel that the authors have avoided this but should discuss it in the text. What implications does this have for the theory that viral receptors in the civet have undergone change to avoid infection?
Reply: I am sorry that we did not clear this in our text. ACE2 did not positive selected in masked palm civet. However, in the backgroud of the ongong spread of SARS-CoV-2, which use ACE2 as receptor, we added analysis of this receptor in our text. The sequence similarity may reflect the potential of binding viruses to some extent. However, there are other factors, such as binding loci, could affect the virus binding ability. Actually, virus could infect civet cells, but no particularly severe symptoms appear. The mechanism of civet to resistant viruses is a complex question which need further study. In this study, we just studied this question based on the sequence of receptors.

7. The authors state that there were more protein coding genes in the civet than the tiger and cat. Do these genes come from the expansions of certain families, or are they completely absent in other species? Is it possible that alleles of genes from reverse strands have been incorporated in the genome? Do these extra genes show up in the RNA seq data?
Reply: I mentioned that there were more protein coding genes in civet than tiger and cat just want to say, the annotation results of civet is better than cat and tiger, two relatively related species. However, genome of these two species were generated in 2007 and 2013, about ten years ago, and maybe that is why the results are not perfect. In our new analysis, we used five species in the family of Felidae closely related to civet, and we realize the comparison with gene numbers makes no sense, and this sentence has been deleted.

8. Were any of the expanded immunoglobulin genes under selection? These seem to be mentioned once in the result but no more if mentioned about them.
Reply: We are sorry for missing these results. However, we did not observe genes under positive selection overlapped with expanded and contracted genes. This information has been added to “Gene family comparison” section of “Results” this manuscript.

Reviewer #3: The manuscript by Liu et el reports the assembled and annotated genome of the masked Palm Civet (Paguma larvata) which is a good contribution given the role of this organism in acting as an intermediate host of severe acute respiratory syndrome coronavirus (SARS-CoV). The assembly is performed well and is of acceptable quality, however I have some major comments on the analysis part which are mentioned below.

i) The reported genome assembly of Paguma larvata is of good reference quality achieved using short read, long read and Hi-C technology. But despite of having an N50 value of 12.97 Mb, the BUSCO completeness is only 95 % which much lower than expected. Do the authors have an explanation for it or I suggest that it should be carried out again.
Reply: Thanks for your advice! As for the BUSCO result, we carried out again using BUSCO v5 (95% was resulted from BUSCO v3), and 96.5% of the 9,226 BUSCOs were assembled. I don’t know whether this new results could make sense.

ii) The methodology for prediction of non-coding RNAs should be mentioned in the main manuscript. In line 248-250, I noted that the number of predicted miRNA, tRNA genes seems to be much higher in this genome, which should be carefully checked.
Reply: The methods for prediction of non-coding RNAs have been added in “Gene prediction and functional annotation” section. We analyzed non-coding RNAs prediction again, and the same results were obtained. We are also confused about

Powered by Editorial Manager® and ProduXion Manager® from Aries Systems Corporation

	this. We also checked results of BUSCO, complete and duplicated BUSCOs is 1.3%, meaning there is not data Redundancy. Maybe the high miRNA and tRNAis the personality of this species.<br><br>iii) RNA-Seq data was generated from seven different tissues of Paguma larvata. This amount of data can be used for more detailed analysis e.g., differential gene expression profile of immunity-related genes in these different tissues. This analysis will add more value to the work.<br>Reply: We appreciate your advice. We did sequenced RNA-Seq data from seven different tissues, but these tissues all from one individual. RNA-Seq data in this study was mainly used to conduct gene prediction. Actually, we planned to collect tissues in different individuals for RNA-Seq. However, after the break out of SARS-CoV-2, experiments on wild animals such as civet who may related to this virus are carried out with great caution. Therefore, we have to give up this idea unfortunately due to various irresistible factors.<br><br>iv) I am surprised to find that the Phylogenetic analysis is performed using only 11 species when several high-quality genomes are available. This should be performed using more species, which can then be used as a good reference in future studies. Given the significance of this genome in SARS, a comprehensive phylogenetic analysis is much needed.<br>Reply: Thanks for your advice. We had reanalyzed phylogenetic analysis using 19 species. Phylogenetic relationship between civet and animals from Felidae, Canidae, Ursidae, Bovidae, Suidae, as well as human, mouse, and rabbit is clear now.<br><br>v) In case of positive selection analysis, pig (Sus scrofa) seems to be a comparatively distant species with respect to the other selected species. Inclusion of phylogenetically closer and a higher number of animal species will help in identification of the positively selected genes in this species with more precision and specificity.<br>Reply: Thanks for your advice! We have re-conducted positive selection analysis using Paguma larvata,C. lupus familiaris, and the five most closely relatedspecies from the Felidae familywith assembled genomes: F. catus, Panthera pardus, Panthera leo, L. canadensis, and Panthera tigris.
**Additional Information:**	
**Question**	**Response**
Are you submitting this manuscript to a special series or article collection?	No
**Experimental design and statistics**<br><br>Full details of the experimental design and statistical methods used should be given in the Methods section, as detailed in our Minimum Standards Reporting Checklist. Information essential to interpreting the data presented should be made available in the figure legends.<br><br>Have you included all the information requested in your manuscript?	Yes
**Resources**<br><br>A description of all resources used,	Yes

including antibodies, cell lines, animals and software tools, with enough information to allow them to be uniquely identified, should be included in the Methods section. Authors are strongly encouraged to cite Research Resource Identifiers (RRIDs) for antibodies, model organisms and tools, where possible.<br><br>Have you included the information requested as detailed in our Minimum Standards Reporting Checklist?	
**Availability of data and materials**<br><br>All datasets and code on which the conclusions of the paper rely must be either included in your submission or deposited in publicly available repositories (where available and ethically appropriate), referencing such data using a unique identifier in the references and in the "Availability of Data and Materials" section of your manuscript.<br><br>Have you have met the above requirement as detailed in our Minimum Standards Reporting Checklist?	Yes

Revision tracked version
Click here to access/download;Manuscript;paper-20220211.docx

The Assembled and Annotated Genome of the Masked Palm Civet (*Paguma larvata*)

Ping Liu[1], Hai-Ying Jiang[1], Lin-Miao Li[1], Jia-Bin Zhou[1], Wen-Zhong Huang[1], Jin-Ping Chen[1]

[1]Guangdong Key Laboratory of Animal Conservation and Resource Utilization, Guangdong Public Laboratory of Wild Animal Conservation and Utilization, Institute of Zoology, Guangdong Academy of Sciences, Guangzhou 510260, China; pingliu0330@126.com

Corresponding author: Jin-Ping Chen, Institute of Zoology, Guangdong Academy of Sciences, Guangzhou 510260, China. chenjp@giz.gd.cn; Tel.: +020-8910-0920

Abstract

Background: The masked palm civet (*Paguma larvata*) acts as an intermediate host of severe acute respiratory syndrome coronavirus (SARS-CoV), which caus~~es~~ed SARS, and transfers this virus from bats to humans. Additionally, ~~*Paguma*~~*P.* *larvata* has the potential to carry a variety of zoonotic viruses that may threaten human health. However, genome resources for ~~*Paguma*~~*P.* *larvata* have not been reported to date. **Findings:** A chromosome-level genome assembly of ~~*Paguma*~~*P.* *larvata* was generated using PacBio sequencing, Illumina sequencing, and Hi-C technology. The genome assembly was 2.44 Gb in size, of which 95.32% could be grouped into 22 pseudochromosomes, with contig N50 and scaffold N50 values of 12.97 Mb and 111.87 Mb, respectively. A total of 21,582 protein-coding genes were predicted, and 95.20% of the predicted genes were functionally annotated. Phylogenetic analysis of 1~~1~~9 animal species confirmed the close genetic relationship between ~~*Paguma*~~*P.* *larvata* and species belong to the Felidae family~~, *Panthera tigris*, and *Felis Catus*~~. Gene family clustering revealed ~~87~~119 unique, 3~~1~~62 significantly expanded, and ~~139~~58 significantly contracted gene~~ families~~s in the ~~*Paguma*~~*P.* *larvata* genome. We identified ~~285~~971

Formatted: Font: Not Italic

Formatted: Not Highlight

Formatted: Not Highlight

positively selected genes in ~~*Paguma*~~*P.* *larvata*, and ~~four~~ one known human viral receptor gene~~s~~ *PDGFRA* is positively selected in *P. larvata*, which is required for human cytomegalovirus (HCMV) infection~~,~~, ~~were positively selected in *Paguma larvata*.~~ **Conclusions:** This high-quality genome assembly provides a valuable genomic resource for exploring virus-host interactions ~~elucidating immune mechanisms in this animal~~. It will also provide a reliable reference for studying the genetic bases of the morphological characteristics, adaptive evolution, and evolutionary history of this species.

Keywords: Masked palm civet, genome assembly, Hi-C proximity mapping, gene family evolution, phylogeny, positive selection

Introduction

In human history, the spread of viruses has caused huge losses to the human economy and threatened the safety of human life and property. Diseases affecting animals of economic value, caused by viruses such as avian influenza and African swine fever, have caused serious economic losses [1-3]. Viruses such as severe acute respiratory syndrome coronavirus (SARS-CoV), Middle East respiratory syndrome coronavirus (MERS-CoV), and SARS-CoV-2, pose~~d~~ a great threat to the safety of human life and property [4-6]. Usually, zoonotic viruses can be transmitted to humans via wild ~~animals~~life as intermediate hosts. For example, MERS-CoV is transmitted from camels to humans [7].

Masked palm civets, *Paguma larvata* (Mammalia, Carnivora, and Viverridae) are wild mammals ~~that are~~ widely distributed in Asia [8]. They are omnivores, although they mainly consume fruits. In recent decades, ~~*Paguma*~~*P.* *larvata* used to be domesticated as meat of human beings, increasing the possibility of transferring pathogenic microorganisms to human beings. In 2003, ~~*Paguma*~~*P.* *larvata* gained attention because of its potential involvement in the outbreak of severe acute respiratory syndrome (SARS), which originated in southern China and spread to over 30 countries [9]. Further research suggest~~s~~ed that ~~*Paguma*~~*P.* *larvata* act~~s~~ed as an intermediate host of SARS-CoV, which transmits this virus from bats to humans [10, 11]. The sequence

Formatted: Font: Italic

Formatted: Font: (Default) Times New Roman, 12 pt

Formatted: Font: (Default) Times New Roman, 12 pt

Formatted: Font color: Text 1

similarity between the SARS-CoV found in ~~*Paguma*~~*P.* *larvata* and human SARS-CoV is greater than 99%. However, ~~*Paguma*~~*P.* *larvata* can live in harmony with SARS-CoV, whereas humans can have severe symptoms following SARS-CoV infection. This shows that the interaction between ~~*Paguma*~~*P.* *larvata* and viruses has a certain particularity [12]. Earlier studies on farms raising ~~*Paguma*~~*P.* *larvata* also found that these animals rarely become ill. The immune system of ~~*Paguma*~~*P.* *larvata* is thus worthy of in-depth study.

Virus receptors, present on the host cell membrane, are important factors in mediating virus invasion and play an extremely important role in the interaction between host cells and viruses. The virus attachment protein can be regarded as the “key” that unlocks the cell by interacting with the “lock” (receptor) on the surface of the host cell, starting a key downstream step in the virus life cycle. In many cases, viruses use multiple receptors to perform different functions during the viral life cycle [13]. Therefore, the virus must first find the lock and then use a specific virus “key” to unlock the cell. The numerous functions of the virus receptor aid the virus in targeting the correct tissue for infection and to cross the cell barrier, which is necessary to deliver the viral genome to the host cell [14]. The interaction with viral receptors is usually mediated by specific proteins on the surface of the virus [13]. Inherent differences in the shape of the virus surface protein (icosahedron or helix) and the composition of the virus coat affect the overall structure of the virus attachment protein. Some viruses infect only certain species; for example, swine fever virus can only infect pigs [3]. Whereas some viruses can spread between animals and humans, such as SARS-CoV [10, 11] and MERS-CoV [7]; the reason lies in the nature of the interaction between the virus receptor and virus attachment protein. For example, SARS-CoV and SARS-CoV-2 specifically recognize the angiotensin-converting enzyme 2 (*ACE2*) receptor [15, 16, 17]. Understanding the nature of virus receptors that cause zoonotic diseases in different species will help in tracing natural hosts during the virus epidemic.

Formatted: Font: Italic

As an important virus host, masked palm civets can be used as a system for reviewing virus and host interactions. However, there is currently no genome assembly of ~~*Paguma*~~*P.* *larvata*, even in Viverridae which has been reported for studying various virus receptors. In this study, we generated a chromosome-level reference genome of

~~*Paguma*~~*P.* *larvata* by integrating PacBio sequencing, Hi-C sequencing, and Illumina sequencing [18-20]. The high-quality genome assembly will provide a valuable genomic resource for ~~elucidating~~ exploring virus-host interactions ~~the immune mechanisms in this animal~~. It will also provide a reliable reference for studying the genetic bases of the morphological characteristics, adaption evolution, and evolutionary history of the species.

Data Description

Statement of Ethics

The study design was approved by the Ethics Committee for Animal Experiments at the Institute of Zoology, Guangdong Academy of Sciences (reference number: GIZ20190702, 07/01/2019) and followed the basic principles outlined by this committee.

Isolation of genomic DNA and RNA

A healthy female ~~*Paguma*~~*P.* *larvata* individual was collected from a farm in Wengyuan County, Guangdong Province, China. Whole blood was isolated from the same masked palm civet and washed with saline nasal spray~~.~~, ~~T~~these~~se samples were~~ n immediately frozen in liquid nitrogen and stored at -80 °C prior to nucleic acid extraction. Genomic DNA was extracted from whole blood using a magnetic universal genomic DNA kit. Different tissues, including heart, intestine, kidney, liver, lung, muscle, and spleen were sampled from the same one female *P. larvata* individual and flash-frozen in liquid nitrogen for ~~total~~ RNA sequencing, RNA-seq data was used to predict genes in following analysis. Total RNA was extracted using the TRIzol reagent [21].

Genome sequencing with different technologies

A combined sequencing strategy was used to obtain the masked palm civet genome. Short-insert libraries with a size of 350 bp were sequenced on the Illumina HiSeq 2500 platform using the 150 bp paired-end protocol. For PacBio library construction, we sheared the genomic DNA to 20 kb fragments, which we used to prepare a single-

molecule real-time (SMRT) library for sequencing with the PacBio Sequel system [22]. Liver tissue was used to construct the Hi-C sequencing library. Briefly, cross-linked chromatin was digested with Dpn II and ligated in situ after biotinylation. DNA fragments were enriched via the interaction of biotin and blunt-end ligation and then subjected to Illumina NovaSeq PE 150 sequencing [23].

Genome assembly and quality assessment

We applied the FALCON v0.3.0 [24] to correct errors in PacBio long reads, according to PacBio short reads (<5 kb) and then generated consensus sequences. The primary scaffolds were assembled with these subreads, and the scaffolds were further corrected in PILON v1.22 [25] using short sequencing reads. To anchor scaffolds onto chromosomes, we aligned the Hi-C sequencing data to the assembly using BWA v0.7.17 [26] and detected valid contacts. Preassembled scaffolds were clustered, ordered, and directed onto pseudochromosomes using the LACHESIS software [27]. To increase the accuracy of the assembled genome, we artificially corrected the LACHESIS-based assembly, filled gaps, and removed duplicate sequences [28].

Genome size was estimated based on k-mer distribution analysis with the program in GCE [29] using Illumina short reads. We used Benchmarking Universal Single-Copy Orthologs (BUSCO v5) [30] and Core Eukaryotic Genes Mapping Approach (CEGMA) [31] to evaluate the completeness and accuracy of the genome assembly and BWA v0.7.17 [26] to align the Illumina short reads to the assembly and evaluate the assembled portion. We assembled expressed sequencing tags (ESTs) using Trinity v2.8.4 [32] with RNA reads from seven different tissues (heart, liver, spleen, lung, kidney, muscle, and intestine). To further examine the quality of the genome assembly, we aligned ESTs to the assembled genome using BLAT [33] ~~using~~ with parameters of identity ≥90% and coverage ≥50%. The quality of the genome assembly was evaluated by mapping RNA-Seq reads ~~from the five different tissues~~ to the ESTs using TopHat2 [34].

Repetitive elements annotation

Repetitive sequences were identified at both the DNA and protein levels by

integrating homology-based prediction and *de novo* identification. Repeat masking was performed based on repeats in the Repbase TE library from the Repbase server [35]. Using this library, we predicted interspersed repeat elements using RepeatMasker v3.3.0 and ProteinMask (http://www.repeatmasker.org) and screened tandem repeats using TRF v4.07b [36].

Gene prediction and functional annotation

We annotated protein-coding genes in the *~~Paguma~~P. larvata* genome using a combination of *de novo* gene prediction, homology-based prediction, and RNA-seq-based prediction. For *de novo* identification, we predicted the gene models using five *ab initio* gene prediction programs: Augustus [37], GlimmerHMM [38], SNAP [39], Genscan [40], and Geneid [41]. We ~~align~~compared~~ed~~ proteins from five sequenced animals, namely, *Mus musculus* [42], *Canis lupus familiaris* [43], *Felis catus~~F. catus~~* [44], *Panthera tigris altaica~~Panthera tigris~~* [45], and *Acinonyx jubatus* [46], to the *~~Paguma~~P. larvata* assembly using tBlastN [47] with an e-value cut-off of 1×10^{-5}. Gene models were generated using GeneWise v2.2.0 [48]. We applied two methods of RNA-Seq-based prediction: (i) we mapped RNA-Seq data to the genome using TopHat2 [49] and further generated the gene models in Cufflinks [34] based on exons, and (ii) we aligned ESTs against the assembly to generate the gene models using PASA [50]. We integrated predictions from the three approaches in EVidenceModeler v1.1.1 [51] to generate a non-redundant gene model set. The final gene models were annotated using the non-redundant protein database of NCBI (NR) and Swissprot, Kyoto Encyclopedia of Genes and Genomes (KEGG) [52], Interprot [53], and Pfam databases v14.1 [54].

Noncoding RNAs include tRNA, rRNA, miRNA, and snRNA. tRNAscan-SE v1.4 (http://lowelab.ucsc.edu/tRNAscan-SE/) was used to find the tRNA sequence in the genome according to the structural characteristics of tRNA. rRNA is highly conserved, and it is possible to select a closely related species as the reference sequence, and rRNA in the genome is searched by Blast v2.2.26 [47]. The INFERNAL (http://infernal.janelia.org/) software that comes with Rfam v14.1 [54] was used predict the miRNA and snRNA in the genome

using the covariance model of the Rfam family.

Formatted: Font color: Auto

Formatted: Indent: First line: 2 ch

Phylogenetic analysis and estimation of divergence time

We used OrthoFinder v2.5.2 [55]~~the Treefam protocol [54]~~ to identify orthologous genes from masked palm civet and ~~ten~~18 other species, including cat (*F. catus*), cattle (*Bos taurus*), dhole (*Cuon alpinus*), dog (*C. lupus familiaris*), human (*Homo sapiens*), leopard (*Panthera pardus*), lion (*Panthera leo*), lynx (*Lynx canadensis*), mouse (*M. musculus*), opossum (*Monodelphis domestica*), panda (*Ailuropoda melanoleuca*), pig (*Sus scrofa*), polarbear (*Ursus maritimus*), rabbit (*Oryctolagus cuniculus*), red fox (*Vulpes vulpes*), tiger (*P. tigris altaica*), wolf (*Canis lupus*), and yak (*Bos mutus*)~~cat (*F. catus*), chicken (*G. gallus*), dog (*C. familiaris*), giant panda (*Ailuropoda melanoleuca*), human beings (*Homo sapiens*), mouse (*M. musculus*), pig (*Sus scrofa*), polar bear (*Ursus maritimus*), tiger (*Panthera tigris*), and yak (*Bos mutus*)~~ (Supplementary Table S1). Diamond was used for sequence similarity searches. We aligned the coding region ~~sequences~~and amino acid sequences of single-copy orthologous genes using MUSCLE v3.7 [5~~5~~6]. All alignments were concatenated to a super-alignment matrix. Based on this matrix, we used IQ-TREE v1.6.10 [57]~~RAxML v8.0.19 [56]~~ which automatically detect the best model and then~~to~~ perform a maximum-likelihood (ML) phylogenetic tree~~ for these species~~ based on this best model. 1,000 bootstrap replicates were performed to obtain confidence values for the branches. Bayesian Evolutionary Analysis Sampling Trees (BEAST) v2.6.0 [58] was used to estimate species divergence times based on amico acid sequences of single-copy genes. The tree topology based on IQ-TREE v1.6.10 [56] was used as starting tree, while ~~The MCMCTree program of PAML v4.9 [57] was used to estimate species divergence times using single-copy orthologs based on~~ splits between tiger and leopard (7.4 Ma), cat and tiger (15.2~~12.3–18.1~~ Ma), polar bear and giant bear (~~17.8–28~~23.4~~.9~~ Ma), ~~dog~~ pig and ~~polar bear~~yak (~~42.0–49~~62.0 Ma), mouse and human (90.0 Ma), as well as ~~dog~~humans and ~~cats~~ tiger (~~52.0–57~~96.0 Ma)~~, as well as human beings and mice (85.0–94.0 Ma)~~ from TimeTree (http://www.timetree.org/, last accessed February 8, 2022~~March 17, 2021~~) as calibration. We ran the Markov chain Monte Carlo for 10 million generations with sampling every 1000 generations, and 25% of the generations were discarded as burn-in. TreeAnnotator in BEAST v2.6.0 [58] was used to summarize the information from

Formatted: Not Highlight
Formatted: Font: Italic
Formatted: Font: Italic
Formatted: Font: Italic
Formatted: Font: Italic
Formatted: Font: Italic
Formatted: Font: Italic
Formatted: Font: Italic
Formatted: Font: Italic
Formatted: Font: Italic
Formatted: Font: Italic
Formatted: Font: Italic
Formatted: Font: Italic
Formatted: Font: Italic
Formatted: Font: Italic
Formatted: Font: Italic
Formatted: Font: Italic
Formatted: Font: Italic
Formatted: Not Highlight
Formatted: Font: (Default) Times New Roman, 12 pt
Formatted: Font: (Default) Times New Roman, 12 pt
Formatted: Font: (Default) Times New Roman, 12 pt

trees produced by BEAST v2.6.0 [58] onto a single target tree, while the target tree was visualized and exported as vector diagrams using FigTree v1.4.4 (http://tree.bio.ed.ac.uk/software/figtree/).

Gene family expansion and contraction analysis

To identify gene family evolution as a random birth and death process model, where the gene family either expands or contracts per gene per million years independently along each lineage of the phylogenetic tree, the ML model originally implemented in the software package CAFE v4.2.1 [5~~8~~9] was applied to compare the cluster size differences (gain or loss) between the ancestor and each species. We used species tree with divergence time obtained by BEAST v2.6.0 [58] as ultrametric tree. We determined gene family significantly expansions or contractions ~~only~~ when the ~~change in gene copy number~~P value was ~~significant (P <~~less than 0.05~~)~~.

Positive selection analysis

For positive selection analysis, we first identified single-copy orthologous genes from ~~*Paguma*~~P. *larvata*, C. lupus familiaris, and the five most closely related species from the Felidae family with assembled genomes~~four other species~~: F. catus, P. pardus, P. leo, L. canadensis, and P. tigris altaica~~*Panthera tigris, A. melanoleuca, S. scrofa, and F. catus*~~. For these genes, based on phylogenetic topology, we employed the branch-site model incorporated in the PAML package v4.9 [~~59~~60] to detect positively selected genes (PSGs). ~~*Paguma*~~P. *larvata* was specified as a foreground branch, the other species were used as background branches. ~~,~~ To determine genes under positive selection, likelihood ratio tests were conducted to determine whether positive selection was operating on the foreground branch. In this study, PSGs were identified only when P < 0.01. Receptors were important factors to intermediate virus infection. To study the immune mechanism of P. larvata, we looked out 28 known human virus receptors [61] in positive selected genes. ~~and PSGs were identified only when P < 0.01.~~

Sequence analysis of virus receptors under positive selection and *ACE2*

Sequences of virus receptor genes under positive selection and the *ACE2* gene in

Formatted: Font: Not Italic
Formatted: Font: Not Italic
Formatted: Font: Not Italic
Formatted: Font: Not Italic
Formatted: Font: Not Italic
Formatted: Font: Not Italic
Formatted: Font: Not Italic
Formatted: Font: Italic
Formatted: Font: Italic

other animal species of cat (*F. catus*), cattle (*B. taurus*), dhole (*C. alpinus*), dog (*C. lupus familiaris*), human (*H. sapiens*), leopard (*P. pardus*), lion (*P. leo*), lynx (*L. canadensis*), mouse (*M. musculus*), opossum (*M. domestica*), panda (*A. melanoleuca*), pig (*S. scrofa*), polarbear (*U. maritimus*), rabbit (*O. cuniculus*), red fox (*V. vulpes*), tiger (*P. tigris altaica*), and yak (*B. mutus*) were downloaded from NCBI (https://www.ncbi.nlm.nih.gov/). Though *ACE2* may not positive selected in *P. larvata*, it is receptor of SARS-CoV and SARS-CoV-2, both resulting in severe pneumonia in human beings. Therefore, we want to know the genetic relationship of virus receptors between *P. larvata* and other animals. Alignment of ~~coding region sequences and~~ amino acid sequences of receptors was conducted using MUSCLE v3.7 [56]. ML phylogenetic trees were constructed using IQ-TREE v1.6.10 [~~61~~57]~~.~~, and 1,000 bootstrap replicates were performed to obtain confidence values for the branches. The sequence identity of amino acid sequences which could demonstrate the genetic distance between receptor genes in different animals was estimated ~~using~~ by p-distance in MEGA-X [6~~2~~2].

Formatted: Font: Italic

Formatted: Font: Not Italic

Formatted: Font: (Default) Times New Roman, 12 pt

Formatted: Font: (Default) Times New Roman, 12 pt

Formatted: Font: Not Italic

Formatted: Indent: First line: 0 ch

Results and Discussion

Genome sequencing and assembly

We sequenced the genome of one female masked palm civet (2n = 44) (Figure 1a). Here, 557.75 Gb sequences were generated from high-quality short-read sequences (272.68 Gb) and PacBio sequences (285.07 Gb), representing over 216× coverage for the genome~~,~~. ~~which~~The genome has an estimated size of 2.58 Gb based on the 17-mer depth distribution analysis of the sequenced reads (Supplementary Figure S1) The final assembled sequence was 2.44 Gb, representing 94.6% of the masked palm civet genome. The assembly consisted of 1,785 scaffolds with an N50 of and 111.87~~12.97~~ Mb (Table 1). We used the Hi-C technology to reorder and anchor 95.32% of the genome onto 22 pseudochromosomes (Figure 1a, Supplementary Figure S2). The GC content and heterozygosity rate of the assembled masked palm civet genome were 42.4% and 0.46%, respectively. The completeness of our assembly was assessed by BUSCO and CEGMA. The BUSCO assessment showed that 96.5% of the 9,226 BUSCOs were assembled, with only 1.3% duplication (Supplementary Table S2), while CEGMA assessment

showed that 91.94% and 3.2% of 248 CEGs (Core Eukaryotic Genes) were complete and partially assembled, respectively (Supplementary Table S3). We also used BWA v0.7.17 [26] to align Illumina short reads to the assembly and obtained a mapping rate and genome coverage of 99.35% and 99.73%, respectively. Overall, all evidence suggests that the ~~*Paguma*~~*P.* *larvata* assembly is a high-quality genome assembly that can be used for ~~further~~ downstream analyses.

Repeat annotation and gene prediction

We analyzed repetitive sequences by combining *de novo* prediction and a homology-based search at both the DNA and protein levels, and found that repetitive sequences occupied 31.80% (775.0 Mb) of the genome of the masked palm civet (Table 1, Figure 1a), which was very similar to the repeat compositions of the cat genome (32.10%), but lower than that of the tiger genome (39.20%) [44, 45]. The most abundant repetitive element type in the masked palm civet genome was long terminal repeat (LTR), representing 542.79 Mb (22.27%) of the genome. ~~We performed a~~An annotation pipeline combining *de novo*, homolog-based search, and RNA-Seq methods was performed to predict gene models from the repeat-masked masked palm civet genome sequence. These analyses predicted 21,582 protein-coding genes, representing 30.61% of the genome assembly (Supplementary Table S4, Supplementary Figure S3a). Among the 21,582 genes, 20,546 (95.20%) were successfully annotated for at least one function term by searching against functional databases (Gene Ontology (GO), InterProScan, KEGG, NR, Pfam, and SwissProt) (Table 1, Supplementary Figure S3b). ~~The number of protein-coding genes in the *Paguma larvata* genome is slightly higher than that in the cat and tiger genomes (20,285 and 20,226 genes, respectively) [43, 44] (Pontius et al. 2007; Cho et al. 2013).~~ In addition to the protein-coding genes, we also identified 14,829 miRNA genes, 47,575 tRNA genes, and 544 rRNA genes in the masked palm civet genome (Supplementary Table S5). The number of miRNA and tRNA genes seemed higher than other species. We double checked the methods and conducted noncoding RNAs prediction again, and obtained the same results. Maybe the high level of miRNA and tRNA genes is the personality of this species, which need further analysis in the future.

Gene family comparison

The evolutionary dynamics related to ~~*Paguma*~~*P.* *larvata* were deduced by comparison with ten animal species. We identified 1,438 ~~5,417~~ one-to-one orthologs among these species (Figure 1b; Supplementary Table S6). Among the 1~~1~~9 species, ~~87~~44 gene families containing 119 genes were unique ~~to~~in ~~*Paguma*~~*P.* *larvata* (Supplementary Table S6)~~.~~, containing a immune-related gene named *VSIG10L*, which is related to familial Barrett neoplasia [63]. Based on the P value threshold of 0.05, ~~A total of 312~~35 gene families (including ~~1,147~~243 genes) were significantly expanded ~~in the masked palm civet.~~; ~~I~~in contrast, ~~139~~89 gene families (including ~~276~~58 genes) were significantly contracted in the ~~*Paguma*~~*P.* *larvata* genome (Figure 1~~b~~c). There were 10 immune-related genes in significantly expanded genes, which were annotated to 3 genes of *TARM1* (T cell-interacting, activating receptor on myeloid cells-1), *IGLL1* (immunoglobulin lambda like polypeptide 1), and *OSCAR* (osteoclast associated Ig-like receptor) (Supplementary Table S7). *TARM1* is a novel leukocyte receptor complex which could encoded ITAM receptor that costimulates proinflammatory cytokine secretion by macrophages and neutrophils [64]. *TARM1* enables immunoglobulin receptor binding activity. *IGLL1* is a protein coding gene. Diseases associated with *IGLL1* include Agammaglobulinemia 2, Autosomal Recessive and Lambda 5 Deficiency [65]. *OSCAR* is a member of the leukocyte receptor complex protein family that plays critical roles in the regulation of both innate and adaptive immune responses [66]. The encoded protein may play a role in oxidative stress-mediated atherogenesis as well as monocyte adhesion. Recent studies showed that *OSCAR* can promote proliferation and migration of lung adenocarcinoma in women [67]. We did not detcted immune-related genes in significantly contracted genes, ~~The expanded genes were enriched in 51 GO terms, and 43 immunoglobulin-related genes were included (Supplementary Table S7, Supplementary Figure S4). The contracted genes were enriched in 22 GO terms, among which there were 13 immunoglobulin-related genes (Supplementary Table S8, Supplementary Figure S5). There were more immunoglobulin-related genes expanded than contracted,~~ which was in accordance with the high immune response level of masked palm civets.

Formatted: Font: Italic
Formatted: Not Highlight
Formatted: Not Highlight
Formatted: Not Highlight
Formatted: Not Highlight
Formatted: Not Highlight
Formatted: Not Highlight
Formatted: Not Highlight
Formatted: Not Highlight
Formatted: Font: Italic
Formatted: Font: (Default) Times New Roman, 12 pt, Italic
Formatted: Font: (Default) Times New Roman, 12 pt, Italic
Formatted: Font: Italic
Formatted: Not Highlight
Formatted: Font: Italic
Formatted: Font: (Default) Times New Roman, 12 pt
Formatted: Font: (Default) Times New Roman, 12 pt
Formatted: Font: (Default) Times New Roman, 12 pt
Formatted: Font: (Default) Times New Roman, 12 pt
Formatted: Font: (Default) Times New Roman, 12 pt
Formatted: Font: (Default) Times New Roman, 12 pt
Formatted: Font: (Default) Times New Roman, 12 pt
Formatted: Font: (Default) Times New Roman, 12 pt
Formatted: Font: (Default) Times New Roman, 12 pt
Formatted: Font: (Default) Times New Roman, 12 pt
Formatted: Font: (Default) Times New Roman, 12 pt
Formatted: Font: (Default) Times New Roman, 12 pt
Formatted: Font: (Default) Times New Roman, 12 pt
Formatted: Font: (Default) Times New Roman, 12 pt
Formatted: Font: Italic
Formatted: Font: (Default) Times New Roman, 12 pt
Formatted: Font: Italic
Formatted: Font: Italic
Formatted: Font: Italic
Formatted: Font: Italic

Phylogenetic Analysis

To confirm the phylogenetic position of ~~*Paguma*~~P. *larvata* and estimate divergence time using whole-genome data, we analyzed the orthologous gene relationships between ~~*Paguma*~~P. *larvata* and ~~ten~~18 other animals (Supplementary Table S~~see “Phylogenetic analysis and estimation of divergence time” section of “Materials and methods”~~1). Based on a super alignment matrix of ~~coding~~amino acid~~–~~ sequences and coding region sequences~~region sequences~~ of 1,438~~5,417~~ single-copy ~~ortholog~~genes with opopssum~~*G. gallus*~~ as ~~the~~ outgroup. The best model of cds and amino acid sequences were GTR+F+R3 and JTT+F+R5. Both trees shared the same topology~~The phylogenetic analysi~~ thats~~–~~ ~~supported~~ the genetic relationship between ~~*Paguma*~~P. *larvata*~~, *Panthera tigris*,~~ and 5 species belong to the Felidae family~~F. Catus~~ were closely related~~,~~. The phylogenetic tree based on amino acid sequences was used as starting tree to calculate the divergence time, which~~whereas the~~ resulted the time of ~~*Paguma*~~P. *larvata* split with the Felidae family~~*Panthera tigris*~~ was approximately ~~37.2~~33.5 ~~(~~million years ago (Figure 1c). ~~We~~ This is the first time of obtaining~~obtained~~ the divergence time of masked palm civets ~~for the first time~~ based on whole genome sequences.

Genes under positive selection~~and ACE2 gene~~

We used ~~*Paguma*~~P. *larvata* as the foreground branch, and other species of *C. lupus familiaris, F. catus, P. pardus, P. leo, L. canadensis, and P. tigris altaica*~~*Panthera tigris, A. melanoleuca, S. scrofa*, and *F. catus*~~ as background branches to detect genes under selection. Based on ~~9~~5,~~414~~ 696 single-copy genes of these ~~five~~seven species, We identified ~~285~~971 PSGs (P < 0.01; Supplementary Table S9). ~~A GO functional classification of significant PSGs in *Paguma larvata* revealed that the majority of genes under positive selection (194 genes) were associated with protein binding (Supplementary Table S10, Supplementary Figure S6).~~ We did not observe genes under positive selection overlapped with expanded and contracted genes. ~~Currently, humans have reported more than 28 types of viral receptors [63]. Four~~ One of these known human viral receptor genes, ~~*CD55*, *HSPG2*,~~ *PDGFRA*~~, and *SCARB2*~~, were included in

Formatted: Font: Not Italic

Formatted: Font: Not Italic

Formatted: Not Highlight

Formatted: Font: Italic

Formatted: Font: Italic

genes under positive selection in ~~*Paguma*~~*P. larvata* ~~*(Supplementary Table S11), which*~~ . *PDGFRA* could activating mutations in gastrointestinal stromal tumors [68], and is required for human cytomegalovirus (HCMV) infection [69, 70]. ~~may be related to the high virus resistance ability of *Paguma larvata*.~~There is no study focus on *PDGFRA* receptor in masked palm civets, further study is needed.

As an important receptor intermediate the infection of SARS-CoV and SARS-CoV-2, *ACE2* receptor was involved in this study though it is not under positive selection. The phylogeny of ~~*CD55, HSPG2,*~~ *PDGFRA*~~, SCARB2~~, and *ACE2* receptors from different animal species was conducted based on amino acid sequences downloaded from NCBI. The relationship of *PDGFRA*~~*these four receptor genes*~~ and *ACE2* ~~under positive selection~~ in ~~*Paguma*~~*P. larvata* was closest to four most closely related species from the Felidae family~~that~~ of *F. catus*, *P. pardus*, *L. canadensis*, and *P. tigris altaica* ~~*F. catus* and *Panthera tigris* in the four phylogenetic trees~~ (Figure 2). The amino acid sequence identities~~y~~ of ~~*CD55 was the highest between Paguma larvata and Panthera tigris, whereas HSPG2 and SCARB2 showed the highest identity between Paguma larvata and F. catus; moreover, amino acid sequence of*~~ *PDGFRA* and *ACE2* wa~~s~~ere ~~the~~ also highest among ~~*Paguma*~~*P. larvata*, *F. catus*, *P. pardus*, *L. canadensis*, and *P. tigris altaica*~~*F. catus*, and *Panthera tigris*~~ (Table 2)~~. ACE2 acted as a receptor for SARS and SARS-CoV-2, and we conducted phylogenetic analysis and sequence identity based on amino acid sequences of different animal species. ACE2 of *Paguma larvata* was closely related to *F. catus* and *Panthera tigris* (Table 2). Amino acid sequence of CD55, HSPG2, PDGFRA, SCARB2, and ACE2 between *Paguma larvata* and human beings were 35.09%, 89.20%, 94.92%, 83.91%, and 81.79%, respectively~~, demonstrating~~.~~ viruses using *PDGFRA* and *ACE2* might easily transferred between these species. However, ~~F~~further research is needed to reveal the differences in the receptor-binding loci of these ~~four~~ receptors in ~~*Paguma*~~*P. larvata* and ~~humans~~other species, as well as the immunoreaction of cells after virus infection in consideration of different severity of symptoms in different species.

Besides virus receptors, other ten immun~~oglobuline~~-related genes were also under selection (Supplementary Table S10). Further studies are needed to reveal the roles of these immune-related genes under positive selection in civet immunity. ~~取比较重要~~

Formatted: Font: Italic
Formatted: Font: Italic
Formatted: Font: Not Italic
Formatted: Font: Italic
Formatted: Font: Italic
Formatted: Font: Italic
Formatted: Font: Italic
Formatted: Font: Italic
Formatted: Font: Italic
Formatted: Font: Italic
Formatted: Font: Italic
Formatted: Font: Italic
Formatted: Font: Italic
Formatted: Font: Italic
Formatted: Font: Italic
Formatted: Font: Italic
Formatted: Font: Italic
Formatted: Font: Italic

的写几句，然后就收一下说具体的需要后期深入研究。

Conclusion

In this study, we present a high-quality chromosome-level genome assembly of *Paguma*P. *larvata* using PacBio single-molecule long-read sequencing, supplemented with Illumina short-read sequencing and Hi-C sequencing. The final genome assembly was 2.44 Gb, 95.32% of which could be grouped into 22 pseudochromosomes. This assembly is highly contiguous with contig N50 and scaffold N50 values of 12.97 Mb and 111.87 Mb. The repeat sequence comprised 31.80% of the genome. A total of 21,582 protein-coding genes were predicted, 95.20% of which could be functionally annotated. Phylogenetic analysis based on 1,4385,417 single-copy orthogroups across 119 animals resulted in a close genetic relationship between *Paguma*P. *larvata*, *Panthera tigris*, and five species from the Felidae family*F. Catus*. The divergence time of *Paguma*P. *larvata* split from the Felidae family*Panthera tigris* and *F. Catus* was estimated to be 37.23.5 Ma. Gene family clustering revealed 87119 unique, , 312243 significantly expanded, and 139 58 significantly contracted gene families in the *Paguma*P. *larvata* genome. We identified 285971 genes under positive selection in *Paguma*P. *larvata*, among which four one known human viral receptor genes were found. The high-quality genome assembly will provide a valuable genomic resource for elucidating the immune mechanisms in this animal. It will also provide a reliable reference for studying the genetic bases of the morphological characteristics, adaptive evolution, and evolutionary history of this species.

Data Availability

Raw sequencing data and genome assembly were deposited at the NCBI under the BioProject PRJNA756156 with biosample accession number SAMN20856571. The annotations and predicted peptides are available in FigShare at the link: https://doi.org/10.6084/m9.figshare.15273879.

Competing Interests

The authors declare that they have no competing interests.

Funding

This project was supported by the surveillance of wild animals and epidemic diseases in important ecological areas of Guangdong Province project from the Forestry Administration of Guangdong Province, GDAS Special Project of Science and Technology Development (2020GDASYL-20200103090).

Acknowledgements

The sequencing in this study was conducted by the Novogene Company (Beijing, China). Thanks for the hard work of Hao Lv and her colleagues. Thanks for the helpful advices of Chen Feng and Chao Feng from South China Botanical Garden, Chinese Academy of Sciences in data analysis.

Formatted: Font: Bold

Figure Legends

Figure 1 (a) Synteny of corresponding chromosomes and gene density across the *Paguma*~~*Paguma*~~ *larvata* genome; (b) Comparison of copy numbers in gene clusters reside in the genomes of ~~*Paguma*~~*P.* *larvata* and other ~~ten~~18 animals. Single-copy orthologs denote the family can have only one gene for each species, and multicopy orthologs denote the family clustered more than one gene for each species. Other orthologs denote the family can have any number of genes for each species except the single-copy and multi-copy orthologs. Unique paralogs denote species-specific gene families, and unclustered genes denote species-specific genes that cannot cluster with any other genes; (c) Phylogenetic relationships of 1~~1~~9 animal genomes based on the maximum likelihood method. The black numbers indicate estimated divergent times as the number of years (millions) ago, while green and red numbers denote gene families subject to expansion and contraction for each species, respectively.

Figure 2 Phylogenetic relationships of ~~four~~ known human virus receptor~~s~~ of *PDGFRA* under positive selection and *ACE2* receptor in ~~*Paguma*~~*P.* *larvata* and other species.

Formatted: Font: Italic

Formatted: Font: Italic

Formatted: Font: Not Italic

References

1. Iwami S, Takeuchi Y, Liu X. Avian flu pandemic: Can we prevent it?. J Thero Biol 2009;**257**(1):181-190.
2. Siddiqui AA. The global threat of bird (Avian) flu its treatment methods and public health preventive measures. SOJ Vet Sci 2018; **4**(3):1-4.
3. Dixon L K, Sun H, Roberts H. African swine fever. Antivir Res 2019;**165**:34-41.
4. Rota PA, Oberste MS, Monroe SS, et al. Characterization of a novel coronavirus associated with severe acute respiratory syndrome. Science 2003;**300**(5624):1394-1399.
5. Zumla A, Hui D S, Perlman S. Middle East respiratory syndrome. The Lancet 2015;**386**(9997):995-1007.
6. Wu D, Wu T, Liu Q, et al. The SARS-CoV-2 outbreak: what we know. Int J Infect Dis 2020;**94**:44-48.
7. Sabir JSM, Lam TTY, Ahmed MMM, et al. Co-circulation of three camel coronavirus species and recombination of MERS-CoVs in Saudi Arabia. Science 2016;**351**(6268):81-84.
8. Inoue T, Kaneko Y, Yamazaki K, et al. Genetic population structure of the masked palm civet *Paguma larvata*,(Carnivora: Viverridae) in Japan, revealed from analysis of newly identified compound microsatellites. Conserv Genet 2012;**13**(4):1095-1107.
9. Guan Y, Zheng BJ, He YQ, et al. Isolation and characterization of viruses related to the SARS coronavirus from animals in southern China. Science 2003;**302**(5643):276-278.
10. Li W, Shi Z, Yu M, et al. Bats are natural reservoirs of SARS-like coronaviruses. Science 2005;**310**(5748):676-679.
11. Wang LF, Eaton BT. Bats, civets and the emergence of SARS. Wildlife and emerging zoonotic diseases: the biology, circumstances and consequences of cross-species transmission, 2007:325-344.
12. Wan Y, Shang J, Graham R, et al. Receptor recognition by the novel coronavirus from Wuhan: an analysis based on decade-long structural studies of SARS coronavirus. J Virol 2020;**94**(7):e00127-20.

13. Marsh M, Helenius A. Virus entry: open sesame. Cell 2006;124(4):729-740.
14. Grove J, Marsh M. The cell biology of receptor-mediated virus entry. J Cell Biol 2011;**195**(7):1071-1082.
15. Li W, Moore MJ, Vasilieva N, et al. Angiotensin-converting enzyme 2 is a functional receptor for the SARS coronavirus. Nature 2003;**426**(6965):450-454.
16. Shi R, Shan C, Duan X, et al. A human neutralizing antibody targets the receptor-binding site of SARS-CoV-2. Nature 2020;**584**(7819):120-124.
17. Wu Y, Wang F, Shen C, et al. A noncompeting pair of human neutralizing antibodies block COVID-19 virus binding to its receptor ACE2. Science 2020;**368**(6496):1274-1278.
18. Bickhart DM, Rosen BD, Koren S, et al. Single-molecule sequencing and chromatin conformation capture enable de novo reference assembly of the domestic goat genome. Nat genet, 2017;**49**(4):643-650.
19. Mahajan S, Wei K, Nalley M, et al. De novo assembly of a young Drosophila Y chromosome using Single-Molecule sequencing and Chromatin Conformation capture. PLoS Biol 2018;**16**:1-28.
20. Zhang Z, Chen Y, Zhang J, et al. Improved genome assembly provides new insights into genome evolution in a desert poplar (*Populus euphratica*). Mol Ecol Resour 2020;**20**(3):781-794.
21. Rio DC, Ares M, Hannon GJ, et al. Purification of RNA using TRIzol (TRI reagent). Cold Spring Harbor Protocols, 2010;**2010**(6):pdb. prot5439.
22. Chin CS, Alexander DH, Marks P, et al. Nonhybrid, finished microbial genome assemblies from long-read SMRT sequencing data. Nat methods 2013;**10**(6):563-569.
23. Belton JM, McCord RP, Gibcus JH, et al. Hi – C: a comprehensive technique to capture the conformation of genomes. Methods 2012;**58**(3):268-276.
24. Chin CS, Peluso P, Sedlazeck FJ, et al. Phased diploid genome assembly with single-molecule real-time sequencing. Nat methods 2016;**13**(12):1050-1054.
25. Walker BJ, Abeel T, Shea T, et al. Pilon: an integrated tool for comprehensive microbial variant detection and genome assembly improvement. PloS one 2014;9(11):e112963.

26. Li H, Durbin R. Fast and accurate short read alignment with Burrows-Wheeler transform. Bioinformatics 2009;**25**:175-1760.

27. Hariharan R, Toyama K. Project Lachesis: parsing and modeling location histories//International Conference on Geographic Information Science. Springer, Berlin, Heidelberg, 2004:106-124.

28. Burton JN, Adey A, Patwardhan RP, et al. Chromosome-scale scaffolding of de novo genome assemblies based on chromatin interactions. Nat Biotechnol 2013;**31**:1119-1125.

29. Liu B, Shi Y, Yuan J, et al. Estimation of genomic characteristics by analyzing k-mer frequency in de novo genome projects. 2013. http://arxiv.org/abs/1308.2012.

30. Manni M, Berkeley MR, Seppey M, et al. BUSCO update: novel and streamlined workflows along with broader and deeper phylogenetic coverage for scoring of eukaryotic, prokaryotic, and viral genomes. Mol Biol Evol 2021;**38**(10): 4647-s4654

31. Parra G, Bradnam K, Korf I. CEGMA: A pipeline to accurately annotate core genes in eukaryotic genomes. Bioinformatics 2007;**23**:1061-1067.

32. Grabherr MG, Haas BJ, Yassour M, et al. Full-length transcriptome assembly from RNA-Seq data without a reference genome. Nat Biotechnol 2011;**29**:644-652.

33. Kent WJ. BLAT—the BLAST-Like Alignment Tool. Genome Res 2002;12:656-664.

34. Trapnell C, Roberts A, Goff L, et al. Differential gene and transcript expression analysis of RNA-seq experiments with TopHat and Cufflinks. Nat Protoc 2012;**7**:562-578.

35. Bao W, Kojima KK, Kohany O. Repbase Update, a database of repetitive elements in eukaryotic genomes. Mob DNA 2015;**6**:4-9.

36. Benson G. Tandem repeats finder: A program to analyze DNA sequences. Nucleic Acids Res 1999;**27**:573-580.

37. Stanke M, Schöffmann O, Morgenstern B, et al. Gene prediction in eukaryotes with a generalized hidden Markov model that uses hints from external sources. BMC Bioinformatics 2006;**7**:1-11.

38. Majoros WH, Pertea M, Salzberg SL. TigrScan and GlimmerHMM: Two open

source ab initio eukaryotic gene-finders. Bioinformatics 2004;**20**:2878-2879.

39. Korf I. Gene finding in novel genomes. BMC Bioinformatics 2004;**5**:1-9.

40. Burge C, Karlin S. Prediction of complete gene structures in human genomic DNA. J Mol Biol 1997;**268**:78-94.

41. Guigó R, Knudsen S, Drake N, et al. Prediction of gene structure. J Mol Biol 1992;**226**:141-157.

42. Gnerre S, MacCallum I, Przybylski D, et al. High-quality draft assemblies of mammalian genomes from massively parallel sequence data. Proc Natl Acad Sci USA 2011;**108**:1513-1518.

43. Kirkness EF, Bafna V, Halpern AL, et al. The dog genome: Survey sequencing and comparative analysis. Science 2003;**301**(5641):1898-1903.

44. Pontius JU, Mullikin JC, Smith DR, et al. Initial sequence and comparative analysis of the cat genome. Genome Res 2007;**17**(11):1675-1689.

45. Cho Y S, Hu L, Hou H, et al. The tiger genome and comparative analysis with lion and snow leopard genomes. Nat Commun 2013;**4**(1):1-7.

46. Dobrynin P, Liu S, Tamazian G, et al. Genomic legacy of the African cheetah, *Acinonyx jubatus*. Genome Biol 2015;**16**(1):1-20.

47. Altschul SF, Gish W, Miller W, et al. Basic local alignment search tool. J Mol Biol 1990;**215**:403-410.

48. Birney E, Clamp M, Durbin R. GeneWise and Genomewise. Genome Res 2004;**14**:988-995.

49. Kim D, Pertea G, Trapnell C, et al. TopHat2: accurate alignment of transcriptomes in the presence of insertions, deletions and gene fusions. Genome biol 2013;**14**(4):1-13.

50. Haas BJ, Delcher AL, Mount SM, et al. Improving the Arabidopsis genome annotation using maximal transcript alignment assemblies. Nucleic Acids Res 2003;**31**:5654-5666.

51. Haas BJ, Salzberg SL, Zhu W, et al. Automated eukaryotic gene structure annotation using EVidenceModeler and the Program to Assemble Spliced Alignments. Genome biol 2008;**9**(1):1-22.

52. Kanehisa M, Sato Y, Kawashima M, et al. KEGG as a reference resource for gene

and protein annotation. Nucleic Acids Res 2016;**44**:457-462.

53. Quevillon E, Silventoinen V, Pillai S, et al. InterProScan: protein domains identifier. Nucleic Acids Res 2005;**33**:116-120.
54. Finn RD, Coggill P, Eberhardt RY, et al. The Pfam protein families database: towards a more sustainable future. Nucleic Acids Res 2016;**44**(D1):D279-D285.
55. Emms DM, Kelly S. OrthoFinder: Solving fundamental biases in whole genomecomparisons dramatically improves orthogroup inference accuracy. Genome Biol 2015;**16**: 157.
56. Edgar RC. MUSCLE: Multiple sequence alignment with high accuracy and high throughput. Nucleic Acids Res 2004;**32**:1792-1797.
57. Nguyen LT, Schmidt HA, Von Haeseler A, et al. IQ-TREE: a fast and effective stochastic algorithm for estimating maximum-likelihood phylogenies. Mol Biol Evol 2015;**32**(1):268-274.
58. Bouckaert R, Heled J, Kühnert D, et al. BEAST 2: a software platform for Bayesian evolutionary analysis. PLoS comput biol, 2014;**10**(4):e1003537.
59. De Bie T, Cristianini N, Demuth JP, et al. CAFE: A computational tool for the study of gene family evolution. Bioinformatics 2006;**22**:1269-1271.
60. Yang Z. PAML 4: Phylogenetic analysis by maximum likelihood. Mol Biol Evol 2007;**24**:1586-1591.
61. Ren L, Wu C, Guo L, et al. Single-cell transcriptional atlas of the Chinese horseshoe bat (*Rhinolophus sinicus*) provides insight into the cellular mechanisms which enable bats to be viral reservoirs. bioRxiv 2020. DOI:10.1101/2020.06.30.175778
62. Kumar S, Stecher G, Li M, et al. MEGA X: molecular evolutionary genetics analysis across computing platforms. Mol Biol Evol 2018;**35**(6):1547.
63. Fecteau R E, Kong J, Kresak A, et al. Association between germline mutation in VSIG10L and familial Barrett neoplasia. JAMA Oncol 2016;**2**(10):1333-1339.
64. Radjabova V, Mastroeni P, Skjødt K, et al. TARM1 Is a Novel Leukocyte Receptor Complex – Encoded ITAM Receptor That Costimulates Proinflammatory Cytokine Secretion by Macrophages and Neutrophils. J Immunol 2015;**195**(7):3149-3159.

Formatted: Font: (Default) Times New Roman, 12 pt

Formatted: Font: (Default) Times New Roman, 12 pt

Formatted: Font: (Default) Times New Roman, 12 pt

Formatted: Font: (Default) Times New Roman, 12 pt, Bold

Formatted: Font: (Default) Times New Roman, 12 pt

Formatted: Font: (Default) Times New Roman, 12 pt

Formatted: Font: (Default) Times New Roman, 12 pt

Formatted: Font: (Default) Times New Roman, 12 pt, Bold

Formatted: Font: (Default) Times New Roman, 12 pt

65. Gemayel KT, Litman GW, Sriaroon P. Autosomal recessive agammaglobulinemia associated with an IGLL1 gene missense mutation. Ann Allerg, Asthma Im 2016;**117**(4):439-441.

66. Kim N, Takami M, Rho J, et al. A novel member of the leukocyte receptor complex regulates osteoclast differentiation. J Exp Med 2002;**195**(2):201-209.

67. Wang D, Yang N, Xie S. Sex‐biased ceRNA networks reveal that OSCAR can promote proliferation and migration of lung adenocarcinoma in women. Clin Exp Pharmacol P 2020;**47**(8):1350-1359.

68. Heinrich M C, Corless C L, Duensing A, et al. PDGFRA activating mutations in gastrointestinal stromal tumors. Science 2003;**299**(5607):708-710.

69. Soroceanu L, Akhavan A, Cobbs CS. Platelet-derived growth factor-α receptor activation is required for human cytomegalovirus infection. Nature 2008;**455**(7211):391-395.

70. Yang Z, Tang X, McMullen T P W, et al. PDGFRα Enhanced Infection of Breast Cancer Cells with Human Cytomegalovirus but Infection of Fibroblasts Increased Prometastatic Inflammation Involving Lysophosphatidate Signaling. Int J Mol Sci 2021;**22**(18): 9817.

1. Iwami S, Takeuchi Y, Liu X. Avian flu pandemic: Can we prevent it?. J Thero Biol 2009;**257**(1):181-190.

2. Siddiqui AA. The global threat of bird (Avian) flu its treatment methods and public health preventive measures. SOJ Vet Sci 2018; **4**(3):1-4.

3. Dixon L K, Sun H, Roberts H. African swine fever. Antivir Res 2019;**165**:34-41.

4. Rota PA, Oberste MS, Monroe SS, et al. Characterization of a novel coronavirus associated with severe acute respiratory syndrome. Science 2003;**300**(5624):1394-1399.

5. Zumla A, Hui D S, Perlman S. Middle East respiratory syndrome. The Lancet 2015;**386**(9997):995-1007.

6. Wu D, Wu T, Liu Q, et al. The SARS-CoV-2 outbreak: what we know. Int J Infect Dis 2020;**94**:44-48.

7. Sabir JSM, Lam TTY, Ahmed MMM, et al. Co-circulation of three camel coronavirus species and recombination of MERS-CoVs in Saudi Arabia. Science

2016;**351**(6268):81-84.

8. Inoue T, Kaneko Y, Yamazaki K, et al. Genetic population structure of the masked palm civet *Paguma larvata*,(Carnivora: Viverridae) in Japan, revealed from analysis of newly identified compound microsatellites. Conserv Genet 2012;**13**(4):1095-1107.
9. Guan Y, Zheng BJ, He YQ, et al. Isolation and characterization of viruses related to the SARS coronavirus from animals in southern China. Science 2003;**302**(5643):276-278.
10. Li W, Shi Z, Yu M, et al. Bats are natural reservoirs of SARS-like coronaviruses. Science 2005;**310**(5748):676-679.
11. Wang LF, Eaton BT. Bats, civets and the emergence of SARS. Wildlife and emerging zoonotic diseases: the biology, circumstances and consequences of cross-species transmission, 2007:325-344.
12. Wan Y, Shang J, Graham R, et al. Receptor recognition by the novel coronavirus from Wuhan: an analysis based on decade-long structural studies of SARS coronavirus. J Virol 2020;**94**(7):e00127-20.
13. Marsh M, Helenius A. Virus entry: open sesame. Cell 2006;124(4):729-740.
14. Grove J, Marsh M. The cell biology of receptor-mediated virus entry. J Cell Biol 2011;**195**(7):1071-1082.
15. Li W, Moore MJ, Vasilieva N, et al. Angiotensin-converting enzyme 2 is a functional receptor for the SARS coronavirus. Nature 2003;**426**(6965):450-454.
16. Shi R, Shan C, Duan X, et al. A human neutralizing antibody targets the receptor-binding site of SARS-CoV-2. Nature 2020;**584**(7819):120-124.
17. Wu Y, Wang F, Shen C, et al. A noncompeting pair of human neutralizing antibodies block COVID-19 virus binding to its receptor ACE2. Science 2020;**368**(6496):1274-1278.
18. Bickhart DM, Rosen BD, Koren S, et al. Single-molecule sequencing and chromatin conformation capture enable de novo reference assembly of the domestic goat genome. Nat genet, 2017;**49**(4):643-650.
19. Mahajan S, Wei K, Nalley M, et al. De novo assembly of a young Drosophila Y chromosome using Single-Molecule sequencing and Chromatin Conformation

capture. PLoS Biol 2018;**16**:1-28.

20. Zhang Z, Chen Y, Zhang J, et al. Improved genome assembly provides new insights into genome evolution in a desert poplar (*Populus euphratica*). Mol Ecol Resour 2020;**20**(3):781-794.
21. Rio DC, Ares M, Hannon GJ, et al. Purification of RNA using TRIzol (TRI reagent). Cold Spring Harbor Protocols, 2010;**2010**(6):pdb. prot5439.
22. Chin CS, Alexander DH, Marks P, et al. Nonhybrid, finished microbial genome assemblies from long-read SMRT sequencing data. Nat methods 2013;**10**(6):563-569.
23. Belton JM, McCord RP, Gibcus JH, et al. Hi – C: a comprehensive technique to capture the conformation of genomes. Methods 2012;**58**(3):268-276.
24. Chin CS, Peluso P, Sedlazeck FJ, et al. Phased diploid genome assembly with single-molecule real-time sequencing. Nat methods 2016;**13**(12):1050-1054.
25. Walker BJ, Abeel T, Shea T, et al. Pilon: an integrated tool for comprehensive microbial variant detection and genome assembly improvement. PloS one 2014;9(11):e112963.
26. Li H, Durbin R. Fast and accurate short read alignment with Burrows-Wheeler transform. Bioinformatics 2009;**25**:175-1760.
27. Hariharan R, Toyama K. Project Lachesis: parsing and modeling location histories//International Conference on Geographic Information Science. Springer, Berlin, Heidelberg, 2004:106-124.
28. Burton JN, Adey A, Patwardhan RP, et al. Chromosome-scale scaffolding of de novo genome assemblies based on chromatin interactions. Nat Biotechnol 2013;**31**:1119-1125.
29. Liu B, Shi Y, Yuan J, et al. Estimation of genomic characteristics by analyzing k-mer frequency in de novo genome projects. 2013. http://arxiv.org/abs/1308.2012.
30. Manni M, Berkeley MR, Seppey M, et al. BUSCO update: novel and streamlined workflows along with broader and deeper phylogenetic coverage for scoring of eukaryotic, prokaryotic, and viral genomes. Mol Biol Evol 2021;**38**(10):-4647-s4654
31. Parra G, Bradnam K, Korf I. CEGMA: A pipeline to accurately annotate core genes

in eukaryotic genomes. Bioinformatics 2007;**23**:1061-1067.

32. Grabherr MG, Haas BJ, Yassour M, et al. Full-length transcriptome assembly from RNA-Seq data without a reference genome. Nat Biotechnol 2011;**29**:644-652.

33. Kent WJ. BLAT—the BLAST-Like Alignment Tool. Genome Res 2002;12:656-664.

34. Trapnell C, Roberts A, Goff L, et al. Differential gene and transcript expression analysis of RNA-seq experiments with TopHat and Cufflinks. Nat Protoc 2012;**7**:562-578.

35. Bao W, Kojima KK, Kohany O. Repbase Update, a database of repetitive elements in eukaryotic genomes. Mob DNA 2015;**6**:4-9.

36. Benson G. Tandem repeats finder: A program to analyze DNA sequences. Nucleic Acids Res 1999;**27**:573-580.

37. Stanke M, Schöffmann O, Morgenstern B, et al. Gene prediction in eukaryotes with a generalized hidden Markov model that uses hints from external sources. BMC Bioinformatics 2006;**7**:1-11.

38. Majoros WH, Pertea M, Salzberg SL. TigrScan and GlimmerHMM: Two open source ab initio eukaryotic gene-finders. Bioinformatics 2004;**20**:2878-2879.

39. Korf I. Gene finding in novel genomes. BMC Bioinformatics 2004;**5**:1-9.

40. Burge C, Karlin S. Prediction of complete gene structures in human genomic DNA. J Mol Biol 1997;**268**:78-94.

41. Guigó R, Knudsen S, Drake N, et al. Prediction of gene structure. J Mol Biol 1992;**226**:141-157.

42. Gnerre S, MacCallum I, Przybylski D, et al. High-quality draft assemblies of mammalian genomes from massively parallel sequence data. Proc Natl Acad Sci USA 2011;**108**:1513-1518.

43. Kirkness EF, Bafna V, Halpern AL, et al. The dog genome: Survey sequencing and comparative analysis. Science 2003;**301**(5641):1898-1903.

44. Pontius JU, Mullikin JC, Smith DR, et al. Initial sequence and comparative analysis of the cat genome. Genome Res 2007;**17**(11):1675-1689.

45. Cho Y S, Hu L, Hou H, et al. The tiger genome and comparative analysis with lion and snow leopard genomes. Nat Commun 2013;**4**(1):1-7.

46. Dobrynin P, Liu S, Tamazian G, et al. Genomic legacy of the African cheetah, *Acinonyx jubatus*. Genome Biol 2015;**16**(1):1-20.

47. Altschul SF, Gish W, Miller W, et al. Basic local alignment search tool. J Mol Biol 1990;**215**:403-410.

48. Birney E, Clamp M, Durbin R. GeneWise and Genomewise. Genome Res 2004;**14**:988-995.

49. Kim D, Pertea G, Trapnell C, et al. TopHat2: accurate alignment of transcriptomes in the presence of insertions, deletions and gene fusions. Genome biol 2013;**14**(4):1-13.

50. Haas BJ, Delcher AL, Mount SM, et al. Improving the Arabidopsis genome annotation using maximal transcript alignment assemblies. Nucleic Acids Res 2003;**31**:5654-5666.

51. Haas BJ, Salzberg SL, Zhu W, et al. Automated eukaryotic gene structure annotation using EVidenceModeler and the Program to Assemble Spliced Alignments. Genome biol 2008;**9**(1):1-22.

52. Kanehisa M, Sato Y, Kawashima M, et al. KEGG as a reference resource for gene and protein annotation. Nucleic Acids Res 2016;**44**:457-462.

53. Quevillon E, Silventoinen V, Pillai S, et al. InterProScan: protein domains identifier. Nucleic Acids Res 2005;**33**:116-120.

54. Li H, Coghlan A, Ruan J, et al. TreeFam: a curated database of phylogenetic trees of animal gene families. Nucleic Acids Res 2006;**34**:572-580.

55. Edgar RC. MUSCLE: Multiple sequence alignment with high accuracy and high throughput. Nucleic Acids Res 2004;**32**:1792-1797.

56. Stamatakis A. RAxML version 8: A tool for phylogenetic analysis and post-analysis of large phylogenies. Bioinformatics 2014;**30**:1312-1313.

57. Yang Z. PAML 4: Phylogenetic analysis by maximum likelihood. Mol Biol Evol 2007;**24**:1586-1591.

58. De Bie T, Cristianini N, Demuth JP, et al. CAFE: A computational tool for the study of gene family evolution. Bioinformatics 2006;**22**:1269-1271.

59. Zhang J, Nielsen R, Yang Z. Evaluation of an improved branch-site likelihood method for detecting positive selection at the molecular level. Mol Biol Evol

2005;**22**(12):2472-2479.

60. Katoh K, Standley DM. MAFFT multiple sequence alignment software version 7: improvements in performance and usability. Mol Biol Evol 2013;**30**(4):772-780.

61. Nguyen LT, Schmidt HA, Von Haeseler A, et al. IQ-TREE: a fast and effective stochastic algorithm for estimating maximum-likelihood phylogenies. Mol Biol Evol 2015;**32**(1):268-274.

62. Kumar S, Stecher G, Li M, et al. MEGA X: molecular evolutionary genetics analysis across computing platforms. Mol Biol Evol 2018;**35**(6):1547.

63. Ren L, Wu C, Guo L, et al. Single-cell transcriptional atlas of the Chinese horseshoe bat (*Rhinolophus sinicus*) provides insight into the cellular mechanisms which enable bats to be viral reservoirs. bioRxiv 2020. DOI:10.1101/2020.06.30.175778

Formatted: Line spacing: Exactly 22 pt, Numbered + Level: 1 + Numbering Style: 1, 2, 3, … + Start at: 1 + Alignment: Left + Aligned at: 0" + Indent at: 0.3"

Tables

Table 1 *Paguma larvata* Genome Assembly and Annotation Summary.

Genome assembly statistics	
Total length (bp)	2,437,385,791
Contig/Scaffold N50 length (bp)	12,971,789
Contig/Scaffold N90 length (bp)	2,389,527
Longest contig length (bp)	52,858,878
Number of contigs	1,785
Number of contigs larger than N50	56
Number of contigs larger than N90	210
Genome characteristics	
GC content	42.41%
Number of protein-coding genes	21,582
Average transcript length (bp)	34,567.54
Average CDS length (bp)	1,481.00
Average exon length (bp)	170.53
Average exons per gene (bp)	8.68
Repetitive sequences (% of genome)	
DNA (bp)	8,232,288 (0.34%)
LINE (bp)	299,126,376 (12.27%)
SINE (bp)	613,247 (0.03%)
LTR (bp)	542,789,387 (22.27%)
Unknown (bp)	12,280,567 (0.5%)
Total (bp)	775,001,321 (31.8%)
Gene annotations (% of all genes)	
GO	14,148 (65.6%)
InterProScan	19,770 (91.6%)
KEGG	18,200 (84.3%)
Nr	20,480 (94.9%)
PFAM	17,505 (81.1%)
SwissProt	20,033 (92.8%)
Total	21,582

Table 2 Sequence identity of receptors under positive selection between masked palm civet and other animal species.

Species	Sequence identity (%)	
	PDGFRA	ACE2
Felis catus	**97.83**	**92.39**
Panthera tigris	**97.83**	**92.31**
Ursus maritimus	95.90	89.39
Ailuropoda melanoleuca	95.12	89.23
Canis lupus	96.48	88.27
Sus scrofa	94.73	80.74
Manis javanica	93.32	84.29
Desmodus rotundus	91.83	76.87
Homo sapiens	94.92	81.79
Bos mutus	92.84	79.96
Mus musculus	89.47	79.84
Gallus gallus	77.52	60.88

**Species**	**Sequence identity (%)**	
	PDGFRA	ACE2
Lynx canadensis	**97.95**	**93.17**
Felis catus	**97.85**	**92.67**
Panthera tigris altaica	**97.85**	**92.60**
Panthera pardus	**97.67**	**92.80**
Canis lupus familiaris	96.55	88.93
Vulpes vulpes	96.55	88.81
Ursus maritimus	95.99	89.94
Ailuropoda melanoleuca	95.24	89.79
Homo sapiens	95.05	83.35
Sus scrofa	94.86	82.48
Manis javanica	93.54	85.47
Bos taurus	93.18	81.84
Bos mutus	93.09	81.84
Desmodus rotundus	92.16	79.35
Mus musculus	90.01	81.74
Oryctolagus cuniculus	88.14	83.11
Monodelphis domestica	83.49	71.14

Clear version

Click here to access/download;Manuscript;paper-20220211-clear.docx

The Assembled and Annotated Genome of the Masked Palm Civet (*Paguma larvata*)

Ping Liu[1], Hai-Ying Jiang[1], Lin-Miao Li[1], Jia-Bin Zhou[1], Wen-Zhong Huang[1], Jin-Ping Chen[1]

[1]Guangdong Key Laboratory of Animal Conservation and Resource Utilization, Guangdong Public Laboratory of Wild Animal Conservation and Utilization, Institute of Zoology, Guangdong Academy of Sciences, Guangzhou 510260, China; pingliu0330@126.com

Corresponding author: Jin-Ping Chen, Institute of Zoology, Guangdong Academy of Sciences, Guangzhou 510260, China. chenjp@giz.gd.cn; Tel.: +020-8910-0920

Abstract

Background: The masked palm civet (*Paguma larvata*) acts as an intermediate host of severe acute respiratory syndrome coronavirus (SARS-CoV), which caused SARS, and transfers this virus from bats to humans. Additionally, *P. larvata* has the potential to carry a variety of zoonotic viruses that may threaten human health. However, genome resources for *P. larvata* have not been reported to date. **Findings:** A chromosome-level genome assembly of *P. larvata* was generated using PacBio sequencing, Illumina sequencing, and Hi-C technology. The genome assembly was 2.44 Gb in size, of which 95.32% could be grouped into 22 pseudochromosomes, with contig N50 and scaffold N50 values of 12.97 Mb and 111.87 Mb, respectively. A total of 21,582 protein-coding genes were predicted, and 95.20% of the predicted genes were functionally annotated. Phylogenetic analysis of 19 animal species confirmed the close genetic relationship between *P. larvata* and species belong to the Felidae family. Gene family clustering revealed 119 unique, 362 significantly expanded, and 58 significantly contracted genes in the *P. larvata* genome. We identified 971 positively selected genes in *P. larvata*, and one known human viral receptor gene *PDGFRA* is positively selected

in *P. larvata*, which is required for human cytomegalovirus (HCMV) infection, **Conclusions:** This high-quality genome assembly provides a valuable genomic resource for exploring virus-host interactions . It will also provide a reliable reference for studying the genetic bases of the morphological characteristics, adaptive evolution, and evolutionary history of this species.

Keywords: Masked palm civet, genome assembly, Hi-C proximity mapping, gene family evolution, phylogeny, positive selection

Introduction

In human history, the spread of viruses has caused huge losses to the human economy and threatened the safety of human life and property. Diseases affecting animals of economic value, caused by viruses such as avian influenza and African swine fever, have caused serious economic losses [1-3]. Viruses such as severe acute respiratory syndrome coronavirus (SARS-CoV), Middle East respiratory syndrome coronavirus (MERS-CoV), and SARS-CoV-2, pose a great threat to the safety of human life and property [4-6]. Usually, zoonotic viruses can be transmitted to humans via wildlife as intermediate hosts. For example, MERS-CoV is transmitted from camels to humans [7].

Masked palm civets, *Paguma larvata* (Mammalia, Carnivora, and Viverridae) are wild mammals widely distributed in Asia [8]. They are omnivores, although they mainly consume fruits. In recent decades, *P. larvata* used to be domesticated as meat of human beings, increasing the possibility of transferring pathogenic microorganisms to human beings. In 2003, *P. larvata* gained attention because of its potential involvement in the outbreak of severe acute respiratory syndrome (SARS), which originated in southern China and spread to over 30 countries [9]. Further research suggested that *P. larvata* acted as an intermediate host of SARS-CoV, which transmits this virus from bats to humans [10, 11]. The sequence similarity between the SARS-CoV found in *P. larvata* and human SARS-CoV is greater than 99%. However, *P. larvata* can live in harmony with SARS-CoV, whereas humans can have severe symptoms following SARS-CoV infection. This shows that the interaction between *P. larvata* and viruses

has a certain particularity [12]. Earlier studies on farms raising *P. larvata* also found that these animals rarely become ill. The immune system of *P. larvata* is thus worthy of in-depth study.

Virus receptors, present on the host cell membrane, are important factors in mediating virus invasion and play an extremely important role in the interaction between host cells and viruses. The virus attachment protein can be regarded as the "key" that unlocks the cell by interacting with the "lock" (receptor) on the surface of the host cell, starting a key downstream step in the virus life cycle. In many cases, viruses use multiple receptors to perform different functions during the viral life cycle [13]. Therefore, the virus must first find the lock and then use a specific virus "key" to unlock the cell. The numerous functions of the virus receptor aid the virus in targeting the correct tissue for infection and to cross the cell barrier, which is necessary to deliver the viral genome to the host cell [14]. The interaction with viral receptors is usually mediated by specific proteins on the surface of the virus [13]. Inherent differences in the shape of the virus surface protein (icosahedron or helix) and the composition of the virus coat affect the overall structure of the virus attachment protein. Some viruses infect only certain species; for example, swine fever virus can only infect pigs [3]. Whereas some viruses can spread between animals and humans, such as SARS-CoV [10, 11] and MERS-CoV [7]; the reason lies in the nature of the interaction between the virus receptor and virus attachment protein. For example, SARS-CoV and SARS-CoV-2 specifically recognize the angiotensin-converting enzyme 2 (*ACE2*) receptor [15, 16, 17]. Understanding the nature of virus receptors that cause zoonotic diseases in different species will help in tracing natural hosts during the virus epidemic.

As an important virus host, masked palm civets can be used as a system for reviewing virus and host interactions. However, there is currently no genome assembly of *P. larvata*, even in Viverridae which has been reported for studying various virus receptors. In this study, we generated a chromosome-level reference genome of *P. larvata* by integrating PacBio sequencing, Hi-C sequencing, and Illumina sequencing [18-20]. The high-quality genome assembly will provide a valuable genomic resource for exploring virus-host interactions . It will also provide a reliable reference for studying the genetic bases of the morphological characteristics, adaption evolution, and

evolutionary history of the species.

Data Description

Statement of Ethics

The study design was approved by the Ethics Committee for Animal Experiments at the Institute of Zoology, Guangdong Academy of Sciences (reference number: GIZ20190702, 07/01/2019) and followed the basic principles outlined by this committee.

Isolation of genomic DNA and RNA

A healthy female *P. larvata* individual was collected from a farm in Wengyuan County, Guangdong Province, China. Whole blood was isolated from the same masked palm civet and washed with saline nasal spray, then immediately frozen in liquid nitrogen and stored at -80 °C prior to nucleic acid extraction. Genomic DNA was extracted from whole blood using a magnetic universal genomic DNA kit. Different tissues, including heart, intestine, kidney, liver, lung, muscle, and spleen were sampled from the same one female *P. larvata* individual and flash-frozen in liquid nitrogen for RNA sequencing, RNA-seq data was used to predict genes in following analysis. Total RNA was extracted using the TRIzol reagent [21].

Genome sequencing with different technologies

A combined sequencing strategy was used to obtain the masked palm civet genome. Short-insert libraries with a size of 350 bp were sequenced on the Illumina HiSeq 2500 platform using the 150 bp paired-end protocol. For PacBio library construction, we sheared the genomic DNA to 20 kb fragments, which we used to prepare a single-molecule real-time (SMRT) library for sequencing with the PacBio Sequel system [22]. Liver tissue was used to construct the Hi-C sequencing library. Briefly, cross-linked chromatin was digested with Dpn II and ligated in situ after biotinylation. DNA fragments were enriched via the interaction of biotin and blunt-end ligation and then subjected to Illumina NovaSeq PE 150 sequencing [23].

Genome assembly and quality assessment

We applied the FALCON v0.3.0 [24] to correct errors in PacBio long reads, according to PacBio short reads (<5 kb) and then generated consensus sequences. The primary scaffolds were assembled with these subreads, and the scaffolds were further corrected in PILON v1.22 [25] using short sequencing reads. To anchor scaffolds onto chromosomes, we aligned the Hi-C sequencing data to the assembly using BWA v0.7.17 [26] and detected valid contacts. Preassembled scaffolds were clustered, ordered, and directed onto pseudochromosomes using the LACHESIS software [27]. To increase the accuracy of the assembled genome, we artificially corrected the LACHESIS-based assembly, filled gaps, and removed duplicate sequences [28].

Genome size was estimated based on k-mer distribution analysis with the program in GCE [29] using Illumina short reads. We used Benchmarking Universal Single-Copy Orthologs (BUSCO v5) [30] and Core Eukaryotic Genes Mapping Approach (CEGMA) [31] to evaluate the completeness and accuracy of the genome assembly and BWA v0.7.17 [26] to align the Illumina short reads to the assembly and evaluate the assembled portion. We assembled expressed sequencing tags (ESTs) using Trinity v2.8.4 [32] with RNA reads from seven different tissues (heart, liver, spleen, lung, kidney, muscle, and intestine). To further examine the quality of the genome assembly, we aligned ESTs to the assembled genome using BLAT [33] with parameters of identity ≥90% and coverage ≥50%. The quality of the genome assembly was evaluated by mapping RNA-Seq reads to the ESTs using TopHat2 [34].

Repetitive elements annotation

Repetitive sequences were identified at both the DNA and protein levels by integrating homology-based prediction and *de novo* identification. Repeat masking was performed based on repeats in the Repbase TE library from the Repbase server [35]. Using this library, we predicted interspersed repeat elements using RepeatMasker v3.3.0 and ProteinMask (http://www.repeatmasker.org) and screened tandem repeats using TRF v4.07b [36].

Gene prediction and functional annotation

We annotated protein-coding genes in the *P. larvata* genome using a combination of *de novo* gene prediction, homology-based prediction, and RNA-seq-based prediction. For *de novo* identification, we predicted the gene models using five *ab initio* gene prediction programs: Augustus [37], GlimmerHMM [38], SNAP [39], Genscan [40], and Geneid [41]. We compared proteins from five sequenced animals, namely, *Mus musculus* [42], *Canis lupus familiaris* [43], *Felis catus* [44], *Panthera tigris altaica* [45], and *Acinonyx jubatus* [46], to the *P. larvata* assembly using tBlastN [47] with an e-value cut-off of 1×10^{-5}. Gene models were generated using GeneWise v2.2.0 [48]. We applied two methods of RNA-Seq-based prediction: (i) we mapped RNA-Seq data to the genome using TopHat2 [49] and further generated the gene models in Cufflinks [34] based on exons, and (ii) we aligned ESTs against the assembly to generate the gene models using PASA [50]. We integrated predictions from the three approaches in EVidenceModeler v1.1.1 [51] to generate a non-redundant gene model set. The final gene models were annotated using the non-redundant protein database of NCBI (NR) and Swissprot, Kyoto Encyclopedia of Genes and Genomes (KEGG) [52], Interprot [53], and Pfam databases v14.1 [54].

Noncoding RNAs include tRNA, rRNA, miRNA, and snRNA. tRNAscan-SE v1.4 (http://lowelab.ucsc.edu/tRNAscan-SE/) was used to find the tRNA sequence in the genome according to the structural characteristics of tRNA. rRNA is highly conserved, and it is possible to select a closely related species as the reference sequence, and rRNA in the genome is searched by Blast v2.2.26 [47]. The INFERNAL (http://infernal.janelia.org/) software that comes with Rfam v14.1 [54] was used predict the miRNA and snRNA in the genome using the covariance model of the Rfam family.

Phylogenetic analysis and estimation of divergence time

We used OrthoFinder v2.5.2 [55] to identify orthologous genes from masked palm civet and 18 other species, including cat (*F. catus*), cattle (*Bos taurus*), dhole (*Cuon alpinus*), dog (*C. lupus familiaris*), human (*Homo sapiens*), leopard (*Panthera pardus*), lion (*Panthera leo*), lynx (*Lynx canadensis*), mouse (*M. musculus*), opossum (*Monodelphis domestica*), panda (*Ailuropoda melanoleuca*), pig (*Sus scrofa*), polarbear

(*Ursus maritimus*), rabbit (*Oryctolagus cuniculus*), red fox (*Vulpes vulpes*), tiger (*P. tigris altaica*), wolf (*Canis lupus*), and yak (*Bos mutus*) (Supplementary Table S1). Diamond was used for sequence similarity searches. We aligned the coding region and amino acid sequences of single-copy orthologous genes using MUSCLE v3.7 [56]. All alignments were concatenated to a super-alignment matrix. Based on this matrix, we used IQ-TREE v1.6.10 [57] which automatically detect the best model and then perform a maximum-likelihood (ML) phylogenetic tree based on this best model. 1,000 bootstrap replicates were performed to obtain confidence values for the branches. Bayesian Evolutionary Analysis Sampling Trees (BEAST) v2.6.0 [58] was used to estimate species divergence times based on amico acid sequences of single-copy genes. The tree topology based on IQ-TREE v1.6.10 [56] was used as starting tree, while splits between tiger and leopard (7.4 Ma), cat and tiger (15.2 Ma), polar bear and giant bear (23.4 Ma), pig and yak (62.0 Ma), mouse and human (90.0 Ma), as well as human and tiger (96.0 Ma) from TimeTree (http://www.timetree.org/, last accessed February 8, 2022) as calibration. We ran the Markov chain Monte Carlo for 10 million generations with sampling every 1000 generations, and 25% of the generations were discarded as burn-in. TreeAnnotator in BEAST v2.6.0 [58] was used to summarize the information from trees produced by BEAST v2.6.0 [58] onto a single target tree, while the target tree was visualized and exported as vector diagrams using FigTree v1.4.4 (http://tree.bio.ed.ac.uk/software/figtree/).

Gene family expansion and contraction analysis

To identify gene family evolution as a random birth and death process model, where the gene family either expands or contracts per gene per million years independently along each lineage of the phylogenetic tree, the ML model originally implemented in the software package CAFE v4.2.1 [59] was applied to compare the cluster size differences (gain or loss) between the ancestor and each species. We used species tree with divergence time obtained by BEAST v2.6.0 [58] as ultrametric tree. We determined gene family significantly expansions or contractions when the P value was less than 0.05.

Positive selection analysis

For positive selection analysis, we first identified single-copy orthologous genes from *P. larvata*, *C. lupus familiaris*, and the five most closely related species from the Felidae family with assembled genomes: *F. catus*, *P. pardus*, *P. leo*, *L. canadensis*, and *P. tigris altaica*. For these genes, based on phylogenetic topology, we employed the branch-site model incorporated in the PAML package v4.9 [60] to detect positively selected genes (PSGs). *P. larvata* was specified as a foreground branch, the other species were used as background branches. To determine genes under positive selection, likelihood ratio tests were conducted to determine whether positive selection was operating on the foreground branch. In this study, PSGs were identified only when $P < 0.01$. Receptors were important factors to intermediate virus infection. To study the immune mechanism of *P. larvata*, we looked out 28 known human virus receptors [61] in positive selected genes.

Sequence analysis of virus receptors under positive selection and *ACE2*

Sequences of virus receptor genes under positive selection and the *ACE2* gene in other animal species of cat (*F. catus*), cattle (*B. taurus*), dhole (*C. alpinus*), dog (*C. lupus familiaris*), human (*H. sapiens*), leopard (*P. pardus*), lion (*P. leo*), lynx (*L. canadensis*), mouse (*M. musculus*), opossum (*M. domestica*), panda (*A. melanoleuca*), pig (*S. scrofa*), polarbear (*U. maritimus*), rabbit (*O. cuniculus*), red fox (*V. vulpes*), tiger (*P. tigris altaica*), and yak (*B. mutus*) were downloaded from NCBI (https://www.ncbi.nlm.nih.gov/). Though *ACE2* may not positive selected in *P. larvata*, it is receptor of SARS-CoV and SARS-CoV-2, both resulting in severe pneumonia in human beings. Therefore, we want to know the genetic relationship of virus receptors between *P. larvata* and other animals. Alignment of amino acid sequences of receptors was conducted using MUSCLE v3.7 [56]. ML phylogenetic trees were constructed using IQ-TREE v1.6.10 [57], and 1,000 bootstrap replicates were performed to obtain confidence values for the branches. The sequence identity of amino acid sequences which could demonstrate the genetic distance between receptor genes in different animals was estimated by p-distance in MEGA-X [62].

Results and Discussion

Genome sequencing and assembly

We sequenced the genome of one female masked palm civet (2n = 44) (Figure 1a). Here, 557.75 Gb sequences were generated from high-quality short-read sequences (272.68 Gb) and PacBio sequences (285.07 Gb), representing over 216× coverage for the genome. The genome has an estimated size of 2.58 Gb based on the 17-mer depth distribution analysis of the sequenced reads (Supplementary Figure S1) The final assembled sequence was 2.44 Gb, representing 94.6% of the masked palm civet genome. The assembly consisted of 1,785 scaffolds with an N50 of and 111.87 Mb (Table 1). We used the Hi-C technology to reorder and anchor 95.32% of the genome onto 22 pseudochromosomes (Figure 1a, Supplementary Figure S2). The GC content and heterozygosity rate of the assembled masked palm civet genome were 42.4% and 0.46%, respectively. The completeness of our assembly was assessed by BUSCO and CEGMA. The BUSCO assessment showed that 96.5% of the 9,226 BUSCOs were assembled, with only 1.3% duplication (Supplementary Table S2), while CEGMA assessment showed that 91.94% and 3.2% of 248 CEGs (Core Eukaryotic Genes) were complete and partially assembled, respectively (Supplementary Table S3). We also used BWA v0.7.17 [26] to align Illumina short reads to the assembly and obtained a mapping rate and genome coverage of 99.35% and 99.73%, respectively. Overall, all evidence suggests that the *P. larvata* assembly is a high-quality genome assembly that can be used for downstream analyses.

Repeat annotation and gene prediction

We analyzed repetitive sequences by combining *de novo* prediction and a homology-based search at both the DNA and protein levels, and found that repetitive sequences occupied 31.80% (775.0 Mb) of the genome of the masked palm civet (Table 1, Figure 1a), which was very similar to the repeat compositions of the cat genome (32.10%), but lower than that of the tiger genome (39.20%) [44, 45]. The most abundant repetitive element type in the masked palm civet genome was long terminal repeat (LTR), representing 542.79 Mb (22.27%) of the genome. An annotation pipeline combining *de novo*, homolog-based search, and RNA-Seq methods was performed to

predict gene models from the repeat-masked masked palm civet genome sequence. These analyses predicted 21,582 protein-coding genes, representing 30.61% of the genome assembly (Supplementary Table S4, Supplementary Figure S3a). Among the 21,582 genes, 20,546 (95.20%) were successfully annotated for at least one function term by searching against functional databases (Gene Ontology (GO), InterProScan, KEGG, NR, Pfam, and SwissProt) (Table 1, Supplementary Figure S3b). In addition to the protein-coding genes, we also identified 14,829 miRNA genes, 47,575 tRNA genes, and 544 rRNA genes in the masked palm civet genome (Supplementary Table S5). The number of miRNA and tRNA genes seemed higher than other species. We double checked the methods and conducted noncoding RNAs prediction again, and obtained the same results. Maybe the high level of miRNA and tRNA genes is the personality of this species, which need further analysis in the future.

Gene family comparison

The evolutionary dynamics related to *P. larvata* were deduced by comparison with ten animal species. We identified 1,438 one-to-one orthologs among these species (Figure 1b; Supplementary Table S6). Among the 19 species, 44 gene families containing 119 genes were unique in *P. larvata* (Supplementary Table S6), containing a immune-related gene named *VSIG10L*, which is related to familial Barrett neoplasia [63]. Based on the P value threshold of 0.05, 35 gene families (including 243 genes) were significantly expanded; in contrast, 89 gene families (including 58 genes) were significantly contracted in the *P. larvata* genome (Figure 1c). There were 10 immune-related genes in significantly expanded genes, which were annotated to 3 genes of *TARM1* (T cell-interacting, activating receptor on myeloid cells-1), *IGLL1* (immunoglobulin lambda like polypeptide 1), and *OSCAR* (osteoclast associated Ig-like receptor) (Supplementary Table S7). *TARM1* is a novel leukocyte receptor complex which could encoded ITAM receptor that costimulates proinflammatory cytokine secretion by macrophages and neutrophils [64]. *TARM1* enables immunoglobulin receptor binding activity. *IGLL1* is a protein coding gene. Diseases associated with *IGLL1* include Agammaglobulinemia 2, Autosomal Recessive and Lambda 5 Deficiency [65]. *OSCAR* is a member of the leukocyte receptor complex protein family

that plays critical roles in the regulation of both innate and adaptive immune responses [66]. The encoded protein may play a role in oxidative stress-mediated atherogenesis as well as monocyte adhesion. Recent studies showed that *OSCAR* can promote proliferation and migration of lung adenocarcinoma in women [67]. We did not detcted immune-related genes in significantly contracted genes, which was in accordance with the high immune response level of masked palm civets.

Phylogenetic Analysis

To confirm the phylogenetic position of *P. larvata* and estimate divergence time using whole-genome data, we analyzed the orthologous gene relationships between *P. larvata* and 18 other animals (Supplementary Table S1). Based on a super alignment matrix of amino acid sequences and coding region sequences of 1,438 single-copy genes with opopssum as outgroup. The best model of cds and amino acid sequences were GTR+F+R3 and JTT+F+R5. Both trees shared the same topology that the genetic relationship between *P. larvata* and 5 species belong to the Felidae family were closely related. The phylogenetic tree based on amino acid sequences was used as starting tree to calculate the divergence time, which resulted the time of *P. larvata* split with the Felidae family was approximately 33.5 million years ago (Figure 1c). This is the first time of obtaining the divergence time of masked palm civets based on whole genome sequences.

Genes under positive selection

We used *P. larvata* as the foreground branch, and other species of *C. lupus familiaris*, *F. catus*, *P. pardus*, *P. leo*, *L. canadensis*, and *P. tigris altaica* as background branches to detect genes under selection. Based on 5,696 single-copy genes of these seven species, We identified 971 PSGs ($P < 0.01$; Supplementary Table S9). We did not observe genes under positive selection overlapped with expanded and contracted genes. One of these known human viral receptor genes, *PDGFRA*, were included in genes under positive selection in *P. larvata*. *PDGFRA* could activating mutations in gastrointestinal stromal tumors [68], and is required for human cytomegalovirus (HCMV) infection [69, 70]. There is no study focus on *PDGFRA* receptor in masked

palm civets, further study is needed. As an important receptor intermediate the infection of SARS-CoV and SARS-CoV-2, *ACE2* receptor was involved in this study though it is not under positive selection. The phylogeny of *PDGFRA,* and *ACE2* receptors from different animal species was conducted based on amino acid sequences downloaded from NCBI. The relationship of *PDGFRA* and *ACE2* in *P. larvata* was closest to four most closely related species from the Felidae family of *F. catus*, *P. pardus*, *L. canadensis*, and *P. tigris altaica* (Figure 2). The amino acid sequence identities of *PDGFRA* and *ACE2* were also highest among *P. larvata*, *F. catus*, *P. pardus*, *L. canadensis*, and *P. tigris altaica* (Table 2), demonstrating viruses using *PDGFRA* and *ACE2* might easily transferred between these species. However, further research is needed to reveal the differences in the receptor-binding loci of these receptors in *P. larvata* and other species, as well as the immunoreaction of cells after virus infection in consideration of different severity of symptoms in different species. Besides virus receptors, other ten immune-related genes were also under selection (Supplementary Table S10). Further studies are needed to reveal the roles of these immune-related genes under positive selection in civet immunity.

Conclusion

In this study, we present a high-quality chromosome-level genome assembly of *P. larvata* using PacBio single-molecule long-read sequencing, supplemented with Illumina short-read sequencing and Hi-C sequencing. The final genome assembly was 2.44 Gb, 95.32% of which could be grouped into 22 pseudochromosomes. This assembly is highly contiguous with contig N50 and scaffold N50 values of 12.97 Mb and 111.87 Mb. The repeat sequence comprised 31.80% of the genome. A total of 21,582 protein-coding genes were predicted, 95.20% of which could be functionally annotated. Phylogenetic analysis based on 1,438 single-copy orthogroups across 19 animals resulted in a close genetic relationship between *P. larvata* and five species from the Felidae family. The divergence time of *P. larvata* split from the Felidae family was estimated to be 33.5 Ma. Gene family clustering revealed 119 unique, 243 significantly expanded, and 58 significantly contracted genes in *P. larvata* genome. We identified 971 genes under positive selection in *P. larvata*, among which one known human viral

receptor genes were found. The high-quality genome assembly will provide a valuable genomic resource for elucidating the immune mechanisms in this animal. It will also provide a reliable reference for studying the genetic bases of the morphological characteristics, adaptive evolution, and evolutionary history of this species.

Data Availability

Raw sequencing data and genome assembly were deposited at the NCBI under the BioProject PRJNA756156 with biosample accession number SAMN20856571. The annotations and predicted peptides are available in FigShare at the link: https://doi.org/10.6084/m9.figshare.15273879.

Competing Interests

The authors declare that they have no competing interests.

Funding

This project was supported by the surveillance of wild animals and epidemic diseases in important ecological areas of Guangdong Province project from the Forestry Administration of Guangdong Province, GDAS Special Project of Science and Technology Development (2020GDASYL-20200103090).

Acknowledgements

The sequencing in this study was conducted by the Novogene Company (Beijing, China). Thanks for the hard work of Hao Lv and her colleagues. Thanks for the helpful advices of Chen Feng and Chao Feng from South China Botanical Garden, Chinese Academy of Sciences in data analysis.

Figure Legends

Figure 1 (a) Synteny of corresponding chromosomes and gene density across the *Paguma larvata* genome; (b) Comparison of copy numbers in gene clusters reside in the genomes of *P. larvata* and other 18 animals. Single-copy orthologs denote the family can have only one gene for each species, and multicopy orthologs denote the

family clustered more than one gene for each species. Other orthologs denote the family can have any number of genes for each species except the single-copy and multi-copy orthologs. Unique paralogs denote species-specific gene families, and unclustered genes denote species-specific genes that cannot cluster with any other genes; (c) Phylogenetic relationships of 19 animal genomes based on the maximum likelihood method. The black numbers indicate estimated divergent times as the number of years (millions) ago, while green and red numbers denote gene families subject to expansion and contraction for each species, respectively.

Figure 2 Phylogenetic relationships of known human virus receptor of *PDGFRA* under positive selection and *ACE2* receptor in *P. larvata* and other species.

References

1. Iwami S, Takeuchi Y, Liu X. Avian flu pandemic: Can we prevent it?. J Thero Biol 2009;**257**(1):181-190.
2. Siddiqui AA. The global threat of bird (Avian) flu its treatment methods and public health preventive measures. SOJ Vet Sci 2018; **4**(3):1-4.
3. Dixon L K, Sun H, Roberts H. African swine fever. Antivir Res 2019;**165**:34-41.
4. Rota PA, Oberste MS, Monroe SS, et al. Characterization of a novel coronavirus associated with severe acute respiratory syndrome. Science 2003;**300**(5624):1394-1399.
5. Zumla A, Hui D S, Perlman S. Middle East respiratory syndrome. The Lancet 2015;**386**(9997):995-1007.
6. Wu D, Wu T, Liu Q, et al. The SARS-CoV-2 outbreak: what we know. Int J Infect Dis 2020;**94**:44-48.
7. Sabir JSM, Lam TTY, Ahmed MMM, et al. Co-circulation of three camel coronavirus species and recombination of MERS-CoVs in Saudi Arabia. Science 2016;**351**(6268):81-84.
8. Inoue T, Kaneko Y, Yamazaki K, et al. Genetic population structure of the masked palm civet *Paguma larvata*,(Carnivora: Viverridae) in Japan, revealed from analysis of newly identified compound microsatellites. Conserv Genet 2012;**13**(4):1095-1107.
9. Guan Y, Zheng BJ, He YQ, et al. Isolation and characterization of viruses related to the SARS coronavirus from animals in southern China. Science 2003;**302**(5643):276-278.
10. Li W, Shi Z, Yu M, et al. Bats are natural reservoirs of SARS-like coronaviruses. Science 2005;**310**(5748):676-679.
11. Wang LF, Eaton BT. Bats, civets and the emergence of SARS. Wildlife and emerging zoonotic diseases: the biology, circumstances and consequences of cross-species transmission, 2007:325-344.
12. Wan Y, Shang J, Graham R, et al. Receptor recognition by the novel coronavirus from Wuhan: an analysis based on decade-long structural studies of SARS coronavirus. J Virol 2020;**94**(7):e00127-20.

13. Marsh M, Helenius A. Virus entry: open sesame. Cell 2006;124(4):729-740.
14. Grove J, Marsh M. The cell biology of receptor-mediated virus entry. J Cell Biol 2011;**195**(7):1071-1082.
15. Li W, Moore MJ, Vasilieva N, et al. Angiotensin-converting enzyme 2 is a functional receptor for the SARS coronavirus. Nature 2003;**426**(6965):450-454.
16. Shi R, Shan C, Duan X, et al. A human neutralizing antibody targets the receptor-binding site of SARS-CoV-2. Nature 2020;**584**(7819):120-124.
17. Wu Y, Wang F, Shen C, et al. A noncompeting pair of human neutralizing antibodies block COVID-19 virus binding to its receptor ACE2. Science 2020;**368**(6496):1274-1278.
18. Bickhart DM, Rosen BD, Koren S, et al. Single-molecule sequencing and chromatin conformation capture enable de novo reference assembly of the domestic goat genome. Nat genet, 2017;**49**(4):643-650.
19. Mahajan S, Wei K, Nalley M, et al. De novo assembly of a young Drosophila Y chromosome using Single-Molecule sequencing and Chromatin Conformation capture. PLoS Biol 2018;**16**:1-28.
20. Zhang Z, Chen Y, Zhang J, et al. Improved genome assembly provides new insights into genome evolution in a desert poplar (*Populus euphratica*). Mol Ecol Resour 2020;**20**(3):781-794.
21. Rio DC, Ares M, Hannon GJ, et al. Purification of RNA using TRIzol (TRI reagent). Cold Spring Harbor Protocols, 2010;**2010**(6):pdb. prot5439.
22. Chin CS, Alexander DH, Marks P, et al. Nonhybrid, finished microbial genome assemblies from long-read SMRT sequencing data. Nat methods 2013;**10**(6):563-569.
23. Belton JM, McCord RP, Gibcus JH, et al. Hi – C: a comprehensive technique to capture the conformation of genomes. Methods 2012;**58**(3):268-276.
24. Chin CS, Peluso P, Sedlazeck FJ, et al. Phased diploid genome assembly with single-molecule real-time sequencing. Nat methods 2016;**13**(12):1050-1054.
25. Walker BJ, Abeel T, Shea T, et al. Pilon: an integrated tool for comprehensive microbial variant detection and genome assembly improvement. PloS one 2014;9(11):e112963.

26. Li H, Durbin R. Fast and accurate short read alignment with Burrows-Wheeler transform. Bioinformatics 2009;**25**:175-1760.
27. Hariharan R, Toyama K. Project Lachesis: parsing and modeling location histories//International Conference on Geographic Information Science. Springer, Berlin, Heidelberg, 2004:106-124.
28. Burton JN, Adey A, Patwardhan RP, et al. Chromosome-scale scaffolding of de novo genome assemblies based on chromatin interactions. Nat Biotechnol 2013;**31**:1119-1125.
29. Liu B, Shi Y, Yuan J, et al. Estimation of genomic characteristics by analyzing k-mer frequency in de novo genome projects. 2013. http://arxiv.org/abs/1308.2012.
30. Manni M, Berkeley MR, Seppey M, et al. BUSCO update: novel and streamlined workflows along with broader and deeper phylogenetic coverage for scoring of eukaryotic, prokaryotic, and viral genomes. Mol Biol Evol 2021;**38**(10): 4647-s4654
31. Parra G, Bradnam K, Korf I. CEGMA: A pipeline to accurately annotate core genes in eukaryotic genomes. Bioinformatics 2007;**23**:1061-1067.
32. Grabherr MG, Haas BJ, Yassour M, et al. Full-length transcriptome assembly from RNA-Seq data without a reference genome. Nat Biotechnol 2011;**29**:644-652.
33. Kent WJ. BLAT—the BLAST-Like Alignment Tool. Genome Res 2002;12:656-664.
34. Trapnell C, Roberts A, Goff L, et al. Differential gene and transcript expression analysis of RNA-seq experiments with TopHat and Cufflinks. Nat Protoc 2012;**7**:562-578.
35. Bao W, Kojima KK, Kohany O. Repbase Update, a database of repetitive elements in eukaryotic genomes. Mob DNA 2015;**6**:4-9.
36. Benson G. Tandem repeats finder: A program to analyze DNA sequences. Nucleic Acids Res 1999;**27**:573-580.
37. Stanke M, Schöffmann O, Morgenstern B, et al. Gene prediction in eukaryotes with a generalized hidden Markov model that uses hints from external sources. BMC Bioinformatics 2006;**7**:1-11.
38. Majoros WH, Pertea M, Salzberg SL. TigrScan and GlimmerHMM: Two open

source ab initio eukaryotic gene-finders. Bioinformatics 2004;**20**:2878-2879.

39. Korf I. Gene finding in novel genomes. BMC Bioinformatics 2004;**5**:1-9.
40. Burge C, Karlin S. Prediction of complete gene structures in human genomic DNA. J Mol Biol 1997;**268**:78-94.
41. Guigó R, Knudsen S, Drake N, et al. Prediction of gene structure. J Mol Biol 1992;**226**:141-157.
42. Gnerre S, MacCallum I, Przybylski D, et al. High-quality draft assemblies of mammalian genomes from massively parallel sequence data. Proc Natl Acad Sci USA 2011;**108**:1513-1518.
43. Kirkness EF, Bafna V, Halpern AL, et al. The dog genome: Survey sequencing and comparative analysis. Science 2003;**301**(5641):1898-1903.
44. Pontius JU, Mullikin JC, Smith DR, et al. Initial sequence and comparative analysis of the cat genome. Genome Res 2007;**17**(11):1675-1689.
45. Cho Y S, Hu L, Hou H, et al. The tiger genome and comparative analysis with lion and snow leopard genomes. Nat Commun 2013;**4**(1):1-7.
46. Dobrynin P, Liu S, Tamazian G, et al. Genomic legacy of the African cheetah, *Acinonyx jubatus*. Genome Biol 2015;**16**(1):1-20.
47. Altschul SF, Gish W, Miller W, et al. Basic local alignment search tool. J Mol Biol 1990;**215**:403-410.
48. Birney E, Clamp M, Durbin R. GeneWise and Genomewise. Genome Res 2004;**14**:988-995.
49. Kim D, Pertea G, Trapnell C, et al. TopHat2: accurate alignment of transcriptomes in the presence of insertions, deletions and gene fusions. Genome biol 2013;**14**(4):1-13.
50. Haas BJ, Delcher AL, Mount SM, et al. Improving the Arabidopsis genome annotation using maximal transcript alignment assemblies. Nucleic Acids Res 2003;**31**:5654-5666.
51. Haas BJ, Salzberg SL, Zhu W, et al. Automated eukaryotic gene structure annotation using EVidenceModeler and the Program to Assemble Spliced Alignments. Genome biol 2008;**9**(1):1-22.
52. Kanehisa M, Sato Y, Kawashima M, et al. KEGG as a reference resource for gene

and protein annotation. Nucleic Acids Res 2016;**44**:457-462.
53. Quevillon E, Silventoinen V, Pillai S, et al. InterProScan: protein domains identifier. Nucleic Acids Res 2005;**33**:116-120.
54. Finn RD, Coggill P, Eberhardt RY, et al. The Pfam protein families database: towards a more sustainable future. Nucleic Acids Res 2016;**44**(D1):D279-D285.
55. Emms DM, Kelly S. OrthoFinder: Solving fundamental biases in whole genomecomparisons dramatically improves orthogroup inference accuracy. Genome Biol 2015;**16**: 157.
56. Edgar RC. MUSCLE: Multiple sequence alignment with high accuracy and high throughput. Nucleic Acids Res 2004;**32**:1792-1797.
57. Nguyen LT, Schmidt HA, Von Haeseler A, et al. IQ-TREE: a fast and effective stochastic algorithm for estimating maximum-likelihood phylogenies. Mol Biol Evol 2015;**32**(1):268-274.
58. Bouckaert R, Heled J, Kühnert D, et al. BEAST 2: a software platform for Bayesian evolutionary analysis. PLoS comput biol, 2014;**10**(4):e1003537.
59. De Bie T, Cristianini N, Demuth JP, et al. CAFE: A computational tool for the study of gene family evolution. Bioinformatics 2006;**22**:1269-1271.
60. Yang Z. PAML 4: Phylogenetic analysis by maximum likelihood. Mol Biol Evol 2007;**24**:1586-1591.
61. Ren L, Wu C, Guo L, et al. Single-cell transcriptional atlas of the Chinese horseshoe bat (*Rhinolophus sinicus*) provides insight into the cellular mechanisms which enable bats to be viral reservoirs. bioRxiv 2020. DOI:10.1101/2020.06.30.175778
62. Kumar S, Stecher G, Li M, et al. MEGA X: molecular evolutionary genetics analysis across computing platforms. Mol Biol Evol 2018;**35**(6):1547.
63. Fecteau R E, Kong J, Kresak A, et al. Association between germline mutation in VSIG10L and familial Barrett neoplasia. JAMA Oncol 2016;**2**(10):1333-1339.
64. Radjabova V, Mastroeni P, Skjødt K, et al. TARM1 Is a Novel Leukocyte Receptor Complex – Encoded ITAM Receptor That Costimulates Proinflammatory Cytokine Secretion by Macrophages and Neutrophils. J Immunol 2015;**195**(7):3149-3159.

65. Gemayel KT, Litman GW, Sriaroon P. Autosomal recessive agammaglobulinemia associated with an IGLL1 gene missense mutation. Ann Allerg, Asthma Im 2016;**117**(4):439-441.
66. Kim N, Takami M, Rho J, et al. A novel member of the leukocyte receptor complex regulates osteoclast differentiation. J Exp Med 2002;**195**(2):201-209.
67. Wang D, Yang N, Xie S. Sex‐biased ceRNA networks reveal that OSCAR can promote proliferation and migration of lung adenocarcinoma in women. Clin Exp Pharmacol P 2020;**47**(8):1350-1359.
68. Heinrich M C, Corless C L, Duensing A, et al. PDGFRA activating mutations in gastrointestinal stromal tumors. Science 2003;**299**(5607):708-710.
69. Soroceanu L, Akhavan A, Cobbs CS. Platelet-derived growth factor-α receptor activation is required for human cytomegalovirus infection. Nature 2008;**455**(7211):391-395.
70. Yang Z, Tang X, McMullen T P W, et al. PDGFRα Enhanced Infection of Breast Cancer Cells with Human Cytomegalovirus but Infection of Fibroblasts Increased Prometastatic Inflammation Involving Lysophosphatidate Signaling. Int J Mol Sci 2021;**22**(18): 9817.

Tables

Table 1 *Paguma larvata* Genome Assembly and Annotation Summary.

Genome assembly statistics	
Total length (bp)	2,437,385,791
Contig/Scaffold N50 length (bp)	12,971,789
Contig/Scaffold N90 length (bp)	2,389,527
Longest contig length (bp)	52,858,878
Number of contigs	1,785
Number of contigs larger than N50	56
Number of contigs larger than N90	210
Genome characteristics	
GC content	42.41%
Number of protein-coding genes	21,582
Average transcript length (bp)	34,567.54
Average CDS length (bp)	1,481.00
Average exon length (bp)	170.53
Average exons per gene (bp)	8.68
Repetitive sequences (% of genome)	
DNA (bp)	8,232,288 (0.34%)
LINE (bp)	299,126,376 (12.27%)
SINE (bp)	613,247 (0.03%)
LTR (bp)	542,789,387 (22.27%)
Unknown (bp)	12,280,567 (0.5%)
Total (bp)	775,001,321 (31.8%)
Gene annotations (% of all genes)	
GO	14,148 (65.6%)
InterProScan	19,770 (91.6%)
KEGG	18,200 (84.3%)
Nr	20,480 (94.9%)
PFAM	17,505 (81.1%)
SwissProt	20,033 (92.8%)
Total	21,582

Table 2 Sequence identity of receptors under positive selection between masked palm civet and other animal species.

**Species**	**Sequence identity (%)**	
	PDGFRA	ACE2
Lynx canadensis	**97.95**	**93.17**
Felis catus	**97.85**	**92.67**
Panthera tigris altaica	**97.85**	**92.60**
Panthera pardus	**97.67**	**92.80**
Canis lupus familiaris	96.55	88.93
Vulpes vulpes	96.55	88.81
Ursus maritimus	95.99	89.94
Ailuropoda melanoleuca	95.24	89.79
Homo sapiens	95.05	83.35
Sus scrofa	94.86	82.48
Manis javanica	93.54	85.47
Bos taurus	93.18	81.84
Bos mutus	93.09	81.84
Desmodus rotundus	92.16	79.35
Mus musculus	90.01	81.74
Oryctolagus cuniculus	88.14	83.11
Monodelphis domestica	83.49	71.14

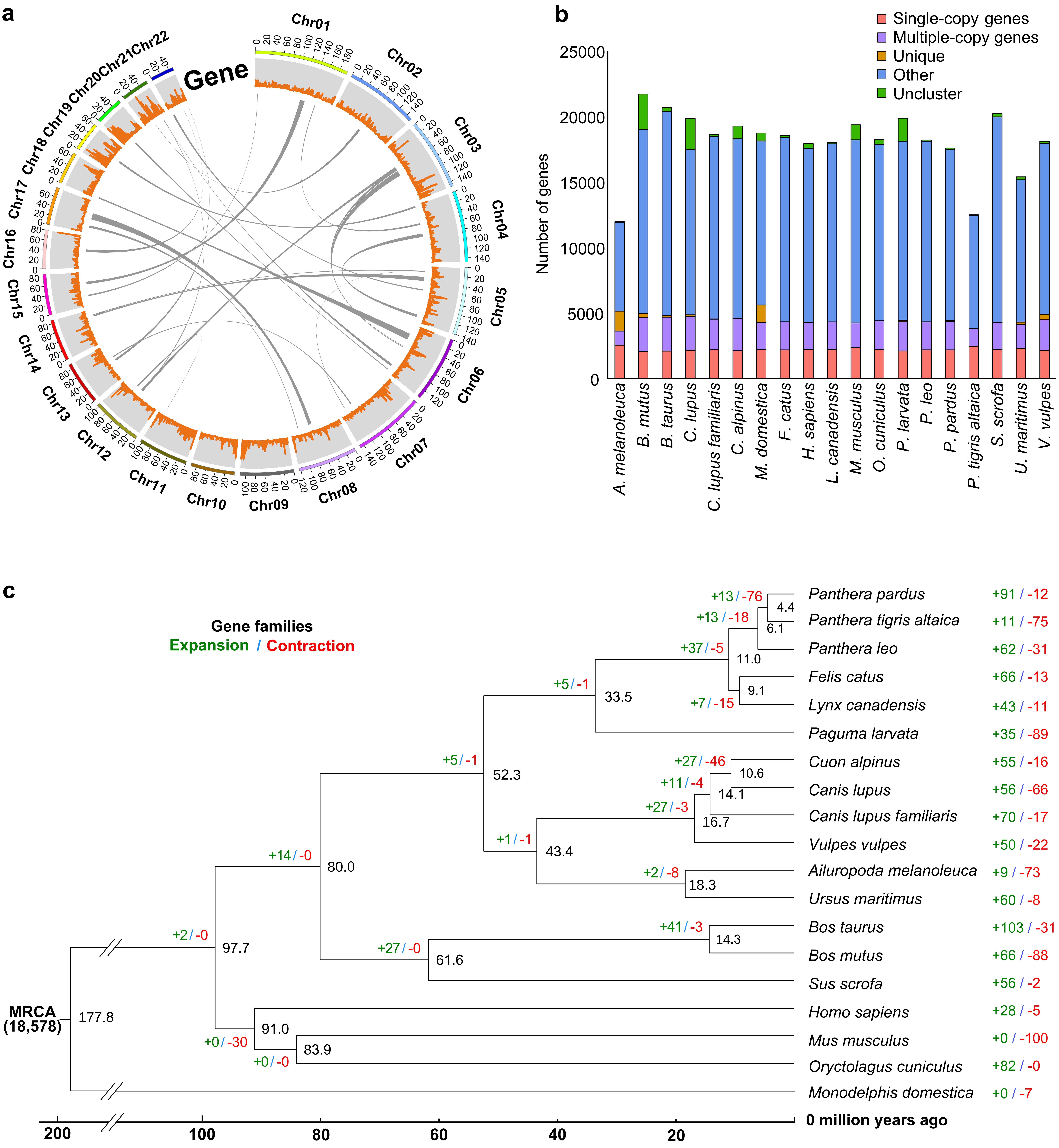
a
Gene
Chr01
Chr02
Chr03
Chr04
Chr05
Chr06
Chr07
Chr08
Chr09
Chr10
Chr11
Chr12
Chr13
Chr14
Chr15
Chr16
Chr17
Chr18
Chr19
Chr20
Chr21
Chr22
b
Single-copy genes
Multiple-copy genes
Unique
Other
Uncluster
Number of genes
25000
20000
15000
10000
5000
0
A. melanoleuca
B. mutus
B. taurus
C. lupus
C. lupus familiaris
C. alpinus
M. domestica
F. catus
H. sapiens
L. canadensis
M. musculus
O. cuniculus
P. larvata
P. leo
P. pardus
P. tigris altaica
S. scrofa
U. maritimus
V. vulpes
c
Gene families
Expansion / Contraction
MRCA
(18,578)
177.8
97.7
91.0
83.9
80.0
61.6
52.3
43.4
33.5
18.3
16.7
14.3
14.1
11.0
10.6
9.1
6.1
4.4
+13 / -76
+13 / -18
+37 / -5
+5 / -1
+7 / -15
+5 / -1
+27 / -46
+11 / -4
+27 / -3
+1 / -1
+2 / -8
+14 / -0
+41 / -3
+2 / -0
+27 / -0
+0 / -30
+0 / -0
Panthera pardus +91 / -12
Panthera tigris altaica +11 / -75
Panthera leo +62 / -31
Felis catus +66 / -13
Lynx canadensis +43 / -11
Paguma larvata +35 / -89
Cuon alpinus +55 / -16
Canis lupus +56 / -66
Canis lupus familiaris +70 / -17
Vulpes vulpes +50 / -22
Ailuropoda melanoleuca +9 / -73
Ursus maritimus +60 / -8
Bos taurus +103 / -31
Bos mutus +66 / -88
Sus scrofa +56 / -2
Homo sapiens +28 / -5
Mus musculus +0 / -100
Oryctolagus cuniculus +82 / -0
Monodelphis domestica +0 / -7
200
100
80
60
40
20
0 million years ago

Figure 2
Click here to access/download;Figure;Figure 2-aa_trees.pdf

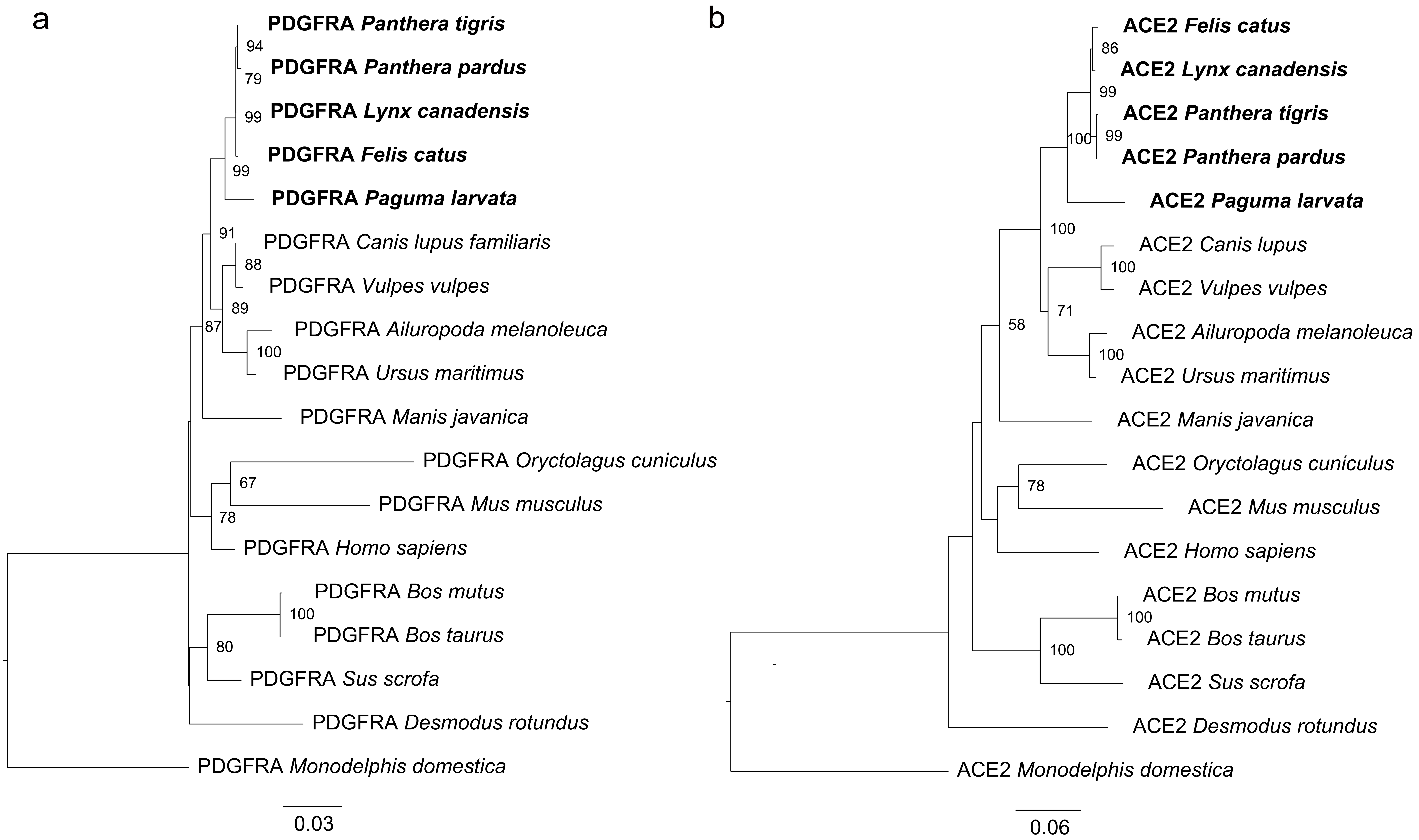

Supplementary tables

Click here to access/download
Supplementary Material
Supplementary Tables-20220210.xlsx

Supplementary figures

Click here to access/download
Supplementary Material
Supplementary Figures-20220210.docx

cover letter

Click here to access/download
Supplementary Material
cover letter.docx

Reply to commonts

Click here to access/download
Supplementary Material
Commonts-20220211.docx
