# Supplementary material for: The assembled and annotated genome of the masked palm civet (Paguma larvata)
Source: Gigascience. 2022 May 18;11:giac041. doi: 10.1093/gigascience/giac041 (PMC9116208; doi:10.1093/gigascience/giac041)
Supplement: giac041_GIGA-D-21-00297_Revision_2 [file giac041_giga-d-21-00297_revision_2.pdf]

GigaScience

The Assembled and Annotated Genome of the Masked Palm Civet (Paguma larvata)

--Manuscript Draft--

**Manuscript Number:**	GIGA-D-21-00297R2
**Full Title:**	The Assembled and Annotated Genome of the Masked Palm Civet (Paguma larvata)
**Article Type:**	Data Note
**Funding Information:**	GDAS Special Project of Science and Technology Development (2020GDASYL-20200103090) — DR Ping Liu<br>Guangdong Province project from the Forestry Administration of Guangdong Province — DR Ping Liu
**Abstract:**	Background: The masked palm civet (Paguma larvata) acts as an intermediate host of severe acute respiratory syndrome coronavirus (SARS-CoV), which caused SARS, and transfers this virus from bats to humans. Additionally, P. larvata has the potential to carry a variety of zoonotic viruses that may threaten human health. However, genome resources for P. larvata have not been reported to date. Findings: A chromosome-level genome assembly of P. larvata was generated using PacBio sequencing, Illumina sequencing, and Hi-C technology. The genome assembly was 2.44 Gb in size, of which 95.32% could be grouped into 22 pseudochromosomes, with contig N50 and scaffold N50 values of 12.97 Mb and 111.87 Mb, respectively. A total of 21,582 protein-coding genes were predicted, and 95.20% of the predicted genes were functionally annotated. Phylogenetic analysis of 19 animal species confirmed the close genetic relationship between P. larvata and species belong to the Felidae family. Gene family clustering revealed 119 unique, 362 significantly expanded, and 58 significantly contracted genes in the P. larvata genome. We identified 971 positively selected genes in P. larvata , and one known human viral receptor gene PDGFRA is positively selected in P. larvata , which is required for human cytomegalovirus (HCMV) infection, Conclusions: This high-quality genome assembly provides a valuable genomic resource for exploring virus-host interactions . It will also provide a reliable reference for studying the genetic bases of the morphological characteristics, adaptive evolution, and evolutionary history of this species.
**Corresponding Author:**	Jin-Ping Chen<br>Guangdong Academy of Sciences<br>Guangzhou, CHINA
**Corresponding Author Secondary Information:**	
**Corresponding Author's Institution:**	Guangdong Academy of Sciences
**Corresponding Author's Secondary Institution:**	
**First Author:**	Ping Liu
**First Author Secondary Information:**	
**Order of Authors:**	Ping Liu
	Hai-Ying Jiang
	Lin-Miao Li
	Jia-Bin Zhou
	Wen-Zhong Huang
	Jin-Ping Chen
**Order of Authors Secondary Information:**	
**Response to Reviewers:**	Reviewer reports:

Powered by Editorial Manager® and ProduXion Manager® from Aries Systems Corporation

	Reviewer #1: The authors have fully addressed my concerns. I have no more comments about this revised manuscript.<br><br>Reviewer #2: Reviewer's comments<br>The authors have given strong responses to my comments, and have carried out additional analyses or modified sections as appropriate, which I really appreciate. My biggest concern was the number of genes unique to P. larvata relative to other carnivorans. My final comment relate specifically to how this is referred to in the text, and the validity of the statement:<br><br>The authors state "among the 19 species, 44 gene families containing 119 genes were unique to P. larvata."<br><br>I still think this reads a little confusing, as to me it gives the impression that 44 unique gene families were found. If the authors rearranged the sentence to something like "among the 19 species, 119 genes belonging to 44 gene families were found to be unique to P. larvata" or something similar, it might be less confusing. I'm still not 100% convinced that these 119 genes are wholly unique to the P. larvata genome. The authors mention that perhaps they are simply not annotated in closely related species enough to be flagged as orthologs. If the authors map these 119 genes to the genome, for example by comparing them against the raw genome files of some closely related carnivorans via blastn, do they hit anywhere in the genome, and if so, do those loci regions being hit contain any CDS annotations in the RefSeq genome file? I realise this is a bit of a sticking point with me, but perhaps even if the authors can acknowledge whether or not this number of unique genes is a sure thing, or could be due to poor annotations explicitly in the text, it might help.<br>Reply: Thanks for your advice! We have rearranged the sentence from “among the 19 species, 44 gene families containing 119 genes were unique to P. larvata” to “Among the 19 species, 119 genes belonging to 44 gene families were unique in P. larvata” in line 296-297, and we are sorry about this mistake. We will try to explain this question in three aspects: Firstly, before phylogenetic orthology inference, we filtered out genes in other 18 species with missing data, and did not start with initiation codon, this step may be deleted some genes which may be potentially in the same orthologs with unique genes in P. larvata. secondly, the unique genes were determined by software algorithms. It's worth noting that results using different versions of OrthoFinder have some differences. In our paper, we used OrthoFinder version 2.5.2 to conduct phylogenetic orthology inference, and 119 unique genes were detected. Actually, we also used OrthoFinder version 2.2.7, and 6 unique genes were detected. Both analysis were conducted twice, and the results were the same. Finally we chose the results of advanced version of OrthoFinder, which showed 119 genes were unique in P. larvata. Thirdly, we must point out that these unique genes just “unique” in P. larvata compared to other 18 species in our study. Just as reviewer said, we blasted several sequences of these unique genes, some of which could hit in other species. We searched other studies, and found out the unique genes were also detected in other species (doi: 10.1093/gigascience/giab027; doi: 10.1093/gigascience/giab016; doi: 10.1093/gigascience/giy108; doi: 10.1093/gigascience/giy094). Therefore, we think the results of our study are credible, and we also appreciated your precious advice.
**Additional Information:**	
**Question**	**Response**
Are you submitting this manuscript to a special series or article collection?	No
**Experimental design and statistics**<br><br><br>Full details of the experimental design and statistical methods used should be given in the Methods section, as detailed in our Minimum Standards Reporting Checklist. Information essential to interpreting the	Yes

data presented should be made available in the figure legends.<br><br>Have you included all the information requested in your manuscript?	
**Resources**<br><br>A description of all resources used, including antibodies, cell lines, animals and software tools, with enough information to allow them to be uniquely identified, should be included in the Methods section. Authors are strongly encouraged to cite Research Resource Identifiers (RRIDs) for antibodies, model organisms and tools, where possible.<br><br>Have you included the information requested as detailed in our Minimum Standards Reporting Checklist?	Yes
**Availability of data and materials**<br><br>All datasets and code on which the conclusions of the paper rely must be either included in your submission or deposited in publicly available repositories (where available and ethically appropriate), referencing such data using a unique identifier in the references and in the “Availability of Data and Materials” section of your manuscript.<br><br>Have you have met the above requirement as detailed in our Minimum Standards Reporting Checklist?	Yes

Manuscript

Click here to access/download;Manuscript;GIGA-D-21-00297-manuscript-March 25.docx

The Assembled and Annotated Genome of the Masked Palm Civet (*Paguma larvata*)

Ping Liu[1], Hai-Ying Jiang[1], Lin-Miao Li[1], Jia-Bin Zhou[1], Wen-Zhong Huang[1], Jin-Ping Chen[1]

[1]Guangdong Key Laboratory of Animal Conservation and Resource Utilization, Guangdong Public Laboratory of Wild Animal Conservation and Utilization, Institute of Zoology, Guangdong Academy of Sciences, Guangzhou 510260, China; pingliu0330@126.com

Corresponding author: Jin-Ping Chen, Institute of Zoology, Guangdong Academy of Sciences, Guangzhou 510260, China. chenjp@giz.gd.cn; Tel.: +020-8910-0920

Jin-Ping Chen [0000-0001-9712-9289];
Ping Liu [0000-0001-7823-2731];
Hai-Ying Jiang [0000-0002-9072-8569];
Lin-Miao Li [0000-0001-8192-3082];
Jia-Bin Zhou [0000-0001-5716-5015];
Wen-Zhong Huang [0000-0002-1803-700X]

Abstract

Background: The masked palm civet (*Paguma larvata*) acts as an intermediate host of severe acute respiratory syndrome coronavirus (SARS-CoV), which caused SARS, and transfers this virus from bats to humans. Additionally, *P. larvata* has the potential to carry a variety of zoonotic viruses that may threaten human health. However, genome resources for *P. larvata* have not been reported to date. **Findings:** A chromosome-level genome assembly of *P. larvata* was generated using PacBio sequencing, Illumina sequencing, and Hi-C technology. The genome assembly was 2.44 Gb in size, of which 95.32% could be grouped into 22 pseudochromosomes, with contig N50 and scaffold N50 values of 12.97 Mb and 111.87 Mb, respectively. A total of 21,582 protein-coding genes were predicted, and 95.20% of the predicted genes were

functionally annotated. Phylogenetic analysis of 19 animal species confirmed the close genetic relationship between *P. larvata* and species belong to the Felidae family. Gene family clustering revealed 119 unique, 362 significantly expanded, and 58 significantly contracted genes in the *P. larvata* genome. We identified 971 positively selected genes in *P. larvata*, and one known human viral receptor gene *PDGFRA* is positively selected in *P. larvata*, which is required for human cytomegalovirus (HCMV) infection, **Conclusions:** This high-quality genome assembly provides a valuable genomic resource for exploring virus-host interactions. It will also provide a reliable reference for studying the genetic bases of the morphological characteristics, adaptive evolution, and evolutionary history of this species.

Keywords: Masked palm civet, genome assembly, Hi-C proximity mapping, gene family evolution, phylogeny, positive selection

Introduction

In human history, the spread of viruses has caused huge losses to the human economy and threatened the safety of human life and property. Diseases affecting animals of economic value, caused by viruses such as avian influenza and African swine fever, have caused serious economic losses [1-3]. Viruses such as severe acute respiratory syndrome coronavirus (SARS-CoV), Middle East respiratory syndrome coronavirus (MERS-CoV), and SARS-CoV-2, pose a great threat to the safety of human life and property [4-6]. Usually, zoonotic viruses can be transmitted to humans via wildlife as intermediate hosts. For example, MERS-CoV is transmitted from camels to humans [7].

Masked palm civets, *Paguma larvata* (NCBI:txid9675) (Mammalia, Carnivora, and Viverridae) are wild mammals widely distributed in Asia [8]. They are omnivores, although they mainly consume fruits. In recent decades, *P. larvata* used to be domesticated as meat of human beings, increasing the possibility of transferring pathogenic microorganisms to human beings. In 2003, *P. larvata* gained attention because of its potential involvement in the outbreak of severe acute respiratory syndrome (SARS), which originated in southern China and spread to over 30 countries

[9]. Further research suggested that *P. larvata* acted as an intermediate host of SARS-CoV, which transmits this virus from bats to humans [10, 11]. The sequence similarity between the SARS-CoV found in *P. larvata* and human SARS-CoV is greater than 99%. However, *P. larvata* can live in harmony with SARS-CoV, whereas humans can have severe symptoms following SARS-CoV infection. This shows that the interaction between *P. larvata* and viruses has a certain particularity [12]. Earlier studies on farms raising *P. larvata* also found that these animals rarely become ill. The immune system of *P. larvata* is thus worthy of in-depth study.

Virus receptors, present on the host cell membrane, are important factors in mediating virus invasion and play an extremely important role in the interaction between host cells and viruses. The virus attachment protein can be regarded as the "key" that unlocks the cell by interacting with the "lock" (receptor) on the surface of the host cell, starting a key downstream step in the virus life cycle. In many cases, viruses use multiple receptors to perform different functions during the viral life cycle [13]. Therefore, the virus must first find the lock and then use a specific virus "key" to unlock the cell. The numerous functions of the virus receptor aid the virus in targeting the correct tissue for infection and to cross the cell barrier, which is necessary to deliver the viral genome to the host cell [14]. The interaction with viral receptors is usually mediated by specific proteins on the surface of the virus [13]. Inherent differences in the shape of the virus surface protein (icosahedron or helix) and the composition of the virus coat affect the overall structure of the virus attachment protein. Some viruses infect only certain species; for example, swine fever virus can only infect pigs [3]. Whereas some viruses can spread between animals and humans, such as SARS-CoV [10, 11] and MERS-CoV [7]; the reason lies in the nature of the interaction between the virus receptor and virus attachment protein. For example, SARS-CoV and SARS-CoV-2 specifically recognize the angiotensin-converting enzyme 2 (*ACE2*) receptor [15, 16, 17]. Understanding the nature of virus receptors that cause zoonotic diseases in different species will help in tracing natural hosts during the virus epidemic.

As an important virus host, masked palm civets can be used as a system for reviewing virus and host interactions. However, there is currently no genome assembly of *P. larvata*, even in Viverridae which has been reported for studying various virus

receptors. In this study, we generated a chromosome-level reference genome of *P. larvata* by integrating PacBio sequencing, Hi-C sequencing, and Illumina sequencing [18-20]. The high-quality genome assembly will provide a valuable genomic resource for exploring virus-host interactions. It will also provide a reliable reference for studying the genetic bases of the morphological characteristics, adaption evolution, and evolutionary history of the species.

Data Description

Statement of Ethics

The study design was approved by the Ethics Committee for Animal Experiments at the Institute of Zoology, Guangdong Academy of Sciences (reference number: GIZ20190702, 07/01/2019) and followed the basic principles outlined by this committee.

Isolation of genomic DNA and RNA

A healthy female *P. larvata* individual was collected from a farm in Wengyuan County, Guangdong Province, China. Whole blood was isolated from the same masked palm civet and washed with saline nasal spray, then immediately frozen in liquid nitrogen and stored at -80 °C prior to nucleic acid extraction. Genomic DNA was extracted from whole blood using a magnetic universal genomic DNA kit. Different tissues, including heart, intestine, kidney, liver, lung, muscle, and spleen were sampled from the same one female *P. larvata* individual and flash-frozen in liquid nitrogen for RNA sequencing, RNA-seq data was used to predict genes in following analysis. Total RNA was extracted using the TRIzol reagent [21].

Genome sequencing with different technologies

A combined sequencing strategy was used to obtain the masked palm civet genome. Short-insert libraries with a size of 350 bp were sequenced on the Illumina HiSeq 2500 platform (Illumina HiSeq 2500 System, RRID:SCR_016383) using the 150 bp paired-end protocol. For PacBio library construction, we sheared the genomic DNA to 20 kb fragments, which we used to prepare a single-molecule real-time (SMRT) library for

sequencing with the PacBio Sequel system (PacBio Sequel System, RRID:SCR_017989) [22]. Liver tissue was used to construct the Hi-C sequencing library. Briefly, cross-linked chromatin was digested with Dpn II and ligated in situ after biotinylation. DNA fragments were enriched via the interaction of biotin and blunt-end ligation and then subjected to Illumina NovaSeq (Illumina NovaSeq 6000 Sequencing System, RRID:SCR_016387) PE 150 sequencing [23].

Genome assembly and quality assessment

We applied the FALCON (FALCON, RRID:SCR_018804) v0.3.0 [24] to correct errors in PacBio long reads, according to PacBio short reads (<5 kb) and then generated consensus sequences. The primary scaffolds were assembled with these subreads, and the scaffolds were further corrected in PILON (Pilon, RRID:SCR_014731) v1.22 [25] using short sequencing reads. To anchor scaffolds onto chromosomes, we aligned the Hi-C sequencing data to the assembly using BWA (BWA, RRID:SCR_010910) v0.7.17 [26] and detected valid contacts. Preassembled scaffolds were clustered, ordered, and directed onto pseudochromosomes using the LACHESIS software (LACHESIS, RRID:SCR_017644) [27]. To increase the accuracy of the assembled genome, we artificially corrected the LACHESIS-based assembly, filled gaps, and removed duplicate sequences [28].

Genome size was estimated based on k-mer distribution analysis with the program in GCE (GCE, RRID:SCR_017332) [29] using Illumina short reads. We used Benchmarking Universal Single-Copy Orthologs (BUSCO, RRID:SCR_015008) v5 [30] and Core Eukaryotic Genes Mapping Approach (CEGMA, RRID:SCR_015055) [31] to evaluate the completeness and accuracy of the genome assembly and BWA v0.7.17 [26] to align the Illumina short reads to the assembly and evaluate the assembled portion. We assembled expressed sequencing tags (ESTs) using Trinity (Trinity, RRID:SCR_013048) v2.8.4 [32] with RNA reads from seven different tissues (heart, liver, spleen, lung, kidney, muscle, and intestine). To further examine the quality of the genome assembly, we aligned ESTs to the assembled genome using BLAT (BLAT, RRID:SCR_011919) [33] with parameters of identity ≥90% and coverage ≥50%. The quality of the genome assembly was evaluated by mapping RNA-Seq reads

to the ESTs using TopHat2 (TopHat, RRID:SCR_013035) [34].

Repetitive elements annotation

Repetitive sequences were identified at both the DNA and protein levels by integrating homology-based prediction and *de novo* identification. Repeat masking was performed based on repeats in the Repbase TE library from the Repbase server (Repbase, RRID:SCR_021169) [35]. Using this library, we predicted interspersed repeat elements using RepeatMasker (RepeatMasker, RRID:SCR_012954) v3.3.0 and ProteinMask and screened tandem repeats using TRF v4.07b [36].

Gene prediction and functional annotation

We annotated protein-coding genes in the *P. larvata* genome using a combination of *de novo* gene prediction, homology-based prediction, and RNA-seq-based prediction. For *de novo* identification, we predicted the gene models using five *ab initio* gene prediction programs: Augustus (Augustus, RRID:SCR_008417) [37], GlimmerHMM (GlimmerHMM, RRID:SCR_002654) [38], SNAP (SNAP, RRID:SCR_002127) [39], Genscan (GENSCAN, RRID:SCR_013362) [40], and Geneid [41]. We compared proteins from five sequenced animals, namely, *Mus musculus* [42], *Canis lupus familiaris* [43], *Felis catus* [44], *Panthera tigris altaica* [45], and *Acinonyx jubatus* [46], to the *P. larvata* assembly using tBlastN (TBLASTN, RRID:SCR_011822) [47] with an e-value cut-off of 1×10^{-5}. Gene models were generated using GeneWise (GeneWise, RRID:SCR_015054) v2.2.0 [48]. We applied two methods of RNA-Seq-based prediction: (i) we mapped RNA-Seq data to the genome using TopHat2 [49] and further generated the gene models in Cufflinks (Cufflinks, RRID:SCR_014597) [34] based on exons, and (ii) we aligned ESTs against the assembly to generate the gene models using PASA (PASA, RRID:SCR_014656) [50]. We integrated predictions from the three approaches in EVidenceModeler (EVidenceModeler, RRID:SCR_014659) v1.1.1 [51] to generate a non-redundant gene model set. The final gene models were annotated using the non-redundant protein database of NCBI (NR) and Swissprot, Kyoto Encyclopedia of Genes and Genomes (KEGG) [52], Interprot [53], and Pfam databases v14.1 [54].

Noncoding RNAs include tRNA, rRNA, miRNA, and snRNA. tRNAscan-SE (tRNAscan-SE, RRID:SCR_010835) v1.4 was used to find the tRNA sequence in the genome according to the structural characteristics of tRNA. rRNA is highly conserved, and it is possible to select a closely related species as the reference sequence, and rRNA in the genome is searched by Blast v2.2.26 [47]. The INFERNAL (Infernal, RRID:SCR_011809) software that comes with Rfam (Rfam, RRID:SCR_007891) v14.1 [54] was used predict the miRNA and snRNA in the genome using the covariance model of the Rfam family.

Phylogenetic analysis and estimation of divergence time

We used OrthoFinder (OrthoFinder, RRID:SCR_017118) v2.5.2 [55] to identify orthologous genes from masked palm civet and 18 other species, including cat (*F. catus*), cattle (*Bos taurus*), dhole (*Cuon alpinus*), dog (*C. lupus familiaris*), human (*Homo sapiens*), leopard (*Panthera pardus*), lion (*Panthera leo*), lynx (*Lynx canadensis*), mouse (*M. musculus*), opossum (*Monodelphis domestica*), panda (*Ailuropoda melanoleuca*), pig (*Sus scrofa*), polarbear (*Ursus maritimus*), rabbit (*Oryctolagus cuniculus*), red fox (*Vulpes vulpes*), tiger (*P. tigris altaica*), wolf (*Canis lupus*), and yak (*Bos mutus*) (Supplementary Table S1). Diamond was used for sequence similarity searches. We aligned the coding region and amino acid sequences of single-copy orthologous genes using MUSCLE (MUSCLE, RRID:SCR_011812) v3.7 [56]. All alignments were concatenated to a super-alignment matrix. Based on this matrix, we used IQ-TREE (IQ-TREE, RRID:SCR_017254) v1.6.10 [57] which automatically detect the best model and then perform a maximum-likelihood (ML) phylogenetic tree based on this best model. 1,000 bootstrap replicates were performed to obtain confidence values for the branches. Bayesian Evolutionary Analysis Sampling Trees (BEAST, RRID:SCR_010228) v2.6.0 [58] was used to estimate species divergence times based on amico acid sequences of single-copy genes. The tree topology based on IQ-TREE v1.6.10 [56] was used as starting tree, while splits between tiger and leopard (7.4 Ma), cat and tiger (15.2 Ma), polar bear and giant bear (23.4 Ma), pig and yak (62.0 Ma), mouse and human (90.0 Ma), as well as human and tiger (96.0 Ma) from TimeTree (TimeTree, RRID:SCR_021162) as calibration. We ran the Markov chain Monte Carlo

for 10 million generations with sampling every 1000 generations, and 25% of the generations were discarded as burn-in. TreeAnnotator in BEAST v2.6.0 [58] was used to summarize the information from trees produced by BEAST v2.6.0 [58] onto a single target tree, while the target tree was visualized and exported as vector diagrams using FigTree (FigTree, RRID:SCR_008515) v1.4.4.

Gene family expansion and contraction analysis

To identify gene family evolution as a random birth and death process model, where the gene family either expands or contracts per gene per million years independently along each lineage of the phylogenetic tree, the ML model originally implemented in the software package CAFE (CAFE, RRID:SCR_005983) v4.2.1 [59] was applied to compare the cluster size differences (gain or loss) between the ancestor and each species. We used species tree with divergence time obtained by BEAST v2.6.0 [58] as ultrametric tree. We determined gene family significantly expansions or contractions when the P value was less than 0.05.

Positive selection analysis

For positive selection analysis, we first identified single-copy orthologous genes from *P. larvata*, *C. lupus familiaris,* and the five most closely related species from the Felidae family with assembled genomes: *F. catus*, *P. pardus*, *P. leo*, *L. canadensis*, and *P. tigris altaica.* For these genes, based on phylogenetic topology, we employed the branch-site model incorporated in the PAML package (PAML, RRID:SCR_014932) v4.9 [60] to detect positively selected genes (PSGs). *P. larvata* was specified as a foreground branch, the other species were used as background branches. To determine genes under positive selection, likelihood ratio tests were conducted to determine whether positive selection was operating on the foreground branch. In this study, PSGs were identified only when $P < 0.01$. Receptors were important factors to intermediate virus infection. To study the immune mechanism of *P. larvata*, we looked out 28 known human virus receptors [61] in positive selected genes.

Sequence analysis of virus receptors under positive selection and *ACE2*

Sequences of virus receptor genes under positive selection and the *ACE2* gene in other animal species of cat (*F. catus*), cattle (*B. taurus*), dhole (*C. alpinus*), dog (*C. lupus familiaris*), human (*H. sapiens*), leopard (*P. pardus*), lion (*P. leo*), lynx (*L. canadensis*), mouse (*M. musculus*), opossum (*M. domestica*), panda (*A. melanoleuca*), pig (*S. scrofa*), polarbear (*U. maritimus*), rabbit (*O. cuniculus*), red fox (*V. vulpes*), tiger (*P. tigris altaica*), and yak (*B. mutus*) were downloaded from NCBI (https://www.ncbi.nlm.nih.gov/). Though *ACE2* may not positive selected in *P. larvata*, it is receptor of SARS-CoV and SARS-CoV-2, both resulting in severe pneumonia in human beings. Therefore, we want to know the genetic relationship of virus receptors between *P. larvata* and other animals. Alignment of amino acid sequences of receptors was conducted using MUSCLE v3.7 [56]. ML phylogenetic trees were constructed using IQ-TREE v1.6.10 [57], and 1,000 bootstrap replicates were performed to obtain confidence values for the branches. The sequence identity of amino acid sequences which could demonstrate the genetic distance between receptor genes in different animals was estimated by p-distance in MEGA-X [62].

Results and Discussion

Genome sequencing and assembly

We sequenced the genome of one female masked palm civet (2n = 44) (Figure 1a). Here, 557.75 Gb sequences were generated from high-quality short-read sequences (272.68 Gb) and PacBio sequences (285.07 Gb), representing over 216× coverage for the genome. The genome has an estimated size of 2.58 Gb based on the 17-mer depth distribution analysis of the sequenced reads (Supplementary Figure S1) The final assembled sequence was 2.44 Gb, representing 94.6% of the masked palm civet genome. The assembly consisted of 1,785 scaffolds with an N50 of and 111.87 Mb (Table 1). We used the Hi-C technology to reorder and anchor 95.32% of the genome onto 22 pseudochromosomes (Figure 1a, Supplementary Figure S2). The GC content and heterozygosity rate of the assembled masked palm civet genome were 42.4% and 0.46%, respectively. The completeness of our assembly was assessed by BUSCO and CEGMA. The BUSCO assessment showed that 96.5% of the 9,226 BUSCOs were assembled,

with only 1.3% duplication (Supplementary Table S2), while CEGMA assessment showed that 91.94% and 3.2% of 248 CEGs (Core Eukaryotic Genes) were complete and partially assembled, respectively (Supplementary Table S3). We also used BWA v0.7.17 [26] to align Illumina short reads to the assembly and obtained a mapping rate and genome coverage of 99.35% and 99.73%, respectively. Overall, all evidence suggests that the *P. larvata* assembly is a high-quality genome assembly that can be used for downstream analyses.

Repeat annotation and gene prediction

We analyzed repetitive sequences by combining *de novo* prediction and a homology-based search at both the DNA and protein levels, and found that repetitive sequences occupied 31.80% (775.0 Mb) of the genome of the masked palm civet (Table 1, Figure 1a), which was very similar to the repeat compositions of the cat genome (32.10%), but lower than that of the tiger genome (39.20%) [44, 45]. The most abundant repetitive element type in the masked palm civet genome was long terminal repeat (LTR), representing 542.79 Mb (22.27%) of the genome. An annotation pipeline combining *de novo*, homolog-based search, and RNA-Seq methods was performed to predict gene models from the repeat-masked masked palm civet genome sequence. These analyses predicted 21,582 protein-coding genes, representing 30.61% of the genome assembly (Supplementary Table S4, Supplementary Figure S3a). Among the 21,582 genes, 20,546 (95.20%) were successfully annotated for at least one function term by searching against functional databases (Gene Ontology (GO), InterProScan, KEGG, NR, Pfam, and SwissProt) (Table 1, Supplementary Figure S3b). In addition to the protein-coding genes, we also identified 14,829 miRNA genes, 47,575 tRNA genes, and 544 rRNA genes in the masked palm civet genome (Supplementary Table S5). The number of miRNA and tRNA genes seemed higher than other species. We double checked the methods and conducted noncoding RNAs prediction again, and obtained the same results. Maybe the high level of miRNA and tRNA genes is the personality of this species, which need further analysis in the future.

Gene family comparison

The evolutionary dynamics related to *P. larvata* were deduced by comparison with ten animal species. We identified 1,438 one-to-one orthologs among these species (Figure 1b; Supplementary Table S6). Among the 19 species, 119 genes belonging to 44 gene families were unique in *P. larvata* (Supplementary Table S6). A immune-related gene named *VSIG10L*, which is related to familial Barrett neoplasia was contained in these genes [63]. Based on the P value threshold of 0.05, 35 gene families (including 243 genes) were significantly expanded; in contrast, 89 gene families (including 58 genes) were significantly contracted in the *P. larvata* genome (Figure 1c). There were 10 immune-related genes in significantly expanded genes, which were annotated to 3 genes of *TARM1* (T cell-interacting, activating receptor on myeloid cells-1), *IGLL1* (immunoglobulin lambda like polypeptide 1), and *OSCAR* (osteoclast associated Ig-like receptor) (Supplementary Table S7). *TARM1* is a novel leukocyte receptor complex which could encoded ITAM receptor that costimulates proinflammatory cytokine secretion by macrophages and neutrophils [64]. *TARM1* enables immunoglobulin receptor binding activity. *IGLL1* is a protein coding gene. Diseases associated with *IGLL1* include Agammaglobulinemia 2, Autosomal Recessive and Lambda 5 Deficiency [65]. *OSCAR* is a member of the leukocyte receptor complex protein family that plays critical roles in the regulation of both innate and adaptive immune responses [66]. The encoded protein may play a role in oxidative stress-mediated atherogenesis as well as monocyte adhesion. Recent studies showed that *OSCAR* can promote proliferation and migration of lung adenocarcinoma in women [67]. We did not detcted immune-related genes in significantly contracted genes, which was in accordance with the high immune response level of masked palm civets.

Phylogenetic Analysis

To confirm the phylogenetic position of *P. larvata* and estimate divergence time using whole-genome data, we analyzed the orthologous gene relationships between *P. larvata* and 18 other animals (Supplementary Table S1). Based on a super alignment matrix of amino acid sequences and coding region sequences of 1,438 single-copy genes with opopssum as outgroup. The best model of cds and amino acid sequences

were GTR+F+R3 and JTT+F+R5. Both trees shared the same topology that the genetic relationship between *P. larvata* and 5 species belong to the Felidae family were closely related. The phylogenetic tree based on amino acid sequences was used as starting tree to calculate the divergence time, which resulted the time of *P. larvata* split with the Felidae family was approximately 33.5 million years ago (Figure 1c). This is the first time of obtaining the divergence time of masked palm civets based on whole genome sequences.

Genes under positive selection

We used *P. larvata* as the foreground branch, and other species of *C. lupus familiaris, F. catus, P. pardus, P. leo, L. canadensis,* and *P. tigris altaica* as background branches to detect genes under selection. Based on 5,696 single-copy genes of these seven species, we identified 971 PSGs ($P < 0.01$; Supplementary Table S9). We did not observe genes under positive selection overlapped with expanded and contracted genes. One of these known human viral receptor genes, *PDGFRA*, were included in genes under positive selection in *P. larvata*. *PDGFRA* could activating mutations in gastrointestinal stromal tumors [68], and is required for human cytomegalovirus (HCMV) infection [69, 70]. There is no study focus on *PDGFRA* receptor in masked palm civets, further study is needed. As an important receptor intermediate the infection of SARS-CoV and SARS-CoV-2, *ACE2* receptor was involved in this study though it is not under positive selection. The phylogeny of *PDGFRA*, and *ACE2* receptors from different animal species was conducted based on amino acid sequences downloaded from NCBI. The relationship of *PDGFRA* and *ACE2* in *P. larvata* was closest to four most closely related species from the Felidae family of *F. catus*, *P. pardus*, *L. canadensis*, and *P. tigris altaica* (Figure 2). The amino acid sequence identities of *PDGFRA* and *ACE2* were also highest among *P. larvata*, *F. catus*, *P. pardus*, *L. canadensis*, and *P. tigris altaica* (Table 2), demonstrating viruses using *PDGFRA* and *ACE2* might easily transferred between these species. However, further research is needed to reveal the differences in the receptor-binding loci of these receptors in *P. larvata* and other species, as well as the immunoreaction of cells after virus infection in consideration of different severity of symptoms in different species. Besides virus

receptors, other ten immune-related genes were also under selection (Supplementary Table S10). While this paper was under review another study has been published on the *P. larvata* genome[71]. On top of these efforts further studies are needed to reveal the roles of these immune-related genes under positive selection in civet immunity.

Conclusion

In this study, we present a high-quality chromosome-level genome assembly of *P. larvata* using PacBio single-molecule long-read sequencing, supplemented with Illumina short-read sequencing and Hi-C sequencing. The final genome assembly was 2.44 Gb, 95.32% of which could be grouped into 22 pseudochromosomes. This assembly is highly contiguous with contig N50 and scaffold N50 values of 12.97 Mb and 111.87 Mb. The repeat sequence comprised 31.80% of the genome. A total of 21,582 protein-coding genes were predicted, 95.20% of which could be functionally annotated. Phylogenetic analysis based on 1,438 single-copy orthogroups across 19 animals resulted in a close genetic relationship between *P. larvata* and five species from the Felidae family. The divergence time of *P. larvata* split from the Felidae family was estimated to be 33.5 Ma. Gene family clustering revealed 119 unique, 243 significantly expanded, and 58 significantly contracted genes in *P. larvata* genome. We identified 971 genes under positive selection in *P. larvata*, among which one known human viral receptor genes were found. The high-quality genome assembly will provide a valuable genomic resource for elucidating the immune mechanisms in this animal. It will also provide a reliable reference for studying the genetic bases of the morphological characteristics, adaptive evolution, and evolutionary history of this species.

Data Availability

Raw sequencing data and genome assembly were deposited at the NCBI under the BioProject PRJNA756156 with biosample accession number SAMN20856571. The annotations and predicted peptides are available in FigShare [72]. All other supporting data and materials are available in the *GigaScience* GigaDB database [73]

Competing Interests

The authors declare that they have no competing interests.

Funding

This project was supported by the surveillance of wild animals and epidemic diseases in important ecological areas of Guangdong Province project from the Forestry Administration of Guangdong Province, GDAS Special Project of Science and Technology Development (2020GDASYL-20200103090).

Acknowledgements

The sequencing in this study was conducted by the Novogene Company (Beijing, China). Thanks for the hard work of Hao Lv and her colleagues. Thanks for the helpful advices of Chen Feng and Chao Feng from South China Botanical Garden, Chinese Academy of Sciences in data analysis.

Figure Legends

Figure 1 (a) Synteny of corresponding chromosomes and gene density across the *Paguma larvata* genome; (b) Comparison of copy numbers in gene clusters reside in the genomes of *P. larvata* and other 18 animals. Single-copy orthologs denote the family can have only one gene for each species, and multicopy orthologs denote the family clustered more than one gene for each species. Other orthologs denote the family can have any number of genes for each species except the single-copy and multi-copy orthologs. Unique paralogs denote species-specific gene families, and unclustered genes denote species-specific genes that cannot cluster with any other genes; (c) Phylogenetic relationships of 19 animal genomes based on the maximum likelihood method. The black numbers indicate estimated divergent times as the number of years (millions) ago, while green and red numbers denote gene families subject to expansion and contraction for each species, respectively.

Figure 2 Phylogenetic relationships of known human virus receptor of *PDGFRA* under positive selection and *ACE2* receptor in *P. larvata* and other species.

References

1. Iwami S, Takeuchi Y, Liu X. Avian flu pandemic: Can we prevent it?. J Thero Biol 2009;**257**(1):181-190.
2. Siddiqui AA. The global threat of bird (Avian) flu its treatment methods and public health preventive measures. SOJ Vet Sci 2018; **4**(3):1-4.
3. Dixon L K, Sun H, Roberts H. African swine fever. Antivir Res 2019;**165**:34-41.
4. Rota PA, Oberste MS, Monroe SS, et al. Characterization of a novel coronavirus associated with severe acute respiratory syndrome. Science 2003;**300**(5624):1394-1399.
5. Zumla A, Hui D S, Perlman S. Middle East respiratory syndrome. The Lancet 2015;**386**(9997):995-1007.
6. Wu D, Wu T, Liu Q, et al. The SARS-CoV-2 outbreak: what we know. Int J Infect Dis 2020;**94**:44-48.
7. Sabir JSM, Lam TTY, Ahmed MMM, et al. Co-circulation of three camel coronavirus species and recombination of MERS-CoVs in Saudi Arabia. Science 2016;**351**(6268):81-84.
8. Inoue T, Kaneko Y, Yamazaki K, et al. Genetic population structure of the masked palm civet *Paguma larvata*,(Carnivora: Viverridae) in Japan, revealed from analysis of newly identified compound microsatellites. Conserv Genet 2012;**13**(4):1095-1107.
9. Guan Y, Zheng BJ, He YQ, et al. Isolation and characterization of viruses related to the SARS coronavirus from animals in southern China. Science 2003;**302**(5643):276-278.
10. Li W, Shi Z, Yu M, et al. Bats are natural reservoirs of SARS-like coronaviruses. Science 2005;**310**(5748):676-679.
11. Wang LF, Eaton BT. Bats, civets and the emergence of SARS. Wildlife and emerging zoonotic diseases: the biology, circumstances and consequences of cross-species transmission, 2007:325-344.
12. Wan Y, Shang J, Graham R, et al. Receptor recognition by the novel coronavirus from Wuhan: an analysis based on decade-long structural studies of SARS coronavirus. J Virol 2020;**94**(7):e00127-20.

13. Marsh M, Helenius A. Virus entry: open sesame. Cell 2006;124(4):729-740.
14. Grove J, Marsh M. The cell biology of receptor-mediated virus entry. J Cell Biol 2011;**195**(7):1071-1082.
15. Li W, Moore MJ, Vasilieva N, et al. Angiotensin-converting enzyme 2 is a functional receptor for the SARS coronavirus. Nature 2003;**426**(6965):450-454.
16. Shi R, Shan C, Duan X, et al. A human neutralizing antibody targets the receptor-binding site of SARS-CoV-2. Nature 2020;**584**(7819):120-124.
17. Wu Y, Wang F, Shen C, et al. A noncompeting pair of human neutralizing antibodies block COVID-19 virus binding to its receptor ACE2. Science 2020;**368**(6496):1274-1278.
18. Bickhart DM, Rosen BD, Koren S, et al. Single-molecule sequencing and chromatin conformation capture enable de novo reference assembly of the domestic goat genome. Nat genet, 2017;**49**(4):643-650.
19. Mahajan S, Wei K, Nalley M, et al. De novo assembly of a young Drosophila Y chromosome using Single-Molecule sequencing and Chromatin Conformation capture. PLoS Biol 2018;**16**:1-28.
20. Zhang Z, Chen Y, Zhang J, et al. Improved genome assembly provides new insights into genome evolution in a desert poplar (*Populus euphratica*). Mol Ecol Resour 2020;**20**(3):781-794.
21. Rio DC, Ares M, Hannon GJ, et al. Purification of RNA using TRIzol (TRI reagent). Cold Spring Harbor Protocols, 2010;**2010**(6):pdb. prot5439.
22. Chin CS, Alexander DH, Marks P, et al. Nonhybrid, finished microbial genome assemblies from long-read SMRT sequencing data. Nat methods 2013;**10**(6):563-569.
23. Belton JM, McCord RP, Gibcus JH, et al. Hi‐C: a comprehensive technique to capture the conformation of genomes. Methods 2012;**58**(3):268-276.
24. Chin CS, Peluso P, Sedlazeck FJ, et al. Phased diploid genome assembly with single-molecule real-time sequencing. Nat methods 2016;**13**(12):1050-1054.
25. Walker BJ, Abeel T, Shea T, et al. Pilon: an integrated tool for comprehensive microbial variant detection and genome assembly improvement. PloS one 2014;9(11):e112963.

26. Li H, Durbin R. Fast and accurate short read alignment with Burrows-Wheeler transform. Bioinformatics 2009;**25**:175-1760.
27. Hariharan R, Toyama K. Project Lachesis: parsing and modeling location histories//International Conference on Geographic Information Science. Springer, Berlin, Heidelberg, 2004:106-124.
28. Burton JN, Adey A, Patwardhan RP, et al. Chromosome-scale scaffolding of de novo genome assemblies based on chromatin interactions. Nat Biotechnol 2013;**31**:1119-1125.
29. Liu B, Shi Y, Yuan J, et al. Estimation of genomic characteristics by analyzing k-mer frequency in de novo genome projects. 2013. http://arxiv.org/abs/1308.2012.
30. Manni M, Berkeley MR, Seppey M, et al. BUSCO update: novel and streamlined workflows along with broader and deeper phylogenetic coverage for scoring of eukaryotic, prokaryotic, and viral genomes. Mol Biol Evol 2021;**38**(10): 4647-s4654
31. Parra G, Bradnam K, Korf I. CEGMA: A pipeline to accurately annotate core genes in eukaryotic genomes. Bioinformatics 2007;**23**:1061-1067.
32. Grabherr MG, Haas BJ, Yassour M, et al. Full-length transcriptome assembly from RNA-Seq data without a reference genome. Nat Biotechnol 2011;**29**:644-652.
33. Kent WJ. BLAT—the BLAST-Like Alignment Tool. Genome Res 2002;12:656-664.
34. Trapnell C, Roberts A, Goff L, et al. Differential gene and transcript expression analysis of RNA-seq experiments with TopHat and Cufflinks. Nat Protoc 2012;**7**:562-578.
35. Bao W, Kojima KK, Kohany O. Repbase Update, a database of repetitive elements in eukaryotic genomes. Mob DNA 2015;**6**:4-9.
36. Benson G. Tandem repeats finder: A program to analyze DNA sequences. Nucleic Acids Res 1999;**27**:573-580.
37. Stanke M, Schöffmann O, Morgenstern B, et al. Gene prediction in eukaryotes with a generalized hidden Markov model that uses hints from external sources. BMC Bioinformatics 2006;**7**:1-11.
38. Majoros WH, Pertea M, Salzberg SL. TigrScan and GlimmerHMM: Two open

source ab initio eukaryotic gene-finders. Bioinformatics 2004;**20**:2878-2879.

39. Korf I. Gene finding in novel genomes. BMC Bioinformatics 2004;**5**:1-9.
40. Burge C, Karlin S. Prediction of complete gene structures in human genomic DNA. J Mol Biol 1997;**268**:78-94.
41. Guigó R, Knudsen S, Drake N, et al. Prediction of gene structure. J Mol Biol 1992;**226**:141-157.
42. Gnerre S, MacCallum I, Przybylski D, et al. High-quality draft assemblies of mammalian genomes from massively parallel sequence data. Proc Natl Acad Sci USA 2011;**108**:1513-1518.
43. Kirkness EF, Bafna V, Halpern AL, et al. The dog genome: Survey sequencing and comparative analysis. Science 2003;**301**(5641):1898-1903.
44. Pontius JU, Mullikin JC, Smith DR, et al. Initial sequence and comparative analysis of the cat genome. Genome Res 2007;**17**(11):1675-1689.
45. Cho Y S, Hu L, Hou H, et al. The tiger genome and comparative analysis with lion and snow leopard genomes. Nat Commun 2013;**4**(1):1-7.
46. Dobrynin P, Liu S, Tamazian G, et al. Genomic legacy of the African cheetah, *Acinonyx jubatus*. Genome Biol 2015;**16**(1):1-20.
47. Altschul SF, Gish W, Miller W, et al. Basic local alignment search tool. J Mol Biol 1990;**215**:403-410.
48. Birney E, Clamp M, Durbin R. GeneWise and Genomewise. Genome Res 2004;**14**:988-995.
49. Kim D, Pertea G, Trapnell C, et al. TopHat2: accurate alignment of transcriptomes in the presence of insertions, deletions and gene fusions. Genome biol 2013;**14**(4):1-13.
50. Haas BJ, Delcher AL, Mount SM, et al. Improving the Arabidopsis genome annotation using maximal transcript alignment assemblies. Nucleic Acids Res 2003;**31**:5654-5666.
51. Haas BJ, Salzberg SL, Zhu W, et al. Automated eukaryotic gene structure annotation using EVidenceModeler and the Program to Assemble Spliced Alignments. Genome biol 2008;**9**(1):1-22.
52. Kanehisa M, Sato Y, Kawashima M, et al. KEGG as a reference resource for gene

and protein annotation. Nucleic Acids Res 2016;**44**:457-462.

53. Quevillon E, Silventoinen V, Pillai S, et al. InterProScan: protein domains identifier. Nucleic Acids Res 2005;**33**:116-120.
54. Finn RD, Coggill P, Eberhardt RY, et al. The Pfam protein families database: towards a more sustainable future. Nucleic Acids Res 2016;**44**(D1):D279-D285.
55. Emms DM, Kelly S. OrthoFinder: Solving fundamental biases in whole genomecomparisons dramatically improves orthogroup inference accuracy. Genome Biol 2015;**16**: 157.
56. Edgar RC. MUSCLE: Multiple sequence alignment with high accuracy and high throughput. Nucleic Acids Res 2004;**32**:1792-1797.
57. Nguyen LT, Schmidt HA, Von Haeseler A, et al. IQ-TREE: a fast and effective stochastic algorithm for estimating maximum-likelihood phylogenies. Mol Biol Evol 2015;**32**(1):268-274.
58. Bouckaert R, Heled J, Kühnert D, et al. BEAST 2: a software platform for Bayesian evolutionary analysis. PLoS comput biol, 2014;**10**(4):e1003537.
59. De Bie T, Cristianini N, Demuth JP, et al. CAFE: A computational tool for the study of gene family evolution. Bioinformatics 2006;**22**:1269-1271.
60. Yang Z. PAML 4: Phylogenetic analysis by maximum likelihood. Mol Biol Evol 2007;**24**:1586-1591.
61. Ren L, Wu C, Guo L, et al. Single-cell transcriptional atlas of the Chinese horseshoe bat (*Rhinolophus sinicus*) provides insight into the cellular mechanisms which enable bats to be viral reservoirs. bioRxiv 2020. DOI:10.1101/2020.06.30.175778
62. Kumar S, Stecher G, Li M, et al. MEGA X: molecular evolutionary genetics analysis across computing platforms. Mol Biol Evol 2018;**35**(6):1547.
63. Fecteau R E, Kong J, Kresak A, et al. Association between germline mutation in VSIG10L and familial Barrett neoplasia. JAMA Oncol 2016;**2**(10):1333-1339.
64. Radjabova V, Mastroeni P, Skjødt K, et al. TARM1 Is a Novel Leukocyte Receptor Complex – Encoded ITAM Receptor That Costimulates Proinflammatory Cytokine Secretion by Macrophages and Neutrophils. J Immunol 2015;**195**(7):3149-3159.

65. Gemayel KT, Litman GW, Sriaroon P. Autosomal recessive agammaglobulinemia associated with an IGLL1 gene missense mutation. Ann Allerg, Asthma Im 2016;**117**(4):439-441.
66. Kim N, Takami M, Rho J, et al. A novel member of the leukocyte receptor complex regulates osteoclast differentiation. J Exp Med 2002;**195**(2):201-209.
67. Wang D, Yang N, Xie S. Sex‑biased ceRNA networks reveal that OSCAR can promote proliferation and migration of lung adenocarcinoma in women. Clin Exp Pharmacol P 2020;**47**(8):1350-1359.
68. Heinrich M C, Corless C L, Duensing A, et al. PDGFRA activating mutations in gastrointestinal stromal tumors. Science 2003;**299**(5607):708-710.
69. Soroceanu L, Akhavan A, Cobbs CS. Platelet-derived growth factor-α receptor activation is required for human cytomegalovirus infection. Nature 2008;**455**(7211):391-395.
70. Yang Z, Tang X, McMullen T P W, et al. PDGFRα Enhanced Infection of Breast Cancer Cells with Human Cytomegalovirus but Infection of Fibroblasts Increased Prometastatic Inflammation Involving Lysophosphatidate Signaling. Int J Mol Sci 2021;**22**(18): 9817.
71. Lan T, Fang D, Li H, Sahu SK, Wang Q, Yuan H, Zhu Y, Yang Z, Zhang L, Yang S, Lu H, Han L, Zhang S, Yu J, Mahmmod YS, Xu Y, Hua Y, He F, Yuan Z, Liu H. Chromosome-Scale Genome of Masked Palm Civet (*Paguma larvata*) Shows Genomic Signatures of Its Biological Characteristics and Evolution. Front Genet. 2022 Jan 21;12:819493. doi: 10.3389/fgene.2021.819493.
72. Liu P (2022): pasa2.longest.filter.gff3. figshare. Dataset. https://doi.org/10.6084/m9.figshare.15273879.v1
73. Liu P, Jiang H, Li L, Zhou J, Huang W, Chen J. Supporting data for "The Assembled and Annotated Genome of the Masked Palm Civet (*Paguma larvata*)" GigaScience Database. 2022. http://doi.org/10.5524/102211

Tables

Table 1 *Paguma larvata* Genome Assembly and Annotation Summary.

Genome assembly statistics	
Total length (bp)	2,437,385,791
Contig/Scaffold N50 length (bp)	12,971,789
Contig/Scaffold N90 length (bp)	2,389,527
Longest contig length (bp)	52,858,878
Number of contigs	1,785
Number of contigs larger than N50	56
Number of contigs larger than N90	210
Genome characteristics	
GC content	42.41%
Number of protein-coding genes	21,582
Average transcript length (bp)	34,567.54
Average CDS length (bp)	1,481.00
Average exon length (bp)	170.53
Average exons per gene (bp)	8.68
Repetitive sequences (% of genome)	
DNA (bp)	8,232,288 (0.34%)
LINE (bp)	299,126,376 (12.27%)
SINE (bp)	613,247 (0.03%)
LTR (bp)	542,789,387 (22.27%)
Unknown (bp)	12,280,567 (0.5%)
Total (bp)	775,001,321 (31.8%)
Gene annotations (% of all genes)	
GO	14,148 (65.6%)
InterProScan	19,770 (91.6%)
KEGG	18,200 (84.3%)
Nr	20,480 (94.9%)
PFAM	17,505 (81.1%)
SwissProt	20,033 (92.8%)
Total	21,582

Table 2 Sequence identity of receptors under positive selection between masked palm civet and other animal species.

**Species**	**Sequence identity (%)**	
	PDGFRA	ACE2
Lynx canadensis	**97.95**	**93.17**
Felis catus	**97.85**	**92.67**
Panthera tigris altaica	**97.85**	**92.60**
Panthera pardus	**97.67**	**92.80**
Canis lupus familiaris	96.55	88.93
Vulpes vulpes	96.55	88.81
Ursus maritimus	95.99	89.94
Ailuropoda melanoleuca	95.24	89.79
Homo sapiens	95.05	83.35
Sus scrofa	94.86	82.48
Manis javanica	93.54	85.47
Bos taurus	93.18	81.84
Bos mutus	93.09	81.84
Desmodus rotundus	92.16	79.35
Mus musculus	90.01	81.74
Oryctolagus cuniculus	88.14	83.11
Monodelphis domestica	83.49	71.14

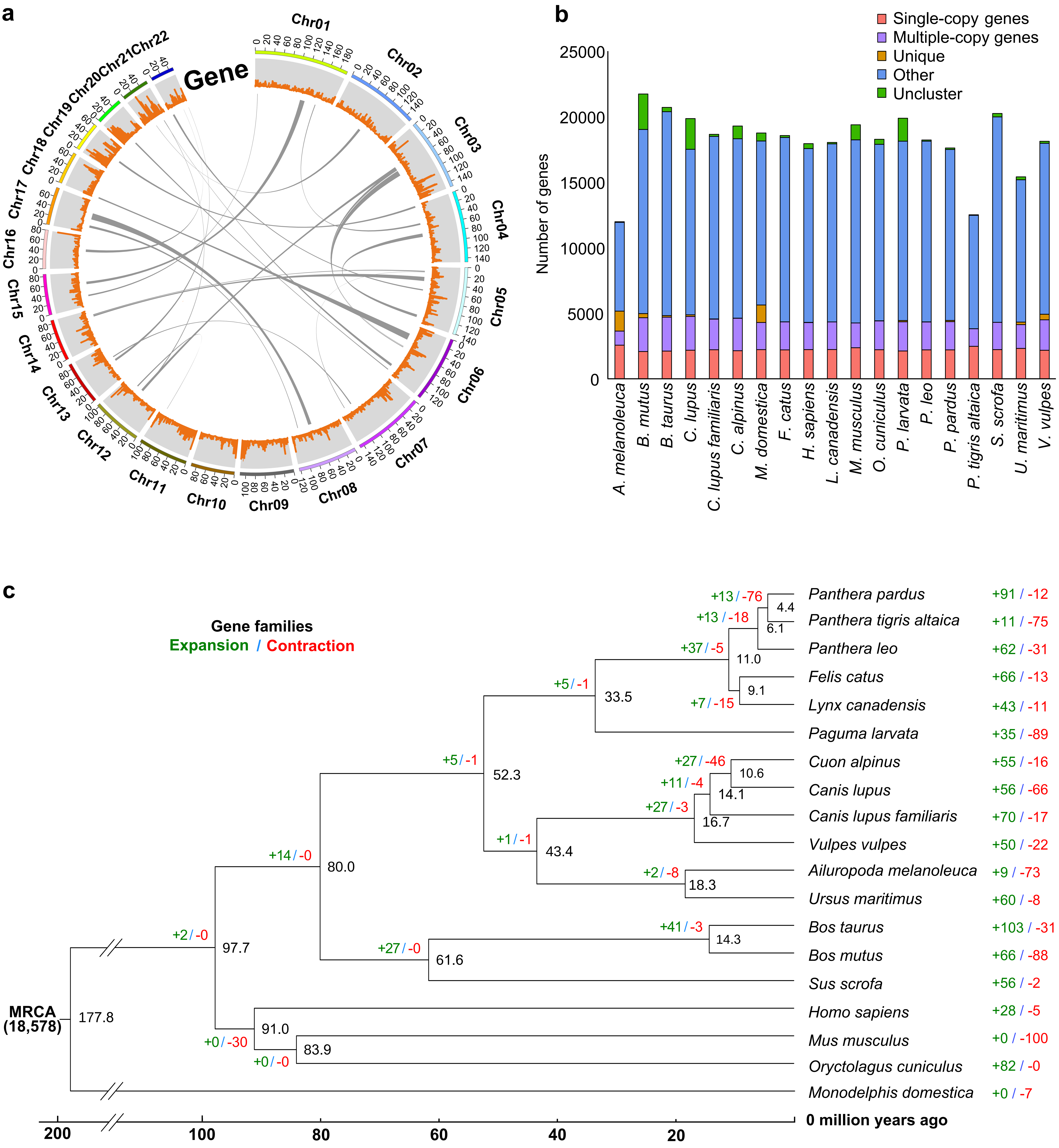

a
Gene
Chr01
Chr02
Chr03
Chr04
Chr05
Chr06
Chr07
Chr08
Chr09
Chr10
Chr11
Chr12
Chr13
Chr14
Chr15
Chr16
Chr17
Chr18
Chr19
Chr20
Chr21
Chr22
b
Single-copy genes
Multiple-copy genes
Unique
Other
Uncluster
Number of genes
25000
20000
15000
10000
5000
0
A. melanoleuca
B. mutus
B. taurus
C. lupus
C. lupus familiaris
C. alpinus
M. domestica
F. catus
H. sapiens
L. canadensis
M. musculus
O. cuniculus
P. larvata
P. leo
P. pardus
P. tigris altaica
S. scrofa
U. maritimus
V. vulpes
c
Gene families
Expansion / Contraction
Panthera pardus +91 / -12
Panthera tigris altaica +11 / -75
Panthera leo +62 / -31
Felis catus +66 / -13
Lynx canadensis +43 / -11
Paguma larvata +35 / -89
Cuon alpinus +55 / -16
Canis lupus +56 / -66
Canis lupus familiaris +70 / -17
Vulpes vulpes +50 / -22
Ailuropoda melanoleuca +9 / -73
Ursus maritimus +60 / -8
Bos taurus +103 / -31
Bos mutus +66 / -88
Sus scrofa +56 / -2
Homo sapiens +28 / -5
Mus musculus +0 / -100
Oryctolagus cuniculus +82 / -0
Monodelphis domestica +0 / -7
+13 / -76
4.4
+13 / -18
6.1
+37 / -5
11.0
+5 / -1
33.5
9.1
+7 / -15
+5 / -1
52.3
+27 / -46
10.6
+11 / -4
14.1
+27 / -3
16.7
+1 / -1
43.4
+2 / -8
18.3
+14 / -0
80.0
+41 / -3
14.3
+2 / -0
97.7
+27 / -0
61.6
MRCA
(18,578)
177.8
91.0
+0 / -30
83.9
+0 / -0
200
100
80
60
40
20
0 million years ago

Click here to access/download;Figure;Figure 2-aa_trees.pdf

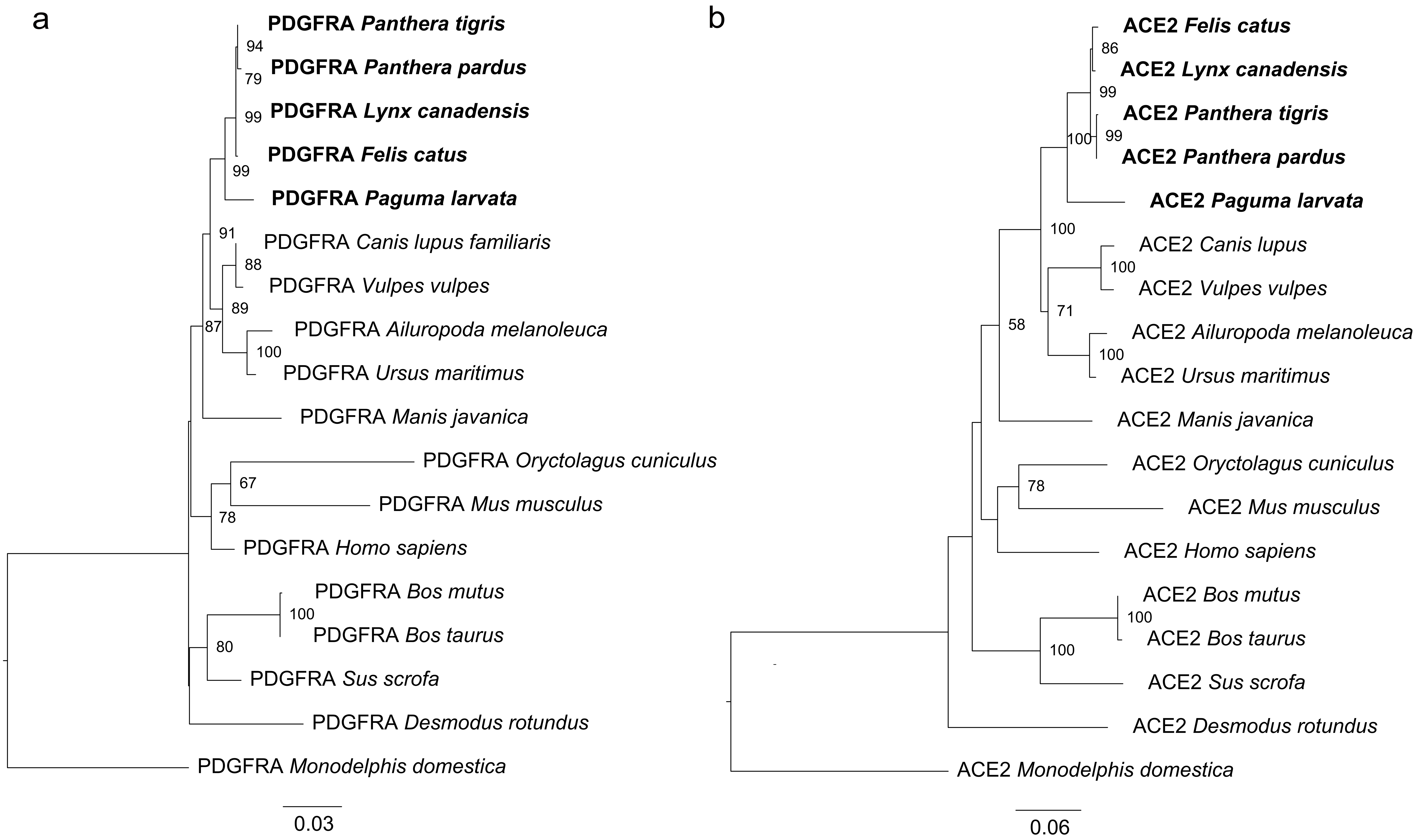

Supplementary tables

Click here to access/download
Supplementary Material
Supplementary Tables-20220210.xlsx

Supplementary figures

Click here to access/download
Supplementary Material
Supplementary Figures-20220210.docx

Cover letter

Click here to access/download
Supplementary Material
cover letter.docx

Commonts

Click here to access/download
Supplementary Material
Commonts-20220310.docx
