# Supplementary material for: The assembled and annotated genome of the masked palm civet (Paguma larvata)
Source: Gigascience. 2022 May 18;11:giac041. doi: 10.1093/gigascience/giac041 (PMC9116208; doi:10.1093/gigascience/giac041)
Supplement: giac041_Response_to_Reviewer_Comments_Original_Submission [file giac041_response_to_reviewer_comments_original_submission.pdf]

Author's Response To Reviewer Comments

Close

Reviewer reports:

Reviewer #1: Liu et al. reported a chromosome-level genome assembly of Paguma larvata, which will provide a valuable genomic resource in investigating virus and host interactions because P.larvata was found to be an important host of SARS-Cov. The methods of genome assembly and gene prediction are appropriate; the continuity, completeness, gene number from the annotation of the P. larvata genome are also up to standard. Some suggestions are given for improving the quality of the manuscript.

1. To obtain reliable and correct results, the closely-related relatives of the masked palm civet, rather than Gallus, should be used as the outgroup in evolutionary analyses, especially the comparison of the evolutionary dynamics of gene families.
Reply: Thanks for your precious advice. We understand your opinion that the genetic relationship between gallus and ingroups is too distant. Therefore, in the new version of manuscript, we used opossum as outgroup, which is more closely related to ingroups than Gallus.

2. Because the authors simultaneously examined the positive selection of several genes, the results of statistical tests should be corrected by multiple testing.
Reply: Your advice is perfectly right. Actually, we used branch-site model to determine genes under positive selection, and likelihood ratio tests were conducted to determine whether positive selection was operating on the foreground branch. In this study, PSGs were identified only when $P < 0.01$. I am sorry that we didn’t mention this in our manuscript before. We had added these details in “Positive selection analysis” section.

3. The gene IDs used in Supplementary Table S8 are not the gene symbol name, and it is better to annotate the gene symbol names to facilitate data utilization.
Reply: We are sorry for the mistake, we had added the gene symbol name in Supplementary Tables.

4. Line 157-159: Given that only five species were used for the homology-based prediction, I suggested adding a couple of mammalian species with high genome annotation quality, such as Homo sapiens, in this analysis, which will be helpful to further improve the annotation quality.
Reply: Thanks for your advice. The improvement of annotation quality is better to be annotated by adding more mammalian species. However, We annotated protein-coding genes in the Paguma larvata genome using a combination of de novo gene prediction, homology-based prediction, and RNA-seq-based prediction (Supplementary Table S4),which can help to get more annotated genes, and the results of downstream analyses based on these genes are all fine.

5. Please provide the versions of the softwares and the parameters used in the Methods part.
Reply: Thanks for your advice. The versions of softwares and the parameters are added in the Methods part of our manuscript.

6. line22, "with contig N50 and scaffold N50 values of 12.97 Mb, respectively" the authors seem to miss some words or values.
Reply: I am sorry that we did miss the scaffold N50 of 111.87 Mb, which has been added in the manuscript.

Reviewer #2: Reviewer's Comments
In this study, the authors provide a high-quality assembly of the Masked Palm Civet. This genome will be a valuable resource for comparative genomics. While the overall study is good, I felt that it lacked a cohesive narrative, with certain results either not making sense as written, or results being mentioned in passing but not explored further. I also found that certain paragraphs suffered from issues with grammar, such that the true meaning may be lost. Certain analyses e.g. RNA seq are mentioned, but never used to validate results. I believe there are major revisions needed for this study. Please see a list of major and minor comments below:

Minor comments
Line 44: The authors say posed as if it longer poses a threat, however I think this should be 'pose'.
Reply: Yes, we have corrected this word.

Line 50: Saying in recent decades but that something used to be domesticated almost seemed contradictory to me, but that might just be me.
Reply: Actually, the period of masked palm civet domesticated was short, just a few decades, so we said recent decades.

Line 90: I'm being a bit nit-picky here, but I feel like a final sentence or two explicitly stating why the genome was sequenced (e.g. to explore virus-host interactions) would be useful.
Reply: This is an excellent idea!

Line 122: I'm not sure if 'the FALCON' is the correct term to use here?
Reply: Yes, it is the correct term.

Line 139: you use the word 'using' twice in quick succession, perhaps consider something else?
Reply: Yes, your advice is precious. We have changed this sentence.

Line 215: a "." Is missing on this line.
Reply: Yes, it is, we have added a ".".

Line 300: How did the authors 'conduct' sequence identity? What is the importance of sequence identity compared to evidence of positive selection?
Reply: Sequence identity was calculated by p-distance in MEGA-X. The results may tell us the genetic relationship among different hosts. We focus on virus receptor genes under positive selection, which may be useful for study of tracing the origin and transmission of virus in the future.

Major comments

1. You mention that you aligned protein sequences using blast, however blast is not an alignment software. Am I missing something?
Reply: Thanks for your advice, we have changed the word of align to compare ?

2. What were the best-fit models of sequence evolution used with RAxML? Were they inferred? There are no mention of models used.
Reply: I am sorry that we did not mention these information in our manuscript. For cds sequences, we constructed phylogenetic tree based on GTRGAMMA model, while for protein sequences, we constructed phylogenetic tree based on PROTGAMMAAUTO model. Both trees shared the same topology. However, this information is about the old results. In our new results based on 19 species, we used IQ-TREE v1.6.10 which automatically detect the best model and then perform a maximum-likelihood (ML) phylogenetic tree for these species based on this best model.The best model of cds sequences calculated by IQ-TREE v1.6.10 was GTR+F+R3, while the best model of amino acid sequences was JTT+F+R5.

3. Were p-values from PAML corrected for multiple testing?
Reply: Branch-site model was used to determine genes under positive selection. Likelihood ratio tests were conducted todetermine whether positive selection was operating on theforeground branch. In this study, PSGs were identified only when$P < 0.01$.

4. The authors mention downloading sequences of receptors under selection and ACE2 from other animal species. It would be helpful if they listed them here. Is there a reason why the authors use mafft here but used muscle previously?
Reply: We are sorry about this mistake. We have listed other species that we get sequences of receptors under selection and ACE2. Actually, we used mafft and musclerandomly in different analysis, because we do not think there are differences between alignment results of them. In the new results, alignment of sequences was all conducted using muscle.

5. I am a little confused about the statement on line 255. The authors say that 87 gene families are unique to P. larvata. I find this number very difficult to believe. This would suggest that 87 different families of genes have subsequently emerged since sharing a last common ancestor with the cat and tiger. I think there could be a wording issue here. But based on these gene numbers, and the divergence

time quoted later (37.2 MY), that's one gene family per 427,586 with 'gene family' implying more than one gene. Can the authors clarify this?
Reply: I am sorry that we did not describe this question very clear. Actually, we means there are 196 unique genes in P. larvata, which belong to 87 gene families. These 87 gene family were not unique in P. larvata. It is worth noting that, in our new results, 119 genes were unique in P. larvata, and these genes are from 44 gene families.

6. Was selection detected in ACE2? If not, what does this mean for SARS-CoV in civets? I feel that the authors have avoided this but should discuss it in the text. What implications does this have for the theory that viral receptors in the civet have undergone change to avoid infection?
Reply: I am sorry that we did not clear this in our text. ACE2 did not positive selected in masked palm civet. However, in the backgroud of the ongong spread of SARS-CoV-2, which use ACE2 as receptor, we added analysis of this receptor in our text. The sequence similarity may reflect the potential of binding viruses to some extent. However, there are other factors, such as binding loci, could affect the virus binding ability. Actually, virus could infect civet cells, but no particularly severe symptoms appear. The mechanism of civet to resistant viruses is a complex question which need further study. In this study, we just studied this question based on the sequence of receptors.

7. The authors state that there were more protein coding genes in the civet than the tiger and cat. Do these genes come from the expansions of certain families, or are they completely absent in other species? Is it possible that alleles of genes from reverse strands have been incorporated in the genome? Do these extra genes show up in the RNA seq data?
Reply: I mentioned that there were more protein coding genes in civet than tiger and cat just want to say, the annotation results of civet is better than cat and tiger, two relatively related species. However, genome of these two species were generated in 2007 and 2013, about ten years ago, and maybe that is why the results are not perfect. In our new analysis, we used five species in the family of Felidae closely related to civet, and we realize the comparison with gene numbers makes no sense, and this sentence has been deleted.

8. Were any of the expanded immunoglobulin genes under selection? These seem to be mentioned once in the result but no more if mentioned about them.
Reply: We are sorry for missing these results. However, we did not observe genes under positive selection overlapped with expanded and contracted genes. This information has been added to “Gene family comparison” section of “Results” this manuscript.

Reviewer #3: The manuscript by Liu et el reports the assembled and annotated genome of the masked Palm Civet (Paguma larvata) which is a good contribution given the role of this organism in acting as an intermediate host of severe acute respiratory syndrome coronavirus (SARS-CoV). The assembly is performed well and is of acceptable quality, however I have some major comments on the analysis part which are mentioned below.

i) The reported genome assembly of Paguma larvata is of good reference quality achieved using short read, long read and Hi-C technology. But despite of having an N50 value of 12.97 Mb, the BUSCO completeness is only 95 % which much lower than expected. Do the authors have an explanation for it or I suggest that it should be carried out again.
Reply: Thanks for your advice! As for the BUSCO result, we carried out again using BUSCO v5 (95% was resulted from BUSCO v3), and 96.5% of the 9,226 BUSCOs were assembled. I don’t know whether this new results could make sense.

ii) The methodology for prediction of non-coding RNAs should be mentioned in the main manuscript. In line 248-250, I noted that the number of predicted miRNA, tRNA genes seems to be much higher in this genome, which should be carefully checked.
Reply: The methods for prediction of non-coding RNAs have been added in “Gene prediction and functional annotation” section. We analyzed non-coding RNAs prediction again, and the same results were obtained. We are also confused about this. We also checked results of BUSCO, complete and duplicated BUSCOs is 1.3%, meaning there is not data Redundancy. Maybe the high miRNA and tRNAis the personality of this species.

iii) RNA-Seq data was generated from seven different tissues of Paguma larvata. This amount of data can be used for more detailed analysis e.g., differential gene expression profile of immunity-related genes in these different tissues. This analysis will add more value to the work.
Reply: We appreciate your advice. We did sequenced RNA-Seq data from seven different tissues, but

these tissues all from one individual. RNA-Seq data in this study was mainly used to conduct gene prediction. Actually, we planned to collect tissues in different individuals for RNA-Seq. However, after the break out of SARS-CoV-2, experiments on wild animals such as civet who may related to this virus are carried out with great caution. Therefore, we have to give up this idea unfortunately due to various irresistible factors.

iv) I am surprised to find that the Phylogenetic analysis is performed using only 11 species when several high-quality genomes are available. This should be performed using more species, which can then be used as a good reference in future studies. Given the significance of this genome in SARS, a comprehensive phylogenetic analysis is much needed.
Reply: Thanks for your advice. We had reanalyzed phylogenetic analysis using 19 species. Phylogenetic relationship between civet and animals from Felidae, Canidae, Ursidae, Bovidae, Suidae, as well as human, mouse, and rabbit is clear now.

v) In case of positive selection analysis, pig (Sus scrofa) seems to be a comparatively distant species with respect to the other selected species. Inclusion of phylogenetically closer and a higher number of animal species will help in identification of the positively selected genes in this species with more precision and specificity.
Reply: Thanks for your advice! We have re-conducted positive selection analysis using Paguma larvata,C. lupus familiaris, and the five most closely relatedspecies from the Felidae familywith assembled genomes: F. catus, Panthera pardus, Panthera leo, L. canadensis, and Panthera tigris.

Close
