# Supplementary material for: The assembled and annotated genome of the masked palm civet (Paguma larvata)
Source: Gigascience. 2022 May 18;11:giac041. doi: 10.1093/gigascience/giac041 (PMC9116208; doi:10.1093/gigascience/giac041)
Supplement: giac041_Reviewer_1_Report_Original_Submission — Zhen Liu -- 11/5/2021 Reviewed [file giac041_reviewer_1_report_original_submission.pdf]

Reviewer Report

Title: The Assembled and Annotated Genome of the Masked Palm Civet (Paguma larvata)

Version: Original Submission **Date:** 11/5/2021

Reviewer name: Zhen Liu

Reviewer Comments to Author:

Liu et al. reported a chromosome-level genome assembly of Paguma larvata, which will provide a valuable genomic resource in investigating virus and host interactions because Paguma larvata was found to be an important host of SARS-Cov. The methods of genome assembly and gene prediction are appropriate; the continuity, completeness, gene number from the annotation of the Paguma larvata genome are also up to standard. Some suggestions are given for improving the quality of the manuscript.

1. To obtain reliable and correct results, the closely-related relatives of the masked palm civet, rather than Gallus, should be used as the outgroup in evolutionary analyses, especially the comparison of the evolutionary dynamics of gene families.
2. Because the authors simultaneously examined the positive selection of several genes, the results of statistical tests should be corrected by multiple testing.
3. The gene IDs used in Supplementary Table S8 are not the gene symbol name, and it is better to annotate the gene symbol names to facilitate data utilization.
4. Line 157-159: Given that only five species were used for the homology-based prediction, I suggested adding a couple of mammalian species with high genome annotation quality, such as Homo sapiens, in this analysis, which will be helpful to further improve the annotation quality.
5. Please provide the versions of the softwares and the parameters used in the Methods part.
6. line22ï¼Œ"with contig N50 and scaffold N50 values of 12.97 Mb, respectively" the authors seem to miss some words or values.

Level of Interest

Please indicate how interesting you found the manuscript: Choose an item.

Quality of Written English

Please indicate the quality of language in the manuscript: Choose an item.

Declaration of Competing Interests

Please complete a declaration of competing interests, considering the following questions:

- Have you in the past five years received reimbursements, fees, funding, or salary from an organisation that may in any way gain or lose financially from the publication of this manuscript, either now or in the future?
- Do you hold any stocks or shares in an organisation that may in any way gain or lose financially from the publication of this manuscript, either now or in the future?
- Do you hold or are you currently applying for any patents relating to the content of the manuscript?
- Have you received reimbursements, fees, funding, or salary from an organization that holds or has applied for patents relating to the content of the manuscript?
- Do you have any other financial competing interests?
- Do you have any non-financial competing interests in relation to this paper?

If you can answer no to all of the above, write 'I declare that I have no competing interests' below. If your reply is yes to any, please give details below.

I declare that I have no competing interests.

I agree to the open peer review policy of the journal. I understand that my name will be included on my report to the authors and, if the manuscript is accepted for publication, my named report including any attachments I upload will be posted on the website along with the authors' responses. I agree for my report to be made available under an Open Access Creative Commons CC-BY license (http://creativecommons.org/licenses/by/4.0/). I understand that any comments which I do not wish to be included in my named report can be included as confidential comments to the editors, which will not be published.

Choose an item.

To further support our reviewers, we have joined with Publons, where you can gain additional credit to further highlight your hard work (see: https://publons.com/journal/530/gigascience). On publication of this paper, your review will be automatically added to Publons, you can then choose whether or not to claim your Publons credit. I understand this statement.

Yes Choose an item.
