# Supplementary material for: The assembled and annotated genome of the masked palm civet (Paguma larvata)
Source: Gigascience. 2022 May 18;11:giac041. doi: 10.1093/gigascience/giac041 (PMC9116208; doi:10.1093/gigascience/giac041)
Supplement: giac041_Reviewer_2_Report_Original_Submission — Graham Hughes -- 11/19/2021 Reviewed [file giac041_reviewer_2_report_original_submission.pdf]

Reviewer Report

Title: The Assembled and Annotated Genome of the Masked Palm Civet (Paguma larvata)

Version: Original Submission **Date:** 11/19/2021

Reviewer name: Graham Hughes

Reviewer Comments to Author:

Reviewer's Comments
In this study, the authors provide a high-quality assembly of the Masked Palm Civet. This genome will be a valuable resource for comparative genomics. While the overall study is good, I felt that it lacked a cohesive narrative, with certain results either not making sense as written, or results being mentioned in passing but not explored further. I also found that certain paragraphs suffered from issues with grammar, such that the true meaning may be lost. Certain analyses e.g. RNA seq are mentioned, but never used to validate results. I believe there are major revisions needed for this study. Please see a list of major and minor comments below:
Minor comments
Line 44: The authors say posed as if it longer poses a threat, however I think this should be 'pose'.
Line 50: Saying in recent decades but that something used to be domesticated almost seemed contradictory to me, but that might just be me.
Line 90: I'm being a bit nit-picky here, but I feel like a final sentence or two explicitly stating why the genome was sequenced (e.g. to explore virus-host interactions) would be useful.
Line 122: I'm not sure if 'the FALCON' is the correct term to use here?
Line 139: you use the word 'using' twice in quick succession, perhaps consider something else?
Line 215: a "." Is missing on this line.
Line 300: How did the authors 'conduct' sequence identity? What is the importance of sequence identity compared to evidence of positive selection?
Major comments
1. You mention that you aligned protein sequences using blast, however blast is not an alignment software. Am I missing something?
2. What were the best-fit models of sequence evolution used with RAxML? Were they inferred? There are no mention of models used.
3. Were p-values from PAML corrected for multiple testing?
4. The authors mention downloading sequences of receptors under selection and ACE2 from other animal species. It would be helpful if they listed them here. Is there a reason why the authors use mafft here but used muscle previously?
5. I am a little confused about the statement on line 255. The authors say that 87 gene families are unique to P. larvata. I find this number very difficult to believe. This would suggest that 87 different families of genes have subsequently emerged since sharing a last common ancestor with the cat and tiger. I think there could be a wording issue here. But based on these gene numbers, and the divergence time quoted later (37.2 MY), that's one gene family per 427,586 with 'gene family' implying more than

one gene. Can the authors clarify this?
6. Was selection detected in ACE2? If not, what does this mean for SARS-CoV in civets? I feel that the authors have avoided this but should discuss it in the text. What implications does this have for the theory that viral receptors in the civet have undergone change to avoid infection?
7. The authors state that there were more protein coding genes in the civet than the tiger and cat. Do these genes come from the expansions of certain families, or are they completely absent in other species? Is it possible that alleles of genes from reverse strands have been incorporated in the genome? Do these extra genes show up in the RNA seq data?
8. Were any of the expanded immunoglobulin genes under selection? These seem to be mentioned once in the result but no more if mentioned about them.

Level of Interest

Please indicate how interesting you found the manuscript: Choose an item.

Quality of Written English

Please indicate the quality of language in the manuscript: Choose an item.

Declaration of Competing Interests

Please complete a declaration of competing interests, considering the following questions:

- Have you in the past five years received reimbursements, fees, funding, or salary from an organisation that may in any way gain or lose financially from the publication of this manuscript, either now or in the future?
- Do you hold any stocks or shares in an organisation that may in any way gain or lose financially from the publication of this manuscript, either now or in the future?
- Do you hold or are you currently applying for any patents relating to the content of the manuscript?
- Have you received reimbursements, fees, funding, or salary from an organization that holds or has applied for patents relating to the content of the manuscript?
- Do you have any other financial competing interests?
- Do you have any non-financial competing interests in relation to this paper?

If you can answer no to all of the above, write 'I declare that I have no competing interests' below. If your reply is yes to any, please give details below.

I declare that I have no competing interests

I agree to the open peer review policy of the journal. I understand that my name will be included on my report to the authors and, if the manuscript is accepted for publication, my named report including any attachments I upload will be posted on the website along with the authors' responses. I agree for my report to be made available under an Open Access Creative Commons CC-BY license (http://creativecommons.org/licenses/by/4.0/). I understand that any comments which I do not wish to be included in my named report can be included as confidential comments to the editors, which will not be published.

Choose an item.

To further support our reviewers, we have joined with Publons, where you can gain additional credit to further highlight your hard work (see: https://publons.com/journal/530/gigascience). On publication of this paper, your review will be automatically added to Publons, you can then choose whether or not to claim your Publons credit. I understand this statement.

Yes Choose an item.
