# Supplementary material for: The assembled and annotated genome of the masked palm civet (Paguma larvata)
Source: Gigascience. 2022 May 18;11:giac041. doi: 10.1093/gigascience/giac041 (PMC9116208; doi:10.1093/gigascience/giac041)
Supplement: giac041_Reviewer_2_Report_Revision_1 — Graham Hughes -- 2/25/2022 Reviewed [file giac041_reviewer_2_report_revision_1.pdf]

Reviewer Report

Title: The Assembled and Annotated Genome of the Masked Palm Civet (Paguma larvata)

Version: Revision 1 **Date:** 2/25/2022

Reviewer name: Graham Hughes

Reviewer Comments to Author:

Reviewer's comments
The authors have given strong responses to my comments, and have carried out additional analyses or modified sections as appropriate, which I really appreciate. My biggest concern was the number of genes unique to P. larvata relative to other carnivorans. My final comment relate specifically to how this is referred to in the text, and the validity of the statement:
The authors state "among the 19 species, 44 gene families containing 119 genes were unique to P. larvata."
I still think this reads a little confusing, as to me it gives the impression that 44 unique gene families were found. If the authors rearranged the sentence to something like "among the 19 species, 119 genes belonging to 44 gene families were found to be unique to P. larvata" or something similar, it might be less confusing. I'm still not 100% convinced that these 119 genes are wholly unique to the P. larvata genome. The authors mention that perhaps they are simply not annotated in closely related species enough to be flagged as orthologs. If the authors map these 119 genes to the genome, for example by comparing them against the raw genome files of some closely related carnivorans via blastn, do they hit anywhere in the genome, and if so, do those loci regions being hit contain any CDS annotations in the RefSeq genome file? I realise this is a bit of a sticking point with me, but perhaps even if the authors can acknowledge whether or not this number of unique genes is a sure thing, or could be due to poor annotations explicitly in the text, it might help.

Level of Interest

Please indicate how interesting you found the manuscript: Choose an item.

Quality of Written English

Please indicate the quality of language in the manuscript: Choose an item.

Declaration of Competing Interests

Please complete a declaration of competing interests, considering the following questions:

- Have you in the past five years received reimbursements, fees, funding, or salary from an organisation that may in any way gain or lose financially from the publication of this manuscript, either now or in the future?
- Do you hold any stocks or shares in an organisation that may in any way gain or lose financially from the publication of this manuscript, either now or in the future?
- Do you hold or are you currently applying for any patents relating to the content of the manuscript?
- Have you received reimbursements, fees, funding, or salary from an organization that holds or has applied for patents relating to the content of the manuscript?
- Do you have any other financial competing interests?
- Do you have any non-financial competing interests in relation to this paper?

If you can answer no to all of the above, write 'I declare that I have no competing interests' below. If your reply is yes to any, please give details below.

I declare that I have no competing interests

I agree to the open peer review policy of the journal. I understand that my name will be included on my report to the authors and, if the manuscript is accepted for publication, my named report including any attachments I upload will be posted on the website along with the authors' responses. I agree for my report to be made available under an Open Access Creative Commons CC-BY license (http://creativecommons.org/licenses/by/4.0/). I understand that any comments which I do not wish to be included in my named report can be included as confidential comments to the editors, which will not be published.

Choose an item.

To further support our reviewers, we have joined with Publons, where you can gain additional credit to further highlight your hard work (see: https://publons.com/journal/530/gigascience). On publication of this paper, your review will be automatically added to Publons, you can then choose whether or not to claim your Publons credit. I understand this statement.

Yes Choose an item.
