# Supplementary material for: The assembled and annotated genome of the masked palm civet (Paguma larvata)
Source: Gigascience. 2022 May 18;11:giac041. doi: 10.1093/gigascience/giac041 (PMC9116208; doi:10.1093/gigascience/giac041)
Supplement: giac041_Reviewer_3_Report_Original_Submission — Vineet K. Sharma -- 11/26/2021 Reviewed [file giac041_reviewer_3_report_original_submission.pdf]

Reviewer Report

Title: The Assembled and Annotated Genome of the Masked Palm Civet (Paguma larvata)

Version: Original Submission **Date:** 11/26/2021

Reviewer name: Vineet K. Sharma

Reviewer Comments to Author:

The manuscript by Liu et el reports the assembled and annotated genome of the masked Palm Civet (Paguma larvata) which is a good contribution given the role of this organism in acting as an intermediate
host of severe acute respiratory syndrome coronavirus (SARS-CoV). The assembly is performed well and is of acceptable quality, however I have some major comments on the analysis part which are mentioned below.

i) The reported genome assembly of Paguma larvata is of good reference quality achieved using short read, long read and Hi-C technology. But despite of having an N50 value of 12.97 Mb, the BUSCO completeness is only 95 % which much lower than expected. Do the authors have an explanation for it or I suggest that it should be carried out again.

ii) The methodology for prediction of non-coding RNAs should be mentioned in the main manuscript. In line 248-250, I noted that the number of predicted miRNA, tRNA genes seems to be much higher in this genome, which should be carefully checked.

iii) RNA-Seq data was generated from seven different tissues of Paguma larvata. This amount of data can be used for more detailed analysis e.g., differential gene expression profile of immunity-related genes in these different tissues. This analysis will add more value to the work.

iv) I am surprised to find that the Phylogenetic analysis is performed using only 11 species when several high-quality genomes are available. This should be performed using more species, which can then be used as a good reference in future studies. Given the significance of this genome in SARS, a comprehensive phylogenetic analysis is much needed.

v) In case of positive selection analysis, pig (Sus scrofa) seems to be a comparatively distant species with respect to the other selected species. Inclusion of phylogenetically closer and a higher number of animal species will help in identification of the positively selected genes in this species with more precision and specificity.

Level of Interest

Please indicate how interesting you found the manuscript: Choose an item.

Quality of Written English

Please indicate the quality of language in the manuscript: Choose an item.

Declaration of Competing Interests

Please complete a declaration of competing interests, considering the following questions:

- Have you in the past five years received reimbursements, fees, funding, or salary from an organisation that may in any way gain or lose financially from the publication of this manuscript, either now or in the future?
- Do you hold any stocks or shares in an organisation that may in any way gain or lose financially from the publication of this manuscript, either now or in the future?
- Do you hold or are you currently applying for any patents relating to the content of the manuscript?
- Have you received reimbursements, fees, funding, or salary from an organization that holds or has applied for patents relating to the content of the manuscript?
- Do you have any other financial competing interests?
- Do you have any non-financial competing interests in relation to this paper?

If you can answer no to all of the above, write 'I declare that I have no competing interests' below. If your reply is yes to any, please give details below.

I declare that I have no competing interests' below

I agree to the open peer review policy of the journal. I understand that my name will be included on my report to the authors and, if the manuscript is accepted for publication, my named report including any attachments I upload will be posted on the website along with the authors' responses. I agree for my report to be made available under an Open Access Creative Commons CC-BY license (http://creativecommons.org/licenses/by/4.0/). I understand that any comments which I do not wish to be included in my named report can be included as confidential comments to the editors, which will not be published.

Choose an item.

To further support our reviewers, we have joined with Publons, where you can gain additional credit to further highlight your hard work (see: https://publons.com/journal/530/gigascience). On publication of this paper, your review will be automatically added to Publons, you can then choose whether or not to claim your Publons credit. I understand this statement.

Yes Choose an item.
