# Supplementary material for: The assembled and annotated genome of the masked palm civet (Paguma larvata)
Source: Gigascience. 2022 May 18;11:giac041. doi: 10.1093/gigascience/giac041 (PMC9116208; doi:10.1093/gigascience/giac041)
Supplement: giac041_Supplemental_Files [file giac041_supplemental_files.zip › Commonts-20220310.docx]

Reviewer reports:

Reviewer #1: The authors have fully addressed my concerns. I have no more comments about this revised manuscript.

Reviewer #2: Reviewer's comments

The authors have given strong responses to my comments, and have carried out additional analyses or modified sections as appropriate, which I really appreciate. My biggest concern was the number of genes unique to P. larvata relative to other carnivorans. My final comment relate specifically to how this is referred to in the text, and the validity of the statement:

The authors state "among the 19 species, 44 gene families containing 119 genes were unique to P. larvata."

I still think this reads a little confusing, as to me it gives the impression that 44 unique gene families were found. If the authors rearranged the sentence to something like "among the 19 species, 119 genes belonging to 44 gene families were found to be unique to P. larvata" or something similar, it might be less confusing. I'm still not 100% convinced that these 119 genes are wholly unique to the P. larvata genome. The authors mention that perhaps they are simply not annotated in closely related species enough to be flagged as orthologs. If the authors map these 119 genes to the genome, for example by comparing them against the raw genome files of some closely related carnivorans via blastn, do they hit anywhere in the genome, and if so, do those loci regions being hit contain any CDS annotations in the RefSeq genome file? I realise this is a bit of a sticking point with me, but perhaps even if the authors can acknowledge whether or not this number of unique genes is a sure thing, or could be due to poor annotations explicitly in the text, it might help.

Reply: Thanks for your advice! We have rearranged the sentence from “among the 19 species, 44 gene families containing 119 genes were unique to P. larvata” to “Among the 19 species, 119 genes belonging to 44 gene families were unique in P. larvata” in line 296-297, and we are sorry about this mistake. We will try to explain this question in three aspects: Firstly, before phylogenetic orthology inference, we filtered out genes in other 18 species with missing data, and did not start with initiation codon, this step may be deleted some genes which may be potentially in the same orthologs with unique genes in P. larvata. secondly, the unique genes were determined by software algorithms. It's worth noting that results using different versions of OrthoFinder have some differences. In our paper, we used OrthoFinder version 2.5.2 to conduct phylogenetic orthology inference, and 119 unique genes were detected. Actually, we also used OrthoFinder version 2.2.7, and 6 unique genes were detected. Both analysis were conducted twice, and the results were the same. Finally we chose the results of advanced version of OrthoFinder, which showed 119 genes were unique in P. larvata. Thirdly, we must point out that these unique genes just “unique” in P. larvata compared to other 18 species in our study. Just as reviewer said, we blasted several sequences of these unique genes, some of which could hit in other species. We searched other studies, and found out the unique genes were also detected in other species (doi: 10.1093/gigascience/giab027; doi: 10.1093/gigascience/giab016; doi: 10.1093/gigascience/giy108; doi: 10.1093/gigascience/giy094). Therefore, we think the results of our study are credible, and we also appreciated your precious advice.
