# Supplementary material for: The assembled and annotated genome of the masked palm civet (Paguma larvata)
Source: Gigascience. 2022 May 18;11:giac041. doi: 10.1093/gigascience/giac041 (PMC9116208; doi:10.1093/gigascience/giac041)
Supplement: giac041_Supplemental_Files [file giac041_supplemental_files.zip › cover letter.docx]

Dear Editors:

We would like to submit the revised manuscript entitled “The Assembled and Annotated Genome of the Masked Palm Civet (*Paguma larvata*)”, which we wish to be considered for publication in “Gigascience”. No conflict of interest exits in the submission of this manuscript, and manuscript is approved by all authors for publication. I would like to declare on behalf of my co-authors that the work described was original research that has not been published previously, and not under consideration for publication elsewhere, in whole or in part. All the authors listed have approved the manuscript that is enclosed.

The masked palm civet (*Paguma larvata*) acts as an intermediate host of severe acute respiratory syndrome coronavirus (SARS-CoV), which caused SARS, and transfers this virus from bats to humans. Additionally, *P. larvata* has the potential to carry a variety of zoonotic viruses that may threaten human health. However, genome resources for *P. larvata* have not been reported to date. A chromosome-level genome assembly of *P. larvata* was generated using PacBio sequencing, Illumina sequencing, and Hi-C technology. The genome assembly was 2.44 Gb in size, of which 95.32% could be grouped into 22 pseudochromosomes, with contig N50 and scaffold N50 values of 12.97 Mb and 111.87 Mb, respectively. A total of 21,582 protein-coding genes were predicted, and 95.20% of the predicted genes were functionally annotated. Phylogenetic analysis of 19 animal species confirmed the close genetic relationship between *P. larvata* and species belong to the Felidae family. Gene family clustering revealed 119 unique, 362 significantly expanded, and 58 significantly contracted genes in the *P. larvata* genome. We identified 971 positively selected genes in *P. larvata*, and one known human viral receptor gene *PDGFRA* is positively selected in *P. larvata*, which is required for human cytomegalovirus (HCMV) infection, This high-quality genome assembly provides a valuable genomic resource for exploring virus-host interactions . It will also provide a reliable reference for studying the genetic bases of the morphological characteristics, adaptive evolution, and evolutionary history of this species.

Thanks for the valuable advices from reviewers, we have completed the revision of our manuscript. I hope this paper is suitable for “Gigascience” now. We deeply appreciate your consideration of our manuscript. If you have any queries, please don’t hesitate to contact me at the address below.

Thank you and best regards.

Yours sincerely,

Ping Liu

Corresponding author:

Name: Jin-Ping Chen

E-mail: chenjp@giz.gd.cn
